# Transcriptome Analysis and Identification of Chemosensory Genes in *Baryscapus dioryctriae* (Hymenoptera: Eulophidae)

**DOI:** 10.3390/insects13121098

**Published:** 2022-11-29

**Authors:** Xiaoyan Zhu, Qiling Yu, Xingyu Gan, Liwen Song, Kaipeng Zhang, Tongtong Zuo, Junjie Zhang, Ying Hu, Qi Chen, Bingzhong Ren

**Affiliations:** 1 Jilin Provincial Key Laboratory of Animal Resource Conservation and Utilization, School of Life Sciences, Northeast Normal University, Changchun 130024, China; 2 Key Laboratory of Vegetation Ecology, MOE, Northeast Normal University, Changchun 130024, China; 3 Jilin Provincial Engineering Laboratory of Avian Ecology and Conservation Genetics, Northeast Normal University, Changchun 130118, China; 4 Research Institute of Forest Protection, Jilin Provincial Academy of Forestry Sciences, Changchun 130033, China; 5 Engineering Research Center of Natural Enemies, Institute of Biological Control, Jilin Agricultural University, Changchun 130118, China

**Keywords:** *Baryscapus dioryctriae*, Eulophidae, wasp, transcriptome, chemosensory genes

## Abstract

**Simple Summary:**

The parasitic wasp *Baryscapus dioryctriae* (Hymenoptera: Eulophidae) was originally discovered in the pupae of *Dioryctria* insects. It can also parasitize many Pyralidae pests, such as *Ostrinia furnacalis*, *Chilo suppressalis* and *Galleria mellonella*, suggesting that this parasitic wasp has a great potential to serve as a natural enemy against agricultural and forest insect pests. The olfactory system plays an important role in this wasps’ reception of chemical signals emitted from their surrounding environment, and various chemosensory genes are involved in this system. In this study, seven chemosensory gene families, namely, the odorant-binding proteins (OBPs), chemosensory proteins (CSPs), Niemann–Pick type C2 proteins (NPC2s), odorant receptors (ORs), ionotropic receptors (IRs), gustatory receptors (GRs) and sensory neuron membrane proteins (SNMPs) of *B. dioryctriae* were identified and analyzed using transcriptome sequencing and bioinformatic analyses. Moreover, the quantitative expression of the candidate chemosensory genes, which are female antennae- and ovipositor-biased expression was validated by RT-qPCR. These results lay a molecular foundation for further investigation of the mechanism of chemoreception by the olfactory system during host recognition, location, and oviposition site selection in *B. dioryctriae*.

**Abstract:**

*Baryscapus dioryctriae* is a pupal endoparasitoid of many Pyralidae pests and has been used as a biocontrol agent against insect pests that heavily damage the cone and seed of the Korean pine. The olfactory system of wasps plays an essential role in sensing the chemical signals during their foraging, mating, host location, etc., and the chemosensory genes are involved in detecting and transducing these signals. Many chemosensory genes have been identified from the antennae of Hymenoptera; however, there are few reports on the chemosensory genes of Eulophidae wasps. In this study, the transcriptome databases based on ten different tissues of *B. dioryctriae* were first constructed, and 274 putative chemosensory genes, consisting of 27 OBPs, 9 CSPs, 3 NPC2s, 155 ORs, 49 GRs, 23 IRs and 8 SNMPs genes, were identified based on the transcriptomes and manual annotation. Phylogenetic trees of the chemosensory genes were constructed to investigate the orthologs between *B. dioryctriae* and other insect species. Additionally, twenty-eight chemosensory genes showed female antennae- and ovipositor-biased expression, which was validated by RT-qPCR. These findings not only built a molecular basis for further research on the processes of chemosensory perception in *B. dioryctriae*, but also enriched the identification of chemosensory genes from various tissues of Eulophidae wasps.

## 1. Introduction

Parasitic wasps play an essential role in maintaining ecological balance, and many of taxa of parasitic wasps have been applied in green pest management. Numerous studies have revealed that the chemical volatiles emitted from their living environment are important cues used by wasps to locate hosts and select oviposition sites [1,2], and their sophisticated olfactory systems are responsible for detecting and recognizing these chemicals. The chemosensory genes expressed in the organs with olfactory functions in wasps are involved in the initial transduction of chemical signals [3], and they mainly include members of three water-soluble protein families (the odorant-binding proteins (OBPs) [4], chemosensory proteins (CSPs) and Niemann–Pick type C2 proteins (NPC2s) families [5]) and four transmembrane protein families (the odorant receptors (ORs) [6], ionotropic receptors (IRs) [7], gustatory receptors (GRs) [8] and sensory neuron membrane proteins (SNMPs) families [9]). In general, the olfaction of insects occurs as follows: When hydrophobic (fat-soluble) odorant/nonvolatile compounds are detected, the water-soluble proteins (OBPs, CSPs or NPC2s) will bind and transport these chemicals through the sensillum lymph to the receptor neuron membranes [4,10,11], and the chemicals specifically interact with the transmembrane receptor proteins (ORs, IRs or GRs). Then, the chemical signals are transformed into electric signals (nerve impulses) to reach the brain [12,13]. SNMPs are associated with sex pheromone perception in moths [5], but little is known about them in wasps. Additionally, some studies have revaled that odorant-degrading enzymes (ODEs) are involved in the inactivation of chemicals to protect the insects from continuous stimulation. Considering that ODEs are a functional category of enzymes comprised of several distinct gene families, such as cytochrome P450 (p450s), carboxylesterases (CCEs/CXEs), alpha-esterases, aldehyde oxidase (AOXs), glutathione S-transferases (GSTs) and alcohol dehydrogenases (ADs), the identification of ODEs is not included in this study but will be presented in our future research [14,15].

High-throughput sequencing technologies have developed rapidly over the past decade [16], and have been widely used to identify chemosensory genes in many insects [17]. In Hymenoptera, an enormous number of chemosensory genes from various species have been reported, and most of them have been identified through antennal transcriptomes. According to our statistics from Insectbase 2.0 [18] and the NCBI (National Center for Biotechnology Information (nih.gov)) (accessed on 10 September 2022), chemosensory genes from approximately 4 superfamilies, 18 families and 89 species have been identified, such as *Trichogramma pretiosum* [19], *Anagrus nilaparvatae* [20,21], *Aenasius bambawalei* [22] and *Diachasmimorpha longicaudata* [23]. In the past five years, an increasing number of studies have investigated the function of chemosensory genes. For example, TjapOBP2 of *Trissolcus japonicus* binds to the alarm pheromone (E)-2-decenal which is emitted from its host *Halyomorpha halys* [24]. EforCSP3 of *Encarsia formosa* exhibits high binding affinity for a variety of host-associated volatiles (such as dibutyl phthalate, 1-octene and β-elemene) [25]. McinNPC2 of *Macrocentrus cingulum* recognizes an aromatic compound, β-ionone, that is commonly found in essential oils [26]. MmedOR49 of *Microplitis mediator* was specifically tuned to the major sex pheromone component (cis-5-decenyl acetate) of *Agrotis segetum,* and female wasps could be significantly attracted by this component [27]. IR64a1 and IR64a2 of *M. mediator* cooperatively sense habitat and host cues to help the parasitoids locate their host efficiently [28]. Moreover, the physical protein structure of the parasitic wasp *Apocrypta bakeri* Orco (SMTL ID: 6c70.1. A) has been investigated by CryoEM in recent years [29], which is a landmark for clarifying how the architecture of the OR family accommodates its remarkable sequence diversity and facilitates the evolution of odor tuning. However, compared with the studies on Lepidoptera, Diptera and Coleoptera insects, the number of functional investigations on chemosensory genes in Hymenoptera wasps is still relatively few, and the gene expansions of some families (such as ORs and SNMPs) might cause difficulty in screening the candidates for further functional research. Above all, the chemosensory genes of parasitic wasps play a crucial role in their chemical communication and regulation of their reproductive behaviors. Therefore, it is important to clarify the molecular mechanisms underlying the olfactory recognition processes during their life activities by identifying and investigating the chemosensory genes in parasitic wasps [23]; this is instrumental in promoting pest control by natural enemies at the olfactory regulation level.

*Baryscapus dioryctriae*, a parasitic wasp that was originally found in the pupae of *Dioryctria pryeri* and the coneworm *Dioryctria abietella* [30], was later cultivated under experimental conditions and found to be parasitic on a variety of Pyralidae pests, such as the greater wax moth *Galleria mellonella*, the European corn borer *Pyrausta nubilalis*, and the rice stem borer *Chilo suppressalis*. *B. dioryctriae* has been applied to control *Dioryctria* pests in the field for many years, and previous studies are mainly focused on how to improve their reproductive ability. Recently, the morphology and ultrastructure of antennal sensilla of *B. dioryctriae* have been investigated via scanning and transmission electron microscopy by our research group [31], and our results indicated that sensilla trichodea and placodea might be involved in chemical perception in this parasitic wasp. However, the molecular mechanism underlying chemosensory perception in *B. dioryctriae* remains unknown.

In this study, we sequenced the transcriptomes of ten tissues of *B. dioryctriae*, including female antennae (FA), male antennae (MA), female ovipositors (Fov), male genitalia (Mge), female heads without antennae (FH), male heads without antennae (MH), female abdomens without the ovipositors and digestive tracts (Fab), male abdomens without the genitalia and digestive tracts (Mab), a mix of male and female thoraxes (T) and a mix of male and female legs (L), using the Illumina sequencing platform. Then, the putative chemosensory genes of *B. dioryctriae* were identified based on the above transcriptome datasets along with manual annotation, and phylogenetic analyses of OBPs, CSPs, NPC2s, SNMPs, ORs, IRs and GRs were also performed based on other Hymenoptera and model insects. Furthermore, the expression levels of these genes were calculated by Fragments Per Kilobase of exon model per Million mapped fragments (FPKM), and the quantitative expression patterns of the candidate chemosensory genes among the ten different tissues were validated by RT-qPCR. After that, the chemosensory genes with female antennae- and ovipositor-biased expression were selected as target genes to be used for our future study. This study aims to provide a gene foundation for Eulophidae wasps and further investigate the chemoreceptor mechanism underlying host recognition/location and ovipositor site selection of *B. dioryctriae*.

## 2. Materials and Method

### 2.1. Insect Culture and Tissue Collection

The *B. dioryctriae* individuals used in this study were provided by the Research Institute of Forest Protection, Jilin Provincial Academy of Forestry Sciences. The pupae provided for parasitism (*G. mellonella* pupae) were kept in a climatic chamber at 25 ± 1 °C, L:D = 16 h:8 h, 50 ± 5% relative humidity (RH) and checked daily for adult emergence. After eclosion, these wasps were fed 10% (V/V) sucrose solution and reared under the same conditions as above. To obtain relatively comprehensive chemosensory genes of *B. dioryctriae*, the following ten tissues of adult wasps were collected under a stereomicroscope (LJ-SZM, Leica, Germany): female antennae (FA, 1200 females), male antennae (MA, 1200 males), female ovipositors (Fov, 1200 females), male genitalia (Mge, 1200 males), female heads without antennae (FH, 500 females), male heads without antennae (MH, 500 males), female abdomens without ovipositors and digestive tracts (Fab, 500 females), male abdomens without genitalia and digestive tracts (Mab, 500 males), thoraxes (T, 500 males and females) and legs (L, 500 males and females). All samples were immediately frozen in liquid nitrogen and stored at −80 °C until RNA extraction.

### 2.2. RNA-Seq Library Construction and Sequencing

Total RNA was extracted using TRIzol reagent (Invitrogen, Carlsbad, CA, USA) following the manufacturer’s protocol, and the integrity of the RNA was determined by a NanoDrop 2000 (Thermo Fisher Scientific, Waltham, MA) and 1% agarose gel electrophoresis [32].

The quantified RNA extracted from the ten samples (FA, FH, Fab, Fov, MA, MH, Mab, Mge, T and L) was sent to Biomarker Technologies (Beijing, China) for building Illumina sequencing libraries, and a single sample per tissue-set type was sequenced. Sequencing libraries were prepared using the NEBNext^®^ Ultra™ RNA Library Prep Kit for Illumina^®^ (NEB, USA) according to the manufacturer’s recommendations, and index codes were added to attribute sequences to each sample. The libraries were validated using the Agilent Bioanalyzer 2100 system to check the quality. According to the manufacturer’s instructions, the clustering of the index-coded samples was performed on a cBot Cluster Generation System using TruSeq PE Cluster Kit v3-cBot-HS (Illumina). After cluster generation, based on sequencing-by-synthesis (SBS) technology, the libraries were sequenced on an Illumina HiSeq 2000 PE150 platform, and then the 150 bp paired-end reads were generated.

### 2.3. Transcriptome Assembly and Annotation of Functional Genes

To obtain the raw data (raw reads) in fastq format, first, the reads containing adapters, reads containing poly-N and reads of low quality were removed through in-house Perl scripts from the raw data. In addition, the Q20, Q30, GC content and sequence duplication level of the clean data were calculated. All downstream analyses were based on clean data with high quality. The left.fq and right.fq functions in Trinity (r20131110) were applied with all default parameters for transcriptome assembly [33], and all the reads from all the samples were assembled into one transcriptome. The Trinity_cluster_with_id_com Pl was used for clustering the transcripts, and unigenes were defined as the longest isoform from each trinity assembly, then the final unigene dataset was generated.

Gene function was annotated based on the following databases: NR (NCBI nonredundant protein sequences), Pfam (Protein family); KOG/COG/eggNOG (Clusters of Orthologous Groups of proteins), Swiss-Prot (a manually annotated and reviewed protein sequence database), KEGG (Kyoto Encyclopedia of Genes and Genomes) and GO (Gene Ontology).

### 2.4. Identification and Structural Analysis of Putative Chemosensory Genes

The seven putative chemosensory gene families of *B. dioryctriae* were preliminarily screened based on the annotation results of the transcriptome databases. In order to maximize identification of all putative chemosensory genes, we also made a manual annotation via the Basic Local Alignment Search Tool (ncbi-blast-2.11.0). All the sequences referenced from model species and other closely related species are listed in the Supplementary Materials (Files S1–S7). Based on the final annotation results, the ORF finder tool (https://www.ncbi.nlm.nih.gov/orffinder/) (accessed on 10 September 2022) was used to predict the open reading frame (ORF) and amino acid sequence of each gene transcript. Then, the obtained sequences were submitted to NCBI BLAST (https://blast.ncbi.nlm.nih.gov/Blast.cgi) (accessed on 10 September 2022) and all putative chemosensory genes were manually rechecked against the GenBank nonredundant (NR) protein database using the BLASTx program (E-value < 10^−5^).

For putative water-soluble proteins (OBPs, CSPs and NPC2s) of *B. dioryctriae*, the N-terminal signal peptides were predicted using the SignalP-5.0 server (https://services.healthtech.dtu.dk/service.php?SignalP-5.0) (accessed on 10 September 2022); the molecular weights (MWs) and isoelectric points (pIs) were obtained using the ExPASy proteomics server (https://www.expasy.org/resources/compute-pi-mw) (accessed on 10 September 2022). Sequence alignments were performed using DNAMAN-7 with default gap penalty parameters of 10 for gap opening and 5 for extension. For putative transmembrane proteins (ORs, IRs, GRs and SNMPs) of *B. dioryctriae*, the transmembrane domains were predicted using the TOPCONS online server (https://topcons.cbr.su.se/) (accessed on 10 September 2022).

### 2.5. Phylogenetic Analysis

Phylogenetic trees were constructed for the analyses of BdioOBPs, BdioCSPs, Bdio NPC2s, BdioORs, BdioIRs, BdioGRs and BdioSNMPs using their amino acid sequences along with the sequences from other insects. All the amino acid sequences used in this study are presented in the Supplementary Materials (Files S1–S7). Using the ”One Step Build a ML Tree” function of TBtools [34], Muscl was called for multiple sequence alignment, TrimAl was used to prune the alignment results, and finally, IQ-tree was called to automatically screen the amino acid replacement model to build the maximum likelihood (ML) trees. Then, the trees were visualized with FigTree v1.4.2.

### 2.6. Differential Expression Gene Analysis

To screen the chemosensory genes with female antennae- and ovipositor-biased expression, which are likely to be involved in host recognition/location and oviposition site selection in *B. dioryctriae*, we performed fifteen comparisons between the nonolfactory tissues (from heads, thoraxes, legs, abdomens, and male genitalia) and tissues with olfactory function (from antennae and ovipositors) (Table 1). After using the EBSeq program package to adjust the read counts through an empirical Bayesian approach, according to the group settings in Table 1, the EBSeq program package was used to conduct differential expression analysis with the conditions q value < 0.005 &|log2 (fold change) > 2 [35].

### 2.7. Heatmap Construction and RT-qPCR Validation

The reads obtained by sequencing were aligned with the unigene dataset using Bowtie [36], and the expression level was evaluated in combination with RSEM [37]. The Fragments Per Kilobase of exon model per Million mapped fragments (FPKM) was used to represent the expression abundance of the corresponding gene transcripts [38,39]. The OmicShare tool (https://www.omicshare.com) (accessed on 10 September 2022) was used to construct heatmaps of differentially expressed chemosensory genes using log_10_ FPKM values.

Based on the results of the differential gene expression analysis and with the conditions q value < 0.005 &|log2 (fold change) > 2, the chemosensory genes with female antennae- and ovipositor-biased expression were preliminarily selected out as candidate genes, and the quantitative expression levels of these candidates in the ten different tissues were validated by real-time quantitative PCR (RT-qPCR) measurement. In this assay, the individual numbers and conditions used for total RNA extraction from the ten tissues were similar to that of Section 2.1, and an equal amount (1 μg) of total RNA from each sample was transcribed into cDNA by TransScript One-Step gDNA Removal and cDNA Synthesis SuperMix (Transgen Biotech, Beijing, China). The *BdioGAPDH* gene (glyceraldehyde-3-phosphate dehydrogenase) was used as the reference gene (internal control) to normalize the expression data. Specific primers were designed using Primer 3 (https://bioinfo.ut.ee/primer3-0.4.0/) (accessed on 10 September 2022) and are listed in Table S11. Then, qPCR measurements were performed using the LightCycler 480 II Detection System (Roche, Shanghai, China) and TransStar Tip Top Green qPCR Supermix (Transgen Biotech, Beijing, China). Each qPCR reaction was used according to the manufacturer’s instructions, and the conditions were as follows: 94 °C for 30 s, followed by 45 cycles of 94 °C for 5 s, 55 °C for 15 s, and 72 °C for 10 s. Then, 95 °C for 5 s, 65 °C for 1 min, 97 °C for 10 s, and 60 °C for 15 s to measure the melt curve. The relative expression levels of samples were calculated using the comparative 2^−ΔΔCT^ method. All qPCRs were conducted in three technical and three biological replicates.

## 3. Results

### 3.1. The Transcriptome of Various Tissues in B. dioryctriae

De novo transcriptomes were derived from ten tissue samples of *B. dioryctriae,* a total of 62.99 Gb of clean data was obtained, with each sample yielding 6.05 Gb, the percentage of Q30 bases was 91.42% and above, and the GC content of each tissue ranged from 39.09% to 42.82% (Table 2). A total of 271,986 transcripts and 47,607 unigenes were assembled, of which 14,994 unigenes (31.49%) were longer than 1000 bp (Figure S1). The N50s of the transcripts and unigenes were 6428 bp and 2801 bp, respectively (Table S1). All the transcriptome data were uploaded to the NCBI SRA (SRR21665100-SRR21665109).

Sequence similarity searches against nine public databases (NR, Swiss-Prot, TrEMBL, KEGG, COG, KOG, GO, eggNOG and Pfam) were used for sequence annotation. The results showed that a total of 20,982 (44.08%) unigenes were successfully annotated in these databases, of which 19,451 (40.86%), 18,736 (39.35%) and 15,996 (33.60%) were significantly matched in the TrEMBL, NR and GO databases, respectively, whereas 26,625 (55.92%) were unmapped to those databases (Table S2).

### 3.2. Identification of Putative Water-Soluble Proteins

**Figure 1:**
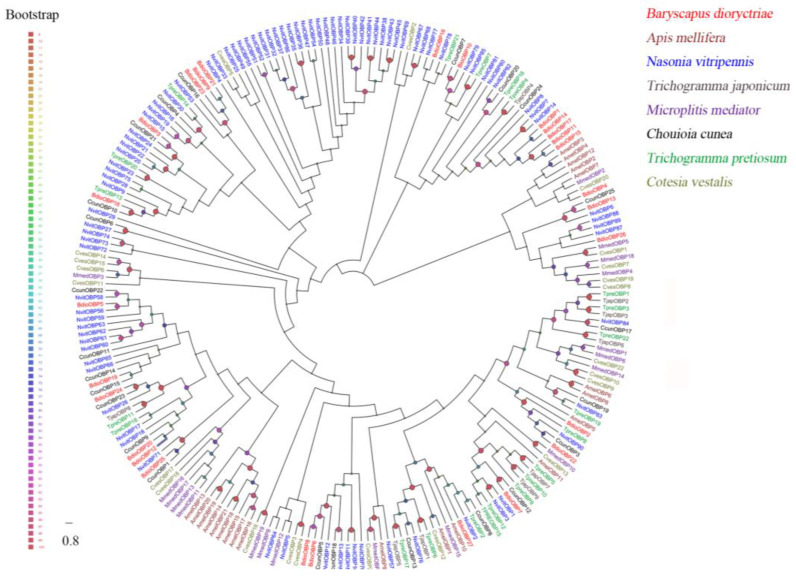
Phylogenetic tree of 233 odorant-binding proteins (OBPs) from *B. dioryctriae* and seven other Hymenoptera species. The protein sequences used in this phylogenetic analysis are listed in File S1. The size and color of the dots reflect their bootstrap values. The color scale is shown on the left, with larger dots indicating larger bootstrap values.

A total of 27 putative OBPs were identified in *B. dioryctriae*, encoding 83–153 amino acid residues with molecular weights ranging from 8.76 Kd to 16.24 Kd. The homology at the amino acid level with known insect OBPs was 36.94–95.41%, and within these OBPs, fifteen *BdioOBPs* (OBP1-3/6-8/10-15/17-18/22) were predicted to be full-length (Table S3). Multiple alignment results showed that 13 BdioOBPs (OBP1-2/6-8/10-15/17/22) contained six conserved cysteine residues (X11-37-Cys1-X20-27-Cys2-X3-Cys3-X26-49-Cys4-X8-11-Cys5-X8-Cys6-X5-18) and were characterized as classic OBPs (Figure S1). An ML tree was constructed using the OBPs of *B. dioryctriae* along with seven other Hymenoptera species. The results showed that many BdioOBPs were highly differentiated into different clades, and the BdioOBPs were closely clustered with NvitOBPs *(Nasonia vitripennis*) and CcunOBPs (*Chouioia cunea*) in multiple clades (Figure 1).

**Figure 2:**
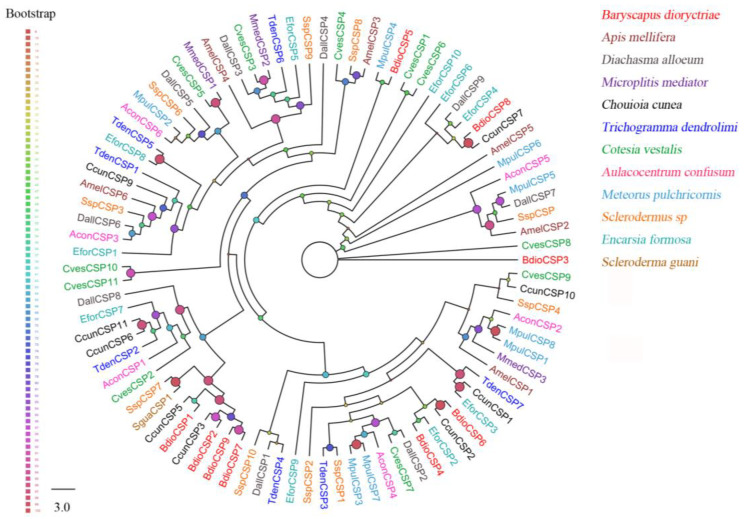
Phylogenetic tree of 90 chemosensory proteins (CSPs) from *B. dioryctriae* and 11 other Hymenoptera species. The protein sequences used in this phylogenetic analysis are listed in File S2. The size and color of the dots reflect their bootstrap values. The color scale is shown on the left, with larger dots indicating larger bootstrap values.

Ten putative *CSPs* were identified in *B. dioryctriae*, encoding 89–156 amino acid residues with molecular weights ranging from 10.23 Kd to 17.82 Kd. The homology between BdioCSPs and known insect CSPs at the amino acid level was 38.89–83.53% (Table S4). Six *BdioCSPs* (CSP1, 2, 4–6, 8) were predicted to be full-length, and all of them had four conserved cysteine sites (X38-75-Cys1-X6-8-Cys2-X18-19-Cys3-X2-Cys4-X37-51) (Figure S2). Additionally, the ML tree based on 90 CSPs from *B. dioryctriae* along with 11 other insect species showed that most BdioCSPs (CSP1–2, 6–9) were closely related to CcunCSP (*C. cunea*) and clustered together into the same clade (Figure 2). BdioCSP4 and EforCSP2 (*E. Formosa*), as well as BdioCSP5 and MpulCSP4 (*Meteorus pulchricornis*), were closely clustered, respectively. Only BdioCSP3 was independent of all other parasitoid CSPs.

**Figure 3:**
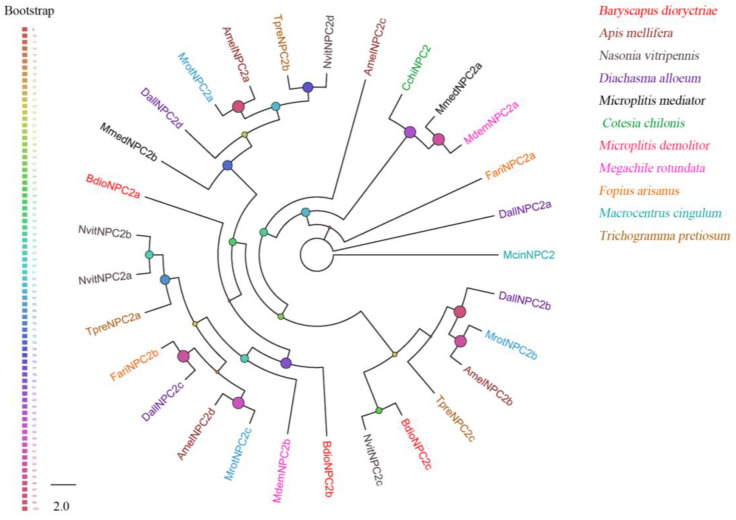
Phylogenetic tree of 29 Niemann–Pick type C2 proteins (NPC2s) from *B. dioryctriae* and other 10 Hymenoptera species. The protein sequences used in this phylogenetic analysis are listed in File S3. The size and color of the dots reflect their bootstrap values. The color scale is shown on the left, with larger dots indicating larger bootstrap values.

Three putative NPC2s were identified with full length in *B. dioryctriae*, encoding 149–178 amino acid residues with molecular weights ranging from 15.81 Kd to 20.16 Kd, and the homology with other known insect NPC2s at the amino acid level was 59.06–85.5% (Table S5). The results of multiple alignment showed that all three NPC2s have six conserved cysteines (X20-24-Cys1-X13-14-Cys2-X4-5-Cys3-X43-45-Cys4-X6-13-Cys5-X40-42-Cys6-X8-33) (Figure S3). BdioNPC2c formed a clade with NvitNPC2c (*N. vitripennis*), but BdioNPC2a and BdioNPC2b were far from the other clades (Figure 3).

### 3.3. Identification of Putative Transmembrane Proteins

**Figure 4:**
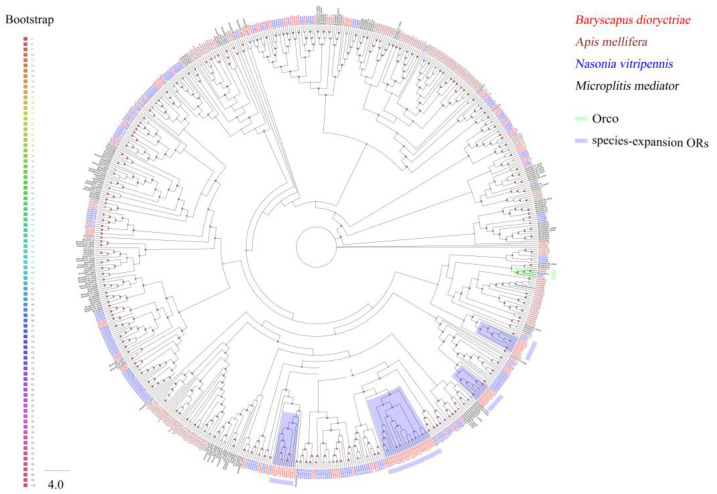
Phylogenetic tree of 705 odorant receptors (ORs) from *B. dioryctriae* and three other Hymenoptera species. The protein sequences used in this phylogenetic analysis are listed in the Supplementary Materials (File S4). The size and color of the dots reflect their bootstrap values. The color scale is shown on the left, with larger dots indicating larger bootstrap values.

A total of 155 putative *ORs* over 500 bp in length were identified in *B. dioryctriae*, encoding 166–475 amino acid residues. The putative *BdioORs* showed 24.88–94.95% similarity with other orthologous genes of Hymenoptera species. Seventy-nine BdioORs have full length and encode 5–7 TMDs (Table S6). The full length of *BdioOrco* was identified successfully, encoding 475 amino acid residues with 7 TMDs and displaying 94.95% similarity to CcunOrco of *C. cunea*. As expected, BdioOrco grouped with other Hymenopteran Orco sequences and formed a single clade in the ML tree of ORs (Figure 4). Other ORs were segregated into different clades, and some ORs from the one species clustered into the same clade. In *B. dioryctriae*, they were marked as species-expansion ORs in Figure 4, e.g., the eight BdioORs (OR19, OR100, OR148, OR83, OR22, OR3, OR17 and OR28) clustered into one clade. Additionally, most BdioORs showed a close relation to ORs of *N. vitripennis* (Figure 4).

**Figure 5:**
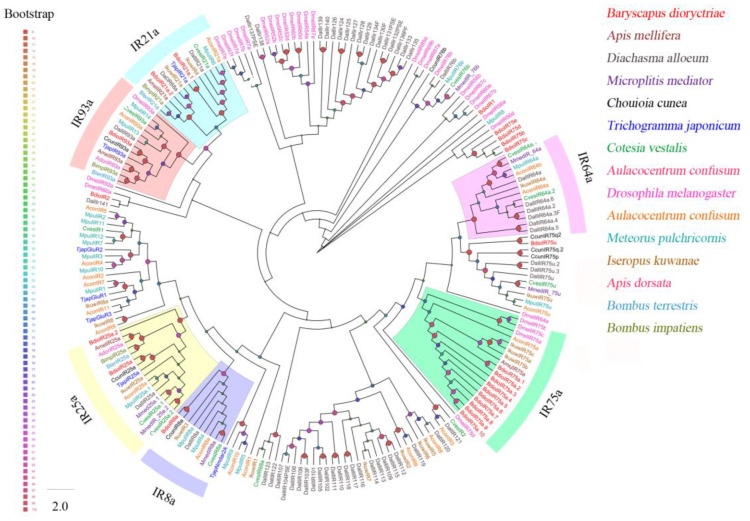
Phylogenetic tree of 211 ionotropic receptors (IRs) from *B. dioryctriae* and 13 other Hymenoptera species. The protein sequences used in this phylogenetic analysis are listed in the Supplementary Materials (File S5). The size and color of the dots reflect their bootstrap values. The color scale is shown on the left, with larger dots indicating larger bootstrap values.

Twenty-three putative *IRs* were identified in *B. dioryctriae*, encoding 106–937 amino acid residues with a similarity of 37.19–82.12% to the orthologous genes of known insect species. Five BdioIRs possessed full length and were predicted to have 3–4 TMDs (Table S7). Phylogenetic analysis showed that the BdioIRs were divided into several different subfamilies (Figure 5). *B. dioryctriae* has one homolog each in IR8a and IR93a; two homologs each in IR21a and IR25a; and 10 homologs in IR75a. The sequence alignment results showed that all of these IRs represent distinct IR genes (Figures S6–S8).

**Figure 6:**
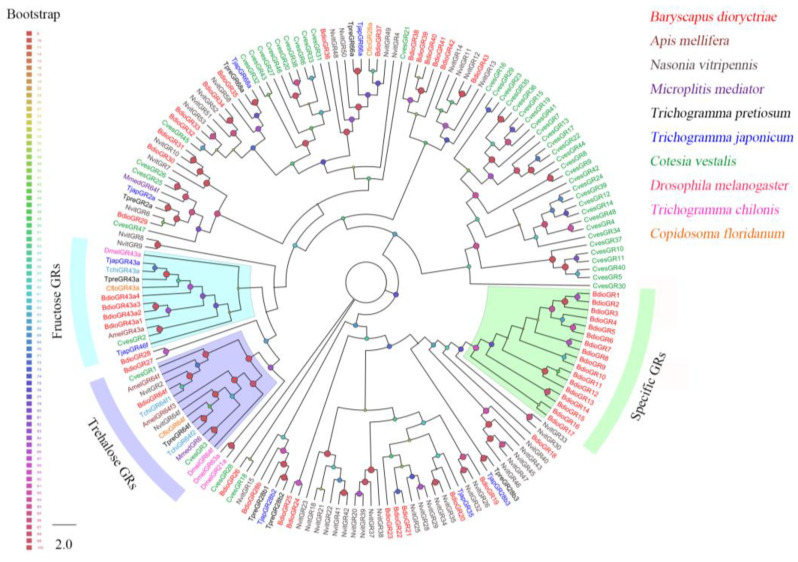
Phylogenetic tree of 120 gustatory receptors (GRs) from *B. dioryctriae* and nine other insect species. The protein sequences used in this phylogenetic analysis are listed in the Supplementary Materials (File S6). The size and color of the dots reflect their bootstrap values. The color scale is shown on the left, with larger dots indicating larger bootstrap values.

Forty-nine putative *GRs* were identified in *B. dioryctriae*, encoding 50–780 amino acid residues with 24.68–98.31% gene similarity with other known insect genes (Table S8). The phylogenetic analysis showed that BdioGR43a.1-4 and BdioGR64f were clustered into the fructose receptor subfamily and trehalose receptor subfamily, respectively; thus, these five genes were slightly predicted as sweet taste GRs (sugar receptors). In addition, there was a species-expansion clade consisting of 17 BdioGRs that were clustered together based on the sequences of the GR database used in this study (Figure 6).

**Figure 7:**
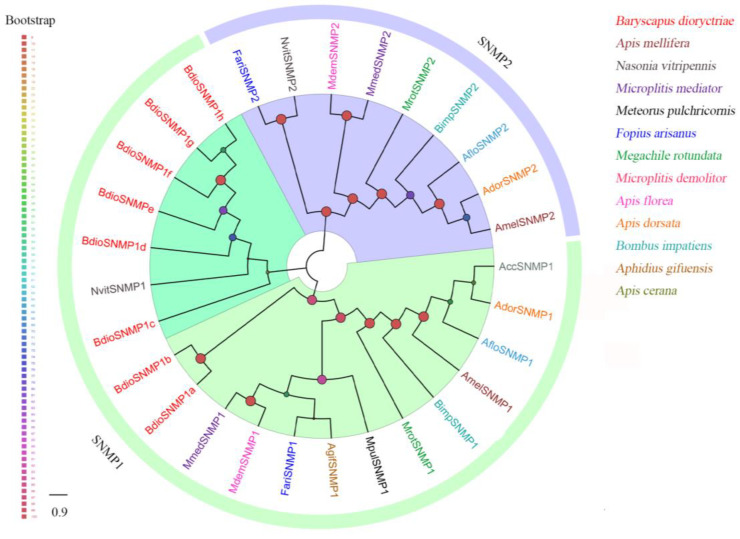
Phylogenetic tree of 29 sensory neuron membrane proteins (SNMPs) from *B. dioryctriae* and 12 other Hymenoptera species. The protein sequences used in this phylogenetic analysis are listed in the Supplementary Materials (File S7). The size and color of the dots reflect their bootstrap values. The color scale is shown on the left, with larger dots indicating larger bootstrap values.

Eight putative SNMPs were identified in *B. dioryctriae*, encoding 125–525 amino acids and showing 37.76–88.08% similarity with other orthologous genes of Hymenoptera. The phylogenetic tree showed that all eight BdioSNMPs belong to the SNMP1 family (Figure 7). Of these, five BdioSNMP1s (SNMP1a, SNMP1b, SNMP1e, SNMP1f, SNMP1h) were predicted to have two TMDs; both BdioSNMP1c and BdioSNMP1g have only one TMD, while BdioSNMP1d showed no TMDs (Table S9). Additionally, the SNMPs from *B. dioryctriae* and other 12 Hymenoptera species were combined with ten CD36 homologs (including epithelial membrane protein, croquemort, peste, santa maria and debris buster from *Drosophila melanogaster*, *D. busckii*, *Tribolium castaneum*, *Culex quinquefasciatus* and *Anopheles aquasalis*) to construct another phylogenetic tree (Figure S9). The result confirmed that all of these BdioSNMP1 homologs belong to the SNMP family, rather than being separate from CD36 proteins (Figure S9).

### 3.4. Differential Expression Gene Analysis

**Figure 8:**
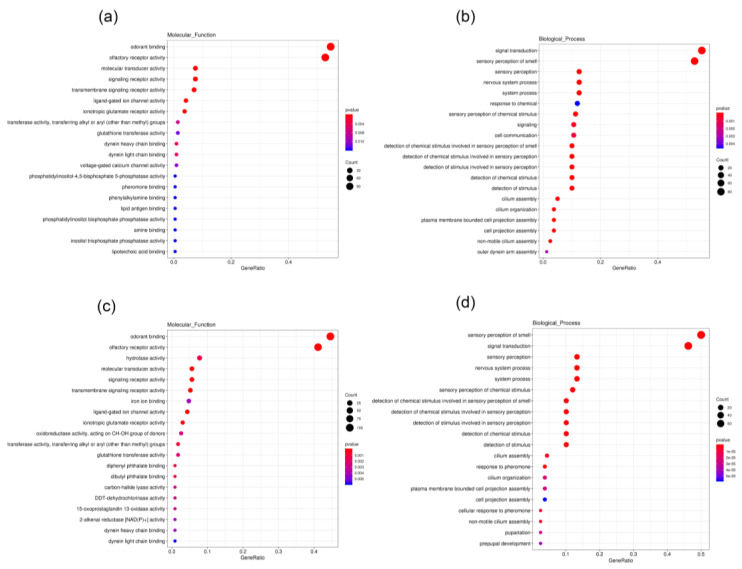
GO enrichment of 393 up-regulated unigenes in FA. (**a**) Unigenes enriched into the molecular function category. (**b**) Unigenes enriched into the biological process category; GO enrichment of 365 up-regulated unigenes in MA. (**c**) Unigenes enriched into the molecular function category. (**d**) Unigenes enriched into the biological process category. (The size and color of the red and blue dots represent the count and *p*-value of the category, respectively, and their rulers are shown on the right side of each subgraph).

Ten cDNA libraries from different tissues of *B. dioryctriae* were established in this study (Table 2). The expression levels of genes in different tissues were evaluated by their FPKM values (Table S10). According to the group settings in Table 1, the EBSeq program was used to conduct differential expression analysis based on the conditions “q value < 0.005 &|log2 (fold change) > 2”. Compared to other tissues, a total of 393 and 365 unigenes were found to be up-regulated in female antennae (FA) (Groups 1–5) and male antennae (MA) (Groups 6–10), respectively. Up-regulated DEGs in both female and male antennae were classified into three major categories—cellular components, molecular functions and biological processes—in the GO enrichment analysis. These up-regulated genes in FA and MA had the same GO enrichment feature. In the molecular function category, they were mainly involved in olfactory receptor activity (GO: 0004984, 112/95 unigenes) and odorant binding (GO: 0005549, 116/103 unigenes). In the biological process category, they were mainly annotated as sensory perception of smell (GO: 0007608, 84/79 unigenes) and signal transduction (GO: 0007165, 88/73 unigenes) (Figure 8). Additionally, a total of 377 unigenes were found to be significantly up-regulated in the ovipositor (Fov) compared to other tissues (Groups 10–15). The GO enrichment analysis showed that, in terms of molecular function, 65 unigenes participated in serine-type endo-peptidase activity (GO: 0004252), and 12 unigenes were involved in odorant binding (GO: 0005549). In the biological process category, they were mainly involved in proteolysis (GO: 0006508, 70 unigenes) (Figure S6).

### 3.5. Expression Profiling Analysis of Chemosensory Genes in B. dioryctriae

The FPKM values of seven chemosensory gene families (OBPs, CSPs, NPC2s, ORs, IRs, GRs and SNMPs) in ten different tissues of *B. dioryctriae* (with a standard of FPKM > 1) revealed that *BdioOBPs*, *BdioCSPs*, *BdioGRs* and *BdioSNPMs* were widely expressed in various tissues (Table 3); *BdioNPC2s* were mainly expressed in Fov, MH, Fab and L; and both *BdioORs* and *BdioIRs* were mostly expressed in male antennae (132 *BdioORs*, 19 *BdioIRs*) and female antennae (173 *BdioORs*, 20 *BdioIRs*), showing obvious antennae-biased expression (Table 3).

**Figure 9:**
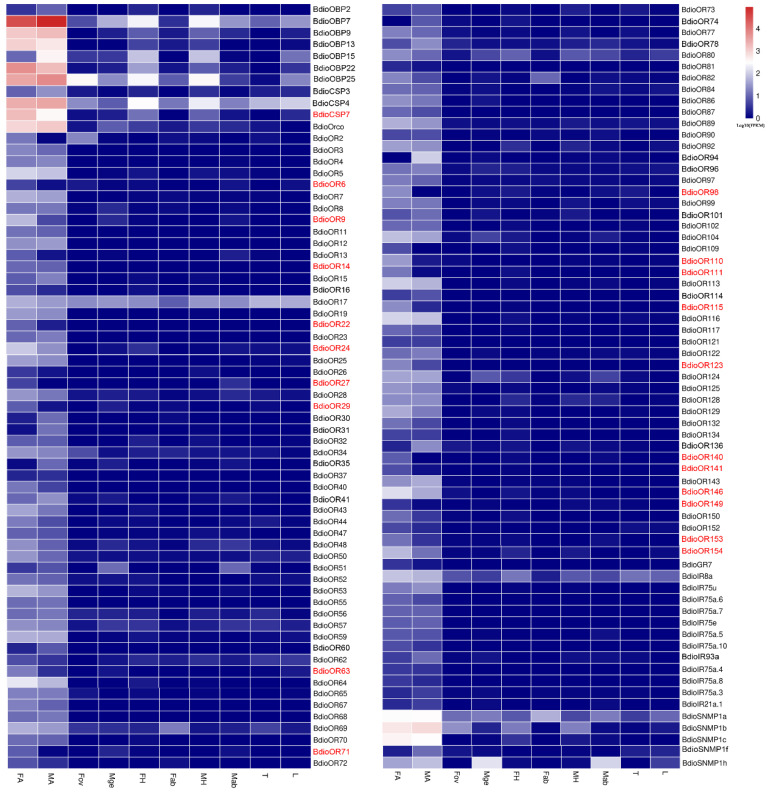
Expression profiles of antennae-biased chemosensory genes in *B. dioryctriae*. The chemosensory genes with female antennae-biased expression are marked in red, with a standard of q value < 0.005 &|log2 (fold change) > 2 when FA vs. MA. FA and MA: female and male antennae; FH and MH: female and male heads without antennae; Fab: female abdomens without ovipositors and digestive tracts; Fov: female ovipositors; Mge: male genitalia; Mab: male abdomens without genitalia and digestive tracts; T: male and female thoraxes; L: male and female legs.

**Figure 10:**
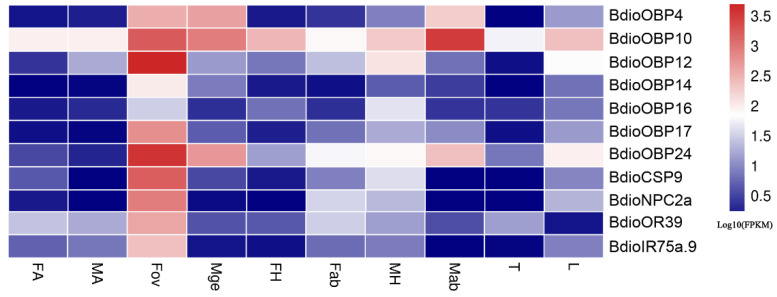
Expression profiles of ovipositor-biased chemosensory genes in *B. dioryctriae* with Scheme 0. &|log2 (fold change) > 2 when Fov vs. other nine tissues. FA and MA: female and male antennae; FH and MH: female and male heads without antennae; Fab: female abdomens without ovipositors and digestive tracts; Fov: female ovipositors; Mge: male genitalia; Mab: male abdomens without genitalia and digestive tracts; T: male and female thoraxes; L: male and female legs.

Furthermore, based on the differential expression analysis (q value < 0.005 &|log2 (fold change) > 2), a total of 132 chemosensory genes were found to be up-regulated in both FA (Groups 1–5) and MA (Groups 6–10) and showed antennae-biased expression, then the heatmaps of the FPKM values were drawn for these genes (Figure 9). Among these genes, 40 chemosensory genes exhibited sexually differentiated expression, and 21 genes showed higher expression in FA than in MA, including one *BdioCSP* (CSP7) and 20 *BdioORs* (OR6, OR9, OR14, OR22, OR24, OR27, OR29, OR63, OR71, OR98, OR110, OR111, OR115, OR140, OR141, OR146, OR149, OR153 and OR154). In addition, a total of 11 chemosensory genes were up-regulated in Fov and showed ovipositor-biased expression, including seven *BdioOBPs* (OBP4, 10, 12, 14, 16, 17 and 24), one *BdioCSP* (CSP9), one *BdioNPC2* (NPC2a), one *BdioOR* (OR39) and one *BdioIR* (IR75a), then the heatmaps of the FPKM values were drawn for these genes (Figure 10).

In total, there were 32 chemosensory genes showing female antennae-biased expression (21 genes, FA vs. MA) and ovipositor-biased expression (11 genes, Fov vs. other nine tissues) based on the DEG analysis (with q value < 0.005 &|log2 (fold change) > 2), and they were selected as candidate genes for a further RT-qPCR assay to validate their quantitative expression levels in ten different tissues.

### 3.6. Quantitative Expression Levels of the Candidates

**Figure 11:**
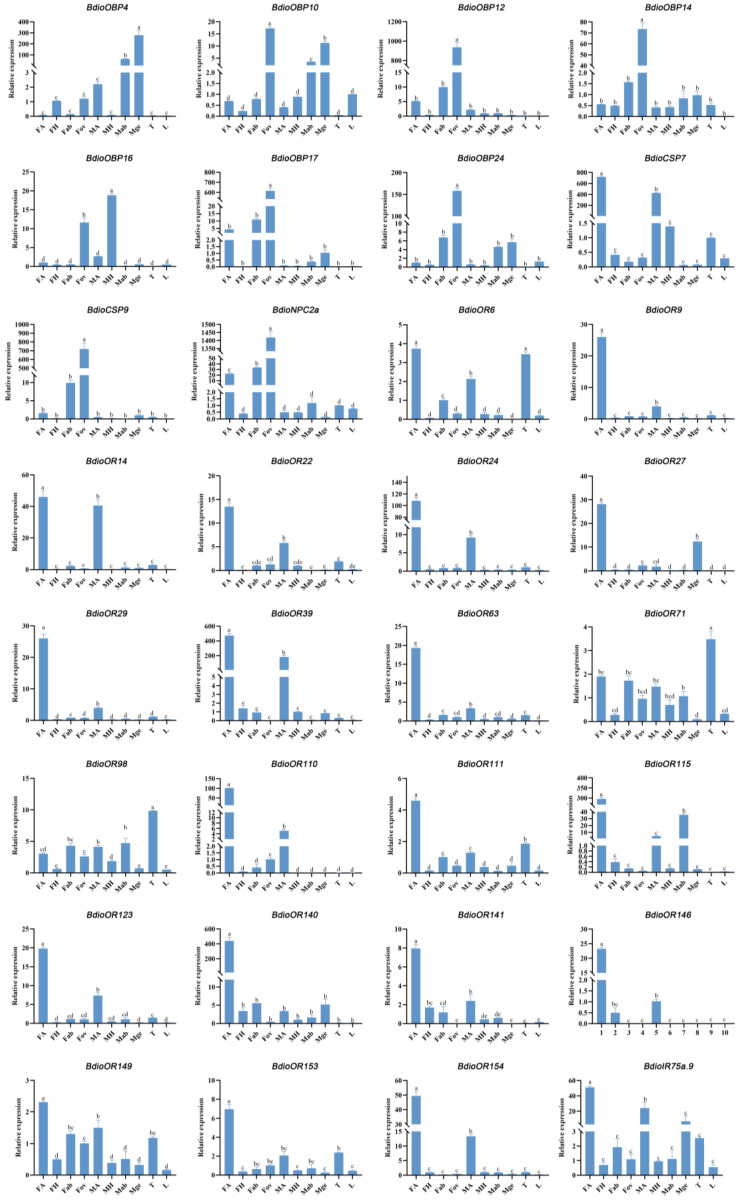
Expression levels of 32 candidate chemosensory genes in different tissues. (FA and MA: female and male antennae; FH and MH: female and male heads without antennae; Fab: female abdomens without ovipositors and digestive tracts, Fov: female ovipositors; Mge: male genitalia; Mab: male abdomens without genitalia and digestive tracts; T: male and female thoraxes; L: male and female legs). Columns labeled with different letters are significantly different (*p* < 0.05).

The RT-qPCR assay was conducted for analyzing and validating the expression patterns of the 32 candidate chemosensory genes with female antennae- and ovipositor biased expression that were calculated with top FPKM values (Figure 11). As expected, there were 26 chemosensory genes (26/32, more than 80%) showing the same expression pattern to the analysis results that were analyzed by the FPKM (Figure 9 and Figure 10). For the other six chemosensory genes, *BdioOR6* showed high expression in thoraxes as well as female antennae; *BdioOR71* and *BdioOR98* also showed higher expression in thoraxes than in other tissues; *BdioOBP4* showed much higher expression in male genitalia rather than in ovipositors; *BdioOR39* and *BdioIR75a.9* showed high expression in female antennae rather than in ovipositors; thus, these two (*BdioOR39* and *BdioIR75a.9*) were redistributed into the female antennae-biased expression group. Therefore, after the validations by RT-qPCR, a total of 28 chemosensory genes with female antennae- (20 genes) and ovipositor-biased expression (8 genes) were speculated as the targets that might be involved in detecting the key cues during host recognition, location, and oviposition in *B. dioryctriae* (Table 4).

## 4. Discussion

For parasitic wasps, it is very important to locate the host quickly and accurately to benefit their population development. *B. dioryctriae* is a gregarious pupal endoparasitoid wasp of many agricultural and forestry insect pests. However, the weak host location ability for nonprimary hosts has restricted their widespread application, although they can develop better in the substitute host (*G. mellonella*) than in their natural host (*D. pryeri*). The olfactory system plays an essential role in many life activities in insects, including foraging, mating, host locating, etc., and there have been increasing reports on investigating the molecular mechanism of chemical recognition during host location in various species [25,27,40]. To date, the olfactory mechanism underlying host recognition/location and oviposition site selection of *B. dioryctriae* remains unknown. Thus, we primarily identified and analyzed the chemosensory genes of *B. dioryctriae* based on the transcriptomes from ten different tissues. There were 274 putative chemosensory genes identified in *B. dioryctriae*, of which the two *BdioOBPs* (OBP1 and OBP5) were obtained by manual annotation, which could maximize the identification of the chemosensory genes of *B. dioryctriae*. Previous studies have shown that the sensilla distributed on antennae and ovipositors enable insects to evaluate host suitability by recognizing both the surface and internal contents of potential hosts [40,41]. In addition, although the chemosensory genes expressed in antennae are mostly involved in the olfaction-related behaviors of insects in general, the genes with ovipositor-biased expression are also worthy of investigation in parasitic insect taxa. For example, *HassOR31* expressed in the ovipositor of *Helicoverpa assulta* helps females determine precise egg-laying sites in host plants [42]; *BdorOBP56d* and *BdorOBP56d-2*, which are highly expressed in ovipositors of *Bactrocera dorsalis,* could bind 3-HA to influence oviposition preference [43]. Therefore, both chemosensory genes with female antennae- and ovipositor-biased expression were screened as target genes for further study to investigate the olfactory mechanism during host recognition/location and ovipositor site selection of *B. dioryctriae*.

OBPs play an important role in binding and transporting liposoluble odors during chemical signal detection in insects [44]. The number of OBPs in various parasitic wasps ranges from 2 (*Scleroderma guani*) [45] to 98 (*Nasonia vitripennis*) [46]. In our study, a total of 27 OBPs were identified in *B. dioryctriae*, which is an intermediate number. Numerous studies have shown that OBPs with specific and/or high expression in antennae/ovipositors play an important role in binding key volatile cues during foraging and host localization in parasitic wasps [47,48]. For example, *Cotesia vestalis* would take longer to seek hosts after knocking out *CvesOBP17/18/19*, which are highly expressed in the female antennae of this wasp [49]. *CcunOBP2* of *C. cunea*, which is specifically expressed in antennae, binds to the host plant volatile 3-carene [48]. The role of *BdorOBP56d* and *BdorOBP56d.2*, which are highly expressed in the ovipositors of *B. dorsalis*, have been mentioned above [43]. Here, five *BdioOBPs* showed higher and more specific expression in antennae compared to other tissues (Figure 9) and six *BdioOBPs* showed ovipositor-biased expression (Figure 11). These eleven genes are worthy of being further examined for their roles in the reproductive behaviors of *B. dioryctriae*.

CSPs are soluble carrier proteins that function in a manner similar to OBPs in chemical signal reception in insects [50]. A total of nine CSPs were identified in *B. dioryctriae*, which is close to the number of CSPs in *C. cunea* (11) [51], *A. nilaparvatae* (11) [20] and *Aphidius gifuensis* (12) [52], but less than that in *D. longicaudata* (19) [23]. Previous studies have shown that some insect CSPs are expressed exclusively in non-sensory organs (legs, heads, thoraxes, midguts, ovaries, fat body and even the female pheromone glands, etc.) and are involved in diverse functions, such as development, reproduction, flight and drug and stress resistance [53,54]. For example, in Hymenoptera, the larvae of *Solenopsis invicta* would fail to undergo ecdysis after knocking down the *Si-CSP9* gene [55]. In our present study, we mainly focus on the genes with potential olfactory function; thus, the *BdioCSPs* that are expressed in nonolfactory functional tissues were ignored. On the contrary, based on the RT-qPCR results, we found *BdioCSP7* showed female antennae-biased expression, and *BdioCSP9* was obviously specific and more highly expressed in the ovipositor (Fov) than in any other tissue. The detailed functions of these two *BdioCSPs* in *B. dioryctriae* could be investigated in the future.

NPC2s are considered small soluble proteins and are critical for cholesterol transport in humans [56], while in Hymenoptera insects, they are odorant carrier proteins, similar to OBPs and CSPs in the olfactory system [57]. A total of three *NPC2* genes were identified in *B. dioryctriae*, which was fewer than the total of ten members of the NPC2 family known in most Hymenopteran species [20], such as *A. nilaparvatae* (4), *Microplitis demolitor* (8) and *Fopius arisanus* (10) [20]. Previous related studies on Hymenoptera insects revealed that *NPC2s* are generally specifically expressed in antennae, including *MmedNPC2* of *Microplitis mediator* [57] and *McinNPC2* of *Macrocentrus cingulum* 57. Among the three *BdioNPC2s* identified in this study, *BdioNPC2a* was highly expressed both in female antennae and ovipositors, especially in the latter. This may be caused by the functional differentiation of arthropod NPC2 proteins. The *BdioNPC2a* with this special expression pattern has been considered a target gene used for our ongoing study to explore its physiological function in *B. dioryctriae*.

ORs are important chemoreceptors involved in recognizing chemical volatiles and pheromones [7]. A total of 155 ORs were identified in *B. dioryctriae*, which is close to the number of ORs *in M. mediator* (169) [58] and *A. mellifera* (170) [59] but less than that in *N. vitripennis* (301) [60]. The different numbers of *OR* genes in wasps may be affected by species specificity and sequencing technology and depth [20,27]. In Hymenopteran species, a large expansion event of the OR gene family has been found, and gene gain and loss events are common in bees, wasps, and ants [61]. This is also the case for BdioORs, which form many species expansions, and the specific expansion of these genes in *B. dioryctriae* may contribute to sensing some specific external information substances for this parasitic wasp. The up-regulated expression of these genes in the antennae also supports this inference. There were 104 *BdioORs* highly expressed in the antennae, of which 18 *BdioORs* were expressed at significantly higher levels in the female antennae (FA) than in other tissues. Compared with Lepidoptera, Diptera and Coleoptera, although many ORs have been identified in many Hymenoptera taxa in recent years, functional analyses of ORs are still lacking in parasitic wasps due to their large number of OR genes. Screening ORs that are highly and/or specially expressed in tissues with olfactory function (antennae and ovipositors) in parasitic wasps might be an effective way to narrow the range of target genes critical for host location. Therefore, the 18 *BdioORs* with female antennae-biased expression were purposefully screened for further study.

IRs are sensory proteins that evolved from ionotropic glutamate receptors (iGluRs) [62], and are usually expressed in combinations of sensory neurons that respond to various odors [44]. A total of 23 *IRs* were identified in *B. dioryctriae*, which is similar to the numbers in *A. nilaparvatae* (22) [20] and *A. gifuensis* (25) [63]. Additionally, members of the IR8a, IR25a, IR93a, IR21a and IR75a families were identified in *B. dioryctriae*. The co-receptor families of IR8a and IR25a have distribution patterns and functions similar to those of the Orco family but are expressed in different sensilla [7]. There were two IR25a homologs identified in *B. dioryctriae* (Figure S7), similar to the parasitoid wasp *M. mediator* and *Cotesia vestalis* [64,65]. In *Drosophila melanogaster*, IR21a/IR25a are involved in cold–warm sensation [66], and IR25a/IR93a are essential for heat and moisture sensing [67,68]. For *B. dioryctriae*, which could survive the long, cold winter in northeast China, the BidoIR25a might be also involved in temperature sensing. IR75a is a receptor that probably originated in the neodipteran ancestor (>200 million years ago), and previous studies found that IR75a of *D. melanogaster* and *D. simulans* is tuned predominantly to acetic acid [69]. In this study, we found a large expansion event of the IR75a subfamily in *B. dioryctriae*, and through sequence alignment, proved that all these IRs represent distinct IR genes (Figures S6–S8). Moreover, pines are the main hosts of *Dioryctria* pests, and they release acetic acid during storage [70]. These acetic acid odors might be detected by IR75a in *B. dioryctriae,* similar to how the genes in this family work in *D. melanogaster* and *D. simulans*; thus, we hypothesized that BdioIR75a might be involved in the habitat location of *B. dioryctriae*, but further experimental verifications are needed.

The GR family consists of several major subfamilies, mediating the perception of carbon dioxide, fructose, various sugars, bitter compounds, etc. [8]. In parasitic wasps, the number of GRs identified via RNA-seq ranged from 2 (*M. mediator*) [64] to 41 (*Aenasius bambawalei*) [71]. In *B. dioryctriae*, forty-nine GRs were totally identified based on ten transcriptomes, of which five *BidoGRs* were predicted as sweet taste GRs, including four BidoGRs (GR43a.1-4) and one BidoGR (GR64f) that were clustered into the fructose receptor and trehalose receptor subfamilies, respectively (Figure 6). *DmelGR43a*, known as the fructose and sucrose receptor in *D. melanogaster*, was believed to play a key role in sensing the internal fructose levels in the brain of *D. melanogaster* [72]. *BdioGR43a.1* showed highly expressed in heads (Table S10), suggesting that it may perform the same role in *B. dioryctriae*; the potential trehalose receptor *BdioGR64f* was highly expressed in antennae compared to other tissues (Table S10), which might take a part in the ingestion of *B. dioryctriae*. Additionally, GR43a and GR64f were clustered together into the sugar receptor subfamily (Figure 6), which was consistent with previous studies [73]. However, there were no candidate CO_2_-subfamily GRs identified in this study, which might be caused by the lack of a transcriptome dataset for labial palps of *B. dioryctriae*, considering that CO_2_ detection often occurrs in specialized sensilla distributed on the labial palps [74,75]. Although the functions of GRs have been well-researched in *Drosophila* [76,77], there are still many unknowns in other non-model insects as well as wasps.

SNMPs are transmembrane structural proteins that play key roles in the peripheral olfactory system [9]. The number of SNMPs in various species ranges from two (*Drosophila melanogaster*) [78] to sixteen (*Onthophagus taurus*) [79]. A total of eight SNMPs were identified in *B. dioryctriae*, which is much more than the number of SNMPs found in *Iseropus kuwanae* (2) [16] and *M. mediator* (2) [80], suggesting that *BdioSNMPs* have large species expansions among Hymenoptera [81]. In general, numerous studies have found that most insects have two types of SNMPs, namely SNMP1 and SNMP2 [50], and all identified *BdioSNMPs* belong to the SNMP1 subfamily (Figure 7 and Figure S9). This paralogue of the same *SNMP* type is similar to that in the hessian fly *Mayetiola destructor,* which encodes six *SNMP1* paralogues [39]. SNMP1s are believed to contribute to pheromone detection in moths, and our previous study has proved this essential role for SNMP1 in the detection of sex pheromones in *Helicoverpa armigera* [82]. In the parasitic wasp *M. mediator*, *MmedSNMP1* is significantly expressed in the sensilla placodea (an olfactory sensilla) of antennae, and possibly involved in perceiving plant volatiles and sex pheromones [80]. In *B. dioryctriae*, there were five BidoSNMP1s more highly expressed in antennae than in other tissues (Figure 9), of which, *BdioSNMP1f* showed male antennae-biased expression. We slightly speculated that *BdioSNMP1f* might participate in detecting the sex pheromones emitted from female *B. dioryctriae*, but this claim needs further functional validations.

## 5. Conclusions

In this study, a total of 274 putative chemosensory genes were identified based on transcriptomes from ten different tissues of *B. dioryctriae*, including 39 water-soluble proteins (27 OBPs, 9 CSPs and 3 NPC2s) and 235 transmembrane proteins (155 BdioORs, 49 BdioGRs, 23 BdioIRs and 8 BdioSNMPs). There were 28 chemosensory genes with female antennae- and ovipositor-biased expression. Our study preliminarily narrowed the range of candidate genes involved in recognizing key cues during host recognition/location and oviposition and provided a molecular basis for exploring the olfactory mechanism underlying these behaviors in *B. dioryctriae.*

## Figures and Tables

**Table 1:** The comparison groups of differential expression gene analysis.

Group	Control	Treatment
Group 1	FH	FA
Group 2	Fab	FA
Group 3	Fov	FA
Group 4	T	FA
Group 5	L	FA
Group 6	MH	MA
Group 7	Mab	MA
Group 8	Mge	MA
Group 9	T	MA
Group 10	L	MA
Group 11	FH	Fov
Group 12	Fab	Fov
Group 13	FA	Fov
Group 14	T	Fov
Group 15	L	Fov

**Table 2:** Transcriptome assembly summary of *B. dioryctriae*.

Sample Name	Read Number (bp)	Base Number (bp)	GC (%)	% ≥ Q30
FA	21,365,459	6,389,699,322	40.85	92.34
MA	20,419,896	6,086,831,598	42.82	93.90
Fov	21,149,237	6,330,810,618	41.04	92.77
Mge	21,426,811	6,405,805,010	41.15	92.36
FH	20,186,680	6,046,853,996	41.36	91.85
MH	20,573,530	6,160,481,816	40.61	91.42
Fab	22,013,855	6,595,099,202	41.80	91.94
Mab	20,787,145	6,215,089,286	42.05	92.82
T	21,018,731	6,293,838,018	39.09	91.93
L	21,630,718	6,466,720,454	39.39	92.03

**Table 3:** Summary of chemosensory genes expressed in various tissues of *B. dioryctriae* (FPKM > 1).

Tissues	Gene Number Expressed in Different Tissues						
	OBPs	CSPs	IRs	GRs	NPC2s	SNMPs	ORs
FA	21	7	20	16	0	7	173
MA	20	6	19	11	0	7	132
Fov	21	5	2	10	1	4	23
Mge	23	8	1	14	0	6	34
FH	23	6	1	12	0	5	31
MH	26	7	3	13	2	4	28
Fab	17	6	2	7	1	3	17
Mab	19	5	2	12	0	5	31
T	13	5	2	12	0	4	23
L	23	8	2	14	1	5	18

**Table 4:** Twenty-eight chemosensory genes with female antennae- and ovipositor-biased expression.

Gene Family	Chemosensory Genes with Female Antennae-Biased Expression		Chemosensory Genes with Ovipositor-Biased Expression	
	Gene Number	Gene Name	Gene Number	Gene Name
*BdioOBPs*	0	-	6	OBP10/12/14/16/17/24
*BdioCSPs*	1	CSP8	1	CSP10
*BdioNPC2s*	0	-	1	NPC2a
*BdioORs*	18	OR9/14/22/24/27/29/39/63/110/111/115/123/140/141/146/149/153/154	0	-
*BdioIRs*	1	IR75a.9	0	-
*BdioGRs*	0	-	0	-
*BdioSNMPs*	0	-	0	-

## Data Availability

The datasets analyzed in the current study are available from the corresponding author on reasonable request.

## Supplementary Materials

The following supporting information can be downloaded at: https://www.mdpi.com/article/10.3390/insects13121098/s1, Figure S1. Distribution of unigene size in the *B. dioryctriae* transcriptome assembly; Figure S2. Multiple amino acid sequence alignment of BdioOBPs; Figure S3. Multiple amino acid sequence alignment of BdioCSPs; Figure S4. Multiple amino acid sequence alignment of NPC2s in *B. dioryctriae* and six other Hymenoptera insects (*Apis mellifera, Nasonia vitripennis, Microplitis mediator, Diachasma alloeum, Trichogramma pretiosum* and *Microplitis demolitor);* Figure S5. Multiple amino acid sequence alignment of BdioSNMPs; Figure S6. Multiple amino acid sequence alignment of BdioIRs; Figure S7. Multiple amino acid sequence alignment of BdioIR25a and BdioIR25a.2; Figure S8. Multiple amino acid sequence alignment of BdioIR75as; Figure S9. Phylogenetic tree of 29 SNMPs from 13 Hymenoptera species and 10 CD36 homologs (including epithelial membrane protein, croquemort, peste, santa maria and debris buster) from *Drosophila melanogaster*, *D. busckii*, *Tribolium castaneum*, *Culex quinquefasciatus* and *Anopheles aquasalis*. The protein sequences used in this phylogenetic analysis are listed in File S7; Figure S10. GO enrichment of 377 unigenes upregulated in Fov; Table S1. Assembly of unigenes and transcripts of *B. dioryctriae*; Table S2. Annotation of *B. dioryctriae* with BLAST; Table S3. Sequence information of identified OBPs in *B. dioryctriae;* Table S4. Sequence information of identified CSPs in *B. dioryctriae*; Table S5. Sequence information of identified NPC2s in *B. dioryctriae*. Table S6. Sequence information of identified ORs in *B. dioryctriae*; Table S7. Sequence information of identified IRs in *B. dioryctriae*; Table S8. Sequence information of identified GRs in *B. dioryctriae*; Table S9. Sequence information of identified SNMPs in *B. dioryctriae*; Table S10. The FPKM values of chemosensory genes in *B. dioryctriae;* Table S11. Primers of candidate chemosensory genes and GAPDH used for RT-qPCR. Supplementary Data S1. The amino acid sequences of 233 odorant-binding proteins (OBPs) from eight Hymenoptera species; Supplementary Data S2. The amino acid sequences of 91 chemosensory proteins (CSPs) from 12 Hymenoptera species; Supplementary Data S3. The amino acid sequences of 29 Niemann–Pick type C2 proteins (NPC2s) from 11 Hymenoptera species; Supplementary Data S4. The amino acid sequences of 705 odorant receptors (ORs) from four Hymenoptera species; Supplementary Data S5. The amino acid sequences of 211 ionotropic receptors (IRs) from 15 Hymenoptera species; Supplementary Data S6. The amino acid sequences of 120 gustatory receptors (GRs) from ten insect species; Supplementary Data S7. The amino acid sequences of 29 SNMPs from 13 Hymenoptera species and ten CD36 proteins from five insect species.
