# Supplementary material for: Transcriptome Analysis and Identification of Chemosensory Genes in Baryscapus dioryctriae (Hymenoptera: Eulophidae)
Source: Insects. 2022 Nov 29;13(12):1098. doi: 10.3390/insects13121098 (PMC9780838; doi:10.3390/insects13121098)
Supplement: Supplementary file 1 [file insects-13-01098-s001.zip › insects-2000003-supplementary.pdf]

Supplementary Materials

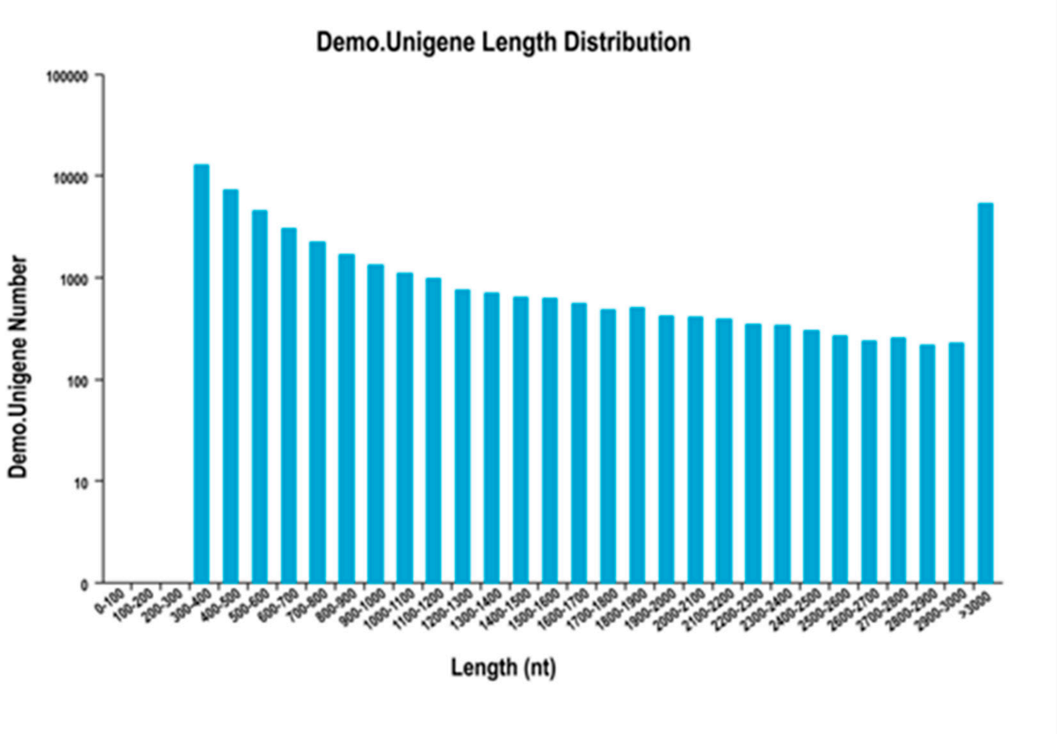

Figure S1. Distribution of unigene size in the *B. dioryctriae* antennal transcriptome assembly.

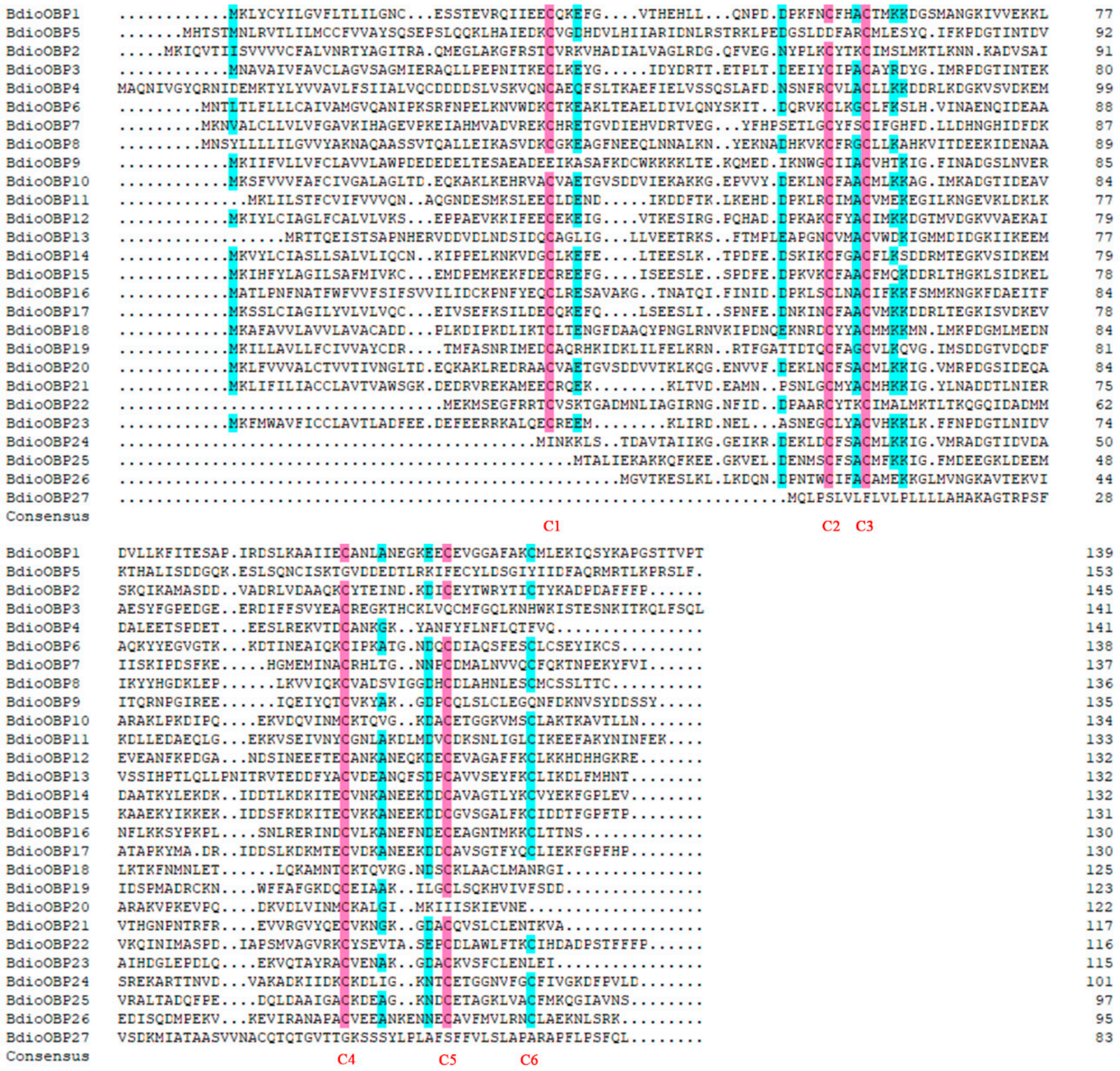

Figure S2. Multiple amino acid sequence alignment of BdioOBPs.

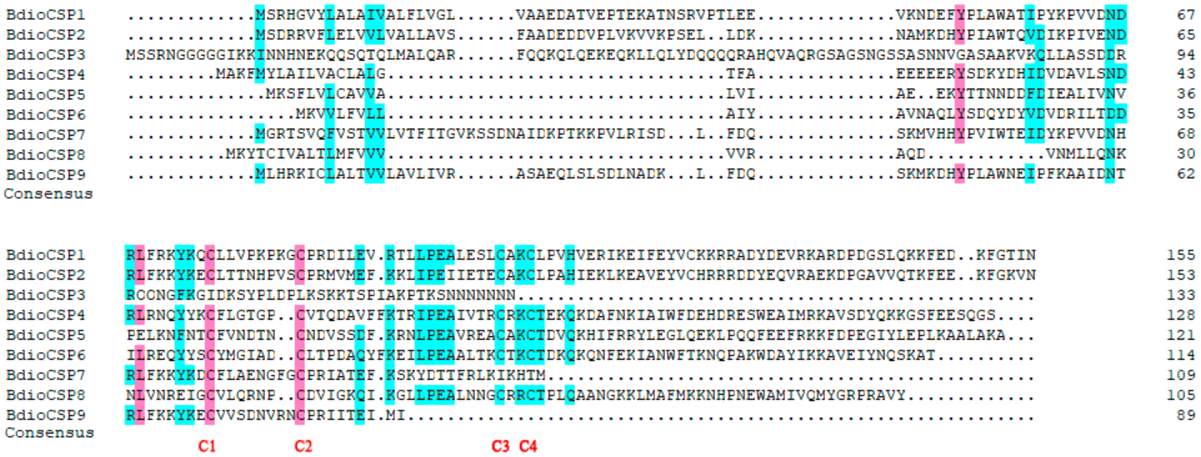

Figure S3. Multiple amino acid sequence alignment of BdioCSPs.

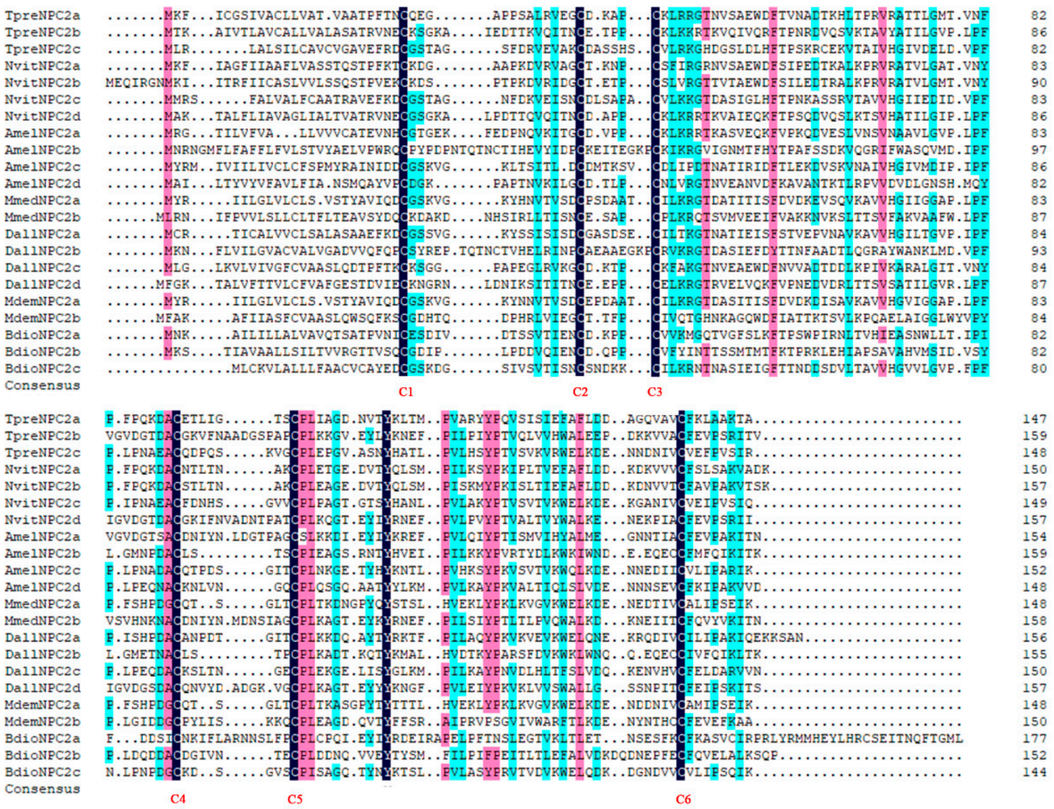

Figure S4. Multiple amino acid sequence alignment of NPC2s in *B. dioryctriae* and 6 other Hymenoptera insects (*Apis mellifera, Nasonia vitripennis, Microplitis mediator, Diachasma alloeum, Trichogramma pretiosum, Microplitis demolitor*).

```
BdioSNMP1a   .....MAMNRMMKFAIAGPCLMMFGILFGFVAFPKLLKGGIHKMVNLKPGTDVRALWSKIPFVI..DFKIFLFNVTNPDEIKNGAKPIVREVGPYYFEEWHEKNNLVDNE   103
BdioSNMP1b   .....MGKMSLMKKLGIGGGIMFVLSILMWFGFPKLLVSMIKSQINLKPGTDVRGVWSKIPFPI..DFRVYLYNVTNPDEIKAGAKPIVRQVGPYYFEEWHEKDNLIDRD   103
BdioSNMP1c   ..............................................................................................................     0
BdioSNMP1d   ...................................MMITPLWFTHLKEVALTRGTLKRQTWTKF..PFACELRVYAFNVTNPDEILKGEKPILKEIGPFVYDVWHEKINLVDDE    77
BdioSNMP1e   MGKIDLFSTRIRKIGAAGLCLFIFGIILRTAILHSMLESQIKKRIVLKKNSAMREIWSRF..PY..EMHVYLFNVTNPDEIAKGQKPIVKEVGPFVYDVWQEKINQIDYE   106
BdioSNMP1f   ....................................................MRKIWSKWSSPH..EYHVYFFDIINPDEIVKGEKLIVKETGPYIYDVWLERIDMVDHE    56
BdioSNMP1g   ..............................................................................................................     0
BdioSNMP1h   .MMENPKLLRYRVCAAIGLCVSILGVISLEYVIPSMEKRRILKAIALKDGWPARKTWNELSFSN..EFRAYLFNITNPDEISNGGKPIVREIGPYVYDVQLEKVEVADHE   107
Consensus

BdioSNMP1a   EEDSVTYSPKNTFIFRPELSN.GLTGEEELMLPHIFILAMVFATMREKPSAAPLINKAINSIFKNPENVFVKAKAMDLMFRGLPIDCSVTDTAGA.AVCSLL..KANADD   209
BdioSNMP1b   EDDSVEYAIKNTFYFNAEKSGEGLTGEEELMLPHVFILAMVMTVVRDKPTAVPVINKAVNSIFKSPDNVFMKMKAMDLMFRGLMVDCTVSDFAGG.AVCGML..KENPDG   210
BdioSNMP1c   ........MKNTYYFNAEKSN.GLTGDEEIVIANLFALGLVNTLLREKPSAIPIFGKAIDSIFKKPDNLFIKTTPKKILFDGIPIDCTAKDFAGA.AICGEVREKYEDFG   100
BdioSNMP1d   ETDTVTYSVDNTYLFNTEKSK.NLSDTMELTVASYLAFAGVNSAMKDYAALISVASKAIDAVGRNPPSIFLKEPLRTFLFGDEYYDCRGLTDFAPKVMCNSARDHYTEYR   186
BdioSNMP1e   DNDSLSYLSKDTYFFNTEKSN.GLSDDTELTLIHLVCIATPNKLLKINPTVLSYGVKGIYNIMNNPESIFFKFKVRTFLFDGIRFSCEGKDDFFATAVCNDIKQFWFSYR   215
BdioSNMP1f   ENDTLGYKLKKTFIFNATKSH.PLTEEDEVTLVHLVILGTANSLLRSRPSLLPLASKSIDSLFNKPKSLFTKVSVKTYLFDGFQIDCVGVEEFFAKAVCNDAKEFWFNYR   165
BdioSNMP1g   ..............................................................................................................     0
BdioSNMP1h   ENDTMSFKIKRTFYFNATKSY.PLSDEDELILTHYLILGTATGVLKTRPTVLPLASKSIDRLFNNPKSMFFNTKVKTYMFDGYELNCVGVEDFFAKAMCSDIEEFWYDYL   216
Consensus

BdioSNMP1a   LIVDD.PDHFRFALLGAKNGTTSKNRIKVLRGVKAQNDVGVVIELNNKTKMTTWNDTKCDAYQGTDGYVFHPYLYADEDIVS..FAPDLCRSIAAYTESTFKKQGLMVNR   316
BdioSNMP1b   LIVYD.EDHFGFALLGAKNGTAAKQRLRVLRGRKNLMDIGKVIEYDGKKNMSKWDDEKCDAFNGTDSYVFHPFLYEDEDVVS..FAPDLCRSLGAYFQKKTKLAGLNTNR   317
BdioSNMP1c   MIKTA.EDEYLFSLWATRNATESKKPTRVLRGLKNIMDVGRVVEYNYKNNLSIWDDEYCDRLNGSDGTIFHPNFDKHGKDPMIAFNDGLCRAITLTYEKPSKFKGLKTLQ   209
BdioSNMP1d   MRKRAEDLYSYSVFSGVSATTAYSIKHYL.................................................................................   215
BdioSNMP1e   MEKGD.DYFALSIFGTMNGTDFYNGKTRIQRGTKNMMEVGDIVEYNDMNNMSIWKDDICDALLGTDGTMFHPFLDANKNNVLHVTRPFYCRSFTYQYDSDVEFEGLKLVR   324
BdioSNMP1f   FVQVREDYYTSSFLDTFNNTVAWVGESRINRGIKNITKIGEFVKYNNTNNFAKWDDEKCDAFVGTDGTMFRPFLSRTDD..LHVVNEYLGRSFTYEYDSDVTNSGIHCFR   273
BdioSNMP1g   ..........................MRINRGIKNIEEIGKVVRYNNTNNFGKWGNEKCDAFVGTDGTMFPPFLSRSDD..LHVVHTLTSRSLTYKYESDISYGGFDCYR    82
BdioSNMP1h   MVKVRESVYTSSTMGIWNNTLAWVGETRINRGIRNITKIGEFVEYNHTKNFGKWGDEKCDKFVGTDGTMFPPFLSRKDD..LHMVFTTLKRSLTFHYDSDVVHDGIKCYR   324
Consensus

BdioSNMP1a   YTAWLGDPVK.HPEQKCYCPTSG.CLKAGMMDLHKCVGVPLVASHPHFFRADEEYLTTVDGLSPSAEKHMIFIDFEPFSGTPLEAQKRLQFNIQIHKVEKVKIMKNFPSA   424
BdioSNMP1b   YTAYLGDPST.DENLKCNCEAPDKCLKAGMMDLHKCLGVPLVASHPHFYMADESYLEMVDGLSPKQEDHEIFLDFEPFTGTPMYAKKRLQFNIPITKVDKFKIMKTFPTA   426
BdioSNMP1c   YTTDLGTDPD.NPLHKCYCQAPDNCLKKGVYDAYKCVGAPIVVSNPHFYLADKEYLEQVEGLKPDKALHAVAIDLDAFSGTPVQAHIRAQFNFFLFKVEKYKIMKNFPSA   318
BdioSNMP1d   ..............................................................................................................   215
BdioSNMP1e   YTASLGSDLGTNPKDKCFCISPDRCLKDGVYELLKCFNLARIGSNPHFYLADPYYGTTVEGLKPMKEKHMSIIDIDPLTGLAVRSHFRIQFNVELTAIKKYKLMQNFPDA   434
BdioSNMP1f   YTASLGTNPETNTNDKCYCLTPDDCLKNGVFETWKATNWPCIISNPHFYLADPFYNGRVTGLNPNREKHISTIDVHPLTGTVVSAKFRAQWNGPLTKQPKYRLLKNVYET   383
BdioSNMP1g   YVATLGADLETNPDDKCYCLSPDQCLKNGVFEGWNAVHWPVVGSNPHFCLADPYYIERVSGLNPDPKKHLSTIDVHPLTGIVVGAKFRLQWSAPLTKQPKYRLFENVSEA   192
BdioSNMP1h   YVATMGIDLETNPDDKCYCVEPDRCLKNDLFDTWKAVRFAAVVSSPHFYLTDPYYDDIIVGFHPDKEKHISYIDVHPLTGSVVNSKIRTQWCVPVSKQPKYRVMKNMADT   434
Consensus

BdioSNMP1a   MLPLFWVEEGLVIPDSFVSQVKMLHMVVTVMKCLTWIKIVAGLGMTGYAGFLYYKSTQSSQKLEITKPPKQENGVKPP...ISTLDANFLRAQVPPAI   519
BdioSNMP1b   MLPLFWVEEGVLLPDELIAQVKMIHTLLTVVAVMKWLMLVGGLGMGGGAGFLFYKATQNGNKLEITKVPKQNGKPSSTEKKISPLNVNTLQAQVPPVL   524
BdioSNMP1c   LLPIFWFDEHTVLPKFLIKEVKMGHRLLMMGNVFKYLVLLGGLGALGYGSFMQFKEMQGNEVKIKKTVKAKNGNETNGNDKKMPMNISSTISVPPIVE   416
BdioSNMP1d   ...................................................................................................   215
BdioSNMP1e   MLPMTWIDEVTIMPDYFISKIKNIYKMADVVKTLSYLMMFSGLTMCGYSIFREYKLKKEKRAVVKTVALEMRNNETNGD....................   513
BdioSNMP1f   LLPMIWYEEKFTLPARDIRSLKKLSSKIAFEKLFTIMIIVSGLMLFVVGVAYEISL.KYKSLKSKT.................................   448
BdioSNMP1g   LLPIGWTEERYYLPPPEMENLKKLNFKIVVEKFLAYHIIAIGFALLFMGILFEV.L.KRKKPK....................................   253
BdioSNMP1h   LLPLGWLEDSFTMPESNIKRLKKLSSMIAMEKICVYVVTAIGLAFFSVGVTCEVLN.RRKSKQPN..................................   498
Consensus
```

Figure S5. Multiple amino acid sequence alignment of BdioSNMPs

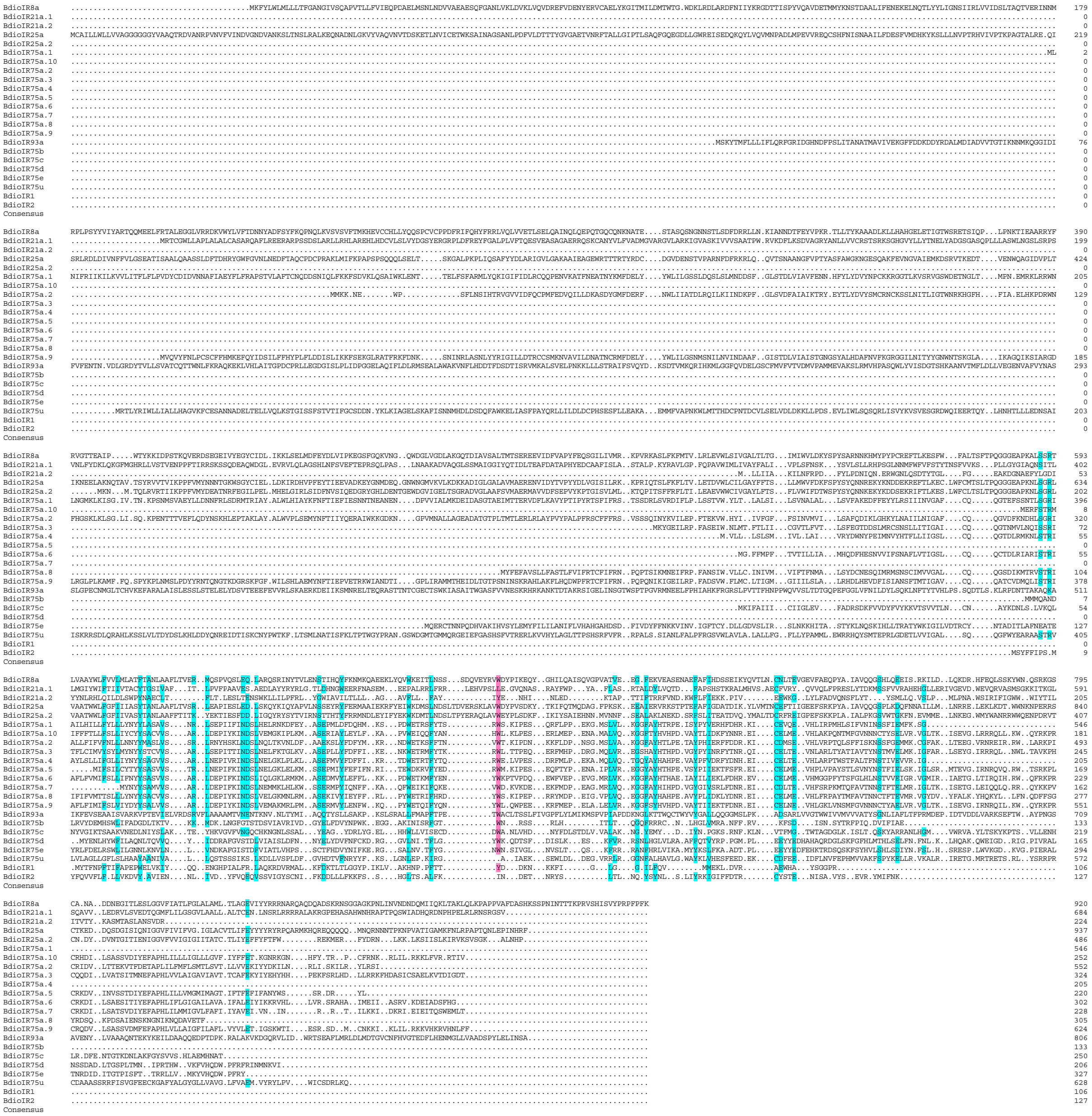

Figure S6. Multiple amino acid sequence alignment of BdioIRs.

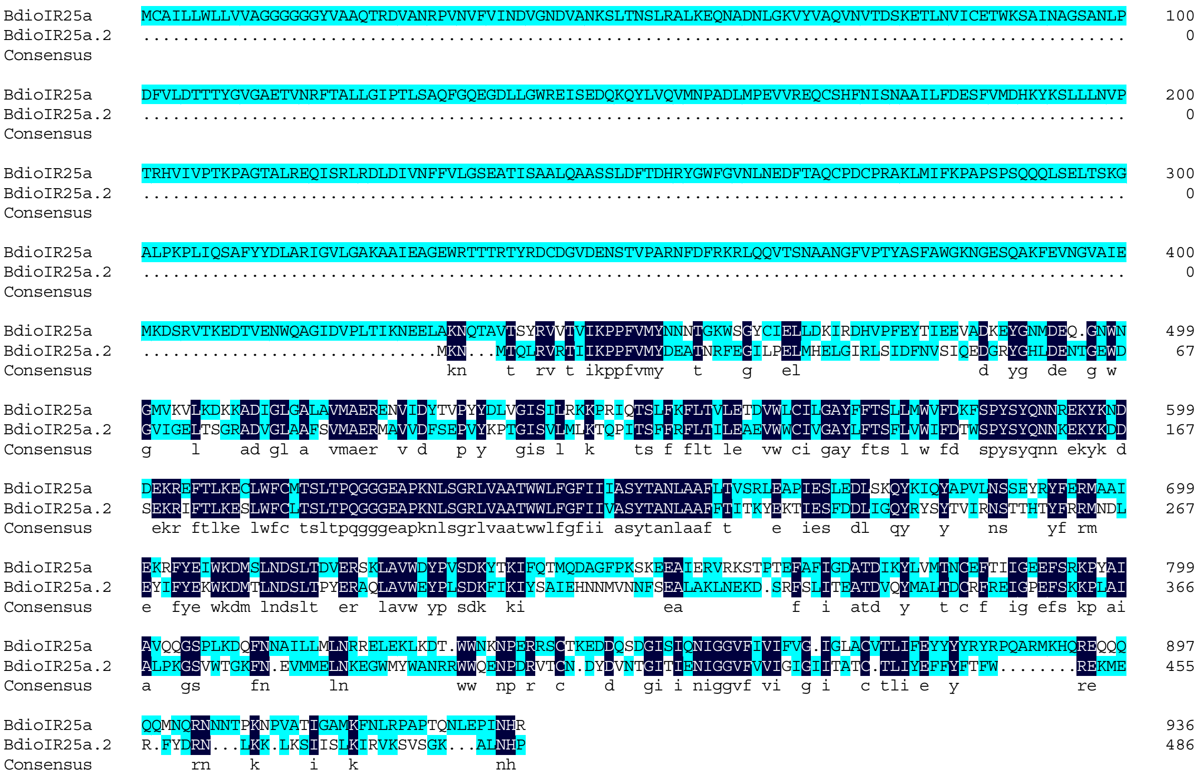

Figure S7. Multiple amino acid sequence alignment of BdioIR25a and BdioIR25a.2.

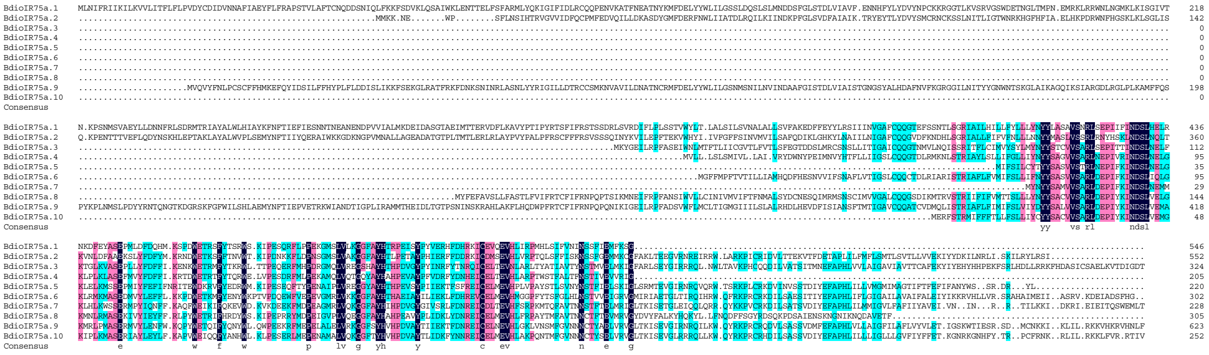

Figure S8. Multiple amino acid sequence alignment of BdioIR75as.

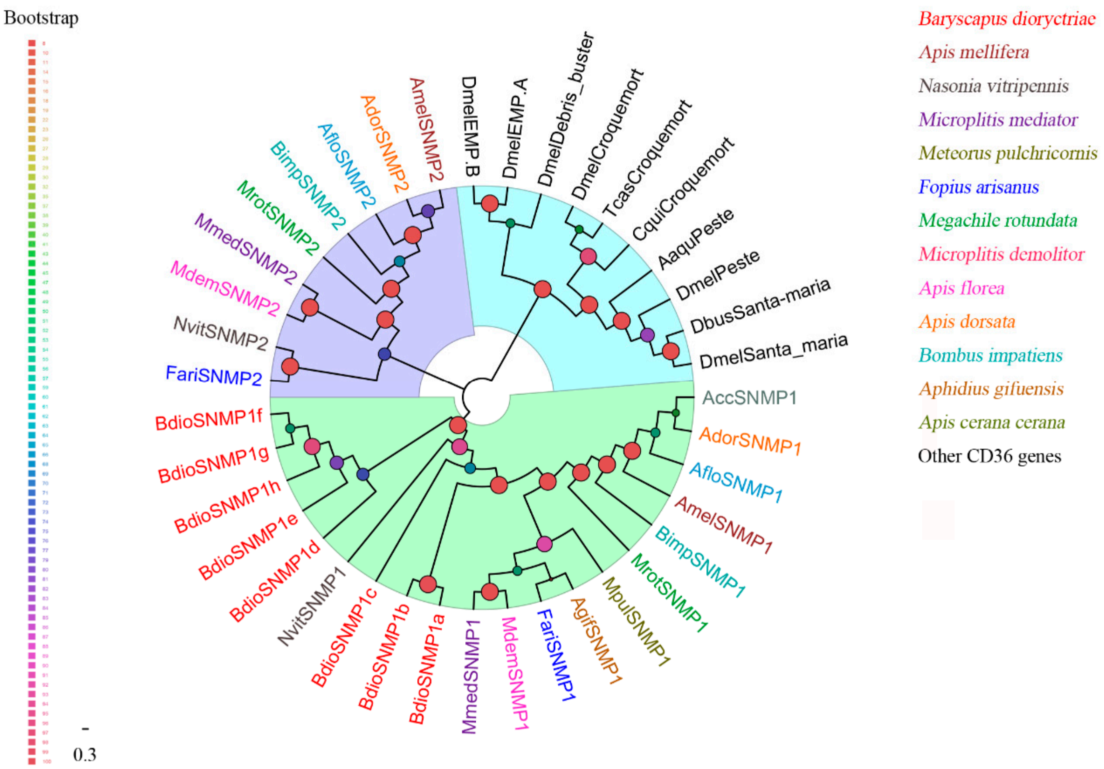

Figure S9. Phylogenetic tree of 29 SNMPs from 13 Hymenoptera species and 10 CD36 homologs (including epithelial membrane protein, croquemort, peste, santa maria and debris buster) from Drosophila melanogaster, D. busckii, Tribolium castaneum, Culex quinquefasciatus and Anopheles aquasalis. The protein sequences used in this phylogenetic analysis are listed in File S7.

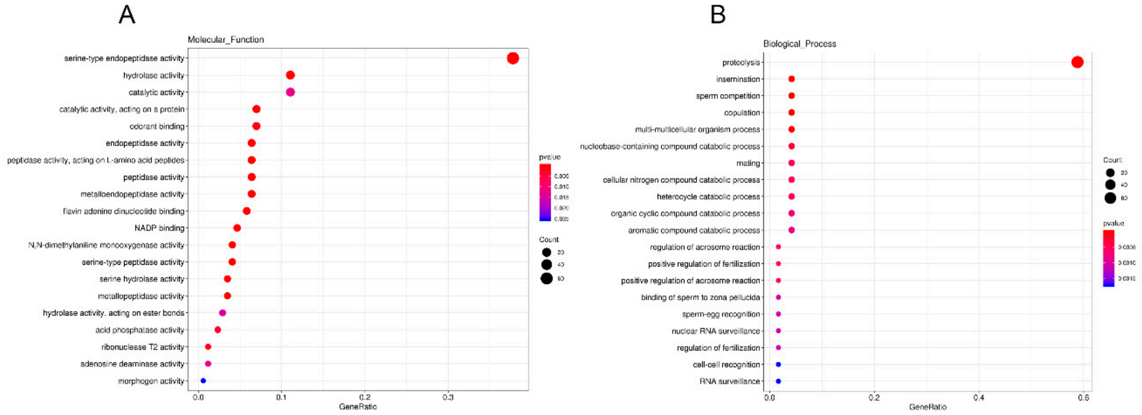

Figure S10. GO enrichment of 377 unigenes upregulated in Fov.

Table S1. Assembly of unigenes and transcripts of *B. dioryctriae.*

Length Range	Transcript	Unigene
300-500	26,853(9.87%)	19,858(41.71%)
500-1000	27,491(10.11%)	12,753(26.79%)
1000-2000	36,840(13.54%)	6,749(14.18%)
2000+	180,752(66.46%)	8,245(17.32%)
Total Number	271,986	47,607
Total Length	1,129,693,305	63,738,476
N50 Length	6,428	2,801
Mean Length	4153.5	1338.85

Table S2. Annotation of *B.dioryctriae* with BLAST.

Anno_Database	Annotated_Number	300<=length<1000	length>=1000
COG_Annotation	6,051	2,668	3,383
GO_Annotation	15,996	7,239	8,751
KEGG_Annotation	13,689	5,510	8,179
KOG_Annotation	11,591	4,922	6,669
Pfam_Annotation	15,347	6,445	8,902
Swissprot_Annotation	9,038	3,405	5,633
TrEMBL_Annotation	19,451	8,938	10,513
eggNOG_Annotation	14,772	6,025	8,747
nr_Annotation	18,736	8,211	10,525
All_Annotated	20,982	10,116	10,860

Table S3. Sequence information of identified *OBPs in B.dioryctriae.*

Gene Name	ORF (aa)	Sp (aa)	Full-Length	pI	Mw(Da）	BLASTX best hit			
						Sequence ID	Query (%)	E-value	Identify (%)
BdioOBP1	153	19	Yes	5.64	15362.88	CCD17777.1, putative odorant binding protein 8 [Nasonia vitripennis]	92%	2E-24	40.77%
BdioOBP2	145	23	Yes	8.82	16240.98	XP_016842824.1, general odorant-binding protein 83a [Nasonia vitripennis]	93%	1E-37	50.74%
BdioOBP3	142	17	Yes	4.86	16064.3	QGW50316.1, odorant-binding protein 21 [Chouioia cunea]	100%	1E-66	72.03%
BdioOBP4	141	0	No	4.47	16052.18	QGW50325.1, odorant-binding protein 30 [Chouioia cunea]	78%	2.E-08	36.94%
BdioOBP5	139	23	No	5.47	17447.96	CCD17832.1, putative odorant binding protein 63 [Nasonia vitripennis]	76%	3.E-14	76.00%
BdioOBP6	138	20	Yes	6.56	15414.85	QGW50329.1, odorant-binding protein 34 [Chouioia cunea]	97%	3E-82	87.41%
BdioOBP7	137	20	Yes	5.88	15530.93	QGW50307.1, odorant-binding protein 12 [Chouioia cunea]	100%	1E-80	87.59%
BdioOBP8	136	20	Yes	6.19	14918.19	QGW50300.1, odorant-binding protein 5 [Chouioia cunea]	97%	2E-35	47.73%
BdioOBP9	135	17	No	4.62	15419.58	QGW50311.1, odorant-binding protein 16 [Chouioia cunea]	62%	2E-14	37.21%
BdioOBP10	134	17	No	8.44	14350.97	XP_001601068.1, general odorant-binding protein 56d [Nasonia vitripennis]	100%	2E-66	71.64%
BdioOBP11	133	20	Yes	4.76	15184.63	QGW50302.1, odorant-binding protein 7 [Chouioia cunea]	100%	8E-54	67.67%

BdioOBP12	132	0	Yes	4.35	14819.91	QGW50306.1, odorant-binding protein 11 [Chouioia cunea]	96%	1E-80	87.40%
BdioOBP13	132	19	Yes	5.63	14696.91	QGW50325.1, odorant-binding protein 30 [Chouioia cunea]	92%	2E-34	49.18%
BdioOBP14	132	19	Yes	4.83	14875.24	QGW50325.1, odorant-binding protein 30 [Chouioia cunea]	98%	2E-43	63.85%
BdioOBP15	131	19	Yes	4.88	15028.28	QGW50325.1, odorant-binding protein 30 [Chouioia cunea]	99%	6E-40	53.08%
BdioOBP16	130	19	No	4.61	14662.85	QGW50325.1, odorant-binding protein 30 [Chouioia cunea]	98%	9E-56	67.19%
BdioOBP17	130	27	Yes	8.47	14820.24	CCD17777.1, putative odorant binding protein 8 [Nasonia vitripennis]	56%	3E-13	45.95%
BdioOBP18	125	17	Yes	8.67	13929.48	ANG59312.1,odorant binding proteins OBP1 [Chouioia cunea]	87%	3E-70	95.41%
BdioOBP19	123	17	No	6.06	13928.3	QGW50328.1, odorant-binding protein 33 [Chouioia cunea]	100%	2E-43	56.82%
BdioOBP20	122	18	No	6.3	13350.81	QGW50304.1, odorant-binding protein 9 [Chouioia cunea]	86%	1E-39	66.98%
BdioOBP21	117	19	No	7.52	13173.29	QGW50311.1, odorant-binding protein 16 [Chouioia cunea]	98%	1E-27	45.22%
BdioOBP22	116	0	Yes	6.54	12825.98	QGW50299.1, odorant-binding protein 3 [Chouioia cunea]	100%	8E-63	74.14%
BdioOBP23	115	17	No	4.68	13214.17	ANG59312.1,odorant binding proteins OBP1 [Chouioia cunea]	87%	3E-70	95.41%
BdioOBP24	101	0	No	8.27	10927.76	QGW50298.1, odorant-binding protein 2 [Chouioia cunea]	95%	7E-35	79.17%
BdioOBP25	97	0	No	4.58	10665.23	QGW50305.1, odorant-binding protein 10 [Chouioia cunea]	97%	1E-33	58.95%

BdioOBP26	95	0	No	6.3	10644.42	QGW50323.1, odorant-binding protein 28 [Chouioia cunea]	94%	4E-17	38.89%
BdioOBP27	83	0	No	10.03	8767.44	QGW50303.1, odorant-binding protein 8 [Chouioia cunea]	40%	2.00E-11	94.12%

Table S4. Sequence information of identified CSPs in *B.dioryctriae.*

Gene Name	ORF (aa)	Sp (aa)	Full-Length	pI	Mw(Da）	BLASTX best hit			
						**Sequence ID**	**Query (%)**	**E-value**	**Identify (%)**
BdioCSP1	156	23	Yes	8.17	17825.78	QGW50252.1, chemosensory protein 5 [Chouioia cunea]	100%	2E-66	62.82%
BdioCSP2	154	22	Yes	6.12	17803.82	QGW50250.1, chemosensory protein 3 [Chouioia cunea]	85%	6E-68	75.57%
BdioCSP3	133	0	No	10.36	14561.11	QGW50250.1, chemosensory protein 4 [Chouioia cunea]	33%	8E-10	75.00%
BdioCSP4	128	20	Yes	5.45	14899.89	QJT73561.1, chemosensory protein 2 [Encarsia formosa]	89%	6E-41	53.91%
BdioCSP5	121	16	Yes	5.06	13879.91	NP_001011583.1, chemosensory protein 7 [Chouioia cunea]	86%	2E-45	83.53%
BdioCSP6	114	16	Yes	5.36	13316.3	QGW50250.1, chemosensory protein 2 [Chouioia cunea]	98%	3E-49	80.53%
BdioCSP7	109	23	No	9.55	12645.75	QGW50250.1, chemosensory protein 3 [Chouioia cunea]	82%	5.E-17	38.89%
BdioCSP8	105	20	Yes	9.85	11905.33	QGW50251.1, chemosensory protein 7 [Chouioia cunea]	80%	2E-45	83.53%
BdioCSP9	89	23	No	8.88	10232.13	QGW50250.1chemosensory protein 3 [Chouioia cunea]	58%	9E-13	55.77%

Table S5. Sequence information of identified NPC2s in *B.dioryctriae.*

Gene Name	ORF (aa)	Sp (aa)	Full-Length	pI	Mw(Da)	BLASTX best hit			
						Sequence ID	Query (%)	E-value	Identify (%)
BdioNPC2a	178	18	Yes	6.29	20165.52	XP_018572438.1, protein NPC2 homolog [Anoplophora glabripennis]	87%	1E-57	64.57%
BdioNPC2b	152	19	Yes	4.02	16837.22	XP_044266260.1, NPC intracellular cholesterol transporter 2 homolog a isoform X1 [Tribolium madens]	96%	2E-59	59.06%
BdioNPC2c	149	17	Yes	5.47	15810.31	XP_016841539.1, NPC intracellular cholesterol transporter 2 homolog a [Nasonia vitripennis]	87%	1E-57	85.50%

Table S6. Sequence information of identified *ORs in B.dioryctriae*

Gene Name	ORF (aa)	Full-Length	TMD	BLASTX best hit			
				Sequence ID	Query (%)	E-value	Identify (%)
BdioOrco	475	Yes	7	AIY24336.1, odorant receptor Or83b [Chouioia cunea]	100.00%	0	94.95%
BdioOR1	467	Yes	7	NP_001177544.1, odorant receptor 142 [Nasonia vitripennis]	83.00%	3E-147	53.30%
BdioOR2	440	Yes	6	NP_001177473.1, odorant receptor 17 [Nasonia vitripennis]	91.00%	2.E-152	53.07%
BdioOR3	431	Yes	7	NP_001164671.1, odorant receptor 77 [Nasonia vitripennis]	98.00%	2E-79	33.64%
BdioOR4	424	Yes	7	XP_012345844.1, odorant receptor 67c-like [Apis florea]	91.00%	5E-66	35.22%
BdioOR5	422	Yes	6	NP_001177429.1, odorant receptor 2 [Nasonia vitripennis]	100.00%	0	76.07%
BdioOR6	422	Yes	6	XP_014238328.2, odorant receptor 22c-like [Trichogramma pretiosum]	91%	3E-91	41.97%
BdioOR7	418	Yes	7	ANG59311.1, odorant receptor Or1 [Chouioia cunea]	100.00%	0	88.07%
BdioOR8	418	Yes	7	QGW50394.1, odorant receptor 67 [Chouioia cunea]	54%	1E-46	36.84%
BdioOR9	417	Yes	7	NP_001164404.1, odorant receptor 141 [Nasonia vitripennis]	100%	2E-79	34.52%
BdioOR10	413	Yes	7	NP_001177544.1, odorant receptor 142 [Nasonia vitripennis]	93.00%	8E-64	33.16%

BdioOR11	410	Yes	7	NP_001177605.1, odorant receptor 264 [Nasonia vitripennis]	99.00%	0	65.20%
BdioOR12	410	Yes	6	NP_001177618.1, odorant receptor 285 [Nasonia vitripennis]	97.00%	3E-124	46.65%
BdioOR13	408	Yes	6	NP_001177574.1, odorant receptor 202 [Nasonia vitripennis]	99.00%	4E-128	47.30%
BdioOR14	407	Yes	7	NP_001177467.1, odorant receptor 10 [Nasonia vitripennis]	93.00%	5E-105	43.93%
BdioOR15	406	No	6	NP_001177501.1, odorant receptor 60 [Nasonia vitripennis]	99.00%	3E-177	60.49%
BdioOR16	405	Yes	6	XP_023315030.1, odorant receptor 4-like [Trichogramma pretiosum]	98.00%	3E-81	36.50%
BdioOR17	404	Yes	7	XP_015438926.1PREDICTED: odorant receptor 47a-like [Dufourea novaeangliae]	98.00%	9E-79	32.84%
BdioOR18	403	Yes	7	QGW50387.1, odorant receptor 60 [Chouioia cunea]	73%	6E-178	85.08%
BdioOR19	400	Yes	6	NP_001164671.1, odorant receptor 77 [Nasonia vitripennis]	99.00%	1E-64	35.22%
BdioOR20	399	Yes	8	NP_001177519.1, odorant receptor 100 [Nasonia vitripennis]	99.00%	1E-151	56.64%
BdioOR21	398	Yes	6	NP_001177567.1, odorant receptor 191 [Nasonia vitripennis]	100.00%	7E-163	56.78%
BdioOR22	398	Yes	7	NP_001164671.1, odorant receptor 77 [Nasonia vitripennis]	99.00%	4E-79	34.09%
BdioOR23	396	Yes	6	NP_001164405.1, odorant receptor 154 [Nasonia vitripennis]	98.00%	1E-127	53.18%
BdioOR24	396	No	7	NP_001177529.1, odorant receptor 115 [Nasonia vitripennis]	96.00%	5E-98	41.09%
BdioOR25	396	Yes	6	NP_001177473.1, odorant receptor 17 [Nasonia vitripennis]	99.00%	2E-86	36.65%
BdioOR27	395	Yes	6	QGW50342.1, odorant receptor 14 [Chouioia cunea]	69.00%	1E-73	47.29%
BdioOR26	395	Yes	6	QGW50398.1, odorant receptor 72 [Chouioia cunea]	98.00%	7E-99	39.90%
BdioOR28	394	No	6	XP_034180494.1, odorant receptor 67c-like [Osmia lignaria]	98.00%	3E-79	35.01%
BdioOR29	394	Yes	7	NP_001177505.1, odorant receptor 65 [Nasonia vitripennis]	95.00%	2E-51	31.40%
BdioOR30	392	Yes	7	XP_016845831.1, odorant receptor 207 isoform X2 [Nasonia vitripennis]	97.00%	2E-36	26.52%
BdioOR31	391	Yes	7	XP_016845869.1, odorant receptor 232 isoform X1 [Nasonia vitripennis]	98.00%	3E-68	33.83%
BdioOR32	390	Yes	7	NP_001177534.1, odorant receptor 125 [Nasonia vitripennis]	93.00%	3E-152	57.10%
BdioOR33	390	Yes	7	NP_001177547.1, odorant receptor 146 [Nasonia vitripennis]	99%	4E-144	50.77%
BdioOR34	389	Yes	7	NP_001177541.1, odorant receptor 137 [Nasonia vitripennis]	100.00%	3E-127	46.82%
BdioOR35	389	Yes	7	NP_001164404.1, odorant receptor 141 [Nasonia vitripennis]	100.00%	1E-76	35.11%

BdioOR36	388	Yes	7	QGW50355.1, odorant receptor 27 [Chouioia cunea]	98.00%	4E-117	45.14%
BdioOR37	388	Yes	6	QGW50398.1, odorant receptor 72 [Chouioia cunea]	100.00%	2E-86	36.39%
BdioOR38	387	Yes	6	NP_001177480.1, odorant receptor 25 [Nasonia vitripennis]	99.00%	0	65.12%
BdioOR39	387	Yes	7	XP_031784326.1, odorant receptor 175 isoform X1 [Nasonia vitripennis]	97.00%	3E-148	55.32%
BdioOR40	387	Yes	8	NP_001177699.1, odorant receptor 96 [Nasonia vitripennis]	98.00%	3E-94	40.67%
BdioOR42	387	Yes	6	NP_001164404.1, odorant receptor 141 [Nasonia vitripennis]	100.00%	2E-95	38.07%
BdioOR41	387	Yes	7	NP_001164404.1, odorant receptor 141 [Nasonia vitripennis]	100.00%	3E-71	34.43%
BdioOR43	385	Yes	7	QGW50359.1, odorant receptor 31 [Chouioia cunea]	100.00%	0	78.70%
BdioOR44	385	Yes	6	NP_001177475.1, odorant receptor 20 [Nasonia vitripennis]	99.00%	2E-147	53.89%
BdioOR45	385	Yes	7	NP_001177588.1, odorant receptor 229 [Nasonia vitripennis]	98%	2E-99	44.44%
BdioOR46	381	Yes	8	NP_001177699.1, odorant receptor 96 [Nasonia vitripennis]	97.00%	1E-86	35.86%
BdioOR47	378	Yes	6	QGW50398.1, odorant receptor 72 [Chouioia cunea]	100.00%	4E-93	40.82%
BdioOR48	377	Yes	6	NP_001177699.1, odorant receptor 96 [Nasonia vitripennis]	100.00%	4E-155	59.07%
BdioOR49	377	Yes	7	NP_001177496.1, odorant receptor 50 [Nasonia vitripennis]	99.00%	7E-61	34.75%
BdioOR50	375	Yes	6	XP_014238328.2, odorant receptor 22c-like [Trichogramma pretiosum]	86.00%	9E-51	36.86%
BdioOR51	372	Yes	7	QGW50402.1, odorant receptor 77 [Chouioia cunea]	58.00%	7E-110	77.52%
BdioOR52	371	Yes	7	NP_001177536.1, odorant receptor 128 [Nasonia vitripennis]	99.00%	1E-69	33.33%
BdioOR53	367	Yes	5	XP_016845881.1, odorant receptor 233 isoform X1 [Nasonia vitripennis]	77%	2E-45	34.60%
BdioOR54	366	Yes	7	QHN69155.1, odorant receptor 24 [Sirex nitobei]	98.00%	3E-58	30.05%
BdioOR55	356	Yes	5	XP_016836498.1, odorant receptor 261 isoform X3 [Nasonia vitripennis]	78%	3E-61	36.33%
BdioOR56	354	Yes	6	XP_016845831.1, odorant receptor 207 isoform X2 [Nasonia vitripennis]	95.00%	3E-36	28.45%
BdioOR57	352	Yes	6	NP_001177473.1, odorant receptor 17 [Nasonia vitripennis]	94.00%	4E-142	69.35%
BdioOR58	350	Yes	6	NP_001177513.1, odorant receptor 87 [Nasonia vitripennis]	93.00%	3E-139	59.82%
BdioOR59	340	Yes	5	QHN69115.1, odorant receptor 28a [Sirex noctilio]	64.00%	8E-14	25.11%

BdioOR60	327	Yes	6	NP_001177541.1, odorant receptor 137 [Nasonia vitripennis]	99%	2E-67	34.56%
BdioOR61	323	Yes	6	XP_031785566.2, odorant receptor 46a isoform X1 [Nasonia vitripennis]	98.00%	3E-115	52.50%
BdioOR62	322	Yes	6	NP_001177506.1, odorant receptor 66 [Nasonia vitripennis]	93.00%	3E-64	40.73%
BdioOR63	321	Yes	5	XP_014215181.2, odorant receptor 42a-like [Copidosoma floridanum]	97.00%	8E-69	38.87%
BdioOR64	319	No	6	QGW50355.1, odorant receptor 27 [Chouioia cunea]	100.00%	2E-82	44.24%
BdioOR65	315	Yes	5	NP_001177473.1, odorant receptor 17 [Nasonia vitripennis]	97.00%	3E-75	42.49%
BdioOR66	308	No	5	NP_001177705.1, odorant receptor 190 [Nasonia vitripennis]	99.00%	2E-87	46.25%
BdioOR67	307	Yes	5	NP_001177473.1, odorant receptor 17 [Nasonia vitripennis]	94.00%	4E-57	35.74%
BdioOR68	302	No	4	NP_001177483.1, odorant receptor 28 [Nasonia vitripennis]	99.00%	2E-138	64.69%
BdioOR70	301	Yes	5	NP_001177511.1, odorant receptor 79 [Nasonia vitripennis]	97.00%	2E-98	58.61%
BdioOR69	301	No	4	NP_001177476.1, odorant receptor 21 [Nasonia vitripennis]	99.00%	3E-108	55.63%
BdioOR71	301	Yes	5	NP_001164404.1, odorant receptor 141 [Nasonia vitripennis]	100.00%	4E-65	36.96%
BdioOR72	298	Yes	5	NP_001177536.1, odorant receptor 128 [Nasonia vitripennis]	70.00%	2E-49	38.57%
BdioOR73	293	Yes	5	QGW50376.1, odorant receptor 49 [Chouioia cunea]	86.00%	5E-130	71.65%
BdioOR74	293	Yes	5	NP_001177543.1, odorant receptor 140 [Nasonia vitripennis]	90.00%	2E-52	38.38%
BdioOR75	290	No	4	XP_016845869.1, odorant receptor 232 isoform X1 [Nasonia vitripennis]	87.00%	4E-28	32.06%
BdioOR76	288	No	6	QGW50405.1, odorant receptor 81 [Chouioia cunea]	100.00%	6E-96	52.43%
BdioOR77	285	Yes	5	QGW50334.1, odorant receptor 4 [Chouioia cunea]	100.00%	5E-126	61.49%
BdioOR78	285	Yes	5	QGW50398.1, odorant receptor 72 [Chouioia cunea]	98.00%	5E-58	33.68%
BdioOR79	284	No	4	XP_031777526.1, odorant receptor 229 isoform X1 [Nasonia vitripennis]	95.00%	2E-55	38.93%
BdioOR80	281	No	4	NP_001177508.1, odorant receptor 68 [Nasonia vitripennis]	99.00%	3E-91	51.60%
BdioOR81	278	Yes	5	NP_001177477.1, odorant receptor 22 [Nasonia vitripennis]	96.00%	2E-97	60.22%
BdioOR82	278	No	4	QGW50401.1, odorant receptor 76 [Chouioia cunea]	85.00%	1E-39	34.60%
BdioOR83	277	Yes	5	XP_014238328.2, odorant receptor 22c-like [Trichogramma pretiosum]	100.00%	9E-50	36.86%

BdioOR84	277	Yes	5	NP_001177552.1, odorant receptor 161 [Nasonia vitripennis]	89.00%	1E-27	31.50%
BdioOR85	276	No	5	XP_033208298.1, odorant receptor 46a-like [Belonocnema kinseyi]	95.00%	4E-45	36.06%
BdioOR86	276	No	4	XP_031786746.1, odorant receptor 22 isoform X1 [Nasonia vitripennis]	88.00%	1E-35	35.80%
BdioOR87	276	No	4	XP_016845831.1, odorant receptor 207 isoform X2 [Nasonia vitripennis]	98.00%	8E-28	28.27%
BdioOR88	273	Yes	5	NP_001164458.1, odorant receptor 98 [Nasonia vitripennis]	97.00%	4E-145	76.03%
BdioOR89	273	No	3	NP_001177487.1, odorant receptor 36 [Nasonia vitripennis]	35.00%	4E-14	46.88%
BdioOR90	269	No	4	QGW50397.1, odorant receptor 70 [Chouioia cunea]	60.00%	2E-69	65.85%
BdioOR91	269	Yes	6	QGW50398.1, odorant receptor 72 [Chouioia cunea]	100.00%	2E-51	36.40%
BdioOR92	266	No	4	NP_001164396.1, odorant receptor 80 [Nasonia vitripennis]	80.00%	8E-89	61.86%
BdioOR93	264	No	4	NP_001177566.1, odorant receptor 189 [Nasonia vitripennis]	98.00%	1E-51	36.33%
BdioOR94	264	No	4	NP_001177544.1, odorant receptor 142 [Nasonia vitripennis]	90.00%	1E-09	30.77%
BdioOR95	262	Yes	7	QGW50398.1, odorant receptor 72 [Chouioia cunea]	99.00%	4E-39	32.12%
BdioOR96	261	Yes	5	NP_001164404.1, odorant receptor 141 [Nasonia vitripennis]	96.00%	2E-65	42.02%
BdioOR97	261	Yes	5	QGW50394.1, odorant receptor 67 [Chouioia cunea]	87.00%	1E-46	37.12%
BdioOR98	260	Yes	5	QGW50398.1, odorant receptor 72 [Chouioia cunea]	95.00%	1E-38	31.20%
BdioOR99	258	No	4	XP_016845869.1, odorant receptor 232 isoform X1 [Nasonia vitripennis]	97.00%	2E-43	35.00%
BdioOR100	257	No	4	XP_014215181.2, odorant receptor 42a-like [Copidosoma floridanum]	100.00%	9E-56	37.21%
BdioOR101	256	No	4	XP_031784325.1, odorant receptor 166 isoform X1 [Nasonia vitripennis]	90.00%	3E-64	51.07%
BdioOR102	256	No	4	XP_016845869.1, odorant receptor 232 isoform X1 [Nasonia vitripennis]	93.00%	4E-40	34.51%
BdioOR103	252	No	4	QGW50380.1, odorant receptor 53 [Chouioia cunea]	100.00%	3E-136	73.73%
BdioOR104	248	No	4	NP_001177435.1, odorant receptor 9 [Nasonia vitripennis]	100.00%	6E-79	54.58%
BdioOR105	246	No	4	AXM05155.1, odorant receptor 32[Campoletis chlorideae]	97.00%	5E-55	40.57%

BdioOR106	244	No	4	NP_001177496.1, odorant receptor 50 [Nasonia vitripennis]	100.00%	7E-52	38.52%
BdioOR107	243	No	3	NP_001177703.1, odorant receptor 160 [Nasonia vitripennis]	54.00%	1E-17	42.86%
BdioOR108	242	No	4	NP_001177543.1, odorant receptor 140 [Nasonia vitripennis]	88.00%	3E-51	40.55%
BdioOR109	239	No	4	QGW50356.1, odorant receptor 28 [Chouioia cunea]	46.00%	2E-10	28.83%
BdioOR111	237	No	4	QGW50338.1, odorant receptor 9 [Chouioia cunea]	56.00%	1E-66	74.07%
BdioOR110	237	No	4	NP_001177575.1, odorant receptor 203 [Nasonia vitripennis]	98.00%	2E-82	54.70%
BdioOR112	233	No	4	QGW50394.1, odorant receptor 67 [Chouioia cunea]	95.00%	5E-40	37.78%
BdioOR113	229	No	4	XP_014238328.2, odorant receptor 22c-like [Trichogramma pretiosum]	95.00%	7E-61	50.00%
BdioOR115	227	No	4	QGW50345.1, odorant receptor 17 [Chouioia cunea]	69.00%	3E-21	56.98%
BdioOR114	227	No	3	QGW50394.1, odorant receptor 67 [Chouioia cunea]	97.00%	5E-45	39.56%
BdioOR116	219	No	4	QGW50358.1, odorant receptor 30 [Chouioia cunea]	88.00%	1E-109	79.27%
BdioOR117	217	No	4	QGW50394.1, odorant receptor 67 [Chouioia cunea]	99.00%	3E-43	40.28%
BdioOR118	217	No	4	XP_031786757.1, odorant receptor 125 isoform X1 [Nasonia vitripennis]	100.00%	8E-30	35.62%
BdioOR119	217	No	4	NP_001177543.1, odorant receptor 140 [Nasonia vitripennis]	92.00%	3E-29	33.17%
BdioOR120	216	No	3	XP_032455989.1, odorant receptor 14 isoform X1 [Nasonia vitripennis]	91.00%	5E-39	41.38%
BdioOR121	215	No	4	QGW50398.1, odorant receptor 72 [Chouioia cunea]	80.00%	4E-36	39.20%
BdioOR122	213	No	5	NP_001177590.1, odorant receptor 232 [Nasonia vitripennis]	99.00%	2E-26	32.43%
BdioOR123	211	No	4	QGW50355.1, odorant receptor 27 [Chouioia cunea]	95.00%	2E-99	74.26%
BdioOR124	211	No	3	NP_001177550.1, odorant receptor 157 [Nasonia vitripennis]	100.00%	4E-15	24.88%
BdioOR125	209	No	4	NP_001177703.1, odorant receptor 160 [Nasonia vitripennis]	98.00%	6E-76	60.19%
BdioOR126	208	No	4	QGW50405.1, odorant receptor 81 [Chouioia cunea]	100.00%	2E-31	48.56%
BdioOR127	203	No	3	NP_001164401.1, odorant receptor 105 [Nasonia vitripennis]	99.00%	6E-91	66.83%
BdioOR128	203	No	4	XP_016845881.1, odorant receptor 233 isoform X1 [Nasonia vitripennis]	99.00%	1E-52	43.41%
BdioOR129	197	No	4	NP_001177528.1, odorant receptor 114 [Nasonia vitripennis]	94.00%	2E-49	46.32%
BdioOR130	196	No	2	NP_001177478.1, odorant receptor 23 [Nasonia vitripennis]	69.00%	5E-14	33.33%

BdioOR131	196	No	3	XP_023247295.1, odorant receptor 63a-like [Copidosoma floridanum]	82.00%	8E-13	28.14%
BdioOR132	195	No	3	NP_001177473.1, odorant receptor 17 [Nasonia vitripennis]	99.00%	4E-39	41.24%
BdioOR133	192	No	4	XP_031777522.1, odorant receptor 137 isoform X2 [Nasonia vitripennis]	98.00%	4E-43	38.42%
BdioOR134	190	No	3	XP_031789370.1, odorant receptor 170 isoform X2 [Nasonia vitripennis]	97.00%	2E-36	40.54%
BdioOR135	190	No	3	NP_001177705.1, odorant receptor 190 [Nasonia vitripennis]	87.00%	8E-34	38.55%
BdioOR136	188	No	3	QGW50404.1, odorant receptor 80 [Chouioia cunea]	61.00%	2E-10	33.90%
BdioOR137	187	No	2	QGW50394.1, odorant receptor 67 [Chouioia cunea]	93.00%	2E-35	40.00%
BdioOR138	187	No	3	NP_001164404.1, odorant receptor 141 [Nasonia vitripennis]	87.00%	6E-23	32.52%
BdioOR139	185	No	3	NP_001177565.1, odorant receptor 188 [Nasonia vitripennis]	96.00%	8E-35	41.57%
BdioOR140	184	No	4	NP_001177492.1, odorant receptor 45 [Nasonia vitripennis]	98.00%	6E-64	58.24%
BdioOR141	181	No	2	NP_001177588.1, odorant receptor 229 [Nasonia vitripennis]	100.00%	2E-50	50.83%
BdioOR142	180	No	3	NP_001177544.1, odorant receptor 142 [Nasonia vitripennis]	99.00%	2E-27	33.70%
BdioOR143	179	No	3	NP_001177529.1, odorant receptor 115 [Nasonia vitripennis]	96.00%	3E-33	35.47%
BdioOR144	175	No	3	QGW50394.1, odorant receptor 67 [Chouioia cunea]	93.00%	8E-24	36.14%
BdioOR145	174	No	2	NP_001177499.1, odorant receptor 56 [Nasonia vitripennis]	54.00%	1E-06	43.16%
BdioOR146	173	No	3	NP_001177543.1, odorant receptor 140 [Nasonia vitripennis]	93.00%	3E-38	50.30%
BdioOR147	173	No	0	XP_016845881.1, odorant receptor 233 isoform X1 [Nasonia vitripennis]	74.00%	9E-25	38.64%
BdioOR148	173	No	2	XP_035727254.1, odorant receptor 22c-like [Vespa mandarinia]	100.00%	1E-23	34.08%
BdioOR149	170	No	0	XP_031781937.1, odorant receptor 62 isoform X2 [Nasonia vitripennis]	98.00%	6E-52	51.76%
BdioOR150	170	No	2	NP_001177588.1, odorant receptor 229 [Nasonia vitripennis]	95.00%	2E-48	47.24%
BdioOR152	169	No	4	NP_001164404.1, odorant receptor 141 [Nasonia vitripennis]	66.00%	4E-14	35.09%
BdioOR151	169	No	4	XP_043281331.1, odorant receptor Or2-like isoform X1 [Venturia canescens]	89.00%	8E-18	30.26%

BdioOR153	167	No	2	XP_031781937.1, odorant receptor 62 isoform X2 [Nasonia vitripennis]	100.00%	1E-73	66.47%
BdioOR154	166	No	4	NP_001177544.1, odorant receptor 142 [Nasonia vitripennis]	77.00%	2E-36	51.54%

Table S7. Sequence information of identified IRs *in B.dioryctriae*

Gene Name	ORF (aa)	Full-Length	TMD	BLASTX best hit			
				Sequence ID	Query (%)	E-value	Identify (%)
BdioIR21a.1	684	Yes	4	XP_016844519.1, ionotropic receptor 21a isoform X1 [Nasonia vitripennis]	89%	0	58.28%
BdioIR21a.2	224	No	2	XP_023247289.1, ionotropic receptor 40a-like [Copidosoma floridanum]	100%	9E-93	65.93%
BdioIR25a	937	Yes	3	XP_016841447.1, ionotropic receptor 25a isoform X1 [Nasonia vitripennis]	97%	0	75.19%
BdioIR25a.2	486	NO	3	XP_001603675.1, ionotropic receptor 25a-like [Nasonia vitripennis]	93%	0	64.10%
BdioIR75a.1	546	No	2	XP_031780582.1, ionotropic receptor 75a-like [Nasonia vitripennis]	98%	0	66.36%
BdioIR75a.10	252	No	2	XP_031777892.1, ionotropic receptor 75a-like [Nasonia vitripennis]	79%	4E-83	65.00%
BdioIR75a.2	552	NO	3	XP_031780582.1, ionotropic receptor 75a-like [Nasonia vitripennis]	92%	7E-166	47.46%
BdioIR75a.3	324	NO	3	XP_016841447.1, ionotropic receptor 25a isoform X1 [Nasonia vitripennis]	97%	0	75.19%
BdioIR75a.4	205	No	2	XP_031777892.1, ionotropic receptor 75a-like [Nasonia vitripennis]	88%	9E-74	60.77%
BdioIR75a.5	220	No	1	XP_016843278.1, ionotropic receptor 75a isoform X1 [Nasonia vitripennis]	99%	1E-89	61.19%

BdioIR75a.6	302	No	2	XP_031777892.1, ionotropic receptor 75a-like [Nasonia vitripennis]	93%	5E-107	56.34%
BdioIR75a.7	228	No	1	XP_031777892.1, ionotropic receptor 75a-like [Nasonia vitripennis]	92%	5E-73	56.60%
BdioIR75a.8	305	NO	3	XP_031777892.1, ionotropic receptor 75a-like [Nasonia vitripennis]	77%	2E-93	56.54%
BdioIR75a.9	624	Yes	4	XP_016843290.1, ionotropic receptor 75a-like isoform X1 [Nasonia vitripennis]	85%	0	49.81%
BdioIR75b	133	No	0	XP_016843278.1, ionotropic receptor 75a isoform X1 [Nasonia vitripennis]	93%	1E-22	41.73%
BdioIR75d	206	No	0	XP_031777891.1, ionotropic receptor 75a-like isoform X2 [Nasonia vitripennis]	95%	9E-66	48.24%
BdioIR75e	327	No	2	XP_031777891.1, ionotropic receptor 75a-like isoform X2 [Nasonia vitripennis]	93%	2E-82	43.69%
BdioIR75u	628	Yes	3	QGW50289.1, ionotropic receptor 75q2 [Chouioia cunea]	100%	0	75.39%
BdioIR8a	921	Yes	3	QGW50292.1, ionotropic receptor 8a [Chouioia cunea]	99%	0	82.12%
BdioIR93a	806	No	2	QGW50291.1, ionotropic receptor 93a [Chouioia cunea]	98%	0	77.57%
BdioIR75c	250	No	0	XP_016843278.1, ionotropic receptor 75a isoform X1 [Nasonia vitripennis]	86%	3E-48	40.72%
BdioIR1	106	No	0	RLZ02186.1, ionotropic receptor 117 [Cephus cinctus]	97%	8E-08	31.43%
BdioIR2	127	No	0	XP_031777891.1, ionotropic receptor 75a-like isoform X2 [Nasonia vitripennis]	94%	6E-14	37.19%

Table S8. Sequence information of identified GRs in *B.dioryctriae.*

Gene Name	ORF (aa)	Full-length	TMD	BLASTX best hit			
				Sequence ID	Query (%)	E-value	Identify (%)

BdioGR43a1	508	Yes	7	XP_014219005.1, gustatory receptor for sugar taste 43a-like [Copidosoma floridanum]	94%	2E-165	52.61%
BdioGR43a2	196	No	3	QAY30709.1, gustatory receptor 43a [Trichogramma chilonis]	83%	2E-62	61.59%
BdioGR43a3	146	No	2	QAY30709.1, gustatory receptor 43a [Trichogramma chilonis]	79%	2E-54	73.28%
BdioGR43a4	159	No	3	NP_001164386.1, gustatory receptor 3 [Nasonia vitripennis]	99%	8E-71	70.89%
BdioGR1	97	No	2	QGW50259.1, gustatory receptor 2 [Chouioia cunea]	87%	2E-12	40.00%
BdioGR2	258	Yes	5	QGW50259.1, gustatory receptor 2 [Chouioia cunea]	67%	5E-21	33.52%
BdioGR3	270	Yes	5	QGW50259.1, gustatory receptor 2 [Chouioia cunea]	64%	1E-21	33.33%
BdioGR4	277	No	6	QGW50259.1, gustatory receptor 2 [Chouioia cunea]	0.39	6E-16	34.55%
BdioGR5	215	No	3	NP_001164388.1, gustatory receptor 47 [Nasonia vitripennis]	88%	1E-18	35.60%
BdioGR6	362	Yes	7	QGW50259.1, gustatory receptor 2 [Chouioia cunea]	91%	1E-12	26.35%
BdioGR7	326	Yes	7	NP_001177460.1, gustatory receptor 43 [Nasonia vitripennis]	91%	2E-11	24.68%
BdioGR8	162	No	3	NP_001177449.1, gustatory receptor 30 [Nasonia vitripennis]	98%	1E-21	38.36%
BdioGR9	110	No	3	NP_001164464.1, gustatory receptor 24 [Nasonia vitripennis]	98%	3E-05	31.53%
BdioGR10	366	Yes	7	NP_001177460.1, gustatory receptor 43 [Nasonia vitripennis]	85%	2E-26	27.44%
BdioGR11	374	Yes	7	NP_001177455.1, gustatory receptor 38 [Nasonia vitripennis]	82%	6E-26	30.65%
BdioGR12	208	No	5	NP_001177455.1, gustatory receptor 38 [Nasonia vitripennis]	93%	2E-07	27.00%
BdioGR13	380	Yes	8	XP_016845192.1gustatory receptor 46 isoform X1 [Nasonia vitripennis]	92%	1E-24	26.49%
BdioGR14	114	No	2	NP_001177454.1, gustatory receptor 37 [Nasonia vitripennis]	99%	7E-18	43.36%
BdioGR15	95	No	2	NP_001177449.1, gustatory receptor 30 [Nasonia vitripennis]	85%	1E-09	45.68%
BdioGR16	369	Yes	7	NP_001177449.1, gustatory receptor 30 [Nasonia vitripennis]	92%	3E-17	26.89%
BdioGR17	110	No	2	NP_001177449.1, gustatory receptor 30 [Nasonia vitripennis]	82%	4E-16	46.15%
BdioGR18	232	Yes	5	XP_016845192.1gustatory receptor 46 isoform X1 [Nasonia vitripennis]	97%	7E-15	31.38%
BdioGR19	215	Yes	5	NP_001177444.1, gustatory receptor 22 [Nasonia vitripennis]	90%	1E-12	25.24%
BdioGR20	95	No	0	NP_001177442.1, gustatory receptor 20 [Nasonia vitripennis]	72%	0.019	31.88%
BdioGR21	228	No	4	NP_001177452.1, gustatory receptor 34 [Nasonia vitripennis]	92%	1E-34	37.79%
BdioGR22	123	No	2	NP_001177450.1, gustatory receptor 32 [Nasonia vitripennis]	79%	5E-07	31.58%

BdioGR23	78	No	0	NP_001164388.1, gustatory receptor 47 [Nasonia vitripennis]	80%	3E-08	46.03%
BdioGR24	350	Yes	8	NP_001177460.1, gustatory receptor 43 [Nasonia vitripennis]	86%	4E-11	28.75%
BdioGR25	178	No	4	NP_001177442.1, gustatory receptor 20 [Nasonia vitripennis]	88%	1E-06	30.06%
BdioGR26	129	No	2	NP_001164464.1gustatory receptor 24 [Nasonia vitripennis]	89%	2E-07	36.44%
BdioGR27	142	No	1	QGW50274.1378QGW50274.1Chouioia cuneagustatory receptor 19 [Chouioia cunea]	100%	1E-51	58.04%
BdioGR28	780	No	0	QGW50275.1, gustatory receptor 20 [Chouioia cunea]	80%	0	93.34%
BdioGR29	423	No	4	NP_001177425.1, gustatory receptor 6 [Nasonia vitripennis]	45%	2E-74	62.56%
BdioGR30	235	No	3	XP_031779957.1gustatory receptor 7 isoform X2 [Nasonia vitripennis]	68%	1E-67	64.38%
BdioGR31	205	No	4	NP_001177436.1, gustatory receptor 10 [Nasonia vitripennis]	95%	1E-75	65.48%
BdioGR32	123	No	4	XP_032452205.1, gustatory receptor 58 isoform X1 [Nasonia vitripennis]	75%	1E-11	39.78%
BdioGR33	409	Yes	8	NP_001177466.1, gustatory receptor 53 [Nasonia vitripennis]	98%	4E-74	36.47%
BdioGR34	50	No	0	NP_001177465.1, gustatory receptor 52 [Nasonia vitripennis]	64%	1E-05	68.75%
BdioGR35	243	No	3	XP_014227433.1, gustatory receptor 68a-like [Trichogramma pretiosum]	86%	2E-49	45.91%
BdioGR36	99	No	0	QGW50266.1, gustatory receptor 9 [Chouioia cunea]	91%	5E-14	45.16%
BdioGR37	284	No	4	QGW50266.1, gustatory receptor 9 [Chouioia cunea]	93%	7E-165	84.59%
BdioGR38	141	No	0	QGW50274.1, gustatory receptor 19 [Chouioia cunea]	90%	5E-42	58.14%
BdioGR39	559	No	3	QGW50273.1, gustatory receptor 18 [Chouioia cunea]	0.95	0	98.31%
BdioGR40	570	No	3	QGW50286.1, gustatory receptor 31 [Chouioia cunea]	99%	0	88.18%
BdioGR28b	332	Yes	7	XP_014208041.1，putative gustatory receptor 28b [Copidosoma floridanum]	96%	3E-23	25.31%
BdioGR41	763	No	3	QGW50277.1gustatory receptor 22 [Chouioia cunea]	99%	0.E+00	97.11%
BdioGR42	124	No	1	NP_001177439.1, gustatory receptor 13 [Nasonia vitripennis]	81%	1E-07	33.33%
BdioGR43	315	Yes	6	QGW50285.1, gustatory receptor 30 [Chouioia cunea]	76%	2E-117	70.90%
BdioGR64f	498	Yes	8	XP_008208306.2, gustatory receptor 2 isoform X2 [Nasonia vitripennis]	98%	0	69.64%

Table S9. Sequence information of identified *B.dioryctriae* SNMPs

Gene Name	ORF (aa)	Full-Length	TMD	BLASTX best hit			
				Sequence ID	Query (%)	E-value	Identify (%)
BdioSNMP1b	525	Yes	2	XP_001606602.1, sensory neuron membrane protein 1 [Nasonia vitripennis]	99%	0	66.60%
BdioSNMP1a	520	Yes	2	QGW50408.1, sensory neuron membrane protein s2 [Chouioia cunea]	100%	0	88.08%
BdioSNMP1e	513	Yes	2	XP_008215384.2, sensory neuron membrane protein 1 [Nasonia vitripennis]	97%	1E-163	44.84%
BdioSNMP1h	498	Yes	2	XP_008215384.2, sensory neuron membrane protein 1 [Nasonia vitripennis]	94%	2E-117	37.76%
BdioSNMP1f	448	Yes	2	XP_008215384.2, sensory neuron membrane protein 1 [Nasonia vitripennis]	99%	2E-113	40.76%
BdioSNMP1c	416	No	1	XP_014231879.1, sensory neuron membrane protein 1-like [Trichogramma pretiosum]	99%	2E-162	55.29%
BdioSNMP1g	253	No	1	XP_011499316.1,PREDICTED: sensory neuron membrane protein 1-like [Ceratosolen solmsi marchali]	98%	5E-57	38.74%
BdioSNMP1d	215	No	0	XP_008215384.2, sensory neuron membrane protein 1 [Nasonia vitripennis]	90%	5E-47	41.54%

Table S10. The FPKM values of chemosensory genes in *B.dioryctriae.*

Gene Name	FPKM value									
	FA	FH	Fab	Fov	MA	MH	Mab	Mge	T	L
BdioOBP1	61.22	65.8	39.44	34.85	45.48	62.4	31.61	24.86	28.55	15.93
BdioOBP2	2.16	1.44	0	0	8.03	1.42	0	0	0	0
BdioOBP3	0.13	2.07	8.98	14.06	0.1	3.24	2.74	4.63	0.94	2.63
BdioOBP4	0.52	0.61	1.39	338.48	0.67	7.83	181.71	430.37	0.05	11.97

BdioOBP5	3.8	16.29	4.08	12.32	1.24	15.95	24.63	16.42	14.03	13.66
BdioOBP6	173.98	278.36	6.23	19.62	168.55	152	2.8	20.12	107.14	91.46
BdioOBP7	30535.43	234.69	2.13	4.82	90943.1	264.45	27.45	49.78	8.12	29.03
BdioOBP8	211.59	1587.74	36.73	43.65	75.59	1239.13	11.48	44.59	452.9	272.07
BdioOBP9	1278.88	7.08	0.79	0.06	1727.49	8.12	1.77	1.01	0	1.08
BdioOBP10	96	292.97	80.51	1718.8	96	208	3164.07	847.68	55.26	244.1
BdioOBP11	97.2	181.14	0.81	7.53	65.45	190.27	0.13	9.66	1.13	184.99
BdioOBP12	1.53	6.55	22.82	4991.72	16.91	127.14	5.63	12.23	0.36	68.97
BdioOBP13	1055.75	3.87	0.78	0	541.53	2.97	0	0	0	0.36
BdioOBP14	0	0.65	0.3	98.6	0.1	3.9	1.82	7.11	0	5.73
BdioOBP15	8.85	79.52	0	2.73	386.58	84.52	0.19	2.68	0	11.36
BdioOBP16	0.6	5.72	1.31	31.24	1.01	42.74	1.42	1.34	1.52	6.39
BdioOBP17	0.33	0.72	5.83	606.73	0.13	16.19	9.36	3.65	0.4	12.02
BdioOBP18	974.56	2823.77	14.23	83.61	157.19	1572.94	58.57	43.97	78.18	101.25
BdioOBP19	15.66	82.59	1.16	2.06	11.11	97.31	0	5.6	46.42	15.53
BdioOBP20	4.58	72.18	22.83	73.47	1.95	66.92	178.18	92.5	47.79	50.78
BdioOBP21	1.66	58.81	3.7	76.61	0.57	47.25	30.5	15.25	27.45	32.96
BdioOBP22	5732.13	35.7	0.74	0.42	1685.36	7.45	1	1.16	0	2.32
BdioOBP23	3.07	2.37	0.14	0.67	2.94	3.87	2.03	0.9	0.43	0
BdioOBP24	2.58	14.04	59.01	3552.46	0.88	78.55	244.03	517.78	6.67	96.42
BdioOBP25	3306.95	253.95	7.36	344.12	6532.22	361.54	3.15	21.07	0.32	18.75
BdioOBP26	51.75	39.75	0.17	19.56	18.66	55.18	0.33	1.99	0.37	17.54
BdioOBP27	38.66	356.4	7.86	8.28	13.49	390.86	3.33	11.11	133.72	31.32
BdioCSP1	629.29	584.37	48.96	90.5	203.13	511.81	248.71	375.73	319.23	596.51
BdioCSP2	1240.69	683.9	9.37	911.24	1633.4	938.38	298.63	1075.72	58.48	163.72
BdioCSP3	8.03	1.27	0	0.78	23.66	1.88	0.15	0.15	1.15	1.65
BdioCSP4	2499.23	331.66	12.85	22.99	3124.26	207.9	18.31	7.16	60.82	100.55
BdioCSP5	0	0	0	0	0	0	0	2.72	0	0
BdioCSP6	6	74.44	75.19	91.87	2.8	196.64	425.53	72.74	64.2	58.52

BdioCSP7	1711.75	11.82	0.06	0.49	364.19	8.55	0.64	1.19	0	1.66
BdioCSP8	0.93	0.26	290.2	0.36	0.08	0.5	611.96	1613.51	0.81	54
BdioCSP9	3.46	0.63	7.55	1618.69	0	38.82	0	2.36	0	8.27
BdioNPC2a	0.59	0	33.71	896.91	0	20.65	0	0.23	0	19.15
BdioNPC2b	0.51	0.95	0.89	0.17	0	1.29	0	0	0.36	0
BdioNPC2c	0	0	0	0	0	0	0	0	0.2	0
BdioOrco	875.63	3.22	0.81	0.68	1298.49	2.52	1.64	7.47	0.69	0.08
BdioOR1	28.08	4.58	9.4	2	18.21	3.8	3.03	3.45	2.45	1.46
BdioOR2	13.18	0.09	0.49	19.22	0.17	0.34	0	0.16	0.53	0
BdioOR3	19.13	0.04	0	0	9.81	0.09	0	0	0.04	0
BdioOR4	15.09	0.1	0.03	0	18.79	0.03	0.16	0.12	0	0
BdioOR5	117.7	0.72	0.03	0	83.97	0.61	0.42	0.24	0.25	0
BdioOR6	3.52	0.29	0.02	0	0.55	0.06	0.75	0.22	0	0.44
BdioOR7	52.83	0.22	0.31	0	34.85	0.13	0.22	0	0.13	0
BdioOR8	13.74	0.14	0.39	0.2	13.49	0.03	0.39	1.58	0	0
BdioOR9	65.66	0.21	0.03	0.09	4.32	0.06	0.71	1.73	0.04	0.06
BdioOR10	3.43	0.03	0	0	3.59	0	0.02	0	0	0
BdioOR11	11.78	0	0	0	8.47	0.1	0.06	0	0	0
BdioOR12	25.77	0.06	0	0	29.82	0.06	0	0	0	0
BdioOR13	7.64	0.03	0.01	0	0.71	0	1.12	0.23	0.17	0
BdioOR14	9.75	0	0	0	8.59	0.06	0.05	0	0	0
BdioOR15	7.59	0.28	0	0	17.79	0.26	0.04	0.04	0	0
BdioOR16	5.16	0.57	0	0	1.76	0.21	0	0	0	0
BdioOR17	48.4	22.11	7.14	22.67	29.9	27.34	23.22	28.44	55.63	42.8
BdioOR18	2.55	0	0	0	0.98	0	0	0	0	0
BdioOR19	32.88	0.09	0	0	23.01	0.13	0.04	0.04	0	0
BdioOR20	15.73	0.93	0.5	3.35	9.11	0.72	0.9	1.26	0.95	1.22
BdioOR22	8.03	0.1	0	0.05	1.07	0	0	0	0.05	0
BdioOR23	8.95	0	0	0	15.92	0.05	0.09	0	0.05	0

BdioOR24	88.5	2.02	0.04	0.1	26.15	0.16	0.58	0.43	0.55	0.56
BdioOR25	36.89	0.06	0	0	21.87	0	0	0	0.15	0.33
BdioOR26	2.74	0	0	0	0.73	0.46	0	0	0	0
BdioOR27	3.5	0.06	0	0	0.11	0	1.83	0	0.06	0.12
BdioOR28	28.14	0.87	0.15	0.66	12.98	0.43	1.74	1.08	0.82	0.42
BdioOR29	6.83	0.21	0.29	0	0.66	0.12	0.11	1.18	0.31	0.07
BdioOR30	1.03	0	0.27	0.06	11.1	0	0.07	0.1	0	0
BdioOR31	0.46	0	0.1	0	12.26	0.13	0	0.06	0	0
BdioOR32	7.71	1.08	0.1	0	6.98	0.43	0	0.05	0	0.17
BdioOR33	58.66	16.66	14.3	21.77	43.62	17.72	41.15	53.11	19.32	14.81
BdioOR34	26.44	1.01	1.28	4.64	18.99	0.67	0.58	0.57	0.76	0.22
BdioOR35	0.36	0.09	0.04	0.18	8.82	0.25	0.61	1.13	0.04	0
BdioOR36	18.7	1.72	0.59	4.7	6	0.85	2.41	2.64	2.25	1.55
BdioOR37	1.38	0	0	0	1.83	0	0	0	0	0
BdioOR38	2.17	0	0	0.15	0.83	0	0	0	0	0
BdioOR39	25.69	3.35	30.62	411.79	16.7	13.85	2.71	2.91	13.14	0.44
BdioOR40	12.84	0	0	0	3.8	0	0	0	0	0
BdioOR41	8.96	0.1	0	0.29	24.01	0.32	0.86	0.89	0.18	0
BdioOR42	21.3	5.66	0.08	0.74	32.28	4.79	4.78	6.17	1.04	0.29
BdioOR43	37.78	0.45	0	0	13.67	0.04	0.04	0.03	0	0
BdioOR44	16.32	0.33	0.04	0	4.9	0.05	0.33	0.35	0.95	0.15
BdioOR45	7.98	0.81	0.52	1.87	9.54	2.47	6.84	1.59	1.37	0.72
BdioOR46	1.57	0	0	0	1.04	0	0	0.05	0.14	0
BdioOR47	8.6	0.13	0.22	0.1	5.32	0.53	0.31	0.15	0.06	0
BdioOR48	26.27	0.59	0.14	0.38	8.53	1.63	2.53	1.64	0.76	0.36
BdioOR49	1.5	0.1	0	0	1.39	0.29	0.05	0	0	0.1
BdioOR50	27.97	0.48	0.27	0.71	9.61	0.36	0.44	0.47	1.43	1.18
BdioOR51	2.56	0.04	0.08	0.03	5.42	0.07	9.34	10.47	0.14	0.05
BdioOR52	11.99	0.62	0	0.51	7.93	0.09	0.07	0.68	0.48	0.54

BdioOR53	58.02	0.32	0.21	0.08	27.2	0.09	0.29	0	0.04	0
BdioOR54	1.99	0	0	0	2.14	0	0	0	0.29	0.1
BdioOR55	10.51	0.03	0	0.03	11.87	0	0	0.05	0	0
BdioOR56	10.91	1.11	0.04	1.46	14.16	1.17	0.74	1.39	1.55	0.22
BdioOR57	25.32	2.15	0.47	0.58	18.88	1.02	2.46	2.61	2.4	0.99
BdioOR58	1.47	0.55	0.24	0	0.06	0.41	0.65	0.47	0.94	0.06
BdioOR59	51.64	0.64	0.16	0.12	44.91	0.24	0	0.16	0.38	0.52
BdioOR60	1.46	0	0	0	6.69	0.05	0	0.05	0.16	0
BdioOR61	6.69	12.44	4.54	11.69	3.58	11.16	21.24	10.95	3.11	30.21
BdioOR62	5.04	1.55	1.06	0	2.24	1.91	0.91	0.62	2.64	2.82
BdioOR63	14.74	0	0.09	0	1.94	0	0	0.6	0.18	0.81
BdioOR64	174.25	0.82	0.07	0.01	64.88	0.16	0.02	0.06	0.08	0
BdioOR65	17.98	0.29	0	0.7	15.17	0.22	0	0	0.08	0.13
BdioOR66	0	0	0	0	0	0.3	0.07	0.14	0.53	0.92
BdioOR67	17.85	0.11	0	0	8.28	0	0	0	0	0
BdioOR68	10.58	0	0	0	13.78	0	0	0	0.12	0
BdioOR69	49.99	1.44	16.16	2.42	32.3	0.59	1.08	1.83	3.58	0.89
BdioOR70	10.66	0.31	0	0.09	8.77	0	0	0	0	0
BdioOR71	7.45	0.19	0	0	0.18	0	0.5	1.38	0.04	0
BdioOR72	5.25	0	0.48	0	1.88	0	0	0	0	0
BdioOR73	3.61	0.35	0.04	0	5.34	1.05	0.24	0.08	0.79	0.23
BdioOR74	0	0.6	0	0.4	6.71	0	0	0	0.04	0
BdioOR75	2.61	4.12	1.78	0.59	0.87	4.19	2.99	0.47	2.87	4.12
BdioOR76	0.99	0.06	0	0	0.22	0	0	0	0	0
BdioOR77	16.57	0.27	0.05	0.47	9.91	0.58	0.09	0.2	0.55	0.07
BdioOR78	4.82	0.49	0.47	1.39	22.03	1.11	1.56	0.56	0.64	0.37
BdioOR79	28.71	9.28	13.99	4.84	12.64	8.38	5.74	6.19	5.5	3.24
BdioOR80	21.29	7.89	0.46	0.59	9.41	6.12	4.11	4.52	1.45	2.99
BdioOR81	1.71	0	0	0.03	0.61	0	0	0	0.05	0.05

BdioOR82	20.64	0.15	8.85	0.09	4.87	0.06	0.55	0.71	0.27	0.21
BdioOR83	15.02	0	1.38	0	0.7	0	0.63	0.28	0.19	0.06
BdioOR84	11.32	0.05	0.04	0.18	8.45	0.19	0.58	0.56	0.27	0.76
BdioOR85	24.35	18.28	58.66	4.71	16.12	17.48	8.08	5.26	10.46	3.06
BdioOR86	23.6	0	0.07	0	12.96	0	0	0.6	0	0
BdioOR87	9.09	0	0	0	4.84	0	0	0	0	0
BdioOR88	111.38	107.98	64.9	151.63	179.29	125.28	180.66	162.26	134.92	126.28
BdioOR89	47.64	0.51	0.35	0.45	26.5	0.92	0.54	0.37	0.72	0.42
BdioOR90	3.99	0	0.33	0	4.58	0.19	0.42	0.22	0.05	0
BdioOR91	10.59	14.61	15.02	4.5	10.03	17.11	7.95	8.76	9.52	7.59
BdioOR92	37.02	1.88	0.08	0	24.09	1.26	0.31	0.08	0.03	0.42
BdioOR93	0.09	0.31	0.09	0	0	0.5	0	0	0.09	0
BdioOR94	0	0.07	0	0	106.73	0.19	0.37	0.39	0.2	0.09
BdioOR95	0.75	0	0.11	0.06	1.5	0.23	0.69	0.18	0.11	0.05
BdioOR96	11.66	1.14	0.28	0.3	17.58	0.26	0.33	1.35	0.44	0.74
BdioOR97	15.28	0.34	0.56	0.25	8.22	0.36	0.34	0.33	0.22	0.28
BdioOR98	22.41	0.79	0.14	0	0.09	0.07	0.47	0.54	0.9	0
BdioOR99	18.19	0.35	0.05	0	13.37	0.75	0	0.33	0.52	0.34
BdioOR100	1.53	0.3	0.27	0	1.09	0	0	0	0	0
BdioOR101	5.79	0	0.15	0	10.22	0.95	0	0.68	0	0
BdioOR102	9.7	0.29	0.1	0.1	9.72	0	0	0	0.2	0
BdioOR103	4.63	2.45	0.18	0.99	0.53	1.98	2.07	6.01	0.98	0.55
BdioOR104	70.87	0.97	0.02	0.36	39.33	0.22	1.06	3.72	0.21	0
BdioOR105	0.17	0.27	0	0	0.42	0	0	0	0.14	0
BdioOR106	0.07	0.25	0	0.48	0.07	0	0.12	0.65	0	0.22
BdioOR107	1.9	0.35	0.38	1.17	1.35	0.48	0.79	1.34	0.82	1.49
BdioOR108	1.8	0	0	0	0.6	0.13	0	0	0.15	0
BdioOR109	5	0.41	0	0	0	0	0	0	0.18	0
BdioOR110	31.52	0.08	0.07	0.37	0.88	0.22	0.17	0	0	0.24

BdioOR111	12.92	0.45	0	0	0.31	0	0	0	0	0
BdioOR112	0.82	0	0.36	0	0.19	0	1.25	0	0	0
BdioOR113	96.61	0.27	0.03	0.09	53.87	0.14	0.33	0.09	0.09	0.14
BdioOR114	3.26	0.08	0.04	0	5.71	0	0.07	0.1	0	0
BdioOR115	22.3	0.13	0	0	1.5	0	0	0	0	0
BdioOR116	112.19	0.52	0	0.04	83.46	0.16	0	0.06	0.3	0.13
BdioOR117	9.05	0.24	0	0	4.57	0	0	0	0	0
BdioOR118	0.29	0	0.2	0.2	0.49	0.1	0	0	0.1	0
BdioOR119	4.85	0	0.23	0.18	0.46	0.1	0.36	0.2	0.22	0
BdioOR120	2.14	0	0	0	0.67	0	0	0	0	0
BdioOR121	3.05	0	0	0	2.99	0.03	0	0	0	0
BdioOR122	10.81	0	0.14	0.05	15.05	0.17	0.37	0.35	0.32	0.15
BdioOR123	20.37	0.08	0	0	4.29	0.09	0	0	0	0.09
BdioOR124	38.34	2.81	0	0.06	38.56	0.53	3.94	6.64	0.31	0
BdioOR125	27.46	0.29	0	0.38	16.4	0.06	0.26	0	0.17	0
BdioOR126	0.77	0	0	0	0.13	0	0	0	0	0
BdioOR127	1.15	0.14	0.07	0.11	0.32	0.06	0.59	0.5	0.18	0.03
BdioOR128	22.41	1.52	0.16	0	22.06	1.54	1.49	0	0	0.14
BdioOR129	45.07	0.34	0	0	16.12	0	0	0.39	0.17	0
BdioOR130	1.63	0	0	0	0.73	0	0	0	0	0
BdioOR131	2.24	0	0	0.72	1.01	0.2	0.18	0	0	0.12
BdioOR132	11.38	0.29	0.07	0	4.11	0	0	0	0	0.04
BdioOR133	1.63	0	0	0	0.76	0	0	0	0	0
BdioOR134	3.81	0.45	0	0	2.96	0	0	0	0	0
BdioOR135	0	0.16	0	0.19	0	0.05	0.05	0	0.35	0.98
BdioOR136	1.36	0.68	0.04	0.8	21.07	0.73	0.26	0.3	0.23	0
BdioOR137	1.37	0	0	0	0.84	0.07	0	0.2	0.83	69.88
BdioOR138	0.92	0	0	0	0	0	0	0	0	0
BdioOR139	0	0	0.42	0	0	0.66	0	0.15	0.97	0.92

BdioOR140	7.68	0.39	0	0	0	0	0.38	0	0	0
BdioOR141	4.74	0.16	0	0	0.52	0	0	0	0.05	0
BdioOR142	2.13	0	0	0	0.36	0	0	0	0	0
BdioOR143	25.51	0.15	0.14	0.14	42.92	0.22	0	0	0	0.08
BdioOR144	3.59	0.02	0.31	0.05	2.68	0	0.44	0.61	0.11	0.02
BdioOR145	1.32	0.69	0	0.17	0.79	1.57	0.69	0.4	0	0.23
BdioOR146	136.52	0.61	0.14	0.44	45.34	0.3	0	0.04	0	0
BdioOR147	0.56	0.2	0	0	0.37	0	0	0.09	0	0
BdioOR148	0.34	0	0	0	0.34	0	0	0.49	0	0
BdioOR149	2.17	0.04	0	0	0.38	0.33	0.37	0.15	0.15	0.02
BdioOR150	10.31	0.41	0	0.16	2.78	0	0.03	0.11	0.03	0
BdioOR151	0.87	0	0.11	0	0.55	0.26	0.11	0.11	0	0.32
BdioOR152	4.21	0.07	0.07	0.1	1.79	0.07	0	0.13	0.47	0.27
BdioOR153	12.04	0.4	0.02	0.19	2.37	0.12	0.51	0.19	0.09	0.11
BdioOR154	61.84	1.26	0	0.16	12.21	0.87	0	0.15	0	0.37
BdioGR1	0	0.31	0	0	0	0.07	0.07	0.14	0.22	0.14
BdioGR2	1.53	0.13	0	0.18	0.34	0.13	0.57	0.3	0.26	0.15
BdioGR3	1.26	0	0.14	0.5	0.15	0.18	1.15	0.62	0	0.07
BdioGR4	0	0.53	0.2	0.07	0	0.52	0	0	0.07	0.07
BdioGR5	0	0	0.13	0.13	0.66	0.72	0.2	0.57	0	0.63
BdioGR6	0.78	1.82	0.27	0.5	0.46	1.8	0.89	1.28	1.32	2.5
BdioGR7	2.36	0	0	0	0.75	0.03	0	0.03	0	0.03
BdioGR8	1.13	0	0	0	0.25	0	0	0	0	0
BdioGR9	2.15	0	0	0	1.28	0.24	0.64	0.69	0.95	0
BdioGR10	0.37	0	0	0	0.37	0.07	0.06	0.18	0	0.68
BdioGR11	0.44	0.1	0	0.06	0.2	0.03	0	0	0.07	0.65
BdioGR12	0.36	0	0	0.06	0.3	0	0.12	0	0.13	0.24
BdioGR13	2.83	1.28	1.11	5.47	1.81	1.88	7.91	8.21	7.39	21.36
BdioGR14	0.45	0	0	0.11	0.22	0.24	0.9	1.75	0.48	0.29

BdioGR15	0.88	0	0	0	0	0	0.59	0	0	0
BdioGR16	21.45	12.05	3.3	23.37	19.95	21.03	11.36	20.7	41.22	45.92
BdioGR17	0	0	0	0	0.89	0	0	0	0	0
BdioGR18	0.48	0.64	0.14	0.94	0.09	0.7	1.17	0.38	0.31	1.34
BdioGR19	0	0.7	0	0	0.15	0.78	0.51	0.05	0.21	0.4
BdioGR20	0	0.74	0	0	0	0	0	0	0	0
BdioGR21	0	0.07	0	0.19	0	0	0	0	2.19	1.05
BdioGR22	0	0.85	0	0	0	0	0	0.69	0	0
BdioGR23	0	0	0	0	0	0	0	0	0.42	0
BdioGR24	0.52	1.58	0.23	0.3	0.07	1.01	0.07	0.18	0.25	0.15
BdioGR25	0.15	0.35	0.09	0.45	0.15	0.89	0.04	0.07	0.24	0.4
BdioGR26	0	0	0	0	0.27	0	0	0	0	0.81
BdioGR27	0	0	0	0	0	0.24	0	0	0	0
BdioGR28	67.05	90.85	59.34	48.85	100.05	85.42	85.45	59.69	43.62	50.65
BdioGR29	1.31	2.11	2.97	0.48	1.85	2.56	1.25	1.21	2.55	5.43
BdioGR30	0.4	0.58	0	0	0.07	0	0.54	0	0	0
BdioGR31	0	0	0	0	0	0	0.12	0	0	0.23
BdioGR32	0.08	0	0.88	0.41	0.04	0	0.67	0.25	0.36	0.18
BdioGR33	1.07	0.52	0.14	0.22	0.44	0.27	0.04	0.33	0.04	0.77
BdioGR34	0	0	0	0	0.44	0	0	0	0	0
BdioGR35	0.43	0.84	0.45	1.92	0.61	0.52	1.3	1.74	0.33	2.43
BdioGR36	0.5	0	0	0	0.17	0.18	0	0.59	0	0.91
BdioGR37	3.41	0.81	0.44	0.66	2.22	0.75	0.08	1.64	0.24	4.72
BdioGR38	0.17	0	0.46	0.08	0	0.17	4.1	3.44	0.26	0
BdioGR39	5.28	15.88	3.18	3.09	2.29	16.23	3.41	5.62	3.64	4.01
BdioGR40	11.34	6.56	3.42	21.19	9.17	8.72	18.72	29.39	6.47	24.94
BdioGR41	12.33	31.11	1.57	2.97	8	40.52	4.38	2.21	5.17	4.11
BdioGR42	0.19	0.33	0	0.28	0	0.44	0.48	0.8	1.89	0.87
BdioGR43	0.17	1.16	0.08	1.14	0	0.39	0	0.55	0.6	0.19

BdioGR43a1	2.85	6.18	0.12	1.25	1.79	6.56	0.96	2.37	2.15	5.11
BdioGR43a2	0	0	0	0	0	1.29	0	0	0	0
BdioGR43a3	0.22	0	0	0	0	0	0	0	0	0
BdioGR43a4	0	0	0	0	0	1.09	0	0	0	0
BdioGR64f	28.04	14.03	0.65	7.91	30.36	19.3	3.19	6.77	7.85	9.64
BdioGR28b	0.7	0.05	0.04	0.17	0.95	0	0.27	0	0	0.13
BdioIR21a.1	1.58	0.05	0	0.05	3.34	0	0.26	0.34	0	0
BdioIR21a.2	0.43	0.53	0.14	0.23	1.03	0.6	0.6	0.42	0.13	0.27
BdioIR25a	1.54	0.86	0.09	0.68	2.51	1.5	0.05	0.22	0.21	0.67
BdioIR25a.2	0	0	0.3	0.28	0	0.33	0.79	0.34	0.04	0.03
BdioIR75a.1	9.84	0.21	1.86	0.18	10.03	0.62	1.21	0.71	2.98	0.59
BdioIR75a.10	4.58	0	0	0	2.43	0	0	0	0	0
BdioIR75a.2	3.11	0.03	0.62	0.06	2.71	0.02	0	0	0.35	0.34
BdioIR75a.3	1.75	0	0.15	0.16	3.1	0.13	0.23	0.32	0.15	0.03
BdioIR75a.4	2.74	0	0	0	2.44	0	0.08	0	0	0
BdioIR75a.5	6.54	0	0	0.1	5.47	0.05	0.42	0.2	0.05	0
BdioIR75a.6	8.57	0	0.03	0	4.24	0	0	0	0	0
BdioIR75a.7	8.25	0.1	0	0	7.82	0.07	0	0	0	0
BdioIR75a.8	2.6	0.06	0	0	1.69	0	0	0	0	0
BdioIR75a.9	4.4	0.34	5.16	246.63	6.22	6.82	0.08	0.49	0.17	7.57
BdioIR75b	1.4	0	0	0	0.76	0	0.29	0	0.1	0
BdioIR75d	1.07	0	0	0	1.45	0	0	0	0	0
BdioIR75e	7.37	0	0	0	7.77	0	0	0	0	0
BdioIR75u	16.16	0.17	0	0	24.82	0.18	0.03	0	0	0.07
BdioIR8a	79.49	13.7	0.99	5.52	58.42	6.55	4.27	3.31	12.54	8.31
BdioIR93a	3.36	0.07	0.02	0.19	10.51	0.52	0.5	0.66	0.28	0.09
BdioIR75c	2.48	0.09	0.12	0.43	2.26	0.39	0.81	0.79	0.21	0.37
BdioIR1	0	0	0	0	0	0	0	0	0	0.31
BdioIR2	3.12	0	0	0	0.36	0	0	0	0	0

BdioSNMP1a	286.5	6.16	50.39	10.41	331.2	4.3	15.98	15.62	3.35	9.64
BdioSNMP1b	536.53	14.55	0.22	24.3	820.85	16.73	0.31	1.32	0.21	0.79
BdioSNMP1c	394.33	2.17	0.02	0.08	331.87	0.77	0.32	0.2	0	0.12
BdioSNMP1d	73.18	35.44	6.75	24.64	174.02	47.43	28.67	30.37	58.17	63.83
BdioSNMP1e	59.98	38.94	28.54	55.08	51.99	56.76	56	64.04	62.97	126.49
BdioSNMP1f	1.16	0	0	0.7	10.78	0.15	0.07	0.27	1.09	1.25
BdioSNMP1g	0	0	0	0	0.13	0	1.19	1.18	0.05	0
BdioSNMP1h	37.92	0.34	0.12	0.07	74.49	0.27	113.27	153.48	0	3.4

Table S11. Primers of *B. dioryctriae* candidate chemosensory genes and *GAPDH* used for RT-qPCR.

Gene Name	Primers (5'-3')
BdioCSP10-F	GTTGTCAGACACTACACATTCC
BdioCSP10-R	AGACCATTATCCTCTCGCTTG
BdioCSP8-F	GGTCGTACATCCGTACAGTTTG
BdioCSP8-R	ACAGGCTTCTTCGTTGGTTTG
BdioIR75a.9-F	CGCCGAGTTAGTACGAGTTG
BdioIR75a.9-R	GAAGCACTGAGAACATCTTGAC
BdioNPC2a-F	TTGATCCTCTTGGCTCTTGTTG
BdioNPC2a-R	GTGGCTTGTCGCAGTTCTC
BdioOBP10-F	ACGGAGGGTAAAGTATCAATCG
BdioOBP10-R	GACGCATTCGGTGATTTTATCC
BdioOBP12-F	TGGTTGATGGTAAAGTCGTTGC
BdioOBP12-R	CGGCTACTTCGCATTCGTC
BdioOBP14-F	CGTCGCATTTGTACCTTTAGC
BdioOBP14-R	GGCAACGTTACCTAACTTCAAC
BdioOBP15-F	GGGCGATATCAGTGACTCTTC
BdioOBP15-R	GTACTCGTACTCGTTCAATGTG
BdioOBP22-F	AAGCCCGAACCACCAACG

BdioOBP22-R	CCTTGCCGACGATGAAACAG
BdioOBP3-F	AGAGTTAGTCAGCTCACAATCG
BdioOBP3-R	ATCAACCGATACCTTGCCATC
BdioOBP8-F	CACGGACGAGCAGAAAGC
BdioOBP8-R	CGTAGACGACAGGTTCACC
BdioOR110-F	CTCCTGAATCCAAGACAAGTTG
BdioOR110-R	CAGCGGAATATCGGTTGTAATC
BdioOR111-F	GGTTATATGTCGGCGGCAAG
BdioOR111-R	CGAGTCTGTGGCGAAAGTATC
BdioOR115-F	CCTTCGTGGGCTTATTCATGTG
BdioOR115-R	CGTACCATTCGCACTCGTATG
BdioOR123-F	CGGAACGAACTGAAGGAATGTG
BdioOR123-R	GATGACGGCGGTGGTGAC
BdioOR140-F	GCATGGTTGTAGCGGGATC
BdioOR140-R	TCCGATGTAGATTGCTGATGTG
BdioOR141-F	CCACGGTTCTTCATAGACTTCC
BdioOR141-R	GGACCTTCATGCTCATCAACTC
BdioOR146-F	AGAAGTTCAGCGAAAGCAAGAG
BdioOR146-R	GCACGAGTTACACCGAAGTTC
BdioOR149-F	ACTGCAAGCGTTCCCAAC
BdioOR149-R	ACTTTCTGTCGGAGACTTCATC
BdioOR14-F	ACGCATCTTCGGTCCCTTC
BdioOR14-R	GACAGCCAATCCAAGCACTC
BdioOR153-F	AAAATACGTTGCTTGCTTTACG
BdioOR153-R	GCCAGCCATAACTTGTAACAG
BdioOR154-F	TGTGTAGGATTGTTCGCTGTG
BdioOR154-R	CAATTCCACGGCTTTGACTAAC
BdioOR22-F	GCAGGCATGAGCGATTGG
BdioOR22-R	TTGGCGTCCGTTGTGTATC

BdioOR24-F	TAGCCGCATTCCTATATCTGTG
BdioOR24-R	TCTTCGCAGTTCTAGCCAATC
BdioOR27-F	CGAACGACTGTGTTTGCTATTC
BdioOR27-R	GCGAAGAAGGTGGAGATGATG
BdioOR29-F	CTCGGTAGATGTAATCGCTCTG
BdioOR29-R	TATGCCATTCTGACTCTGATCG
BdioOR39-F	CGACAGTAGCGAACAGGTATTC
BdioOR39-R	ACAATTTGCCAGCCGTCAAC
BdioOR63-F	AGAGTGGCAGAGGAATTGAAC
BdioOR63-R	CACGAGGAACTTGAAGATGTTG
BdioOR6-F	AGCACTTCTCGCCTGTACC
BdioOR6-R	CGTCGTCAGCCTAATTCTACC
BdioOR71-F	TCGTAGGCAGCATCCAAGAG
BdioOR71-R	TGCGAACCTGAATAATCCACTG
BdioOR98-F	AGCGTGAAATGCGTATCTCTG
BdioOR98-R	ATGTCAACTGCGGCTATAACTG
BdioOR9-F	AAGAGCGAGTTCTCAGTCAAC
BdioOR9-R	CGTATAGTGCCGTGCCTTG
BdioGAPDH-F	CCCATGTACGTGTGTGGTGT
BdioGAPDH-R	TCTCGAAGTTGTCGTGGATG

Supplementary Data S1. The amino acid sequences of 233 odorant binding proteins (OBPs) from 8 Hymenoptera species.

>BdioOBP1

MKIQVTIISVVVVCFALVNRTYAGITRAQMEGLAKGFRSTCVRKVHADIALVAGLRDGQFVEGNYPLKCYTKCIMSLMKTLKNNKADVSAISKQIKAMASDDVADRLVD
AAQKCYTEINDKDICEYTWRYTICTYKADPDAFFFP

>BdioOBP2

MNAVAIVFAVCLAGVSAGMIERAQLLPEPNITKECLKEYGIDYDRTTETPLTDEEIYCIPACAYRDYGIMRPDGTINTEKAESYFGPEDGEERDIFFSVYEACREGKTHCKLV
QCMFGQLKNHWKISTESNKITKQLFSQLA

>BdioOBP3

MAQNIVGYQRNIDEMKTYLYVVAVLFSIIALVQCDDDDSLVSKVQNCAEQFSLTKAEFIELVSSQSLAFDNSNFRCVLACLLKKDDRLKDGKVSVDKEMDALEETSPDET
EESLREKVTDCANKGKYANFYFLNFLQTFVQ
>BdioOBP4
MNTLTLFLLLCAIVAMGVQANIPKSRFNPELKNVWDKCTKEAKLTEAELDIVLQNYSKITDQRVKCLKGCLFKSLHVINAENQIDEAAAQKYYEGVGTKKDTINEAIQKCI
PKATGNDQCDIAQSFESCLCSEYIKCS
>BdioOBP5
MKNVALCLLVLVFGAVKIHAGEVPKEIAHMVADVREKCHRETGVDIEHVDRTVEGYFHPSETLGCYFSCIFGHFDLLDHNGHIDFDKIISKIPDSFKEHGMEMINACRHLT
GNNPCDMALNVVQCFQKTNPEKYFVI
>BdioOBP6
MNSYLLLLILGVVYAKNAQAASSVTQALLEIKASVDKCGKEAGFNEEQLNNALKNYEKNADHKVKCFRGCLLKAHKVITDEEKIDENAAIKYYHGDKLEPLKVVIQKCV
ADSVIGGDHCDLAHNLESCMCSSLTTC
>BdioOBP7
MKIIFVLLVFCLAVVLAWPDEDEDELTESAEADEEIKASAFKDCWKKKKLTEKQMEDIKNWGCIIACVHTKIGFINADGSLNVERITQRNPGIREEIQEIYQTCVKYAKGDP
CQLSLCLEGQNFDKNVSYDDSSY
>BdioOBP8
MKSFVVVFAFCIVGALAGLTDEQKAKLKEHRVACVAETGVSDDVIEKAKKGEPVVYDEKLNCFAACMLKKAGIMKADGTIDEAVARAKLPKDIPQEKVDQVINMCKTQ
VGKDACETGGKVMSCLAKTKAVTLLN
>BdioOBP9
MKLILSTFCVIFVVVQNAQGNDESMKSLEECLDENDIKDDFTKLKEHDDPKLRCIMACVMEKEGILKNGEVKLDKLKKDLLEDAEQLGEKKVSEIVNYCGNLAKDLMDV
CDKSNLIGLCIKEEFAKYNINFEK
>BdioOBP10
MKVYLCIASLLSALVLIQCNKIPPELKNKVDGCLKEFELTEESLKTPDFEDSKIKCFGACFLKSDDRMTEGKVSIDKEMDAATKYLEKDKIDDTLKDKITECVNKANEEKDD
CAVAGTLYKCVYEKFGPLEV
>BdioOBP11
MRTTQEISTSAPNHERVDDVDLNDSIDQCAGLIGLLVEETRKSFTMPLEAPGNCVMACVWDKIGMMDIDGKIIKEEMVSSIHPTLQLLPNITRVTEDDFYACVDEANQFSDP
CAVVSEYFKCLIKDLFMHNT
>BdioOBP12
MKIYLCIAGLFCALVLVKSEPPAEVKKIFEECEKEIGVTKESIRGPQHADDPKAKCFYACIMKKDGTMVDGKVVAEKAIEVEANFKPDGANDSINEEFTECANKANEQKDE
CEVAGAFFKCLKKHDHHGKRE

>BdioOBP13
MKIHFYLAGILSAFMIVKCEMDPEMKEKFDECREEFGISEESLESPDFEDPKVKCFAACFMQKDDRLTHGKLSIDKELKAAEKYIKKEKIDDSFKDKITECVKKANEEKDDC
GVSGALFKCIDDTFGPFTP
>BdioOBP14
MATLPNFNATFWFVVFSIFSVVILIDCKPNFYEQCLRESAVAKGTNATQIFINIDDPKLSCLNACIFKKFSMMKNGKFDAEITFNFLKKSYPKPLSNLRERINDCVLKANEFND
ECEAGNTMKKCLTTNS
>BdioOBP15
MKSSLCIAGILYVLVLVQCEIVSEFKSILDECQKEFQLSEESLISPNFEDNKINCFAACVMKKDDRLTEGKISVDKEVATAPKYMADRIDDSLKDKMTECVDKANEEKDDCA
VSGTFYQCLIEKFGPFHP
>BdioOBP16
MKAFAVVLAVVLAVACADDPLKDIPKDLIKTCLTENGFDAAQYPNGLRNVKIPDNQEKNRDCYYACMMKKMNLMKPDGMLMEDNLKTKFNMNLETLQKAMNTCKTQ
VKGNDSCKLAACLMANRGI
>BdioOBP17
MKILLAVLLFCIVVAYCDRTMFASNRIMEDCAQRHKIDKLILFELKRNRTFGATTDTQCFAGCVLKQVGIMSDDGTVDQDFIDSPMADRCKNWFFAFGKDQCEIAAKILGC
LSQKHVIVFSDD
>BdioOBP18
MKLFVVVALCTVVTIVNGLTDEQKAKLREDRAACVAETGVSDDVVTKLKQGENVVFDEKLNCFSACMLKKIGVMRPDGSIDEQAARAKVPKEVPQDKVDLVINMCKAL
GIMKIIISKIEVNE
>BdioOBP19
MKLIFILIACCLAVTVAWSGKDEDRVREKAMEECRQEKKLTVDEAMNPSNLGCMYACMHKKIGYLNADDTLNIERVTHGNPNTRFREVVRGVYQECVKNGKGDACQVS
LCLENTKVA
>BdioOBP20
MEKMSEGFRRTCVSKTGADMNLIAGIRNGNFIDDPAARCYTKCIMALMKTLTKQGQIDADMMVKQINIMASPDIAPSMVAGVRKCYSEVTASEPCDLAWLFTKCIHDAD
PSTFFFP
>BdioOBP21
MKFMWAVFICCLAVTLADFEEDEFEERRKALQECREEMKLIRDNELASNEGCLYACVHKKLKFFNPDGTLNIDVAIHDGLEPDLQEKVQTAYRACVENAKGDACKVSFC
LENLEI
>BdioOBP22
MINKKLSTDAVTAIIKGGEIKRDEKLDCFSACMLKKIGVMRADGTIDVDASREKARTTNVDVAKADKIIDKCKDLIGKNTCETGGNVFGCFIVGKDFPVLD

>BdioOBP23
MTALIEKAKKQFKEEGKVELDENMSCFSACMFKKIGFMDEEGKLDEEMVRALTADQFPEDQLDAAIGACKDEAGKNDCETAGKLVACFMKQGIAVNS
>BdioOBP24
MGVTKESLKLLKDQNDPNTWCIFACAMEKKGLMVNGKAVTEKVIEDISQDMPEKVKEVIRANAPACVEEANKENNECAVFMVLRNCLAEKNLSRK
>BdioOBP25
MQLPSLVLFLVLPLLLLAHAKAGTRPSFVSDKMIATAASVVNACQTQTGVTTGKSSSYLPLAFSFFVLSLAPARAPFLPSFQL
>AmelOBP1
MASNTKQAFIYSLALLCLHAIFVNAAPDWVPPEVFDLVAEDKARCMSEHGTTQAQIDDVDKGNLVNEPSITCYMYCLLEAFSLVDDEANVDEDIMLGLLPDQLQERAQSV
MGKCLPTSGSDNCNKIYNLAKCVQESAPDVWFVI
>AmelOBP2
MNTLVTVTCLLAALTVVRGIDQDTVVAKYMEYLMPDIMPCADELHISEDIATNIQAAKNGADMSQLGCLKACVMKRIEMLKGTELYVEPVYKMIEVVHAGNADDIQLV
KGIANECIENAKGETDECNIGNKYTDCYIEKLFS
>AmelOBP3
MKTIVILLFTLCIVSYMMVRCDDITLCLKQENLNLDDIDSLLEDESERMLRKRGCIEACLFHRLALMNDNVFDVSKFDVYLNDTDMDMDLKDSIRKIIRQCVDNAKNEDKC
LTAQKFSRCVIDYVKFHITQYMISNANSNTTSEEESSDNST
>AmelOBP4
MKITIVSLLCVIYCALVHADTVAILCSQKAGFDLSDLKSMYESNSEEQMKKLGCFEACVFQKLHFMDGNTLNVEKLESGTRELTPDDFTEDVHEIIEQCVSKAADEDECMV
ARKYIDCALEKMKFLDDELEKIAGN
>AmelOBP5
MHVKSVLLLITIVTFVALKPVKSMSADQVEKLAKNMRKSCLQKIAITEELVDGMRRGEFPDDHDLQCYTTCIMKLLRTFKNGNFDFDMIVKQLEITMPPEEVVIGKEIVAV
CRNEEYTGDDCQKTYQYVQCHYKQNPEKFFFP
>AmelOBP6
MKGLGVSLLVALLLVLLAIEDTMSKKMTIEEAKKTIKNLRKVCSKKNDTPKELLDGQFRGEFPQDERLMCYMKCIMIATKAMKNDVILWDFFVKNARMILLEEYIPRVES
VVETCKKEVTSTEGCEVAWQFGKCIYENDKELYLAP
>AmelOBP7
MKKFLVIFVYILSVAVIIRANGINEILKIMAVSMKDIRYCIIHMGLTFKDFIKMQELLQEEDISEGNIKKYLTNYSCFITCALEKSHIIQNDEIQLDKLVEMANRKNISIDVKMLS
ECINANKSTDKCENGLNFIICFSKLLSDMYEDTFEDTLKHKSYV
>AmelOBP8
MTIEELKKTIKNLRKVCSKKNDTPKELLDGQFRGEFPQDERLMCYMKCIMIATKAMKNDVILWDFFVKNARMILLEEYIPRVESVVETCKKEVTSTEGCEVAWQFGKCIY

ENDKELYLAP
>AmelOBP9
MFKNYHFFFILVITLIFLYFGEADIKKDCRKESKVSWAALKKMKAGDMEQDDQNLKCYLKCFMTKHGILDKNAEVDVQKALRHLPRSMQDSTKKLFNKCKSIQNEDPCE
KAYQLVKCYVEFHPEVLQTVPFL
>AmelOBP10
MKYSILLSLLITCLICSPSVHCGTRPSFVSDEMIATAASVVNACQTQTGVATVDIEAVRNGQWPETRQLKCYMYCLWEQFGLVDDKRELSLNGMLTFFQRIPAYRAEVQKA
ISECKGIAKGDNCEYAYRFNKCYAELSPRTYYLF
>AmelOBP11
MKAAEIWLVSLYWYLILQIALVYGEISDIDEFREMTSKYRKKCIGETKTTIEDVEATEYGEFPEDEKLKCYFNCVLEKFNVMDKKNGKIRYNLLKKVIPEAFKEIGVEMIDS
CSNVDSSDKCEKSFMFMKCMYEVNPIAFIAP
>AmelOBP12
MLYNNLTIVIILIMCGVQNLRARSVNIFQDIADCVDRSNMTFHELKKLRDSSEARIKLINEEENFRNYGCFLACIWQQTGVMNGSELSTYNIAGIIEGQYHDDEDLKTFFHKI
ALTCEDDVHRKFLHVNDECDVALSFKLCMLKAMRNYP
>AmelOBP13
MKTIIFIFAFCLVGILAVSEESINKLRKIESVCAEENGIDLKKADDVKKGIFDKNDEKLACYVDCMLKKVGFVNADTTFNEEKFRERTTKLDSEQVNRLVNNCKDITESNSC
KKSSKLLQCFIDNNLMKIFE
>AmelOBP14
MKTIVLIFGFCVCVGALTIEELKTRLHTEQSVCKTETGIDQQKANDVIEGNIDVEDKKVQLYCECILKNFNILDKNNVFKPQGIKAVMELLIDENSVKQLVSDCSTISEENPH
LKASKLVQCVSKYKTMKSVDFL
>AmelOBP15
MKTILIISAICICVGALSIKDFQNAIRMGQSICMAKTGINKQIINDVNDGKINIEDENVQLYIECAMKKFSFVDKDGNFNEHVSREIAKIFLNENEINQLITECSAISDTNVHLKI
TKIFQCITKFKTINDILNS
>AmelOBP16
MKTFVIIFAICVCVGAMTHEELKTGIQTLQPICVGETGTSQKIIDEVYNGNVNVEDENVQSYVECMMKKFNVVDENGNFNEKNTRDIVQAVLDDNETDQLIVECSPISDAN
VHIKISKIFQCFMKYKTITDILNS
>AmelOBP17
MKTIVIISAICVCVSAMTLDELKSGLHTVQSVCMKEIGTAQQIIDDINEGKINMDDENVLLFIECTMKKFNVVDENANFNEKISSDIVRAVLNDNEADQLLAECSPISDPNALI
KISKILECFFKYKTINQILNS
>AmelOBP18

MKTFVIISAICVCVGALTLEEFQIGLRAVVPICRIETSIDQQKEDDFRDGNIDVEDEKVQLFSECLIKKFNGYDDGGNFNEVVIREIAEIFLDENGVNKLITECSAISDADLAVK
SAKLLKCIGKYKTLKEMLSG
>AmelOBP19
MKTIVVIFAFCICVNAMTIEELKIQLRDVQEICKAESGIDQQTVDDINEVNFDVEDEKPQRYNECILKQFNIVDESGNFKENIVQELTSIYLDENVIKKLVAECSVISDANIYIR
FNKLVKCFGKYKTMKEVLNL
>AmelOBP20
MKTIVVIFAFCICVNAMTIEELKIQLHDVQEICKTESGIDQQTVDDINEVNFDVEDEKPQRYNECILKQFNIVDESGNFKENIVQELTSIYLDENVIKKLVAECSVISDANIYIRF
NKLVKCFGKYKTMKEVLNL
>AmelOBP21
MKTIVIISAICVCVGALTLEELQIGLRAVIPVCRIDSGIDEKKEDDFRNGIIDVENEKVQLFSECLIKKFNAYDDGGNFNEVVVREIAEIYLDENEVNKLITECSAISDADIHLKS
SKLIKCFAKYKTLKEIMNE
>NvitOBP1
MMKNLTLCFLVVVLGVIKVNGNEIPHEIRHMVVGVRDKCHRETGVDIEHVDRTVEGYFHPSELLGCYFSCIFNHFDLLDKDGHLDWDKLVPRIPESFKEHADEMIAACRST
TGKDPCDSALNIVQCFQKTNPSKYFVI
>NvitOBP2
MSGQSLLLLALGIFLPHCLAGTRPSFVSDKMIATAASVVNACQMQTGVATADIESVRNG
QWPDTMELKCYMYCLWEQFGLIDEKRELSLNGMLTFFQRIPAYRVEVEKAINECKALATGDTCEYAYTFNKCYAERSPRTYYLF
>NvitOBP3
MKSLLLCFLVVILGVTKVKSNEIPQEIQAMVVGVRDKCHRETGVDIEHVDRTVEGYFHPSELLGCYFSCIFNHFNLLDNDGHLDWVKVVNVIPPSFKDHADEMIAACKTTT
GKDPCDSAVNIVQCFQKTNPAKYFVI
>NvitOBP4
MKAVAIILVVCLVQGLQALNKSETPGLNDQMKECLTQNDLDADLYTELWKDHPKLNAPQKKVNCFLACLYKKVGALSADGAIVLPEGLIEERIINWSPELREKCKKQAG
DDVCELAGCLDKPNGFLSATV
>NvitOBP5
SSAISQFGKLKDAPIPSRQYYLRHRRNRERKKGAGALRIGIEFQRDSLCYALCIAFLAADIDPTAEIEIDRSGSRAMRWIVDEDFPRERHRPTANCVTRTHNAITSCLGSFPLV
RCT
>NvitOBP6
MKFLTSVLSCFVIHAMLVRCAPFHETLDDDPDLNDSIDLCAAEVGLLVEETRKSFNMPIEAPGNCVVACVWKKIGLMELDGKIVKEEMISSLHPTLKQMPNITPIHEDDFYH
CVDEANDYEGGCIVVSEYFKCIIRDLFNQLSI

>NvitOBP7
MKAFLCTFSIVLAAAMSVNGDMPGELKPAFQECHNELLGTPQEEPTGPPNMDDPKVKCIHACVAKKIGHMVDGKIVAEKEIESAKQHMPNADNSLTDKITECANKANEQS
DECEVSAAFHKCIVEKVGPPEHHH
>NvitOBP8
MKAFLCVLGVIIAAASASCGMPEEMKQAFKECHTELGMPDEKPHGPPNPDDPKIKCFHACIMKKAGKMVDGKLDADKEIEFAKKRMPNADDSMIEKITECVKTANEQSD
ECEVAGAMHKCIMEKVGSPPHHHRH
>NvitOBP9
MKAVVIVLAVCLAGVFAEDPIKDINKEYIKGCLIENGFDPQQYPTGLRNAKVPEKQEQNRNCYYSCMMK
KMNLMKADGSLNEDALRQKFNMNLDTLGKALSTCKDQVKDDKCKLAACLMANRGA
>NvitOBP10
MDRHLIIALTLFSVVFMVQSLSQEDIDARNKCLKEHGFTIEPKYVSAYKTIDIRAKCYASCLMRETGVVKEDGSIDLNKVLEKISDSENKTLDEVVKKSFIPCTEKKGDNDC
DTGHQILTCIVATISILKESMKIV
>NvitOBP11
MSRHLIIALALFSAVFMVKSLSPEERVARDKCLKENGFSREPDFIGIDAVDMRSKCYAACALRGYGIMKEDSSIDINKILEHISDTKNKDIDVKKSLIIPCAEKKGETDCDTGY
LITNCVALAVRKLDK
>NvitOBP12
MRLLTALLLIGIVAVVNAKSGSTAFTITQNDRNVFRNCMTKIGIPDDEMVAVLDNHEKDADEKVKCYNGCLYKAFKVIKDDGTVDTEAAIKFFKVEDMESDKNIIVKCSN
ESNSNKEKNDCDTAQTMESCYYKLKKEQ
>NvitOBP13
MRLFAFANVLGIVLLIHNSATKTNVERFWDYVDVLKDCAKENGISIESYAYASKKNNTDGIYEKSKCVEACMFKSHKIMRPDGTIDMEKAIEHLLTGNPGEKRDLMKKNI
ESCEIPNGDNECEVAHTMVKCALGYD
>NvitOBP14
MKAFLCTFSIVLAAAMSVNGDMPGELKPAFQECHNELLGTPHEEPTGPPNMDDPKVKCIHACVAKKIGHMVDGKIVAEKEIESAKQHMPNADNSLTDKITECANKANEQS
DECEVSAAFHKCIVEKVGPPEHHH
>NvitOBP15
MMRILLLGFFCSIFALSHQEIPEPSYTHWQEHLQSCLDQTGLDLSIFGVSRIDDVTEQNLKKLAEVTADKRGCLVACVFQKQGMISKEGVLQANPPRPDPTTKLETTFEEAIA
ACRSEKNFCKLGNCLFEIYFKYK
>NvitOBP16
MRVILLSSILLGSIAISRQNPVTYTDKDNNVIIPPCLAETGLNLSVLGVAKIEDVRDSSFYNLKTLTEDKRGCFVACVYKKLGIITEENVLINDRVIPPGVAVPKKKLATAFED

ATEACRAQKDLCKLGNCLYEIYFF
>NvitOBP17
MKFFTVATFAMCIIGTFAAFTMTEEQAKDLQDKLDCIKETGADIATLLNIKNGIPTLYDDKVNCFAACMLEKFNIMKPDGSMDETVARLRASKSMSQEKVDRVLSSCKSEV
GKDKCETGGKILECLMKNDAVPILS
>NvitOBP18
MKSFAVIFAFCFVGAIAALTEEQKAKLKEYKYACITETGVSEDVIESVKKGEQVTFDEKLNCFSACMLKKVGIMNADGTVNEEVARAKVPQDLPKDKVDQVINTCKAEVG
KDSCETGGKVLACLMKTKAVSVLH
>NvitOBP19
MRILLLSIFGSIIAFSHQQENPELSDAHWQEDLQSCLDQTGLDLSIFGVSRIDEVTEQHLKKLTKVPADKRGCLVACVFQKQGMISKEGVLQNNPPHPDPTTKFETTFEDAIA
VCRAEENFCKLGNCLFGIYFNYEI
>NvitOBP20
MKELIVIVGFLVAAMPSPAAFQIDSSKRMNQTVTECLTGYNIDPAVLDINTEDVHLMMDELSGEQRGCVTACVYKGFDWLKDDGSLDIDALTMDEDPEDTAEFIKDIVDC
RNKVGTEACKFFHCLDTKGT
>NvitOBP21
MKTLFFVVLGLVAVSAAVPVEHSFPQFENGTPKMKEQVNTCLRNYKIDAAVLELGDEKNFEKTDKLTKLEWGCVRACVYKGANFMRADGSLDIEVLTDGDEPEDKKKF
ESVVGICRAEAGKDDCKFFQCMDEKDDS
>NvitOBP22
MKTSPAVVLALCFVNVFGNSITELKNGSSRVKVNVRQCLTDYHIDPAVLELDIDRNDDLYSKLSEEKKGCVTACVYRGFNWLRPDGSLDIDLLCEGETPEESEAERKRYTK
IVAECRAEVGKDDCKFFNCLNLKDL
>NvitOBP23
MKTVIVFFFILVGILAETTTNVDSRDDDMTTCLVEYGLDPGPNNPTEDQKNCYFACMFKTIGYMKKDGSFNLDLILSDAYRSEKRVESKRKLDNIVSMCKQRAGNDICKLA
GCYQEHRN
>NvitOBP24
MNAAIIILAFCLAGALARGIIDKNESGVEVNDACLLEYGINPDQVYNDGSDGSEAVTALTDEQIYCVAACIYKDYGIMRPNGTIDTEKADSYFGEDDSRERDIFFAVYNACS
EGRVGCKLVQCMFSELKNHWGSSTSDSKKELKPLFNRPDFIRAN
>NvitOBP25
MKKIVFIISTFCFVMIQGIRKELNPKKINLTEFTEAMQSCGTMLGFDREFKLHLFGSQEDYNKTLCLSFCALRKLKFYVIEDEIKKELAHVRNARLKEEVYKALDVCKHLLD
DPCKLFDCFFDYAKVVDAENESLNLSKFFSEGSL
>NvitOBP26

MKTFAIVLTLCIVGAYASTLKDDQKAKLREYKESCITETSADKAVIDSIIKGGPINRDEKLDCFSACMLKKIGIMRPDGSIDVESARAKAATTNVDVAKANEVIDKCKDLKG
KDTCETGGAVFGCFITNKDFPVLN
>NvitOBP27
MKKILCFIVFCLTSNVWGETSATVEPPTKLKTCANVTRITVAVNLLDKECMKTSSSSAILLNGDENNVEVKDIEMNVYALCLLQKSSIMNEQGKINLNFDIFKIVKNLYKRT
DQRGFGLAFIIKSLEKCRQTDGPDQFSTATKIMKCLLDNQKTVIRCDQH
>NvitOBP28
MKIFVIVALCAVAVYAEENEVLKQYERDCMTENGIDPTVQDPKNLTLEDGNCYYACYFKKFGIIKEDGSYDVAAIKEKYSKPNSVEAVQKKLDEITQTYCQDKVGNHCNL
AACLSKISKEQWKI
>NvitOBP29
MKVFVALALCVIAVNGEVTESSSTEFPSAVDIYKMKIFKYSMECLFERKLDLSKFALQKDVKKAVEDLHKDEKACFAGCVFKKLGAMNDDGTFNEDKLFMGATAETLPIF
KQTHDAAVKHCTDKVGKDELCKFAACIVIQAPAYASSLNATSGI
>NvitOBP30
MKLHALLVLCFATASANIRLTDQQLKEYVQVCLAKTRLSQGFYQSGDEAQKILTEEQKSCFLACMFKRTGIIDHDGSVNLKLGDEELPRTPAIEACITTAKEDICKLAICLH
KTGKFSITSVADSPRYPRYH
>NvitOBP31
MKSYILPIAICFAVIDMIFIRVLQCSFLPLEKMNKRHAYIVTGYTIVTDVELLRELMLRNDSKDEKAIDLENKFTCAVACFSDAKINVSREEIKSDLMNTLDTCHQKDEGDNC
NLLECVKVLIPPFKALLIFA
>NvitOBP32
MKSYVLAFAICFAVIDLSFALGKEDQEKCLRKNGLNNSTDVELMKAFVRSDGKNEFHLEREFSCVVACVIDERRTEDNVNTSTYQLLTDLISEAHNKIPDEQWRDMKTTL
DKCHQQDEGDDCKLLYCVKILRDPFKELIVSFD
>NvitOBP33
MKIYLFLFCFTSIDLASALTAPKEQQLACLNENGVTNSTDVELFNIIMQDVFKDEFILETSQDKQFSCVFAYIINSIIKRIPQDSIYQELTRVIQTVIISMKNNFYGFFSNGRYIIID
KLKFSYR
>NvitOBP34
MGCMCSVINANFHFRRAADDKLSDAKKREMKDTLKACSLATGDDDDCTLLQCVSILQPPFVWILPKTSGI
LGYLKNV
>NvitOBP35
MKLFFALFVLSFALLHSATGAKDSLVECLQENGLKMVDLDFMRKIKPNTDMPRNKLIEDKLACAFACSFNRDNSWKDENVFTFMTDVIKKDYRIPVGLKKQMLDTLKSC
NAEAKGDDCTLLQCIKVTRYPFMDFVFLHNTEMKPEYESSQDKQI

>NvitOBP36

MKFALPALFIISCAAIHLTAAANTGNDSMTECLQKYGLKMDDLEFLRKPNTEVQPMKNKAIEDKVACALACTFKKESQYRSIFPFLKNVLRIDRQIPVNLKKDMLDTLDDC
NGEAKGNDCKLLQCIKITRNPFMNLVFSYGA

>NvitOBP37

MQSYLFSLIVCFASMNLCLAYYYTEEQQRECLFKNGLNNSTDIELFKEFIRTDSKDQKVFPLEDQFSCAVACVFNLGKPDPSEDRIYHKLLYSIKNDDQIPGELKRYMMDKL
DHCHRQDEGDDCKLFICIKLFRPPFKETIISIE

>NvitOBP38

MKIFVTFFILCFVCKPSNGTSLVKACLVENGFGASGKDLEIVKAVANPEYVGILRQVPKDKLSGVFACIFQDRNPTTNLYASLKRLIEMDDKVSKAEAQKMQDSLTDCHKE
AGDDAKLLNCVNAFDSPFDEIMATIRDVPDSLGFCYEKCKLTISEMYKIEQNRLNLKEIMTFVPEEKMACTTGCKVDKSGRSLTSRLTDLIHKSKKHDEKKKKEMIETLNR
CSAQVSGVKVETYNLIKCLNLYKPPFIDLY

>NvitOBP39

MKTFTIILILGKAYCQFGNASRLEDMKTCLVENGFTDSHADLERLQAIGDPEHVDRLKDVSREKMAGVIACMFQRRHGNKNLNEALQNLVERDDKVTEEERKKMLETLK
SCNANATGDNIKLLSCLNIMAPPFDVLIAAFRDLDDSVGICFPRCNVTIGEMYKMQKNMEMKTKVKDLLKIVNEQKMGCFMTCIVDEEAKINRLSPPFLRVLTDRINKSEN
HDEDQKREMLKTLSKCNAQVAEVKDKTYEMIKCVNMFKPPFIDIY

>NvitOBP40

MKTFAIILILGFAWRVSNASKYENMKTCLVENGFTDDETDLELIRAIGEPEHVDRLQDVSMEKMAGVMACLFEKQQGNGNLNNALESLVGRDDKATEEEKRKMLETLKT
CNTNAAGDNTKLLSCLNIMAPPFDVLIASIRDFDESVAVCFPKCEITIGEMYKMEENKSKVKGLLEIVNEQKLACFMACIVEEEEKNRKSPHFLKALTDLINKSEEHDENQK
KEMLETVDKCNAQVAEVKDKTYRIIKCVNMFKPPFVDLY

>NvitOBP41

MKTFVFILLIVYVCQWSDARETDFEDLIFNDLMSMCLIENGFTNSTADVERMYAIGDPEQADKLKDVPKEKIVDVIVCLYHKLYPYRNLYPSLRQLIDRDDTVTVGQIRQM
EHTMSDCYKEVGGKINNAELLKCVDITEPPFDHLFAAIRDVRQALQWCFVRCGVLISEMYTMEKNRDLPFKEYIKYIPHERISCLMACKAEQAVVNNSDQTLQKGLADLIK
RSKNVDEIEKAEMLKSLKKCSKKVYGYEDEHYELVKCIDLFKPPFIDLF

>NvitOBP42

MHYAIFYICITFAIILVLAFVWQPITASFRVKYSETCLMENGFTDSPSVVACVYQKQYADKNLNNALKVLIDRDDKVSEDEAKKMKETLATCNTNAAGDNAKLLSCVNLV
APPFDTLIAVIRDLPESQDPCFLKCDMKIGMPMNIQIITISSVITENGIRLSDSHFSGEMYKAEQNRNKMKDLVKHVPEEKLSCIFGCKVDEFDKTKPGGKVHDENHKKEMLE
TLKRCTDTVAGQPNENTLLGKCLDLFNPPFVDIY

>NvitOBP43

MKTFAVVLVIGFALQVSNALMPSDLETVVETCLVENEFTSSKDDVALVDYIGKPEGVDKLKDVPREKLAAVLACMYHKQYKNTTLYDMLKYLTEMDDKVTKDQYQQM
KETLTTCVQQCNGINLIRRDYPQGNDVELLKCVKAVDPPFDYLIATLRDLGEEPLNFCYERCGLKILIRILIIRSLRTLHTRDY

>NvitOBP44
MKLHFYAMNTLVSFFVLCFVWQLSHAIAFSDLTTCLIENGFTNTTSDLKRIYAIGDPKQADKLKDVSKEKMVNVVACLYDKLYPHQKLYPSLRQLIDRDDTVTSDQIEQM
ENTASACYRKVDGKINNVKFLNCVNITEAPFDHLFATIRDVQQALQWCYVRCRILISEMYTLQRNRYNPLKDYIQYIPTDKLGCFMACKAEESTMKNPDQTLQKRLTDLIN
LKKNIDESKRKEMLQTLNKCLTQVPADEDERYYEVIKCINLFQPPFVDILYNNFYSAYNIEKKELNI
>NvitOBP45
MKTFAIILCLGFTSQVSHAFFPPDVEKCMVESGFTNIPDDLGIIDGVGNPNDTDKLKDVPKEKLAIATACMFHKEYTNTLYDMLKLLIGMNDKATNDQRQQMMDTLNTCH
SEVKDNDAELLMCVKAFEAPFDYFIETLHMFGKSALYPCYGHCGLKIPDLYILEEHRNHVDELLKRIGTDNVYCITACKVERKEDNPALALYTMLSEIIDRRLKDDKNTEA
KAALEKCYTDGEYQKVEMAGSLGETIPKGSKVRDTYQLLKCMKVFEEPWINLMRN
>NvitOBP46
MKRFLILFIFCLTSPLGRADIYDDTVTCLKERGLKETDIRILDAVGEPGNEHILKDAPKDKLVDAIACIFRKDHKTNKALFGTLTELINMDSEIDNNKRKELLVTLNACNKKT
DGNDTNLLKCLEATQPPFDKYAAIIRDVTNVIKECFFSNKLTFSDLRYRKMLHRESVSEVLKHVGEDNITCAMTCEFDKNKHVYKDMDPNETIHKRLETLITKSSLPEEKKR
EMKETLDRCDHQARGDSCKFGKCVKLMRPPFVQLYFKNP
>NvitOBP47
MKSFLIIQLICLSGLLRAVNAESVTTCLVKNGIKSIDTATFEEVVKTNGDALKTVDKQVACVIACAFDDFTQKKESIYNYLTRIIKKDLDDEYSASERKEMLDILNYCNGLAF
GNDCKFLHCINVTKAPFADLI
LTVGIEDSGKGEDQKMFM
>NvitOBP48
MKNLSIVLIACFAVFQLTSADSNHIKECLTEHGLQETDMNLLKSLGSVKFADTTIKDEDREKLDCVLACIFQKESPGQSLHEVIKDVLSEDTYKSENLRKEMTETLNTCETQ
AGDDDCKLLQCVQITKLPFYDLLYTFVVARDMQKCFVDNGLNASDAVNLEHLNEPGWVEEILKKVDEDKLACTMFCLYSKQDKITHDKSLQQRFETRINESDKLIADQK
KEMLETLNRCGIEAAGDNCKLVKCIKILRAPFFNLYHQ
>NvitOBP49
MKRFIIIVCFYFIYLHTSSARRSDNLKKCLVEHGLRNKLYKRAIGGSNINSLDRDVNVDLKEKLACTLACSFRKEHSGNETLHAIFTEALNFGNFHILEDVKQQMRETLNNC
YTEVESNDCKLLHCIKIIESQFMDVVLAITNERPVRLSFENTNSVKKFKKIKFRIKWFKVMHMMIRSLQRHIPYKQ
>NvitOBP50
MKRSLVVLSFCFLLLQSVRIVADDESEELKECLQDNGLPTNIHKEVGKGEFEKLKDVPESKIACVIACSFNKRIRSNDFLFTSFVKAVNKDKSLGEDQKKEMLETINRCSKE
AKGDNCKFLDCLKITKPPFVNLIITPKDANDKKI
>NvitOBP51
MNRLFIFLIVLVAGSRADKRHDYGQKMSQCLIENGLNETHLKYVFRIGKVNLEIPSDVSEEALACMLACVYNFTITMRETQKTLDEAILDVINLDDMFNNDENKRKALKET
LDKCKTEAAGDNCKLLQCIKVTRDPFDKIITAGRYNYGYLRRDNE

>NvitOBP52
MKNLFVILALCVVSLYALDGFDFYRKKMDECLSENGLSEYDLKYVFKIGKPDYESPKNISDKTLACMLSCMYHLYKNRQTIDKAIATVINRDDSLTAEKKKNLLGTVRNC
TREAGDDDCVLLHCLDVTKEPFSKIITAGKKIKPSKIDTD
>NvitOBP53
MKILLFLLIFCVVGIYTQKHNDSAEKNKHAMDIEDCLNQHSNITKKGLSVKDIILKSIAPYDLGCITSCLKKKELKNGVTLNSYVIQNAYLPSTKFPDWYEKKNEDYQYVVI
ANRCINEAKEDECKLFMCLKAWELPFAHILSIKIERYITF
>NvitOBP54
MKLCWIILIALCIFGINARPNSEPDNDGGFEPLALQCLRELKKDPTLSAKNCDEIESSLTDDERNCILACMFRRNDPDKKSLYEYLKSQLSTIDNRIQVYRDELLEKLNSCKAL
VGEGNDCGVMKCIELFKPPF
AHWYLQTN
>NvitOBP55
MRNSLVVIVLICFSQIHAIPLTKKKEKFVPAEDSKEQCMIKFGLDPDFVDYLIGLHRPQIEINAYIGSKHSCIHACMVKLDQNLNPYDYVVDRVSADTKEEYERLIKLVNKCN
EKDSGNGCVLLDCVRRNKELRDFVY
>NvitOBP56
MKLFVFCVFALCLTAANALFGPKLKEKLLEREDACLRETGNTLLSIDHVRRTKTLPEDGSLDKFALCLLKKHRIVNDDDTVNKDKHRYYLILDDGRKKEYAEDCVLSSGG
SNNGEIARHLLSCLLKTDIFFIDWSYRSQEVLSQMRQRQKQKTQA
>NvitOBP57
LTFNFRTNTDILFQASTGNIVNDTLNRKFLLLVKTCANKKLHMSDYGSINEDVNSCHLIFDNSSMLETVEKCKNRRETASRDMTCFLKSHVLIIDPYIGAHESA
>NvitOBP58
MKFFISCVLVIFCSSSAIGLLSHEAILSLQRDQDDCVRESGVTRSTVEQAHLDRVIHNDENMAKFAACMLKKFNVMSDDGKINEDVYSYHLISDNPAMFETAEKCKKRTGS
DVDETASKIMTCFLNSDVFVLDPYIGVHKRA
>NvitOBP59
MKFYALCVILLCSSAAFALLEARVRDYLYEYQRDCMIESGADTSLVAAADRARIIPNDGLLDTFAICMLKKYNILHKDGSVNQDHDSYTIFSDNPDVYRISERCKAKIGKD
AGETARKIMNCFAEDGDSLLPYSTHPPPTPC
>NvitOBP60
MKIYVLCAVLFFTPTVFGIYSSAIWDALLHANEEPCGRSAGLSEESIESSRRARYLPESPEMNVFAFCVIRVLNIMSKDGKVNPDIGSYLVPTNTPDITKVISEKCRTHVGVDA
GDTARTILNCYLQADQLVISLPSDAQLTFN
>NvitOBP61
MKIYVICAVLLFAPAALGLFSNGIWDVLHANEAKCQLNSGASDASIEDARRARKLSESPEMNAFAKCMLGIYNVMRPDGSINPDFQSYTVPTDVPNNTWRISQKCITLGGT

DSGDTARKIFNCYTENNQLVMAWTPKVSV
>NvitOBP62
MKIYVICVVVFFLAPAVFASFSPLIEDDFHAYEADCGASDESIEAARRARQLPQSPQMNAFALCMMQKYKVMAADGSVNPDVRSYGIITDGPDNTWRVSEHCRTLNGNSA
GETARMIMNCYLDNNQLVMGLTPRVSA
>NvitOBP63
GTFLPSIDDTLHEYETNCARVSGATHSAIEIARNTKMLANTARLNAFAMCMLQQFNVMDSNGIVNPDVMSYSIISNVPNATAGISQQCISKRGIDAVNTARMIMNCYLRAN
QMVLALSRRDCT
>NvitOBP64
MKKFTLIFVSCYLVFSSMHRVMCVTQCFFNELNLVDQRGFPERSAVIGIMTQNIQDPELRDFVEESVIECYHYINNNNSGRQEKCQFSQSLLSCLAEKGSERCEDWDDE
>NvitOBP65
MKSILFIFAIVCVVGVFSDDDKKDLTREQILECVAESGVDETKVEDIKLGNQGLETTREIDCFAACVFKKQGIMNEAGVITPDKPMDNEAAKQCVATTGADACDTAGKVL
KCFISNNLVSLMDLDDD
>NvitOBP66
MKSVLVVFAAICIAGVLSDPKGDIDACVAESKVDTKLFEDMMHTPDFKATREMDCFAACMFKKDGVLDADGNVDASKLPNVDVSKVCGALRGKDACETAGKIIGCFAE
KGVMDVFHIV
>NvitOBP67
MKTSALLLVAFGIFAFTELSTASLDKWFEECVKSYGHTEESVSKLPDLEKSCVIHICFMRDV
GLINEDNSLNVNYLLERRKSHVPESKIYDAVRTCNAESIDTLAKTCEAVKCLMDLLHESDFNTQPNVTD
>NvitOBP68
MFAFAVFAFTNVLNPMYFHTFYETTFFLSCVESIFKLTESERSCAFQTSFLRELGLINKDNSFNVNDLLKQRKSGIPESKIHDAVKTCDVESLDSLEKTSKAVKCLMGLLRN
MWLM
>NvitOBP69
MKLFAVVLVFFALGSSSVALDEEERGVLRQIRNVCVVESGLSPYELGFIYRAIRPAKKLAQASRCVIQKISELQSENETVKHIADRGKAALANAPISNIADNVLGSCQNLLGQ
NGCIQVLELAAKIIDNLRSRRQ
>NvitOBP70
MISGEEISLLVIFTICWGINLKCKHAGEIQLHLQDKEAAEKCSKDIGITLETVYATMKNELKDADEKLKCFAACVFKEKEMLKDDGPINVAKAIEDLPDEIKDDVRDAMIKTI
EKCSQKKEANECETVFHAVQCATLDMSKLKLF
>NvitOBP71
MKVAIVACVLTICSIFAGSKADLTEDQRKILQPLKDECFQETGLDAVTLEKFKKEALQKFKTTGEVSNDEKVNCFSACMFKKIGFMSEEGKFEEDTVRALMSENFPPETLD

KAIENCKNEVGKDHCETAAKLIVCFMNNKAGMENV
>NvitOBP72
MLFFTVVLLFSSVCTATKEEEEFKSELAECKNLVGVTEDYVRDVFKSGLKGADEKFKCFIACLIQDSYKFNDGGVFDAERTIANDRGPAGLLRDYTNKALKACSNIKGYSE
CDAIFKVYKCMVENVEKLFNARNDRPSG
>NvitOBP73
MKDDGTLDIEGTSMIVGRRLKYAERNDVIKADQACSNIKGDNACDTIFKIVGCSIKNLERYR
>NvitOBP74
MMDIEGSSMIVGRRLKDVERNDVIIAVQACSDIKGDNACDTIFKIVGCSIKNLKRYR
>NvitOBP75
MRVLLVVVSVCFVGSYADYADDIRKLQEETKRIEEYRRPCLKEVGLYADPANGITSQPASSPTIGQIFCLWACLYRKNGSIRPDGSVDEAAVRSKNPELEGPLDVIISKCENQ
AGENTCKLAGCLAKAHFNLLE
>NvitOBP76
MMQGSLCALVVLSLVCLVRAGPPDWISAEILEMVQSDKGRCMAEHGTTEALIDDVNKGNLPNDKAITCYMYCLFEAFSLVDEEANIEVEMLVGFLPEHMQAVANELIDV
CAKLDGADVCDKMYVMAKCVMEKRPDLWFML
>NvitOBP77
MKIVVLCLVVLSAVACVSAGYREYQNACLDENGLTKEEFYAMKRNQDPRSGCVTACIMKKNGSMKHGIIDARGIKRRMRTLLAPFISKDKLYEKIDYCVDEAENHVGVC
EKAYVLQKCLRTPRANNVQGERQKMID
>NvitOBP78
MKTIVFTLCMMTVAVTCSPRPGGRGGGSMFSRESVKKCMAEMDIKREDIKTLKQNNDPKLSCLNACAMTKEEIMDEAGNIDADKLIKATLEIVQKKKPDINVEELETAML
SCIEKAKEVEDKCMKAKTLVVCSHEYWKANVKGNPSSAGGEEE
>NvitOBP79
MKLFFVTLCVLFAAVYGATKSDSKSEKIFHECLEENDIKESDFKNLEGKKDPKMRCLMACILEKEGALKDGEIDGDVIKKDIIAEFTEVDAQKISDAIDTCVDGANDLSDIC
EKTSFIGECLKVELDKLEMNMN
>NvitOBP80
MGGFVTVLYFLSIIICVYSLNWSEAKKHVQECLDEYQITREDVAKLKKEESPDYNCYIACIMKKRGSLVDGKIDEEKMLEILKQLHVLNSERTEDKFRICATEANKQSNECL
VAGDMIGCLYFKSN
>NvitOBP81
MKVIVLLVTVLTITIHVSCQTDEEVHKIKEKCFDLSDIPVEDRVVYNPENPKLKCFNACTYTGVGMMKDGKIVPEKYIERLQDSLKNEKKSDVEAFMKHMEDCAVMANKL
SDECEVAYSMIKCL

>NvitOBP82
MKRVMALVGAFLLVSAVQCDDMPFWNEKVECAQSMGISPDQMTSMLTSNDAQMNCVHACVLEKIGGMVDGKLSLDSLMESLEKLKAEVKDYDATKAGIHQCFDQAS
GDRCESAGKFAMCMQEHMQG
>NvitOBP83
MRLTLQLITLVSLVAIFKTTESKMTMDQIKNTLKPFKNSCIKKISPDVAMVEATKSGQFPEDATLMCFLKCVLSMMKVMKNGEILLPSIMQQIDIMMPDEYVETMKEICTN
CYEMSLKVDDACEKAY
VFVKCYYNTNSELYFFP
>NvitOBP84
MRRSILITSILIILISQYKLVKCKKMNLDELRDMLRPMSKSCKSKTGVSDEMVAATHQGIFPREKPLMCYFKCLSVMLKVMNKQGEIKPKDVERQIDLLVIPELAPTLKKIGT
DCYNKVAPTNDACAYAFEIVMCGYQTDPKYYFLP
>NvitOBP85
MRSVLLIFCLSSVAVRVSAHVSPVADSFKACLAESGMTRDDFIKALQSSDDSKAQCIAACTMEKEKFMSDDKINVDAIIAKMEDVSQEIGKVQITDLVMNCAAEAKDKSG
KCGVAHSVVRCIHEELRKEGWI
>NvitOBP86
MKSYSVILLAICFAAIYSSSALISIEDKAACLKKNGLNNTEKWDLTAQFDYRLEKPFTCYVACVINAIKKPEETVYGKLSEVIERGHVIPASLKKDMENRLDSCYRYNGEGD
DCKLLYCVKILQSPLIKLSIYSLEDIEL
>NvitOBP87
MKFLIFVISLFTVVARSRQSLADIEACASQYGVENVTRIPDNDRPFKQRDPDYECLRACLWRKQGIMKNGKFDLDKAFNYFKKTTRFPLTVFKEKLSVCVEKGNQEKNECG
VTRVYVDCMNGSPKARK
>NvitOBP88
MKLLIFVISFFIVAAHSQPRSMDWKGCMEEIGVSKDDVKSTEWGDPKSRCVLACTFKKVGVINDGKVVFDVAFDITKGEAQDSSHDKYIEEKVNSCIEKAHQETNECDVSY
VFMECMKTNNNTAKMANGTMSI
>NvitOBP89
VTTNNDLFKIKDAKVVSNVAFDMAKEDTRSSSDKDTQEKVNMCIEKAHQEANECDVTYVFLDCLADGLIMAKKQVVNIK
>NvitOBP90
MKNLALLLLTLCVVSCLLINGARAGVSREQMEKMANGFRNTCVGKTGADMSLVEGIRVGNFVEDPTSMCYTKCIMGLMKTFTKQGNIDVEMLVKQINVMASPDIAGSM
VTNARKCHAETSASDPCELAWLFTKCIYAADPAVYFFP
>CvesOBP6
MRSSIFVLVACALVVAVLGNEERRAKHKEIFEKCLKEVGIEKEDLHKAHEAHKNGEEPDQKIKCFEACLAKEMGTMVDGQFNKDKMLEKLPADIPDREKKVEAITKCSEE

KGSDDCQTAYLVMKCMHDNKVLPPPPHHHHHHHHDQ
>CvesOBP18
MKVFAIVLAVCFVGALAELNDEQKAKLKEFRTHCVTETGVDESFIDKAKNGEWMMDDMKLRCFMKCMMMKIKVMKEDGTLDEEVTKKKMGNDLPADKIDEVMMKC
KDMKGADTCETAMKMIKCYAEQKAFVSLTH
>CvesOBP15
MRVATGVLIAFVLAVGLFGDNSEARRLDIAKKCASETQLNEIPVKDIFSHGKEPNKKIKCFVACMAREDDVIVHGKFNKDKIFAAIPDDFPNRDKVVELLAKCSDEKGSDD
CETAYLGVKCLHDNRIPLPFLLETGN
>CvesOBP10
MSKVYFCSLAVAFLFFALSNVDAKLSITQMRSIAKPWSQKCANKAGTSQELIEGHRRGEFPEDQTLMCYLLCNAKMAKIIDGNDKIDLVTVKQQLDIMAEQPDMERMMS
AFKICADKMTATEPCRLAYDYSLCYYNMDPEIYMFS
>CvesOBP21
MKLKRADYNQTDQQLKCYLLCFMQKNGIFDKDDINVEKALRHLPRGVQGPSKKTLEYCKKIPSNDTCDKAFQLAKCYFKAQPEVLKTVSFV
>CvesOBP9
MSKVYFCSLAVAFLFFALSNVDAKLNTDQVRAMAKPWGNSCANKMGLRQELREAHARGEFPEDEDLMCYLQCNARAAKIMDKTNKIDMTTISTQLRLLVEEQSAERMV
KVFQTCADRMTATEPCRLAYDFSKCYYEIDAETYMFP
>CvesOBP13
MAKFLLSFVNILLLVAYVHSGPVPEEFLDVQPTIRAACVKESGIASEDLINKAAKGEFTDDPQLKCYLKCIFDQFRLVSKRGINFDAMLALSPPSMKESATKMIKECRETKG
KEGDLCDLSYEVTKCLYNSNPETYFIL
>CvesOBP1
MKNFIFSIGLLSIYFATTANSLLSETNLLQRASEDRERCLHLLSNETDGVNITQNSQNFRCYQTCIIKRGGYLQDGKISLEKYEKMMDSRMKRHKELHMNATKACIEEAEKG
ETECDVGVLFIACHRRQLFKQYRRNYARNFDENHHQQLTL
>CvesOBP4
MFKFVVLVALAVVSVNGLSQELKQQFLERLNKEGHECAAEVGASEDDVNELKAHKFPSRKEGECLILCLHKRFNMMHPDGTINTEGAIQTMQPLKADDPELYDKFMTIG
RHCTEDVKIQDDKCRYATELVECAVKKGKEMGLDESIFE
>CvesOBP2
MATLTASVMGTAEVMSHVTAHFGKALEECREESGLSAEVLEEFQHFWREDFEVVHRELGCAIICMSNKFSLLQDDSRMHHVNMHDYIKSFPNGHVLSEKLVELIHNCEK
KYDTMTDDCDRVVKVAACFKVDAKAAGIAPEVAMIEAVMEKY
>CvesOBP5
MTQIMSFLCFSIFSHRNGSIPLCSLSLPRVSLKHSLFVSFSFSWISSMEVSAQHSLFLLNLHLSELTASSSLLNRNVDGIGMSKLL

>CvesOBP19

MKSCKFAIVGILLTVVAKVHSDPELTSDTRDTVKGVIRSCSAKMSDENFIKMIQENDEHSMSIAAKCFAACALSHVGLMKDGKMHVNQIEDKLSSMIDTIKLCADEANKN
ANECVVVGKFGECLKEHDM

>CvesOBP11

MKQYLIAIFILCALIYEIRCDSNLITRKQIREVRKQCVDEVGLGRVIQDNPTRTNNSAEVHHTRKCVRACVAKKFGLINSEGKLDKEKFLERMIPKKDMLLYEKVSEAITTCA
DQQGNDICETHYLVMECMKGFRPFPRMRMNDI

>CvesOBP16

MHQFAVVLFFAAVIVTEAAITPEDLVKFGRARRDCDRSVPVDSSVIDRVIQGEMINDRQFFCYAACVVKELGLATADGSLMVDKVISKIPEGLPNRDAIVNAANECGTRKA
ADPCDTAQMVFNCFHEKNVPSLLMG

>CvesOBP14

MRVATLVLFACALAVGVLGEMPSEPERAEMMKKCMTESGIDESDTKNLFHMSQGEPSQKIKCFHACLSREMGVMVDGNINKDKAIEMLPADFPDREKTIEVFSKCSGEK
GSDDCETVYLVFKCLRDNKIIPPPPPRPE

>CvesOBP8

MKTITLSVVGILLAAFMTVNADANTEMAEKIKKIFEGVVVACKDKITPENFVKLMQNPAAMSSNSELNCFKACAATHLELMKDGKLQVEKFEENVNSFLGDDKKEMAKT
IIDATKSCVEEANQNENECDVAGKFEECMKKFNVPQL

>CvesOBP22

MNIAIVSTLIIAILNAGYFYHAEAVMTKEQIQKMMNSLGKTCASKSGVSPEMQEAHKRREFPEDRGLKCYFGCMVKMSKVDDPLILCSRSIKITKSMWMEQ

>CvesOBP3

MFRFIFLASAAFVAVNGLSQELKEKFMERLETVGGQCATDVGANEDDIAELIAHKYPSRHEGECMIFCFYKHFNMMHPDGSLDAEGAIKAMEPLKADDPDLYEKLMEVG
KHCAETVKADDDKCKYAAQLAQCGTKKAAEMGIDGSILE

>CvesOBP7

MKYFALVFVGLLFTFSNGVSGEQEDCPLKKAVQASIEACKDKLTDDSLNLLKEDEGADNEEIRCFKACILKDSGIMEDGKIQVEKVKSALNEAIEKAEENKDKLEEMAKAI
IEATEKCAEEAGKAENECEKAHLFVGCLKGYENQE

>CvesOBP12

MVRVILSCVFLGFLMQTSLVSAKLPDWVPPEIIEMAAGEKGRCMSEHGTTQGMIDQVNTGSIPNDPALTCYMFCLFESFSIIDEDGVLEYGMLLEMFPEEIKNKASDILGGC
AEQAGADNCEKVYKIATCVQGKDPSMWFMI

>CvesOBP20

MGKTRGVHETTPELRNRMVGMYEAGLGLREIAAAVNCSQRTVKRWLNRFDKEGTVETRERCGRKRATTTEQDAAIVRLATQTPITAAKAVLPALGLTCSVDTIRERLHK
AGIHNWSPSRKHIQKIPLGDVDGAAIQQAVWRPTGTSLGKKKSQKPDDSAKKPGAVVKRKVLKRRAKPSNNTDSEESAASKQPQTEEPPQLQHQDQLNSSFSFQPDNLSQ

VQPRSDESPEMQAASLMNSQPEFNNALSLVVSPQSMYPHHSGVDVAQNWPLDLGQNAHHYPQSQAESVALHQPSSQQHLQELHTPQEQEQRAHEQHMQEHSQSIQQLH
HHQPQHHLHHHPFDEPHHPNCVDQIQSQLSLEPIREPSKVVRSNSCSNSSIENSSDGDGELNSKDDYERSRLRKKKGGKAKGTLETSVEIRNRMIGMSEAGLSTLSIALAINR
SERTVKRWLERWNKEGNVQTKERKGRRRITTKEQDEAIVAMATQNPLTAAKHVAPALGLNCSVDTVRERLHKAGIHSWKLGKKQGEGNVVHTVHLWQPSGNRPNKPKI
NNNESSERKKKGLVKKISAGACNVKRTSNNNRKMKKKKTDEAPKNLTTNTGGVQEQNQIHQYHDNGGMTYSSAQSIMQPVHVSQAQSLVPQQPQIPAPLVSIPPPPPPPS
AASVLPPGTSVPHSMQPMGPIMQRPDCRMISSIPNTQNSGMIGYSSCMIGNNNHMDHQPDPLNYEPFIWTF
>CvesOBP17
MKVLVPIFAICFVGVLSNIAEPDSNRRENLKQCVEESGVDQTAVDKLQDKVWEMDNKKLQCFSKCMMIKMGVINNDGTLNEEYIKKNMSDEFPEGKADEFIMKCKDPKG
ADACENSLLMLKCFFDNTVFTMHKNN
>CcunOBP1
TVIVSFLFCIATVLAELTEEQKEMIKPYKDACLAELKLDEAIIEQSKKEYLEQGKTEFSDQLNCFSACMFKKVGIMTEEGKFDEDMARALASGQFPEDEINKAINACKNEVG
KDICETAGILFECFLKQRISV
>CcunOBP3
VICILILSVHIINEAHAGASREQMEKISEGFRKTCIGKTGADLAIVQEIRNGNFIVDPLAKCYTKCIMGLMKTLTKQGQIDAEMMIKQINIMVSPDIAGHMIAGVRKCHVEVS
ADEPCELAWLFTKCVHDENPEL
>CcunOBP4
LFVICCYCNFAAYSRNITFTNDELDENIQMCLAKTRLSQAFFKSGDENLKYLTEEQKSCFLACMFKKAGIITHDGSVRSVTEEMDIATANAIDRCQSLAEGNLCKLAWCLY
KTDKFSIPVI
>CcunOBP5
MHSFTVIVVFCALFTKNALADSYNVELTEEDREIFKKCIKEGGLTKEELNAAVRNFDKNADRKVKCFRGCLLRSHKVIKDDNTIDGNAAVNYYHVEDVEPLKKLILKCSAS
TTGNDYCDVAQSVESCF
>CcunOBP6
GETSAEDETNITLCVNGTRIDLVVNLLDKECIEKAENS
>CcunOBP7
MKFVIFSFFALFLAVRDIHGNDESIQNLKECLNENNIKVDIEKLKEHDDPKIRCILACVMEKEGILENGDIQYDLLKEDLLEDAEQLGEKKISEIVDYCGNLAKELTDVCDKT
NLIGMCLEEELE
>CcunOBP8
ALLLLLFGLTNAKPGTRPSFVSDKMIATAASVVNACQTQTGVTTADIELVRNGQWPDSMELKCYMYCLWEQFGLIDEKRELSLNGMLTFFQRIPAYRNEVQQAISECKAL
GKYFATGDSCEYAYTFNKCYADRSPRT
>CcunOBP9

SFVIVFALCIAGAFAGLTDQQIVKLREYKIGCLAETGVSEDVVNKLKVGEAVVFDEKLNCFSACILKKVGIMRPDGSIDEQVARDKLPKDWPQDKVDHVVNACKVQVGK
DSCETGGKVLGCLAKTRAIA
>CcunOBP10
KTFAIVLAVCLAVVYADDPLKDIPKDLIKTCLTENGFDAAQYPNGLRNVKVPDNQEKNRDCYYACMMKKMNLMKPDGMLMEDNLKSKFNLNLETLQKALNTCKAQVK
GNDSCKLAACLMANRG
>CcunOBP11
TLLSMFLLFVSVSTTLASFSADIEISLYEYEDDCSKESGLGAADIEIIRKNHAVPDNEIASQWAKCMMQKHNVIKSDGTFNMDVKTIAIPADTDTVIRAFPICKNQIGKDMAG
TARLMMNCYLSNSDGL
>CcunOBP12
MKNVVVCFIVIVFGAININAGEIPKEIAHMVADVREKCHRETGVDIEHVDRTAEGYFHPTETLGCYFSCIFGHFDLLDHNGHIDFDKIIPKIPESFKDHGMEMITACRHLTGK
NPCDMAFNVVQCFQKTNPEK
>CcunOBP13
YCALVLLLCMQAILLVHAGPPDWISPEILEMVQSDKARCMGEHGTTEALIEEVNQGHLTDDRAITCYMYCLFEAFSLVDEDGELEVEMLVGFLPEHMQGVANELIDACAK
EPGTDVCNKMYAVAKCVQQKRPDL
>CcunOBP14
ISVALFVVICIVGVYSHPHGEHGHKLTPEQIARIMADVEECARTNDIGHEVFEDLKAGKNPTPSRNLSCFSACVLKRNGVMNADGSTNHKPTDSDVAKECKDLRGDDDCET
AGKIVSCLHKNNLI
>CcunOBP15
LLLALCVAICFAGVYCDTAPKPEEVQYVKDCASKNNMDQKMIDDLKMQKTFFASQAAMCFTHCVMHHNGMLDDEGNMTDMFKKIPEAAECQSMTGNDKCETAAKIM
DCMVKKENAD
>CcunOBP16
MKVLLVLVCCLAVAMAQFSSDKQRASAMNECQEELKVPDSEVEDPSKLGCLYACMHKKVGYTDADGTYNLRKLAGSAYNQRFEEAAQRVMNMCAEQAKGDPCKMA
LCLE
>CcunOBP17
IVITILLVCATQFKIIECGKKMDIDGLKDMLKPMSKSCKTKTGVSDELIAGTANGIWPRERSLMCYFKCLAVMLKAMNKQGEITLREINRQLNILVIDELVPRMKQILEQCLA
TATPSDDACEYAFNLIVCGYKADPTL
>CcunOBP18
MQLFDVACILGIVVIINALSNEQRYDNYIAVLKNCLKELGLSEEVYAYASVINNTDGAYDKAKCADLCMFKALKIMKPDGHIDLEKALEHLLSGEPGVQRDIMKTNIETCS
KKKEDNDCDTAHNMMTCAVG

>CcunOBP19
KSLVQIAPFVLILLSFETVNAKMTMDQIKNTLKPFKTSCLKKTGVDIDLVDGTKSGHFPEERSLMCFTKCVMQMMKVAKNGEILINAMMQQVDLMMPDEYVDEMKSIITN
CGPEANTKDDGCESAFTFAKCFYQSNSDI
>CcunOBP20
MKRAFSVLCVFLILGYAYSIDLQFVSQMKECGSEMGFSPEQVMEMMAKNDGQVGCLRACVLEKLGALQNGNLDKNVLASLLEQNKDTIPNYEQIRANLDTCYGEVTSG
GLTDQCQIGGKFSTCMQEHMTG
>CcunOBP21
AAIVFTLCSVGAFAGLIEGDVSPPKPNITKECLKEYGIDIEQTNGAPLSDEEIYCIPACAYKDYGIMRPDGTIDSDKAESYFGVNDHEERSIFFSVYEVCREGKTHCKLVQCMF
DNLKNHWKSS
>CcunOBP22
LPVCLIFSVFCFSSAHALLTKEAIESLRTHQKYCVRTSGVSEDHVEMARLDRQIHEDEYQEKFAVCMLNKFNIMNTDGSINKDEISYVLLTDNPWSYQTAKDCTALVGSNV
RETARKITNCLLQTDIIV
>CcunOBP23
CFAIVLALCVVGAYAATLSDEQKAKLKGFKEACITESGVNADLVNSIIKGGEIKRDKNLDCFSACMLKKIGIMRDDGTIDVETTRAKARTTSVDVAKADKIIDKCKELVGK
DACETGGNVFGCFILGKDFP
>CcunOBP24
CIFVILVIAGCVYGDLSEDHREARKQRLDKCRKEMGITEENPLSRPPNLDDPKEKCFYACLMKESGKLVDGKMVAEKVLSAEKKRRPNYNDDIEAKLTYCVETANEQSDE
CEMAATMKKCTFEKLGPL
>CcunOBP25
MKLLLLTCSYLIICMKMSTSAPNHERVDDVDLNDSIDQCAAQIGLLIEETRRSFTMPIEAPGNCVMACVWDKIGLMDIDGKIIKEEMITSIHPTLELLPNITRVTEDDFYQCVD
EANRFGDPCTVISEYFKCLIKDLF
>TpreOBP1
MKLKVSGSIIFILSIYLLNVQCAKMSLDELKKMVKPISSTCQKKNNVPQDLLLASYSGVFAREKSLMCYYRCLATMLKL
MNKQGQFALDKMFTQVDLLVVEELAPRIKEIAKICFDSTPKIDDPCEYTYDLVVCAYNIDSSLQGILSKSQMKNR
>TpreOBP2
MTVRPRPRLGLGLLLGCYAISLVYAGTRPSFVSDKMIETASTVVNACQIQTGVTTADIESVRDGQWPESQELKCYM
YCLWEQFGLVDEKNELSLNGMLTFFQRIPAYRNEVQNAINECKALGKYFATGDSCEYAYTFNKCYAERSPRTYYLF
>TpreOBP3
MRLSTTAMILSVFFISHIAVESKKMNIEELKKMSKPMMNSCQKKTGVKTEELEAAEKGTFPTGNKPLMCYFRCLAV

MFKLTDKDGNISLHHLLHQIDLLVIDEIAAGVNDMLQFCFEHTPKLEDSCEYIYELVICMHKRNTEMNFFEGSLLS

>TpreOBP4

MRRFASFLALFAMVVLASGDMEQMKEAFKSCKAEVGIAEDTQMKDIPSSKVGCLHACVMKKFDNMKDGKVVVE

NILQRAEKKMNPLPEEMKEKLTKCADDANGKGDECEVASYMHECWWDSMKSMGPPKGPSN

>TpreOBP5

MQKIALCLAIFLVTYRVEAANEVPAEIRDLIAGVREKCHRETGVDIEHVDRTVEGYFHPSETLGCYFSCVFNQFNLLD

HDGHLNFDEVLKRLEGLESFKEHGTEMIEKCRHLTGKNPCDSAFNLVQCFQQTNPEKFFVI

>TpreOBP6

MQPARVFSALAAILTFQALVVYAKRPEYITDEIMDMISNDKNRCMAEYGTTEALIDQVNDGHIPNDRAITCYMYC

MFESFSLVDEDGEIEIEMLVGFIPEEFQEIAAELIEACATLPGEDVCDKMYKRSSCVQAKRPDLWFMV

>TpreOBP7

MYCTNRLFIAGAQAAALGHVSPVSESFYACLAESNLTKAQFIETLKSNDTEVAQCIASCTMEKEKFMTGEQIHENAII

KKMAEVSQEIGREAITYLVKVCAEEARELKGKCGVAHSVVRCIHDSLLAEGWI

>TpreOBP8

MKNTFFIGFCIFCIVCTGEVFSAATQEQMESMSNGLRRTCVNKLGITTADIEGIRGGNFVDSPGARCYIKCVMGLM

KTFTKQGTIDIDVLVKQISIMTPSTIGKKLIEGAKTCYDEVSSDDPCELAWMFTKCTYLKGPDSFFFP

>TpreOBP9

MSVSTHLPIYVFFCSFVVLSSVANVRSAEAPKEIQGLIAGVREKCHRETGVDIEHVERTTDGHFHESEVLGCYFSCVF

NSFDLLDHDGHMDFDKLLKKLPAVESFADHGAAMVAACRHITGANPCESAFKIMQCWQSTYPDKYFVI

>TpreOBP10

MYLIVGLVLVSCILHVHANEVPMEIKNLVAGIREKCHRETGVDIEHVDRTVEGYFHESEVLGCYFSCILNSFDLLDHD

GHIDFDKLVVRLKGTDSFREHGMEMVAACRGTTGKNPCDSAFKVFQCFQKTNPAKYFVI

>TpreOBP11

MKTTLVFLAVCLAVTFASTLKDEQKAKLREFKEACIKESGVDAAVVDGIVKGGPITRGDKIDCFSACMLKKIGIMKPD

GAIDVEAARGKVKTTNADPDKANKVIDACKDLVGKDACETGGNVFSCFITKKDFPVLD

>TpreOBP12

MKSFTFGLVLVVMGVCNAAEIPAEIKGMVAGLREKCHRETGVDIEHVDRTVDGYFHPSETLGCYFSCIFNAFDVLD

KDGHVDWDKAITKLEAVESMKEHGMEMINACRTVTGKNPCDAAFNIVQCFHKTNPEKYFVI

>TpreOBP13

MKTAAFLLVVCFVAVFAEDPIKDQAVSKDLIKACLTENGFDAAQYPAGLRNAKVPENMEQKRNCYYACMMKKM
NLMKTDGALNEENLRSKFSTNLETLNKAIDTCKAQGQNDFCKLASCMMANREI
>TpreOBP14
MVRVRLALVLAFLVSATVCHNITLTNDQLDTYIKTCLTKTRISQAFYKSDDENLKRLSERQKSCFLACMFKKSGIISDD
GTVSSVTDDQEDSATNKAIKRCTKRAKGDICRLAWCLRKLEKFSLPIIVQKPRIVQY
>TpreOBP15
MKGILYLTITILCMHNVKAGEVPEEIKHLVVGLREKCHRETGVDIEHVDRTVEGYFHPSETLGCYFSCLFNSFDLLDH
DGHLDWDKAISKLDAVGSLRDHAMDFINACRGTTGANPCESALNIVQCFQKAYPDKFFVI
>TpreOBP16
MKCIFTLTCLLVLTHTIHCEYEDTMFMDEMIKCAKEMGISADQLKEALETKNDEKLSCVNACAMKHLGTLSNGKIQ
KEKIFELIDKYADKIKDSDKLKEVVTSCADEVSSSGDMPECQLARKFTTCFENHFKV
>TpreOBP17
MKLFIEIFILAVAAFCLVTAGRPDFVTDEILEMVAGDKARCMNEHGTTESMIDAVNEGNIMNDRAITCYMYCLFEA
FSLVDEDGILEVEMLVGFLPENMQASAETIVNSCIDESPGDVCDKMYATAKCIYDKRPDLWFML
>TpreOBP18
MKYLAVILAFCLAGAYAGLSDEQKAKLVEHRKVCVAETGLDPVVVENIKKGQPVQFDEKLSCFAACMLKRIGIMRP
DGSMDEQVARAKLPKDLPKDKVDAVINSCKTQVGRNQCETGGKVLGCLLKTKAVSILA
>TpreOBP19
MKIKLATCIIILINLSAIDSKMTIEQLKNTMKPFKNTCLKKVADVDPVMVEGTKQGNFPDDPTLKCFFKCTLQMLKVL
KNGELSVPAMMNQIDIMMSEELVDKTKAIVVDCDGKSKNLGDICERSFAFVKCFYEADSELYFFP
>TpreOBP20
MRLIVLFFICVLRVRADSNGSDLGSKDDDMVTCLINSGLDPGIYSGQKIGASAPTENQTNCYLACMFKKIGYMTKD
GSIDVESILSTSHGLRKRAKARQRLDEIVNQCNMHAKDDVCKLARCFQDLRKSLLEKN
>TpreOBP21
MKNIVIIISLIVTAHAADHSLDKDELEVKEYFEQCLSEHGLKESDLEELKNKADPQILCITACVFEKQGLLMKNGEFNK
KEIIKVEQEEDPNFKQDDFDEIFSFCEEKAKGIDDACLKGNTLTMCFLDEISQLDDKN
>TpreOBP22
MKFAVFTCLMVLLVVQHYPLVQCKKMNIEELKGFTKPLTKTCKTKTGISEATLAQIAKREFPPDPVLKCYFRCIAQM
GKMMDKKGNLILENMIKQVELLIVDDISPRVKSVFTECFGEMTAEESCQLAFDFIMCIERIDQELNIIV

>MmedOBP1

MKNIIIFTTAITIFTFINFSQTEARMTMTQIRNAMKPLGKTCLGKTGLSKEVQAGQHNGEFPEDEALMCYHSCLLKLAKISDKSGNINLDTVHKQIDLMMPEDLIARAKAVT
TDCFGEIKSTEICRMSFEFVKCYFIKGPEIVFFP

>MmedOBP2

MKSIIFLGVLLTVLISNKAEAKSVQKRECPFKKPFEANAPKCMDKISEENMGRMMQGNMDNDEIRCFVGCVFENAGFVKDNKVQMDKVREAVDNFVDDYKYSKEVGDQ
VYGVVSDCAPQAEKGANNCEVSSNLLICFKTNNKFT

>MmedOBP3

MRGSVLAIVACALVVGVLGDDDMKEKHKEIFKKCAEETGVTKEDLHNHKRGEEPETKIKCFHACIAKADGAMVDGKLNKDKVIEKIPADLPDRERIIEAVTKCSEQTAAD
ECETAHLVFKCLRENKALPHPPHHHHHHHDE

>MmedOBP4

MKCFTLTAAGILFTVLITVNNASSNSNMEELVKKSMEETFKACKDKLTPENFALLNKDPHADNQEIKCFKACGMNHAGIMADGKIQIEKMEEKLNSLLGEDKKDFSKIIIG
RAKPCVEEANKGENECDVAAGFEACVQKTINTKSDN

>MmedOBP5

MKNFVVIVILALYFTATTESLQEIMNTFQKARLEVRAPCLHLLSNETLTTLKTRRHLDNPEIRCFKACLMERQGYLKDNKIFIDEYEKLIDVNLKRIKELNMKFARACVNEA
EKSENKCELAHNYNRCILHQTRKHYNQTAEENDENQNQHL

>MmedOBP6

MKNTLFFTLAAAFLLGYNIPHVESRMSMAQTINTMKPLGKTCAAKTGLSKEMQDGQHEGQFPEEEALMCYHTCLLKMAKVADKTGKLNIDAMVKQIDMLMPEDLVDK
AKTACSGCADEVTATEGCRPSWEFMKCWYGRAPELYFFP

>MmedOBP8

MDSNIKYMCFLYIFIVVFMFFSEAIDQSDPHASTRKKCSGEFKLTDEILKLGEQDPSDFSCYLFCLFKDINIMNQKGEFDPNLAAQEVQDNLREAARKYIFMCYDLVKPNMT
SDGCKNALEMVQCFKEKAPEMYEMLGLFHPPSNEPLKMTQ

>MmedOBP10

MAKFLLSSVGVLVLIAYVQSGPVPEEFKDVQPTIRAACVKESGLTNEELVNKAALGEFTDDPQLKCYLKCIFDQFRLVSKRGINFDAMLALSPPSMKENAIKMVKECRDTK
GKEGDLCDLSFEVTKCLYNSNPETYFIL

>MmedOBP11

MKIFAVIFAICIVYAVAIGNLTEEERVELDRLANICVNETGFYEGHNSDDPAKNWISYGFKLQCYFSCMLKKMNIMNEDGTLNEEMIRKKIGDEVPADKIDAVITKCKDLKG
ANKCETATMIMKCYSDERLSLDPAEKSV

>MmedOBP12

MAIVRICNTTDPVDLRVLNDYLMNHNLNRLHIKSHHPLACFLLCVYSEFNWMDRHGGFKVHNIKAWMLRAELSENDTDILLRKCISLELTDPCTRAQYFTECFWTNHQDV

TVDHRHSLHSIMHKDVHQDKIYN
>MmedOBP13
MKIIAVIFAVCFAGALAELTVEQLAKLREHSTACITETLVDDANVDAAMHHNIWRMDDLKLRCYFFCLLKKLKVMNEDGKLNEEITRQRLANLFPADRIDGVIMKCKEM
KGADACETAILMAKCHADERGLLGPSPRSA
>MmedOBP14
MKGVKSIPLIAIAAVFCISINFFSTDAAFTVEQIESMMKPLGNNCVSKVGLSPELQEANRKKEFPEEKPFMCYLHCLARVTKVFDKNNQIDLEGTLKQVRLVMPDHLVEGSV
KAYTVCSRAAISEDPCEKAFQYAKCYYETDAPSYFYP
>MmedOBP15
MKNILLGICIFIPSVFCGTRPSFVSDDVIGFAASGVNACQRQTGVATADIEAVRNGQWPESRQLKCYMYCLWEQFGLIDEKGELSLNGMLTFFQRIPAYRVEVQKAIRECKSI
GEYLANGDNCQYAFTFNMCYAEVSPKTYYLF
>MmedOBP16
MKLFAVLFAVCFVGALAELTPEQLAKLHESRSTCITETGVEEGNVAKANDGEWLMDDLKLRCFFSCMLKKIKVLNEDGTFNEEKARKRIANDLPADKIDSVITKCKDLSG
GDVCETAMLMMKCYADEKALTKIITEKSS
>MmedOBP17
MKIFAVIFAVCFVAALAELTEEQKAKLREHRTACVTETGVDEANVDAAKQGDWKMDDLKLRCFFACMMKKIKVMNEDGTLNEEITRKRMANDLPADKIDGVMMKCK
DMKGADMCETAMMMMKCYADEKAFTKIITEKSS
>MmedOBP18
MRYSVFVFVGILFTFFISSDAESSGEKECPLKKAFQESIDACKDKLSEENLALLEKDENADNEDIRCFKACILNDSGVMSNGKIQIDKIEEAINAAIENVKEDEEKAKAIGESM
INGAKNCAGPAEEGENECEVAHRFITCLMEHAAEEKKKHNE
>MmedOBP19
MYRLAVVFIFASVVVLSESAITAEDLVKFGMARRTCDRTNRVDPSVIDRVLQGEMINDPQFDCHVACVLKELNLLTADGSLNVEVAASKVPENLPYYNQLVGAIRSCGSR
KGNDQCETAHMLFVCFHENNIPNLILG
>MmedOBP20
MQMQVNADIKRDCRKQTGVSWASLKKLKAADYNQNDPKLKCYLKCFMQKNGIFGEDDIDIEKALRHLPTGIKGPSKTTLEYCKKIPSVDSCDKAFQLAKCYFKAQPEVL
KSVSFV
>TjapOBP1
MQPAPVLSALAALLTFQALIVHAKRPEYITDEIMEMISNDKNRCMAEYGTTEALIDQVNDGHIPNDRAITCYMYCMFESFSLVDEDGEIEIEMLVGFIPEEFQEIAAELIEAC
ATLPGEDVCDKMYKRSSCVQAKRPDLWFMV
>TjapOBP2

MKLKVSGSIIFILSIYLLSVQCAKMNLDELKKMVKPISSSCKKKNNVPEDLLLASYAGVFPREKSLMCYYKCLATMLKLMNKQGQFSLDKMFNQVDLLVVEELAPRVKQI
AKDCYDQTPKRDDTCEYTYDLVVCAYNTDSSLSVFSR
>TjapOBP3
MRLSTTAMILSVFFISYIAVESKKMNIEELKKMSKPMMNSCQKKTGVKTEELEAAEKGTFPTGNKPLMCYFRCLAVMFKLTDKDGNISLHHLLHQIDLLVLDEIAAGVND
MLQYCFENTPKLEDSCEYIYELVICMHKRNTEMNFFEGSLLS
>TjapOBP4
MSRFALFLALFAMVVLASGHNIEKMKEAFKSCKTDVGVAEDTPMKDIPSSKMGCFHACVMKKFDTMKDGKIMVENLLERAEKRMNPLPAEMKEKLAKCADDANGKGD
ECEVAGYMHDCWWDLMKSMGVTPKGSSN
>TjapOBP5
MKLFLEFFILAVTAVCMITAGRPDFVTDEILEMVASDKARCMKEHGTTESMIDAVNEGNMVNDRAITCYMYCLFEAFSLVDEDGTLEVEMLVGFLPENIQASAETVISFCS
EQSADDVCDKMYDTAKCIADKRPDLWFMV
>TjapOBP6
MKFVVFACLMVLLVAQHHLPVQCKKMNIEELKGFSKPLTKSCKTKTGISEVILAQVAKREFADDPVLKCYFKCIAQMGKMMDKKGNLILENMLKQVELLIIEELSPRVKK
VFTQCFEEMTVKEPCQLAFDFIMCIERTDQELNIIV
>TjapOBP7
MQKIALFLAIFLVAYRVEAANEIPPEIKEMIAGIREKCHRETGVDIEHIDRTVEGYFHPSETLGCYFSCVFNSFDLLDHDGHLDFDKCLKRLEGVESFREHGTEMIEKCRHLS
GKNPCDSAFNLVQCFQQANPEKFFVI
>TjapOBP8
MKTALVFLAVCLAVTFASTLKDEQKAKLREFKEGCIKESGVDAAIVDGIVKGGPITRGEKIDCFSACMLKKIGIMTADGAIDVEAARGKVKTTNADPDKANKVIDACKDL
TGKDACETGGNVFSCFITKKDFPVLD
>TjapOBP9
MYLLGSLVLASCIFCVHANKVPDEIKNLVGGIREKCHRETGVDIEHVERTVEGYFHESEVLGCYFSCILNSFDLLDHDGHIDFEKMVVRLKGADSFREHGMEMVEACRGTT
GKNPCDSAFNVFQCFQKTNPSKYFVI

Supplementary Data S2. The amino acid sequences of 91 chemosensory proteins (CSPs) from 12 Hymenoptera species.

>BdioCSP1
MSRHGVYLALAIVALFLVGLVAAEDATVEPTEKATNSRVPTLEEVKNDEFYPLAWATIPYKPVVDNDRLFRKYKQCLLVPKPKGCPRDILEVRTLLPEALESLCAKCLPVHV
ERIKEIFEYVCKKRRADYDEVRKARDPDGSLQKKFEDKFGTINC
>BdioCSP2
MSDRRVFLELVVLVALLAVSFAADEDDVPLVKVVKPSELLDKNAMKDHYPIAWTQVDIKPIVENDRLFKKYKECLTTNHPVSCPRMVMEFKKLIPEIIETECAKCLPAHIEKL

KEAVEYVCHRRRDDYEQVRAEKDPGAVVQTKFEEKFGKVNC
>BdioCSP3
MSSRNGGGGGIKKINNHNEKQQSQTQLMALQARFQQKQLQEKEQKLLQLYDQQQQRAHQVAQRGSAGSNGSSASNNVGASAAKVKQLLASSDDRRQQNGFKGIDKSYP
LDPLKSKKTSPIAKPTKSNNNNNNN
>BdioCSP4
MKVSIICLVLMAAIVLVAARPDESYTSKFDNINVDEILHSDRLLNNYFKCLMDEGRCTAEGNELKRVLPDALATDCKKCTDKQREVIKKVIKFLVENKPELWDSLANKYDPD
KKYRVKFEEEAKKLGINV
>BdioCSP5
MAKFMYLAILVACLALGTFAEEEEERYSDKYDHIDVDAVLSNDRLRNQYYKCFLGTGPCVTQDAVFFKTRIPEAIVTRCRKCTEKQKDAFNKIAIWFDEHDRESWEAIMRK
AVSDYQKKGSFEESQGS
>BdioCSP6
MKSFLVLCAVVALVIAEEKYTTNNDDFDIEALIVNVPELKNFNTCFVNDTNCNDVSSDFKRNLPEAVREACAKCTDVQKHIFRRYLEGLQEKLPQQFEEFRKKFDPEGIYLEP
LKAALAKA
>BdioCSP7
MKVVLFVLLAIYAVNAQLYSDQYDYVDVDRILTDDILREQYYSCYMGIADCLTPDAQYFKEILPEAALTKCTKCTDKQKQNFEKIANWFTKNQPAKWDAYIKKAVEIYNQS
KAT
>BdioCSP8
MGRTSVQFVSTVVLVTFITGVKSSDNAIDKPTKKPVLRISDLFDQSKMVHHYPVIWTEIDYKPVVDNHRLFKKYKDCFLAENGFGCPRIATEFKSKYDTTFRLKIKHTM
>BdioCSP9
MKYTCIVALTLMFVVVVVRAQDVNMLLQNKNLVNREIGCVLQRNPCDVIGKQIKGLLPEALNNGCRRCTPLQAANGKKLMAFMKKNHPNEWAMIVQMYGRPRAVY
>BdioCSP10
MLHRKICLALTVVLAVLIVRASAEQLSLSDLNADKLFDQSKMKDHYPLAWNEIPFKAAIDNTRLFKKYKECVVSDNVRNCPRIITEIMI
>CvesCSP3
MLNRLTMQMLIIFIVSATIASLSSFVSAEVLNTSSMYSTKYDNIDINMVIKNDRLVNNYVACMLDEKPCTPDGEELKKNIPDALATECASCSPAQKNIAEVMYHHLIDNRPDL
WSKLEVKYDPSGSYRRRYLSLDKDDDDDDDDNNDEEETQSTTMKV
>CvesCSP9
MNDYLINILIVSSIVVPVLLTAKELKKFYQISSKPIAQTVLKRKKI
>CvesCSP6
MKLLVVLFLGLIGLSVVSSGSTNTTYVPSNVEILKAMENSRVRTPIFKCLMGTGTCAGTGGKVKKFMLEAMKNNCNTCSASDKKRAVNLMKEIRRRYPVDWASLVAKYDP

QGVYERKLLAQIENQS
>CvesCSP11
MFNCFIDGIKCTADARDLKEFFPEMLETCCKECSENLLRETKKMGDFLFEQKRDYGIRLLDKYDPNREHRNRCDAALKAKGIDLTRF
>CvesCSP10
MKFLIVFVAVAVVVVLGGDVYDSSKFDNVDMDAIMKNDRLLQNHFNCLIDGKGCTPEAEEIRKHIPEIMETCCAKCSDKQKEAGKKVTQFLIDNKPDMVKRM
>CvesCSP1
MNIKFFYLLSTLVGVFALVLNVNAKYEDVPSDIQKKYLPYIDCVLDRKSCDFIGGQVKKLLPDWFKNECADCNPMIKSEAKKRIPDIQKYFPEDWQLIEQKYGGKSNARQT
RPTFSAPSKPTIVIEEEKSRPKFSAPSKPTDRYNQPQKKLTISENSASPEVLFANDRSFGAKAVLCLVMKKNCDWQMKIFKKSMYIFANINTCPGCENPKVHKFFTQAKNLLE
RSFPHILQDIIDNKGE
>CvesCSP5
MKVVLILFAVIAVALSAKTTYTSKFDNIDVDNILNNKRLLEGYVKCLMGTGKCTSEGNELKEKLPEALATECEKCTPAQKEKSEKIIRFLVNNRRELWDQLAVKYDPKDEYR
QKYLDQAKAKGIDV
>CvesCSP4
MKYIAVGVFLAVFGLVVCRPDDGQYTSKYDNVDIDEILKSDRLINSYFKCLMTGDGCTPDGAELRKILPDAIETGCSKCSRKHKDGSKKIINHIIVNKPDLWKQLEAKYDPE
GTYKQKYKAELEKEGFTV
>CvesCSP7
MNMYFKVFILVILCAGIGFAEEKLYSDKYDHINVDAILRNPRQRNGYYNCFAETGPCVTADAKFFRDHFPEAIVTNCKKCTPKQLENFDKLTDWYTTHELEKYNALVELAL
KKFMKKN
>CvesCSP2
MDARAGINAPANHGIGLLVREMHPRLVGLFWCHCAVMHTSVILLLVVCASHCYQPPPAEAYMTRWDKLNLDLVLENKKLLHHYYNCLMNKGPCPPDGRELKRALPEALS
TECAKCSKSQKEGAIKIIKYLREYKPKEFGILANKYDPDGVYRRKYFASEDTNNLT
>CvesCSP8
MKTQIIFLCVLGVIALVSCAPHQSYANQREISSEEIMRVESQLPCVLNQGPCSEIGMKAKMVLPEILKTRKCLTCTSEENEKIGRILYIMHEKFPHHLIALNNIYGNKQQSTNFH
NFH
>SguaCSP1
MKSIIVVFTVFAIVFAQESTPEYYTGKWNDLNTHDIVDNARLFKKYKQCIMAETNTGCPQEVIELKKVLPEALETVCSKCSPVQVEKIRDTLKYVCEKRKTDFDDILKHIDPE
GTHRPKFEEKFGTLGC
>SspCSP1
MAFINFTILTIFSIILIAFAEKIELYSNKYDHIDADTILSNERLRNQYLNCYLGSGSCITPDARFLRDTF

PEALVTKCKRCTQKQHILFEKITLYFTEKEPDTWNKILLKAIENSRKRH

>SspCSP2

MNRVSFVALLALATIAFVCGQEEELYSDRYDYIDADDILKNERLRNQYYKCFYGSGPCVTADAKFFKNNF

GEAIVTKCKKCTQKQISNFDKMIVWYNENAPDQWEALVKKLVENAQENAN

>SspCSP3

MKEIIIFIAIIAVALAAEKYPSKYDDVDVDRILQNSRVLTNYIKCMLDEGSCTAEGRELKKTLPDALQSGCSKCNEKQKQTAEKVINHLRTKRPRDWDRLVAKYDPQGEYKK

RYENLTTKKV

>SspCSP4

MVNIISSILLFSFVFLQYSFAEEMYSDKYDNIAIEDILNNDKVREEYYNCFMDTGPCVTEDAAYFKGNFVEAMATQCKKCTQKQQENFEKVIVWYTENQPEKWQTLIQKAL

EDAKKLNIPMEATS

>SspCSP 5

MASTIKVVCVICVFVTLALADGSEERGGKSRVSDDQLNMALSDDRYLRRQLKCALGEAPCDPVGRRLKSLAPLVLRGSCPQCSPEETRQIKKVLSHIQRSFPKEWSKIVQQ

YAGV

>SspCSP6

MKVAIVVLAVISCALAQRYTTKYDNIDLDQILRSERLLNNYVNCLLDAGNCTPDGKELKKSLPDAASGCSKCSEKQKEGSEKVIRFLVNERPQVWDKLAKKYDPTGEYKL

KFQGQAQTHGIQI

>SspCSP7

MKSIIVIFTVFAIVFAQESTPEYYTGKWNDLNTHDIVDNARLFKKYKQCIMAETNTGCPQEVIELKKVLP

EALETVCAKCSPVQVEKIRDTLKYVCEKRKTDFDDILKHIDPEGTHRPKFEEKFGNLGC

>SspCSP8

MQFKSVILIVGLVIISISAEEQYTSRFDTINVDEILKSERLLNNYFKCLMDRGRCTPEASELKKVLPDAL

ETDCMKCTNLHKKMARKVIDYIVKNKNEMWQELVGKYDPKGTYRTKFEKDAVAAGINIQA

>SspCSP9

MAQHLLLVLLVVLAVARCVFTAPEDTTTDNKYTTKYDQVDVDAIVRNERLVNSYVGCLLDRNSCTPDAAELKKNLPDALQTACVSCSEAQKDVADKFSQFLIDHKPDQW

KLLEEKYDPDGEYKKRYLNDA

>SspCSP10

MEFGVILIFIISCGLSFGSDYYSDVYDKIDVDSILASDRLFNQYISCLLDKGPCTADGRSLRRIIPEAIS

TRCEKCNEKQKSTTKKVLVHLKEKKPDVW

>MpulCSP7

MTNHFIIYIHSSCVRIFIINLYDYFICFLLFFILDRFPEAVVTNCRKCTRRQSENFDKLTDWYTKHEPEK
YNAIVGMALKKLMKKS
>MpulCSP8
MYCPILLIILASLVITIHADELYSDKYDGLDIDGILANEELRKQHENCYMDRGPCDDAAEFFKSHFPEVVATACSKCTEWQSQAFDKIADWYNKNDEATWNAFVAKNMELA
KTMNIR
>AmelCSP1
MRHNYIVILILSLLTWTYAEELYSDKYDYVNIDEILANDRLRNQYYDCFIDAGSCLTPDSVFFKSHITEFQTQCKKCTEIQKQNLDKLAEWFTTNEPEKWNHFVEIMIKKKDE
GA
>AmelCSP2
MASAIKALLIVCALFIYTVTAETEEGQSGRSRVSDEQLNMALSDQRYLRRQLKCALGEAPCDPVGRRLKSLAPLVLRGACPQCSPEETRQIKKVLSHIQRTYPKEWSKIVQQ
YAGVS
>AmelCSP3
MKVSIICLVLMAAIVLVAARPDESYTSKFDNINVDEILHSDRLLNNYFKCLMDEGRCTAEGNELKRVLPDALATDCKKCTDKQREVIKKVIKFLVENKPELWDSLANKYDPD
KKYRVKFEEEAKKLGINV
>AmelCSP4
MKTILIALVPVCFLLGEVFSEDKYTTKYDNVDIDVVLNTERLLNAYVNCLLDQGPCTPDAAELKRNLPDALENECSPCSEKQKKIADKVVQFLIDNKPEIWVLLEAKYDPT
GAYKQHYLQNRVKEESY
>AmelCSP5
MKIKILLFFTILALINVKAQDDISKFLKDRPYVQKQLHCILDRGHCDVIGKKIKELLPEVLNNHCNRCTSRQIGIANTLIPFMQQNYPYEWQLILRRYKIMKYY
>AmelCSP6
MKIYILLFVLVTITCVIAEDYTTKYDDMDIDRILQNGRILTNYIKCMLDEGPCTNEGRELKKILPDALSTGCNKCNEKQKHTANKVVNYLKTKRPKDWERLSAKYDSTGEY
KKRYEHGLQFAKNN
>AconCSP1
MDGQLRALVFTMLVVALTPVSHSYQWPKPNTYMTRWDKVNLDEILESKRLLQHYFNCLMNKGPCPPDGQELKRALPEALKTACAKCSNSQREGAIKVIKYLREYEPKKF
GILANKYDPQGVYRHRYLESEYQSNST
>AconCSP2
MFRGTLLVLVVSLMLAAVYADDEKYSDKYDTLDIDAALADDATRNKYFNCFIGNGPCTEDAAYWKNNFPEAVVTKCAKCTDWQKTAFDKIAAWYAENDEQAWTALMEK
SIAEAKARNIPGAK
>AconCSP3

MKVISVLVLLAGLAIAAERPGENLNIDHVNYVLNNQRLLTNYIKCLLDERPCTGEVRELKKLLPELLKNGCNKCDTSKRAIAEKVVRHLQTKRKQEWATLLAKYDPKGEY
QKRYNSAQAPHA
>AconCSP4
MIFQSFILVIVGLSAIVAQELYSDKYDHINVDEILANSRLRESYLQCYLRSGPCVTADAKFFRDTFAEAVLTQCVKCTARQTEIFNKITDWYTKNEPEKYNMVIAKAVKFLAM
NNH
>AconCSP5
MTLIRIFFILGIFFCSALSQQSSSYISSDKLDELLKDERLLNFHLKCTLGTGPCDKVGHSLKPLIPLVLRGTCRRCSPQDVENIKKVIIFLQNKKPKELAKIYDKYGK
>AconCSP6
MGEGNCTPEGKELKKSLPDALATGCKSCSEKQKTGSEKVIRFLVNEVCHHFLLFIRYCHFHYRSGDVSVHYVFKSC
>CcunCSP1
MTPKRSLIVVATALLVLVAGVVRAEDKKYDSKYDNLDVEAILQNDAERNIYYACFMDTGPCPNEAAIFFKGHAPEAVVTSCRYCTQKQLEMFEKIVSWFVDNSPQEWNALI
EKTINDARKQGLSF
>CcunCSP2
MKVILFVFLAFYAVAAEQLYSDQYDYVDVSKILSDDALREEYYNCYMGTSPCLTADAQYFKEILPEAALTKCVKCTDKQKDNFQKIATWFTKNQPEKWDAYTKKAVEIYN
QSQKAPE
>CcunCSP3
MSRPEINLEFVVSITLLALCIARAADEDNVPLKKPIDMAELFDKNAMKDHYPIAWTEVNTKTIIDNDRLFKKYKECLTNEHPVSCPRMVMEFKKLIPEMIDTLCAKCLPIHIE
KFKEAVEYICHRRRAEYDQVRREKDPDGAIQKKFEEQFGKVNC
>CcunCSP6
MDKRMCWLALCWLLGGCLDKPINNEAIVNGYPWPEPGTYMTRWDKIDLNELFKSKRLMRHYFNCLVNKGPCPPDGRELKRALPEALENGCAKCSKSQLESAIKIIRYLRE
FEPVKFEILANKFDPKGIYRKRYLDPTPDE
TNNSITDENSVDENDQKLKRLIKR
>CcunCSP5
MLRRGVYHALAIATMFLAGVVVAQDTSEESTEITTELPFQKNNRTNEFYPISWTKYNYKFIVDNERLFRKYKQCLLVDKTTGCAHDVLQLKKIIPEVLESMCAKCLPVHVE
RFKEIVEYVCKKRRADYDEVRKAKDPAGLLQKKFEDKFGKVNC
>CcunCSP7
MKHTCAVVVLMLLLLLAIVASAQDVNILLQNKNLVSREIGCVLQRNPCDVIGKQIRGLLPEALNNGCGRCTPQQATNAKKLIAYMKKNYPNEWVMIAQMYGRAKAVY
>CcunCSP9
MKQLCTLVFCCVALLLAVNAAEYNSKYDNVDVDRILQNGRVLTNYIKCMLDEGNCTPDGRELKKTLPDALATGCIKCNEKQKATADKIINHLMKRRPADWEKLLRKYDPK

GEFKKRYEAQGRKI

>CcunCSP10

MATKLVFVLAICALAAVVCAKELYSDKYDNINIDAILANDSVRNEYYNCMLDFGPCVTPDAAYFKGLLGEIIITNCRKCTDKQRYMFKQVLKHYTLKEPQKWQELVLKVL
KELPKLK

>CcunCSP11

MHSYYSSRSTRRAYTLNGFYERLFLLEMFLRVCNLVYFIAGALPEALENGCAKCSKSQLESAIKIIRYLREFEPVKFEILANKFDPKGIYRKRYLDPTPDETNNSITDENSVDE
NDQKLKRLIKRHRSTIA

>Dallcsp1

MKSSVFFVLAILGAAFIAAEGGNRYADKYDSVNVDQLLGNERIYKQHLNCLLDQGQCSRQAQSLKDVLPEVLSTSCAKCSPVQRQMARKVVGYIQKNKPDDWKLLTTKF
DPQGRYTEEIRRFILSNV

>Dallcsp2

MLRGAVVIALVFLSAVIAEEKYSEKYDYVDVDGILANDKQRESYYKCFAGIGPCKTAAARFFRDTLPEAIVTRCKKCTARQSVNFDKISDWYTTNEPEKYQIIVAKAVRDIM
AKSA

>Dallcsp3

MRRLIFFVLIAVALAEERPMYTTKYDKFDIDSIIKNDRLFKNYIDCLMDEKPCTPEGNEFKRNLPDALETGCASCSKAQKTMAEKFYHHVIDNRIDDWMRLENKYDPRGNY
RKNYLGLDIETTTVAL

>Dallcsp4

MKIAVFVLLSCLVAVISARPDKYTTKWDNIDVDQILNNDRILNNYVNCLLEEGNCTAEGRELKSVLPDALETECEKCSRKQRDGSKKIIKFLVQNKQDLWEKLMDKYDEEK
KYRGKYEDQARAEGIEIQS

>Dallcsp5

MKVAFVLLAVVAVSLAKPQGYTTKYDNVDLDQILRNDRLLNNYVKCLLDEGHCTSDGKELKASLPDALATGCTKCSEKQRAGSEKVIRYLVNERPKVWQKLAAKYDPHD
EYRVKFQGEASARGIQV

>Dallcsp6

MRAVVILCLLIGSVIAQKAGKYDNVDVDAILKNNRVLTQYIKCMLGEGSCTAEGRELKKVLPDALKTNCAKCDEKQKSTAEKVINHLRSNRPNEWNRLVAKYDPQGEYEK
RFEAAASAKN

>Dallcsp7

MFTKVLLISLLMCAAVMGQEAEQRSRVSDEQVNIALNDPRYLKRQIKCALGEAPCDPVGRRLKSLAPLVLRGSCPQCSREETHQIKRVLSHIQRQFPREWSKVIKQYAGV

>Dallcsp8

MEAKPSTQVLVFFLFTVVILTRDIECYTWPRRNTYMTRWDKVNLDEILQNKRLLHHYFRCLMGVGPCPPDGQELKRVLPEALETACAKCSKSQKEGAIYVIKYLREYMPK

KLEMLANRYDPDGKYRRRYYHSTSVDNNTT

>Dallcsp9

MKARMALFLVGMLSLVVGIEAQDVEALLKNPEFVNFEINCMLDEGPCDLIGNSIKNVLPEALNNNCRRCTRSQARIIRRLIDFMETAYPEQNQRIRNRYIRSPTSELADELP

>EforCSP1

MKFAVAVVLCALASVTLAAEMPTIPSKYDSINVDMILKNDRIFRNYMKCVLENKSCSPEGRDLRMYLPDA

LRTRCSNCTPKQKATAQKIIKFMMEKKKDDWKKLLEAFDKTGEIEKSFKESGGIL

>EforCSP2

MKLFAVVLLTLASFALAQQEEEKYDDKYDYLDVDGILKNDRLRQQYLDCFLETKPCVTADAIFIKKNFPE

AVVTKCRKCTEAQKMGFEKLINWMTKNDPETWRAILRKSIEDFTKKGNERRAKEKGERLRNSA

>EforCSP3

MTRICLLVAFLVAGILADEEARRGLYSSEFDNLDVEAILNDDAERDKYYACLMDTGPCHSEAAVFFKDLVPEVVVTSCKYCTPRQLEIFGKIVTWYIDNKSKEWKELVVKTI

EDARKRGLLDY

>EforCSP4

MKSYAIVLIMVVMITKTESQNVQMLLQNRQLVEREISCVLNRGPCDIIGNVIKSTLPEALNNNCRNCTPQQAQASQQIIAFMRAYYPRESQEILQLYGRRGK

>EforCSP5

MNAVLMILGLLAGMALGQDQYTTKYDNIDVEAIIKNERLLKNYVGCLLDNNPCTPEGTELKKNLPDALETNCKSCSDIQKRISDRLTHFLIDNRPDDWALLEQKYDPTGSY

KKQYLG

>EforCSP6

MTSYKTQFALLMVVFLATTYVQAQNIDVMLRNRQLVQRQIKCVLKKAPCDAIGKQIVAQLPEALYNDCRRCKPQDAQNSRKLLAFMQKNYPNELQQMYIIYKPQPH

>EforCSP7

MDKRMCWLALCWWLGGCSNLDGGNDKAGLSTAGTADGYLWPKPNTYTTRWDKVNLDEILGSKRLLQHYFNCLVNKGPCPPDGRELKRECWFRVL

>EforCSP8

MKSTIALVLCLVLVVAGEEQQYTSKFDHIDVDRVLKNDRLLRPYLNCLLKDMQCTPEARELKRLLPDALATKCEKCTAKQKEGSEKVIAFLSKNKPEEWEQVLEMYDKDH

IYRTKYAAEAKARGIQV

>EforCSP9

MSHKYLIALCFVVLVQAFAFAEEEEKYSDKYDDIDLDEVLKNDRLREQYFKCFMDEGPCNTGVIKFFKEK

FPEALATQCKKCTEKQKAGFEKLITYYSEKEPENYQKVLEKLLNKKSA

>EforCSP10

MLKILSLAFLTMVFGVFVIDATYSTADPEIDFYIHNPKLIRKYLDCVEKRSTSTCGIIARRISRLIPEAL

FNQCRACTPDEAAKAHKIIQFVRTYYPYDFNLIWRMYYPGAPSGQY

>MpulCSP1

MYCPILLIILASLVITIHADELYSDKYDGLDIDGILANEELRKQHENCYMDRGPCDDAAEFFKSHFPEVVATACSKCTEWQSQAFDKIADWYNKNDEATWNAFVANNMELA
KTMNIR

>MpulCSP2

MSERVRRIRNLQTIAIGINVAVAQSRSISFSTSSQYNTRVNMKVAVVFIIAVVAVVMGAKYTTKYDNVDLDQILKSNRLLNNYVDCLLSKRTCTPDGKELKENLPDALKTECA
GCSEKQKAGSEKVIRYIVNQRPDLWEQLAKVYDPNNEYRVKFQDKAEEKGIKI

>MpulCSP3

MTNHFIIYIHSSIVRIFIILLYDYFICFLLFFILDRFPEAVVTNCRKCTRRQSENFDKLTDWYTKHEPEK
YNAIVGMALKKLMKKS

>MpulCSP4

MSRKIVELLCSLIIIHINVISAVDHVHFSPEILLRNERLLARFVDCLVDEGPCLGPIAKFKKEIPKMMET
QCARCQEQIKVLSAKVMQHIREHKPEKWKKIQEIYDPDHKHQEKLDGLLDDYQ

>MpulCSP5

MALLIKIFLLSVIACATHGQESTGRGISQFHISDEQINMALNDRRYLRRQIQCVLGEAPCDPVGRRLKSLAPLVLRGSCPQCSKDESRQIKKVLAHVQRTFPKEEWTKIIKQYA
GVLSKN

>MpulCSP6

MRNQTVIFVALVSILSMIINTSNARDFDDLQRDGTYMIQVIDCVLGTAACDEYGEKVKALIPEALNNNCKRCNPEQKRKFKLMSAFMKMAYPDQWQQIQEKYYSNRYIHA
DY

>MmedCSP1

MKVAIIFLAIIAVALAATTKTYTSKFDDVDVDGILGSDRLLRNYVNCLLDRGPCTKEGVTLKEILPDALATSCESCTEKQKTKSEKVIRHLVNNKKELWDELAVKYDPNNEY
RKKYEDQAKAKGINV

>MmedCSP2

MKMFIVLMLAAVTVASVSSFVSAEAVNNKGMYSTKYDNIDINAIIKNERLLNNYVGCLMDEKPCTPDGAELKKNLPDALASECASCSPAQKNIANVMYHHLIDNRPDLWS
KLETKYDPSGGYRKRYLNQDHDQDQNEGNEEEIKSTTMAI

>MmedCSP3

MKYLGFLVIAVIFSAVSCDELYSDKYDNLNVDEALANAEVRQTYFNCFMDKGPCGEDATYWKGNFPEAIATNCKKCTEWQKEAFDKIADWYTVHEPDNWNSFVDKMVQ
GARNFGDSRK

>TdenCSP1

MKAVCVMLFCCMSVALAANAYTNKYDNVNVDQILNSPRLLQSYMKCMLDEGNCTPDGRELKRTLPDALATGCTKCNEKQKAVAGKVINFLMTKKANDWARLLAKYDP
NGEFERRYRAQGNRIFAN
>TdenCSP2
MSKGPCPPDGRELKRVLPEALATGCAKCTKKQIEAAVKVIKYFREFEPERFELLANVYDPHGIYRRKYFDNSLDNDVITSNSLTGERNRRMAAVAAAAAGLHSDNHPRKRLI
GQ
>TdenCSP3
MANRFVLALVLCTLVAGCLADEEKYSDKYDYIDVDNILGNERIRNQYFNCFLDYAPCLTADAKFFRDHFPEALVTKCKKCTEKQKEAFEKLVLYYTEKEPEKWRAALTKAI
AESGKKRAKN
>TdenCSP4
MAGLRYLIACLAVLSTIATTSALDPNAFLRMDAVLADPALFQTYLSCFVDEGECPPDGQMLKKLLPELIATRCNKCTENERRTACNAIMMLEQPQYAREWQKLQQIYDPRG
VNYAYLNRFKSACASVDYA
>TdenCSP5
MKATLAMVLCMAVVAVVMGADEVKYTTRFDSIDIEQVLHNDRILKSYINCLLKDAACSPDARELKRLLPDALATNCEKCSEKQKAGAEKVISFLAKNKPETWTEILAKYD
KDNVYRTKYAEQAKKLGVPV
>TdenCSP6
MKALLSYVAVFGLLLLLLLTTSGAEESKYTTKYDDLDIDSIIKNERLLNSYVGCLLDRKPCTPDATELKANLPDALANECSRCSERQRQISDKLSHHLIDHRPEDWQQLEAK
YDPTGTYRRVYLSDRQRLKAAVATTDNTDVGPAAASGA
>TdenCSP7
MERKSVLSIILVVFICAVLTRADEDSREWILYPTTHDDFDINALLSSDQDRNSWYDCFMHKSDCPSDTAKFFKERLSEAVMTSCKFCTEKQLQIFQKFVLWYAQNDPTAYGV
LLHQMMAEAEHKGIDTIM
Supplementary Data S3. The amino acid sequences of 29 Niemann-pick typeC2 proteins(NPC2s) from 11 Hymenoptera species.
>BdioNPC2a
MNKAILILLALVAVQTSATPVNICESDIVDTSSVTIENCDKPPCVVKMGQTVGFSLKFTPSWPIRNLTVHIEASNWLLTIPIFDDSICNKIFLARNNSLFPCPLCPQIEYIYRDEIR
APELPFTNSLEGTVKLTLETNSESFKCFKASVCIRPRLYRMMHEYLHRCSEITNQFTGMLN
>BdioNPC2b
MKSTIAVAALLSILTVVRGTTVSQCGDIPLPDDVQIENCDQPPCVFYINTTSSMTMTFKTPRKLEHIAPSAVAHVMSIDVSYPLDQDDACDGIVNTECPLDDNQVVEYTYSMF
ILPIFPEITLTLEFALVDKDQDNEPFECFQVELALKSQP
>BdioNPC2c
MLCKVLALLLFAACVCAYEDCGSKDGSIVSVTISNCSNDKKCILKRNTNASIEIGFTTNDDSDVLTAVVHGVVLGVPFPFNLPNPDGCKDSGVSCPISAGQTYNYKTSLPVLA

SYPRVTVDVKWELQDKDGNDVVCVLIPSQIK

>TpreNPC2a

MKFICGSIVACLLVATVAATPFTNCQEGAPPSALRVEGCDKAPCKLRRGTNVSAEWDFTVNADTKHLTPRVRATTLGMTVNFPFPQKDACETLIGTSCPLIAGDNVTYKLTM
PVARYYPQVSISIEFAFLDDAGQVAVCFKLAAKTA

>TpreNPC2b

MTKAIVTLAVCALLVALASATRVNECKSGKAIEDTTKVQITNCETPPCKLKKRTKVQIVQRFTPNRDVQSVKTAVYATILGVPLPFVGVDGTDACGKVFNAADGSPAPCPLK
KGVEYLYKNEFPILPIYPTVQLVVHWALEEPDKKVVACFEVPSRITV

>TpreNPC2c

MLRLALSILCAVCVGAVEFRDCGSTAGSFDRVEVAKCDASSHSCVLRKGHDGSLDLHFTPSKRCEKVTAIVHGIVDELDVPFPLPNAEACQDPQSKVGCPLEPGVASNYHAT
LPVLHSYPTVSVKVRWELKDENNDNIVCVEFPVSIR

>NvitNPC2a

MKFIAGFIIAAFLVASSTQSTPFKTCKDGAAPKDVRVAGCTKNPCSFIRGRNVSAEWDFSIPEDTKALKPRVRATVLGATVNYPFPQKDACNTLTNAKCPLETGEDVTYQLSM
PILKSYPKIPLTVEFAFLDDKDKVVVCFSLSAKVADK

>NvitNPC2b

MEQIRGNMKIITRFIICASLVVLSSQSTPVEKCKDSPTPKDVRIDGCTETPCSLVRGTTVTAEWDFSILE
DTRALKPRVRATVLGMTVNYPFPQKDACSTLTNAKCPLEAGEDVTYQLSMPISKMYPKISLTIEFAFLDDKDNVVTCFAVPAKVTSK

>NvitNPC2c

MMRSFALVALFCAATRAVEFKDCGSTAGNFDKVEISNCDLSAPACVLKKGTDASIGLHFTPNKASSRVTAVVHGIIEDIDVPFPIPNAEACFDNHSGVVCPLPAGTGTSYHAN
LPVLAKYPTVSVTVKWELKDEKGANIVCVEIPVSIQ

>NvitNPC2d

MAKTALFLIAVAGLIALTVATRVNECGSGKALPDTTQVQITNCDAPPCKLKRRTKVAIEQKFTPSQDVQSLKTSVHATILGIPLPFIGVDGTDACGKIFNVADNTPATCPLKQGT
EYIYRNEFPVLPVYPTVALTVYWALKENEKPIACFEVPSRII

>MrotNPC2a

MLRETILVFAALLVLAAATEVNKCGTGEKLEDPNQAKITGCDVPPCKLKRRTKYTIENKIVPDRDVDNLVNSVNAAILGVPLPFVGVDGTSACDSMYNVDGTPAGCSLKKG
VEYIYKREFPILQIYPTVSMVIHYELLDGNHSIACFEVPAKITN

>MrotNPC2b

MNRNTEFLLVFLFLALSVACAERIPWRTCPYPDSNTPTNCTIHEVYLDPCAEAKEEKPCKVKRGITANMTFHYTPEFTSDKMNVRIFWASQVMDIPFLGMDPNACLSTTCPV
VAGQRNVYHADIPILKKYPVRTYDIKWKIWNEEEQECCFMFQIKIVK

>MrotNPC2c

MAYLTYAFILVAFFVADSMQSNFQTCSGQPAPKSVVISGCNSLPCNFKRGTNVEARITFGVTQNTKSLKPDVDVELGKIHVKYPLPQQNACMDLESSRCPLEKGEVAVYKLK
MPIEKSYPKVSLTIQLSLLDERSQSQACIRIPAKVVD

>McinNPC2

MTSAKIFILWVLGVWVMIINGYEDCTNLKDISNPNGTSLGTLQGFNVSDCDVNKVCIIDHNSTTTLSMDFTLNERVEVVNTKVEASLGFISLELKIGHPAPCNDTNSGLECPL
EPSDTAYHYTVTDQIKFRIRNQKINVKWSLLNEDAKTIVCAQVAVMFR

>AmelNPC2a

MRGTILVFVALLVVVCATEVNHCGTGEKFEDPNQVKITGCDVPPCKLKRRTKASVEQKFVPKQDVESLVNSVNAAVLGVPLPFVGVDGTSACDNIYNLDGTPAGCSLKKDI
EYIYKREFPVLQIYPTISMVIHYALMEGNNTIACFEVPAKITN

>AmelNPC2b

MNRNGMFLFAFFLFVLSTVYAELVPWRQCPYPDPNTQTNCTIHEVYIDPCKEITEGKPCKIKRGVIGNMTFHYTPAFSSDKVQGRIFWASQVMDIPFLGMNPDACLSTSCPIE
AGSRNTYHVEIPILKKYPVRTYDLKWKIWNDEEQECCFMFQIKITK

>AmelNPC2c

MYRMIVIILIVCLCFSPMYRAINIDDCGSKVGKLTSITLDCDMTKSVCDLIPDTNATIRIDFTLEKDVSK
VNAIVHGIVMDIPIPFPLPNADACQTPDSGITCPLNKGETYHYKNTLPVHKSYPKVSVTVKWQLKDENNEDIICVLIPARIK

>AmelNPC2d

MAILTYVYVFAVLFIANSMQAYVPCDGKPAPTNVKILGCDTLPCNLVRGTNVEANVDFKAVANTKTLRPVVDVDLGNSHMQYPLPEQNACKNLVNGQCPLQSGQAATYYL
KMPVLKAYPKVALTIQLSLVDENNNSEVCFKIPAKVVD

>MmedNPC2a

MYRIILGLVLCLSVSTYAVIQDCGSKVGKYHNVTVSDCPSDAATCILKRGTDATITISFDVDKEVSQVKAVVHGIIGGAPLPFPFSHPDGCQTSGLTCPLTKDNGPYQYSTSLH
VEKLYPKLKVGVKWELKDENEDTIVCALIPSEIK

>MmedNPC2b

MLRNIFPVVLSLLCLTFLTEAVSYDQCKDAKDNHSIRLLTISNCESAPCPLKRQTSVMVEEIFVAKKNVKSLTTSVFAKVAAFWLPFVSVHNKNACDNIYNMDNSIAGCPLKA
GTEYKYRNEFPILSIYPTLTLPVQWAKDKNEIITCFQVYVKITN

>DallNPC2a

MCRTICALVVCLSALASAAEFKDCGSSVGKYSSISISDCGASDSECILTKGTNATIEISFSTVEPVNAVK
AVVHGILTGVPIPFPISHPDACANPDTGITCPLKKDQAYTYRKTFPILAQYPKVKVEVKWELQNEKRQDIVCILIPAKIQEKKSAN

>DallNPC2b

MKNFLVILGVACVALVGADVVQFQPCSYREPTQTNCTVHELRINPCAEAAEGKPCRVKRGTDASIEFDYTTNFAADTLQGRAYWANKLMDVPFLGMETNACLSTPCPLKA
DTKQTYKMALHVDTKYPARSFDVKWKLWNQQEQECCIVFQIKLTK

>DallNPC2c
MLGLKVLVIVGFCVAASLQDTPFTKCKSGGPAPEGLRVKGCDKTPCKFAKGTNVEAEWDFNVVADTDDLKPIVKARALGITVNYPLPEQDACKSLTNGECPLEKGELISYG
LKMPILKAYPNVDLHLTFSLVDQKENVHVCFELDARVVN
>DallNPC2d
MFGKTALVFTTVLCFVAFGESTDVIECKNGRNLDNIKSITITNCEEPPCELKRGTRVELVQKFVPNEDVDRLTTSVSATILGVRLPFIGVDGSDACQNVYDADGKVGCPLKAG
TEYYYKNGFPVLEIYPKVKLVVSWALLGSSNPITCFEIPSKITS
>FariNPC2a
MYPIICALLVCLSVLTSAADFRDCGSQVGKYTSISISDCEDSDSECILTKGTNVTIKITFNTDEPVNVVK
AVVHGILTGVPIPFPIAQPNACNNPETGITCPLKKGGPFTYTKTFPILSQYPKVKVEVKWELQNEKNQDIVCIMIPARIE
>FariNPC2b
MLGLRVLIIAAFCVVGSLQDTPFTKCKAGGPAPEALRVKGCNTTPCRFVKGKDVEAEWDFNVVADAEALKPVVKAKALGITVDYPLPEQDACKSLVNGECPLEKGELVSY
GLRMPILKAYPKVDLHLTFFLVDPQKNVHVCFEIDAKVVDN
>CchiNPC2
ADKMYRITLGLVLYLTVSANAAFQNCGSKIGSLNNVTVSNCDPDASICILNRGKNVSIEINFNLDKDIKRVSSVVHGIIAGATLPFPIAHPDACVASGLKCPMTKDGGPYKYK
TNLAVETFFPKIKVDVKWELKDETATNIVCVLIPSQLK
>MdemNPC2a
MYRIILGLVLCLSVSTYAVIQDCGSKVGKYNNVTVSDCEPDAATCILKRGTDASITISFDVDKDISAVA
VVHGVIGGAPLPFPFSHPDGCQTSGLTCPLTKASGPYTYTTTLHVEKLYPKLKVGVKWELKDENDDNIVCAMIPSEIK
>MdemNPC2b
MFAKAFIIASFCVAASLQWSQFKSCGDHTQDPHRLVIEGCTTFPCIVQTGHNKAGQWDFIATTKTSVLKPQAELAIGGLWYVPYPLGIDDGCPYLISKKQCPLEAGDQVTYF
FSRAIPRVPSGVIVWARFTLKDENYNTHCCFEVEFKAA
Supplementary Data S4. The amino acid sequences of 705 odorant receptors (ORs), from 4 Hymenoptera species.
>BdioOrco
MMKMKQVGLVADLMPNIRITQAVGHWLFNYYSEGMRFPHKIYCMVTLFLMLFQYGTMAINLVKEADDVDQLTANTITVLFFLHPIVKVIYLAARAKIFYKCLGIWN
NPNSHPLFAESNQRYHTLAVTKMRRLLFSVCGAVAFSVICWTGITFMEDPIRTIHDKETNETTIVPIPRLMIRSAYPYNAMSGAAHIFSLIFQFYYLVITMGICNMFDVLF
CSFLLFACEQLQHLKAIMKPLMELSATLDTVVPNSGELFKAGSADHLRESSGIQPSSNGENVLDVDVRGIYSNRQDFTATFRPTAGTTFNGGVGPNGLTKKQEMLVRS
AIKYWVERHKHVVRLVTVVGDAYGVALLLHMLTTTITLTLLAYQATKVNGVNVYAGTTIGYLLYTLGQVFLFCIFGNRLIEESSSVMEAAYSCHWYDGSEEAKTFVQIV
CQQCQKAMSISGAKFFTVSLDLFASVLGAVVTYFMVLVQLK
>BdioOR1

MQIAVISCRSRDVLFLMQIDAILHFSRDDHSRKCQYLARTNREMASTNDNSASQRQIMQTKGCSSDGSNGLNVVGMDFFDSRYFTVNKVLLRLMGLWPYESALKKM
TTRILVTIFAALSVIPQFICLSSSGSNFHKAIQALATLLYFIGLWLKYILYVISESRNKILFRNIAENWKLLETDEERNILLKYSETGRFLSIGYVAYMAAALVLFVTLPAIPPVL
DKYLPLANGSRPKIFLLDGEYFDDKLENYRAIYIFEMISSTLPPFLLVPIDSTYALTVEQCVGLLAVVKLRLKLITKRAKQATSGNYQTKDVAYEKLVKAAELHQKVLHF
AQVVQTSYSTAFLVVMGVNVILLSAGSVVIIMNLDVPIEFFRYTLIYIGLMIHMFYLNWPGQKMIDSSGGIFYDTYNNEWYKCSLKCQKLLKFMMLQSIEPLELTCGGY
YTMNFQNYGSLTKTSLSYITVFASFR
>BdioOR2
MHEHCRSLKRFAVAVVGIPRKKSSQSHILARVETTAMNLAKGFDVEIGLTRRLLRSTGIYPDPNGFQSVASRGYFLIPVIFMLYFCNIPQSVTLKKIWGDVSAVIDLLTTTS
IPFGIAIFKHVGLRYNADVLSQLVLSMSDDWKSSKTKKESEVMWKNAKISRIISITIFSLATGSTITFFLMSLITSHNEIKRQIENNVTNIIRPLYMSGDFFYNVQRSPHFEIT
YVFQIITMLYAGTTFSGIDAFFAVLMLHLCGQLSNLKENLRCLADSNAANGEPSSVILYRLITRHQHLARFAESVEDTFNVMFLPQLLTSSLVLVFQAYQLVTTVTSGDL
QLSELLHRIYFTLCFTFSLFVYCYIAENLRNESMEVGDAVYQCEWHNLSGAEMKAIMMIILRARRAFIITAGKIVEFSLETYCSILKSSAGYVSVLLAMKDRLN
>BdioOR3
MRLPEVIWPSRDILMTFGLWPMHPNATLTRRLFANLRFIVGLSGVISLIVPELMTVAAHWGDIDVLEDYGALALVHILVASKIVYIFLRRRSFRSMYQYLMDFWDISDDP
REREHFERLAKLAKLASIIFTVATVSNMLSFEATPLVNWMRNRREINEGLRNRPLLLELEYPFLKFHTPLKNFFFSLCRYVNLKNYKIFLISFDSYPFDAHRTPNYELVFI
WQSFGGCCAVLSVSGFDCTVMVFIVHVSAQFRLIAARFRAITRKISEQTTVELDHVLRAEIGKCIAQHQHTLRVADDLNSLLSPIMFAQIMTSGMQICLSGYGVIEGDSS
AVRIIKFFVLLTSLLIELIIWCVPAEILIQESGAIADTIYYDVPWFKLTPSLQRDLGFVMLRAQKACEVNALYRVMCICTITEVVNAAYSYMALLSGMST
>BdioOR4
MSAVVAFESFTDVSHDPPKRNDNAHFVPMAIQETYDSIVNWNRVLLAMIGLWPDENGEYNVFSKFRFFAATFLIFYGGTGPQTAELFLFCRDIDSVMENLTNSNFAM
ANSLVKILVWWRCTTDLKPIYNCIRADWQKPKTDMEYKEMLDVARLGRSMTKFLFVMAQGNVAICCTLNIFMHVTGLFPTRFLYFPRSYYPYNTSVTPIYQITWLSQF
VAGFTVATTYIAVDGFLGFLVLHICGQMAILKNELKYLTTPIKREETNALGLIREKLRYVTRRHAHLISLVEIIENDFNVMFLFQVLSLSIQICFQLYQGLKAMASEEGASF
FQVFFMCYFTFCVVLLFYVYCYVDEQLTVQTSGIRIAAYECEWYNLPIDEIKALTFIIFRTEKPPCITAGKFCSFTMVTFGEVMAKNIYLFLLVLRT
>BdioOR5
MTTRVSPKLPQSSSQPYFNSDYIKDTEYVVRVAKTLLTPIGIWPRDGDNTPMSNLVFRIRVDVIFCLMLFLLTPHFVWTWFVADNLRKLMKIIAAQVFSSLAVLKFWTLII
NKKDIRHCLDVMENDYQEVESEDDRQIMIKNAKIGRFFTVAYLGLSYGGALPYHIIMPLLEPRVIRSDNTTMIPLPYPSEYVFFVVEFAPLYPIVFVTQIFISALILSINTGV
YSLIACVVMHSCCLFEVTGNKIDKWLKNRTPIAGQPLDHALTTRLRKIVDFHVQAILYAETMENALTIVMLAEMGGCTLIICLLEYGILLDMEDGDWLGCTTYAMLM
TSIFVNVFILSFVGDKIKEQSELIGFSAYSIDWLELPKEVILKDLKFIMARANQPTRLTAGKLFDLSLQGFCDVAKTSMAYLNFLRTLEIT
>BdioOR6
MSSVQVDRREGDSFFLLSNENLFSLLRQKKKRSADFDWALGLNRLGLDFMGVWPRERQPDTFARMARLPLMVVWIVVSLILPEIYALMQYYRDLQLAADNLWNTLI
ASASVVKLSLLWYRREVLQQAIRVSSIDWLSDKSSRERDLMLRQASRARLFTLAGYATMFGCVFGFLVAPLFGLSIRLVNNITDSPSRHLPQPTYYPYDVTISPYFELTYVT
QLIGTALLGLTLTVPEGCFVALLFHAAAQCEILAERCRGLALPHPRRELRAMVDMHVRLIRFVDAIEMSFNMCVLSTVIALAIITACIAFIILHIIGSEQESLQVIQLIVLIS

GVSIFMISMFVYCLASEILAENSTKLADAAYYSSCKLVSHASAKDFFIIMMRSQIPLHMSAGKFFYLSLNAYLQFVKTTGAYVSMMLTIAQ

>BdioOR7

MEETSSFYRRVRRVQTRVLRFAGLIPLEGRTHYFLGTIIMSSYVNFAFAAVSSVYIWAFVEDCINKRFNPDITSELFSFVGFHFRFMYIFGRRKKLTRMLGYVDDLWKGVS
SEEKIHVRMFVRKVSRLSCCYSGIILTTITLYVLSSQLPQLTAETTNETIHRVLPYPFYFDVQSTPRYEILLGIQIVCLLTVTQTSVCVDTSIAFLIMIACGHFRLIQVRLNAIA
RDIEDNEEGRRRPMATVKEEIRDNDEKVGNLDFERSDERIRKRVRDCVAYHSEILEFCNDIFKLSSEIFMIELISTTYNLSLIGILLAGNMPLAEKFKFAPVLLILTTQLFVC
QYPPDLLLQESVGVADAAYIIPPFRKDRLRIDRLLLTLLTRSQIPYQLLAGGQIPLSIESFGNMIRGAVSFFTVLRNFN

>BdioOR8

MEQLHFYLFRRKDRVRRIKPNSKIAVSYFQNKRWSLIPLLEELCGFSPYQRKVKQYSLQLFYSLLASTILFAVSNTLTKDVGNKFGMAVECVVFIFIFIAIVVKLVLASIHQ
RSRQMVYKKIAEHYEKMKEPEEAAVLGKSAEDGFKYTLRYMYVVSVVVSIFVSVPVVRSFYDFCFTHNEFKIPIPIPMNYGFDHHKYWYIIALHFITWMFSCLLIQFTY
DLSFILQVRHTAGLFKIVVIRLRRASDLVDENENIKMSDDTADNSYSLISSAIDLHAECFEIVRLIEQSYTTSWFVILLLSTSGMGAGIYLVSTKFYDPIETLRFALVFMTSLL
HFQMVFSQGQIIIDSAESVSDACCACKWYGLPNKSCKLLSMMMLRTSMPCILTGAKLFPLSMESFSKMLKASLSYYTVFKARS

>BdioOR9

MDIFDNSYFRFNKMMLVALGLWPFNTQILQSCLQLFWTLVLVGVAAPIMNAIVVHMPDVDRIMEDTYNLLYAIQLFLKYLSLCYVQKEMKSLYTVIANNWLLIVDEE
EKKILNEYSKRGRYLSVFYTSMLAITWIFNMSIPLVPEILNVVYPLEVPRPHLYLLKSEFSVNKDDHFYAIYFWDCFTIFCVVIIFGGVDSMYMVTVEQGTALYAIITHRLS
KIRDPFIIDLQGEKLNSYDWLIHTIKLHQTVYRLRNLAILTYHFSRTFCYKLQNRCHSHLYSYVTEIRDSYSVTFFCLSLINLVHLPLNCVVLMVYSHDRVLTIRYTFILGC
LLIHIFALFWPGQKLIDHSTRLLHDAYCSDWYEAPKKTKILLQFFMLRCSKPFVMTAGGLMEMNFEVFLKILKTAASYVTVLASYR

>BdioOR10

MAENSCRLEMQSRKRSNPAMDPCVKRIFEGRYFSINRKILMCLGLWPYGIKYKIYIIRIVIISTLMLLSVPHLIDLILQTEDRDRQMMGILAFLTLWLMILKMVGMISAEE
KMREMHRMIASNWQLIEDENERDILHTYAERARTVVIGYSAGLLIASAFACMIPSIPKVLDRIMPLENPRPEIVLIQGEYFIDKKPYFTLFFFFNYFTITCVIIVTIPFETMF
VVSVEHCCSLYAVVRSRLLMMRRITQKNSSATEVDDAYEWLVQAIQLHKRALTFTTLLNDSYSTSFLFHLGISIFVFPLACCAMISQLHNILNCIPYLMYIAAVLIQGYIFF
KPGQRTIDHQDDIYDAAYCNKWYETSLRTKKLLVIFMIRCSKPATITAAGLLEMNFELYLRMLKAVASYSTVAASFR

>BdioOR11

METNELPKEIIFEEYVQINSRMLRCMGLTVNVRGNRRDARTRILESLPTIITNIICIVDAIFQLQWIIDLWSTNKDLVTQITTSGISNIVCICKGLRMAYCRETLQSLFEKLD
LMWTNTRIPNEIREAVLSGAQNTLTFCKWYISMMIGLGICFALPPLVHVCSQYLSRSTANHTHDFSQRVFLVRYPFEVNSLATYLAVLLEEEYILFYSALYWVCCDTLFA
QLTTHTSLLFEVLRYNIETATNNEKDDDRLKKNVIGFIKRHQKLTRICQQIEKIFSPVLFTTMLLTSINICVNLFELRAMIIEGKFDEALLHSFHLGNIFCQLLVYCIFAERL
TQQTVTISNATYNFKWTEKHYSLRVYLQTLIMKSQKPFYCTAYGFFTIGHNRITACFQIVNRALSFYMMLQTTT

>BdioOR12

MSITDNNNLSKNEDIEQFLNMNLKLLSCLGIKIYLNGSQVIEKRKLLDKIPTLITIGFQVYYAITQILLITEAMQNHQYMIATEVGSYLFSNMMISSKGCRLIIAVTEVDSL
LKELASVWRQNVPGHKIQNKILHKADRARFYCKIFIVFTIGGIFIYCLPILIHFVSLYKHRNTANYTRDYKRIFRVKYPFEINSFATYFFVILIEEYVVIGTALFWMCGDTLF

AQLTTHASLQFEILRNDIESLMNEQIVDYAFRKRFSILIERHKHLLRICNRIEHIYSFIIFMTMVLSGLNICFNIFDMIELVNDGDYKAAALHSVLAIDTLGGMVFYCVFAE
TLTEQTGAIGDAIYNSNWTDKDYKLSIYFRMLIMKSQNPFYCTAYGYFPIGHTRLTRILNAALSYFMMLRTTA
>BdioOR13
MENSYSTRKYFGVYRYGTSFIGAWPYQSKTSAKIAQILFSIQHISIILPEVIKLFQSTHDLDLIIESVPPIFYNVILATMFGNAIHHKRDTQTILEKIHNDWKSLKDGKERQIL
SNYADVGKLMTWLYVGACYLTVAMFCAFPLSPMVMDYIVPLNQSRPKMPLYRTQFFIDEQKYFYLILLHNYLATYMGIVALVNTDLYIANITQHICGMLAVLRRRIEN
TQRTGIVYKLDDSLFNETVDCLKMHNNILDTLQVMNSTLSNSLGFLLLILVLLMSFTGLVVLLKWGMWTEVCRFGLFTNAELFHIFAYSYHSQRLIDHNGKVYNAIRC
SGWYQSSIRTRGLFNLMLLRCSKPFRMLCINFPLSLENFTLVNYYYFNKVILINKHFFIIFKIIKTSFSYFTVVKSTL
>BdioOR14
MRERGTMSKNKEKEEHAEVHESETAENYEYAVKIMRRLLQSVGIWPFTSATYPRFLRLFLIAAMCFLLTFFMVPIALHTFMIVNDFKTRARIFGPFVFGMINMIKFVTLI
LRENDFRECLDWLSHDWRMTRRRIENDLMLASARTGVTFTTMCASFMWVSGMSYSTLFSFMQQRTVVNNISLKAFAYPGYYVFFDPYDHFYKIYTIQSMCGLAAFTT
TAAVASIAIAFTMHVCGQIAVISHALHSLASGSFDKDTFCYIIEKHLRALRLANKLEDLLNGICLVELMSSCFHICILGYYIVMEFDGHNPGPYTYFMLIVSFTFNIFTFCYI
GEQLSEKCNNIGNMVYHVNWYDLSPNDTRSLIMIILSTHRPVVLTAGKITILCMRSFSNVLKAALSYLNMLRTVTTS
>BdioOR15
MSEQVAKPVWNKDSKFALSFNKFICWPLGVWPLDYNSVFSNFRLVFAFVSQIWFMGVQIAAASLHCGNATDTVDYVMMSVCAFMAMSKIVTFRIHMNKIRTVFLSA
LSDWVLIDDDKDRSIMKSFAKTGRFVFKLQIGFSYVSNTLITIGALPFLMPPMVTNGTLFNISDATETMLIRELPVRSGCMFGHYRDEIYGFLYVFESIMINITAHGNVGC
DVFLFTLLMHLCGQIELIKKDLREIGTIVEGSVESREQIKQFIQKHKLILELAKAIEITSSGMLVIQLFCNAGLNLMLGIRVIMALQNKLIFDAIRPLCGLTVLMLQLYLVSY
ASDRLCSQSESIVDVLYKSKWYELPPKLRKSLCFVAMRANKPVNLIAGQFYTLNIENFKNILKASFSYFSILRVMF
>BdioOR16
MKSKTERSGYEECVGVSRLLLQLIGVWPQSHGKGNFFQRNYVFVTPTVIFFILIVPQARKLMFVWRDLNAVVDILSIGLMIVTICFVKFLRVWHNYADLNILLENIHTD
WQTTPADEQKIMWRNARYSRLVVIVIAISSILDCNLLPLIRILQYYSDLKNAPDNVSIPKPQIMVADYYIDVQYGTMGDVMFAAQYITIMLCCGIYPGYDGMFALTTLH
YSAQLQNLQRRVEDLVDRVRRERCSFIDVVVPIINRHQHIYRCTALVEKNFSSIVLVQILAVSMLLCVQGYQIVDLLSKNNMTDFTQLIFTICYVYNSLFCLFIYCFIAEQL
RVQSENLLISISEIEWHELSPCDSKLLLMMMTRAKNPICLTVGKLSILSLEYYCTVLKSSASYFSVLMAVRDKIV
>BdioOR17
MKPPILSVKADIDEVMWPNRLMLVFIGIWPVPERDGYFARVFARFRLCCIIFTAVSFLTVQSITIYKFWGDIKLIAEISCVMLEIVLMLYKVIYVVIRHDKFKDTYRVISELW
SLAGNQEERNAVAEFPSRSKLITIVFFWSLMADVVAFNGGSVILCIRSHIALNGSQYNRYLPLPLWYGIDIEQSPIFELLLASQTVGLAVLATTGVACDTIIMAIIMQFVGQ
FKLVSLRLKALGRLVDHDISKDKVRRQMGLCIEHHQRVLSTFQSAEKLLNPLTFGQLMLASLEVCMCSFAMIEIESSAAYRLRFIMFLISIFIQLSVFCWPGELIWRESVAI
AEAAFYEVPWLAIPCRKQRDLALMMLRAHRPCQISALGMQIMCLRKLSEIFNSALSFMAVLRSMKEQ
>BdioOR18
MRASIKMAGERSGFDTAFGITRRAMQIHGIWPGFSASKFRMLRYAFFPSAFMILFFINVPQTVMLFRLNGDLSAMLNVLTLANVPIGIALAKLLGASYNQEVLHHLIISI

SEDWETTKKEHELAVMWEKAKVSRLLSKVSLILGEGTTLAYTVRMFYVLFNSKVPNKPLYMHGSFPYDTQSVPNYQITWILQIIATLMSSGVFSAVDALFISFVLHLCG
QLTNLQVAFSQVGKKGGPTRGPELIKSIEVLVKKHRRINEFADIIEYSFNIMFLCQVFLSTLLLCLQGYLFVITITSGQIVLVEMVFMLYFTVCFTFSIFVYCYVAELLREEVV
KLGTSIFYCKWYNLPAKEARLLVVSLLLVRKPLEITAGKLCVFSLNLFCVILKTAAGYMSMLLAVRDKLA
>BdioOR19
MYSRSLDEMLWPNQLPLQALGLWPTTTVIDRSCTISSFDRFRLGFSLVGLSCLVGPQLYSVKFYWGDVTYLAETGSLVMPFALLLFKLLYMIARRRDFVRIYRWIRRFWR
ISNAPKERAIYESIARIAKTVSIVALVACVADCVVVVGVPLLTELMGQRDCNDKMARQCRRLLPVDVVYGFDYEKTPLFESMFVLQTISVVIATTIATASDASILTAIVHV
VGQFRYINYRLRVIGKKFSAGKVTNVEADIGWLIKHHQIVVSVADSLTDLISPIVLGELLCSGLQICISGYAIIEGNATGLTLVKCIVFFASMMIQLTMWCVPGDFLDAES
GRVATSVCHELPWPQLPTSLQRDLVFVMLRAQRTCKVVAVYQATNVAKLTEVLKAAGSYMALLQNLKQ
>BdioOR20
MMMMHVLEEIKSTNTEYNLLPFQFFLLTFWGIWLPDNLSTKTKKLLQIFFHVVVLLDMIICVEMMIFIEESIKLHSFKLINVFLTSATITGIYKAIITSLNREVIRSFIRRFFD
QEFFKPENSEEASILEANNLQVRKVITSYSASMAGIFAIRAISPMISGDDKSMPVDAWYFYDVQKWYYYWLTYIHQVILGSSLICVHVGIDTLFVGLLLKLSYQIDILKYR
LQKISGMCYSKEKSSKHYKVAEKDIIFECIEQHEAIYKFGKGLNALFQDILLIQVITSIPNLCINIFVLSTDTGDIDIDYISILFNSTSSLIQLFISCWYGNEVILNSLDLKNAIF
NIDWTKLDLSTRKLFIIQMLRSSKPIEFTVGYIIPMNIDSFIKIIKTSYSAFNLLHQT
>BdioOR21
MDILKSTYYIRCNKYLRFFGQWPYQKLTNRVINLTLQILITCTIMLPQCIKLYQVRHDFGMVILALPAMFYHAAYIFKIIFCAIKSSKIRVVLDRVQRDFRVFKGEDIEIIHE
YSARALKINTFYTIYMYAAVAAYSILPLTLHTADTLFPLPFNQTRLQGKPRLLTYFNEKLDNSNFFIICHGMIADMLTIVFIIGFDTLCFALVYHTCALFNIVTYRMRDAIY
NQRKAKKLDTVSKEYNDVVYQNFVETIIMHKYVLESVEIIESAVSALYLLSIGCAMLPLTLTGFQVISNKGAVDQMLRFSLFAFGEIVHLFYYNWPGQKITDHGSIVYES
CYNSEWYGEAVSNKCKMLMKLMILRSQKPCYLTAGKLYILGLENFAAIVKVSMSYFTVLSSVM
>BdioOR22
MHLNEVLWPHKYLLTMFGMWPMHPNAPLARRRFANFRFIVASLIVISLIVPEIMTVVVYWGDIDILRDFGSLTVVNTLATLKLLYYKYRERSLRSVYQHIKNFWNISDD
PQERRHYERPAQVMKVLTIFCWFAGMSDWLSFEAFSLIGIVKSRRAIDESLRQRPLLLDLFYGFDTQRTPNYELIYVWQTIGVFYIVSAIVGCDTTMIVFIVHVSAQFKFI
AARFRAIGREIGEQRMMMKDDDDRKLRAKIGKLIAQHQHTIKVTEELSTLLSPMVFGQIVTSGLVICLGGYAIMEGNSNAAYFVKYIILFSTLLLQLFAWCASAEFLIHE
SISIAEIIYCEVPWFKLKPSLRRDLNLVLLRAQKACYVSALYQVMSLSRITEVFNAAGSYMAMLNSMK
>BdioOR23
MDEYKIFDSPYYNIQNFYWKLLGFWPLDRYKWIKRVIVITIIVSLIIPMAIKWVEELTSDIDIVLEVTGYLIYLSGCLIKYLIFLRSETQMMFLFDKIANDWRIMKYDEEIQK
LKEYGLAGRQLTKTYMGVLNVALVIYIFVPLIPVFLDVIIPLNETLSRSLPYFAEYFVDEQVYYFELTAHAWIAIPLTVQVFCAFDTTYAVCVQHACALFYIVEIRMRRAA
MIATSEKYTDKKKQVEDEYNSIRAAIMLHMEAIQFIDEIEKSYSIIFLMIVTLNSAIIVLAAVVVLMSLEKGNMKQLTRFIANYIAFSAHMLFNMLPSQQVIDHSANIQN
AVYNCNWYNVSPKTSQLLKIVMLRSLRPSSFTGAKLVELNLENFSNVFRKTISYVTVVASMR
>BdioOR24

MMAKMIDDTGIEYKLFYDTFFVMKITGFWRPTTIKQRWLKWLYGLYSIITSINVSLIIIESLAYIAISKDNFLDAFAQNWYLPAIVSHTFYVGLNVKMKRKKILNLLRTNI
MVERWANLRDAVEIKIIKASDKTERNIFRYWMSIVAANGFMNVLNPITSENPDNELIFNTWKPCDTRISSCFWLCWTSQALSYANASITNVSCCCIALNLIERVCSHVR
ILQHRLSLMPHLVSTGVLNTAEKEKVYLVQCIHDHQNIYSTTKDISDLFSRVITLYFVLNLSILCTNIYLLSNQPLFSPKFIAAFLYLCCMLTQALVLCWCGYKITQINDEIS
YGIFSMDWLELRRDTRKTLRFIMLSVSKSIPLFNNFLVELSPEMFLKIIKVSYSAYNLLETMK
>BdioOR25
MDGQEGFDFVFKLCSICLRSIGAWPELYESFVGRALTECHYFANLFILIFFVNFMQTVQLIYFWGDVDVMTNVLSTADLPILNVSCMMIKFWFKRQVLRDLISMVRND
WSRDKSKKELELMTSSATMARTLATICVSLGQGSCLFYLVLHVYLQFTLNLDFRLLYLNAYVPYDSQTSPNYEFTWLFQCLSTFISTAGFIGFESFFVIVVMHVCGQLHC
LRHRLGNLNYGDASVDVRTNIAEIVEQHNHLYSVAYNIEDIFNSVNLVQLMNFTVIFCLLGFRAVIMIKHGDGTLIEAIFVVGFFTYLSSNLFICCYVAEQLRSESLAVEF
AAYDSCWYNMETKDARLLINVLMRSKKPFEITAGKFFIFSLNTFSSLMKTSASYMSVLIAMKNEDDS
>BdioOR26
MDIYDSRYFSINKRLLTVYGLWPYDRPYRKIITMLGLVLLPILNVPHLCGLVIHFDNVEAVFEILCVQVYLVGAWFKLLTIALADKKMKILFDNIVKNCKSINDESERKIL
HHYSEKGRFMTIFYLMVGLLCLVAFSFLPLIPTILDLIHPLKEKRPYIYLYTYAEFFIRNKDPYYWFFYVWDFVTVWMVVSICVAIDTMYSACIKHCLGLFAIVRHRVRAI
GRSVWDGSLKGDANSTYQQMVRVIELHQKVLQFTDIMKDSYNICFLVLILIHLIGLPLCGVVTIIYIHDPINAIRGFLAFLGLAIHQFYLSWPAQELTDYSGDLFVDSYSN
EWYTCAPDTKKLLRFFALRCTKASSLTAGGIMDMNMENFRRIMKTAASYITVFASFRATESN
>BdioOR27
MKYFFSFAEMDIDFARRASIDCPENADEIRERLCLLFENSAFVHRISGLWPKNDQFLGQYRAIKCHAGIISTFFAVLMQLSLITRIDKDHKAQFMMEFGIGLSALVKILVC
IFNRRGIIKLQQATYKVNSQTRESSSEKRIAYWWLGLQKKLVSFTVVSYTCVYFAYLLTPLITGDMFPLLGLVPNVFRELPWIVVVYPIELIVSGLRCVMIMSCDCYFITFA
CQLFSELQLVQRAILDVNRRSIERVIKRHRRVLDYGRMICRNYSIAIVTQHCELSIFACMGALSLPYLKNMALLYNFLGLLIMTATQLLLLCLVGDKIAAESLKISDCIND
VYAEFLDDVTSLKAILTMLTRAQRPLLLQVGEKFIIDLNLYRLTMANISSVFLVLKKFIDE
>BdioOR28
MLHLNRRLLSVVGAWPLEPDASYSTKMFSLLRIIATCVFCTAMMVMQLITLYINWGDINVMTEIGCMLLGFAVSYFTYLYAVHQRSDFCIAYKFICEFWNAAENQEER
NNIVELANHSRICTIVYFYLAYVEVPVMIFGAASVTFFNYVTANGTDYDRQLPIDLYYGIYCQMSPNYEIIFVIQSLALFYGATAAAGVNSTTMSIIMHIAGQFRVVSLRL
KAIGQIVSDGYQFQNKPTNVLARKIKLCIIHHERVLSVFEFVNDLVGPAIFGLFIIAGIEICFTGWAIMKSDSNVANTIKFVILLMIVWLSISLWSWPGEMIIQESSNIGHAA
FQNIPWYILPCSVQRDLGFIVRRAQRQSQLDAMGFEVMHLRKLTEVSNTAASYMALLRSIKE
>BdioOR29
MKKMMCYRSVMSPSIEVGLRLIGLFPGATQIKGFFIILLIAAINIPFQVWNAIEAYGDVDRFMNNFGPTLVMINFAIKLVAFRLSHRAVKYLVNEVIKDFVNFAKSSNHK
FMAKNLKTSNSIYKLIAISYNALISWCIIVSVGSYYKTKSVEKTEYFIPVTYPYITKNWKNYSLANVFLLFQMIVAANGHAIIEGLLALVVLHAATQVHNIRLAVKCYSEV
CCSSNDKARINIALRSLLLAHQKYLKFAVNVEKAFTCISLSQLTIVTLQMCAVEYAILTLIENNAMTAEGYMYLNLLIADLWGAATYCAAGEYFIIQSDYIYRELCQCPW
YKFRLGDMKTMLMLFPKMQKPPVITYGKLGSLSLFCFTSILRASFSYLSVFRAIKTRQHRGTN

>BdioOR30
MKSQSAVNLNYFQHRAWQLLALNEKINGIWPYQNKFVQYPLQIFHFFGLSSMLFALLNGLRTVAAGDIEIILENAFWIMFTSTLSCIQLLNLTGQRRRRKFYEKVVKQ
YCAMRIIPEEAALLSESADQSLKFVLVYLSALLSCVVVFYSVPMIRALYEYTIGHEHELKITLGAGLDYGVDQGKYWFLLVPIYLNFFFSGFVVGAGFEFSFILQVRHVRT
LFEIVRMRFDRAMEFVDKDDQSFSTIVSAIKLHVECLKMVNMIEDNYSTFFFLLLVMNSAFSALSLHMVTHNMQDPIEAVRFGCLFALIALHFYVIYNHGQMIIDASL
AIHDARYLCEWLDLPKKSRELFAIAMMMQSTEACQLTGGKLFPLSMEMFGKMFKAGLSYYTVFEQ
>BdioOR31
MAAKTIGKKDDKSPLTFDQMCKLYISFFKLISVLPFDGRPLSFVGTQLLLYFRLFFVVILNSASMFYCFNTSSLEIEIISLTMLNFGIGFRYVLVLYKRKAFASLLNECRDLW
RFCNEDGQQTVLYYERKFRHVFVIFYIFGILVNISYPLDALNTRLPPLEPNGTGYHPFPFRWFADNTEGNWPGYWTVFVMQIISVQNAVDYAVPCDSSMALFSMISTGY
FKHLQDRLLFCHKNPTEAKRDVIRCIIFHQRLFKFCSRVNDFYKIISLSQILNAGYSISIVLIKLVSSDSDKYKYLMILGVFICQLYLVHFAPDYLQDESEEVAIAAYFAYGNS
LECSGVGKYLLIMITAAQQPVKILAGGIFVLSLENFGNMLKSAFSFFTVLRNMNF
>BdioOR32
MDILPLNFRTLSYCGIWLEDAESFIFLKKFWGYLLRTIIFYFTVTEVIELYMLKDDVQEVIDVMFLTVTYICLCLKILNFSIQKHNLKKLIQYFHEDSYKITSAEENDLLQKY
VIQHTQIFKNILLLSQTTGIFFCLIPFVTLKPADYIAPFKTYQFYDDSTTVGFSITCIIHFSAIIFGIFVNVSTDTMIYGFIILATGQFDIVSYRIIKAIEDKDIDLFRKSIIHHIHIN
KTVRKMQQVFISVIIPLFILSLLTLCASIFQMAQNDIVSVAFLGFTMYLSCMLAQVFLYCWYGNELKLKSQVIVSNVGGCNWIAMDHRERKNLYFLLMFANKGYQISW
KGQCSLSLDTFVWVTINIFYAHRIAQFEFNTLFFTLLHVFLDYKNVIFCV
>BdioOR33
MDLDVGTVEKVFGNEYLHLNKKMLILSGLWPYSSKLENLIKRILIAIVLAVSAFPHTNAIRLWCSKDLGMCAENIAGWVYTTGVLIKYSVTIFKEKKISRVYELLAKTW
TSIEDPDEKKIIFENAKIGKIKTIGYAGYLALASFVFTQTGMIPVIVDFIVPLNESRPKIEMVHTEYGVDQDKYFYEIYYFYCVLGPVSAGVLMSIDTMYTAFVHQNLAIFN
IVKHRLHLATKNPSKGYEKKEDDFAYNMIISAIRLHQKAIEFNDLMENTFDILFLWLVIICVLFLSFGAIAIMENATNVIDLIRMSMLEFGVFIHLLYMSWPGQLIITESSD
LFVQVYTNNWYHISPRSKVLIQIMMLRCMKPSCLTAAGLYVMNFENYAAVIRMMIHT
>BdioOR34
MDIYDSRYFLVNKKLLISFGLWPYQSFIRKLTIRFLLTIAMCMMLVPQIFQVYFSIGNIDKLIEHVSVVVYVLISFTKLYTAIFHEKKLDILYSNISRNWGIIQDAKERQILMD
YSERGRLLTLGYMAYMVAGCIAFDSLPLLPKVLNILRPMNISRPPTYILDGELFLDRNKYYLVQYTIEVMTTAISAGLMTAFDSMYAASVEHCLGLFAIIKFRLQNSTKSI
CTDNDVLRKNNVLRQLIQTIRLHSKILIFTDVLAATYESSFLLFVGATMILLSLECVLMLIKSDQFMQLIRYGIVLIGAIGHLFFLSWPGQKLIDHSASLFEDGYNNEWYE
TSIQSQKLLAFVTLRSMKPCCLTAGGIYVMNFANFAMTVKTSLSYMTVFSSLR
>BdioOR35
MGIFEGRYFHLNRKLFILIGLWPYEIKTKNIVRTLFIILSIEQSLPHLISAIMHHDNMDRLMLNIFSFVSIIAMGTKVLGLSLNENHLKELFESMERIWHEIRDENERKILLK
YAERGRLLTISYCVFMSVAGSMVATQQLIPKLLDRIFPLDEPRNVMAFVDGEFFVDREEYFMTLFFVDLISCLVVCMVTASSDAMYMVCCHYCCAIYAIVRYRLSKLRR
MVLEDASPIEESNSYDWLVHTIKLHQQTLKFTYDLDDGYSLSFLFYVPCYLITIPLSACGILANLEKPLNLISYAGYFSALSMTVIIMCWPGQLVLDQGNMLRDEALYDN

EWYATPQRTKKCLLIFMTRCAKPEALTAGGLVFVCLETYSAIMRTAISYITVAASFR
>BdioOR36
MHEAVRAYYDNYKNGIASMLMVVGLWRGDARQSRILQNILTTFAITELTILILLVLNFTVHSSRNILGLVAGISSIMSFSTVYIKGTVVLVLWRDMKDLKRDLSTLFEEDL
KVASNREHLLAYYPAFYRFFKVHSYIGTGFVVFSNCATLWAALHGKYVRTFPIKVPFDYVPGDSLHWIIFAFEVTSSAVIAIITVGMDMMFGLFAFHMAGQLRLLSHRF
RSLKSGENYKRELKECIDRHCMLIDQKYTMERVFGLTSLLLAFSCASIICSLIYQLTQSKSVTLFQAIFAIDYIGVKLTQAYTYAWFGETITVESERCVESIYYSAWAESGDV
QAMKDVIIIRAQSPMHITALEFMILRLDMFVKIAHASISYFFLLKTIEAKVTWAE
>BdioOR37
MDIFDGEYFATSKKLAIFVGLWPYDTRKKYFVGIFWLTMGIFTAIPQVLGAKVHHSDVDKLIEHVLMIIFITGCFFKLFTLFISQKELKTMYRGIVENYRIIADAKEKAIVS
ECTARGMEIANAYCVFLVSEVSAVIFPPLIAVLLKQFIPSMEKLPYIYIFNGEFLVDDRDAHYWKIYFCDVALMLFILMIVMGVDSMYIVCIEHCIGIFSVIKYRLQLPDKLI
DNGRMIERNDAYGFVVEIIELHKRILKFTETLEASYSMCFLIILLLEIGALPTACYLIISNLKNPISCLRFTIAFNCLMVHYFALCVPAQRLMDTSNNIFSDAYYSEWYESSP
RTKKLLWFFMLRCKKPCALTAGGLVTMGLENYMRVQKTALSYVTFLCSLR
>BdioOR38
MDGVKGFEHAFGMCRTNLGVVGLWPSSNTTKSKGLAKTVLFAVSFVIVATFINIAQTTKLIMIWGDLTNMIDNISTANLPIWVVLMKMVIFRTHKKALGSLLTDVIND
WNAPKTKLQMKNMCENASIAHKISFLCVILGFGSVNGQMAIRISQEFNILPGPPEKRLPMVSSYFPYNYTISPVFELTWLMQYAGAGLATLVYSGVYCLFVGLVLHLR
GQVANLRVQLEGSDEDDIVIPRMQFRKRIGLVVHRHQSLNRFAKKIEKIFSMMFLAEILSCTVQICLQCFMLVTLLSENTGGISFLQILFMMIYVMHVVTHVFICCYVAD
KLRDESLSICDSAYNVPWYNLSARDAKLLIMIMHRAEKPLEVTAGKFVAFSLRLYAQVSLD
>BdioOR39
MEIFDGRYYESCKRFMSICGLWPYQNDKRRYLTSVGFYLLNATLAVPQLILLAQKFGKDIIITVENCVSVMFSIGVVAKYTVTFISKKRLGLLLTQISKDWQRLTDVKERE
ILTKNTEQGRFLLIIYTYYVFAAWILYTLLPFLPVALDLILPLNQSRPVLMPFYANYVFIDQSDYHYTSCCHIAVVYLTCFFLFSGTDTTYVITVKHTRGLFEIVCHRLENLL
DIEKGNEPSELVDDEKIITKIKKAIQLHNETIRCCDLLENSFNLCFLMVNCMSVVGLALCVVYLLIIFEDIFKAIRVIAFIIGLIMHLLYLNWVGQQIIDSSEQVFLSAYSSN
WYFMSNRARKMIDIILVRSLTPCALTAGKLSTLCMESFGIVIIICAIIIT
>BdioOR40
MRVATILPFHFFMLTIFGVWCPVNWSPLLKRLRKLYLLLVILATHILVILQLARLIFVRMSFDEFNDTFYVVIATFAACFKINCNLFHYDELLRLVDMFQQASCLARDDFE
RNVKRKYDDTCGYVGKALLLFVELTVFLFLIVPLNGGIESRQLPYEMYHVYSLASPLVYWLSYAHHVFAGLLYGGLNIISDTLISGFLVQLCGQIDFVAYRMSNLPMEA
RRICCDRDEAMKTIVETTLLKENIRHHVHILNIAETIVKAFNSIIVLQFYLSTLQICMSVYQLTTRVTNVVELFSMGFFLMCMFVTLGVYCYFGNELSLKSVNLSDAIFNI
DWTSLSVSTKRNLVFMILRSSKPIEISDGAFVRLTMQTFASIVKTSYTAFNVLKSS
>BdioOR41
MDVFEGRYFNVNQRLHVILGLWPYLPETKKKIIRYVYLAFMKALSMPHLLSMIVNLEDIDRVMLNFISYFTVIIGMFKMAGLIAEEDRMKKLYESIASNWLTIQGDKER
EILHKYSARGRLLSILHLVFASLTLVVLLLPMVPKILDKLDPLSEPRPDMYVLEGEFFVDRKTHFAEIFFVDWITIAATFAPAIAADSMYVTCSQHSCALYAIVSYRLQQLR

RMALDDGPSTTGNCYDWLVETIRLHQTALTFTTTLNDAYSTAFLLMMPFCLFLVPFDSCAILANLDRPLYLIPYVAYFVSTAVHMFILCWPGQTILDHQIILSDDACCNE
WYTTPFHTKKLLLIFMTRCTQPATISGGGLFLLSYETFSTVFRTAVSYITVAASFR
>BdioOR42
MDIFDSPYFRVNKKFLRVLGLWPYDFQRKMVQIFWIIVVLLIAVPHVTAVIVNASNMDKVMEHLLNLLIATQVLLKYVTLATAENKMRYVYNNIANNWLTIVDREEK
RILVEYSKRGRTLTMIYICLLTSTAAAFFVMPLVPEILNVIDPLPVPRSHIYILDGEFLLENRDDHFYKIYMWDTASVLCVITIYIGIDSMYTVTVEQGTALYAIITYRLSELR
GRFLNDLQDETVSSYKWLVHTVQLHDTVLSFTNNISDSYSTCFFCLSIMNIVLLPLSCVVAIVHWHNLVLLTRYLLAIASLLIHVFVLFWPGQKLIDHSTRLLDDAYCNE
WYETPEKTKKLLRILMLRCSEPCTITAGGLLEMNFEVYLKIVKTAASYITVLASFR
>BdioOR43
MQSELKEYEKYTGNVKAMLRYCGLWPAVGNQLVSYTLAFAAFFTTFSTMLAVLNFCLHHASNIIVLTKGAGLAISLCTASLKVCIFFYHRRDLRYLHDNLTGWYLED
MKDTDRRSQLLTRVSLYSRFFWVGTVAAFATIALYASISIIAWAKYGKYLRVFPVIYPLVGKPKGLLHWIFYAYEVTTGMFLSFVTAGVDCSFGMYSLQMCGQLRVLADK
FRNLKADGDYKMKIRDCIQRHHMLYASKKKLENLFGILTIWFAITAAIVLCTLIFQFSQAVKAKSTSLQLGLLTLYFLLKSLQAFSFSVYGNAITVESKSCLDAIYNSCWP
DVYNVSLKNDILIVLAQKPITLVAKGCMLIQLEMFAKIINTSVSYFFLLQTLEEGSR
>BdioOR44
MRIHGIWPGYCCSDSKFMRYVHLIPSCMIFFFINVPQTVQMFHVNGDFNEILNLLTMTDVPIAIAFVKVMCVSLNNKVLNKLIVSIAEDWTSTTKSSDLTTMWFIAKIS
RLVSVVSVCLGECTAVAFTAFMFYLMYTAFANHSENEDFFKPLYMHGWFPYDTQISPNYEITWVLQIIASFLSAGTFSSVDALFITLVLHLCGQLKNLQMAFRELGEME
DIETKTFVRTLAVLTERHQRINHFAGTIEYSFNKMFLFQVCASTILLCLQGYSFVIILTEGNIVLMEVIFMLYYTVWFIFSIFVYCFVAEILQDEVDKLGQAIFYCNWYNWP
AKEARLLIIPLVRINKPLQITAGKFVVFSLNLFNSILKSAAGYMSMLLAVRENLK
>BdioOR45
MKLHDWESVSRSHLQFMRFFGLIPLDGKTLDYLVTKLLMVYAILINVFYNSPYTYYAIDKFSKKIFEAEYICEAVVVQRVFFCFFTLRANRGELTRLVENCRELWGHLTD
DETETVEGFERKARQFRNFLIGNGIAICTAFYGTFFMLTASIPKDQKPLVFKFFVEVHTEPWYTIVMIMQFVVNYTIAVQMGVVEGIGLYLIMMACGYLRVIRNRLLVISE
KNQPEDDDEMVNEEIINCVKFHQNIMLYCREIEKLMSKVYAITVITTVYNISIIGIKLLENGDDKFIYITILTNNVFVFFTVQWAPEHLLSESEAISQAAYFASQRYEPQLK
MKMRRTLLLMTLRSQRPMQLTAGGIISLSIETFGAMIKNAFSFFAVLRSFAE
>BdioOR46
MKAAKILPFHFLMMTLFGIWCPAHWSTFQKYLRNLYMLLVMVVMFSLISLQLARLAFVELSLDEFNDTLYLAIATCGACFKFSYYLAHRDEVSRLIDMFQVSRCLARD
DFERGIKRRYDADCTLVAKWLVTMVWTCVAAMIATPLSAGPAARQLPHEVMHVYSMSSPVAYWLSFAHHSCAAVLIAGTSIISDAIVAGFMSQLCAQFDFMAHRLQ
LLPRQARAFGSVHVKTSLLRESIQHHVYCYEIAESIIRTFKGLIVAQFYASAMQICLSVYQLAFRATNIVEFLNYFSFFVCMLFTLAIYCYYGNELTHKSKQFCDAIFETD
WMSLTVRSRRTLAFMILRASKPIEIADGAFVRLNMQTFGSVVKVSYSAFNVMRQSK
>BdioOR47
MDFYDSRYFCIPKILQISVGLWPYHPSKKLLRTLWLSLSLLVPIPQLIGVKVNFNDAGKLMDHICMVLVPLSIFMKMFTLVLAEEKCKIVYTGIVDNWAAIADVEERKIL

KRYSERGRTINIVYLGFQSLVYVAFLFLPLIPVFLNAINPLDEPRPYIYVLHGEFLVENQDNYYPWIYLWDGAATTLVLAVAAPIDATYVCTVEHLLGLFAIVEHRLLDLK
SHNASQGHVVDILKLHHRALEFVDIIEEQYSLCFLFLIITQLIMFPLICNLMLMSLGRPLDMFRYAMFAGCLVVHLFNMMWPGQKLIDHNDRIFQAAYQNEWYTCSL
KTKKILRLFMLRNLRPCVITGGGLTVMNFENYFAILKTAASYVTVFASFR
>BdioOR48
MLSFHFNVLTIMGVWCPVDWSVGWQFAYKIYSVVVIFLMYSLGLSQLAQAIFVKQTFDEFNDAFFILLSTNFACFKAACDLLNQRQIVELLRMFGKECCVPRDDFELK
LQKRYDGTCRRIAVNLLAMVELTALFVLVAPLASNGIKTRELPYKVLLPYSLSNPLVYWLTFAHHAFGAVSFTAISITNDAIITGFMLQLTGQLEILQHRFEELPSNVAKS
NTKDYERRLLRECVQYHTHIFKTAKLITRTFSDIIICQFCISALEICVSVYQLSTRADNSVELLTYAMYLVCMLGQFFVYCYFGNELTLQSKRLSNAIFNINWTPLSVEFKK
HLAFVIQRSSKPIEISCGAFVRLTLESFVNIVKMSYSVFSVLKSSA
>BdioOR49
MCKYISSHLTAKNGLKFLNFVMICHGTYPLPNIQSKFIKAMYGLLWWFWMINNAALMALFFYVVFTMNNTIDSASYSLIEVSTIFERAFILILFRFQQYRIMVLMEMME
KQLLVKTEKKSLSISNLYLTLITLMFTCYFSVLYYYLKLDIQSYSFFTKFAISFPKVVADRLFKLNIFNKMVAGFHALVLLIADGMMVLFIHANTMRLKYLDLSLKAAASC
DRLKKCIKEHQDIYFMINETNFIARLVIVKTVVFFIAYCFCSGIQIFNQKVQSSELSLHCLIVAAVFLRVYVCAESAQNMISAAEDMNTTINVSIWYSHKLDFVKSMIFML
LRNQKLPKIHLTVLMPNLNRRYLGMILRITYTYFMTILTLLRKRTS
>BdioOR50
MPSSLQLRLCHYYFYALVAVDGFEWAFGLNRLGLDFFGVWPSKNNSHRWRNAITIPTFILVLFSGMFLPQAQALVEVHRQLPLVADNVVTFNVCVVGMAKISVLWM
GRKEIAELVRQVYDDWLTDKSTSELHSMRRHAQRARLLTILSTSIALASIVGFLFSPFVNISIRQINNITDLHPTRVLPLQTYYPYYDVYRSPVYEFTYLHQIICGYVAATSH
VAPENFFGALVMHACGQCEILEAMFERLAASASTQDSTTFVEQLRRVVTRHVRIIRFVDDVESLFNIMILGHVLFVVVNVGFLGFSCVEAFGENAESLPLMQVVTLTG
TSVTLLSLSFIICSTVEMLTTRVSFSLKKNSIFLPTVEFFIRKCILQS
>BdioOR51
MKFNSKLKAALFFTKYSVILLRCWPPRNPSKIQYIIFELSWWLMFASSLCLLFPLVAAVYIYRGDSEIMTKCICLSCAVSQCTVKTLFCRIRRSKLQKLLEEMEDFVENPSE
LDRKILEKYISQCTILHVSVVIWVYLTSVSFIIGPFLIPQPFPTDAVYPFSVNNTFIKIIIYCHQSLVGLQTSAAVLLDCLVAVLLWFVCARFEALAISINTYNSFDDLKIHIKQ
HQSLLRYTNDVKETIDLFVFATILTSVGGVLFSAIQFIVDQPLAIKGQYAVVAVTASVGLFICSWAADTLLQLGHLIGINVFQSKWYSMDKKTQTSIMYLITQSQHPVTM
KANRILPALSLQFFSQFLVISLKFFASMRMMVGIQ
>BdioOR52
MDILPVPFRAMRILGLWYENPKPHCLVKYIWRPLNVFFLVTVTISLFSSFLAYINDVKDFTECLFITLSYLGSTLKLANFLARHSSIVKLLAKFRARMCQPQNDREICIIES
YERIIAYGCNGHLSIALLCGFTFAMMPVLAILAGSHEYFMPYNSLGFIDITNRLHFWLIYAHQVAGDTVGIIVDVALNFIFVGLLMMACAQIDLCCRRLVNSCNDVDIR
RAVEECVKHQLLIWRIVREVEALFMMIVVMQIFLSLITICTSVYNMSQVSLAESVPMFIFFECMLCELFCHCWFGNELKLKSTAIRDAIYNSEWTTLKPSNRKSLHIIMM
SCHREMNISFHGLCTLNIEIFVWVLKMAYSAYNLLKTVDK
>BdioOR53

MASSFEEMTLLYSRFLRFISILPLDERGFASLTSRTLFTYYFVSSIIHWSMFVQHIINKAAVPTVDMICETVMVLGMNARYYLLLPRRIEFAALLEESRSIWYAIDEEDRRAV
QHFERKMRRLFLAFLGSCIVTDVAYFASAVILNLEAVKKANDTHGPQRILPYKWYMEGGEDMWLGFKFNFALQVIVVANAGVMTTTVDSAAPLLIMLGAGYCRAL
QQRLYRVYDRAMKSNDDYSRADLELLTCCRLHSRVLCYCMKINRMAQTLFFVQLACTGYNTSLIAIKLAGVSATTYVANNLRKKKLFSNHRRRIRTDTSTCHLDYSM
SANCSSASGRRITFSTRARQFRRLRTASPIHGSIVELANCWY

>BdioOR54
MNPLNFKNVRFIAIFIYTCVHFTMNIANLPDSMHSISHLLDTFCEVMSCCTMLIKFVSYKVQRQKFAELLCDLSLDYQLNAYKNEQEIDEFIAYMDMAKSFLKATWVF
SLAGAGAYFSKNWFFEGLGIPTYTDLANVTVMRLPYSVKFFYPVTNMKVYALAVMYQLPMIAIAPFGVIANGSLLVAMVAHVCGQLSVLARHRFPRFGHNTNEFQQ
LVADAVRLHYRIMSLAKTINSAFSFIIFEERMSASIVICFLGYASIMTWNQQDISQLLFVISDMLAQLLIIFEYSYIGQCLINESLYLNDALCDTPWYTLDAANMKYYIILLA
RTQQPLTLNAGKFYVYSLESFVTILKSAMAYLSVLKAVI

>BdioOR55
MNNYFRNRFHKLYFFILFFLIHKRKKIDLLLKSFFLRNRQSHSLVQGAQTGSKSFATPKMDRDYTKYLSYHKLIDTLIFRSGLWHHFEDKISPYRLIIIYGLVISNIIGLKVFS
AVISSYGDVSACLHAVQNTVAIYAPMSRMISYLWYRQEFVFLDKELGAKFKEELEKEENRPTLLKNISLYNKFLYFFIASVGIAIFLYWAPFAVLNFKYKKSVHIMPGSYP
FPVEPRSSFYWAIFTYETTMLWFTFHSLVGVDSAFGMHCFRISGLIRLTAERFRDLDPNDPKSKDRLQQCVATHQLIIRAKLALEKAYGVLIIFTYLISAVVICIVLWEINQ
VCVYIYRTYVRVWIRHSSC

>BdioOR56
MELFKYRPWKILAKCEKYTGYWPYKQTCSLCTLYFVILVTRILPQIIHLVKILHVNFVDVLECIIGVPILTVMIEKLVVPWMKHNCKLQIYEKIREHFRATSDVEEATILRD
FYDRGWQFSVNYMVMSFSFTGLYQLTPVAKKPFNHYFRNVTLEKQLPVPVDYLVDHDKFFYPVLLHIYVGALTAVLMEITFNLSFILIIHHICGLFKIISIRVHKVLDLAN
RAKVTAKVAIKIENSMKSTIILHNEALELVTLLEESVSVPCFVILILNSAIFGANYFIITMHLHDEFHLFRYCILFTIIFSHFYLIFSQGQKIIDSSSSVFDACWDCQWYTTSKK
STLLAVYNKTAKQQAE

>BdioOR57
MTSMSSASGFDVEIGPSRVILRFLGIYPDPKRKLSWISRSYFLIPALTMFYFCNIPQSVTVTRVWVDLNAVLEVLTTSDIPIGIALFKLLGLRYNADVLSRLVSSMSDDWKSP
KTKEELQIMWANAKLGRLMSIIVISLAEGTIVAQFAMVVFFSYSEYKRQVESNVTERFRPLYMNADFFYDVQKSPRFEITWLFQCFSTIFAASSFSAVDAFFAVLVLHLCG
QLNNLKENLKSLSSVGARSEETYAVTLADVIARHEYLDKFAKSIEDAFNVMFLVQMVASSMVLCLQGYQLVMIITAGDGIPLFELIFMIYFTLCFMFSLFVYCYVADVLR
KEVKKDALICCIFTKFFF

>BdioOR58
MCEIKDVILGNESSSMHVLQLPFKLLTYTGIWMPVDWKLPRQKFAYVVFSIFCNGLYLMTHFSMLGYLMMARSWEEFSDRLFLIPTGVSGVHKLVFILFNRRRIVELAN
SLLKRYCQPMDGVELAIQNKYDETIRVITIACACLVNITMMNLVALPLLPSSSNHTLPLKVWLPYSVKSKLSYWITYAHQTFGILLVGTSAVGSTIMINGFLFQACAQFE
LLSHRFHKLPKVIEKLRNSEAPEQFICEFEKKAMKENVRHHLFIFSFAETFNDIFQSVIFQQFCISSIVVSVSVYQLSTRPEKNMEFIMCFFYLICVLTEFLVYCWFGNEVM
FEVKLNRKKTFARKKNNNN

>BdioOR59
MNVNYFVYSIAMLYVTLQTIMTGLSLPENLEYVDQTIDCSNELIGLALVIYKLYAYKTKAPLLAKLLGDVDANLSIDRFKSEREVAIFVKYMSYAKTYFKVSIVTGYITSA
FYFLHVFVLDGGHIPTQTSIHVPTICKIIVIFFDNSRNHLSTSDSHNVTTYLPPYRVKFYVPLVDLTTYSVAFIYLFPMMVVGANSYMVDGNLLVVLVAHACSHLAVLSH
CWLELLGDQPEQFRRHAAEAIRKNYYIMKYRYHLNNCRMMILGNSISRLQIRSRHQFGLFHHDAAAEGRRGICPVPFHVRRSSGLATRGKSSYINHCGIRVWRDMC
CFCLQLCRSMSHR
>BdioOR60
MLNVLAFLTAMVVFLKMVALTIAENKMKELLRSIANNWLAIRDEKEQEMLVQYSARARIITIFYSITMAVCGTSISMLPSIPKILDKINPLGKPRQNIYLLEGEFFVDREE
YFIELFFCDAAITLMVTITTMAVDTMYIVATEHCCALNAIISHRLRNLSKKSLENSSKGDDDDSYNKMVQIIKLHQTAFSFTDAINDGYSLNFLFIMTVNLLIIPLDVCAI
LAHLHNPISLCPYALLFVAVMIHFFVIFLPGQRVLNHNLGIFKDAYCDDWYILPLRTKKMLLIFMLRCNQPASLSAGGLFLVNFETYSMVMRTAVSYITVLASFR
>BdioOR61
MEVLPVPFAVLTYFGIWKPTEWTSNWKKNAYNFLSFLVLFLMYSFVASELIEVVVYLFKYKDISETLFILLTTSSVCFKAVAFLMKKRDMMYLGDMLRENYCTPKNEDE
KMIQKKYDDINSIVTKVCLMMVYGNVLSMLAVPFFQTSEDRNLPFKTWLPYSIDSRCSYWMSFGHQFLGLLCCASVSVANDTIVTGFMLQACAQLEILEHRFRILPKY
VEKARKYMADKEVEWREENMLKQHVLHHNHIYDYISNVVDIFNSVIFVQFCTSAMVITVSVYQLSMKSPGTIDFIMVFLYLMSMLTEFFMYCWFGNEVTVKVGRVK
>BdioOR62
MSLLHNKLFKFFLKSVGLWPNHFNIIGPLVIVSIVVTIFPFELWNASNEANNPVALMDVLSVFLAKLSIFIKICIMWKNKRKVDDMFTDFLKDWSKTEPSTEKQVLVSVS
HTFFIIVISMYASSFSIYCLGSVITYFNKPVENRTLFLPSFYPFHYRRSPVYEGICIMQYIQGSMLCIADAVIHCIFLTLILYASVQIESLNSCICKYSNNLDGHDKNAGDLVK
DGLSSRNTHMRNIVIQHSKILKLVEKINDVYSYVSFFQMGLNSLPICAVAFVVVTAMESADLLLLAKFILFTLAMLEQSFIFCFIGQYLQNKAIEGFY
>BdioOR63
MKVIHFLLMLKLLYYKWRQRSFRSMYQHLKNFWQISNDPQERRLYERPANLANLVTIAFYLMGMMYNLLNFEFIALFDWMANRREINDSLWHRSLLVEYYYGIDTQ
RTSTYGIVFISQNLCCLCAINAFIGCDCTMLVFTAHVFAQFKLIAARFHDIGLKTMSKQLVVENELRFEIGKLVAQHQHTIRVAEELNALLSPLMFCQIMSSGLQICLGG
YDLIEGFNIFKFLVIFLTLLIQFVVMCAPAELLIQESSAIAETIYHDMPWFKLTPSLQRDLGFVMMRAQKKCHVTAFYQVMSIRRITEVFNTAGSYMTLLRSMKN
>BdioOR64
MDDLVKAYYRDYKGAMVSVLLVSGMWPDDSKQSRVLRTILNIISVSMPATLMFGVVNYTIHSPRNILLLVGQIGLVISFGTVCTKMMTMAVMWRGVKSLIGGLSALFE
SDLKVPENQPHLLAYYRPFYRFFKVYYIYCLGLTVLSSSIPVLAAFRGHYVRVHRIRPPFPYTPGGAVHWIIFVFEVSATALLSLNSIGIDSVFGLFSLQMVGELRLLSHRLR
SLKSGENYRRELKECIDRHCMLIDQKYMMDRVFGLTSFLLALSCAAITCSLIYHLTQSESVSLIQAFFAIDYIAVKLVQAFTYAWFGNTITVESERCV
>BdioOR65
MYITIVLYDLLMHVRDDWLRPKTEEERVIMKEKARTSRLVSVTILIVSQMTIYSYLIAFLYNRVKIMRMANDTIVKPYYCPSKFFYDSSDGIAYELHWILQFISVFFATMTF
ASVDLFFADLVMHLCGQLIILSKRIEKLSPALFDGKGQTVETFLAEIVERHEHLNRFAGHIEDAFFIILLLQLVISALVVCLTGFQLMLMTSTIQGMSISDLLFMASFNLLF
LFSIFIYCFTCENLRTESVDVRAAAFRCSWPELKPSQARCLILIMIRSNKAYEITAGKFAVFSLELFCSVLKSCMGYISMLAAVRVRVDDE

>BdioOR66
MFESTIGLVTVFGSLAQCTSLYFRVKSVQNLYDRIKIDWETITDKREKIILKEFSRQGYVLTFIFMGCGIIGYIWFICLTYLPLFLDKNIANDYSKIFPYYSHDWIINDNYRKL
QVFVHASLGCLYAGLLFITVTTLYICNVKHICAIYAIVGFRLRKLLHVGNVRILVKMKNSTRRSDDKIISELLDTIGKHKEAYMGVRTLNRVFSRMLFTIEIFLLGCLAMLI
FDFTYHIEKSMLLSSRIAVMTLLYVGFVFYVNYAGAQVIEYSNAVLTSTHYMQWYLMSTDIRKILLMIMQRSLKADTLDAGL
>BdioOR67
MEKYFGTEINICRAVLNTFGLWPATNRKVNLKIILRFTYLAVVCLCFCNIPQTVKLFESAGNFDEMIEIMTNCDVTMMVAFIKALNMWFNREALARLLDLMLEDWKV
AKTTGNNMIMQKYAKLSHLTSIILFWVCESAILAYLILKSYNRFKESQTFRDGHVNLSERIRPLYMPGTFPYDVQKTPYYEITWICQIMSSVSTGSTFAAVDSLFSALVLHL
CAQLKVLRGQLRDLLANSVMKERACNDLDLSVIVERHKCLIWFAETIEDSFNLQLFAQLISSLLIFCLTGYQIVVVRHISFFFYLSSH
>BdioOR68
MQENALIARKMSRICAILGFATVHGHLTIRIGQELELLPGKPSTRLPFIDSYFPYDYKPTPIYEITWILQYAGAALATFAYSGIYCLFVALMLHLCGQFAHLKERMVVLVD
ESGDKFETKLAEIVQRHDKLNNFAATIEKIFNPMLLAEILGCTVQFCMQGFLLITLTTSNEMGFPVLHLLFMAVYVMHIGTHLFICCYVAEKLRDESSSMFRAAYDCQ
WYRLPPREAALLIMVMHRSKMPLKITAGKFCSFSLSLYAKVCIYYDYINLCVAYYNQQNHFQIFKTSGGYLSMLLAVRDRLAM
>BdioOR69
MFHDWRNVNDHRESMWENVRLCKLIMTGYVILTYGTVVVYALGMLILLQSHSSELDTGIINSSTSKPMFVISKFFFETDSSPTFEVIWLCQFVTATLSISAYTSFDGFFIFS
ILHLCSQLSILRTRITMMNFQMQSKQFVFAKVFKPIVKRHVHLQNLTYCIENNFNKVFLVQMICYSITLCLQSYQLVNCLTEEMENTAATLAFIVIFTSANIMSLFMYCF
VAEKLQKESTDTFYATYDVCWYELNSRESISLMNLMNASKLPLKITAGKFATLSFAYFAKVLKTSMGYLSMLLAVKSKH
>BdioOR70
MKCFTEKKDIIRSMRANWLLVMREAEPHNKKLMLSATMKAQSRSNMYFFVVGATAVLYITMPFLRDSEFRDRKYPFFGRYYFDRDSDLIYYVCYLSQMLTGTMIGLA
NYVTDTMFLVCAYHFCAQIQILHNDLMDLGYDGTPVAHSQLSNLIKRHQAEIKNAQALQSMFSQSSLQQVLLSCLMICMNGFKLIISLSNQDVDFVMYLTCLMLTLL
QILCYCQPGNELIIQSQSLDEAIYQSAWIDMDKSSIKNLIFMIQRSQRPLAITAGKIYVLSMENFMRIVKTSMSGLSVLQAVHRRDG
>BdioOR71
MKTVHSRIANNWQHIEDYCEREILHKYSERARTMTIFYSAWMMSTGVIAGALPLFSKILDFIRPLESPRPNIYVSGGEYFFDRETQFLPIFFADYIAIMLLVSVTTAVDTM
YFVSVEHCCALYTIVRSRLLALRLKMPKNSPETEDIDSYEWLRRTIQLHQDALEFTSLLNDSYYMPFMFTMIAGFHTIPMNSCAIIANLNNPLQMLPYAMILVAIMIHGF
LFSYAGQKIIDHQLSLLDAAYDNKWYEVPLRTKKLLLTFMLRCSEPVIITAANLLPINLEVYFKIMKTAAFYITVASSFR
>BdioOR72
MDVLPHHFRTLRFFGIWYESEKQHWLVKYIWQPLNINILVFFAILQLGALSIYRDNISDFTECLFLSLTYVGLCFKLINWLSRHDTMNRLLDEFRARICQPRDDSERDIL
KKYKRVVALVFNIHMGFAQFNNFTFAVAPVASMLAGNPEVFLPFKTYEIYDKNDSTYYWVTYVFQVMANDIGIFVNISFDTILAGFLLLTCGQIDLCCHRFLMANGKE
IKRSVKECVCASDVDKEDREEVGIVFYVRRRAADVSESDHFLYQYLQYFSDKHQWGRNGHIFHVSRVSTVSTLLLLLVRK
>BdioOR73

MVIQMRKPPHYRTLNEVDHQVGYSLLAVCVFNSFPYIPLALDAMMPLENGSRPRLYPYHPDYFVIDAEEYYFELFTFHGIFVVAFATLAPAAIDSMMVANIIHACALF
AVVCHRLENIGKLSESEKSGSRLADDRLVNRQCKKVCQLHLSSINSVGQIQDSFSMSFLIVMGAGMVAIGMLMFDFLYSLDPFDIIRVGLIVVLMFGIQVCLFYMNWV
VQQVINSSEEVFQYAYRSEWYNLSVESRKLVSIIMQNSMKPVALSAMSICDLNLQMFAAVVKTSGSYATVMLSMQNT
>BdioOR74
MEALDIYDSYYFRINKMLLRFIGLWPYDSKNKKMVTLSWMIFILNFPHIAGIVVHLLNWDKLLEHLCIFFILAGLFLKYVTVAIVEKKMSSVYNDIAHNWLMITDEGE
KRILVKYSKKGRTLTTAYMSVLASGAIFFTCLPLVLEILNVIDPLPIPRPHIFILNGEFLVENPNDHFIMIYLWESVSVILTCLICTAIDSMYMVSVEHGTALYAIVSYRLKRL
QHRKINDSQDATNSSYAWIIHTVKLHQAVLSFLENINDSYSLNFMAMTVASFIFIPAACVTINNKPYIVMR
>BdioOR75
MTRIDHQTVFDDVSLPYVGLLRIIGLLPFKDRDYSFLGTRLLLLLFVGTSIINWTMVIGHSYTIRAVTVDTVCEIVLVFGMHLRYYVILSKRPRLLTMMKECRAIWTECMT
ERGYRAVCSFERQLRVLLILFLFTFSFITVLYTSNAMQQRLPALEPNGTGHRMLPHKWYIVDAEAHWPGYWITFSIQQLVVNCSSLITAGCDTASPFLMTMMSGYLRYL
ADYFHSSGELESACTHRHYNEFIRYVKLHQRILTYEPDCIVEKFTCVNFMLVLDSARSWKILAGWYFSYK
>BdioOR76
MINLERGYNLMMICLKVLGVDPSASEEKYRRLSSFLWWFYLLNYVALLLPTLYTFYSAMNDDSRDLMMMSLTSIELRAIIENIVILLNCKLQMKQFRMLLRVMRNSVV
EQNSATIGTSLVVKYIKICLLIPTMYCGITYLYINEIEWESEKKLITSAIYPFKLDSAVKKVFVLINQLVIAVQCSSLYIFDGIVALLICMCTVRLKILKDKLKTFEDRNFKQLV
REHHEILKLIDDVNGFVGILIAKTAFSFVSSTMTTVIQWNRQRTMNELTLPFLMIVVFGVRLYIC
>BdioOR77
MRERAARARLITVADHAVILLSYLGFVLLPLAHFELRSLSNLTDHGPRHLLLQSCYPYDYSASPYYELTHAAQHVAGFFITVCSTVPENLFGALVFHSSAQFEILGLQFR
RFFDLGSPARPRHTRRKLRALVDRHVHLMNMVTGIEDSFTYVILAQVACTSVMICSAGFQTIALFGGGHNEPSPFQLVTLLGIIFTMMMHTLLECYASEILASKSQGVS
TCMYNCRWYAVSNHPILRDLIPVLRISQIPRQLTAGKILTLSLPTFCNILKTTAGYISMLVAVTTR
>BdioOR78
MDIFDSPHFRLNKKLLRVLGLWPYDPQRKIIRICWIIVTLVMTAPHVIAMIVDVSEVDKILEHACIVLIVAQGFVTYVALAYSENEIKTVYNNIANNWLEIVNDEERRILF
EYSKRGRLLTIIYMSLVMTTGAAFMVMPLVPEFLNAIDPLPVPRSHLFILNGEFLIDRDKNFYKIYMWDTASVFVVVTLFIGMDSMYTVTVEQGTALYAIITDRLSKLRG
RLLSDLQGASDSSYEWVIHIIKLHQTVLSFMDNISNSYSRCFFCLSIINVVHLPITCVVVRKKL
>BdioOR79
MIMSCDWQSISASHLWYFRLFALVPFEGKTFDYLGTKLLMAYLSFFNIFYITCYSFYSTVKLSQKVFEVDYICETITVYGNVFRFFTLYISRKHIVKLVDDSCELWSFLRSDE
DAETLKTFERKARMSRNFLLINTLSVVTIFYITFFAMIAPIPKEQWPTVFRYYVEVHAEPWYTTVQILQFIMGYTIAVQVAAVDNVGLYLTMMACGYLRVTKHRILGAI
DQLKDEETLHDEIVDCVKFHQQIMLYCQGIELTLRKYFLVTILTTVYNVSIIGVKLLEVRRR
>BdioOR80
MADDYETENSIERWSNLISTSHRFSKFDYTLYLGNSAMYYLQLILNYPSTPVEDREMMMRAHYPFDYKSSPIFEIMTAIQIVLGLVMCLLQALSESLLVALILHLCGHIDL

LLERIEEFSMSCVNNPTNTSLVLVIKQHLKILNLFHKVDKVYTYASFVQVFLSTFIICSVGFITLTITDDIVIVVKFFMFGISVTWQGYSFCFFGQRLINQSEKISNKLYEALW
YNADSKHVRDLSYIISKTQKPLTLTAGKFTFLSAQTFTTIMKTSFSYISVLRASIK

>BdioOR81
MRAIGVWPLDDSQTLKTRCKFLLPGILLLFFIIIPQTRKAIQARNDLNLMLEVLTTADIIEGIALLKLLGMWYHRIGLKKLVREIARDWDRTSPGEQQVMWSNARLSK
WAAIFCYSSSTGSVITHSMVFLITTTKDYQNSSIDKSLFLKSHFPIDTSHSPTYEIISLCQCIGALLSCAAFSSFDGFFVCSILHFSGQLHNLKHRFRNLVTKGNTDTVTFTKL
LKLAVLRHQHLISHTDLIENSFNQTFLVQVFATSIALCLQGYQFVMVNECKTHSAC

>BdioOR82
MVLVLGIHARYYVIVKRRAKLLAMMQECRAIWHECINEEGRRTVSAFERKVRVLLMILIGSCFFVAVLHNGNAIFRKLPALEPNGTRRRTLPYKWYWQNVEEHWPG
YWITFSIQQIAVNGSLLISVTCDTVAPFLMTMVSGYFRYLADYFHSRAELKSTYPDQENHELIHHVKLHQRLFKFCTELEQFGRLIFFIQILTTGYNLSLAGIKIIGVSGHS
TITGKFIYCLLTRGCEKCIFLQIFIIHKAHLSRSSCTSNVVIVPPVYRRIRTSTNSYPSC

>BdioOR83
MFKQARRGRLLSAIGYITMYTHLTLFLVGPMIDLRLRIINNITDPDPRRFLPVSSYYPYEFYKSPYYELTYLNQVMCLVTSATAFSTFDNIFSVFIFHATGQCEIVTQMMYR
LYNGTGQMKFVDRIRELIDCHNRLIGFVKAIEESFNFIILFHMLNSMMLICSVCYGYVKASNGTPSVVDIVMFATALFNFNEHIFIYCMTAEVLASNSLEMFNSIYGSNW
YELPAKEARYVILILQRSRHPLRLSAGNFFYLSLNSYVQILKSTFGYVSMLLTVS

>BdioOR84
MTLECCLFGLFVFSLFLKLVLGSKDQAKRRQIYVRIAKHFESISDPEEVAVLNEGAENGFKYTLVYICIVCVLVGIFDSVPIVEAFYDYLFVHGELRLQLPLPMNFGFDHL
KYWYLITLDYIMFIYSGILVDLGYDLAFMLLIRHVKALFDIVGIRLRKAGEINDEFENDVMSEDAANEAYSLISSAIDLHSECLKLIDLIEGCYTMGFFVILLLSTGCFGGSI
HVISTKVNDPVETFRYFSVFLLVVIHFQVVFSRGQLIIDSAEGILDECVFTNY

>BdioOR85
MDILPYHFRICKIFGLWYDNSHYLILKSIWRCLVFLMLVLFNLSQIIELITGRGSVEETVEVLFTTLTYILCLLKIFNFVLRYNEMYDLLEDFRISLCQPSTKEERKIFKDYSSK
INYVSLSYISFSHFAGIAMLCKALLSYYKSEYNLPFKVYQPYDITIHRYYWISYSIQLFAIIVGITLNTTMDTIGYSFIMLATCQYEIISSRYRNFTKPIKDCIEHQVLLQEIVLK
IQKNFITIIVPLFSSSLITFCVSIFHVSQVRILLAYYVHTLILCL

>BdioOR86
MQSNVRTSKWLLRFLIFIMFGTMIIYDVEYLVKFGANTVRLNDTIYRPLYLTAYFPFETHESPVFELISVGQCIGSNLSAFAFIAYDGLFGFSILHLSGQIYNLKIRMRNLVQ
EHRITGVPFTRCIESVIQKHSHIVSHAEVLEESFNNTILLQVFTLSVALCLQGYQFIKVFFSFLFHPPLPMIIQKYRLSQSLYDRIPKLTTDMNVEPALLIFIIFYTLTNLNCIL
VFCAIAQVIRDEVSRKFIENPFASMLYFFSESKSVRRDLPDAVVRVTP

>BdioOR87
MISFPNHAWKILIQLEKLAGYWPYGSKIKSSILRICIILVLCSYIIPLVLRIIKEWHSHFYIILENFLVISIIIIVTTKILVASLFQNTRQRMVEQIKSHFENVDDEEEVQILNKHS
EKGWKLSIEYVVVAVTCCGLYDIMPTIEMSFDYLFNNGTIEKKFPLYLDYHVNEEEYFLQILAHTCITLTVGMIAEFSFDVTFFITVQHNCALFEIVKHRLKKTSDIFDKS

DKNEPLKDVDEKRAYEIVTSAVDLHNEALTLAKSLNAAYNANFIILLF
>BdioOR88
MHIFHRKISIYFTTAVFIAIILRCIAPLVDQSGEKMLPVDAYCPCDISNPGCYWLLFWHQSVGTGMATLVHAAKDCLIIALLLQTCAQLEILKHRLLSIAETSKEAHAKN
MTHDQIALLEKQLIADCILDHESIFEFSTILNKSLTVMLFGQIAVTLPNLCLSIFLLSRQSIASVEFMMTTQFFSAVVIELFFFCWYGNEVTLTSLDVESAIWEMEWETLTN
SAKKDLLYMMVRTSKPILFRVGPIMNMNIDSFLKIMKSSYSAFSVLQSTGV
>BdioOR89
MLRKPVEQSEGWKDLEWSLGLNRVALRMLGVWPEERHLAKEPWSVKMRVPLLILVTLLWVGLPQMCALYLVIDELPLAIDNFVTNCGSVTACAKYLFLWRNKHGK
CFSSLGVALPIAGEPWPARIDRRGLAAFRRQCLELDCRSAVAGDRFGEERLAAGEARLGARAHDRARGPRQALHRGRLLAAGRLLRWLYVPAPARLQHPHRQQHH
RPRPAPPSLSDLLPLRLLAQSVLRADRAPATVRRLLHRPLRLHAGQLLWRAGLSRERPVGDTR
>BdioOR90
MRIWRRKSVVQNVASHSESNFSATKHQITTTRSADDNLETYFNLNIKMLLAMSIFIDLDGKTNFEKFGIFAKIPILTVITIVISVTGAFDQFVWMVQASSYDRQLATTLST
NIFSNISSVSKGVCLSVAAKDLQSILINLSRMWNTYRPEAECRESVIKRAKKTITFVKGYIWMTVALIANFSGPPLQYIFFQYVGLNPTNTTFDYSIRIFLLKYPFNVDSSLV
YYSVLCHEFWILFGLTLCWVCCDTLFASLTTHLSIQFEVTENDTM
>BdioOR91
MTILFLYEALINIGCACVYLLPAIPKIMDIVSPLPNPRQKIYILESELYLDRETHYQFYYITEVVTSIIYAISGASIDVMYFVCVEHCCALYAIVNYRLRELRSAMINSSTNTEI
DSYDYVTGIIRLHRIALNFTTTVNEAYSMLFLFILITNMGAIPLDALTVISNLDQPFNCARFGILFLLFGVHLFMLFWPGQNVLDLSSELTDAAYCNEWYTVPIRTRKML
VIFMIRCTQPSSMDAGGLMVMNFETFSTIMRTAISYITVAASFR
>BdioOR92
MLTLKTVGQENKRYNLKCAPFSPFIYVCPIHLYFYEKYLFSSPLRFLIFYSKNEATFHLSSTTTFRRNTRILCMTYTVSVISYAYMPFFISDVEDRVLAYREWLPYSLDNVN
HYYLTYLHQSIAVTIAAFGNAVAETMVSGFMIQICAQFAILEERFKRMPLVLNEMRENQNSEADILSAERSMMARLIKHHLRIFELTEILNDIWVFVILVQFIVSIFVLCV
CTYTLAFMKVINSDFLSILLYLFCMFMQIFLYTWYGNEVTLRV
>BdioOR93
MDYSKDCPYYKINKLLLICFGLWPNQSNFSKYFNGFIFAIWTTTASVAIAQGLTDETSTDPVTFSETLVALVTIIGEIRQWTYLHFKAKMIKRLFNRIEHDWEAATDPDEV
AIVKKFSQKANLLMNIYYATGLISYTWYVSLTYIPQFLNKSNAKDYSKIYPYYSQLWIVNENFRSTQIFVHANLGAFYAGYMNLTSTRFYISSAMHICAMYSIIGYRLRNI
VKKAERVEEDILNQLLGIIAKHKEVIKSAQILHELYSNNYFI
>BdioOR94
MDIFKSRYFNFNRQIFLVMGLWPCRTRLIRKYALLSVKVVIVIPILVFVLQVVSMIVNFHDLDRFINNLFFFLVGFHLTLKVVAMHIAEDEVCVYFYYRIEFFIIINLQMQK
VYKSIADNWKLIVDEKEKEILVEYSERGRKKAIAYFSYIESVAFSLFLSPTIPKILDIIIPLPEPRPRIYILETELFLDQDTYYVTYYLVDVVSIIIYAAVAVATDIMYIVCVEHCC
ALYAIIKYRLRNMATMVSSTGAKVDCYDYAAIKLH

>BdioOR95
MGLWPYGPGKRNIRKYILLLIQFMLAIPPLISIIVNISDIDRVMLNLVAFFIGLLMFFKILVLIVAEEKMKGLYRNIADNWLAISDDKEKKILVHYSDLGRKKTLFYFGLVQI
GTASLFTLPAIPKILDIMRPLPESRQNIHILESELFLDREIYYWLYYLWDVVTIIIEGTISVSVDTMFIVCVGHCCAMYAITSFTATINNAFSMLFLYILIANLATIPLLAVTVIS
NLDKPFNSIRFGMLLILLAVHLFMLFLARTKCS
>BdioOR96
MLLITAATFMVIPIVPELLNAIDPLPVPRSHLYVLNGEFLLENRDDYFFQIYLCDSASIICVIVLFMSIDPMYMVIMEQGMALYAIITYRLLKLRQKTVDVSQDKTNNFYE
WLVHTIKLHQSVLSFTNSIEDSYSACFFCLSIINIIHLPVTCVVVMVHLDDPVNLIRYGLSLASLLIHVFILFWPGQRLIDHSTRLLDDAYCNDWYITSAKTKKLLRIFMLR
CSEPIMITAGGLVEMNFEVYAQIVKTAASYVTVLASFR
>BdioOR97
MHYRAIFDTVPVLRSFYNYFCLHDELNVQLPIQMDYGFDHKKYWYVILLNFSMFVYGGILVDVAFDLSFILQIRHAQALFKVIEVRLKKASELAISEYEGESMSEGVAD
DSYSMISKAIDLHSQCLELLDLIEETYTVNWFLILLLNTASFAGATHLIFLRLYDSIETFRNSFIYVLFLLHFYVIFSQGQTIIDSSSAVSYACYDCDWHCMPKRSSRLLTVM
MIRSSIPCEITGAKLFPLSIETFGVMLKAGISYYTVFRA
>BdioOR98
MSTAAITGPTFPLIPKLLDIVKPLENPRPNFYVLEGEYFIDRDTHFTLIFFMNYITMMLIVFVVITIDSIFIVSVEHCCALYAIIRYRLLELRRIVQGSSSATENGDSYEWLRRT
IQLHQNALNFVTLLNDSYSAIFMCVFIATLYVIPLNSCAIVANMHNSIHLLPFVLYFVGIMIHGFCMVFPGQRFIDHQLHLLDAAYSNEWYTTPLRTRKLLLIFMLRCSL
LTTLTAGGLLPVNFEVYWKILKTSASYVTVAASFR
>BdioOR99
MDAKTVKNEDGKAPLLFDEMCRMYINVHKLITILPFEGCKLSFIGSRLLTGYFVFFSIIHCTMFVVYCYNASQLEVEVICLTVLVLGVNSRYYMWLFKRKAFAPLLEEC
RDLWNFCDEEGRLIVSGYERKLRRVYMIFYTNAIFINVTYAADAVMTRLPPLEPDGSERWALPYKWYADNTEDNWPGYWVTFCVQLLVVFNTITYSVTCDATMPLFA
MICSGYFKHLQHRLLASQANQDPILRKRELVHCIRFHQKLLK
>BdioOR100
MYHHIEDFWNISDDPQERKNFERLANVAKIVSIVAFLSGIADGLGFEIVALINLIRNHRQAINGSVYHRPRPMQLFYGLDTQRTPTYEMIFVWQSVSMLYSILTINGSDTI
IMVYIVHLAGQFKLIGARFRKVGRSIDDNYDEIHTEIGKLVAQHQNTIRIWKELNELLSLVMFCQIMSSGLQICFGGYAVIKGDSSKAQIITFLAFFVSLLIQLVEFCAPAE
LLIQESSAIAETIYHEMPWFELSPSLRRDLGFVM
>BdioOR101
MFPGVLPAVLDLVNPLNESRQKFLCFYGEYYIDQQVHYYKLFFHTIVCVLCTIILFTTIDSTYVNCVQHVVGLFGLVDLNLKKALKLTENSCKSKALELKMHKYVLKSIS
LHIKSIELSNKRKKLLIILKYSNLQKCYWYRFVDLLQSTYTTCFFLTTGVVLIALSLGTVDLMMSTSNTVNFLRVMFAWCGIVVYLFYVSIPGQRITDSSLAISESMCVYYY
SFFNLKEKKILNRTFFIQFLFRLARISSGDRKTY
>BdioOR102

MTNTQDPCVMFDKMVGIYLRTFRWIAVLPFEERDYSFIGTQLLTFFMICVSIVHWSMITVYCFTASSVEVADICFMAAVLSLNLRYYMMLYKRKTFASLFNKCRALWSF
CNPEGQQIVMGYERKFRVVCGAFFFSCCAINAASALGAIVTRLPPLEPNGTERRILPYRWYAENTEDMSPGYWPNFCIQVFVNLVASVYVVLCDSAISLLAMICSGYFR
HLKDQLSSFQRDTGTRRRELVRCIIFHQKLFVCVSMHT
>BdioOR103
MEFQKGVEYQLFSIPFTVLTLCGAWCPDGWSKKEKSFYAIYTVVIMLLGIVFFTEIMINIVLTFNSDEFNMENVFTAIVVGVGIYKKVNVLLFRSNIMYFINKYATKQWH
KPANFEEGVIYSENLSERRRVTILYSALILAAVMLRATVPILEARTFLILPLEAWYPYNVDNIIGFFLTYFHQIISGVTLSCMHLSTDTLFVSLLMQMYCQISILKHRLQRVG
ELSLEKGFTNEEIKSLIMQRVREHESIYR
>BdioOR104
MNSVKIHQDFNQAMKLIRLLLLPMGLWPTTSEIYQRFVRPVAITVCLVTMLSMVISLCLFIALPGEDLKIRLSFIGLLGFGLMSLFKYVAAIYQVRKLAECIWNMSLSWQ
ELRNDRERKIMTKNAKTSKLLIVACVLLMYGSGMPYVTMLPLSKVPVMRGNDSLWTLTYPGYLFPNLQVKSIYNLIFTLHCMCGLVRYTVTCGIYSIVVLFIMHISSQIT
ITNMMLDKLVGDYKIFGQIISQHRRLFK
>BdioOR105
MITDPNHKREFPFPMRIESPLYESPYYEMYFALEMIATLAVGLCVSTFASYLFVINLFTATQFRILNMHLQSLCDFEINSNYKKNLQHQKEALEKLKSYVVKHQMLIGLT
KEIEDLYCYVMLEQVFGSTLEICFCGLQILLARTTLQRTILSIEFLLSSVSQLYFYCYSSHIIFEASESVSQAMYRMKWFKMTDPQVHKEFSQLFQIITIRAGRPCILTCGKFY
PLTLATFMSVSNSEYTYLEKLR
>BdioOR106
MKIKLSARHGFIILSICMKLLAIHPQPINEKRYIYLARRGLWWIYFINHLTLLPLTVRTIIQVSDDILSVSYSVMELTGMLESLIILITFKNQENRLKALLLFVKDQLIVKKLT
ADLDNSFMYVLSFLSIAVIYTTVMVMYINKPDSQVDGRVLMTTTYPFSINTTVLKVLTYFNQMLQLMHTSVILVSDGISVLILYTCTIKLKVIENEIKETENYCSFTENIHQ
HQIVLMLVKYLNLILNVI
>BdioOR107
MVVFGAFVKLVAINWFQGPRRRIYEKVFNHFNEIIDMEEAEILRDYSNKGYRYFTIYMYSNLAIAALFCFSPFTLGGYNHLFSNGKFFRNLPIELNYGAASDEYVLLILIS
LIEAGHSTCFFFPLLINTSISGALIHVISVLLSDPLEMFRHSIIFLLLLIHFYMIFSQGQKIIDYSAGMFQARYNCKWHEMSKSMRTMWIIIMIRSSRPCFSTGGKCFILSRETY
SKMLKAGLSYYTVFENK
>BdioOR108
MCRAFGNRTGKQSFYPLSMDIYDERYFCMNKRFLRCIGLWPYDPISKIVRALALIVVFIFTIPNSVGLVINFHNLDLLMEHLYFLLFVTAYFMKIFAIGIAEKQMKTVYVR
ISENWIMITDINEKEILRKYSERGRIITIGYLVYMITSLVFFVSLPAIPTVLNIINPLPQPRPRAYVFQSEFLIEDKDAHYAKIYIWDVLTGFFVIFCFVATDTTYMVVVEQYLA
LYSIVKYDIPFLLSSG
>BdioOR109
MEQLNLYLFRRNDRVRRIKPNRRTVVSYFQQRRWQLITLLEQLSGFWPQQRKLQQYPLQLYHAFVILTLFFAEANKLRECIGVDPDMTIECCLLLFVCVGMLVKLMFA

SIQQYNRRKVYKRIAKHFETMRDSEEATVLSEGAERGFKHTLYYMYENDVGTICALLVTFVSVPVLRAFYKYFFNHHELKVQMPVPLNYGFDHNKYWYIIALDFLVYF
FSTLLVEFTFDLSFVLQIQHAAA
>BdioOR110
MEDEILTSYANIGKLLNFLYMSAVLSTLVAFMCLPLTPVFLDIILPLNESRPKKPLILAEFFIDEEKYFHSILLHAFFTAYYGIIPLLGADTLYMNCVYHACGMLKILGCRIES
SFRETHENEELEKENYQQIKTCIKLHQEIIEFCDDINEAFSKSFFGVLILNITLMSISGISVVINFGDINLINDVIKYGMFTVSQIFHVFSYNYMGQLVLDSGEKIIENIFFTDT
IQSGMKHR
>BdioOR111
MGDKDENQFKFEAYFELIVKVLVYLGIKIDLTGDGEVERSKISDTIPFPIWSFLILGTFSVAGEYFYIQDSMKKNNMFLAAQVTSHLLSNVICIAKGYRLVFTVSDLEGTL
QNLSSLWRKYLHNHEIREKIFKKANKTLLFCKMFMYATFSAVLSFCLPPTYNLFNEYRHQKDDNYTHDFEQRIFFFKYPFEVNSFPRYLSIMLEEGYLLIGAGLMWVAS
DVIFAYRRGLDVGSKRR
>BdioOR112
MDYGFDHKKYWFFIFLQSSLCAYSGTISNIGFDLSFILQIRHAESLFTIVRMRLKKASELTVDLKNDDLNDDDDPYTLILSAIKLHVECFQMVRMIEKTYSNAWFVMLLL
NSAFFGAILHLIITKLDDPTETFRFFCGFLVLTMHFQVVFSHGQKIIDSSNSIYDACFESKWLELPTKSRKLLTTMMMRSTKITPITGAKIFPLSIETFGNMFKAGLSYYTVF
HQQRPYRFDT
>BdioOR113
MGRETRLYKRKLADFNWAFGLNRLSLQLMGIWPDEAVAGVRDGEASAKSKLPRIPLMVLWMVVGLFLPQMYALSQVFRELPLLVDNLTTSCAALTSCVKLVLLWNG
RHVIGSLVRSVARDYSRSRSARDEEAMARQARRARLFTISGYAIMFACFVGFVTSPFFGLSIRIVNNITDIAERRYFPLPTYYPYDFVTSPKFELTYAALLIAIAFSGMSFSTA
DNFFGVLVF
>BdioOR114
MDYGVDHNKYWFCIFLQFLLFVCSGVVSDISFDLSFILQIRHLQALFKVVRMRLKKASELAVDWRNNDLNGNDEPYAMIINAIKLHEDCFQMVKMTEKIYNVAWFVI
LLLNSTVFGTVIHLLDTKLDDPFETFRGLCGFFLLLLHFQVIFFHGQKIVDSSNSIYDACFESDWLELSTKSRKLLAIMMMRSTKACEITGGKCFPLSTETFSKMAKAGLS
YYTVFRKQR
>BdioOR115
MIKVANLGRSMAKGLTMITQFNVMVVNLSLQLYFNIAGIYPTKRMYFKSYYPYNFSATPIYEITWIFQVIASSITAAAYTSVDSFIGFSVLHVCGQLAILKNELEQLSGSD
ARDNPHSLEYIKQKLRYITQRHDHLINFAGTIEDIFNMMFLVQMISLTIQICLQLFQCINALSSEQGASLLQFTFVGLFMCCVVLLFYVYSYVGEQLIVQTSNIRNAAYEC
EWYDL
>BdioOR116
MNIQNSPWYEIVFLVQFLSGKVSCTITVGVCSLAASYVVHVCSQLEIVMRLLDDYVRGNDAGSRGKMKEIVEKHIRALRFAMRVEKYLNPLCCIEFVGCTLNIVLLGY
YFITEWESRNAISTLTYCTLLMSFTFNIFIFCYIGEILTEQCMKVGEKTYEIDWYKLPGKKAEDIKMIMIISSCYPAKITAGNIVYLSMESFSNVIKTSATYLNMLRTLML

>BdioOR117
MNYGFDHKKYWYVLTMDYLLFVYSGILVEFAHDLPFILQARHVQALFEVVRMRLIKASEFVDNNENEATGDEAVDESYSKISSAIDLHSECLEIVEMIEETYTVNWFLI
LLISTGGFGGGLYAVSTKLHQPLELLRYSLLYMLCIMHFQVVFSQGQVIIDSSEKVSEACYECKWYGLPKKSCKLLSTMMMRSSTPCLLTGAKLFPLSMESFSKMLKA
>BdioOR118
MSENVVESTKGLIFAATYLDLIFKMFNFVLRRSKISDLLEFFRLMQWVPEVKEEEAIIKNYRRKIANLYWWYMSLGLSCGVSVILAPFISFEDSGFRLPHRVYTPYDISVPI
HYWISYSIQSFAINIGIIISISMDTMVYTSLMLITCQYEILHDRFINNRVSIKELVKHHILLRLTVLKIQDLFARVVAQLFACSLMTLCISIFQISQVNSICSEF
>BdioOR119
MTSRSVVAMDPCDMRIFEGRYFNINRRLLMCLGLWPYERKSKIYAIRIIAIGFLVSWSVPQLIDVIVHNENVDRQTLGLSVFLSLWIMILKIVGMFLAEKNMKEIHRRIA
NNWQLIQDANEKDLLRKYSERAQTVTIIYSAGMIGSCLFGCMTPVFPKILDKIMPLEKRRPDVFLTEGEFFIDKRAHFSLFFILQYLGVTLMGLICAAFETMYIVSV
>BdioOR120
MHFYLRYVLNPDYRMYIFDAYSFYDDQVSPNYEITWCIQFFCTFGSSAGFIGFESLFAIIVLHLCGQLTCLRDRIGNLTRDGDYDYEVLKREIAAIVERHNYLYSAACEVE
NIFTSVHLIQLFNFTLIFCLLGYQSVTMIKEGEANIVEIIFCIGFFVYLSSNLFVCCYASEQLRSESLAVEFATYATSWYNMKPKEAMLLMNILMRSKIPFEITA
>BdioOR121
MSTSLLELFSHRNNQDFEFSVLKICQGCHSWSPLTYPVVTHFRHRLQKPNKLAHDDLPGADVGYETMTNIIQLHQRVLEFTQILENSYSQCFLALIIIHVICLPTSSTCII
ANIDQPVNLFRYALAFMCLTIHQLFLNWSAQSLTDASTDLFENGYCSEWYLNSLKAKKLLMVFLQRCMRPSMISAGGLVNMGMETFYGITSKAISYITVFASFR
>BdioOR122
MDEPYKLLGYDTTFAIQTIAIQSGITCAIASNAFVSIFAMLGAGYFRQLADYLADLPEQIDRNEILSCIKYHQRFLQFSNNLNEFARTTMLSYMAVSCYTMSLVLIKLNG
TDPGKYSHLPILINCMFQLFLIQWPQDVRSDESEKMAYATYSTMMKRLDDKRLCSNLQITLIRAQHPMYFHAGGFIILSFETYTTMMRGAFSFFMVLQSLDVQ
>BdioOR123
MIFAFEVVSGVYAVTVTVGCDSLFGLYALNLVGEMRLLTHRFRNLKSRNDYRNELKECVDRHVVLMEAKNVMQRLFGLMSVLLAVTTAVIICANIFTLTLNKTIPTYK
AAFLIDYIVAKLFQAYMFSWFGNSITVESEACLNAIYDAKWAGSGDIRLMNDILMIRSQSPIHFETMSIMKVRFDMFSKILNTSISYYFLLKTVAEKLSNKAN
>BdioOR124
MMTSLIKNWNSNFVIALENFIELIPPSLLAMKIITIFFVEKKRAQILEQVARHFETVIEEDEVKVLSDFAKKGLMLTITSTLCILAGISIFITMPYVRMFVHYFFGDGELHKF
LAVPVDYLVDEKKYFYWIVLEMLFTLVVSLAVQLSFDSTLFMTVRHGCALFEIVKLRLARASEIFEKYAQNKKVSDSDEEKAYEILTSAVDLHNEALE
>BdioOR125
MSMIVFVTYDMSFAMCVQHVCSLFDVITLRLQRASQCACKQSASVNYTDADRQVFKNVTEAIELHKVVIKNVNILEDAYNTCWFFILLMNTVGLGGVLVIVLENLG
VIEQLIRYGIFFLMIFIHKFYVFSPGQNIINYSLLVIENCYSSNWFQMSPRCKYLIRFMMLRSIRSLELTGGKMFILCMATYAAMLKAGMSYFTMLANTRETE
>BdioOR126
MSDSSRNLTLMSLATIEARSMLEYIAVLVIFKCQTSRFKTLLSLMRNELIGKELLTTSFELVMKYIKILILISMMYLIIVVIYFKRFEWQSEKRMIATAIYPFKLDTNLKKILILI

HQLLLIVQVCSIFLFDYIVILSIYMCTVRLMLLKQNLEISEDTKLIQHIREHQEILKLIENVSEFIVIPIYKTAFSFIGSSISAGIQLTRQK
>BdioOR127
MHIGADTMLSGFMLQSCIQIKLLKYRLKNFLRVCKQYSRNPYTEGNRKNIETLLLKNYIRDHQFTISYAKTVNIDFSGWLLAMLAVVVPNICINVYLLSFCEIGLNVDFI
TSLGLFSISLFQIYLPCWYGNEVTLQSMEIGNAIYDMDWVDLSPTTRRSLIIMMIRAAYPIKLTVARIIPMNIKSLLSIIKTSYSALSVLQQV
>BdioOR128
MQLIVAFNGAITATTVDSAAPFLIMLSAGYFRALQCKLDRAYDKANSEDEVLADRELNGCWRLHQRILDHCSDIDKITQKLFFVQLMCTGYNTSLLGIKLAGNDPDH
WKYLPMFILNVTQMFICQWAPDHLIDESSAISTAAYRISFPWMKRRIGKLIALMIERAQRPVQMTAAGVAYLSLENFGTMLKGALSFFTVLRSFNI
>BdioOR129
MLRWWMGLIAANCFMGAINPIISHNPDNNLMFNSWLPCDRHESSCFWICWSYQVISYVHASITHVCSDCLIFNLIERVCSHVRILQHRLLLLPHLVSTGVVDSAEKER
LYLVECILDHQNIYSSAKEISKLFRAIVSVHFILTFSILCTNIYLLSMQPLFSNSFIAVCLFLCCILAQSLIHCWYGYKLTQIVYKLLF
>BdioOR130
MWKYARYSRLVATVGVCSLISDCHLLYLRFAVQYHFDQKMIGENETFIKPFCMSVDFYFDVQWSPIYELMMFLVYIAVNICAVSYAGYDGLFVLFILHYTAMFGNLRVE
IERLVEKRKDGKFLDVLVPAVKRHEYLCRCMMWKEKRIFVKKKMFTVVNVVSTSAHRVSRKYSATCSSCKLFAYPCTCACRDTNLSL
>BdioOR131
MTIECVLEMLICFGPILKLIFASIYQRNRRKVYERIAKHFETISDPEELAVLNEGAEQAFKYTLSYIGIIIVALTIFDSIPISRAFYDYFFTNHELRMDLPIPMNYGFDHNEYW
YIIAIDYIMWIYSALLIMFTYDLSFMLKIRHAEGLFKIVSIRLKKASGIADEFENGIMRDEDAKESYSIISSAIDIHSECFE
>BdioOR132
MADDWNASKSKADSDTMKKNAKLSRLTTIVLIWVFECSVVALLALNIFIQLKESKMFKQGKINASDRVRPLYMSSEFLYDVQKTPYYEITWFLQFLTTFLPATAFAAIG
GFFTALVLHVCAQLKNLRRHFEEVLSGAAMKEDKLYDLDISRIVERHEHLIWFADTIEDVFNIPFLVILSSSALIFCLSGYQIVME
>BdioOR133
MYVASCEHACALYAIISYRLRNLVRATCDKSARREDESYSMLVRTMELHQTALRFTSTISDTYSMCFLFFLVINVPLIPLDACAILSHLHTPFGLLLYVILLGMIVGHLFIQ
FWPGQRILDHGVNLYVDAYCNQWYAVPQPTRKLLLVFMLRCTQPASVSAGGLFLMNYELFGRIMRTAVSYIAVLASFRKN
>BdioOR134
MCLAYLTMGLPVIIRCVEEFFHRPMDLDLFLENLAGSIWYVGLFAKYLASILTVDKFRFVYAEITRCTEEISDDGSREVLKRFANRGRQLTVGYTAYIYFNVVPYFSSSIIIP
MTLNYLIENATYEKELAHYGEYFIDQDNYFVYLYIHTIVAGLVMISIIPGIDSTFVVVTHHLIASLYIIEHKLKKNF
>BdioOR135
MIQGLADETSADVLIAFETFVAIVTVFGSLTQGTSLFVRVKSVKSLFDRVKMDWKNVRNIEEKMILMKFSRQARVLANIFIGGAMAGYAWFLCLTYFPLIFDKNTTKDD
SNIYPYYSRSCIISENYRNVQVFVHASFGCLLAGLLFTSSTTFYICNVKHICALYTIIGFRLRRLLCTDTLKLKKMKKKER
>BdioOR136

MKYFISFSKMDADFAKRAAIDRPETVAEMEDRRSMLFENSAFFHRVVGLWPKEDQLFGNFRAWKCRAGIFSTIAIALMQYCVLAVNEDKSRRAELCIEAILSIAAFMK
LMPCILKRKMLIELHHKTQEINLKESSSEKKIAYKWLSLQTILITSIIISYLLVTAYSLLCTCFIHYTEVINSQTMSYCN
>BdioOR137
MMRFFTRIRLRKTSELAAGHENKNMSADAAAQSYTLMSSAIDLHSECFELVKLIEKSYTSTWFIILVLSTASFGAGIQLVSTKIHDPIETFRYISAFMVILLHFWLVFSQGQ
LIIDSANSVSDECYQFKWYALPKKSWQQSLMIMMIRTSIPCQLTGAKLFPLSIESFGKMLKAGLSYYTVFRARCA
>BdioOR138
MDRLMLNVLVVLSLLVGILKVVGMIIAEKNMIKLHKSIADNWQSTQDESERDLLHKYGERARTVVIFYCTAVGILGVIAGTLPLLPKLLDIVNPLEYSRPVFDLLQGEY
FIDKRAYFVPLFFIEYFLILLSIMVVITVDTTYIVSVEHCCTLFEIVRNRLLKLKCVVQKISSATGDDGHDSYEWLRH
>BdioOR139
MAILTKFSQQGKVLIIIYFASGVISYGWFLSLTYIPQILNESNTKDYSKIYPYYSHRWIIDKNFRTMQTFTHANLGAFFVGFMNLASTSLYICSAMHICAMYAIISYRLRNL
VKTNETLLLDKMIEGKGIFEELSDIIAMHKEAIESVQILHKGFSNSFFVAQIFSLLSLPMLIFDFQYQIDKSG
>BdioOR140
MVDFFIATMLWYCAVKTELLKEQISEAKGKLDIKRCACQHQLLVLFTNRVQNGIKYMILKTNFTMFMAIVCGAFQLINHEPFQVIVRFMCMVVAGSVRLYVSAKPAD
DLKENSENLVAAACWAASHQQSTSDSKMALMLAQRCSKTIVISVTSIIEAYNLQYYGSFLATSVKYFVNLRAVFEDK
>BdioOR141
MVAESVCFRYLMLTFSRKKLVKAVNMCAKLWSFVSATESIVVRSFERKAYFFRTFMLINSIGVVTSFIVTAYFIRVPGPTVNDTERRVLQFRFFVEVYEEPWFTLTAILQDV
VTYSISLEVAGIETVGLYLMMMACGYLRVIRNRLSKIQHNTLRADKTEKFNEDFDDILSCAILHQKIML
>BdioOR142
MDLFKGRYFNTNRILLTCIGFWPYAFKSKIPVRIIVMTVDMLLILPQIVSAIIHHDDIDRLMLNMVCFFCATFMFLKMIALCASESQVKILYKNIADNWSKIRDEREREILI
EYSERARKITIWYTAILMTLCSLITILLPMSPKILDTIDPLDEPRPAVYFMEGEYFVDRDEHFLPLFF
>BdioOR143
MLEMWFLLAIFLFTVTKCLNILVKRDLFYDYLSKSVLSERWAKFRDADEKSIVEQSKKNEVFIFKVWGGALFVNALFLIFNPIITENPEKKLLWESYSPCDRQVPKCFWI
WYIHQAYSYAIITVLNLAYDCLNYAVMERSCAHVKILQHRLKLLPELVKTGALPTVESELDYIILPRER
>BdioOR144
MSHCRKYRLRLKKALEIFEQCDGGNKIIDSDEEEAFNIISSAVALHIEAIQLARSVDQAYNVCWFIILLMNMMILAPIIVTMLFYLNNLFELCRYTVGLIMFLLHFYVIFE
QGQLINDHSSSVFTACYDSNWYITPKRIKMSIAMVMVNSLTPCQLTGGNMFVLSMETYGKQFIH
>BdioOR145
MSLLTAQITAYFFILYLFLKLDTRTCSFTKKLESSFSQSFYALQFIYTSNRLVIAYSGSIIICADIAVVLLIRMNAIRLKILDYNIKRKGHKNLKNCIREHQDIFKSIKLTNSIVSL
LVFKTVVFFILYCLCAGVLIFNVSNYWNAPLSIYFIKNIHFRSIWHLHPIYLFICVF

>BdioOR146
MKYSKNGILLTKGYIAYMLMATAFYITLPLTPLLLNVVVPLNETRRPDFIFKGEFPVDDMRDHYIEIFVFDAFGCLVTVYVLIAVDAMYACCVEHCTGLFAIIKYRLKSC
TSYTEVRDRDVAYELIVDIVKLHTKTLAFAELLEETYSKFFLFIIGMNMLHLTLVSVIVSSTV
>BdioOR147
MVLQRRSEFLAGLQNQLPAADASSDQRNYRTNDRRLRRAFFDYASVGYFRALRCKLYRAYDKANSEDEVLADRELNDCWRLHQSILNHCLDIDKITRTLFFMQLICT
GYNTSLLGIKLAGNDPDHWKYLPIIIVNITLMFICQWAPDHLIDESSSISTTAYRISFPWMKRRIG
>BdioOR148
MYQHLKKFWQISDDPQERRLYEQPANQAKLITIVIYVIAISNLLSFEIVAFISSATKHPEPNDSLRYRPLTVDVAYGFDTQRTPVYEIILISQNICGFCIVGSVVGFDSNMFV
YIVHVSSQFKVITARFIAIGRTVGEQSLAKDELHAQISKLIAQHQYTIRVVTELKSLLSP
>BdioOR149
MLEDWRVESQTSKVMRENCKRSQLVAKFIIILNNSMNSTYFLKCLASYIFDEVSDRKFLGRCKFPIDGRQSPFYEVINIGLFFLSSIHFNANALVDGFLAILVFHACSKIDI
VHREIKCYSKVCEMNHEGNEITLMALQKLYQKHLEFIKFSKNIQDVFSLAVFFFIYLC
>BdioOR150
MAKTEWSFDEVASSHLWFMRLLGLVPLAGKTLDYIGTKLLFCYFHVINVLYNGMWMGYGVRMVACKRFEVDFLCELIVVEGVCFRYLILATSRKKLVKIVNSCTELW
TYVTASESKVVRSFERKAYFFRTFMLVNGLAVVVAFSITAYFMRIPGATVNDTERRVLPFRSA
>BdioOR151
MHSIFSAWNMTIAMIGPLIPLFPKMLDAIKPLESPRPNKHISGAELFIDKETHFFLYFFLDYIAILLSLFVVTAIDSMYVVTVEHSCALYAIIRSRLLKLKPTVQESSPATEGV
DSYEWLCRTIQLHQKSLKLTSLLNDSYSLSFLFALVTALFIIPLNSCAIIANLNN
>BdioOR152
MDPTSEAVVVTSALTITLLPIIPKILNFMRPLENPRPSIYISASEIFIDRETHFLPYFILEYIEIMTIALVAITVDTMYIVSVEQCCALYAIVRFRVLELRSIPQKKSSGTEDVDSYE
WLYRTIQIHQDALIFSSLLNKSYSKLFLFLMILALFIIPFDSCAVCIFNIN
>BdioOR153
MCESNNENRKNTLLALRKLYQKHLEFIEFSKTIRDVFSLVAFFHVSFLTLLQVMAGFMFIDGLERGAKTVNLIHYAVLTTSFLITTGYYCICGEYLNYQSKTIFYELYNCY
WYTFSIDNKKAIKFILTKAQKPVSLTMGKFSDLSLIYFTTIVKTSFSYLSLVRAVR
>BdioOR154
MMSALVIFITMPALPSVLDKVSPLSNGSRPKIYILDGEFLVDKLENYRSIYFFEAFACTLTVFIFSSIDSTYAVTVEQCVGLFAVVKSRLKLATKHAKQASTVDYQTKDGPY
EKLVKAVELHKKVLQLSISQKKKLFVFFFHYFYYIFYFSYYVFSRNRFILNITK
>AmelOR1
MENTTNYRNIHYKSDAEYTVHVAKTLLTLIGIWPRRNTFIDNVKFYVQIGIVFFLMCFLLL

PHVIYTYFDCENLTKYMKVIAAQIFSLLAIIKFWTIIINREEIRFWLMEMEIQYRDVECEEDR
LVMMNTAKIGRFFTIVYLSLSYTGALPYHIILPLISERIVKEDNTTQIPLPYLSDYVFFVIEDS
PIYEMTFVLQIFISSIILSTNCGTYSLIASITMHCCGLFEVTNRKIKTLCKWNNRDLHDRVIDI
VQSHLKAIEYSARVGESLSIVFLSEMLGCTIIICFLEFGVIMELEDHKTLSTVTYFVLMTSIF
VNVFIISFIGDRLKQESERIRETSYFIPWYDFPTEVAKNIKTIILRASRPSSLSGAKILELSLQA
FCDVCKTSAAYFNFLRAMTV
>AmelOR2
MMKFKQQGLIADLMPNINLMKATGHFMFNYYTDSSTKHIHKIYCIVHLVLILMQFGFCGI
NLMMESEDVDDLTANTITMLFFTHSVVKLVYFAVRSKLFYRTLGIWNNPNSHPLFAESNAR
YHQIAVKKMRILLLAVIGTTVLSAISWTTITFIGDSVKKVIDPVTNETTYVEIPRLMVRSWY
PYDPSHGMAHILTLIFQFYWLIFCMADANLLDVLFCSWLLFACEQIQHLKNIMKPLMEFSA
TLDTVVPNSGELFKAGSAEQPKEQEPLPPVTPPQGENMLDMDLRGIYSNRTDFTTTFRPTA
GMTFNGGVGPNGLTKKQEMLVRSAIKYWVERHKHIVRLVTAIGDAYGVALLLHMLTTTIT
LTLLAYQATKIHAVDTYAASVVGYLLYSLGQVFMLCIFGNRLIEESSSVMEAAYSCHWYD
GSEEAKTFVQIVCQQCQKAMSISGAKFFTVSLDLFASVLGAMVTYFMVLVQLK
>AmelOR3
MSVKTARNIRDYHNIHYRSDAEYTVRVAKILLTMVGIWPRRNTFSNNVKFYVQTTIVFFL
MCFLLLPHVIYTYFDCENLTKYMKVIAAQVFSLLAIIKIWTILINRNEIRFCLMEMEVQYRD
VECEEDRLVMMNTAKIGRIFTIVYLFLGYGGALPYHVILPLISERIVKADNSTQIPLPYLSDY
VFFVIEDSPTYEITFVVQMFTSFLIMSLNYGIYSLIASITMHCCGLFEVTNRRIETILKNRDLR
GRIADIIQSHLKAIEYSALVGKSLSIVFLSEMLGCTIIICFLEFGVIVEWEDHKTFSMVTYFVL
VTSMFVNVFILSFIGDRLKQESERIGQTSYFLPWYEFPTEIAKNIRIIILRASRPSSLSGAKML
DLSLRVFCDVFKTSAAYLNFLRTMTV
>AmelOR4
METKHTEKDLKQAFYVQTFLKIIGAWPIAIESSLGSKIQKWFIISFYLFLQICIVAPCILDVFL
KEKNGSRRINLFMLLISTLNQVFKYVITLNRANELRIAIHEIKKDWLTATPEDRFIFVMNSRI
GQRIMLIMAFIMYISGLGYRMVLPLLKGKIVLPNNVTIRLLPCPTYFTFFNELVSPYYEMIF
MLQLLARFFIYTVLNSTVGISLMLSLHMCSLLKILTRKMADLTDGSIISEKIMQQRIVDIIEY
QTRIKRFLSNTELITQYFCFYDIGCSTCLICFIGYSIIVEWENHNIASTVIYFSGLVTCTLMIYI

ICYIGQLLLDESNNLAQTCITLNWYRFPKKKARYLILMIIMSNYPIKLTAAKVVDVSLTTFT
DVMKAAVGYLNMLREVI
>AmelOR5
METKHTEKDLKQAFYAQSFLKIVGVWPIPIGSPLSSKIRNWFITFFSLFLQICIVGPCILVMFL
KEKNGKRKINLFKLLTNTLNQLFKYIITLNRANELAIAMNEIKNDWLTATSEDRWIFTANSK
MGQKVMLIVAVTVYSSGLGYRMLLPILKGKIVLPNNVTIRLLPCPTYFTFFNELVSPYYEMI
FMLQLLAGFFSYTVLNGTVGISLMLSLHMCSLLKILTRKMANLTDRSITSENIIQEKIVEIVE
YQTKIKRFLGNAELITEYFCFYDIGCNMCLMCFIGYSAILEWENHNIAAIVVHFMLLGTCIF
IIYIVCYIGQLLLDESNNLAQQCITLSWYHFPTRKARCLILMIIMSNYPVKLTAAKVVDVSL
TTFTDVMKAAMGYLNMLREVI
>AmelOR6
METKEKDLKQAFYAQPFLKIIGAWPILIESSLSSKIQKWFIISFSISLQMCIVVPCILVMFLKEKNGRRKINLFMLLTNILNQVFKYVITLNRANELRIAIHEIKKDWLTATPE
DRFIFVTNSRIGQRIMLIIAVITYSSGLGYRMVLPLLKGKIVLANNVTIRLLPCPTYFTFFNELVSPYYEMIFMLQ
ILAGVFVYTVLSGTIGISLMLSLHMCSLLKILRRKMIDLADGSITSENTMQKRIVDIVEYQT
KIKRFLGNTELITQYFCFYEISCNTCLICFIGYCIILEWENSNVVAIVVHFMLLGTCILVTYIV
CYIGQLLIDESNNLARTCITLNWYHFPTRKARCLILIIIMSNYPVKLTAAKVVDVSLTTFTD
VMKAAMGYLNMLREVI
>AmelOR7
METKEKDLKQAFYAQPFLKIIGAWPIVIESSLSSKIRKWFIISFSISLQMCIVVPCILVMFLKE
KNGRRKINLFMLLTNILNQVFKYVITLNRANELRIAIHEIKKDWLTATPEDRFIFVTNSRIGQ
RIMLIIAVIMYSSGLGYRMVLPLLKGKIVLPNNVTIRLLPCPTYFTFFNELVSPYYEIIFMLQI
LAGFFIYTVLSGTIGISLMLSLHMCSLLKILRRKMIDLADGSITSENTMQKRIVDIVEYQTKI
KRFLGNTELITQYFCFYEISCNTCLICFIGYCIILEWENRNVIAIVVHFMLLGTCIFVTYIVCY
IGQLLLDESNNLARTCITLNWYHFPTRKARCLILIIIMSNYPVKLTAAKVVDVSLTTFTDVM
KAAMGYLNMLREVT
>AmelOR8
MVQIRNAREGINHTFWFAYPLSRMLGYWPLNVPSSAFSKILNSFTIFFSYLLPLIVLIPGLLY
VFLKERNGRRKVKMLMPHINSIAQMTKYTIILRRTKELGKLLDEIKKDWSTATQENRRIFS
ERASIEHKLTMIVAITIYGGGFLYRAILPLSKGRIVLPNNVTIRLLPCPGYFGSLDEQVTPNY

EIIFTLQVLGGFVTHTAVCGIKSACLMVCMHMCGLLRILTNKLTDLTNDNDERVVQEKIVH
IVEYQTRIKEFLNHVDQFVPYVYLIEIFVGVLITCILGYCIIVEWEDSDAMAIIAYVALQTTC
VFGTFSICYVGQLLVDESESVRQACKTLKWYRLPTKKARSLILLIIMSNYPIKVTAGRLVD
VSLVTFTSIIKSAVGYMNILQQVT
>AmelOR9
MARIRNAREGINHTLWFAYPLSKMVGCWPLNIPSSTFSKIFNAFIIFISYLLSLIVLVPGLLYL
FLKEKNGRRKIKMLMPLMSTIAQMTKYTILLRRMKEFNKLLDEIKKDWSTATQENRQIFS
AKASIEHKLTTVIAITIYGGGIFYRMILPLSKGRIVLPNNVTIRLLPCPGYFGSLNVQITPNYE
IIFTLQILGGFVIYTALCGVKSSCLMLCMHMCGLLRILTNKVMELTSDKDEKVVQEKIVYI
VQYQTRIKEFYNYVDQFVPYVYFIEMIVGVLITCVLGYCIIVEWEDSDAMAIIAYVVLQVT
CVFGTFSICYAGQLLVDESENVRQACNTLKWYRLPTKKARSLILLIIMSNYPLKVTAGRIV
DVSLVTFTSIIKSAVGYMNILQQIT
>AmelOR10
MVQIRNAKEGLRHTFWFAYPFSRMLGHWPLSVSSSAFSKILNSFIIFISYLLQMIVVIPSLLY
VILKEKNPKKKIKLLMPHLNSIVQMIKYTILLRQMKLIDKLLDEIKKDWSIATEENRRIFSRT
ASVEHKLTSIIAITIYSGGFFYRMILPFSKNKIVSNNMTIRLLPCPGYFGSLDEQVSPNYEIIFI
LQVFGGFVIYTAVCSTKSICLMLCMHMCGLLRILTNKVMELTNDNDERVVQEKIVHIVEY
QMKIKEFLKQIDQFVPTIYLFEVFIQVLIMCIIGYCIIMEWKESNGMGLITYVIVQMTCLIGS
FSVCYVGQLLIDESENIRQAFIALKWYQLPVKKSRSLILLIIISNYPIKVTAGKIIDLSLVTFITI
IKTAVSYMNMLQQIT
>AmelOR11
MVQIRNAKEGLKHTFWFAYPFSRTLGYWPLVSPSAFTKFFNSFTIFTLYFLELIVLIPGLLYV
LQVKNPRTKIKLLMPHLNSIAQMAKYTIILQRAKEFSKLLDEIKKDWLLATEENRQIFSERA
SIEHKLTTVIVVTMYGGGFFYRTILPLSKGKILLPNNMTVRLLPCPSYFGSLNEQATPNYEII
FTLQVLGGFIIYTVLCGTKSACLMLCLHMCGLLKILTNKVMDLTNDSDEQVVQEKIVHIVE
YQTRIKEFLNQLDQFVPAIYLIEVVIQVLIICIIGYCIIMEWEDSNAMAMVIYVVFQVTCVIG
TFSVCYVGQLLLDESENIRQAYNTLNWYRLPVKKARSLILLILMSHYPIKVTAGRIMDLSL
VTFTSIIKSAVGYMNMLRTVT
>AmelOR12

MARIRNAKDGIRHTFWFAYPFSRMLGYWPLSVSSSAFAKISNYFIIFLSYLLTLIFMVPGLLY
IFLKVKNGRSRIKLLMSHINGIVQMAKYTILLRKTKEIAKLLDEIKKDWMTASEENRQIFST
RASIEHKLTMVVVVTMYGGGFFYRAILPLSKGKIVLSNNVTIRLLPCPGYFGFLDEQVSPN
YEIIFTLQVLGGFVIYTAVCGTKSICLMLCLHMCGLLKILTNKVMELTNDKDEKVVQEKIA
HIVDYQTRIIEFLNDLNQFVPSVYFFEIILEVLIICIIGYCLITEWEDNNTMATVIFVIFQITCFI
GTFAVCYAGQLLVDESENVRQACSTLNWYRLPVKKARSLILLILMSNYPIKVTAGRIVDVS
LVTFTSIIKNSVGYMNILQQVT
>AmelOR13
MGQPYSLKLVYPLLKILGAWPKSSPSSVLSTILKCCLISICYLIQLMVLIPGILYIFLKEANLG
GKIKMFVPHMNGITQVSKYTILLRQIKEFNIILKEVKRDYSLATDKNMWIFTTRAYIGHKM
MIAIAIAMYSSGVGYRMILPFLKGRILLPDNTTVRLLPCPGYYMFLNEQVTPNYEIIFTIQV
LGGFLNYTTLCGTTGITTMLCLHMCSLLEILINKMNDLTCQSDECEIIVRKKLADIVEYQM
KIIDFLNHVEQLTSYLYFCEILEYVCGACVIGYCLITEWENSNAAALIVYFILEFLCIFCTLTI
CYIGQLLIDESDKVRQISVTLDWYRLPVNEARGLILVIIMSNYPIKVTAGKIVDISLITFTDIV
KTSVGYLNILRTVA
>AmelOR14
MSRVGKAENGMRHTVWFAYPLLRILGAWPNRVSSSTLSKIFNWYLIFTCYTLQLIVLVPGF
LHVFLKEKNGRKKMKMMIPQVNGYLQLCKYSLVLRWTNKLRVLLNEMKEDWLNTTEE
DQLIFRAKASFGHRVMSMIAIVTYSAGLGYRTILPLSKGRILLPNNTTKRLLPCPGYFVFFN
EQVSPIYEIIFIIQVLGGLLTYTIMCGTIGMCVMFCLHSSSLLRILLNKIYQLTKQLDVNEVV
VHEKIVDIVKYQTKVKGFLKNVEQLTTYLFLLEIMVETSIGCVIGYNVVTEWEDSNAAAM
IIHLMMQVSTISCTFIMCYVGQTLIDEGNNVRRMSITLDWYRFPVKEARNLILVIIMSSYPV
KLTAGKVVDISLATFTDIIKTTVGYLNMLQKVT
>AmelOR15
MSRIGNAEDGMRHTIWFAYMLLGKLGAWPNRATSSTSFSRTRNCILIFMCYSVQLIILIPGL
LHFFLKEKDSRKKVKILIPLINGYLQLCRYSLVLRSANKLCHLLNEMKKDWMNISEEDRLI
FRRKASIGHRLMSVVAIIMYSAGLGYRTFIPLSKGRILLPDNTTIRLLPCPGYYIFFNEQITPN
YEIVFTLQVIGGLLSYTIMCGTTSMCAMLCLHATSLLRILVKKINELTKQPDINESAVHMKI
TDIVRYQTKIKQFLNDVEHITTYLFLLEIIDETGIGCVIGYCAITEWEDSDATAAIIYLLLEAS

VFGVTFTMCYVGQILIDEGNNVRRMSITIDWYRFPAKEARNLILVIIMSSYPVKLTAGKVV
DISLSTYTDIIKATVGYLNMLRKVT
>AmelOR16
MENISGIAKAEEDLKYATRFVKPILATIGAWPISSSTSFFLKALQRLGHIFTYFLFFLIMIPTLA
YVFLKEKNSKVRLKLMGPIINCSMQFFKYTIIIWRRKEIQEGLHAIRHDWIQATEEERLIFRS
KMKIGRRVVLIAAFTMYGGGLCYRTILPLLKGTVITADNITIRPLPCPSYFIIINEQQSPIYEIL
FVLQVMAGMAIYAVISGTCGISALLVLHACSMLRILVNKIKKLVNKSDMSEVTLQRKIMDI
VEYQMKIKRFLKNIETVTEYICLIEMIGGTCLMCLVGYCILMELENTNTMAVVVYITLQISII
FCVFILCYIGQMLVDENYIVSQASSTINWYRLSIKNMRCLILIIAMSNYPMKLKAAKMME
MSLTTFTDVMKMSMGYLNILREVI
>AmelOR17
MENISGIAKAEEDLKYATRFVKPIMGMIGAWPISPSTSFLLKVLQRLRHIFTYFLFFLIMIPTL
MYVFLKEKNNKVRLKLMPPIINCSIQCFKYTIILWRRKEIQEGLYAIKHDWIKATEEERLIFR
SKAKIGRRVVLVVAFTMYGGGLCYRMILPLLKGTIVTANNTMIRALPCPSYFFILNEQQSPI
YEILFVLQIIAGIAIYAVICGFCGIFALLVLHAWSMLRILVNKIKKLVDKSDMSEVVLQRKIM
DIVEYQMKIKRFLKNIETITEYICLIEMIGSTCMICLVGYCILMEWENTNTMAIVIYITIQISII
FCIFILCYIGQLLVDENYIVSQASSTINWYRLSIKNMRCLILIIAMSNYPMKLKAAKMMEMS
LITFTDIMKVSMGYLNILREII
>AmelOR18
MNAEKLMIEGKPPNANYKNDLSFNVRLNVWTLRTIGTWPRSPDHSWLETLEHVCLNLFC
YELLAFILIPCSIYIILEIKDFYNQLKLGSALSFFLMAVMKYCVFIIREDDIRKCVELIENDWK
NVRYQEDRKIMLENASFSRRLIVICGTFMYGGVIFYYIALPLTRAKIVEEGGNLTYRRLVYP
FPKVLLDARHSPINEICYTIQLLSGFVAHNITVAACGLAALLAIHACGQLQILMSWLEKLVD
GRKNDNENLDQRLANIVKQHVRIINFIALTEDLLHEISLIEVVGCTLNICFLGYYSMMEWD
SKQPVSGVTYIILLISVTFNIFIFCYIGQLLAEQTVKVGEKSYMIDWHRMPWKKSLAIPLMI
SMSHSTTKITAGNIIELSISSFGDVIKTSVAYLNMLRTFTT
>AmelOR19
MNMEHFIVEKKSYNASYKNDLFFNVQLNVWTLRTIGTWPKSLDRSWLETIEHVCLCFLN
YVLLAFILIPGVMYFLLEMKDFYDQMKLGSALSFFLMAVMKMCVFIIRENDIRKCIECIED

DWKNVKYQEDRKIMLENASFSRRLIVICGAFMYGGVVFYYIALPFTRAKVVEEGGNLTY
RRLVYPFPKALLDARRTPANELLYTIQLLSGFVAHNITVAACGLAALLAMHACGQLQILMS
WLEKLVDGRENDDENLDQRLVNIVEQHVRIINFITLTEDLLREISLVEVVGCTINICFLGYYS
MMEWDTEHLIRGMTYIILLTSVTFNIFIFCYIGELLAEQTVKVGEKFYMIDWYRMPWKKS
LAISLIISISRSTTKITAGNIIELSISSFGAIIKTSFAYLNILRTLTS
>AmelOR20
MEKSKDKANQKFYLTDYEYQKNVNLSIQYNRWLLKPMGLWPNSYTSKDYPYWLINIVC
YCLISFLFIPCTLYLFLEIEDFYGKLKQFGPLIFCMMAFVKYYYLIFHKTDIRECVERIKWD
WRNITYAKDREIMIMYANFGRKLVMVCTFFMYSGFAFYYIAIPISVGRVKTDNLTFVPLVFP
FSRFIVDTRYSPTNEIVFSIQLMAGALMHGITSAACSLVATFAVHACGQMQVLMNWLQHLI
DGRLDMDERLDGRIADVIRQHVRVLKFLALTEKTLQQISFTEFLGCTLDICLVGYYVIMES
KSNDVTSVITYIILLISLTFNIFIFCYIGEIVAEECRKIGEISYMIEWYRLMGNKKLFCILIIAMS
NSSIKLTAGNIVNLSISTFTDVVKTAVTYLNVLQKTT
>AmelOR21
MSSVKIDQDYKSNVNLSIKYSRRISKMIGLWPIFDKISTIHKFLRMLYNTICYCLLMFMIVL
GWMYIAFEVKNIYDGLKFVSLMSFCMLSITKYHLINIHKDDVRECVKRIEWDWKNISYSE
DREIMLMNANFGKRLIIVTTTVTYSGFVFFYIAIPMKIGKIPAPDANISFIPTMFPFPKYIADV
RYSPINEIVFFFQFMCGFLVHGVTSSACSLAAIFTVHACGQIQVMMIWLEHLIEGRLDMCY
SVDQRIAKIVSQHVRILKFLSLIEKILQQVSYMEFLECTVNVCLLGYCAIIEWESNHLTEVV
TYVIILITIIFNIFVFCYIGELLADQSRKIGEVTYMIEWYRLSGKKKLCCVLIIAMSNSSMKLT
AGNLIELSMSTFSDVVKTSFAFLNVLRTLT
>AmelOR22
MEKSKINSISCIQTNHDYKRNVNLSIQWSRWILKPIGLWPNSSTISTTGKYLYRLINVICYSL
ISFLSIPCSLYVILEVEDIYNRIKLFGPLSFCVMAFLKYHLLILHKDNISECIKRIEWDWKNIT
YSKDIEIMITNANFGRRLVIICTFFMYSGFAFYYIAVPISVGKILAEDDNITFIPLVFPFSRFIID
TRYSFINEIVFSIQLIAGALMHTITTAACSLAAIFAVHACGQMQVLSNWLKHLINGRSDMY
NNVDSRIASIVSQHVRILKFLALTEKALQQVSFVEFLGCMLNICLLGYYVITEWSSSHLTSA
ITFFILLISLTFNIFIFCYIGELVAEQCKKIGEISYMVDWYRLEGNKKLCFVLIIAMSNSSIKLT
AGNMVELCLTTFSDIVKTAVAFLNVLRTLTI

>AmelOR23
MSSVKINQDIKNNINFSIKYSRLILKMIGLWPIFDKSSTIHKYLQWLYNVICYSLIMFIIISGW
IYISLEVENIYDRLKFVSLMSFCMLSITKYHLINIHKDDVRECVKRIEWDWKNISYSEDREI
MLMNANFGKRLIIVTTTVTYSGFVFFYIAVPMKIGKIPAPDANISFIPTMFPFPKYIADVRYS
PINEIVFLAQFICGFLLHGITSSVCSLAAILTVHACGQIQVMMVWLKHLIDGRLDMCNSIDQ
RIATIVNQHVRILKFLSLIEKILQQVSYMEFLECTMNVCLLGYCAIMEWESNHLTEVITYLI
LLITIIFNIFIFCYIGELLANQSRNIGEVTYMIEWYQLFGKKKLCCVLIIAMSNSSTKLTAGNL
IELSMSTFSDVIKTSFAFLNVLRTLT
>AmelOR24
MSYTKTDHDYKRNVNLSIQWSRWILKPIGLWPNSSTISTTGKYLYRLINVICYSLISFLSIPC
SLYVILEVEDIYNRIKLFGPLSFCVMAFLKYHLLILHKDNISECIKRIEWDWKNITYSKDREI
MITNANFGRRLVVICTFFMYSGFAFYYIAVPISVGKIPAEDDNITFIPLVFPFSRFIIDTRYSFT
NEIVFCIQLVAGVLLHTITTAACSLAAIFAVHACGQMQVLSSWLKHLINGRSDMYNNVDSR
IASIVNQHVRILKFLALTEKALQQVSFVEFLGCMLDICLLGYYVIMEWSSSHLTSAITFFILL
ISLTFNIFIFCYIGELVAEQCKKVGEISYMVDWYRLEGNKKLCFVLIIAMSNSSIKLTAGNM
VELCLTTFSDIVKTAVAFLNVLRTLT
>AmelOR25
MEKQQYVIAQDDGKKANLSIQWNRWLLTPIGAWPNLRKSRIGKCYSLLISIICYGLIGFML
VSCSMFLMVEIKKVYNRIKMIGPLSFFLMTFMKYYLLLLHENDIREGIECIEWDWKNMKH
QEDRNIMIEYANYGRKLVLICTFFMYSAFAFYYLVLPFSVGKIEDGNLTFIQLPFPSSSLIADI
RYSPYNEIVLSVQILTGVVMHAITSAACSIAAVFAVHACGQMQVLMNWLDHLVDGRSDM
SKAIDDRIANIVIQHDRILKFLALTEKALQQISFVEFLGCTANMCLLGYYLIVEWNPKEIILS
VTYVALIISITFNIFIFCYIGDGVAEQCQKVGEMAYMIEWYRLTGKKKLCCILIIAMSNSSVK
FTAGNMVELSIYTFSDVVKTSVAFLNMFRALT
>AmelOR26
MMNQLNEQSVLMPVSYARDYEYSIQVNRWLLKPIGAWPNLTKATRTEKLLVKLLNFICHS
LIIFTVMPCIMYIFYEDESLKTRMKAIGPTSHWLMGELNYCCLLMRAKEIVYCIEHIKYDW
KTVRRARDRELMIKNAKLGRFIACIAALCMHSGIMSYTVITGFKKITFQIGNDSYSMYRLP
CPFYTNLLDVRFSPMNEIVFALQLLSGFISTSVTVGACGLAAVLAMHACGQFNVVMIRSD

KLVKDNNEKKQDEQTLHKKLGFIVEHHLRTLSLVWYMEKVMNMICLVELVGCTMNMCI
LKYYFLTEKSKTILGIYAIVYASMVFNIFIFCYIAEIVTEQGKKVGEKFYMTEWYQLPHKTA
LGLVLIISRSSMVIKITAGKLIQISIATFAAVFKASFAYLNMIRTIAM
>AmelOR27
MMNQTAITEEIKTNSDYSLQLNRWFLKPIGAWPLFSTTTKFEKTVSLILNIICYAIVILCATPS
LMQIILAEESFYLKLKTLGPVSHWFVSTVNYTALLMKSKDIRYCFEHMEADWQTIKRMED
QQTMLKNAKFGRYVAASCAIFMQGGILCFCFVTILTTETIQVGNETRVLHVLPCAVYKKLV
NVEENSINIFMLCFQFVAAAIANSSTVGIFSLAAVLAAHAYGQLSVVMVWITEFVNQSRNQ
KKTDDFKEIGIIVERHLRVLNFITYLENIMNRIYFLELFRCTMIICIVGYYILTEWAEKNVQN
LTTYFMMLLSICFNIFIICYIGEILTEQCMKIGEVVYMTDWYYLPDKTILNLILIILRSTVVVQ
ITAGKLFNMSIYTFGDVLKTAFAYLNLLRQMT
>AmelOR28P
MSNRSVAIKTDPDTNSDYCLQLNRWFLKPIGAWPSFPSTTKHERIISFLLNVSCYSSLLFTLI
PCLLHMLLEDESFYLKMKVLGSLAHWFVGTMNYTTLLLRGKEIRLCVEHIRTDWQTVTR
EEDQQVMLKNAKFGRYVAAVSAAILQSGVNCXCCMTISRTELIQIGNETRIVHVLPCAVYR
KLIDVTHSPNSELIIASQFLSGFIVNSSTAGIFSLAAILGAHACGQLSVVMTWITEFVNKSKK
REKMIFREIGLIVEHHLRTLNFISCIEETINRIIFLEVFRCCLHICCLGYYILMEWSDYDKRSM
IIYFMLFVSVCFNIFIICYIGEILAEESMKVGEVVYMTDWYYLPDKTILDLTLIIARSSVVVQI
TAGKLIHMSIQTFTDVIKTGFAYLNLLRQVT
>AmelOR29
MKNQQVVITQDDYKRKTNLSIQWNRWLLTPIGAWPNLRKSRIGKCYSLLISIICYSLIGFML
VSCSIFLMVEINNIYNKLKMVGPLSFFVMTIMKYYFLLFHENDIREGIERIEWDWKNVKH
QEDRNIMITYANYGRKLAFICFFFMLCAFIFYFLIQPFGGGKIVDGNLTFIQLPFPISILIADV
RDSPYNEIMLSIQILTGIVMNAIRSAICSVAAVFAIHACGQMQVLMNWLNHLVEGRSDMSK
KIDDRIANIVIQHDRILKFLALTERALQQISFVEFLGCTANMCLLGYYLIVEWNPKELIVSFT
YIAIIASITFNIFIFCYIGELVAEQTEKVGEVAYMIEWYRIRGKKKLCCVLIIAMSNSSIKFTAG
NMVELSIYTFSDVVKTSVAFLNMLRALT
>AmelOR30
MEKNRSIIGHDDYERNVNLSIRWNRFLLKSLGTWPNLRESRIGKCYSVLIGIVCYGLISFML

TSSNMFLVVEVKDTYNRIKMIGPLSFFAMTLIKYYFLTFHEENIRKGIEHIEWDWKNVKHE
EDKRIMIEYANYGKKLALISIFFVYSAFVFYYFVVPISVGKIRDENLTFIPLPFPSSKLIADMR
QSPANEILFSVQVLSGVIIHAITATAVSIAAVFAVHACGQMQMLMNWLECLVDGRSDMNKI
VDKRIAKIVVQHDRILKFLALTERALQQISFVEFLGCTMNMCLLGYYLIVEWNPKEISLSLT
YISLLISFTFNIFIFCYIGDLVAEQCQKVGEMTYMIEWYRLTGKKKLCCVLIIAMSNSSIKFT
AGNMVELSIYTFSDVVKTSVAFLNMLRALT
>AmelOR31
MTSKSVISEESFDSLCDYSLQLNRWLLKPIGAWPSSSSSSKLERIVSFFLIVLCYGFILFTVIP
SLFHIVLEDENLHMKLKVFGPLSHWFIGGINYTTLLLQNKEIQYCVEHMQTDWKIVNRAK
DQQVMMKYAKIGRYIAALCAIFMQTGVLTYCVVTAFSTRIIEIGNETRIVHMLPCPVYKELI
SIDTSPTNEIVLISQFVSGFIVNSIAVGAISIGAVFTAHACGQLTIIKRWIREYINRSKDNNKNV
VINEIGEIVEYHLRILNFIEGIEDVLNRFCFMELFKSTLDISMLGYYILTEWADHDIRNLTTYF
MILTSMSFNIFIICYIGDILMEQCRKVGEVLYMTNWYYLPYKDILDLILIISRSNAVIKITAGK
LTNMSIYTFGNVMKTTFTYFNLLRHVT
>AmelOR32
MIDKFASIQQTNNNLSNYSIQLNRWFLKPIGAWPPSPSTTKLEKIISIVLIICCYSSICFTVIPC
LLHVMLEDESFRDKLKVLGPLSHWFIGAINYTTLLLRSKEIRYCIEHMQRDWRIVTRTEDQ
QIMMKHAKIGRYIAVFSAAFMQGGVLSNCAVTAFSTQTIEIGNVTKTIHMIPCTAYKKLIAV
DTSPTNEIVIASQFLSGFIVNSSAVGAVSIAAVFAAHACGQLSLLMVWIREFVDHSKKIHDK
NIGLNKIGKIVRHHLRTLSFVTGIENVMSGICFMELFKCTVNICMLGYYILTAWSVHDIQN
MVVFLVILLSMIFNIFIICYIGDILTEQCKMIGEAVYMTNWYYLPGKDILNLVQIILRSSMVIK
ITAGKLVHMSIYTFGNVMKTAFAYLNLLRQMT
>AmelOR33
MMTSKSVPIEQDNHSLSNYSVQLNRWFLKSIGTWPLSPSTTKLEKTISFLLIICCYCFICFTV
IPCLLHIILGDDSFREKLKVLGPLSHWFIGGINYTTLLLRKKEIRYCIKHVQRDWRIVTRME
DQQVMIKHAKIGRYISMMCAAFMQGGVLSYCAVTAFSTQTIEIGNETRIVHMIPCIVYKKL
IATDTSPTNEIVIASQFVSGFIVNSSAVGAVSIAAVFAAHACGQINLLMAWIRQLVNHSNVN
NKNVGLDKISNIVRHHLRILSFITGIENVMSGICFMELFKCTMNICMLGYYVLTAWIDNDM
RNLIVCSVILFSMIFNIFIICYIGDILTEQCKMIGEAVYMTNWYYLPGKDILDLIQIILRSSMVI

KITAGKLVHMSIYTFGNVMKTAFTYLNLLRQLT
>AmelOR34
MMIDKFVPIEQDNHSLSNHSVQLNRWLLKSIGAWPSFSSTTKLEKIISFVLIICCYCFICFTVI
PCLLHVILEDDSFHEKLKVLGPLSHWLVGGINYTTLLLRNKEIRYCIEHMQRDWEIVTKTE
DQQVMIKHAKIGRYITMFCAAFMQGGVLSYCAVTAFSTQTIEIGNETRIVHMIPCVVYKKL
IASDTSPTNEIVIASQFVSGFIVNSSAVGAVSIAAVFTAHACGQVSLLMAWIRQFVDHSNIQD
KNIVLNDIGEIIRHHLKILSFITGIENVMSGICFMELFKCTVNICMLGYYILTAWTGHDIQSLI
VFSVILFSMIFNIFIICYIGDVLTEQCKMIGEAVYMTNWYYLPGKDILNLIQIILRSSMVIKIT
AGKLVHMSIYTFGNVMKTAFTYLNLLRQMT
>AmelOR35
MLVLKDSSSVSYSKDWIYSVQINRWLLKAIGIWPLSLCVTTTEKIHSVILTLISIFLIGFLLVP
CTLCTLLDKTGDLDTKIKMIGPFSFCIMAAIKYYVLLSRGSHIGKCIEDIRVDWFRVSSHNC
LEDRKIMMENARIGRSLAIFCAGFMYSGGFFYTTVMPLCTKRTEIIDNEIVRSQAFPIYRGL
LDPRTSPSFEIVQLMQCLAGFVIYSVTVGSCSLAAVFVMHACGQFQILVTKLRRLIDGLKE
DKDMENIVHEQRLGNIVEHHLHILGFISQIEELLNEICFVEFIGCTLNICFLGYFLLKEWEQS
ETIGILTYCILLISFIFNIFILCYIGEILSEECKSIGLSAYMIDWHRLPGKKALSLILISAASNSST
KLTAGKLVELSLSSFCSVLKSSLAYLSLLRTLTT
>AmelOR36
MTDDISAIQKKFGSLNEYSIQVNRWLSKTIGVWPLPSSTSKFEKITTRILILFCWTIAVLDTT
SGLLHFVLVKEDIIIKLKSLAPISYILGGGLNYAVLLLRKNDIRYCIDRIEADWKVITRMADR
QVMLKNAKIGRIISCCIVGFMQLGTFCFCTILGVFKRTIKIGNDSMEIYVLPSPTYKIPVDTN
PGHDIVLGFQYVAAYITSATVISAFSFATVFACHASGQLTIMIIWIEEFINRSQKENKNRIDEIS
VIIEHHMRILSFLERAEHLLSPICFMEMFKNILSICLFSYCILAEWSEHNIRILSTYILAVINITL
NTFLICYIGEVLTERCKEIGNMVYMTNWYRLPKKDILNLIMIITRSSVEYKMTAGKIIDMSV
ITFGNIIKTVFGYLNILRQVTML
>AmelOR37
MMADDIATVQKEFENLNEYSIQFNKWFSKTIGVWPLPSSTSKFEKIMTRILILFCWIIALFD
AISGLLHFVLVKEDIIIKLKSLAPISYIFGGGLNYAVLLLRKDDILYCIEHMETDWKTITRMT
DRQIMLKNAKIGRIISCCILAFMQVSAVCFCTVLGVFKRTIKIGNESMEIYVLPSPIYKIPVD

TNPGHDIVLGFQYLAAYITSATVVSAFSFATVFACHASGQLTIMIIWIKEFINRPQKENKNRI
DEISVIIEHHMRILSFLERAEHLLSPICFMEMFKNILSICLFSYCILAEWSEHNIRILGTYIFAVI
NITLNTFLICYIGEVLTERCKKIGNMVYMTNWYRLPKKDILNLIMIITRSSVEYKITAGKIID
MSVITFGNIIKTVFGYLNILRQTTML
>AmelOR38
MMADDIATVQKEFNNLNEYSIQFNKWFSKTIGVWPLPSSTSKFEKIVTRILIIVCSIITLFVII
PSMLHFILVKEDIISKLKSLGPISYCFGGGLNYAVLLLRKNDIRYCIDHIETDWKVITRMTDR
QVMLKNAKIGRIISCCIVGFLQIGTFCFCTILGVFKRTIKIGNNSMEIYVLPSPAYKIPVDTNP
GHDIVLCFQYLAAYITSATVVSAFSFAIVFACHASGQLTIMIIWIEEFINRPQEENKNVHIDKI
SVIIKHHMRILSFLERAEHLLSPICFMEMFKNILSICLFSYCILAEWSEHNIRILGTYIITVINIT
LNTFLICYIGEVLTERCKEIGDMVYMTNWYRLPKKDILNLIMIITRSSVEYKMTAGKIIDMS
VITFGNIIKTVFGYLNILRQTTML
>AmelOR39
MMADDIATVQKEFDNLNEYSIQFNKWFAKTIGVWPLPSSTSKLEKIMTRILILFCWITTLFV
TISSLLHFTLVKEDIIIKLKSLAPISYCFGGGLNYAVLLYRKSDILYCIEHMEVDWKAITKTA
DRQIMFKNAKIGRIISCCIAAFVQISAVCFCTVLGVFKRTIKIGNESMEIHVLPSPTYKIPVDT
NPGYGIILGLQFLTGYIMSATVVIAFSFATVFACHTIGQLTIMVTWIEEFINRPQEENKNVHI
DKISVIIKHHMRILSFLERAEHLLSPICFMEMFKNILSICMFSYCILAEWSEHDIRILTTYTFA
VMNLIFSTFLICYIGEILTERCKEIGNMVYMTNWYQLHDKDILNLIMIIVRSSVEYKMTAGK
IMDMSVITFGNIIKTVFGYLNILRQTTML
>AmelOR40
MADDITAIQKKFGSLNEYSIQLNRWLSKTIGVWPLPSSTTKFEKIMTKILIFLCWIIALFVITS
SLLHFTLVKEDIISKLKTLGPISYCFGGGLNYAVLLLRKDDIRYCIDHIETDWKAITRTGDRQ
VMFKNAKIGRIISGCIASFMQVSTICFGIVFGVFKQKIKIGNESMEIHVLPFPTYKIPVDTNL
EHSIVLGFQFLTGCIMSATVVIAFSLATVFACHAAGQLTIMVTWIKEFVNRPQEENKNMRV
NEISVIIEHHLRILSFLGRTEHLLSPICFMEMFKNVLSICMLSYCILVEWSGRDIRALSAYTFS
VMNIALSTFLICYIGEVLTEKCKEIGNMVYMTNWYRLSDKDILNLIMIITRSSVEYKMTAG
KIIDMSVITFGNIIKTIFAYLNILRQMTIL
>AmelOR41

MADDIVAIQKKFGSLNEYSIQVNRWLSKTIGVWPFTSTTSKFEKIMTKILIIVCSIIALFVTVP
SMLHFILVKEDIITKLKMTGPIIYCIGGGLNYAILLFLRDDIRYCIEHIEADWKTITRTGDRQV
MFKNAKIGRIISGCIGSFLQFSTISYCTVFGVFKQTIKIGNESMEIHVLPFPTYKIPVDTNLEH
GIVLGFQYLTACIMTATIIIAFSLATVFACHAVGQLTIMVTWIEEFVNRPQEEKKNMRINEIS
VIIEHHLRILSFLERTEHLLNPIYFMEMFKNILTTCMLSYCILVEWSGHDIKVLSAYSFTITNII
LSLFLICYISEVLNEKCKEIGNIVYMTNWYRLSDKDILNLIMIIIRSSVEYKMTAGKIIDMSVI
TFSNIIKTIFAYLNILRQVTIL
>AmelOR42
MADDIVAIQKKFGSLNEYSIQVNRWLSKTIGVWPFTSTTSKFEKIMTKILIIVCSIIALFVTIP
SMLHFILVKEDIITKLKMTGPIIYCIGGGLNYAILLFLRDDIRYCIEHIEADWKTITRTGDRQV
MFKNAKIGRIISGCIGSFLQFSTISYCTVFGVFKQTIKIGNESMEIHVLPFPTYKIPVDTNLEH
GIVLGFQYLTACIMTATIIIAFSLATVFACHAVGQLTIMVTWIEEFVNRPQEEKKNMRINEIS
VIIEHHLRILSFLERTEHLLNPIYFMEMFKNILTTCMLSYCILVEWSGHDIKVLSAYSFTITNII
LSLFLICYISEVLNEKCKEIGNIVYMTNWYRLSDKDILNLIMIIIRSSVEYKMTAGKIIDMSVI
TFSNIIKTIFAYLNILRQVTIL
>AmelOR43
MMADDIAAIQKKFGSLNEYSIQLNRWFSKTIGVWPLPSSTSKLEKIMTKILIFLCWIIALFVI
ISSLLYFALVKEDIISKLKTLGPISYCFGGGLNYAVLLLRKNDIRYCIDHIETDWKAITRTGDR
QVMFKNAKIGRIISGCVAGFLQLSTISFCTVFGVFKRRIKIGNESMEIYVLPFPTYKIPVDTN
PGHNIVLGFQFLAAYIMSATVVIAFSLATVFACHAIGQLTIMITWIEEFVNRPQEENKNMRV
NEISVIIEHHLRILSFLGRTEHLLSPICFMEMFKNILSICMLSYCILAEWYGRDVRVLGAYAF
SVTCITLNTFLICYIGEVLSEKCKKISNMIYMTNWYRLSEKDILNLIMIMIRSGMEYKMTAG
KIINMSVVTFGNIIKTILAYLNILRQMTIL
>AmelOR44
MADDIVAIQKKFGSLNEYSIQVNRWLSKTIGLWPLTSTTSKFEKIMTKILILLCWIIALFVTT
LSLLHFILVKEDIITKLKMIGPISYCVGGGLNYAVLLFLRDDIRYCIDHIETDWNAITRTQDR
QVMLKNAKIGRIISGCIAGFMQLDSICFCTVLGVFKQTIKVGNESIRVYILPYPTYKVPVDT
NPGHSILLLLQFLTTCIMSTTVVIAFSLATVFAYHAVGQLTIMVTWIEEFVNRPQEEKKNMR
IDEISVIIEHHLRILSFLGRIEHLLSPICFMEMFKNILSICMISYCILAEWSGRDVRALSTYAFC

VTCIILNTFLICYIGEILSEKCKKISDMIYMTNWYQLSDKDILNLIMIMIRSGVEYKMTAGKI
VNMSVITFGNIIKTIFTYLNILFQMTML
>AmelOR45
MADDIAAIQKKFGSLNEYSIQLNRWLSKTIGVWPLSSSSSKFEKIMTKILIFLCCIIALFVIIPS
LLHFTLVKEDIISKLKTLGPIGYCFGGGLNYAILLLRKNDIRYCIEHMKADWKAITRTDDQQ
IMLKNAKIGRIISCCFAAFMQFSTVIFCAVFGVFKRTIKISNESMEIYVLPFPTYKIPVDVNPG
HNIVLGFQFLAGYITTGTVIIAFSFATVFACHAVGQLTIMITWIEEFVNRPQEENKNVRVEEI
SVIIEHHLRILSFLERTEHLLSPICFMEMFKNILTICMLSYCILAEWSGHDIRALSAYASAVM
NISLGTFLICYVGEILTEKCKEIGNMVYMTNWYRLPKKDILNLIMIITRCSMEYKMSAGKM
IDMSVITFGNIVKTIFAYLNILRQMTIL
>AmelOR46
MSDDLVEVEKKFGSLNEYSIQFNRWILKPIGAWPISLCTTRNEKIISKILIIVCWSLSLFTLIP
GLLHFILEKEDTYLKLKTIGPLSHWVIGGFNYAVLLLRKNDILHCIEHIRVDWNIITKKQDQ
QVMLKYAKIGRYIAAFCTAFLQGGVLCTCIALGAFKTTIKNGNETIEIYSLLCPAYKLPVQT
NPTHDIILGTQLLSAFITSSSAAGAFSLAAVFASHALGQLNIMVAWINEFVNRPIDLNNVYV
NKISIIVEHHLRILSFITHIEHLMNPICFMEMFKCMVGMCMPSYYILAEWSEHNVQNLAVY
VMIIISMTCNIFLICYIGEILTEQCKKIGEIIYMTNWYELSNKDIFNLMMIISRSSISVNMSAG
KLIDMSVLTFGNTVKSVFVYLNMLRQMTMI
>AmelOR47
MADDIVAIQKKFGSLNEYSIQLNRWFSKTIGVWPLPSSSSKLEKIITKILIFLYWIIVLFIIITSL
LHFILVKEDIVSKLKSLGPISYCFGGGLNYAVLLLRKNDIRYCIDHIEADWKVITRMGDRQV
MLKNAKIGRIISCCIVCFMQIGTLCFCTILGVFKRTIKIGNDSMEIYVLPFPTYKIPVDTNPG
HAIILGLQYLTSFIMSATVVIAFSLATVFACHAIGQLTIMISWIQEFVNQPQKQNKNIRIDEIS
VIIEHHLRILSFLERTEHLLSPICFMEMFKNILSICMFSYCILAEWSEHDVRVLGIYAFAVICIT
LNTFLICYIGEVLTERCKEIRNMVYMTNWYRLPDKDILNLIMIITRSGVEYKMTAGKIIDIS
VITFGNIIKTVFAYLNILRQMTIL
>AmelOR48
MADDIVAVQKKFGSLNEYSIQLNRWLSKMIGVWPLPSFTSKFEKIMTKILIFFYWIILLFIIL
ASSLHFLFVKEDIVSKLKTLGPISYCFGGGFNYAVLLLRKNDIRYCIEHIETDWKIIKRMED

QQVMLKSAKIGRIISGCIAGFMHIGTFCFCIVLGVLKRTIKIGNDSMEMYVLPFPTYKIPVD
TNPGHGIILSLQYLTSYTSSATVVIAFSLATVFAYHAIGQLTIMISWIQEFVNQPQKQNKNIRI
DEISIIIEHHLRILSFLERTEHLLSPICFMEMFKNILSICMFSYCILAEWSERDIRVLGIYTFAV
MNVILSTFLICYIGEVLTERCKEIGNMVYMTNWYHLPDKDIFNLIMIIVRSGVEYKMTAGK
IIDISVITFGNIVKTVFVYLNILRQMTIL
>AmelOR49
MADDLAKIEKKFGDLSEYSIQFNRWILKPIGAWPASFYKSRIEKIVSKILIVICWISSLFTLIP
GVLHFFLEKEDIYVKLKILGPLTHWLVGGFNYAVLLLRKDDIYYCIKQICADWNIITKKQD
QQVMLKNAKIGRYVAVFCTVFLQGGVFCTCLALGAFKKTIKVDNETVNIYNLPCPAYNMP
VDTNPTHDIILGTQLLSAFICSSSTAGAFSFVAICASHALGQLNLMVIWINEYVNRPKKLNN
NAYINKIGIIVEHHLRILSFIARVEHVMSPICFMEMIKCMVGICMPIYYILMEWSEHNIQNLT
VYVMIIISMTYNIFLVCYIGEIITEECKKIGDIVYMTNWYELSDKNILNLMMIITRSSMNINM
TAGKMTNMSVLTFGKIVKSIFAYLNVLRQITMI
>AmelOR50
MTNDINVAKQRSDNLSEYSIKLSRWYLKPLGAWPASSSTTKMERIISQILIVICWCIILFTVIP
GILYILFVKQDIYVKLKIFGPLSHWCIDGFNYAILLLRKNDILHCIEHLRADWKLITRTQDQ
QVMLRNAKMGRYIAAFCAIFMQVIIFFTCFILGIFKRSIHIDNKTVELYNLPCPAYKIPFDTD
PTIHDIMLGTQFLSAFVVSSSASASFTLATIFTCHVLGQLNIMMIWINEFVDRLQRKENKDN
HINKIGVIVEHHLRILSLIARIERITCPIYFMELFKCMMGMCMPSYYFLAEWSERNIQNLTIY
VMVALSMSFNILLVCCIGEILREQCKKVGDMVYMTNWYQLPDKDILNLIMIISRSSVEVKI
TAGKIITMSIYTFGNIVKTVFAYLNMLRQITMM
>AmelOR51
MRSTNNIDNLPLNDRYESDIQYTFQFCHWILKPLGIYYFIYNQANKFEKILSIILILICFFIIQF
VIVPFGYYILFYEKDMNTKIKFLGPLTFCLSALFKYSYLGIKSSELGHCIKHVEKDWKMLQ
NEDHRVIMSRYVIMGRNLITLCAAFMYTGGLSYHTIMPLLSKRKVENFTIRPLTYPGYEAF
LNIQKSPTYEIIYCMHCIYVIVVGNITMAAYSLTTIFITHACGQIKIQMLRLENLKNEKKVLE
TGIESHLAVVVKNHVEILRFAKNVETTLRELFLVEVIVSTLLMCLLEYYCMVEWETSDSAA
ILTYVILLFSFTFNILIFCYVGELLLGQGSEIATALYEIEWYNLPGRKARDIILLLVISKYPPKL
TAGKIFILSMNTFSVVLKSSVVYLNMLRTITEL

>AmelOR52
MFDRSYNNSQLKNIHYENDIHYTLQMCQWLLKPIGVWPFVYDRTSRFEQLISIILMATCFS
SLLFIILPSGHHIFFVEKDMHLKVKLLGPVGFCLSSTIKYCYLGVKGVFFEQCIKHVKNDW
KMVQDPSYRIIMLKYATISRKLIIMCAVFLYTGGMSYHTVMQFLSKEKDNNNTFKPLTYLG
YDPFFDTQSSPIYEIVFCMHCFAAMIMYSVTTVAYSLAAIFVTHICGQIQIQATRLQNLVEN
KDKKNNCDPFALIVHDHVEILRFSKNVEEALREICLAEIIESTIIMCLLEYYCMTEWQNND
AIAILTYFTLLISFTFNIFIFCYIGEILSEQCSQIGTISYEINWYKLPAKKAHDLILLISISQYPPK
LTAGKIIDLSFNTFSSVVKTSVIYLNLLRTVTD
>AmelOR53
MHDRSHDNINGQLKNSHYKSDIHYTLQMCQWLLKPIGVWPLIYNQTSRFEQLISIILMGTC
FSSLLFIILPSGHHILFVEKNLHMKVKAFGPAGFCLSSTIKYCYLGLKGSSFERCIEHMRKD
WMMVQDPNHRTIMLKYATISRKLITMCAVFLYTGGMSYHTIMQFLSKGKNKDNYTIRPLP
YIGYDPFFDTQSSPTYEIVYCIHCFTAMIMYSISTVAYSLTTIFVTHICGQIQVQIARLQDLVE
SKEKRKYKDCDPFALIVHDHVEVLRFSNNIEEALREICFTEIIECTIDMCMLEYYCIMEWSV
GDTITLLTFFTLLISFTFNIFIFCYIGEILTEQCSQIGTVSYEIDWYKLSPKEAYDLILLISISQHP
PKLTAGKIIELSLNTFSTVAKTSVVYLNLLRTVTDW
>AmelOR54
MHDRSYHDIESQLKNSYYKSDIHYTLQMCQWLLKPIGVWPLISNQTNKFEQLVSIILMITC
FSSLLFIILPSGHHYFFVEKNLNMKVKALGPVSFCVSSTIKYCYLALKGSSFERCIEHMKRD
WMMVQDPNHRTIMLKYATISRRLITICAVFLYSGGMSYHTVMQFLSKGKNNYTIRPLPYIG
YDPFFDTQSSPTYEIVYCIHCFTAMIMYSISTVAYSLAAIFVTHICGQIQIQIARLQKLVECKE
RKKYESCNLFALIVHDHVEILRFSNNIEEALKEICFTEIIECTLNMCMLEYYCLIEWSAGDTI
TFLTFFTLLTSFTFNIFIFCYIGEILTEQCSQIGTVSYEIDWYKLSPKEAYDLILLISISQYPPKLT
AGKIIELSFNTFSSVAKTSVVYLNLLRTVTDW
>AmelOR55
MHFSVRNLINKPRNPNYEKDITYVMKHNKWVLISIGIWPTVLKNIGKFLPKIVIGINNLMC
FFILIQSALHIILEQKDTLLRLKFFGLIFFSFMSLMKYWALTIRKPEIEHCIQQVQSDWKQVK
MENDRELMLKYGIIGRNLTIYSILFMYISGIMYISFMQYAMRLQINNDNQTNKVLIFPAYSN
SIQKSPIYEITYGIQCICGYVLDSVTSGACGLAALFVTHACGQIDVVISRLDDLVAGQFYKK

NSNPNIQVIKIIKHHIKILKFSAVVEKVLQEVFFLEFISSTFVICLLEYYCITDWEQNNIISLTS
YALLLISLTFNMFLLCYIGDLLIHKSGNIGVAVFMIDWYHLPAKTIQNLILIMAMSNSPAKLS
VGRIVDLSLSTFGNVLKTTFVYLNFLQTAVMQ
>AmelOR56
MYLSIQNPINEPRNPNYEKDIAYVTKYNKWVLTCIGIWPIILKNINKILPKIVIGINNLLCSFI
LIQSALHIIYEEKDVLLRLKILGLIFFSFISLMKYWALTIHKPEIKYCIEQVQSDWKQVEMEN
DRELMLKYGILGRNLTIYSILFMYMGSITYMSITQYAMGLQFNEHNQTIRVLIYPTYGYNIQ
KSPIYEIIYGVQFMCGYVVDTITSGACGLAALFVTHACGQIDIITSRLDDIVAGQFYSKNLN
PDIRLMGIIKHHIRILKFSAVVETILQEVFFLEFIGSTFVICLLEYYCIADWEQKNIISLTSYVL
LLISLTFNMFLLCYIGDLLIQKSSNIGVAVFMIDWFHLPTKTIQNLILIMAMSNTPAKLTVGRI
VDLSLSTFGNVLKTTFVYLNFLQTAVMQ
>AmelOR57
MHVSVRDPINELRNPNYEKDIAYVTKHNKWVLASIGIWPTVLKNIGKILPKIVIGFNNLLC
FFTLTQSALHIILEQKDTLLRLKFLGLIFFSFMSMMKYWALMIRKPEIEHCIEQVQLDWKQ
VEIENDRELMLKYGIIGRNLTIYSILFMYLSGIIYVSIMQYAMGSQINEHNQTIKMLIYPAYG
GYNIQKSPTYEIIYGVQCICEYVFDTIASGACGLAALFVTHACGQIDVIMSRLDDIVAGQYK
KNSNANIRLMEIIKHHTRILKFSAVVETVLQEVFFLEFVSSTFVICLLEYYCITDWEQKNIISL
TSYILLLISMTFNMFLLCYIGDLLIEKSGNVGVAVFMIDWYHLPTKTIQNLILIMAMSNTPA
KLSVGRILDLSLSTFGNVLKTTFVYLNFLQTAVM
>AmelOR58
MHLFVRDQTNQPRNLNYEKDIVYVTKHNKWILNSIGIWPTVLKGIDEYLPKIAIALSNLVL
SFTVIQCVLHILLEQKDPILRLKILGLTFFSFISLMKYWVLTMRKPKIKLCIEQIQHDWKQVE
FERDRKLMLKYGIIGRNLSMYSIVFMYSGGIIYHTVMHYKLGSYVDEYNRTIKLLIYPTYS
RLYDVQKSPVYELVYILQCICGYMFDAVTVGACGLAALFATHICGQIDIVMAKLEDLVDG
KFSKENSNPNIRLIEIIEHHIKILRFSAMVETVLQEVCFLEFIGSTFVICLLEYYCITDWQQNN
TIGLTTYSLLLISLVFNIFLLCYIGNLLIEKSSNIGIVCCMIDWYQLPIKTIQGLILMIAMSNSP
AKISAAGIADLSLSTFGSVLKTSFAYLNFIRTTIM
>AmelOR59
MHPITLNESDCKARNLKYKEDIAYVTKHNKWILKSIGIWPSIFKDVSKFLPKIMFGLCNFV

LFFAIIPCILYIVIEENDTMIRFKLFGLLSFCLVALIKYWTLLYRKSRIKNCVEQIWIDWEQVE
LYEDREMMLKYGQMGRNLMIICAMFTYTGGTIFHTILQYKVGTFIDEYNRTIKPVIYPTYN
GLFNVQRSPIYEFVYILHCMCGYVMHSVTAGACGLTALFATHACGQIDIVIARLNDLIHGK
YSKEKINLNARFTKIIEHHLRILRFSATVQEVLQELCFLECIGSTFLICLLEYYCITDWELNN
TISLTTYIILLISLIFNIFILCYIGELLMEKSSNIGLSCFMIDWYYLPSKTIRGLILMIAISSNPTK
ISAGGIVDLSLSTFGNVLKTSFAYLNFFRTTIM
>AmelOR60
MHLTILNESDYRARNLKYKEDIAYVTKHSKWILKSIGIWPIILKDVAKFLPKIVIGISNFVLL
FAIIPCILYIILEEKNNLIKLKLFGLLMFCSIALMKHWALAYRKPKIKNCIEQIQNDWEQVKL
YEDREMMLKYGQVGRNLTIICAVFMYTGGIIYHTILQYEIGTFIDEYNHTIKPVIYPTYSGL
FNVQKSPIYELIYVLHCTCGYVMYSITAGACGLAALFVTHACGQIDIVIARLNDLVHAKYG
KGKFNLNARLIKIVEHHLQILRFSATVQVILQEVCFLEFIGSIFLICLLEYYCITDWELKNTIS
LTTYIILLISLTFNIFILCYIGELLMEKSSSIGLSCFMIDWYHLPVKTIQGLILIIAISNSPTKISA
GGIVDLSLFTFANILKTSFVYLNFIRAAIM
>AmelOR61
MHLTTLNKNNCKVRNLKYKEDIAYITKHNKWILKSIGIWPSVLKSVSRFLPKIMFGFNNFV
LLFSVIPCILYIVYEEKNIMIKFKLVGLLSFSLIALIKYWTLTYRKPRIKDCIEQIWIDWEQVE
LHEDRKVMLKYGQIGRNLTIICAVFIYTGGSIFHTILQYKIGTFIDEHNRTIKPVVYPTYNAL
FDVQKSPIYELVYLLHSICGYIMYSVTAGSCGLTALFATHACGQIDIVIARLNDLIHGKYTK
NTFNLNTRLVKIVKHHLRILRFSESIEMALQELCFLECIGSTFLICLLEYYCITDWELSNTISL
TTYTMLLISLTFNIFILCYIGERLMEKSSSIGLSCFMIDWFQLPTKTIHDLILIIAMSNNPIKIS
AGSIVDLSLYTFGGVLKTSLVYLSFLRTTIM
>AmelOR62
MGKRKESIDERIRNFMVQKMVLKIIGIWPTNGERSFFGRWIFAVTTQIGIIYILSLEIYRHCL
DIDDTMDAFVMDLSAVISLAKLFILRLNSKHAWVLINSVVEDWSAVHDSRHEYIMTEYLK
KGRIVSLMILYLGYASGFSFIVKALPFGDILPFQMFQNSRNSSMNPDIPLKLNYFLASYCVF
GSLPLLHHVCVLLLQGIFIFVNAVAHCGNDGLFFSLTMHLCGQFEILKTRIAKIEFVDRRKI
GPLVKRHCQLAVLVNDLEQTFNMIIFVQLLMSALLICVEGFVFLVCLSTKDNIGALKSMVL
MVTLLIQLYLYAYAGDALESRTEEIAQAAFHSFWYQSRGRTARDLILIICRGNSSYHVTAGK

FVFMNIFTFKEILKSSASYLSVLKVMMDT
>AmelOR63
MLKKMKTTSNKDFAYAMTPLKFLAWPVGTWPLQVFNTFSIIRATFSTFLLLLMLTILQVEL
YLDSSNPEYNLDALILINAGILAVTKVICFHVRSLGLVSNFTSAVKDYKELNSEENRVIVRR
HAYMGRAACISLIFCSYVGCTLFMIVPIVAGDKEEVINVTEESAMKYPVPFENTLILINMPE
NMYFLIFIVEYLMLLLTTTGNLGSDSLFFSIVFHLCGQVEILRLEYNKLSNENERTTKHITLL
IKRHIYLLKLGDMLNKTISSILIVQLSSSCMLICTTGFEFILALSIGNIVMIVKTFAVICVLLIQ
LFAYSYVGEYLKTQTEGLGNSIYFCTWYDMPKNVSHNITFIIMRAQHPVLLTAGKFFVINM
ETYMSILRISMSYLSVLRVMVNS
>AmelOR64
MKTTSNKDFAYAMTPLKFLSWPLGTWPLQVFNTFSIIRAMFSTFLVLLMLAILQVELYLDR
SNAENNLDALVLINGGILAVAKVMCFHIRPLGLISNFTSAVKDYNELNSEENRVIVRRHAY
MGRVACASLIFCSYVGSTLFMTVPMLAGDEEEVINVTEESAIKYPMPSENTLTLINMPEKM
YFVIFIVEYLMLLLTSTGNLGSDSLFFGIAFHLCGQVEILRLEYNKLSNENERATKDIILLTK
RHIYLLKLSDMLNETISSILIVQLFSSCVLICTTGFEFILALNIGNIVMTIKTFIVMCVLLIQLF
AYSYVGEYLKTQTEDLSNSVYFCTWYDMPKNVTQNIIFIIMRAQHPVFLTAGKFFVVNME
TYMSILKTSMSYLSVLRVMVNS
>AmelOR65
MKTTSNKDFTYAMTLLKFLSWPVGTWPFQVYDTFSLTRTIFSISLLLLMIIIVQVELYLDRT
NAENNLDALLLINCGILAVGKVMSFRVRSTGLVFNFTSAVKDYNESNDEENRMIMRRHAY
MGRVACTSLISCSYVCSTLFITVPMLAGDEIQVINATEENAIKYPIPSKNALEIINMPDNLYF
VVFIVEYMMLLFTSIGNLGSDSVFFGIVFHLCGQVEVLKREYSKLFNKNEKITEHFILLIKR
HIYLLNLSKMLNETISSILIIQLFSSCVLICTTGFQFILALSIGNIVLTIKILIIMCVLLIQLFAYS
YVGEYLKTQTESVGNSVYFCTWYDMPKNVSKDIIFIIMKAQRPVLLRAGKIFVVNMETYI
SILKTSMSYLSVLRVMVNS
>AmelOR66
MKTTLNKEFAYAMTPLKFLSWPVGTWPFQVYDIFSLTRTIFSISLLLLMIAIVQVELYLDRT
DAENNLDALLLINCGILAVAKVMCFRIRPVGLVSNFSSAIKDYNELNSEENRVIVRRHAYM
GRVACASLIFCSYAGSTLFMTVPMLAGDEEEVINVTVESAMKYPIPSKNILAIINMPENMYF

VVFIIEYIMLLLTSTGNLGSDSLFFGIAFHLCGQVEILRLKYNKLSNENERTMKHISLLTRRH
IYLLKLSDMLNETISSILVIQLFSSCVLICTTGFEFILALSIGNIVMMIRICIAMCVLLIQLFAYS
YVGEYLKTQTESLGNSVYFCTWYEMPKNVSQNITFIIMRAQHPFLLTAGKFFVVNMETYM
SILKTSMSYLSVLRVMVN
>AmelOR67
MKTTSNKDFTYAMIPLKFLSWPVGTWPFQVHEIFSISRTIFSISLLLLMVVILQVELYLDRSN
AENNLDALLLINCGILAVAKVMCFRIRPIGLVSNFSSAIKDYNELNSEENRVIMRRHAYMSR
VACASLISCSFIASTLFMTVPMLTGDKKDIINVTEKSIIKYPIPSKNALAIINMPENLSFMVFI
VEYMMLLFTSTGNLGSDSLFFGIVFHLCGQVEILKLKYNKLSNTNERTMEHIILLTKRHIYL
LNLSKMLNETVSSILVIQLFSSCVLICTTGFQLILTLTFGNVVLTIKILAEISILLIQLFAYSYVG
EYLKTQTEGIGNSVYFCTWYDMPKNVSKDIIFIIMKSQRPVLLTAGKFFVINMETYMSILKT
SMSYLSVLRVMVNS
>AmelOR68
MTILQPIFNILTICGCRMPSSCRTSYKRMLYILYATFVLLLLYSFCISQFLNVIINVRTADELCN
SFYMFIASLLSCCKIVALLMNHKAIKIFRRKLEEEPCKPTNTKEVTIQKSFDKNIGSITIYYT
VMVEFTVFCMIVSSLVTDFRNQRLAYEAWLPFNCSAPNYYYYYIAYVHQIIALIGTSLLNV
ACDVTICGLFVHMYSQQEILKHRLKESVNVENRLNIGKIVYFHNYLYGYAFMVQEKFKKII
GIQLLSSTLVVCFILYKLANTSLISTKFLEFVLYLACMMTQIFVYCWYGNQLKLKSVEVVD
TIFELDWISLDNRSKKDLINIMRRAMNPIELTCAYIFTIDLRTFVTILKMSYSTYNFLQRTKVN
>AmelOR69
MQLLRTIYHLLTSCACWRPPFLSPLKNFAYTVYYCYVILLIYGATFCQFVDLLLIVETEDEF
CDNFYLTLAIFISCHKMYSMLVNRENIILVNRMLESEPFQPETEEEMDMRDKCDKQARLN
AIYYAILVELSVMSLSFGGLLKAESHKLPYRMWLPYNYTSLSAHIFIYTQQVVSLIVSAMIH
VACDSFIWALLMHICSQIEIFNCRLRKIKHEKNEVTKLCIHYHNLIYRLATTINEQFKMVIFV
QFTVSTLTICVNLYILMGTQITFERIMQLAIYSSCMLTQIYIFCWYGNEVKLKSLDISNMIFE
LDWPDLDNTTKRDLLMIMMRASYPIEMTSVHVITMNLDSFVILLKTSYSAYNLLQSNRE
>AmelOR70
MQALQWTRFLLSVCGCMPPTSWKSSFKKSLYNIYTCVIWLLILSLVSTQILDIIINVKNKNE
FIENFYITLVVFVTSCKMTIILRYRKNILSLMDDLQHEPFSPMTHEENEIRTKFNKMNERTSI

CYTILVLVSATWIFVRSFFTDFKKRKLTFRAWLPYDYSELLPFALSYAHQATTSMFCSCQNI
SCDTLFAGFLVQIYCQFEILEERLKNVQQDESNYSAKQCVKHYHQIYKFSRTLNEKFKVIL
FLQFCAIAFILCFNLYRMTTITMIPKLLEASLYLIRVLVQILYYCWFSNEVKLKSLEVPGMIF
KSDWTSWDDKTKKIFLIIMTRATQPFEFTSGYLVTLNLEFFVALIKASYSVFNLLQRTK
>AmelOR71
MRILRWTFLLFALCGCFPPSSWTTRLKRYLYKIYAVFSFVALNSFLLSQILDMVYNVKGTD
DFSDNFSVTVVVFVTCFKLITILTRRENILLLCNTLKQEPLSPINTEEFEIFLKFEKLTDWNTL
GYFILLMSSSLCILMGSLLANFKIRKLAFRTWLPYDYSTASAFLLAFAYQVVVATVCTFACV
ASDTLYSGLLIHISCQFEILEHRLKNIGSDKNYTMKQCVRHHNHIYKYGEMVNDAFQSIMF
FQFCTSLSMICFNFYRIMQIEMDSRYVGTILYMVCSLMQIFYYCWFSNEVKLKSLELSDMI
FRSNWTSLNNNVQRAILLVMRRSMKPIEFTSIYIVSVNLDSFMTLLKSSYSAFSVLQQSRES
>AmelOR72
MHLLRWTFKLFVATGYFLSPKIKSPRKRFLYNVYTVVVTLFLLSFLLTLIMQIVFNVRTADE
LSENFGITITVFTTICKFINLLFRRGIIISLLDLLQKEPFLPMDIEEIKIHTKYNKLIEKVSIFYT
LQNVSCLVALIGATLITDFKKKKLTFEAWIPFNYTASWFLFSLTFIHQCGCAVVTSFGISIFDT
LFAGLLLQVCCQLDTLVYRLQNIKEDAIQSLKYCARQHELIYRFTELMNKLFSSILCLQFLI
SAVAICFSVYRVIYTKTDSQFAGAIIFVFSALIQIFYFCWHGDIAKYKSLEIPDMIFNSNWPN
LSNEAKKILLIIMARSLTPVEVVSAHIIPLNLESFKRLIKATYSAYNMLQQTK
>AmelOR73
MHKLSLSFALLTYGGYWRPTKWPASSYKYHLYNIYSAFMIFLLYFITFCTCVDSLISKNLKT
MSEKFSLCISVFGVSLKVANLFLQRGKIINIMNSLTKENSIPRDEQEEIIQRRNDNYARKVTI
YCEILNESAVFFATVGQYKRFINTRTLPVSDWIPYDLSSTELYIISLLYQTVGLLICANASVG
NETLIAGLMIQAGVQFEIFCHRAQNLPSLVLTVTRNSNVFAETVNTVFQYMIFLQFTISSVV
LCLSIYKFSTVDPLSMNFVWSGFYLCCMLMQVYLYCWFGNEVTLKSNKVSDAIYEMDW
TILPSNVMKDLLLVIARSKKPVKITSGQIFILSTESFMKIMKISYSSFNILKNSTMK
>AmelOR74C
MQGEGYTDVSLKVSQFLLKSAGIWVIGNDAEERQRKFAVFYTLAALIYGIYVNAVDIYHN
LDNLAHCVFLTCNMMCILLGLFKCFVISFFRIEFSRIVSYAQKHFWRLDYDYDEKILFGEC
QKFCRLWIIVVSMISQSSLAFYIITPIYENIGKNKSERILPFKMWVDLPLSVTPYYEIMFVIQL

LAVEQIGIAYVCSDFFLCILNLHALYQFRMMQQELSKIWSAIEQQTTSVAAYTRGCHVALK
KCIRRHQSLIEFCNKLEQVFTFPILSHVVVFSLLMCFDTYEILLADIPTLKRLIFLCHMVASFI
HIIFFTYICHGLMEESGNVGLATYSGWWTTLPMNETGRMLRKDIRIIMMKSMRPCYLSRS
GFFPMSLETSTA
>AmelOR75
MRRRGSKDVSIIWTSFLMKIVGLWLATDRNEQRQRDFALIYTVGTLFISICIAFRDIYYSWG
NFSNSVFICCNILYVAIVLLKISVLYAHREEFFNLIAFTQKNFWRLYDDPQELLIITGCKKLC
NFSIVLIIFCAQGTCAGYMVTPLIENIGKNESDRALPFNLWIDFPVGLSPYFELLFILQILCVY
HVATCYICFDNLLCIVNLHVAGQFRILQHRLKNLGNAIRDETGLPRYEKCCYERLKDCVVQ
HQTLIEYCKRLEDIFTVMVLGQVMFLAVVICLVGFQLFLADTSASKKASLVLNLGGTFFQL
LIFTYSCDNLIRQSVNVGNAVFSGPWVNLPMSKAGILVRKNLIIVIMRSQKICCLTAGKFFP
VSLETSTAVLSTAISYFTLLKQSSLENM
>AmelOR76
MKSKEVRDLSITVTAFYMKIAGFWTSTNYVEERRRNVTMSYTLFAILFAATTEARDLYFS
WGNFSDSIYVACNIITVSLVLIKLLTSFIYNEELLGIIRYAKTNFWHSNYDTCEKSIMNKCQR
TCNYLVFVFTFFAQGTVLGFILRPILVNRGKNESDRILPFNMWLELPLSITPYFEVMFFVQV
VFVYHVCVCYHCFDSLLCILNLHTASQFRILQHRFANTCNEKRGKRDEDEESALSFYEYSK
LKAYIRQHQALIEYCKKLEQVFNSIVFGQVLLFSLLMCLDGYLILMEETPFGRRVTFTFHIT
GCMCQLLMFTYSCDCLIRDSMDIADAAYNCSWSFLPMDKYGKMIRRDLMFVITRSRTPC
CLTACGFFAVSLETYTKVLSTAISIFTILKRYEKEFKSDSS
>AmelOR77
MNGLLNGGDASMTMTAAFMKLVGLWTAKNRREQRARKFALIYTVAAMLFALWIEFTDF
YYSFGDFSTCLFNTCNIIYITMPLLKIFVIVLNKKDFFHLIFYTEKHFYKDNYDEHEQRIFTN
CRRQCIIFVCFLTFSTKGTLVCYIVSPLVENIGKNQSERALPFNMWVNLPLSTSPYYEIIFTIQ
VLSLYHIGVGYFCFDNLLCVLNLQLAGQFQILQYKMANIVDLLKEKNEKRIINTSYFAKKC
YEAFKKCIREHQALIAYCEKLEKVFSLIILCQVLTFSLIICLDGYQIIL
>AmelOR78
MQSENQLDVSITLSTFFLRNIGLWMSDDPGEQRRMRILLVYTVWILLLGMIINGRDLYFTFL
YNGDILYALTNNVTMVMGLIKIYIILLYKGKFLNLIVHMQQNFWNVNYDYHEKEILDDCR

KTCIFFVSSLTTIGICAMLSYLMTPFAIRSGNNESERMLPFNMWLDMPLSKTPYYEITFLIQ
AMCVYYIGISNFCFDTVFCIMAVHLAGQFRILQYRFTKLCDTDNQICKKNLILEEQMQKFH
EKFKKYVRRHQALIDYHQKLENVYTTIMLSQVLLFSVLICLFGYQVLLATASLARRSIFIFL
LMGAMFLLFMFTFSCNGVMEQSDNVAVGTYSALWTVMPMEKFGRMLRKDLIMVIMRSR
RVCCLTANRFFPISLETYTKILSTAVSYFTLLSKHVDNS
>AmelOR79
MQAEYRLDISINLSTFLLKNVGVWMSHDPGEQRRMRMLLVCTVWMLLLGIVINTRDLYF
TMLYNGDILYVVTNNITLIISLVKICNIIIYKGKFLNLIVDMQENFWNVDYDYHEKEILDDC
KKICIFFISSVTTIGICAIISYLMTPFVAQSGSNESERMLPFNVWITFPVTRTPYYEIIFFIQAICL
YYIGISSFCFDNIFCIMAVHLAGQFRILRYRLTKLCEQEIYEKDSTLTKQMHKFYEQFKECV
RHHQALIDYHQNLENVYTIITLGQVLVFSVLICLFGYQVFVATASFARRSIFVFMLNGSMFL
LFMVTYSCNGVTEHSDNVAIGAYSALWTIVPMDKFGRMLRKDLIMVIKRSRRVCCLTANG
FFPVSLETYTKILSTAVSYFTLLNNRIENANGL
>AmelOR80
MQTESQLDISINLSTFFLKNVGIWMSDNPNEQRRIKMLFLYTIWNLLFGTVVNSRDLYFTL
LYDGDILYVTTNNITMIMGMVKICIILIYKKKFLNLIVYMQQNFWNVNYDHREKQILDDC
RKTCIFFVSCVTIMAICAMICYIMIPFIAQSGSNESERMLPFNMWINLPISRTPYYQITFLIQA
TCVYYVGISYFCFDNIFCIMAVHLAGQFRILRYRFTKLCDMEYGIKENSQSILSKQMHKFY
EKFRKCVQHHQALIDFYQNLENVYTMITFGQVLVFSVLICLFGYQVLVATISFARRFIFVFM
LNGSMFLLFMVTYSCNGVIEHSDNVAVGAYSALWTIMPMDKFGKILRKDLIIVIRRSRRVC
CLTANGFFPVSLETYTKILSTALSYFTLLSNRIENSS
>AmelOR81
MQTESQVDISMNLSTFFLKNVGVWISDNPSEQRWRNMLLGYTTWILLSGIIINGRDLYFTL
LYNGDILYATTNNITMIMGLVKICIILMYKKKFLNLIVYMQQNFWNLNYDHCEKRILDDCR
KTCTFFVSSVTSMAICAMICYLMIPFIVQSGKNESERMLPFNMWINLPVSRTPYYEIIFFIQA
MCVYYVGISTFCFDNIFCIMAVHLAGQFRILRYRFTKLCDVEYENSQSILSKQMQKFYEKF
KKCVQHHQTLIDFYQNLENVYTTITLEQVLVFSVLICLFGYQVLVATASFARRFIFVFLLNG
SIFLLFMVTYSCNGVIEHSDNVAIGAYSALWTIMPMDKFGKIFRKDLMIVIRRSRRVCCLTA
NGFFPVSLETYTKILSTALSYFTLLSNRVENA

>AmelOR82
MQTESQVDISMNLSTFFLKNVGVWMSDNPNEQRQIKMLLINTTWILLSGIVINGRDLYFTL
LYHGDILYSITNNITMIMALIKISIIIIYKGKFLNLIACMQQNFWKVNYDYREKEILNDCRKT
CIFFVSCLTTMVICAMISYLIIPFIAKGNNESERMLPFNMWINLPLSKTPYYEIMFLIQAMCV
YYIGVASFCFDNIFCIMAVHLAGQFRILQYRLTKLYDVECIEMHKKDSILANRVPKFYEKFR
KCVQHHQALIDFYQNLENVYTRIAFGEMLVYSILICLFGYQVLVATASFARRSIFVFLLNGS
TFLLFMVTYSCNGVIEHSDNVAIGAYSALWTIVPMDKFGRMLRKDLIMVITRSRRVCCLTA
NGFFPVSLESYTKILSTALSYFTLLSNRVETANDT
>AmelOR83F
MQTDNQLDISISLSTFFLKNVGVWMPDNSDEQRRMKMLFLYTIWMLFCGTIISTRDLYFTL
LYNGDILYAMTNTITTIMALIKICIILTYKGKFLNLIVYMQQNFWNVDYDCQEKEILDDCRK
TCIFFISSVTTIGMCTVMSYLTTPVITQSGSNESERMFPFNIWINLPITQRTPYYQIIFFVQGVS
VYYIGISYFCFDNIFCIMAVHLAGQFRILRYRLMTLCDTEPETREKDSRSTFAKQVYKFYEQ
FKKCVRYHQALIDYYQNLENVYTIITLGQVLVFSVLICLFGYQVFVAAASTARRFIFVFLLS
GSMFLLFMFTYSCNDVMEHSDNVAIGAYSALWTILPMDKFGRMLRNDLIMVIKRSRRVC
YLTANGFFPVSLETYTKILSTAVSYFTLLNNRVENA
>AmelOR84
MRSTRDISIIWTSFLMKIVGLWLAADRDEQRRRDFALIYTVGALFIIVCIGFRDIYFTWGNF
SDSVYISCNNLYLMIVVLKVGVLYAHKMEFFDLVTFTRNNFWRSYPDPEEELILAECKRIC
TIFVVVISFCAQGTCTGYMITPIIANVGRNESDRELPFNLWVDLPVGLSPYFEILFTVQILCV
YHVGVCYICFDNLLCIVNLHVAGQFRILQHRLRNLNVAVTGDRESYRANVCHAKLRSCVI
RHQTLTKYCKQLENIFTIIVLGQVLFLALVICLVGFQLFLMDTPASRKVSLTLNFAGTLCQL
LMFTYSCDDLIRESVNVGNAVFSGPWAELPMDKVGRVVRKNLIIVVARSHRVCCLTAGKF
FPVSLETSTAVLSTAMSYFTLLR
>AmelOR85
MSSNKVGGDLSITVMTFYMKIVGFWIASNYVEERRRNLTISYTFFAIFFAMATEARDLYFS
WGNFGDSILIICNLVTVILVLFKISISLMYRNKLHKIIQYAKTNFWNLKYDLHDEQIIINTCK
RYSTFFVCIFTFFSQGTVFSFVIRSLKENIGKNETERIHPFNLWLDESWYMTPYFEMVFIIEIL
SLYHVGVCYLYFDNFMCIINLHIAGQFRILQHRFSNVCNEMCEKCCYQLSRKSPYLSICKY

AKLKIYIRQHQTLIEYCRKLEMVSNFIIFGQVLLFSLLICLDGYLILMEDTSNMSRLIFTFHLI
SCMCQLLMFTYSCDCLIRDSTNIANATYNSLWSFMPMDKYGKMLRKDLILVIMRSKSPCY
LTALGFFPVSLETYISILSTAISYFTLLRNRAEQTIMDA
>AmelOR86
MHATPYSDVSIVVSQFLLKLTGVWMTVNDGEKRRRRIAMAYTFVIQVYGLYLNIGDIYHS
WDDLSHCIFLTCNTLCIVLTMFKFSILFIRRTEFKNLILFARKNFWHLDYDRHETILFTKCRK
FCTLWTLTVFSFTQASLTFYIITPICANIGKNKSERILPFKMWVDFPLSETPYYEIMFVIQLLT
VQQIGIAYTCNDNFLCVLNMHVVCQFRILQHRLTKLWSIIDERADKFNYASKCYEALKECI
RQHQSLIEFCDKLEHVYTLPIFGHVVVFSLLMCFDTYEIFLANVPVSMRLIFFFHMVGSFIH
IIFFTYICGGLIEESSNIGLATYSGWWTVLPMDEAGRMLREDVKVMIMKSMRPCHLSAGG
FFPVSLETSTALMSSTLSYFTLMRESSKDK
>AmelOR87
MGAKAVAKVVAHSKLVARHSNKDFALSMTAFLMKIVGLWLAKNEQEQRKRRLTLMYTVI
AILFGVWVQFRDFYYSWPNFGNCAYTACNILCLIMVLLKLFVLFVHRKEFIDLLVYTHENF
WHTNYTNNELLLLQNCKRISMLCITLINVCAQGTIVSYVLTPIVENIGRNHSDRVLPFNMW
VDLPTLFLSPYYEILFVLQVLSLYHVGVCYICFDNLLCLMNLHAATQFRILQHRLSDLGSG
WDTRRSFNKIDRETSWSSCMENCYATFKLCVKQHQDRITYCHRLNDIFTIIVLGHILVFSLL
MCLVGFQVLMANSPPTRRLIFVFHITGSLCQLLLFTYSCDSLIQESTNVGSAVYSGPWICLP
MNRIGRTLRRDLRMVIIRSRKPCCLTASRFFPVSLETCTTVLSTAMSYFTLMRQSFAN
>AmelOR88
MNGRNVRNLSITVTAFYMKVAGFWVANNYAEKRRRNVAMFVTIFFAFMGISIEGRDLYFA
WGDFEDSIFAGCNVITIVLVLLKIFVLYINNEELLNVVNYAKTNFWRESNYEPHEKKIIDDY
RRLCSFLVCSFTFFAQGTVVCFVITPVFVNNGKNESDRIHPFNMWFDRSLSLSPYYEIIYTIQ
VLSAYEIGICYHCFDNLLFVINLYTAGQFRILRYRFENICGKNDDKNYYKVSKSSKYCINEY
KSFKTCVQQHQALIEYCKKLEDVFSIIVLAQVLLFSLLICLDGYLVLMEDTSRAKRVIFTFH
LMGCMCQLLMFTYSCDCLMHDSMSVANAAYNSLWPCLPMDKYGKSLRKDLTFVIMRSR
SPCCLTACGFFPVSLETYTGILSVAVSWFTSLKKYEKKLLQYACIANQSLLKEEESKIIFYHQ
NI
>AmelOR89

MQREVELDVSVNLAAFFLKNVGLWASNDPGHERRRKVILVYTMWCVTLSSVVIIRDVYF
TWFYNGDILYVVTNALSMMMITVKVCVIVVHKEEFINLIVYMQENFWNDNYHDLREREI
LENCKRTCAFFVSLVTAIGICAILSYLATPLIVQTASNNSERMLPFNMWLKLPLSESPYYEL
MFVYQIMTFYFIGISYFCFDNIFCIMTVHLAGQFQILRHRFDRLCNAEDRIAEKGAHAREF
YDRFKARVPYHQALIDYCEKIENVSPTIILEPVMVFSVIICLFGYRILWANAPSTRRSIFIFLLI
GAMSLLFMFTFSCNCVTEHSENIAIGAYSALWTAMPMDKFGKMLRNDLIMVIKRSRRVCC
LTANGFFPVSLETYTTILSTAVSYFTLLRNNVEKANEA
>AmelOR90
MEKELDISVNLSSFFLRSIGLWIGDGSTNERRRKGMLAYTIWCTFFSTIISSRDLYFTWIYNG
DILYALTNYMSVMMILLKICVIVVHKSEFINLILYMQRYFWNVNYDSREKEILNGCKKTCA
FFVSTVTFIGICAILSYLTTPFTARIGNNESERILPFNMWVNLPLSQTPYYELLFLIQIITLYYI
GICYFCFDNVFCIMAIHLTGQFRILGYRFAKLCNIEHEMREKDTVLSKHVHTCYEKFKEYV
RYHQALINFYTKLENVYTMIILGQVIVFSALICLFCYQVLLANAPSARRSIFIFLLIGAMSLL
FMFTYSCDGVIEQSDNVAVGAYSALWTIMPMDKFGRMLRNDLIMVIERSRRVCCLTANGF
FPVSLETYTTILSTAVSYFTLLRNNMENDKDD
>AmelOR91
MSFLESDVSVSLTSIFMKLVGLWMAADQYEQRLRNISVTYNLVAILFALYLQTTDIYYSWG
NFSACLFSVSNTLSLILPLLKIFILLSNKEDFFRLIVYMQRNFLQGNYDDHERKIVFGCKRK
CTFFICFFTFFTMATIVSYIAGPIIGNIGKNESDRVLPFNMWINLPLSMTPYFEITFTLQVLSL
YQIGVSYFCFDNFLCIMNLHLAGQFKVLQYRISTIADRVIEKEEKKEKLIIDSLYFSNKCYTT
FKKYIRQHQALIAYCRKLEVVFNWIVLEQVLMFSLLICLDGYQILMANGDIKTRLTFSFHIL
ACLCQLLMFSYSCDCIIRESVSVATAAYGGPWTLLPMTISGRMMRKDLIIVIMRASIPCCLS
GKGYFIVSLETYTSVLSTAASYFTLLRNNIESDN
>AmelOR92
MNFLENDVAVSLTSIFMKFVGIWMXQYQQRMRNIMVAYNVIAIFFALWIQTMDMYHSWG
NIRACLFSTSNTLSLILPLLKIFILLCHKQDFFRLVLYMKRNFLXNYDDHERKIVIGCNQKCT
FFICFFTFLTIATTASYMVIPLIVNIGKNESDRVLPFNMWVNLPLSMTPYFEISFVLZVLSLYQ
IAVSYFCFDNFLCIMNFHVAGQFKVLQHRISTIADLTIKTEEKKEKLIIDSLHFSNKCYTTFK
KYIRQHQTLIAYCRKIEVVFNWIVLEQVLMFSLLICLDGYQILMADEDIKTRSIFSFHILSCL

CQLLMFSYSCDCILRESVSVATAAYEGPWTLPPMTISGRMMRKDLIVVIMRASIPCCLSGK
GYFIVSLETYTSVLSTAASYFTLLRNNIESEIMKHD
>AmelOR93
MNFLESDVSXIFMKLVGIWMAGNGZEXITLFTAIIFSGYNLZMYFILGDFSACLFFISIILSSI
MLLLKIIILFSHREDFFHLILYMKRNFLXNYDDHERKMIGCNZKCTSSSVSSRFSRWRPLLL
TSSVRLLVKNIGKNESDRILPFNMWVNLLLNITSYFEITYTLQXFSLSLYHIGVSYFCFDNFL
CIMNLHVAGQFQVLQYRISNIIYXRFNEKKEKLIVDSXATKCYAIFKKXQHQALIYLIYCRK
LEEVFNLIVLEQVLMFSLLICLDGYQILMADGDVKTRLIFSFHILGFLCQLLMFSYSCDCIIR
WTLXPLLSMTSSRRMIRKDLILVIMRSNVPCYLTGRGFFIVSLEMYXVLSTAAXFTLLKQR
TEATS
>AmelOR94
MTLKYRGDVSFSLATFFLRVVGFWLASSRLEEWFGNATVMYSIITIIFSMWVQMRGLYFS
WGDFGVCTFIVCNSLGLVMDLLKILVVFVHKKKFLGLIAYMQKNFWHLDYDQRENSIIAD
ARQLCVYFVCVFSFFSQSTVFSYMFMPMISNIGKNESDRILIFNMWLDLPLSMSPYFEIIYV
IQALCLYQVGICYLCVDNMFCIMCLHLASQFRILQYRLANVSNVEDEEGVEENMNSSNRC
YAILKNCIRYHQALIQFSITLEEIFTIITLGQVLIFSTLICFVGYQVLLVNMTLSWRISFLCFLIT
NMCQLWMFTYSCDCMTRESVNVASAVYCIPWTRIPMDKFGKMIRKDLQFIVVRSRRACC
LTGCGFFDISLETYTKIMSTAMSYFTILKQRIVEVENT
>AmelOR95
MKNDDFSINLSSIFIKLMGIWMANDQSEKYVRNVTILYSIIALLFGLWLQITDMYYSWGDF
SECIFSMCNMLSIAAPLLKLITLVVHREDFFYLILYLQRKFLHGDYNDYERNIVLNCKRKCT
FFTCSLTFTTLATVVSYVINPLVANIGRNESDRVLPFNIWIDLPLTITPYYEITFVLEVISLYHI
GVSYFCFDNFLCIMNLHVAGQFQVLQYRISNIIDSIDKEKKEKLIMDSCYFASKYYAIFKKC
IRQHQALIAYCRKLEEVFNLIVLEQVLMFSLLICLDGYLVLMADTSTTTRLIFGLHITVCLC
QLLMFTYSCDCIIRESLSVATAANRGPWPMIPMTTSGRMMKKDLILVIMRSGTPCCLTGRG
FFVVSLETYTNVLSTAASYFTLLKQHSEAHS
>AmelOR96
MNLKYRKDLAFTVASFYLRVVGFWLTTSRLEEWFRIGVVGYTILAITFSAWVQIRGLYFN
WGDFSACTYIACDGLGLVMDFFKIFSLFIYEKKFLGLMVYMQKNFWHYNYDEKEDLIVK

DTKRITAYFVCILTFSSLSSIFTYMFRPLLTNIGRNETDRILIFNMYLDLPLSISPYYEIAYTIQI
AALNQAGSCYFCFDNIFCILCLNVACQFRILQYRIANVPILKMKGNPDANKNSSDECYKAF
KNYVQQHQALLDFCETLEEIFTIIVIGQILMFSILFCFLGYQVILADLTPSYRISFISYLFAGM
CQLWMFTYSCDCITQESAKIASAAYASPWINLPMDKFGKMLRQDLQIVVMRSRRACCLTA
CGFFPISLETYTKIMSTTMSYFTLLKQRTVDT
>AmelOR97PSE
MKXFQKNGKYKSTVFDISYYKTFKKYLKFLGQYPNQSRWNKEFNTNVMICSLISFLIPGLSRVYISIVEKNLNALMEIIPIVFATISCAIKLLNHRINKKNFDKLFDLMSKE
WEMKNDRNQTCILDEFTKQGNKFAEIYKNVLLSALLLFLLLPLFPSFLDIVFPLNETRQQFQIFKMKYFVNEDEYFYPIYFHSVWSSFVIIMITVTIDSLYMLIIHHASGLF
AMCGYQIAKATECNDRHNENELFRQCVMTHNKAYKFFEIMNKSSRNSYFLQISLTIIGISIIAVQIVMYLHKPEEAFRISLFLIAAQFHLFIITLTGQVIADQSSKLSNNMY
CTTWYRMPPNVQKIFHIIQIKSSKPCKLTAGGILELNLENFGIALKTCMSYFTIFLSLQD
>AmelOR98
MDMFQKTGKEYSNIFDIPYYKVLKKYLQFLGQDPYQECKYRNIITIIMLISMIAIFIPTTFEI
YVSIHDKNTDAVMECLPNLCASLSSVVKILNVHFNRENFNKLLEFVVKEWDELKLNELHI
LEEITIQGSKIAHLYRNTLLSFLILFLLVPMYFPILDMIDALNQTRSRQQLLRVNYMVFNAD
DYFFYVYLQLAWGAIVIVMIVITVDSLYIIIIHHVCGLFAVCSYEIQKTVKDLTVFTDIEKCS
YKELKNCVIKHKKAIKFYNILNNSSQLSYLLQIGINIMGISTTAFQLAVNLDTRPQEAIRNAV
FCGANQFHLFVLSLPGQILLDHCAELSNTIYCSMWYKLPVKIQKMFNIMLMRSKKSCALT
VYGLYELNMENFGTTFKACISYFTMMLSLK
>AmelOR99
MDIFQKIEKQHNEIYDIPYYKMMEKYIRFLGQDPRQKDEFRNIIVFILIISIASIVIPTTLELYI
SLRNKDVDGVIECIPHFIASSISAVKLLNLHFNRQNYNILFHFVTKKWQQLKSTYELNALD
ETIMQGKKMAQLYRNTLFSFLILFLVVPLVSPILDIVHPLNQTRSRQQLLRVNYIVFDIDDYF
FYVYLQLAWGSIIVVLTIIAADWFYILIIHFNSGLFAVCGVQVLEATMNSNLISKDAFSENSS
YEKFRTCVIMHNEVIEFYNILNENCQYSYLIQVGLNMLGMSTTAVQTVINLDRPDVAIRSA
VFFGADQFHLFLLSLPGQILLDHCADFANAIYDSTWYGTSLEIQKMLYMMQIRSKKLCALT
AGGLYDMNIENFGITFKTCMSYFTMIMSFK
>AmelOR100PSE
VHIFQIYEEQCLDIFEIPYYKSLKKWLILSGLYPPKNIIIILVAISIISVTLPLMFAIYTSLHAKNI
DAMFECLPSLGVCIVAMFKLQNIYNNSENFKKLFTFVAKQWYQLKLNNEIRILEEIIMQGN

KMAQIYKNTLLLSMTIFFFVPLIFPILDIVYPLNETRPRQQLYRVNYFIFNHEDYFFYVYFQL
VWSSFVCVIVIIIFDWLYILIIHHNSGMFAVCGYQIQKIFAEKVFSNIHIYEQFKNCLIVHSEAI
QFFSILDESSRNTYLFLVGTNIMATSISAVQVVLNLDKLEVAIKSAVFLIAAQFHLFILSIPGQI
LLNHYSNLKNNIFMSSWYNMPIEVQKMFYVMQIRCKKPCSLTACGLYEMNMENFGTALK
TCMSYITMILSLK
>AmelOR101
MDIFQKTKKQHNEIYDIPYYKMMEKYIRFLGQDPRQKNEFRNIIVFILVISIASILIPTTLELY
ISLRNKDMDGVIECIPHFIASSISAVKLLNLHFNRQNYNILFHFVIKKWQQLKSIYELNALD
ETIMQGKRMAKLYRNTLFSFLILFLLVPLVSPILDIVHPLNQTRSRQQLLRVNYIIFDTDDYF
FYIYLQLAWGSIIVVLTIIAADWFYILIIHFNSGLFAVCGVQVLEATMNSNLVSKDAFSENSS
YEKFRTCVIMHNEVIEFYNILNENCQYSYLIQVGLNMLGMSTTAVQTVINLDRPDVAIRSA
VFFGANQFHLFLLSLPGQILLDHCADFANAIYDTTWYGTSLEIQKMLYMMQIRSKKLCALT
AGGLYDMNIENFGITFKTCMSYFTMIMSLK
>AmelOR102
MNIFQKTRRQCPDIFDIPYYKMVEKYFQLLGQDPRLKNEFRNFIVTVVVISISGNIVPTSIEL
YTSLCDKNMDAVIEGLPHFIAATISAVKILNVYFYRENFDKLFQFVTNEWNKLKLNNELHI
LDKTIIRGNRTAHLYRSALLIALVLFLLIPLISPMLDVFLPLNETRPRQQLLKVNYLVFNDDD
YFFYVYLQLAWGSIIVVVTSVAVDSLLILIIHHCSGLFTVCGYQVQKVISNAKSFNGTVLNN
YTYEQIKNCVIMHDEAIQFYNILNESNRNSYLIQVGLNMLAISATAVQAVVNLDRPEEAIRS
AVFCGANQFHLFVLSLPGQVLLDHCSEFSNNIYSCIWYRAPVRIQKVLYIMQIRSKKLCTLS
AGGLYEMNIENFGITFKTCMSYFTMIMSLK
>AmelOR103
MDIFQKTERQYPEIFDIPYYKMVEKYFQLLGQDPRLKNEFRNFIVTVVVISISGNIVPTSIEL
YTSLCDKNMDAVIEGLPHFIAATISAVKILNIYFYRENFDKLFQFVASEWDKLKLNNELHIL
DNTIIQGNKMAQLYRSALLTALILFLLIPLLSPILDIVLPLNETRPRQQLLKVNYLFFNDDNY
FFYVYLQLAWGSIMVVVTIVAVDSLLILIIHHCSGLFTVCGYQVQQVTGNAKSLNKIVSNN
YTYEQIRNCVITHDEAIQFYNILNESNRNSYLIQVGLNMLAISATAVQAVVNLDRPEEAIRS
AVFCGANQFHLFVLSLPGQVLLDHCSEFSNNIYNCIWYRVPVRIQKVLYVMQIRSKKLCTL
SAGGLYEMNIENFGTTFKTCMSYFTMIMSLK

>AmelOR104
MDVFQKTRNKCINIFDIPYYKLLEKYMKFLGQDPRQRDGFRNIIVIVMVASISGILIPTSLEL
YTSLRDKNMDAVIECLPHLIAAATSVVKLLNIHFNRENFKKLFEFITKEWEKFELNNQFHV
LEEITIKGSKMAQLYRNTLLSFMVLFLLVPLIFPFLDIVHPLNETRPRQQLFRVNYLIFNHND
YFFYIYLQLAWGSIIVVMIIVTVDSLYMIIIHHSSGMFAMCGYKVQEATKYQNLFNDRIISE
NYTYEQLKNCITIHNKALQFYNILNESSRNSYLIQVGLNMMGISVTAVQTVVNLDRPEEAI
RTAVFLGAEQFHLFVISLPGQVLLDHCTELANNIYSSTWYRIPVKIQKVLHMMQIRSKKPC
SLTAGGLYEMNMENFGITFKTCMSYFTMLMSLKK
>AmelOR105
MSMLQKSNEQEYNAFDIAYYKTLKLYLTICGINPYQNNSISIIIIIMIISVCMSFLCPTSIQLWE
AISNKDFDNIIQNIPQVITVIASMIKILNIYSNKMQFKNLFYSLAQDWKLLESKEELIMLDKF
TQYGSKLALLYRRTLLTFLVIFLFLPLCNPILDVILPLNETRSRQNIFNVNYIILDNYEYFYIV
YMHLSCSAVIIVIIIISVDSLYISIIYHACGLFAACGYQIQKLTKVHTIEKNGPNISNIDYEEFK
QCVIMHYKCLQLYDVLEKCCRNLYLIQMGLNIMIISVTCVEVVVFLDRPKEAIRAIIYVIAQ
QFHLYAISLPGETLLNQSSKLADKIYDSEWYKIPMKVQKVLHIMQIRSNKPCILTAAGLYE
MKIESFGITIKTCMSYFMMFLSLRE
>AmelOR106
MDVRLEERYLKINKIYSIIVGMWPNQKRKTIPRIFVELIAILAHLTQGGNMVLFFSLTLAMD
QIPFLIAAILLMIKYNNFIINEQKFKELFVSILNDWQKKKTHEEEMILEKYADKSLFFILIYV
VNAYFCTVLFLILPLTPILLDIFIPLNESRPRVQMYPAYYYIENEADYYYPILIFSIVSLLTAMC
VYIATDTTLVYVVQHACGLLTLAGYRFRNSLNDLYSMRKDSKMDEKIYRRMCYAIKTHK
RALAYLTKIEDFYSMNIFAQVGASILCLTVTLMKIATIKWSMETNQYYGFVIAQVVHIFFLT
AQGQFVIDSHDNVYRDMYEPYWYNVQYKIQAMFVLILRRNLNPPLLTAGGLMQLNLNTF
AQVVKTSVSYFTVLKSV
>AmelOR107
MDQQAMEELYLKDNKFFGQLVGVWPDQGKFMKFLMRFIILIVMIIAFIAQISRVAVFYSVD
VLSDQIPYIDLGFALMLKQYNYILNEKKLRELLHNIISDRLVKRSKEEEEIFEIYFKRAMFFC
SFYEVSIYSCGFMFLSMPSIPLIMNVIMPLNESRSRELVYPSYYFVDEQKYYYLITGHMLAV
CLGHVFVYIACDINLIHVVHHGCALLTISGYHFKHAMDNVDLCNEKYSDELMEKTYAKV

SQSIDAHKKAVEYVNKIDACHIHYFFILLGMIIVTFTGTFIKLTSMEIGGRFFTFCTFTIGQLT
HLLFLMVMGQFLIDSNEEVFKTIYDARWYYGSSKTQSLYLLVLRKCLNPPKLTGGGLIALN
LDSFVKVLKTSFSYYTVFRSS
>AmelOR108
MNKQAIEDQYLRINKFFGQLVGVWPYQERFTKFCIRLTIFAIIILTLTTQIYQVIVFCTLDALS
NQLPYLNALFILLFKQYNYILNEDKLRDLLNDIIFDRLMVRSKKELEILNMYSRRATTLCIF
YEVIVIFSAIMFIMIPTIPPILNIIMPLNESRDREFIYPTYFFIDEEKYYYPILTYMATVILIVSSV
YLACDTNLVQIVHHGCALLAISGYHFKHAVDDMKFSNGNYIDLLMDETYKKVKQSIKAH
KTAVEYVDKIDACHIYYFLLIIGMIVLAFTGTFLKLSTMEIEIRFFTFCGYTVAQLTHLFFLTI
MGQFLINANDEIFNTIYEAHWYNGSSRTQSLYVLVLRKCLNPPTLTGGGLIVLNLDSFVQIL
KLSFSYYTVFRS
>AmelOR109
MDERAIEDQYLKINKFFGQLVGVWPYQKKFFKTCIRFITFTIMIFSLATQISRVIVFYSLDVL
SDQLPYINAGIVTLFKQYNYILNEDKLRELLHDIVSDRLIERSKEELEILEMYSRRTTALCAL
YKVMVYSCAFMFLVIPTIPPILNIVAPLNVSRSREFIYPTYYFVDEQKYYYPILTHMIAVILVL
SSVYLACDTNLVQIVHHGCALLAISGYHFKHAVDDVKFCDGYYIDASMDETYVKIRQSIK
AHKTAVQYVDKIDACHIHYFLLVIGMIVLAFTGTFLKLSTMEVGIRFFTFCAYTIAQLIHLFF
LTIMGQFLINANEETFKTIYEADWYNGSSKMQSLYVLVLRKCLSPPKLTGGGFVALNLDSF
VQILKASFSYYTVFRS
>AmelOR110F
MDKQTIENQYLKINKFFGQLVGVWPFQERFIKTCMRFIVSVIMLLDLATQISRVIVFYSFDV
FSDQIPYLNAAIICLFKEYNYVLNENKLRELLNDIISDRLIRRSKKELEILELYSRKATTLCIL
YKVMVYSCAFMFLVIPTIPPILNIVAPLNVSRSREFIYPTYYFVDEQKYYYPILMHMIAAILV
LSSIYLACDTNLVQVVHHGCALLAISGYHFKHAVDDVKICDENYITLMDETYTRVRHSIKA
HRTAVEYVDKIDACHIYYFLLTIGMIVLTFTGTFVKLSTMEMGIRFFTFCAYIAAQLTHLFFL
TIMGQFLINANEEIFRTIYEARWYNGSSKTQSLYVLVLRKCLTFPKLTGGGLIILNLNSFVQI
LKASFSYYTVFRS
>AmelOR111
MDKRVIEDQYLKINKFFGQLVGVWPYQQRFIKFCIRFITSVIVVLTLAAQISRVIIFYSIDVLS

DQLPYLDVGFVLLFKQYNYILNEDKLRELLNEIISDRLIKRSKEELEILEIYLKRARVLSTVY
EVSIFFCGFMFLLIPSIPPILNIISPLNESRGRELIYPSYYFVDEEKYYYPILMHMIAVALILTSV
YVACDTYLVYIVHHGCALLAISGYRFKHAVDDIKLCGGDCIDPLTDETYTKVRQSIKAHK
MAVEYVDKIDACHIHYFLLIIGMIVLAFTGTFVKLSSMEVNVRFFTFCAFTVGQLTHLFFLT
IMGQFLINANEEIFKTIYEARWYNGSSRTQSLYILVLRKCLSPPKLTGGGLVALNLDSFLQIL
KASFSYYTVFRS
>AmelOR112
MDARTVEKNFLKVNKIFGLITGVWPYQNYRSKMAERFISVTVMMSGFVTQFAYLVLNPT
MDKIATNLPYSIASFGTFVKMGNYFLDETKLTTILNHIFEDWATIKSKEEYEIMYKYSRRGL
FITISYFLHIGVTETFMLILPMVPPILDIIVPLNVSRKRVFLYPAYFWLDDEKYYVLLLGHMII
TLLMICFIFCACDTNYVYAVQHACGLLAIAKYRFKNVCKNLKEDHAIPLEEIKYKSICESIK
AHQHALKYLKLIENSYHTYLFVSMGLLIMAISVSLLEVANGKNGSRELVQATFLFAQLFHT
FILTVQGQFVINELQDVYESIYESPWYTFSPRIRSLYVLSLRSCLNFPTLTAGGLIVLNLQSFA
EIIKAAVSYYTVMQTT
>AmelOR113
MDSDILEKRFLKITKRFAKLSGIWPDQNKYLKYISWIIIYVISIPSIVVQIARIVHISTANVIVE
QSGIATAIFLSLLKEANYILNATKVKSLFNDMYMDWRMDRPKKEFEIMSTYAQRGSFLAM
FYFINAYCCSLLFLQVPWTARLLYMIKSQNTSPPMLYVIPGYYFVDDDRDYYYFIQLHMSL
SIIMVANVYVAYDTCYMVFVQHVCGLLAVAGYRFKHAIDDSASKNSEEKIKETCKKIRSSI
QGHEGAIRYLKKIEDTHVNLLFISLGLIIMCFSITLLKVVTMDYCLDFYKYSSFLIVQLMHL
CYVMIQGQFVIDSCNEIYYSIYEASWYNINPKIQALYILALRRSLTPPRLTAGGLIELNMQSF
SEVIKLSISYYTVLRST
>AmelOR114
MKFIGIWPEERKWNQASNYLVLIPFLMILCFICAPQTINLTIISNDFNLVIENLSMGNITITLS
LLKTIAFWINGKPLKSLLNCMANDWIKVTSKTEQETMARIASITRNTIIKSTVMCHTVVAF
YVFLRYISMKYNENKLLFRAYFPYDTTVSPNYELTILGQFVAALYAATSYTAVDTFVAMLIL
HVCGQLSGIKNELSRLPTYDKKDLKRRLKEIVQKHEYVNRFAETIENCFNVMLLIQILGCT
VQLCFQCFQAIMSFGGGEAQEYLFFQLMFLLVYVFYVMLQLYLYCYVGERLSVESMEIVN
AAYNTEWYTLPTNITKMLIIVMCRAKSPLTVTAGRFCSFTLQLFSEVLKTSMRYLSVLYAV

KDKIKR
>AmelOR115
MDFAMGWNRFNLTLLGVYPEPRKMSRNSRLMSSLIFWFTTLVTFTFICAPQTANLILKSTS
LDEVIENLSINIPIVFALIKQIVLRYYKKALTELLGEMLADWSGPIGDQDRETMLRNARLSR
AISIVCSTLTYFMLLAFVSLQVWSNAENASETDLGGLLHPATFPYETSKSPNYEITWLGQL
MGTVLTAICYSCFDTFLAVLVLHLCGQLTVLGTALEDLVNATRRNDYKTFEQRLSSIVNRH
NHLSRFAVIVEDCFNITLLVQTLICTAMFCLTGYRMITSVDREDEADVPIVGIIFFIIHVIYTM
LHLFIYCYVGETLLGQSTGIGLSTYHCNWYDLPSRRAVLLMIVIRRANVSFQITAGKFSPFS
LEFFNAVLKTSAGYLSVLLAMKDRLVEGK
>AmelOR116
MTNHLEKQIKLKKINSNKHLQNNLSIIYYIGLWPDRVKYKYLYNLYTICSLIFLVGIIIVSEII
YIIINWGKIEIMMTGLTILMTNSTYAAKVIYIICRYERIKNLVDITNSEIFNRDNDKYKHIISY
YNWQGIFHHIAYQGFASICIFSYSCIPLQSAFSGKSKQLPIAGWYPYNVTSTPIFEIACLHQV
LVILINCINNIAIDTLITGFIIITCCQLTILKKCIARNNNINIEKSPSKIYNKFYENLKHCVKHSII
IFDFTKQIQDIFGIIIFFQLFVNCIIVCLAAFNLSQIKNYITPEFFGSLLYICCMIYQIFIYCWHG
NELYLHSMKICLSAYKNNWWNNNKNFNYALLIIMIRTQIPLIIIVGKVMELSLQNFLLILRTS
YSIFTLLKTFTT
>AmelOR117
MKKPFNKSIDYYILPNKIFCSIAGMWPIDEKSSIFSKIFAYVRLIFGLIIVNSFFIPQIIIIVMNW
KNIKIIAGIGCVLTTITQVLFKMIYLIARREKTYSLYYKIRNLWNSSNDSKERPYEEFAYWAR
IFSIIFYSSCMCNVFTFSIAAAIDYFKFEYNANNTENNRHLPFIVWYGTDISASPSFEIVFFYQ
IISSSICASVISGLDTSLMTIILHVSGQFKLINIWINNIGIEINCNPNYMRKLKVDLIKCIRHHQ
QLIHVVNNVNNLFTPIIFIQLLTSGIEICLSGYAVLDNNSANADLLKFISYFISMGIQLLLWC
WPGEILIQESQEIGHVIYLNIPWYNLPPIYQKYLYFMIVRSQQYCRITALTFQTLSICTLSNVF
NTSVSYFTLLRQMQQ
>AmelOR118
MINRPLEYSLRIFGIWPDSPYPKLKIITWIIILPTFLVFQYWYCITHIKLGLIDLLDGLSLTLSN
TLVFIKLIVIWFHKRTFYEILMSMKEDLNNNKHSATENKRIIMDKSMLSSRISNFLISYFAITF
FLYSGVALVIFDEDQGKFLVRMEFPFIATISPRYEIILITQFIFESFIVYGAATSIALIAALILYVG

SQIDLFCQNLTFHSYKKRESQDTIKDIIVRHQKIIQLSKNIETIFTYISLCQFVSNMLVICFISF
VLTVSLHTEQTIVLIMKCLPYYIAVNCEAFILCYTGEYITSKSENINKAVYNFLWYNLKPRD
VRIMLMIILRSQKQLTLTAGKFICLSLEAFANMLKASASYVSVLYARY
>AmelOR119
MHTQRDTSEITYSHDTATSRKLFYLLVVVGGQMAHASGHEWMKSIRTLTSIKINYLKYSG
LGEIDSSCSRILKYAYFVYKVWMLVSMCILAITVFADIYTNMDNLSITTDDGCIFAGIFVVIF
KAMNLQIQLESVKKIIDKYHTRNKVMFFGFCVIGACLGFALLCFTPMENGLPIRAKYPLNT
TVSPWHEISFFVETCAVSGGLLGIIVMDSMTTFKCSLITMLLDALSVNFENCNGETKRTICN
RHGKEERNDNNNRFLDRYKKCVQFHQRLVVISRDYNKIYSLSMLVQMISSTSIICLTGFQA
VVVGGQSSNIMKYGIYLSAAMSQLFYICWLGNELGYASSTLDKNQWFSGWCNERLTGIG
QVFTLSTVFTRKSIILRASVFYVLSLETFIAIIKRSYSFFTLLNNMDLTDH
>AmelOR120
MSNQTNTMNIRNYIFINQLVLKFVGFYPINILRYVICISCIMFIVIPQIIMIYINWNDLNIVME
TGSTLLTILLAALKSIVWIFNRKKLEFFIEFMLTDYWKIIETNVFEYLQEYAIYAKNITKGYF
FSMCNALLFFFSLPIIETLTKNENLNNFTIKNFPFAASYPITFYKFPFYEIAYISQILATSICCLM
MLAIDSLIATALLHTCGHFTVLKENLKNLDTYIYDLTKTNLKTNSKYINKNLYEIKTQIIYII
KHHQLVLWFCDNMEKNFHLILFLQAITSSLIICFVGFQISIALTERSKFLESFSHLIVSLFQLL
LFCFPGDILIRQSFNISIAAYSMQWYQLPTFIKDEICMIILRSQRPSFITAGKLYIMHLENFTAI
LSTAFSYFMMLQSFNTEA
>AmelOR121
MHTSESKKYSKDYEWAVRLNRFSLNVICLWPVEEQNMRKQSWTKLHIMTCFMLITFVCTI
PCLCALKQCNNLMEVTDNLAYSIPLIITTIKFIVVSSKKKVLSLIVNMVAKDWAKLKTDHE
KDIMIRRARIARIINIFGYILICILIWLLMILPRFGITIRYVTNETDAKKLFPLPSYYIFDVSETP
YFEIMYALQSISLLIAAFCYAGVDNFFGILILHICGQLTNLRFQLANIKESEASNFILIAIVKD
HIRLIRVIELLKMFVEQIINLIITIFKYFFKCLKMYIEEEQFSLFRIIYLICNFTNTFLQTFLYFM
AGQMLVTQSEEVHNAAYECEWVSLKYTKAKSLIIIMARSKKPLYLTAGKLFPVTMLTFCNI
LKISLSYISFLLTIL
>AmelOR122
MNMDVFDKQYRIYRIILKIVGLWPYDKSIYVWIQRICLSMYFLIGVIFQIILLVKSEITLRNYI

VTLSAIFPLLLFFIRYIYYITMFPYVEILFDNIRTEENLLQDTTEIQIQTKYLDISSHIIYIFCCM
TFAFIVAAIIFLVNPVILDLRNPLNESRIFYFDLLFLLDDQSAYIKIFLILNFMLNILFGLLSITS
TESFTNIFSYYICRQFNIVNYRIRKIIEDLSTRNLSKIDLKIKDIHRVVDIHCHAIELLYKALIT
MDNRIEIFGSTLIVIYHLMIAFYNNHCGQLIIDSNLGIFNELFASTWYRIPLKAQKLLLFMIL
RSSMGCEICLSGLFTPSYAGLTSMMSSSFSYCTVIYSIQ
>AmelOR123
MNVFDNQYRTYRIILKIVGLWPYDNSIYVRIQRICVLIYFLIGILIQIFSLVKSEISLRNCIVTF
STTFPIVLFCLRYIYCLTLFSYAKVLFDDICIEEHLLQDTTEIQIQTKYLDISSHIIYIFCWLSFI
CVASTWIFILNPVILDVIMPLNKFRLHYSVIFLSNDRRKFIDIFLVLNSIIIFTFGLLSLICSELF
TNIVSYYICRQFHIVCYRIRKIITDLSMPNLPKTDLKLRDIHRVVDIHCHAIELLYKALVTTD
NRMEILGSTLIVIYHVMMALYNNHYGQLIINSNHGIFNELCASTWYRIPLKAQKLLLFMIL
RSSMGCEICLSGLFTPSYAGLTSMMSSSFSYCAVIYSIQ
>AmelOR124
MNVFDNQYRTYRIILKIVGLWPYDNSIYVRIQRICVLIYFLIGVLVQIFSFVKSEISLRNCIVT
FSTTFPTLLFCLRYIYCLTLFSYAKLLFDDICTEEHLLQDTTEIQIQTKYLDISSHIIYIFC
>AmelOR125
MNMDVFDKQYRIYRIILKIVGLWPYDKSIYVWIQRICLSMYFLIGVIFQIIVLVKSEITLRNY
IVTLSAIFPLLLFFIRYIYYITMFPYAKLLFDDIRTEEYLLEDETEIQIQTRYLDISSHIIYIFCCM
TFAFIAAAIIFLVNLIILDLRNSLNEFRFYFDLLFFFDDQSAYIKIFLILNFMLNTLFGLLSITST
ESLTNIFSYYVCRQFNIVNYRIRKIIEDLSTPNLSKIDLKIKDIHRVVDIHCHAIELLYKALTA
MDDRMEILGSTLIVIYHLMIAFYNNHCGQLIIDSNLGIFNELFASTWYRIPLKAQKLLLFMI
LRSSMDCELRLSGLFTPSYAGLTSMMSSSFSYCTVIYSIQ
>AmelOR126
MNVFDNQYRTYRIILKIVGLWPYDNSIYVRIQRICVLIYFLIGVLVQIFSFVKSEISLRNCIVT
FSMTFPTVLFCLRYIYCLTLFSYAKLLFDDICTEEHLLQDTTEIQIQTKYLDISSHIIYIFCWLS
FICAAASCILILNPVILDVIMPLNKFRLHYSLIFLSNDRRKCIDIFLVLNSIIIFTFGLLSLICSEL
LTNIFSYYICRQFHIVCYRIRKIITDLSTPNLPKTDLKLRDIHRVVDIHCHAIELLYKALITMD
NRMEILGSILIVIYHLMMALYNNHYGQLIINSNHGIFNELCASTWYRIPLKAQKLLLFMILR
SSMGCEICLSGLFTPSYAGLTSMMSSSFSYCAVIYSIQ

>AmelOR127
MNVFDNQYRTYRIILKIVGLWPYDNSIYVRIQRICVLIYFLIGILIQIFSFVKSEISLRNCIVTF
STTFPTLLFCLRYIYCLTLFSYAKLLFDDICTEEHLLQDTTEIQIQTKYLDISSHIIYIFCWLSFI
CAAASCIFIVNPVILDVIMPLNKFRLHYSVIFLSNDRRKCIDIFLVLNSIIIFIFGLLSLICSELF
TNIVSYYICRQFHIVSYRIRKIITNLSMSNLPQIDLKLRDIHRVVDIHCHAIELLYNALITMDN
RVEIFCSTIVVTYHLMTALYNNHYGQLIINSNHDIFNELCASTWYRIPLKAQKLLLFMILRS
SMGCEICLSGLFTPSYAGLTSMMSSSFSYCAVIYSIQ
>AmelOR128
MNVFDNQYRTYRTVLKIVGLWPYDNSIYVRIQRICVLIYFLIVVLVQIFSLVKSEISLRNCIV
TFSTTFPTLLFCLRYIYCLTLFSYAELLFDNVHTEEHLLEDTTEIQIQTKYLDISSHIIDIFCW
MSFICVASTCIFMLNPVILDVIMPLNKFRLHFSLIFLSNDRRTYIDIFMVLNLIIILIFGLLSIVC
SESLTNIFSYYIYRQFDIVSYRIQKIIADLSMPNLPKTDLKFRDIHRVVDIHCHAIELLYKALI
TMDNRMEIFGCILVVAYHLMIAFYSNYCGQLIIDSNLGIFNELYASTWYRIPLKAQKLLLLM
MLRSTVGCELHLSGLFTPSYAGFTSMMSSSFSYCAVIYSIQ
>AmelOR129F
MNVFDNQYRTYRIILKIIGLWPYDNSIYVWIYRLCLLIYFLVVVLVQIFSLAKSEISLRNCIV
TLSTTFPTLLYCLRYIYCLTLFSYTELLFDNIRTEEHILQDMTEIQIQTKYLDISSHIIDIFCWL
SFICVAATWIFILNPVTLDVIMPLNKSRIHFSLIFLSNDRRTYIDIFMVLNLIIILIFGLLSLICSE
SLTNIFSYYVCRQFDIVSYRMRKIIVNLSMPNLPKTDLKLRDIHRVVDIHCRTIELLYNALII
MNNRMEIFGSALMVMYHLMIAFYNNHCGQLIIDSNFGIFKELYASTWYRIPLKAQKLLLF
MMFKSSVGCELRLCGLFTASYAGFTSMMSSSFSYCAVIYSIQ
>AmelOR130
MNVFDNQYRIYRIILKIIGLWPYDNSIYVWIQRLCLLSYFFANIIFQIVSLLRSEITLQNSILIL
SITCPLVLFLLRYIGSIACFPTIKIVFKHIRTEENIVQDSIESQIRMKLIDDSHHIINIFFWMTYT
TIVIFIIYVSYPIILDFMIPLNESRTHFIYYITTFSHNQSIYLDILDFNFMFTGIFGLLSVACSESI
TGIYSYYICILLKIVSYRIQKIIMYLAMFKLSPKQIDSKLIELYRVVDIHNQTIELLVNATLIKK
NQLEMLFCFTLVAIHLVIIFLNNYNGQIVMNSSQELFDELYNSMWYFMPLKAQKILLLIML
QSTTKHAFNILGLFTPCYAGFSTMLSSSFSYFTLMYSIQ
>AmelOR131

MDVFDKYYHTYRIVLKIIGLWPYNNSVYVWIQRLCISALFLGNIIFQILSLIRSEITLRNCILI
LSTTCPLIIILLRYISFIIFFPMVKLLFHHICVEENAVQDLIEIQIRMKYIGNSRHMIEILLRVTF
LTITLFSIFLLYFVTMDFIMPLNEFHRHILLYVTLFSVNRTIYFYILYLNFLFVITFGLLSLICTE
SIVGLYSYHTGMLFKIISYRIRKIITYLTMFNVSSKQIDSKLAELHRVVDIHNQAIGLVVNAI
TIKKDQLEILITLIIFANHLMIMFLCNYNGQILINSNEEFFHELYIPVWYFVPLKVQKILLLIMI
RSSMACIFHIFGVFIPCYVGFTTMLSTSFSYFTLIYSIQ
>AmelOR132
MDVFDKYYHSYRTVLKIIGLWPYNNSIYVWIQRLLLLTLFLGNIIFQIMSLLRSEITLRNCILI
LSTTCPLIIISLRYICFILFFPMIKYLFHHMRMEENIVQDSIETRIRTKCINDSCHMIDIFLWMI
YAIFAFCIILLLCPIILDFIMPLNESRIYIAHYITIFSDKRIIYVDILCLNYMFLMIFVVLSIMSTE
SILGLYSYHTSMLFKIISYRIQKIITYLTIVNLSSKQIDSKLAELYHVVDIHNQAIQLLENAIIV
TKDHLEILICLMLFVKQLMIMFLCNYNGQILIDNSEELFDELYFSIWYFVPLKVQKILLLIM
TRSSTTCMFHILGVFVPCYTGFTTMLSTSFSYFTLMYSIQ
>AmelOR133N
MEENIIQDSIEAQIRTKYISDSRHMIEILLWMAYATITLYSILGLCPIIFIILLNESPIRMLHYVT
LLSVNGTIYFYILCLDFLFIIIFGLLSMICTETIVGIYIYHTSILFKIISHRIQKIIAYLNMFNLLS
NQIESKLAELYCVVDIHNQAIQLLVNAITIKKDQLEILISLIIFVNHLVIMFLCNHTAQILINN
NEEFFHELYISVWYSVPLKVQKILLLIMIRSSMACIFHICGVFVPCHAGFTTMLSTSFSYFTL
MYSIQ
>AmelOR134
MDIFDKHYYSYRTVLKIIGLWPYNNSIYVWIQRLWISALFLGNIIFQIVLLLRSKITVRNCILI
LSTTCPLIIISLRYICFILFFPMIKYLFHHMRMEENIIQDSIEAQIRMKYIGDSRHMIEIFLWMA
YANITLYSILGLYLIIFIMPLNESPIRMLHYVTLFSVNGTIYFYILCLDFLFVIIFGFLSIICTETII
GIYIYHTGVLFKIISHRIQKIITYLTIIDLSSKQIDSKLAELYRVVDIHNQAIQLLVNAITIKKD
QLEILISLIIFTNQLVFIFLCNHTAQILINNSEEFFYELYISVWYFVPLKIQKILLLIMIRSSTAC
MFHIFGVFVSCHAGFTTMLSTSFSYFTLMYSIQ
>AmelOR135
MDVFDKQYHSYRTVMKIVGLWPYNNSIYIWIQRLLLLTFFLGNVIFQIVSLLKSEITLRNCI
LILSITCPFIIVSLRYVCFIVFFPTIKLLFHHMRVEENIVQDLIEIQIRTKYINDSCHIIDIFFWVA

CTNITLSSISLLYFITLNFIMPLNEFRIIHYITLFSVNRTMYFNILCLDFIFVVIFALLSVICTESII
GLYSYHISVLFKIINHRIQKIITYLTIVNLSSKQIETKLAELYRVVDMHNQAIELLVNAIIIKKD
QLEISISFIFFVNQLIIMFLCNHSGQILIDNSQKLFNELYISIWYFVPLKVQKILLLIMIRSSTRC
MFHILDIFTPCYAGFSKMLSTSFSYFTLIYSMQ
>AmelOR136
MNVFDKHYHTYRTLMKIVGLWPYNNSIYVWIQRLWFLMFFFGNIIFQIMSLLTSAITLQNC
VLIFSTTCPLIIVLFRYIGLILFFPTIKLLFHHMCMEEAMIQDSIEAQIRRKYIDDSCYMIDIFF
WMTYVGIALCSILLLCPITLDFIMPLNESRTRIVHYVTIFSDKSIIYMDILCLNYMLLAILVIL
SATCTESILGLYSYHTSIMFKIIGHRIQKIVKYLTMFNLSSKQIDSKLAELYRIVDIHNQAIEL
LLNAIVIKKDELEILISFIFFTTQLVITFLNNNCNQILIDNSQELFIELYISMWYFVPLKVQKIL
LLIMIRSSTACMINILGVFTPCYIGFSKMLSTSFSYFTLMHSIQ
>AmelOR137C
MDIFDKRYCTYRTMLKIVGLWPYNNSIYVWIQRLWLLIFFLGNIIFQVVSLSSEITLRNCILI
LSLIFPLTIILVRYVSCVIFFSMIKLLFHHMRMGGNIIQDSTEIKIRKKYINDSCHMMNIFFWII
YGIAALSIIFILYPMTLDFIMPLNRTRIRIIHYITIFPYNRTMYLDILSLNFMFVGIFGSLSLACT
ESIFGLYCFHASILFKIIIYRIQKIVTYLTMFNLSSKQIDTKLTELYRAVDIHNQTIGL
>AmelOR138N
VVSLSSEITLRNCILILSLIFPLTIILVRYISCIIFFSMIKLLFHHMRMERNMIQDSTEIKIRKKYI
NDSCHMINIFFZIIYGIAVLSIIFILYPMTLDFIMPLNKTRIHIIHYITIFPYNRTIYLDILSLNFM
FVGIFGSLSLACTESIFGLYCFHANILFKIISYRIQKIVTYLTMFNLSSKQIDMKLTELYRAVDI
HNQAIGLLVNAIIVKKDQLEMLISFMILMAQLIITFLCNYNNQILIDNSQELLDELYISAWYF
VPLKVQKILLLIMIRSSTSCTFHILGVFIPCYTGFSKILSTSFSYFTMIYSIQ
>AmelOR139P
MDIFDKHQSYHSYRTIMKIIGLWPYNNSIYVYIQKLZLLIFFLGQIIFQXDAVIAFFSISLLYPI
ILDFINSLNESRTRIIHYFTIFFHSRIIYIDILCLNYIFLAIISLLSIICIESMIGLYTIVTTSLFFKIIG
YRIQKIITXLTIFNLSSKQINSKLVELYHVVDFHNQVIELLVNMILIRKDQLEIFMFFIFLVSQ
MMIMFICNYSSQILIDNSQELLYDLYISMWYFVPLKVZKILLLIMIQSSITYMISILGVFILCHI
GFSTMLNTSFSYFTLIXSTQ
>AmelOR140N

LRGLYSVWGVNYDAVIECMPPIISIFQSASMYFNGIFNTKKIKNILLFIKNDHKYYINRPENII
LQKYDLQGKKITFYYILYVYTTLFVYLLLPTIPLIIDFITSSNHSQKRNFLFELDYGMDKQQ
YFYYISIHSYIGTAIVANLIASCDTMYMLYAQHAYALFAIVSYELKTIHILNTNNLINVTDHH
LLEKYKNITLLSKDEKKVYRKLFICIKNHQNAIKYSNLLESLFTKSILVQLFFNVLCLSITGV
ETVIKLGNLSEMMRFGSFTFAQAVHIFFLCLPGQRLLNHSEELHVSACEVTWYIFPKKYQN
LYKFLLARSLIFSKLTAFKVTTLSMQTFLAIIQTAMSYFTVLLSTT
>AmelOR141
MIDEKTKREFDKTIDLNLFLLKLCGIVPCGDGFARNILAWLAFSCLTIYSISYVHEFITNTTN
LTTALESVAMIISIVGGHARYTILLWFRDICQTMLNVCEIFWSNLKPHEKKIVQSYTRKTTR
LTRWYLASCVLTIAFYAFLVLFGSLFDQSKDFEHMRNDSSLVPSEAGNILESMSKRHLPYAF
FLDVQKTPWYEIVYAVQLIGMFNVGFTCVGVDTVGALFILIICGYFDTIQSRIENLHSFDTS
LSSSLLNILSRKITTAKMSDIKTEASNSVQMRNLRMCVIHHQLLLNRFCEDIEHLTSGMFFI
QVIASTYNISLVGFKLLEDTPDKFKYITQLIILIIQLFLCNWPADLLLSKSIDISRATYSMPWY
GYSYNLQKITNILMIRSQKAVRLTAGKFIGLSLETFASMISTAASFFTMVRSMN
>AmelOR142
MKNRLTPEKAILFTKLSVALTCSWPPSPLATKAQHLFFNALWCIAFLTSVMLFLPLLAAIYV
YRKHPVILGKTVSLTAAVAQVTIKMIICRLQQKRFQMLYSEMENFCKQATNEEKIILQRYV
DRYKYFHSFYILWSFLTTIFVICGPLYTVQTFPTHAIYPFSVRRHLYKGLIFFHQSLVGFQVSS
GMAIDTQIALLLRYATARFEILGIQFNNAKSDGEFDACIKKHDELLRYSREIRQSIKFLILAT
NGTTVIAVIFGSLNLIANQPLILKALYAIVVFSASVELFMYAWPADSLMHMTMKMATKVY
NMDWYGKDIRTQRKILFIILRSQKYESFGINGIVPALSLSYYGKYLYTSLSYFNALRIMVED
TVN
>AmelOR143
MNIRQILYILELIGTFTCTWPINPNISKRRIIFRNIFWIFSILNVILLMTSLMLAVVYFRNDILM
SLKTASEMAALLEVVLDLILCKWNNSEFQVLIEEVKSFVEMANEYEIKILQGYVNRYKKF
FSTVSMGYISTAISFSLMPLFSAQKLPADGWLPFSTEPFGIYCIIYFNHVYCILQTAFCIFVDF
TIVILFSFPAAKLDVLRSKLRHVNNYDTLVSCIKEHQKIIGFVEDTKATVETLLFKTNVTMG
STVMCGAFPLLNNQSLAAISQFLPLVLSGILHLYVIAWPADDLRESSVQFSNSISDIQWLGQ
SNKMKSCVIFMMMRSQKAFLIRMSNLLPPLSLEYCSNFITTVSSYFMAMRTMIES

>AmelOR144
MGMLNMDIRQVLHILELTGTFTCTWPINPKDSKKYIIIRNILWTFTILNVIFLTISLMLAIFHF
RSNIPKSMKTASEMAALLEVVLDLVLCKWNNSELQVLIEEVKSFLEMASEYEIKILQGYIN
RYKKFFSTVSMGYILPASSFILMPLFSAQELPAEGWLPFSIEPLGIYCVVYVNHIYCILQTSF
CIFVDFTIVILFSFPAAKLDVLRSKLRHVNNYDMLVSCIKEHQKILGFVEDTNATVETLLFK
TNVTMGSTVICGAFPLLNNQSLDVVTQFLPLVLSGMLHLFVISWPADDLRESSIQFAESIND
IQWLGQSKKMKSCVIFMMIRSQKLFLIRMSSLLPPLSLEYCSNFVTTVSSYFMAMRTMIES
>AmelOR145
MGMLNMNIRQVFYILELTGTFTCAWPINPNDSKTYIIIRNILWIFTILNVIFLAISMIFAIFHFR
SDIPKSMKTASEMAALLEVALDLALFKWNNSELQILIEEVKSFLEIADEYEIKILQGYINRY
KKFFSTVSMGYILPASSFILTPLLSDKELPTEGWLPFSIEPLGIYCAVYVNHVYCILQTLSFCI
FVDFTIVILFSFPAAKLDVLGSKLQNVNNYDMLVSCIKEHQKILGFVENSNATVETLIFKTN
ITMGSIVICGAFPLLNNQSLDVVTQFLPLILTGMLHLFVIAWPADDLRESSIQFAESINDIQW
LGQLKKMKSCVIFMMIRSQKLFLIRMSSLLPPLSLEYCSNFVTTISSYFMAMRTMIES
>AmelOR146
MFRNATPEKAIAFTQFIVSLSCCWPLPSTATKLQTRCFKIIRSLLFLNSLLLFFPLLYFVYVNR
NDNTTFCKAMSLSLAVVQVPLLSSFCITQYDRFQRLIKEMKFCCENANSYERQVFQGYAK
SYATFYGVSAIWFYWCALIVVVGTLFISDPFPTNAEYPFPVHFEPVRSIVFVQQALVGFQCS
AHLCVNIFCALLLLFAAARFEILMNELRAVENIESLIKCIEKYYAIRRYAEEVVNSARYTTLI
TLCICGVESVFGGIIFIGRQPFTVKLQFLTLSATTLLAVFMCAWPADYLMDVSENTMRAVY
ESEWYKRSLKLQKFVLFATIPQTPVILKVRCIIPAFSLNYYCSFITNVLSMFTALRVLMYKD
EN
>AmelOR147
MLKQVSPEKGIYIIWLSVALSLCWPLPINSTRKQIVCMKILQIGAIISAFMILLPLIYTIYLNL
DNLNIFFKSICLLMGVFQHIVQTITCFIKYDSLQRVVEEMMICIKEMQLNEIMCAYVAKCNI
FYGGTIVLIYTTATVFILGPTFLPITFPWETEYPFQVNYTSRNFIIYMHQFFFTYQCAAHICVS
MFVALLLWFTSARFECLVKELQKTTNIEMLIVCLKKQLLLRRYAEDVVNCIRFIIFYTMAVS
TIVLTLSGIILITTSSLLVKIQFLTICISILLEIYMYAWPADHMYDMSITVLQSVYDSMWYGQT
LNMQKLVLITLIYQKPVTISINVVLPTTYFALLLF

>AmelOR148
MLKYVTPEKGIYIVWLSVALSLCWPLPASSTRKQIVCIKILQIGAIISAFMVLLPLIYAIHLNI
HNLINLFKCICLLICVFQNIIQTIICFIKYDVLQRVVEEMMTCVKEEQLYKVLCIYVKKCNIF
YGGTIVLTYGAATVFVLGPTFLPISFPWETEYPFQINDTSRNIIYIHQFFFTYQCAAHICLSLF
GALLLWFAAARFECLVEELQKITNIDMLIVCFKKLLLLRRYAEEVVSCIRFLVFYAIAVGTF
MLTLSGIIMIINSPILVKIQFIIICMSSLMEIYMYAWPADHMQDASINILRSAYNSIWYEQSLD
MQKDLLIILMYQRPVILSINVLLPELTLRYYCSYVANAFSVFTALRAVVEDK
>AmelOR149CN
PARRVVEEMIICIKEAQQYERKIFCKYIENCNIFYGSSLTITYLVVIIYIMGPIVLPTPFPVDTE
YPFHVNSTIIKIIIYLQQSLLIFQCAGHLCISIFCALLLWFTAARFECLIVELQKITNIGMLIICI
KKQLRLRRYARNVVNSFRFMIVYAIGVSTFALILYGIIMIVKAPLIMKIESVTLSFVLLLQIYI
YAWPADHMKDM
>AmelOR150C
MLKQLTPEKAIHITWISVAITFCWPLPANSTKIQVFMFKTLQIISIINAFILLLPLLYSVYLHFD
DIVIVFKSIALCVGLSQMIIQTAICFVKYNTLQRVIEEMITYVKEAQQYERKIFHKYIKKCYT
FYGCSIICMYLTGLAFIIGPAFSPASFPADAEYPFQINYKSIKVIIYLQQTLVGFQCTAHICLSV
FGALLLWFTAARFECLIVELKKITNISMLIVCIKKQLHIRRYAKKVVIGFRFIILCAIGISTFAL
TLGGVIMIKKAPFIVKVQFITLILTLLTEIYIYTWPANHMKDMSINVSQSIYNITWYKQTLR
MQKDVLTVLMYQQPIILSINCILPELTLHYYCS
>AmelOR151
MLKQLTPEKVIYITWVSVALTLCWPLPANNGKIQVFMFKALQIISIINAFILLLPLLYSVYLH
FDDVIIVSKCVAVSIGLTQVITQTIICFAKYDSLQHVIEEMIICIKAAQQYEEKIFHKYIEKCY
TFYACSITCMYLTATAFIIGPAFSPASFPIDAEYPFQINYTSVKIIIYLQQTLVGFQCAAHVCLS
IFGALLLWFTAARFECLAVELQKITNIGMLIACVKKQLRIRRYAKKVVISFRFIILYAIAVSTF
VLILDGIIMIMKVSLIVKVQFITLSLTMLTEIYIYAWPADYMKDMSTNVSKSVYNITWYKQT
LRMQKDVLNILVYQQPIIFSVNCILPELSLRYYCSYLSNAFSIFTAIRVMIEDDP
>AmelOR152
MLKQIISEKTIQIIWFSVAITFCWPISLNSSKTQVFIFKILQIISIINVFMLLLPLLYSVYLHFNDI
IIVSKSIALSVGLIQVIVQTIICFIKYDSLQHVVEEMIIYVKEAQQYEKKIFHKYIEKCHIFYG

CSIACIYLTATVFVIGPVFSSASFPADAEYPFQVNSTSMKIIIYLQQSLIAFQCAGHACLSIFG
ALLLWFVSARFECLAVELQKTTDIGMLIVCVKKQLHIRRYARRVVISFRFIILCAMGVSIFSL
TLGGIIMITKSPFIVKVQFITLILTLLTEIYMYAWPADHMKDMSINVSKSVYNTIWYEQTLR
MQKNLLNILMYQQPIILSINCILPELSLRYYCSYLSNAFSIFTAIRVIIENNPS
>AmelOR153P
MVKEMIPEKTIHITWLSVALCWPLSVNSGKTQVFIFKMLQIISIVSACMLLLLSSYSIYFXH
GQCRIFKNYHRFIDVAQNIIQTVICFYIIKEMKICIKETQEYEIEIFQKYIAKFKTVWGCNITC
MYLTALAFTIGSVFISTSLSCDAEYPFQLNYTLVFAIRYQSFLSYQCAYACADHXLLWFTAP
RFECLCVELQNVTNINMLIVCXYAKKMINWFRFIIFNAIGLSILVFTLASIILIMISICMYIVV
CSCMYNFINKNYYMYIWPADYMTDKSINVSRKIYDSMXYKQMLKMQKNLLKXLIFQRP
VXIYRLZLLSKLILRYYCLYLSNVFSIFTALHVLEDNI
>AmelOR154
MLKKVTPENVIYIIRLSVAICCCWPRPFNSTKNQIFAFKVLQISTIISAFMVFLPLLYSIYLHN
DNIIHVFKCICLSIGITQLIVQTLICFIKHNSLQRVVEEMVNCVKQAQQSEIEIFYKYIEKCKI
FYGSSIAFSYLAATAFMLGPAILPISFPLEAEYPFHVNESLITIIIYMHQSLVSYQCSANVCVSI
FGALLLWFTVARFECLIEEFQKCSNIDMMIACIKKQLQLKRYAEEIINCFRYIVLYGIAVTTF
ALILCGIILLMNIPLIVKIQFVIICITIMTEVYMYAWPADYVKNMSINISRSVYELSWYEQTIE
MQKNFLNVLVYQKPVIFSISCIVPELSLRYYCSYLSNVFSIFTTLRVLLEDTSA
>AmelOR155
MLKKATPEKIIDIIRFSVAICFCWPYPLNSSRNQIFGFKVLQISTMVSACIMLLPLLYSIYLHN
DDVIHISKCICISIGVTQLIVQTLVCFIKHNSLQRVVGEMMKCVKEAQQNEIEIFSKYIEKCK
IFYGSSIIFSYLTSTAFMLGPIILPISFPFDAEYPFHVNHSLVTIIIYIHQSLVGYQCSANVCASV
FGALLLWFTVARFECLIVEFQKCTDIDMVIACVKKQVQLRSYAKEVIKCFRYIVLYYITITTF
ALIISCIILLMNVPLIVKMQFIIICVTIMTEIYIYAWPADYVKNMSINISKSVYELSWYEQTLE
MRKYLLNVLIYQKPITFSISCIVPELTLRYYCS
>AmelOR156
MIEQVMLKRVIYITWLSVALCFCWPVSANTSRNQIIVFRFFQIFTIISSCLGSLPMFHSIYLHQ
DDIVIVAKSISIMVVLIQLIVQTTICAIKHDTLQHIIEEMITYMKEAKQYEKKIIQKYVSKCYI
LYGSAIIISYLTTTIFILGPIFLPISLPFYTEFPLSLNNTAVYIIIYFHQCFFAYQCSATVCLSIFGA

LLLWFVVIKFECLIMKIQNISNKDMMVICIKKQLQIRRYAKEIANCFRHIIFYTIIATSFNMIL
AGIILIMNPLLVIKIQFMITCFTALIEVYLYAWPAQYMDDMSKNVSISAYNLKWYEQTSEM
QQNILIMLIFQKPISLSINFLMPKLSLRSYCAYLSNAFSIFTALRVILKDNSI
>AmelOR157
MRRARPEKSVYLVWLSVAMTFCWPLPPDTARKRIVGMKVLLIISIVNGCAVILPMLYWIHL
HLDDIISLFKCICVALCLVQYVAQTIVCLVKYDTLQRVVDEMMGLIEERRMYEILRAYASK
CNTLYGASIASIYVCGTSFIFAPLFLPNPFPFETEYPFHVNTTTRIFIIYASHVLVIFQGTAHMC
LCMFGALLLWFTTARFECLIGELRGVTSVDTLVVCLEKHSRLKRYAEEVVSCIRFLVFHAIL
LGTFVLTLCGIVLIINSPLIVKAQFIIICVCILLEIYLYALPADYMYDMSMNISRSVYDSIWYE
QRLDLQKALLTVLAFQKPIAVSINVLLPELTIRYYCSYVSNALSIFAALRTVVE
>AmelOR158F
MLKQITPEKSIYIIWLSVALSFCWPLHINSTRKQIMYIKILQISAVVNAFMVLLPLIYTIHLN
MHNLINLFQCICLLICIFKHIIQTVICFIKYNALQRVVEEMMICVKEEQLYDILCMYVKKCNI
FYGGTIVLIYGTATVFVLGPIFLPISFPWGTEYPFQVNYTTINVIIYAHQFFLVYQCAAHTCL
SLFGALLLWFATARFECLIKELQKITSIDMLIVCLKKLLFLRRYAEEVVSSIRFLVFYAITISTF
TLTLSGIIMIINCPLFVKMEFITISISLLVQIYIYAWPADYMQDMSINVLRSAYNSIWYEQTLD
MQKTLLIMMAYQKPVTFSINVLLPELTLRYCCSYVSNALSIFTALRAVVEVT
>AmelOR159CN
YRLEHAMDTYKGQNEIEIYYIICTKLIKAIEVYKLAVKFVFFCQIFFFFQGNFNLHFPLLKV
VYMLQQLENIYKLCINLILLIRKFCFLFLITYLGQNIENHSNEVFEKCYDSLWYTAPVATRK
LLLIIMINIMKPCQCKMFGGLFKGNIEGFAQIIRICISYFMSLYSTQ
>AmelOR160
MRRPISSYVELFYDKNVISWSKRLLGLSGLWPDNRNDVRFFLYITYVVIFTWLEIVTLVQNI
HDLEKTLKNITLSFPTILIVLKAVMFRMNMHLVLPLLTVVKRDVNEGLYRSAEERRTVVW
YNVAATLFSTSSALSLFFVPTLFYAKPIIGCLLSKYNNCTLPFELPMKVNNVYEITKLQTYA
LFCVYLIPTSTLLTIGATGADSLLVTLTFHLCSQLSIVAYRMRNVNIEPKIYFPKMKALVERH
TELLRLANILANTFSSLMFVQTLGLIFSLCIVVYQLLMTSESGEDMNTIHFIIYSCAVILLAF
CYCFLGECLINESSEVQMACYFTNWYDLPEQYTRSLIFCIARAQKPLYLTAGKFYVFSLETF
AVIVKASMAYLSVLKSII

>AmelOR161
MGEFRNEEYDQLIKPIMITGKIISIWPLAENSSRITITFRRFHLFCMFFLVIVMSVAVTADVV
HNIDDLDEATECALICTAFYLCVVRLLVYSFHQKDMLYVVNTMKEDWLSSSDQDRLIYAE
KTMFAFRLAKYFITTVAITIVMFMSVPILEIYVIGNSDKVLPFRGYFFINQTVSPIFEFLYLFN
VTAGGFGGSMIAGATSFNLVVIIHGSGKFAVLRRRMEALNGADPNSAAIMGDNVIRHQQAI
KFADTLERIINLLALGQFVISTGLICFAGFQITSMMEDKGRLMKYSTFLNSAILELFMFSFSG
NGLIDESEGIGESAYNSGWIGSRFCRSVQIMMMRSKIPSKITAAKFYSMSLESFSAVLSTSFS
YFTVLTATKNE
>AmelOR162
MKRLMDIMQEDWKFHARLRNEYEILCEHYAIARKITTSFVAFLLGLTTPFGAMPLLLNIGD
ALGLCNISDDRPLAFRVEYFVDVDKYYYLLLVHSSIGTLGYTVIVLAINSIIIVYVLHECGL
CEILRESNNMICKIFLKDSLDIYTLDKIMNQFIIVQGETRKLCGNGCNGYRITPPQZKGZMV
SKCQGLCTLAQTHNRVSIKLITKFQLILTYLFFFLLIFQISRFLSLRFAKILEDTNTTSYLLQL
GFNMICISFTQFQAIINIEDTPKVLRYVSITIALLCDLLFVSWTGQQLSNSTERIFEYTTNGK
WYQSSISCRKLLAIMLSKSIAPLRLTACKLYTLNLESFTTIAKTSVSYTMVLCSLQ
>AmelOR163
MFKTIITYPVEVCLRLIGVWPYSSYRIMQRIFWTIIMGNSTVFQLWYCISYFKTADLFDLLD
GITLTLSNTVTFFKLIILWFNYRTIHNILTIVFEDWNNRALTDKKKQLMVDNTRLSSRISNFL
FGIYSVTCILYSASIALISDDIDNTNNELILNNKKLLLKMKLPFDFTIFPLYEFVIVAQFVFEC
FVALTAGMLMAFSAALVLHIGSQIDITCQELIEIPRHKGKTSYILKNIIVKHQRILRLSENVK
YLFLYTSLIQFLSNILVICFLGFILVNALGTEQESTIFIKCFPYYIAANCEAFILCYTGEYLMFK
NESIVHAAYDTLWYNLNPRDSRIVLLILIQAQRKLILSAGNFVTLSVQTFASMQKVSASYIS
ILMTIY
>AmelOR164
MAIKSIINRPVEISLRLIGAWPNSSCQILKYIMWTIVMSIFLIFQYSYCIIHIKTATLIDILDCLSI
TCSNTLLLLKFIIIWFHKRVLFESLIIIAEDWDNCKFEWNMEIMMQKAILSYRIAKLMLIIFIC
SIFMYAVSTFFGPDIGASHSDQKKFLLKMEFPFEATVSPLYEIIITIQLVMQFMFATMAGMF
MTIIATFVLHIASQLDIICDRLSEILDEHKEQELRIRIIKKLIAKHQRTLNLSENIENIFTFISLS
QFFFNILVICFVNFILVTSIGTEQAPTVISKCFPYYIALNFEALILCYTGEYLSSKSENISWIAY

NSNWYELSIYEIRVLLLLIMRSQKPLTLTIGKYMKLSLETFANMLKISASYASVLYALE
>AmelOR165C
MLVLNTLSPSVKFGLHFAGIWPGTPFPYLHKLGWLAAIAALQSYQYRYIVMHYKSDNLM
SIIDNLSIAMPFSLVFIKLIVTWINYGVFCDILSTMEKDCQKYAVIDINNLISKTGQISFYTTTI
VMSSYLVSAAFYITGTLAFQRTNSSISRELLFKMDLPFETNESPNYEFVVTSQLLIHVSAAF
TFGTFSALLLMMVLHIGCQIDILCQNLLDIPHISTSHLKFFIIRYQEIITFAERVEKLFTYIALS
QLVSNTLITCCVGFLIVIAIHEDNGLPLLLKSVLFYMVICLEAFIYCFAGEYLRIK
>AmelOR166C
MTSINTISRSVKYGLYFAASWPGASFSILHKFFWTIIFCTLHISQYSYLIMHYKYDALTEIID
NISICLPHSLVCIKLFTAWTQNTLIRNILLSMEEECQKYAIMDTDNLISKTAYLSYRLTSTIICT
CVASTVCYAIGIFSHQEVNVTSSRELLLKMNLPFDTNKSPIYEFVVIIQYFYQVSAAFVFGV
FAAFLLMIVLHVGCQIDIMCQTLMKTTHRDQKKLKFFIKRHQEIILLAEKIEKFFTYIALSQ
LISNTLITCCLGYLIVITLHLGNNIILIKYIMFYVAVCSEAFIYCFAGEYLSIKSKLIADTAYEFL
WYNMNPNESRLLIPIILRAQRGFTFTFGKFATLSMESFTA
>AmelOR167C
MIPIRSISHPIVIGLRLIGIWPKSSYEIIVRFMWVIIMMCAQIFQYQYIINHIGFDNLADLIDSV
STTLPYSLLCFKLISFWTKREIFENILIGMYHDWTNAFATDFIVEDMIKKTELAYYCSNLILS
IYAIAVFLYVGVFLELSHDHDQENRSNLSPELLIKMDLPFTYDESPIYEYVFIVQFIQLFFIAS
SIAVLDALIITLIFHIGGQIEILHKTLKNISINDEKPESSRIIIKSLIDRHYRIIIGSEYIESLFSYIA
LMQLICNTLIICCIGFLIVVALNSNLKLLIRISFFYIAITLEAFIFSIAGEYLSNKSLSVSISAYES
PWYLLSPKNRGVMILLMVRSQRRLTITAGKFMDLSMQGFAN
>AmelOR168
MNFQNLNRLNALANVVSGNFLPMTNINEKSSVISKIYFVIVWIIQLMYLASCTLGLFNVSW
ERALKDGTVNMVLLLEVIILNVYLHSRKKLLRELIGKLNQILINEDEIFRNVTISTTKMLEK
4PSRIYIIVNVISIIVWISSPLIKLFQKDEFYHEDFVMPAVFSNQPFSTGVFISGVFLQLFGGEYL
LFRKISLDLYTMHLNLLITSQYKYLRIKFATILKENGESAKDNDKTIRQEMKLLIRHFETVIE
MTGILKKLLSPNIGILYLNYVFRFCFLSFMFATTSLSEKLTYTIIVSYTTGALIQFYILCYCIQ
DLFEASTSIADDVVYEKWYSYDVRFQRVILMISLANELKCKISNFQNIDLTLPSFMSILNQA
YSICLLFLKTKQD

>AmelOR169
MKMNFQNLNRLNTFVNAVSGNILPITDMKKRLSIVLKIYSILVWTIELSYLAACILGLFNVS
RERALKDSTVNIVISLEVFVLIVYLHNRENLLRELIGKLNCLLIVDDETLRDVTIGTVKPLE
KPLRVYIIASVGSLMIWASLPLAKIFRKSEFYYTDYQVPAVISNEPFPIGVFIGGVALQIFGSA
YTLLRKVSLDLYTMHLILLITAQYKYLRIKFAAILEQETPKDFFYGGIIWQNVPCEYDKMV
KQEMKLLTRHFEIVVEMTVMLKKLLSPNIGILYINYVFRFCFLSFMLATSSGMHFEKCLLV
SYTIGALIQFYILCYCIQQLLEASTTVADDVVHEKWYLHDVKFQHIILMITLANKLKCKLSS
FRNIDLTLPSFMSILNQAYSVCLLFLKARQS
>AmelOR170
MNFQNLNRLNAFANMVSGNFLPMTNINEKLSTILKIYFVVAWIIELIYVAASFLGLFNVSGE
KALKDGTVNIAISFEVIVFNIYLHSRKKLLHKLIGKLNHLLITEDEIFRSVIIDTVKPLEMPLK
IYVIASVASLMIWILSPLIKLFQKDEFYYEDFIMPAVFSKQQPFSNDVFICGIFLQLLGGEDTII
RKISLDIYTIYLCLLITAQYKYLRIKFAIILKEEREITKDHYKNIIWRNDNVRQEMKLVTRHF
ETVIETTTILKKLISPNIGFLYLSYVFRFCFLSFMFAMTTAKYFEKCLLASYTIGALIQFYILC
YCIQRLFEASSSIADDVVYEKWYYYDVRFQRVILMISLSNELKCKISNFQNIDLTLPTFMSIL
NQAYSVCLLFLKARQD

>NvitOR1
MMKMKQQGLVADLLPNIRVMQGVGHFMFNYYSEGKKFPHRIYCIVTLLMLLMQYGMM
AVNLMMESDDVDDLTANTITMLFFLHPIVKMIYFPVRSKIFYKTLAIWNNPNSHPLFAESN
ARFHALAITKMRRLLFCVAGATIFSVISWTGITFVDESVKRIVDPETNETTIIPIPRLMIRTFY
PFNAMSGAGHVFALIYQFYYLIISMAISNSLDVLFCSWLLFACEQLQHLKAIMKPLMELSA
TLDTVVPNSGELFKAGSADHLRDSQGVQPSGNGDNVLDVDLRGIYSNRQDFTATFRPTAG
TTFNGGVGPNGLTKKQEMLVRSAIKYWVERHKHVVRLVTSVGDAYGVALLLHMLTTTIT
LTLLAYQATKVNGVNVYAATVIGYLLYTLGQVFLFCIFGNRLIEESSSVMEAAYSCHWYD
GSEEAKTFVQIVCQQCQKAMSISGAKFFTVSLDLFASVLGAVVTYFMVLVQLK
>NvitOR2
MTSKVSPKLEKAPLAYVNEQYLADTEYVVRVAKTLLMPIGIWPRYGDNSTLSNAIIYIRVC

LIFCLMLFLLTPHFIWTWFKAEDLRKLMKIIAAQVFSSLAVLKFWTLILNKQDIRYCLEIME
NDYRVVESEEDRQIMLKNAKIGRFFTTAYLGLSYGGALPYHIIMPLLQPRVLRSDNTTMIP
LPYPSEYVFFIVEDSPLYEIVFVTQILISSIILSTNTGVYSLIACVVMHCCCLFEVTSNRAEKL
LRGMKYDKSKISPELGKKLSELIDFHVKAIQYAETMENALNIVMLSEMGGCTIIICFLEYGI
LQDLEDREYLGMVTYIMLMTSIFVNVFILSYVGDKVKEQSEAIGFSAYSMQWVDLPNEFI
MKDLKFVMARANQPTRLTAGKLFDLSLQGFCDVAKTSMAYLNFLRTLEIT
>NvitOR3
MAEMKRMEDVFAYYDERMKKPGPSCSNEKFEEDVKYATALNRRIANAIGIWPIFTSTGAR
LGFDICVKTLKNAAVYILLSFLLVPGILHIVVEEGKLKAKILKTGPMILNTMALLKYSVMLF
RKSQIQECLKQLESDWRKAGNDELRALMRRNTAVGHRLSRVCVATFYVGGIFYRLIKTLL
TPIRYTKDGLMIKPLPSPLYKGLFRFNTSASPVYETIFATQMMSGFVVHSTTVTTCSYAVLL
ATHACGQLDIVVYLLKRLIEDDGDNGRLTRVGNEAVDRKLRVIVQLHLKVLRFISSVEDL
MNQICLVEILGGSTILCLTSFYFIVDLQSNDALGLFTYMVMITSLIALLFTYCYVGEIVSDKA
KKVGAKTYMINWYDLPPKKGLCIGLIISVAHSPVQLTAGKMLELSMYNFGCIMKSTAGYL
NLLRTITD
>NvitOR5
MLGEKSHYAVQLNRLFLTPIGVWPIGRDAPLVQRLLKRLAIIGCYLLMSYLLVPTALHTFLE
EPDPAIKLKLIGPMSFHLMAIGKYVSLVGRTEEISACFEHVEEDWKMYSDKNAKPELEMM
KRNAKIGRFLIYLCAAFMYGGGFFYHMIMPLSVGRLVTKQVRAERYLQLVAENASTDNID
VEPVRVLSYPIYGLLAKLDYVTLLLVQFVAGFVLYTITIASCSLAAVFANHVCGQLEIVMSL
LRDFVHDNEDNPRIYALADDAATVERSRSDKFAEIVQRHLRALNFASRVEKNLNAICFVEF
IGCTLNICFLEYYFITEWENQNTVSTMTYCILLISFIFNIFIFCYIGELLTEQSKKIGEVTYAIN
WYTLSGKRAVDLIMIIMIASCYPARITAGKMVNLSLGSFCNVIKTSATYLNLLRTMML
>NvitOR6
METQSTKRIENDRGFNYAVKLTRLLMISCGIWPAKFSTSFQKCLRPILIIICFFIMFFQLIPFCL
FMFLIIKDMRIRLKLLGPLGFSLTSLFKYVVVVIKNREIAKCVQIMVDDWHQLNSTEDRKA
MLINAKTGRVLTMVCMFLMYGGGMPYVTIVPLTKGVTMVGNVSYRHLAYPSYYIFFNPH
VRPIYDVIFATHCICGFTRYTITCAVYSIVIICVMHICSRIAITSSMLQRLADDSDGRLLGTAV
KHHLDILKFATKLENIFKEIFLAEVLGSTYQICLLGYYFITEYEQRAGIATATYLFLFMSFVF

NIFILCYIGQILTEQCESIATTAYTSKWYQLSGREARSIILIVHWNRRRVVLTAGKMLTLSLES
FSSIVKAAGGYLNILRTAVANSN
>NvitOR7
MIESVYKLKMNEDNKQNLERNQGFDYAVQLTRLLLMPCGIWPAKFSSRVQRFLRPFLIVA
CCFVMLFLLVPVCLFMFLIVRDVRIRIKLLGPLGFSLMSLFKYAVVIIRSREIEKCIQNMLDD
WQQVASDEDRDTMFENARTGRVLTMVCMFLMYGGGMPYVTIVPLAKGATMVGNVSYR
ALAYPSYFIFFNPYIRPVYDVVFLTQCLCGFTRYTITCGVYSVVIICVMHICSRITVTSSMLQ
RLADNYDNKLMGTVVKHHLKFLNFAAKLDNIFREIFLVEVMGSTGVICLLGYYFITEYEQ
RESIATITYFFLLMSFVFNIFILCYIGQVLTEKCESIAKAAYTTKWYQLTGKEARSIVFIVSCN
HRPVELTAGKLLKLSLNSFSSIIKAAAGYLNILRTAIVNSS
>NvitOR8
MYSAMDNHDIKDLINAEDFEYAIQILRWLFQPMGIWPLKSAAYPSFLRPISIVISFWSAAFLI
IPGILSVIRVQNDFALRLRLIGPVSFCLVTSFKYFSFLVKNRQFYAYLINVALDWREMKKNN
HNRIIMLRKTQISRFFMTSCSICMYLSGMSYNILLPLTKAPTQVGNVTFKNLPYIGYYIFFD
QYADPYYYVVFVMQCMSSFFCYSTCCGVCCISIQSVLHISGRCDITSIMIKNLNGNCNEKA
LKAVVEFQLQSLKFAREIEKLLNQMFLVEFVGSTFNICLLVYYFMGDFKENDTVGTMTYV
LLFISFTFNIFIFCYLGEHLTEQCASAGAAVYTMDWFRFSAKKSRDLFLIVLFCQRPVVITAG
KMVNFSLLSFASLMKASAAYLNMLYKMG
>NvitOR9
MVKICPDMNSEDVSAIKNAQGYAYAVQLTRWLLLPLGLWPTKSIIYQKILRPVAILLCLFIM
LFVIIPLCLFIFLVVKDLGIRLKLIGPLGFGLMSLFKYVVVIVKQRDVASCFLGMAVDWQEL
SSLSDRKVMLRNAKTGRLLTIICVIFMIFGGMPYITVLPLTKGPIMRGNVSLRPLAYPSYFV
FFNPQIRPIWDYVFVTHCMCGLVRYSVTCGVYSIAILCIMHICSQITITSSMLDRLVENFDN
MLLGKIVTQHLRFLKFASKLEDLFNQICLVEVLGSTCIICFLGYYLITEYEQREPIATVTYFL
LLCSFVFNIFILCYIGEILTEQCESIGTTAYMIRWYHLSGKEARNVVLIIASTQRPVVMTAGK
MVNLSLQSFTNVIKASASYLNMLRTVTANAN
>NvitOR10
MSKQNLISNLIELKSIRNAQDFEYAVQITRWLLQPLGIWPMKSSTFFSSILRSLSIATCTFLLG
FLLVPCCLHMFLIEKDLGVRLKMIGPLSFCLMNIFKYYAVLIKDGQISSCIVDMAGDWHRL

EGSEQRGYMLENAKTARVFTTICALFMYGGGLPYSTILPLTRDAIIVGNDSYRHLAYPSYFI
FFNPHIRPIYDLVFFAHCLCGFVMYSVTCGVCSIAILCIMHICSQCSITSATLRSLTSDVDEKT
FGKIVTQHLRSLKFASKLEKILNDMCLVELIGCTFNICMLGYYFITEFEQSETVGTITYSLLL
ISLTFNIFIFCYIGDLLTEQCENIGEVAYMINWYQFSGKDARNIILIVASTQRPVVLTAGKMV
TLSIRSFCNVIKASVTYLNMLRTLTASES
>NvitOR12
MADKKGYEEAIEATRAVLRAFGVWPNRHKISENWLSRSHFLAPAFLIICFINIPQTLKIIKV
WRNLNEVLDILVTANIPSFVALIKLLCVRYNKKVIGLLLVSMENDWKSLKTLVETRIMWK
NGKLGSLITLVIYTLTCGSYVAYVIMITYINVGGSKQEDVITLNESKKLRPLYMRSYFIYDV
QKTPVYEIIWIFQFVSMGVATFTFMAVDSLFAVLMMHLCGQLINLQERLKNFTNMLGQTK
TRNFSYQLSTIVSRHEQLNRFAKAIENAFNTMFLVQMLLSGMVLCLQGYQIVIILTGRDTV
QIIELLFMVYYTLCFAFSLFVYCYIAEILRIESMEIGNAAYHCDWYDLSAFERRLFILTIIRSK
TPFEITAGKFAAFSLEFYCSILKTSGGYLSVLLAVQDRLAA
>NvitOR13
MTVEIAEDSMERIVALSDDQNVDGYNHAIGPCRFFLRLLGTWPDPYGNVDSWTTSARCL
VITATMFLFATISQTVKMALSYKDLNLVTEILTNCNIPTTIATIKIASIWYYRWVLRDLVRQII
EDWEMSHDRHESAIMWRSAKISRIFSIGCMFMTEGTLLTQCVVGLFRPISYAFKTDLNQSI
EWPLYMKGSFPYDVQSSPNYELSILGQLLSNVFASTSFSSADSFFIVLMFHLIGQLSILKLTI
LDLPSKIENSDDRSKFIDRFAFVHMRHNRLWRFSMAIEESFNTMFLIQMIPCIFALCTQGYQ
LIMIMDADNVSLMELIFMIYFLVLFLFTIFTYCYVTEILRCKSLELSYAVYDCDWTILPAKEA
RILLLILVRTQHPFEITAGKFASFSLPFYCRILKTSAGYLSMLLAVKKRSEQVASKVVL
>NvitOR14
MSKKSGFDVAVGPSRAFLCFVGVWPNPEGSETTFETIQCIIVTLTMIIFANIAQTVKVFMVW
GNLNSVIEILTTADMPIFVALMKFLVAWYNRKVLKGLVILMMEDWSRSYSSSNLDSMWRT
ARFSRKLSAVCIGLAQGTITAQFIMVVVFDVNNKGEAERTLYMISYFPYDTQVSPNYEITW
LGQCFSNIFAAGAFSAVDAFFAVLVLHLCCQLSILRKELVMLADHHKKQGDNSEEFSRKLA
RIVEKHEYFNSFAKTIEDSFNTMFLSQMIASSLALCLQGYQLVMIITNTEGKLPVFQLIHMI
YFTCCFSFSLFVYCYVAEELRFESTELDYAAYDSDWYNLPPKDTKLLLLLMHRSRKPLEIT
AGKFCAFSLRLYCSILKTSGGYLSMLLAVKDRLVVEAD

>NvitOR15
MAEKEQGFQTAFSVTRFVMRFQGIWPGVDKPRTGFSRFQFIPAALMMVFFINAVQTMELT
RVGGDLNMIIDILTFADIPIFIALVKHVGIAYNNKVLYKLLYLISEDWKEVTKESEKKVMWQ
KARLSRIFTMIEVSLGLGRLFIHTIRMTYAMLHPTSFDPTGKLIRPSYMRGYFIYDSQSTPIY
EITWGCQFVATAFGGCAFASADALFVALVFHLCGQLTNLQTEFREVGKNTSGKKLEFVRSL
ARIIKKHRRICHMADTVEYCFNKIYLVQVSSSSVIFCLHGYSLVTILFDQDDVVVELIVMTF
FTLGFIYSMFVYCYVAECLSTESLALSSAIFDNTWYDLPPKHAKLLLLPLQRTGKPLIVTAG
KFVVFSLNLFSNIIKTSAGYLSMLLALREKL
>NvitOR16
MDDKEGFEVAVKASRTILRVLGIWPNHHERTESWLSRSYFIMPTFILVYFTSFPQTMEIIKV
WGDLNSVLELLTTFDIPNLISLIKILSVWYNKKVLGLLIMAMENDWKSLKTVFELRVMWK
NVKLGRLITLAIYLLTYSTVATYVVMAVYITANAYKQEFILTPDNSTKLRPQYMRAHFAYD
VQKSPVYEIVWIFQCIAMHLAGLSFMAIDSLFSILVLHLCGQLINLQERLKNVTENLTKRH
NLSYQLSRIVMRHEQLDRFAKAIENAFNTMFLAQILLSGVVLCLQGYQIVIILTSRDTVQVT
ELLFMIYFILCIAFSLFIYCYIAEILRTESTEIGNAAYECNWYDLPACETRLFILTMIRSKTPFE
ITAGKFTAFSLQLYCSILKTSGGYLSMLLAVKERLAL
>NvitOR17
MSDKEGFDVAIQTSRTILRFLGVWPDPKRKESWIYSGHFLIPAIVMFYFVNIPQTMMVTKV
WGDLNAVLEVLTTSDIPIGIALFKMLGIWYNRDVLGQLVVSMSEDWKSVKSPEERDVMW
RNARLSRLLSVTIIGLAEGTIVAQFAMVIYFNVLEARQYSLTKDNVTARFRPLYMSAQFFY
DAQKSPNYEIHWLFQCSSTIFAASAFSSVDAFFAVLMLHLCGQLNNLREKLKKLPKQISDK
GGGSFVEKLSEIVTRHDHLDRFGNAIEDAFNVMFLVQMVASSMVLCLQGYQLVMITTAG
DGIPLFELIFMIYFTCCFTFSLFVYCYVAEVLRTESMEVGNAAYESNWYDLPSCETKLLML
VIIRAKKPFKITAGKFAAFSLGLYCSILRSSGGYLSMLLAMKDRLAS
>NvitOR19
MTTKEEGFDVAIGITRFVMRTHGIWPGFSVSKAGIMRYAYLPAALMLLLFVIIPQTVQVIFV
SRDLNAVLNVLTLGNVPVGIALAKLLGVSYKQNVLHQLILSVCEDWKHTTKESELVVMR
LNARKSRMFSIICIVLSEGTAMAYSARMFYAAFSTHTKAQATGIDDCEKPLFFIGKFPFDPQ
SYPNYQITWTLQIIATFLAAGAFSSVDALFVTLVLHLCGQLTNLQAAFSEIGEENAEKGTM

FVSKLSKLIERHRKINVFADIIEYSFNMMFLVQVLSSTLLLCLQGYLFMIILSGQDGLLVEMI
FISYFTICFTFSIFVYCYVAELLQEKSLQLGYAIFYSKWYNLPAKKARLLIISIVRCKRPLEIS
AGKFCIFSLNLFCNIVRTSAGYMSVLLAVKDKIT
>NvitOR20
MARKEEGFDVAVGFSRFFMRLHGIWPGDTSSKFTWARFAFVPPAVIILMFINIPQTVQIFFV
GGDLNAILDILTLANVPLGIALAKILGVSYNHNILRQLIVSVSGDWKHTTKKSELQVMWR
NARISRTFSILFIGLAEVTVLANTARMFYILYSTRSEAESSGIKNYKKPLYYTGKFPYDAQSS
PNFEITWVMQILATILAAGSFMAVDALFVTLVLHLCAQLTNLQTAFRKIGEDKHEKEVDFM
SKLSKLMKRHRKINEFADIIEYSFNMMFLFQVMSSTFLLCLQGYLFVILISSQKVILVELIFM
VYFIICSSCSIFVYCYVAEILREESLQLGNAIFYSKWYNLPANKARLLIIAILRVQKPLELSAG
KFCIFSLNLFCNIVKTSAGYISVLLAVRDKIVQP
>NvitOR21
MKIRKSGYDECVGFTRLIMTIIGTWPGAEYSQHWYARYMFSIPLFFSMFFMIIPQTRMLLH
VKDDLNYIIEILTTADVMIIVACLKLIGVWYNKKDLRYLLNEIEKDWTITEKEEQHVGNAM
WENVKLGKFIMNGYAVLTYGTVVLYAAGMLLLMNSQKIEDFDNENITQSRLMFVRSKFPF
ETQGSPTFEIIWFLQFLAAVMSIAAFTTFDGFFIFSILHVCAQLVNLQCNFRNLISRCRLTKRT
FVQHMRDLVERHIHLQRFTQIIENNFNKVFLMQMIGYSVTLCLQGYQLVISLTENSEQNFIT
IAFILVYTTANILSLFVYCYVAEKLRKESTEIFYAVCAMPWHEVKPEESKMIVNIMYAAKHP
FEITAGKFAVLSFSYFVKVLKTAMGYLSMLLAMKSSHKM
>NvitOR22
MMANNNKLGFDESVGVTRWTMNVIGLWTLDERRDLQTRFRSLLPAFLILFFIVIPQTRKAT
LAHDDLNLMLEILTTADIIEGICLLKIFGLWYNKADLKKLVIQISEDWTHTNNDEQGIMWS
NARLSKFVCLFCISSSSGSVLTHAIVFLVTNVGANETRSLFLISQFPFNTQHSPVYEIVCFCQ
FAGALLSTFIFSSFDGFFVFSILHFSSQLSNLNIRIRSLTEKTSGDKCQFVESLKSVVKHHQH
LISYTDIIEYNFNKIFLVQIFATSIVLCLQGYQFVMIISESGTKLLTSLIFILVFTTGNVLSLFMY
CYIAEIIRNESQRLLRAVYEMKWYTLPAKDSCLLLIVMCRLKMPVEITVGKFAPFSLEYFAS
VVKTSVGYLSVLLAVRNKIND
>NvitOR23
MEVKTLVKSDTQISISNNLNGLSGFDHSVKVTRVISRMCGVWPGFEEKKSFTERFFFIVPG

MVTFFSITLPQLRRVMIHRKDLSTVLELMTTGIVMELISILKLLAIRYNQSGLRWLLRRMV
DDWKIYDKGQYYKIMWVYARHTNTIVTICIALTTGNIAAQIIRQYAIYIIERHYSSANETVI
KPTILKSDFYFNEQIEGIYELVVAAQILGGFSVAFSFTAFDGFFVCSIMHVSGQIHKLQMQIE
DLVQCYERREGAFSEVLGPIVHRHRDLRGYAAVIEENFNKIFLVQMLVTSVFLCLQGFEFA
MVVAEGGTEMVPHLIFIVCFVASNLVSIFTYCFVAEQLRTQSNQLFRSIFQIRWYDLTPKDS
RLLIIIMVQTKKPIEITVGKFVPFSLDYFCSVLKTSAGYLSVLLSMKDRL
>NvitOR24
MEESPGFLHAFGICRTCLTMSGLWSDTHFKKSKKFVISVLYAANVFVILTFMNVAQTVKLF
LIWGDFDEMSQIISTSDFSVGMLVVKMFVFRSYRKALALLIEFVEKDWLDLKTISEEETME
QNAHTANKIYLTCFFLGNSAVNSYTLLRLGQEMSFLPGPPDKRQPLFDAYFPYDDKRSPAY
EITWLMQYAGIALANLAFTGMYCLFVGLMLHLCGQFANLRIKLIEAVSRKEGESEKKSDG
AKTFRERLAFIVERHNSLNKYAQVIEKIYHWIFFVEILSSTIQMCSQWFMLVTVISNTQGGL
PYLQIGFLLIFTAHSGFHLFACCYAAERLQNESLSIFEAAYSCEWYNLSPQDAKMLLFIMQR
TKTPLRVTAGKLCVFGLELFAKILKTAGGYLSILLAMRDRLVIDEEPI
>NvitOR25
MDGKRGFDHAFSLCRINLGTVGLWPNSKNGKGHQEVASLIFFIISLFTIIVFVNLAQTVKLI
MIWGDLNHMIDNISTANLPIAVVVFKMLTFRRYKKTLTRLLGIAMDDWCTKKTSREAENM
SKNARTARKMSLVCVVLGFGSVNGQLAVRISQELDILPGQTEKRLPMLSSYIPYEYQTSPA
YEITWFMQYLGAVLATLVYSGVYCVFVGLVLHLRGQVANLRFMFESVDDPEEDKGKNFR
RRLRSLVERHESLNRFAEDIENIFTLMFLAEILSCTIQICLQVFLLVTLMSNDNGGVPILQILF
MMVYAMHVGTHVFICCYVADKLRDESLSICDLAYNYEWYRLPARDARLLLFIMLRAERP
LEVTAGKFCAFSLRLYAQILKTSGGYLSMLLAVKDRSTNF
>NvitOR26
MDKKRGFDHTFGMCSINLGIVGLWPNSKNTKFQEFRSNVSFVFAIFSVSVFISMSQTAKLI
MIWGDLYQMIENISTANLPITVTVFKMLIFRSHKKVLGELLALAIGDWCTKKTEEETANM
CANARLAHRISMICVFLAGGTVSIHAVLRTCQELDIMPGPPEKRLPLFSSSYVPYDYKSSPI
YQVTWLMQLTGTSCATLVFSGVYCAFVGMVLHLRGQVANLRLKLENICEIREKGEGLVE
ARRDFRKKLGFIVERHLVLNRFAADLETVFTLMFLAELLSCTIQICLQVFLLVTLLSNIKHG
FPILELFFLMVYIMHVGTHVFICCFVADKLREESLLICNSVYNYQWYKLSAQDAKMLIFV

MHRGDRPLAMTAGKFCAFSLQLYAQILKTSGGYLSMLLALKDQS
>NvitOR27
METKAVAMTDSRAQVSNYFPDSSGFHKSINITRTISRVCGIWPELEEKKSIAARYYFIVPTIV
IFFTMTVPQVRRAVLHRKDLSAVLELMTTGIVMELIALLKLLGIRLNESGLRWLLRRMIDD
WKTSNSKERNIMQEYSNLTRFIMTLCITLTIGNVVAQTTKQFAIYFMERYQSMANETVIKP
TFLKSDFYFNEQPEGIYEAVVAAQILGGFYVAFAFTACDGFFVFSILHVSGQICNLQLQIEGL
VQNHEQRRCSFIKVLAPIVVRHRDLRGYAAVIEENFNKIFLVQMIATSIFLCLQGFEFAMVIT
KSGSEMVPYLMFILCFVASNLVSIFTYCYVAERLREQSENLFRAIFEIRWYDLAPNDSKLLII
IMTQTKTPIEITVGKFVAFSLGYFCSVLKTSAGYLSMLLAVQDRL
>NvitOR28
MDGEKGFLYAFGMCKKSLTLIGLWPKSKSSNYAEAVVVLRFTLTTLLIVSFVNIVQTIKLLA
VWGDLDAMTDIISTANLPIAVAVFKMMVFYKHRKAFEPLLSFVEADWKSYKTDSDMTNM
WSNAQTTRRISMICVILGAGTVNGHLFIRLGQEAKILPGKDGATRLSFVDSYFPYDYSPTPI
YEITWAIQYIGAALATCAYSGIYCLFVALMLHLCGQFSNLRKKLRRVVTNEDDKRKFVEK
LAEIVKRHENLNNFARVIEKIFNLMFLAEILGCTIQFCMQGFFLLTLSSKEGMGLPILHILFM
VIYVLHIGTHLFICCYVSEKLQDESVSIVRAAYNCEWYNLSAKDAMLLVMIMNRAKKPLR
ITAGKFCAFSLSLYAQIFKTSGGYLSMLLAVRDRIT
>NvitOR29
MDDKKGTSFIHAFGLCRINLTVLGIWPTLRSSKRDETAALFRLVLSLTIIILFINTVQTIKLFI
MWGDLDAMTDIISTANLPIGLMVFKTFVFLYHKEALVPLLSFVQTDWSNFKTVSEAANM
WSNALAARKISLLCVVIGWVTVNCHLAIRIGQELRFMSGKNGLTRLPFFDSYFPYDYTPSP
VYEITFVIQYIATMLATFGYSGLYSLFVALMLHLCGQFANLRDRLYTVTQKKAGVTFQQRL
GYIVMRHQCLYNFAQVVEKMFNLMFLAEILGCTIQFCMQGFFLLTLSSKEGMGLPILHIMF
MVVYVAHIGTHLFICCYVAEKLQDESVSIAKAAYECQWYHLSPKDVMLLIMIINRAKDPIE
MTAGKFCTFSLSLYAQIFKNSGGYLSMLLAMRDKIT
>NvitOR31
MDDKDGFEYAFGVCRKELIIFGMWPKPNDTMDHKVFAIFRLVLCIALNFIFINLVQTIQLFI
MWGDLFAMTDIISKASLPIGLVLFKTLVFIYYREALLPLLAYASSDWKKPKSSLEAANMWS
NARTARQLSITCLFIGLSAVNYHMAVRICQELRIIPGKTKVERELYFNAYFPYNYTESPAYEL

TFAMQYFATVLATFSYSGLYGLFVGLMLHLCGQFANLRVKMDKVAKQADSAKFRQNLTAI
IIRHQFLFRFSQIIEKIFNVIFLGEILGCTIQFCLQGFFLCTLSTEDVGLLVMYIFFMVFFIGHIG
SHLFICCYVSERLQDESVSIANAAYKCQWYHLPAKDVMLLVMVINRAKDPIQITAGKFCV
FSLSLLAQIFKTSGGYLSMLLAVRDKIT
>NvitOR35
MSDKIEDAQKKLETREQRLRDFKWALGLNRLSLRLMGVWPGDDEAEGLGRLAILLRVPF
MIAAMFFCLFLPQMGALALVIHELPLVIDNLMTSCAAFTCCIKLYFVWRSKQVLRPVIQSV
SADWLRPKLDWEREAMIREASRARIFTVSGYAVLAGCYTGFAFAPLFGFDIRMISNITDYG
EKHLLVQSYFPYDYSKSPNYEITQVSQLIAGFFIGMSVSVPDNYFGALLFHASAQFEILGAN
LENLVRQDDKALRSRQFNRRFGIFVDRHVHLMTMVTAVEYSFSFVIMAQIFCMSIMVCSL
GFQILGMIEGTTADKPSLLQVLTLLGTLFTLMMHTLVDCFACETLELRSAGIFENVYNSRW
YTVPKQSVAKDVIPMMVVSKNPRKLTAGKIFTLSLATYCSILKSTAMLIAVNRR
>NvitOR36
MERSQKNQLQDFDWALGLNRFSLRLMGIWPADQDESSKSLLTVSRIPLMILVLLCGLFLPQ
MWALALVIEQLPLAIDNLMTSCPAFTSCIKLFFIWRSKTILQPVIDSALQDYLRPKSKSEETA
MQREALRGRLVTIADYSIMASCYVGFIFMPMLGFNVRIINNLTDCDTQRVLLVQSYFPYDY
ARSPAFELTHLLQLAASFFVGMAISIPDDYFCALLFHASAQFEILGLQIESLPIDGSKSGRLL
SGFIERHVHLNRMVSAVERSFEFVIAAQIFCMSIMVCCLGFQVLRMLDSAAEKPTPVQILT
LGGTLFTMLLHTFVDCFASENLAARSSELFFKIYSSRWYSLSWSKMRCLVPMMLVAKTPR
QIRAGKILSMSLATYCSIIKSTAGYISMLIAVSGR
>NvitOR37
MESLEKYRSQEFDWALGINRVSLRLLGIWPADQDESSKSLLTVSRIPLMVLVIFGGLFLPQM
WALALIIEQLPLAIDNLMTSCPAFTSCIKLFFIWRSKTILQPVIESVLQDYLRPKSEWEELTM
RREASKGRLITIADYSLMTICCVGFIILPTLGFHVRIVNNVTDYASYGNRALLVQSYYPYDY
YESPAFELTNLVQLTAAFFVGMTVAIPDDYFCALMFHVSGQFEILGLQIENLMGKDDAKEG
VDWSLLGSFVERHVHLNRMVATLEKSFEFLIAAQILLVTVMVCCMGVQVLRTLNGAGEK
PSPFQILTLSGTVFYLLLHTFVDCFVSESLTSRSSEIFFKIYSCRWCALPWNKVRCLLPMML
AAKTPRQIRAGRIMPLSLATYCSIVKSTTGYISMLVAVSGR
>NvitOR38

MKSNNAHESFFAYLNWAIGLNRLSLRLMGIWPDDSVAETKLFTTILRIPLIISVMMLCIVVP
QMYALILVRNNLLLIIDNFMTSFPTLIGCAKFYFLWRSKEVLRPVVCSVTEDWLRPKSDLE
CQKMRDAAVVARLFTVGGYSLITGSLMGFIIAPLCGLNIRVEQNITDYGRQPLLVQSYYPY
DYSQSPNFEITHSSQIVAACFVAMSLAVPDNYFGALVFHISGQFQLLGLNFEHFIKQNEKIV
GIMAVRDFNKSLGVYVDRHVHLIRMVAIVEKSFNFIILIQIFCLCVMACCLGVRILSAIGNP
NDKTAVIQIINLGATLISLMIFAFSNCYASETLASRSAEIFQQVYSSDWYKIPKRSTCCYLIMI
MIMSKNPQMLSAGKILYLSLSTFCIILKSIAGYLSVLIAQSN
>NvitOR41
MHRMRTRVKRLKVSRSSKTKGGFSYEFRIYKIITWPAGLWPLERDNIFNVLRFLLAASSQM
FIVVAALVEIYRKCGNVADVLDYYALSIAFWLSFVRLVLVRIHLAKIHKICYNARKTWARIK
DPDLVKIMISHAKTGKRFYYLQMSIAFVIVTLYVFNPLILRRYDAANLPMQTVCTFNNADV
LKHTAVYFIETLSFVYLAVGFISIDLLFLGIAMHLCGQLKILQKEFSEIVGKSTSQADCIRYVI
SLSRRFQRVVELTDDIRKTFSEILLVNFVVNLFLITSQSVTLLLALKINNYFLAVKCSQTFPIL
LIEMFLYCYVGELLRHAFDDIPRAIYSSRWYLLPPKIRRGYLLHVMAQASKTFDLTAGKMI
RMNMCTFIQLVRSIVSFFSLLLLMFDK
>NvitOR43
MKCFDSYDKSNFKTDPQHAINFFKKLGRFWTIWPVSANASRFTKVYHECSLWFIIINLFFA
SLTLWMSVCVYHKYPILMAKNLSQLMIISDSFTHLVLYRINRSELQVLVQEVFDFMKNSKQ
NEKYIMRKHYNRFLGHYTILIVLYVIASLAFFCGAFILGKKFPMDASYPFSTDSILVSSIIFTH
QTFSIVQNSVLIMIDLLVITLFWYAGARIKILGYKFKIVDSNEKLKNCIKEHQKIIQYVASIV
KAVRFILYKTISIVAIIIISAGLQLLYYDAKVVISQFSLIIIVACFRIITYSSTIEEMNQLNEDLRW
TVYKSSWFCITSEMKQCLQIFIHRCQIPLVTIDGQLLNIMSLAFFAKLIYSTVSHLTTLRAIIE
RS
>NvitOR44
MAFKISPEKAFTFTKLSVFFTAIWPPNCNDSSFKIKLANIFWIYSIISAMCLLIPMLASVLVY
KDNPMIVSKSICLSCAVIQVIAKAIVCRHHQKKLQFLVKELTHFLKKAKKEERQLIEKYINR
RAIFHMTFTLCCFGSSFFVICGPIFLPFSLPADAVYPFSVNSSPIWEIIYVHQASVGIQASSGM
CVDNLVAYILWYTGVRFESLYYKFKHIKDSKEMLQCIKEHQYLLRYGTTVADTFRYVIFTT
VLSVTAGLAFAGIYLFSPQPIFVKGQFVVVSISVVVNLYVTALPSNNLISMCHKVGDVVYE

SLWVGDSPSIMKHWIFIIQRCQKPVVIAIPGLIKELSLQFYSSVLCSTFSYFSALHVIMTKE
>NvitOR45
MKFLIRPKFFFKTLRLIGDMIAIWPKHIGAKKTMIMFHEVKWWLSFTNATGLLIPLVLGVY
YFRNDSITMTKTLSELTALCEVFINLIQCRLQEKKFQVILYEIENFIENSNEQEESLLQDYLN
RYKTLQLFVGSSFISTAILFSCIPIFTSQLLPADAWYPFSVEYFPIRVFLYITQVLAIFQTGFGIC
VDLTVATMLWYSAVQIELLEKNVHKAVSKAELRECARRHQEIIEFTDNIKKGIKFIILKTNA
TMIIVVICGAFQLIHHEPLEVLLRFTLMVLAGCLRLYVSAKPADDLKENSEQLARTAFQTA
LMQKSTSNSKIGLMLAFRCQKPIVLSVTAVIRAYTLQYYASFLSRTVTYFVNLRAVLDD
>NvitOR46
MRKMKCTFTFSIISVYETLKYFGLLTSIWPIFSKNRYLWVFMKAVYYFILVNYLCVFIPLIL
MTLFNINTSATVTITAVEQMIIVEAVYNLLYTRFYSAQFKSAVKEIEEFFKNSSPKERYILDRY
ATTRTFFNIYIAINYFVAIMSFNFGQLFLKDRPYPLNLWYPFTIKSQVIVVIIYIHQVIVITHTL
ILIVFDLIVQIFLWTLAARFELLQADFKKTASEMDLKCNIQKHQYLIRTTEAVIDFTKYMILK
VFLAVTILVISSTLQILHRGPSTIIVQFFFIMKIASMRAFAYCWAGHSLAEKTGGLARSIYNS
YWINQTQRMKTNVLIVMQRCQKPTVVKISGIISSLSFRFCVNYFYMIYSAFMTLRAVLEV
>NvitOR47
MSSWRMGSIKDIMMNVFMMKVIGVALAIWPLKSAGKRWYYAFLQEMVYRFFHVNFWLL
VIPSLWSIYKKRHNLASVLTSVTQLTIVFEILAIMVLSRRQAARLKTLLTMAYDYVSVADD
KVYPVVYKYVRKAQIIFGIITIAYALILLTYLVQAFIENKPPIYAYYPFDIKSPVVWICVYGN
QLLCTFYAAVVIIMDAMMMFMIFVTSIRLELLQNDFKKVKDYPDLVKCIRTHQDIIWYIKE
VYCIKKYMVLKMLISIAIYIMCEGLQLFALNLSWGMRFQVSLLFGIGLFRVYIYAACSQDLI
SSGLDLGYFVYSSLWYNQSHSVMVAKAFVICRCQKSLGIRVCGITDDLNMKFLANFLYRV
FSYTMTLRAIIKTLR
>NvitOR48
MMINKKVTDKLVTIVSTLLNIFLHIDYLFHSFKILKFFSRLFALYPLNSDCTKLEILYDNFVW
LFIHFHFWVAAAATLVAIYKARSDLSIWLIAFSELIIIIEIIVAMILYRLQRSRLKILMHIFEDFV
KDPDDSKIQLIRRNAKEHIKVFSILALLFIIVVIMYVYRALSTRPYQLLLSGYYPCTSDSLII
WLVVFFHQCILVIYSPSTFASDSIVTVLIFAAIIKLQKIRPRFRNIENYAQLVGCINEHQHIIWF
VQEINYVIRLFVFKSIFCLAALQLGVGVILFMPNISVFTRIQFLLLFTVTIFRIYIYSYCAEILT

KSGLDLGFAVYSSRWYDQRRKMVLAKSIIICRCQKPLLIAINGIIPALGMRYLARFLYLTFSY
IITLQAMTRT
>NvitOR50
MFKKIKPNFTVQKNFNILCNCMKILGTWPVHRRHNKIFTCLNHSLWWFYVVNHMMLLLP
TMQTFYNTTKDIISASYSLIEITGIVESMVILITFKLQGSRIQLLLQIIKNQIVVKKPKPALNN
RNVHASVFAIIAVLYVIVVYMYIHKPATLINKGFIMTTCYAFPTEDIRTKIAIYCNQLIALMH
TSVVLVTDGVAVLFIYTCAIKLKTLEIRLKKAPDWTKLKYDIAEHQTILLVIEETNSLAGVL
VVKTVICFMCYSISAGVQIINQHVVTAQMLHQFIIIAIVYLRIYICAETAEMLLTVNGDMLF
TVYSIAFSTPNIVKVKSLIIMRCQKVPKIYVNGLMAALNRAYLRSISYATFSYFMTIRAIVSK
>NvitOR51
MRIHLNAKIAYQYLRLSATLMATWPLSDATKKYRNVFYNMLWWLYLTNHLIILYLTLNTII
THNKNHLTVFYTWLEISFMTENIIVLVSYKLQETKWKQLLYTSKMTINRTEDNIRLENSDL
YPKVFATLFIFFIVIIISYVNKAETYERGLIMTTRYPFEIKSIGLKLFLNLSQFITLLHASSILIT
DAIVVLLLYTCTIRLKIVEQKFRACKYYRHLKLHIYEHQKTLLLIEDTNLLVSKTVLKSIAS
FMSYSIGGGLVLYNKNTSPLQLVQICLVICVIYLRVYVCAEIAEKMISANESIGFTIYFTKWY
EESAKDINAKNIIIQRCQKLPRIYINGFMQSLNRNYVRMITYATFSYFMTIRKIINKTANCVD
C
>NvitOR53
MQNKFKLRDRTNKFTFMFKSYSGVHLGFLCVNFYMKCLGIYPLPSTVSKFWTRVYNLLW
CFYLSNHLLIIFPTFYAFGSTTQDIAVATFSLMEGLCMIECIVLLIHFKYQRSDFKILLSLVHH
ELNKKKRIITLDNGNVYIIAFVLIAIMYVLIIFNYIQRPETVRYHKLLTTARYPFSTRAATIKII
LSCHQIVVLLHMTIILTSDGLAVLLTLICTVRLKNLETKISNEKRGKLPKRIREHQQILLQVE
ETNLIVRIIVIKTVFCFMVFSISTGLQIFHKFEIIQIFIVMIVFLRFYVSAESADNMATCANNL
GIAVYSTAWYEEKTKIRIAKTIIIQRCQKSPRIFITGFMSELNRKYFLVVAYATYSYFTMIRTLI
SKNK
>NvitOR56
MSVYISPNRSFRVLRFLGTHLRIWPDDNKKWNFKTDVFFWFCVINYVLLLLPLFNALYLN
RKNVVAASNTWIEVSGYAEVLAAFIYSKYKRVQLYVLLCEAEKYLLFKKVTIIKKYANTY
AKIFLLVIVFYLFTVFVYWSIEKPITGYEHLITTAVYPFNIRSHPIKGLIYCNQTFNLVYSSILP

VFDGISVLLIFNCTHRLKILEHKFKLAKTSSDLSECVREHDDVSRTIKETNSIVRFLVFKTVC
SFTSNVIPGGLQILNNVALSQSICQVCIILLVYSRIVLCAECAGNMTDAGEDLLFTVYSTLW
YNEEPKIVSMKIFIIQKCQNIPAIHIKGIMSGLGRKYLLTIMYSTFSYLTTLRTVTSDEKS
>NvitOR58
MTIQSVLRRKVDVLLKAIALNKMCVSPKMILLVIKFAAMYLAIWPLDSSGKHWNTAFDCL
WWFYVVNNVLVIIPTLLAFYSSRRDIIAAMFSWLEILALLEALIILANFRYYRSRMQPILKE
AVDYIGSANSRRQLCLEKRASIITTTFGVIVALYIAGIIIYIYRPAVTEWDGMLTTAYYPASMR
SPFADVFIYITQLTALLHNGVLIVSDAFTVLLLYVCTVRLEVLQKNILRVADYDELKLWIRE
HERVLRLVTDTNMVVRINISKTVISFVGYSVGAGLQIISPTVTIVSFQRFALVIAMNAMRLF
FSATFADDLVNSSNSLINTIYSTIWYKDNRDMKIGKIIIMLRCQKLLRISVGGIMPVLGKPYL
TKILYTSVSYFMTFRAITGN
>NvitOR59
MSRNLYHDAKIKCKMNVITYLLKCLNFLRFMGKIYAVWPLKTDDNIRWRFVYECLWWFY
FLNYLVAASFTLNTCGHASDDITIASFSWLEFVSMVESIIILINYKCYHVTLQLLLTEVEDYL
TLADEKKQWVLKEKASIFAVMMCIITFLYFVLVVLYFTNPAITAWETFLTTSYYPPAIRSPV
MDVFLFSNQLIVMCHTSVIVNLDAMVVLLIYICSVRLKVLAADLESVNDDEELKQRIREH
QHILCLAKKTNIAVRLVVSKTVICFISYTVGAGLQLVNPTATVASLQRFGIVLLINYVRLIMN
ATSADELLTVSRNVGLSIYSTDWYGESKIVTSSKFIVMLRCQKLVRIHVDGVMPALTLTFIT
GIISTSISYYTTLRAVTRQN
>NvitOR60
MSRVLQSDSSSREGKHVWSKDAKFALMLNKFIVWPLGLWPLECDDAFSRFRNFYAVVSQ
VWMIGTQATAAYLGCGDVADTVDFVMMTACALMALSKIVTIRLHMSKVHTVFVSALDD
WLAVDVDKSRDVLIPFAKTGRFVFYLQMVSAYMSNTLIIIGALPFLIPPAANGTWANVSET
LQSRQLPMRTGCMFAGYRDEIYGSLYVYESVMIMITAHGNVGCDVLFFILAMHLCGQIEL
LKTDVLKIGEDEKVPGEWKNKIVECVHRHIRLLGMAKALNKVVSGVLVIQLLLNAGLNL
MLGIRMLIEIKRGSIFNAVRPMIGFNVLMLQLYLLSYASDRLSSQAESILDAVYDSYWYKLP
AKLRRDLYFVTMRANKPIYFMAGHFYAMNIENFMNILKASFSYFSILRIMFQA
>NvitOR61
MGGKSEVDEAFAVYRAFLWAIGVWPLEEKSFSQILRYIVAAVVQVTFLLHTFTEILLNNGK

VSDMVDVFFFSSAAFLTFAKHTYLHLHKDAIRENLRCYLDDWSNTKDEHFLRIMREHVKI
YKYQFHIYNLCGYVGTTLFMCRSILINILAKRQLGPGESYNYQFICQTSYLSQDTLAKYYPI
IMAIQYIQCMYCCTSGACTDCFFFGLVFHLCAQFEILKIKWERLGTKDFGVTAVHDRVKV
NALIARHKELVKLGENLESGFNNTILVQLMISIVLICMSGCSILVAIMRNDHVTMLISTNSIS
FMVTETLIYGYASDYLVTQSESIVQAVYSSSWYDMDSSVKKDIVFVMMRAKIPLHITAGKF
FCVTRNTIVQLLKTSVSYLSVLRLTLEMSHQEGQL
>NvitOR62
MCANIFIGHHQFGLRVVGSWPGKSQLPGFYFAIGIMLFFLIFEILNITEVYHDLEELMDNLV
STIGVVLGLFKFITVRVKRRKLKTVINKIFDDWKTDSQFVSEMMVKNCTRSQLVSKFVIFL
YNSMNFTYFLRTVISHIFDEVQDRKFLAQVTFPIVDGRQTPLYEIIIFFQFITASVCFNSQALV
EGLLATLVLHACSKVDVVRREILNFSTICKTDKNDKKDILKTLRKLSEEHFKFIEFSEDIQDI
FSYVSFFHIFFLTLIQVVSGYMFIDGLERGTKPVNLIHYAILTTSFLVSAGYYCIAGEYLTSQS
EIIFNELYNCYWYEFPSSYKKAICFMLLKARKPVKLTVGKFSTLSLIYLTSIMKTSFSYLSLV
RAVR
>NvitOR64
MSDKIVMRHVRVALQVIGLWPGYTSSVGFVIAITWLLTCLTFQLWHAAVVFSKLDALMGN
LGATMAVATATLKLIAFHVKGRNVKIVIKEILNDWAYENRSSNCEVMVQNTKRAKYLTKW
ITGAYNATVITYLVNAIIAYCSGITEQRLYVLPSKFPSFCKQSPVFEIVCFFQFSAALISTNVQ
VLVEGMLTVLVLHAGTKVFLLQKEIQKLSVICQSKTNNKEVISKSTIALINKHLNFIKFVKE
VKDIYYFISFVHVFTFTFLHVIVGYMFIDTLERGDRSIKLFLYGLFTTRALASTTIYCIAGEY
LMNQSMRIFDELYNSAWYEFDVPNIKAITFMIMKARNATSLTPASFGQLSLFYLTSVIRTSFS
ILSLTRATR
>NvitOR65
MSILSRHVKIGLYAIDAWPGVSSSGLFFLVMAYMTFSLIFQILNTTEMITQLDLLMNNLQTT
MPVILVVLKLSVFRVKCRSARLIIADMLSDWKCINETKERKVMMKNAKIAFYLSSTIAICY
NGLILSYLLKAILAYETENIYDRKYVMQATFPINAKSSPVFEMLCLFQFTVSVFAANGHAIL
EGLLTTSVLHANTKAFGVCQEITKFAKSCEANKSRKNIVEAKRRLIKRHLYFINFAEKIQET
YAYISFFHLFLMTLINCIVGYMFINLTINKDNISALLLCIAYMFTALSAVGSYCIAGEYLMSQ
GSLIFEKLYDCPWYKFKPVDTKTFIIMLMKSRHSVTITAGNFGDLSLVYFTNIIKTSVSYLSL

VRAATN
>NvitOR66
MIPIFNKPLECCLKVAGFWPYDFNMLGPVAITSMLVTTLPFQCWNAFALTENLVVLMDSLS
DIFTEVLIYIKIFILWNHRREIRDLLEEIGKDWSIKSIPTEWENIADYCRIICNIDVIVYASASIL
YYPDLLMSYFGKPVNERHMLFQSYYPFDYRRSPIYEVINIVYFFQGILMIIADSVSKTLFIS
MIFHVSSQIYELRNNLEQYSRHSNDGYENKNFKRLKLVVQQHLKILSLVRRIDHIYSYVAL
FQIVFSSIIICVTGFVIITAMESANIMLLVKFMTFIIAMLAQVSYFCFAGQYLLNKGESIVEMI
NSSFWYNSQCKDVKVLIFVLTNAQKPLTVSGANIFNLSAETFTMIVKTSASYLSVLRAMYT
Q
>NvitOR67
MILLINKPLEYSLKLSGFWPFEFNIIGSLALISTLVTTLPFQCWQAFNFTNDFVLLMDSLSDI
LAEVLIFLKLFAMWKSKSCITIILREIFDEWSTEKIPDEWKTLAYYSRMFCNIDTLVYFSAA
ASYYPDLLMSYFGKPIENRKMLFQSCYPFNYLGSPTYELINLMQMIQAVAMMAADSLSKT
LLVALILHVIANIDLLKNEIRIYSTNIANTCNHTNNKKSTVDLKQVISQHRKILYLVQSIDNA
YSYVSLFQIVFSTIIICVTGFVIVTAMESANIILLFKFILYIIVMLSQAFTFCIAGQYLRNEGESI
IHEIYDCLWYYTEPKEIKSLIFVLKSAQIPLTLGGGKLFELSTNSFTMIVKTSVSYLSVLRAV
CV
>NvitOR68
MKIPIIGIPLEYTLKLAGLWPDQSNILGSIVMGSALVTMIPFQVWDTINVSDNLVMVMDNL
SNILSEVLLYTNFIVLLLNKSYLDDLLREIADDYKNNIVTEKWLKLDQNSRRFCNYDYGM
YLGACCLFYLQFALMYTQMPSEDRIMLLKAYYPFDYKSSPVFEIMCFIQVIQGLLMCSIQA
LSESLLIALVSHVSGHIDLMNKQINVVSKSYDGQNSLTLKLVIKSHLKVLNLVNKIESVYTY
VSLTQVCLSTFIICVTGFVVLTMNSANEIVVMIKYIMLYFTLLWQSFSFCFAGQHLLNKSD
MIPYQVYDALWYKAEATEMKAILFIIKRAQTPLSLSAGKFIALSAQTFTLIIKTSFSYLSVLK
ASYA
>NvitOR69
MKIPMVYWPLEYTLRINGLWPGENNILGSIVTASGMVLILPFQVWDAIKTIDNPILLMDSL
SDIMTEIALYAKLIIMWFNRRYVVDVLKEISNDCNQNDVSQNWTLLNYNARRFCKYDYS
WYISATLLYYIQLVTMYIEVPVDGREMLLKSYYPFDYKSSPTYEIMLFLQIILAMSMAIANA

MTESLFIVLILHACSYVDLLLDEIKIFSDNCNKKVLNITDSNNMRFYVHVILKRHIQLLESV
KKIENIYSNVSLVQMFFSVITICVTGFVMITALESKDIVLLIKFATFIWFLLWQIFSFCFAGQY
LLNKGETITGAMYDSDWYNIESNDVKAISFIIKKTQRPLSVTAGKYIPLSVTSFAAIVKTSFS
YLSVLRASYVE
>NvitOR71
MYDEIFIRPHKISLKLIGAWPGYAKLTGFFLVIGSSSVLLFFALWNTIEVFGNLELLVDNLVN
VIGIIVGFFKLTTLRVKRRNLIFMVDTMFEDWQTSKKTIEELNAMKDHFERSKWLCKSIIM
LYNSLILTFLLKPVRSYMNDSIEGRQYLAPVSFPKFIDAKQSPIYEIVIIGEIGTAFFCINSHAL
VEGLLASTVLHASAKIAAVRQEIIRFSKVCRSQNSNKRLIISATRRLVQVHLSCNEFSETIVD
IFAVISFFSILLMTLAQVFSGYMFIFNIENGGETVQTLHYGFLTIVFLVSSGYFCIAGEHLANQ
SELLTMEIYNCFWSEFRIPEQKAIRFILAQSQRPVRLTLGKFDELNLVYLTKIIKTSFS
>NvitOR72
MDDEIFIRPYQISLKLVGAWPGCAKLSGFFFVTGWSSILLFFALWNTTEVYENLDFLVDNL
VNVIAVVVGLLKLTTLRVKRRTLMTILNKMLEDWQTMKMIEEFKAMTDNFERSKWICKSI
VMLYNSLILTFLLKPAISYMNDSVEHREYLAPVSFPKFMDAKQSPMYEIITAGEIVTTFLCL
NSHALIEGLLASSVLHACSKVDAVRQEIVKFSDVCRTQSGDKMLKLTAIRRLVNVHVNCD
EFSENVENIFTVISFFHISLLTLMQVLSGYMFILNLEEGGEILQTLHHGLIIIVILVSCGYYCIA
GEYLTNQNELLNVEIYNCFWTEFPVPQQKAIKFILAKSQRPVRLTFGKFDQLNLLCLTKIIK
TSFSYLSLVRQVH
>NvitOR76
MSTKKIASSIDSFLWPNRYTLEFLGFWPPEPGTSSISKYFAAFRIVFSILAIGFLFVPEIMMVV
VFWGDITVLTGVGCVSTTLAQLNFKMLYVLARRRRFCRAYRKTRELWSMTDHESELRKG
LEKLAGQAKKYSIAFFFTCFCNNISFTTLSVVVWLNYNAQENKSLLERRLPFDVWFGFDL
QRTPNFELVFVGQSISAIFCCFGIVGLDTAMMALILHVCGHFRVIGARLRAIGQGMHNDVQ
SKNSVEYLHTSPKLAIWQCIQYHQQMIKFAEEVRSLLSPIIFVQLLTSGLEICLSGYAVIVNS
DAGNYGDLVKCTGYFLSVFIQLIIWCWPGQILIQDSSEIGRIVLHDLPWWDMATEQQRQFV
FVIFRTQKECQITALGFQVMSMSKLTDVFNTAGSYLALLRRVYEKETEE
>NvitOR77
MAGRGSSVRIDEYLWPNRYLLELFGTWPTDYDGRTLASQLFVNFRVCFVFVAITGVLVPEI

LMIIVYWGDLDVLTGVGCIATPVSLILFKVAYMIIRRNRFHGVYSNLRRLWLAIDDAEEFEP
LEELARLAKRVTIGFFLSCFSNNVSFTTAAVIDWVNYDETRNDSTPRHLPFDVWFSFDVER
SPNFEIAFGCQVISSLYCCTGIVGIDATMMTFILHICGHFRTIAAKWRAIGSKILDNEKYSKS
GQVMPVKKDINQILRQHSEMLRIAEEVRRLLAPIIFMQLLTSGLGICLSVYAVTMNGSKGA
DLFKFIVFFVSIFVGLIIWCWPGQLLMQDSAALGDVVCYELPWHLLGVAEQRNLAFFIMR
AQKECQITALGFQVLSMNKFTEIFNSAGSYFALLRTIHEKQLEAQ
>NvitOR78
MARSFASFDEYTFLNRWGLTFLGIWKSDAEARGGPLRRFLHRLHVTILFTLLMLLLLPQW
MDMYVLWGNIDANAETFVLNVFTITALLKLWCFLSARQIFEVPENARAATEARVYFLKFEI
KQVIDTMKENWRRTMSGDEPGRKTHREILLDMAGKARDYTKRYGLLMYSTATMYFVSP
FVGMQRDNVRIRKYPFFGWYYFDRFSNLYYGICYASQVIIGIVVGTSNYAMDSIFLVAIYHT
CARLQMLQHDLKKIGEDRENRSPEEIVQLIRLHQREIRDAKRLTKIFNGSSLQQLLVSCVIIC
IIGFKLIIALNDGGFEFLVYVAFMFVALLQIFLYCRPGDELIVQSTAVGYAAYQSHWTSLEAE
SIRKIMFMILRSQTSLKMTAGNFYVLSLPNFTMILRMSMSFLSLLRAMYRKSDGFG
>NvitOR79
MRIGARRASRMESTTEASGIMREYDDCIFLNRLGLTMVGIWPLEHNASRLRIVLRRIHLGA
IYVLMLSVVIPQWFDIYCLWGNIDANTETFMSNVFMIAVMIKISNFLNSMRLFEDVLRTMR
LNWLDVMRLSSGELEKKEIMQGLSMKARSRGRVYGLVVVMTGAMYGLMPLIGSNKVAS
LRDRSYPFFGRYLFDRNSDTVYRLCYLSQLMSGSVTAVANFATDAIFLFCVYHFCAQLRIL
QTDLLKLGGPRFDSREALVQLIRRHQKEIRNVRALQSLFSISSLQQLFLSCLMICLNGFKLI
VSLCNREVDILMYIVCLPVTLFQILFYCQPGNELIVQSQSLDEAIQQSHWVNLDRLSKRQL
FFMIQRSQKPLAITAGKIYVLSLENFMRIVKTAMSALSVLQAMYRKTGS
>NvitOR80
MHCSYSFFLVTAFAMWRPRSWDDSKILTALYTLYSILSFTVYYTFLISQILDIVLLAENIQQIT
ENMIQLINVVNVSQKSLCFFLKRKKIIRFMDYFFEDMTLPQSPREKEIQKSFDDESKGNSQ
KLFVLYSVSVVMYVYMPFFISKREDRVLPYRAWRPYSLDNVNYYYLAYLHQSWSVTIAA
TGNAATETLVSGFMIQICAQFEILEHRFMQLPKILKEMRENGESESTVLATERSIIIKLIHHH
WRIFEMTELFNDIFVFVILSQFVTSITVLCVSTYNLALCKSVNNDFVTIFMYLLCMLLQIFM
YTWYGNEITLRSCDLGNRIFLSEWRSLNPPTVKNLLIIAQRTMKPIILSSGYVITLSNVAFTSI

VKTSYSVFNVLNV
>NvitOR81
MHILSLTFTFFKIYGFWRPLSWKSPTLGFLYDVYTFVMFMIVFTFALSQLMSIILTVQTVDE
FTSSSFILLSIVSACFKASNLLLKRKSLVRLLNVLISTTCKYQDDDEKMIQDMFDKKARRN
TVWYMALIQSSVFMITLQSIFINIPQKTLPFPAWLPYNYSNTRLYAISYTHQVIGNAASATLH
AANDALISGIMLQICAQLEILKHRILKLPTIVLKMNSGKEAPMNTVASKESELLGNIIKHHN
CIFQFSKDINDTFSMALFAQFFIAALVICSSVYELSKIVLLSSDFVALLSYLSCMLVQIFLYC
WYGTAVTMKSWSVGDTIFATDWSPLSMGLKKSLLIVMIRAKKPIELKTGKIFTLSILTFAKII
KASYSAFNFMQQA
>NvitOR82
MRVLPITFGILTVCGFWRPISLESSIPKQMYNCYSIFMCFLIYTFTLSHLIDIVISAADFESLTG
SCFMLLSMMNVCCKMTNILYFRKNIVELLQILASDHCTAKDVVERDIEKKFHKRARSVTL
CYWILTETTCMLITLRTFFGSSKQILPFKAWIPYEITGLAVYWTTFFHQTIAHVAAANLQIA
NETLICGLMIQACSQLEILKYRLKKIPDESKIDKFPLQSTVNNAQNTNKKDTKTLLVNCIDH
HRRIIEFSEKLNSTFNVILFVQFAISSLVLCSSVYLLSKMKLVSVHFMSLSLYLSCMLYQIFLF
CWYGNEVILQSLDLGNAVYHMDWTILSTEDKKKLLIVILLVRKPIQFTSSFLVSLSIESYCKI
LKTSYSVFNLLQRTSI
>NvitOR85
MRSLTFTFKVLSLCGIWLPLHWQSHRRLRLFYKIFSISTVVLTNIFILLQGLLLALSEFDWQF
LAEILFTLLTAFSVSFKATNFLMRRDKIICLADMLLKSWCIPRNAVEIEMESRINEFLRVFTIY
FNALAQLSLACLLIMPLVQDPDKRELPFRMWLPYDIRNQWNYWSTYVIEVGPMIVGILLN
VTTDVVVSGFVLQACIQLDMLKHRLNKLPNIVKVAKRKRLASEEVVRSFERKTLHQAAR
HHDYIIKYAKVVTETFDVVIVEQFFAGALIFSVIIYVLTIGKVPILQKLMSVGYLICMLGELF
AYCWFGNEITLKSLEFSDDIYKIDWMALSDSSNKKLIFIMMRATQPIIMSYGHLVILNIESFK
SILKITYTAFNILKESTSTT
>NvitOR86
MLELPYKLLILTGIWMPEDWTHKHQKLGWLIFSIISIGLVFMQFSSLVIFLMISKSCAQFFER
VFLIPAGVSSLQKIYIFITHRKELIDLGKMLLKDYCIPRNFEELSIQHRYEELIRVLTLVCFVL
VNITMMNLLVLPLVTNGENRTLPMNVWLPYPVDSDASYWLTYTHQTLGTLLLGTGAVGS

TLMINGFMHQVCCQFEILSSRFQKLPQIIKRLQLLKKPNHLIYEYEKKSMKQYVQHHLYIF
RVADTINDIFKSVIFQQFCISSIVVSASIFQLSTRPDKDMEFIMVFCYLICVLVEFLIYSWFGN
ELMLESLHFQTSVYQIDWTALSIGSGKDLVFIMMRASKPVIMYCGHFIILSLESYLGILKAS
YSVFNILRRSSN
>NvitOR87
MHILYLPFKLLTLTGIWMPEDWTQKQQKLIWVLYSMVSIGLVFMQLSSQIGYLMQSKTWA
QVNERLFFIPTGISSVHKIFIFIVHRKDLISLGNMLLKEYCIPRNAEELSIQERYNEIIRVLTLA
CAFLVNVTMMNLVTLPLVTSGDNRTLPMRVWLPYKVDSDMSYWLSYAHQTVGIVFVGT
GAVGSTLMINGFMYQVCCQFEILSSRFQNLPLIIEKFQSLKKPNQLIYRYEKRVMRQNIRHH
LYIFRFAEALNKIFKSVIFQQFCLSSIVVSVSIYQLSTRPEKDLEFIMVFFYLVCVLVEFLVYS
WFGNELMLESLNFQQTIYEIDWTSLSTRSSRDLVLIMMRASKPIIMYCGHFIVLSLESYIGIL
KVSYSVFNILRMSEE
>NvitOR88
MHTILQLPFKLMTLTGIWMPKEFTSQYEKQGWTLYSIASITLMAIQSLTLSITLILSENSEQF
FETLFIVPTGLQNLQKIYVVVAHRKKLMDLEKMFSNDYCIPRNVEELLIQRKYDENIRILTL
SCIILMNLTVANLIASPLFDAYFTTMNTRTLPMRIWLPYKMDLNIIFWLTFIQQSVGVIFVGY
CIISTTLMINGFMYHVCCQFRILSCRFKKLPQVIDYFRSLKKPYNVIYQYERRAIKQNVQH
HLCIFRIAENINDTFKSVIFQQFCISSIVVSASIFQLSTRQEIDMEFFMVLFYLICVLVDFYIYS
WFGNQLMLESLNFQRSIYEIDWTTLSTNAGKDLVFIMMRASKPILMYCGHFVVLSLESYV
GILKVSYSVLNLFRRSK
>NvitOR89
MEIIEQLEKMRILQVPFKVLTWSGVWMPEDWTQNQRKLKYNLFSFVCIGLMTIQSCSLTV
YLMMSKTWSQFVETLFLIPPGLSNLQKIFVIMLHRKKVIDLVNMFENGHCIPRTADEWSIQ
QRYDATIRVVTLVCFVLVNVTMVNMVTTPLFLKADERILPMKVWLPYSIETDFFYWLSYM
HQTLGVTLVGSGIIGSTLLINGFVYQVCCQFEILSSRLEKLPQIIRNLRSLKKSDHLVHQYEL
KLIKQIVQHHLYLFSIAETVNEIFKSVIFQQFCVSSIVVSASIFQLSTKPDTKTEFIMVLFYSIC
LLVELFIYCWFGNKLMFESLNFHQAVYDADWTVLSNESGKDLMFIMMRASKPIIMYCGH
FIVLSLETFLSILKVSYSVFNVLRRSHG
>NvitOR92

MQSLKVSFTILTYCGIWQPIYWTSGWHRTSFNFCRVVFRPLPYLLASAQLARIALVDMSFE
ELTEVIFILLSIVNICCKSVSILMRRADLIKLTKMLGIVSASPQDSDEFNIQHQYHQFIRYVTL
SSLVLVEITAITFLIPPFFQPENNRTLPFKIWLPYDYSMDKLFWITYFPESITIILASLISVSSNT
LIFGFLIEACGQFELLNHRFMTMPLYIEDFAKGEKITTYEVCKLEKQLLSRNIRHHTFIFEFV
DIFKKTFSSAIIGQYIVSSLVISTSVYQLSTNTTMDVVFFTNLLYLMCMLLEFFLYCWFGNE
LTVKSEDFGRKVFRTNWLALSTKSNKDIFVAMLRSSKPIIVSTGFFAVLSLESFMKIIKLSFS
AFNVLRTASDYQ
>NvitOR93
MHVLPESFMMFTCAGVWQPVHWSACDSRFLLYKLYTLFSIVLVYTLTISELMGAILLTQSL
EDFTDISFLLISTISVCCKIASIIARRDRVIHLTEMLLEVQCIPKNVRELEITRKFDKIARFTAL
SCIVLAEATVVVMSTGPLFQKAENRTLPFKSWLPYDSTTTPCTFWLSYVHQTAAIVLCATV
NVANDSLICGFMTHSCSQLELLNRRLLELPRAVKLKMKKLPRRLMCNVEAMIVSRHVKH
HVHIFKFAENINVIFTPVILVQFCMSSIVLSLSVYQLAVRSANGIQFITMVMYLTCMLVQFF
MYCWFGNEVTLKSVEFGQAIYNIEWTSLQVQTSKDLMIMMIRAKRPIIMSSGALVTLSIKS
FTSILKASYSTFNVLQRSSHN
>NvitOR94
MHVLPEAFNLATYIGLWEPTHLESSIARCFYKFYTCLSFALIILTMITQILAMLFFTKTLDEF
AETAYMLLSAINASVKGVVILLRRKHVIDLAEMLLKKECVPINATEKRVCSYFNKISRYTV
LSCIVLAEGTISALALLPVVFEQGELVLPLRAWYPYNAGSGLGYWLSYLHQAMALTLIAA
YDVANDTIITGFMVQACAQLELMTCRFHRFSWRGSNAVMRNGARHQLRLFEKRMVAQS
VRHHLLIFRFTEIINSIFAPVILVQFCLSSGVLCITVYQMSASKSNGLKVIVLSLYLVSMLVEF
FLYCWFGNEVTLKSLGFNIAVCEMDWTAMHVQTLKELLIIMVRSTSPIFLSCGPLIKLSLES
FTNILKISYSAFNVLKQFD
>NvitOR96
MLSIHFQVLTISGVWCPNHSSSVQRIFYKCYSFIVVVLMYSLALSQLARIIFVKQSFNEFND
TFFISLSTNFACFKAASNLVNQKQIVSLVNMFKHNCCLAHNDSERSIQKQYNDSCSKIIISLL
ILVETSAFFVVVAPLCGTMDNQDLPYQVLLPYDLSNKLFFWLTFVHHSFGAVLFTAISITND
AVITGFMMHVCGQLIILQHRFALLSRSLANEVSKKGRITDFDMMLERHWLRQIVYHQNHI
SNIAKKICSTFNEIVICQFFISGLEICVSVYQLSVRNNNTVELCTYAIYLMVMLGQFFVVYCY

FGNEITLQSKITHRAIFDIDWTSFSLSLKKDLTLIMLYSSKPIAMSCGPFAHLTLESFTNILKT
SYSIFSVLKTAT
>NvitOR98
MNNQQMNEDLAPIPFRILKFCGWWRPLNMSTWRRAVYSCFTVIMLTLLVTITLTVLIGVT
QMSATDDLFADNVFLMFALINSVFKATNVLLSRRRFIKMLEIVQDTRWRDLRNDEEIEIQD
RYRKTIRKISVYFTTAVFVAIILRVVAPLLDLSDEIKLPVDAYCPCDIRHSSCYWTLYWHQAL
GTGVATLTHAAKDCLISALLLQTCAQLEILKNRLLSIADTCVVAGNKTGAADRVEKLEQKL
IGDCVRDHESIFEFAKILNDSLNVMLFGQIAVTIPNLCLSIYLLSTQKIASMDFMMTTQFFSA
VVIELFFFCWYGNEVTLNSLDVENAISEMDWTLLSTRSKKDLLMMMVRTSRPILFRVGPI
MNMNIDSFLSIMKTSYSAFSVLQSTGD
>NvitOR99
MKFQDSIEYQLLPIPFMVLTLCGTWCPENWSKKRKRIYKCVTTVLVSLGIILLVEMLVFIIV
KSGKDNIDLENIFATICIAVGLYKKINILYHRPKLMNFISNYTKNEWNKPKNFEEATIHLNIL
SETRYISYAYAAFILVSIIFRSITPILESGTFIILPLDACYPYNADNFIAFSLTYLHQIISGVTLTC
MHIGTDTLFVGLLLQMNYQLHILKNRLRQLGNSKTYKNNTQTIKDRELFIKSKISQRVREH
ESIFRFGYDLQKTFKPILMAQMVVVVPSVIINVYFLSIYTDRLNLKYFMTFFFALVSLMQIY
MFCWYGNEILLSSSDVGDALYESNWFALDQSTKKIMLTMITRSSKIFLISAVAIIPLDIDTFIK
IMKTSYSAFNLLQRTTAQ
>NvitOR100
MHEKLIAIQKANVEYELLPFQFLLLTIWGIWHPKDWPVRLKNISNIIFIVVFCLDIIICFEMSI
YLVLSIGTNDFKLVNIFFTSATITGIYKAIKTMQIRESFRTILLNYFNYEHLCSLNTKERMIRE
SNQAQIRKVTVIYSASMAGIFALNAIAPALSQPDSTMQLPVDAWYPYSIQKSLNYWLTYFH
QIILGSSLICVHIGTDTLFVGLLLKLVCQINILRYRLQSLTSLCSKNFEHFNAMGRKFIYRYIH
HQNEIYEFSKVLNNKFQAVLLIQVITSIPNLCINVYTLSKYSGIINMDYISIFFNTTSSLIQLFI
TCWYGNEVLLSSLQIKKSIYEMDWTKLDVPTKKLLIVIMARSLRPIAFSVAHVIPMNIESFI
KIIKISNSAFNVLQQT
>NvitOR101
MHLKFVGILRSNIEYELLPFQFVVLTVWNIWCPKDWPRRLKNTSIILFIVILILNFIMCTEML
IYFILSIGTEQFKLTNLFFVSASITGVYKSLKIMKNRKIIRCFVRNYFNHQWIKLLDDEENEI

HEKINTRIRHITVTYFISMISIILMKDLGPIAESGLAIQLPADGWYPYDIENSVLFGITYVHQV
ILGSFVICAHVGIDTLFVGLLLKLLGQINILKHRLQILGNSLDHKMISLNKFESFQTVQKHLI
LECIHHHKRIYRFGEDLNKIFQEMLLILVVSSLPNICINIYALSSNLKNINMDYIATFFSTTSA
FIQFFIACWFGNEVSLNSVEVRNAVYAMDWNKLDTPTQKLLIVVMARSLKPIEFSVGYIIP
MNVDSFLKIIKASYTAFNLLQQTSSS
>NvitOR102
MHSTLAAIVKNNIEYKILPFQFFLLTFLGIWCPSNWSLKSKTAHNVYFTFIFFLDFLICIEMFI
HFVSSFGTDNFKLINFFLVSANITAVYKSIRLMQNREVLRYFIISYFDYEWTKSHDSVEHEIN
SKIDLRIRRVTVIYSASMIGIVLLKAMSPIAESNGISLPVDAWYPYSIEKSRWFWITYLHQVI
LGSSAVGAHIGIDTLFVGLLLKTSGQIHLLNYRLRNLMLLKECNFAKLKEYSEKNVVLRCI
YHHKRIYRFGGDLNDKFQEILFILVVSSLPNICINVYSLSSYKGNINVQYIATIFSTTSALLQF
FIACWYGNETTFDSLQVINAVYEMDWTNFHVSTKRLLIFIMLRASKPLKFSVAYIIPMNLDS
FIKIIKASYTAFNLLQQTTN
>NvitOR103
MDLSQCLEYRALPMQFYIFTLSGVWCPSNWTSLLKLSYNMYTTTIAISGILFWASMFVNLI
ITKNESEYFYENVFAISTLTYAMYKEFFVLKKRKEIQQMLKLSFDDEWYRPFDNREIQIIDH
YAHETRWVTQVYAIGIIAGLATKAIMPMLNSNSAWVLPIEAWYPYNTSNLKNYLFAYTQQ
LMGGIPLICLHISVDSLFVGLILQMCIQLKLLQYRLQKTFSTDIDLQEEKNIERNIKISDVIIA
NYAFKHQCIFRLGNYLNQEFRGILAGQVMITIPNICINVYLLSQHRGGITLHLVDSFLCFTT
CLMQIFLYCWYGNKIILLSIDVANTAYTTNWLSLNISSKKKLLTIMVRATRSIQFAAGTFIMN
IDSFIEIIKTSYSAYRVLQKTS
>NvitOR105
MIIRKTLEHQVLPIPFHILTLWGIWCPEHVQPRLRRFYFAFTCIVIISEILLTTEVFINLIIIIRNK
RFELDVFFILTSLMNGLYKALNILLTRKRIAKLITIGFEDRWRFPRDDSEKKILQNYKFESW
RIHLIYAGACLAGVTIKLVGPMMKQNADIEFPAPAWYPYDTNKPVYFWLAYVQQMFVGG
ATISMHIGADTMLSGLMLQSCIQLKLLKHRFKHFFQHYEQVKGRLHLSSTKRKVEIALMK
QYICDHQFVYSYANKINRNFSGWLIAVLIVVVPNICINVYLLSFSKIGLNVDFITSLGLFSISL
FQIYLPCWYGNEVMLHSSEIANSIYDMDWVRLSPTARKTLIIVMIRSSKPIQIRAGYFVSMN
LRSFLSIMKTSYSALSVLQQTT

>NvitOR106
MTTTTIIERTGSADVYGIENRLFSISFNVIKLSGFWRPTTFRKPFDYLYEMYTLFCLVGILML
IATIIVDNVVTEKSIRSLIENLYLILTVSNGISKLCNIYHRRDRVISMLQRSSEDRWSVHRDEE
EARIVEESIESESYIIRFCIYLVTINNVSNALNPILNPDPEHDLMVDAYSPCDRSKSALCFWT
AYLYQVFGYVSTSLVHVGCDCLVFNFVDRLCAHLKILEHRILQLPDLVEANACDEIRYLKS
CIEDHHSICEGIKELNDTFYETIFIQFVTSISVLCTNIYLLSMQDLFSAEFIAVFVYLCCAFVQ
NFFYCWYGYKVSVNTLHISDAIFNMNWCILKRESKKILSYVMMKTSQKVFLFNSAVVTLT
PESFVNILKVSYSAFNILQQTK
>NvitOR107
MEVMDTIKSTDIMPLPFFYLKLSGAWKPSSWPSYLRLIYDSYTILMTFFIMKVIIVTEILYVI
FAEENQSKVLKDNVYIICTFINGWFKMFNLICRRKNIANLVKGCIAKQWNPPRDNYESSVL
AATKQTSRKITLAHASVVGSCVVSTLLNSVLSSPPFLPVDAWYPCNITLPICFWTSFVHQSI
GYTVTAIVHVANDNIVVGFMMQICAQLNVLNRRLLLVHVEVEKAARQQKDQSQITSLETT
LVNDCIVNYRDILKFAEQLSETFIETIFIQFCAGLSVICTSVYVLTTLNIFSFEFFGMFLYLWC
MLGQMFLYCWFGNEVVLNSSKLFHSIYNMDWIKLQSQTQTKLLFMMLVASSPIQLFRGAI
IRVNLDAFINILKFSYSAFNILH
>NvitOR110
MDDDILCVRSLQINSVSSKIRKMKEMNPAKSVDVLSTSFLYFKLIGAWRPLNLPKWLRVIY
DLFTISMVILMYEMLIVTEILAIIFAEENRLKVFQDIVHITITHVSGCFKMLFVINRRQSIMLL
VNGCVAKQWYPPRNELEATILTKHNNLSRRITLTYATLVGASLLAAVLNPILYSTRVLTIAT
WYPCNISLPICYWSSYAHQTMGILAMAIAHVATDSLIVGFTIKICTQLNVLNQRLLSINFQL
ENTSARCQKSQEQSLALEAILVNECIVNYKDILRFADLLSRTFIEIVFIQFCVGLTVICSTVYL
LAKLSIFSYDFFGLFLYLGCMLMQMFLFCWYGNEVVLDSTKLFHTIYNINWIELQIQTQSK
LLLMMLVASSPIQLFRGAIIKVNLDAFINILKFSYSAFNLLQKSS
>NvitOR111
MDEIIDPIKRTDVLPISFLYLKLVGAWKPLDLPKCLRLIYDLFTIFMVIFICKLLIISDILCVVF
AEENRFAVFKGIVHVTITHLSGWIKMLHVLSRRRSIMLLVNGCVAKQWNPPRDRHEASILT
SFDNSSRRTTIAYTIQVSAAVSMLVLSPVFSSTWFLPIDNWYPCNISSPICFWPSYVHQSMGI
VAIAVAHVATDTLIVGFMIQICTQLNILNHRLLSIHIKLEDTARRQKNQEQISAVETLLVNECI

SHYIDILKFADLLSKTFVEVVFIQFCVGFSVICSIVYLLAILSILSFDFFGTLFYLGGMLSQMF
IYCWYGNEVVLNSTKLFHTIYNMNWVAFQIKTQKKLLLMMLVALSPIQLFEGAIIKVNLD
AFINVLKFSYSAFNILQKSS
>NvitOR113
MMSEIFPTTYFFLKISGFWRPYSLKLPLYLCYQIYTAFSFATVLSLIILLVLYCAFAHDKFLEL
LLENMYLVISFSNCISKTSNIILRKKNVEKLLQWIREKRWLAERDLEESCIVAHSKLMEKAI
PQFCTLLVCANGIGNLMNPIIRANPDKKLVIEAYPICDRSRPVCFWLTYLHQCFGFVIVNVI
HVACDCLIYNFIDRTCAHLKILGHRLQKLPVLVKGIRHQGIDTVEFEKSYVIDCIKNHQGIFI
FIRELNDTFCETVFFQFLSSILVLCTNIFLLSKQELFSPEFIAVFSYFCCVLAQNFFYCWYGY
KLSVNSLAFVDAIAKINWVELDMKTKKMLVYMMLITSNKVELFNNAVVNLSPASFINIVK
ISYSAFTVLQRTSHKEMKI
>NvitOR114
MLRTEELLANSRANERRNERVGIEDQVFPATFLLLKAAGVWTPTTLKLRSQYMCYRIYSA
FCFISVLALVVTVSIENVVSSNASILESWYMLVIFSHGLLKIKNLQWRRVKVIHLLKECIMN
ERWSIARNQDERAIINESKRAEKFITHLWLSLLLVNGLGNALNPLIHENPNNSLIFECYSPC
DRSLPSCFWTAYAYQLFGYAISSTVHVGCDCLIFNFIERINAHTMIFIDRLQKLPSRVVEGKN
EGCLDASRHEARLLKECIQDHRRIYESVEELNNTFYEVVTIQFLTTISIVCTNIYFLSKQELF
SADFIGVLVFLVCVLTQNFIFCWYGYKLSESSSYIVNAIFNMDWLVLNKRSKGLLLFAMMS
ASNEIKIFHNALVNLSPETFLQFVKMSYSAFNLLQQSN
>NvitOR115
MHQAIKIANGRIDADGIPNVEGLEKRVFPRTFLLLIVGGMWAPTTIKSRALFACYQLYTVF
CFVSVCMLIITILIDNVLSDDKTMESLVEYAYMLIVFSNGLVRIINLVSRRDKILRLLQGNIM
LDRWQSLRDDEELAIIAESKVSEKLVLKIWGSLILMNGISNAVNPIIHENPDNTLMFECYSP
CDRSVSYCFWMTYSYQLFGYVIMSVAHLGVDCLIYNIIDQINSHYKIFLNRLLKLPARVRE
KARDDVAAALRYENNYIKECVADHHSIYKAAGELNDIFNELVFIQFISCISLLCTNIYFLSKQ
ELFSPPFIAVFAFLCCALTQNFFFCLFGNKLSQTGSEIAGAIFGMDWQELQKETRRKLLFIM
LLTSKGIALFNNAVVNLSPETFLKLVKVSYSAFNLLNQSTHK
>NvitOR117
MDLLPVHFRTFQFFGLWYNDPCSYRLIKLVHRSLIVLLIVHLSLFQMIALFSAKRNVDEYT

NTLFLALTYFVHIYKTLVFMAKNRSVNEMLDEFRSDICRTRGPEEEHILAKHVQRANWAY
SGRMILTLFAGSIRVVLPILIGFSTGKLELLPFDTYFFNVKHLVQYALVYVLQTLAIITVIVTD
VCLDSTPCACMILACAQLEICRHRIKHDNMVLYENSEDGPGRGFNEEMALKEYVKHYVLI
QEAVHRIQSVFIAIVLPIFSSALLTLCTSIFQLAQKNHTTGEYCFIILYLCCLLVQTFSLCWFG
NELQSKGEIVTSAVYETDWTVLKPCLKKSYRYLMFMGQNKFIISFHGQCTLTLQTFIWMIK
TSYGAFNLLKQVADT
>NvitOR118
MDLLPMHFRTFRFFGLWYDDPRSYGLAKLVHRSLVVILIVHMSLFQMIALFSVKHSVDEY
TNTLFIALTYFVNIYKILAFMAKNRSVNEMLDKFRLDVCRTRDAEEERILAQYLHTANWT
YSARMILTLCTGVIQIVVPFLIGYFTGKVGLLPFDTFFFNVEDLAQYALVYALQAVAIVTVVI
TDVTLDSTPCACMILACAQLEICRYRIKHDNNVIANEVTGDGEMNSECKPGKELREKMAL
KKYVKHYVLMREIVDRIQSVFISIVLPIFCSALLTLCFSTFQLAQKNQTTGEYCFIITYLCCL
LVQIFCLCWFGNELQFKGEIVSNAVYETDWTVMKPRIKKSYWYLMFMGQNKFIISFHGQC
TLTLQTFIWMIKASYGAFNLLNQVADTKY
>NvitOR119
MDLLPMHFRTFQFFGLWYSDWRSYRFFKLVHRSLELLLIVHVCLLQIIALFSVKHSVEEYT
NSLFIGLTHFANIYKTVVFMVKNQSINEMLDKFRLDICRARGREEEQILAKYLHKANWTY
SARMILTLFCGSISIVVPILVGIFTGKLELLPLDTYFFNVNDLKQWTLAYVLQSLTVITVVVT
DVCLDSTPCAFMILACAQLEICRHRIKHDNMASHEIGKDVPRKGYKEEMALKEYVKHYV
LIREVVYQIQSVFISIILPIFCSALLTLCTSIFELAQVIVFLFNYHTTGEYCFIMSYLCCLLVQIF
CLCWFGNELQLKGEIVSNAIYETDWTVMKPCTKRDYWYLMFMGQNKFIISFHGQCTLTL
QTFIWMIKTSYGAFNLLTQVADT
>NvitOR122
MDVLPLNFRTLWLCGIWHEENEKLTVPRIAYRFLVICLMFYFTFTLSAVVFVENSNVSELTE
AIFLAVTYITLCLKIVNFAFRRAEMIEILHDFRHPYCKAEHSEESEILKGYSKQARKMYIYL
MAFVMSDVAYFWSTFAFKVSKNIMELPYHTYQFYNMSSKAILFSTAALQATSVLYSVSINI
SFDTMTAGLLILTTGQLELNAHRLSKLGEHNVDSMNGYIAHNVLINGTVDKIESFIKTVVIP
FLFFSLLSICASVFQLSEYSVFSLEFLGLFSFAICILLQVLVYCWFGNELMLKSEAVTDAIYR
SDWTMLSPQNRKSLQVMMICNKDGRTVSFGGQCSLTLETFVWILKTSYATISLLNRVSA

>NvitOR124
MDILPLNFRILRYCGIWYELPEHLWLVKIVYKIFVVVVIFSFTLSELIELALTYDDLQNLTEC
LFLTLTFLALCFKMINFMCRQESLKALLNTFRDEICQPKTLEEKDIIEKNRSMLRLFCISYFS
LGILSGSTLVFVPFASFKSSKIELPIKTYQPYDVEDFVLFSLTYFHQILSMYLGVLINVSLDM
LVCGFIFLTCGQLDLCYYRIVSSNMYTMNNNIRHHAVTKDIVKKMQSFSIVVVVPLFIFSLI
TLCTSLFLMPEKEIMSFEFITLFIYLTCMLTQIFLYCWFGNELQLKSKTISDAVYHSNWTRLT
PKLRRNLLFTMFISQNGLMISFHGQCSLSINTYVSILKTSYAAFNLLRKTSNTLGV
>NvitOR125
MDMLPSFRVLAYLGIWIEEGSSFVFLRRLCGLFLSNTIFYFTLTEVIELYLLRNNIEELVDVM
FLTVTFAMLCLKILNFNFRHKGLLNLLTDFRMDVCKARSPEEENILNKYTTKILNIFQNILV
LSQATGIFFCVLPFITLEPADYEIPYKTYQFYDDTTAMGFTITCVIQFIALIFGIFINVSMDTMI
YGFIILSTGQFELISYRINKSSKENDRALLKQCIMHHNCMNNLVKKTTNLFMTVIAPLFFFS
LLTLCASIFQMSQNDIISLEFLGFAMYLSCMLCQVFLYCWYGNELKLKSADLVNEVFGSD
WTVLEYTEKKTLYLLMLSAQRPCDISWRGQCTLSLETFVWIMKTSYTAFNLLQRASDK
>NvitOR126
MDVLPLNFRSLQYCGIWYEFPEHLWLIKTVYRTFIVVVIFSFTLSELIELALTYDDLQNLTE
CLFLALTFLALCFKMINFMCRQESLKALLNTFRDEICQPKTLEEKGILAKYQNILKKVFIFY
MSLGLMSGSSLLIVPLVSMENSRISTLPMKAYQPYDVEDTILLNITYFYQVFSTWIGIIINVS
LDLMVCGFIILICGQLDLCCYRILCTKTKMFHDNNVRHHAVIAEVVRRVKSFFIVVIVPLFI
FSLITLCTSLFQMPEKEVLSLEFFSLFMYLSCMLFQIFIYCWFGNELQLKSKTIVDAVYQSD
WTDLTPKLRRHLLFTMFISQNGLTISFHGQCSLSINTYVSILKTSYGVFNLLQKTSNI
>NvitOR128
MDVLHLNFRVLQYCGIWYEYPENLWLVKMVYKTLIVVMLFCFTLSELTELVLNRNNVHD
LTECLFLSLTFLTCCFKMINFLCRQEGLNRILNAYRADVFQPKTTEEKQMTTQYQNLISKFF
MVYLIMALLSGICLSLIPIISSASNETQFPAKSYQPYNTQDSTLYLITYFHQILSIFFGIFINVS
MDMLVCGFIILACCQLDLCCYRISLNKKDTSTNDHVVHHVLIGHAVNRVQSFFIVIIVLLFI
FNLIVLCTSLFQIPQKNIMTLEFFTLFVYLVGILFQVYVYCWFGNQLQLKSKTISDAIYESN
WPDLTPCKRKDYIFSMFMSQNGFTISFHGQCSLSIKTYVWIVKTSYAVYNLLQNTST
>NvitOR129

MEIYDSRYFVHAKRFQELLGIWPYQSRLKNNCSWVILSFLFIAMIIPQIVGLSVHAGKDSK
RTLECTFGTCYMLAIYMKLLVACADKDKAKFIFEYTARNFKKINDNDERKILIEYSERGRLI
GVVYTIFVLAALGVFVVVPLAPGILDVILPLENGTRSKFFILNGEFLVDKTEYFIEIYAFDSIC
CIVTVLIICATDPLYAAILEHCLAIFAIVKLRLRKYRVRKGLKCVSADEAEYEAIIRAVQLHR
EIIAFIETIQNNCSLYFAFEMGVTLISFTVNFVLAVLKTPDLFDRLRLAMVLFAQAVHLFYIT
WPGQKLIDHGEDLFKETYFNDWYKSSVKCQKALRFMSLRCSKPCKLSGAGVYVLNFAYT
LLILKTSASYITVVAQFEYKIVA
>NvitOR130
MEIYDSRYFVFNKRFQMALGIWPYQSRVKNSITYAGLVLVMIIMLIPQFIRLNTYLGKDIEK
TMENIFIFFYVFGIFVKLFTAHFAEDKLKILYESTAKNFETYTDAVEAEIMKRYSERGRLLTF
VFLLYMISAVAVSVVLPMCPIVLDSTDPLDQPRPRMFILNGEYIVDKYEYYFQIYTLDIISVF
LMICILCATDPMYAAIVEHCLGLFSICKYRLRNFNKSCGLQMVEHAEAERYGGDYAYAAL
VRAILLHKDIIKFTEIIQTSYSLYFLLEMGATIGILTSSSVVVVMKLKQPLELLRWSLFLFGVI
LHIFFLTWPGQKLIDFSSDIFQEAYLNDWYKSSLKCQNLLKFMSLRCSRPCELSGGGLYIM
NFINFATILKTSASYITVFSSV
>NvitOR132
MEIYDSRYFVYNKRFQTALGVWPYQSRVKNAIICGFLLLVMIALVVPQIIRLKMYIGKDKD
KSMENVFGLFYIFAIYVKLFTAVYAEDRLKILYESTARNFQIYTDKMEKKILHENSERGRLI
TLVFIMYMMTALIVFILLPMYPIMTDVIVPLDHPRARMFILNGDYLVDRDEYYFQIYVFEST
SAALTVFILCSTDPMYAAIVEHCLGLFCICKYRLNNFNKPRRTEIIEKANAESQVDEYAYTA
LVEAIQLHKNILKYTKIIQTSYSLYFLLEMGATMGLLTSTSIIIVMKLYRPLDCIRYFLVLIGL
LLHIFFLSWPGQKLINVSGDIFQDTYHNDWYESSLRCQRLLRFMSLNCSKPCQLSGGGLY
VMNFVNFARILKTSASYITVFSSF
>NvitOR133
MEIYDSRYFIHNKRFQKALGVWPYQSRAKNIVVCGLLLLLMLGMLLPQIVRLKKYAGKD
SDKMMENIFILFYIFGIYIKLFTAVYAENRLKVLYESTAKNFQIYTGEAERRILYEYSERGRL
LTLAFIVYMLPAVTVYVMLPMCPIIMDAAKPLDHPRYRMFILNGDYLVDEYDYYFYIYAF
DSMAAIVTVAIMCATDPMYAAIVEHCLGLFSICKLRLKNFNKPNGTKAIEKTYYYSETCGD
EYAYAALVKVVQLHKDIFKYTEIMQASYSLYFLLEMGVTMGVVVCNSVIIVMKLSQPLEL

VRWSLVLIGGLLHIFFLTWPGQKLINFSGDIFQDTYLNDWYESSLRCQKLLKFMSLRCLKP
CELSGGGLYVMNFINFATILKTSASYITVFSSF
>NvitOR134
MEIYDSKYFIHNKRFQMALGVWPYQNRVKNLSICGVLLLVMFGMLIPQLLRLRTYLGKDI
DKSMENIFILLYTFGIYIKLFTAHIAENKMKILYESTAKNFETYTDEAEKKIMKQYSERGRLI
TLAFLIYMVLALILFVMLPLYPIIMDATIPLDLPRPRISVLNGDYLVDENDYYFQIYVFDSIA
CTLTVFIMCSTDPMYAAIVEHCLGLFSICKYRLKNFNKSCGMRMVERADAERYGGDYAYA
ALVRAILLHKEILKYTQIIQTSYSLYFLLEMGVTVGILTATSVIIVMKLERPLDCLRYFLVFIG
LLVHIFYLTWPGQKLIDFSGDIFQDTYLNDWYKSSLKCQNLLRFMSLRCSRPCELSGCGLY
VMNLINFAAILKSSASYITVFSSV
>NvitOR135
MELFDSRYFIINNTCMKLLGIWPYSSHVKNYLRRCGLGLFLLSCYLPQFIPLYMYFGEDMD
QMIQNIGVILYVFGTSVKLITGVTAKDRMKIVYEKTARDFQTIVDKEERNILFEYSERGRTL
SITFIIYMWIALAIYVGLPMGPLVLDYFIPLQNGSRERGFVWKGEYLVDPDKYYLTIYAVEL
FSSVLSVTILSSVGPMYQAIVEHCLGLFVIVKFRLQICTRGGKKAEEESYRLIVKIIRLHNDII
EFTRIIEASYTSYFFIEMDITISLVTLISVNLISRLDYLFDSIRHIFILLGVMIHMFYLTWPSQK
MINHSTDLFHDTYSNEWYNCSIRCQNLLKFVALRCVEPSQLTARGLYVMNFENYASLVKT
SASYITVLLSFR
>NvitOR137
MDIFDRRYFVLNKALLRSTGLWPYEDRRKKLYIRTFVNLILGICVIFPQIVRIYNYFGVNMN
MVLEHAAVLMYITSIYLKFLTSVYYEEKLRVVYDNIAKNWQVIKDENEINILIQYSENGRL
LTIGYTMYIIAAFCSYVFLPIVPVLLDVFNPLNQTRSRFYILGGEYFIINNVEDYGKVYAFDC
LAVIVTVWLISAVDSMYAASIEHCLGLFAIVKLRLQTCTRSICDGQKDECYKMIVRLIRMH
KDIINFTDILESSYSSSFLILVGINVIFLSFECIIVLTRFGQAMEMMRYSMIMVGIVVHLFYIS
WPGQKLIDFSLGLFQDAYLNEWYTCPTRAQKLLGLMTLRCSKPCQLTAGGMYVMNFSNF
AKIVKTSMSYMTVLASFR
>NvitOR139
MEIFDQHYFSVNKALLKSTGLWPYESRRRKFCIRTFINLILGVFVIFPQLVRIYNYFGVNMD
MVLEHAAILLFILTTYLKFLTSVYYEEKLKVVYDNIAKNWQAIKDENEVNILSQYSESGWF

LTISYIMYIVIAASAYSLLPMAPVLLDMIDPLNETRPRLYILGGEYFIVDNVEDYGKVYAFEL
VPAAVTVWLICAVDSMYAASIEHCLGLLAIVKLRLQMCTQPSCDSRKDVSYRLIVQLIRLH
KDIINFTDILESSYSSSFLILVGVNVLFLSFECIIVLTRFGQTMELIRYSMIMVGIVVHLFYLS
WPGQKLTDLSIGLFQDAYLNEWYTCSTRAQKLLNLMILRCSKPCQLTAGGIYVMNFSNFA
KIVKTSMSYMTVFASFR
>NvitOR140
MEIYDSRYFIINKTLMTKMGLWPYQHPLKKFLVRTFLVVFIFVSSMPQLYGLKKNFGVHM
DKIIEHLALLMYIYGIKLKLVTSILSEKKLKKVYENIMENWQQIKDVHERAILVEYSERGRT
LTIGYIMYMTSALLFFIILPITPMVLNVIKPLNESRPWDFIMHGEFPVNDMHAHYGEIYLFD
SLACIATVLVFCTVDSMYATCIEHCIGLFAIVKSRLDLSTKFVNRQGALGIKRDDKVYDLIV
KTIKLHKKIINFTHILESSYSTSFLILMGMNMLYCSLVSVLLIIKSDALMERIRYGTILLGLLI
HLFYISWPGQKIIDLSTGLFEDAYSNEWYETSIRSQNLLKFMRLRCLTPCQLTAGGIYVMNF
ANFASIIKTSTSYITVFASFT
>NvitOR141
MDIYDTYVLNINKKLLSFVGIWPYEEKKKNKFTRVFYLITMFCIIVPQMIGFYQHFGVDID
ELLENTGTIFFTLSIYTKLFTSIIFENKLKILYDSVAKNWKNITEKHEREILVKYSERGRMLTL
GYITYNFAAVIVYTTMPLMPFLLDIILPLNESRPSMFILNGQFYVDKHEHYKKLYAFDCLCI
FVIVPAALAVDTMYVACTEHCLGLFAIIKYRLAMSDKFISTRDIYLTEEKDSSYRWMIHTIR
MHIDILKFANILDKSYSSSFVILMLINTVYVSVLCVLVLISLDKPLNLIRYYMLLVAICIHLFY
LSWPGQKLIDHSEGLFRDAYNNQWYEGSAKSKTLLKILTLRCVEPCLITAGGLVTMNFATY
LTIMKKSVSFITVFSSFR
>NvitOR142
MTMDFYNSRYFSINRRMMTIMGLWPYQDFKTKLFIRTFLAIVLGIALIPQIISIVKYTNEDS
DKVIQGIATLLYVTGITLKILTTITSEKKIEIVYRNIVDNWKLLDDENEIRTMTEYSEFGRLLT
IGYVAYMFFALGLFVTMPMLPMMIDVISPINGSRPRIFILDGEYIADKNENYGKVYIFESLT
CIMSVFVFSTVDSTYAVCVEQCVGLMAVVRLRLKLATAKAARMKYKSDSDEHDIPYQLVS
SSAKLHIKAISFARILDSSYSVNFLLSMGSNVMILSVGSVVILINLGRPMEFIRYSMIFIGLMI
HMFYLSWPGQKVIDSSQGILYDAYNNEWYECSKKTKTLLKFMMLRCIEPCQLTAGGLYV
MNIANFGSLAKTSMSYITVFASFR

>NvitOR143
MDFLDSRYFILNKKMLHILGIWPYQKRLERYAIRSVYFFFMGVSFVPQILCVKKYFKVDSD
KFIRGVTTLLYLSGVSLKLTIAILMNGKIQIVYSKVADNWKMFTDKDEIKTLLEYSEVGRM
LTLGYVVYMVLAVIVFITMPYLPVVIDIVFPINGTRPRLFVLDGEYIVDKYENYNKIYIFES
VCSVVSVPIFCTIDSTYAVCVQQCVALLAIVKLRLKVATKYTKNYLRDHKYNDASQQLIIK
SADLHNKVIEFAQILETSYSMVFLLLMGMNCLILSVGTLVILVNLNNPLELSRYIMIFIGLM
MHMFYVSYPGQQLIDRSSAIFNDAYNNEWYECSIKSQRLLAFMMLRCTKPCELTAGGIYT
MNLENFGSLVKTSISYIAVFASFT
>NvitOR145
MDKLKQSTVDIDTINNIFGNTYFKINKELQELVGLWPYQKGFSVRVVQTIMLFVLSFIMIPH
LNGIRVWCGKDLGICSENIAATIYLSGCFLKYLVVLLCKRDISKVYEKIAINWLTINDPNER
VILDKFSSLGKLKSIGYTVYVSAAGIGFSQFALLPFAFDYFSPLQNGSRPKIRIVRAEFFVDPI
EYYWHIYATYCIVTFVSAFTIISIDTSYTAVVHQNLGIFNIVKYRLSLAKKAVGTSKDLAYE
QIISAVRLHQDSLGFNNLIEVTYRVCFLLLILVCISFLTFGAITILENSDNWIDIVRLGSIEVGA
VIHLFYLSWPGQLVVSESEELYYYTYNNEWYNLSAESKTLLHFMMLRCINPCCLTAAGLY
VMNFENYGAIIKSTVSYITVLSSFRE
>NvitOR146
MNGFETSSLNSKIINEVFDNTYFKINKILQELIGIWPYQKRFDALIKQFIVILILSMVAMPHIN
GIRVWCGKDLGLCAENLAGVTYASGVCTKYFVVTRSKDQMTKVYEKITSNWLTITDPDE
RVILNKFALLGKYKSIGYVGYITVAAICFSQLGLLPILIDVVLPLQNGTREKLRVVKAEFGV
DPYDYYWHIYGAYCAISVVSSGVLMAIDTSYTAVVHQNLAIFNIVKYRLTQAKRAVNTVK
DVAYEQIISAIRLHQDSLEFNNLIEHTYDVSFLILILICVTFLTFGAITIMEESDSYLDMFRLSL
LECGVCIHLFYLSWPGQLVVTESEDLYYYTYNNEWYNLSEKSKTLLKFMMLRCMKPCCL
TAAGLYVMNFENYGAIIKSTVSYITVVASFRED
>NvitOR147
MTNEISQEKLNRVFDSHYFYLNKKLQIVSGLWPYQSRKRKFIHKLTMLCFLGTALVFLLNG
LRHWCGVDIDVCGENLVGLIYVISVLSKLFITSLYEEKFKIIYTRLAINWLELTDPQEHNILIS
FARQAKIKTVVYFVYMAAAGVGFCQIPMIPVFLDFINPLNETRPKILFVKAEFIFDPYKYFY
QLYAFFIGCAASAVFIVCSIDTTFTAVVHQIIGVVSIIKYRLNCATVSFNPNKDVSYKLIVHAI

QLHKEVLQFSDLIEKSYNIFFLVLTGLTVVFLSTGAIVMLVRVGAMLDLIRLVLVLIGAILHF
LFLTWPGQNLIDHTSDLFAAIYATEWYNVSERSKKLLSIIMLRSLKPCVFTAGGLYVMNLE
NFGSIMKTAVSYMAVVSSFR
>NvitOR149
MNKEEVDEAFNDSLLKINKELNIFNGLWPHRPDGDKLFRRIIVLTVLISVTLPHVLGMFIQC
GRNMALCGENICGFCYCSGVIAKFIVPIVSKEKFITLYEKIALNWKEITDPYEQSILEEFSKL
GRLKSWLYFVYCAVAGFAFCQMTALPALMDIILPLNESRPKILVTKAEYPFDPFEYYYELYF
LYCTAAVVSVSVLASTDSTYSVIIHQSLGIFGIVNHRLQKAAKHKNQEESYRVMVSAIELH
KSALEFLELIESTYQSAFLIFIFVTVAFLSFGSLIIVEHSEEIIDLIRMTLIEFGAMIHIFFISWPG
QLVIDHSENLFLSTYTTKWYNMSKKGKMLLLFMMMRCLKPSFLTAGGFYIMNFENYGSI
VKTTLSYVTVALSFH
>NvitOR151
MDEREIDLLYDNYYFKLNKKLQIITGLWPYKSRKYKLGIRAVVYATLSLVMIPLCNGFRTW
CGVNLDICGENLVGICYTMLIFLKYWVTTHSEERLKNVYRLVAKNWMEITDPHEHEILVD
YAKQGRLKTIGYTAYVVVAGIGFCQIPMISVILDIIIPLNVSRTKILFKGGEFILDPYQHFYKL
YVYFVITSFVIMTIIIAIDTNYTIIIHQILGLLTIVKHRLQRLAIPMNLKKDNSYHAIIKAIHLH
NDALQFVDLIESSYSCLFLVFIGFTIIIISISTSIMMAQIGKLLNMIRVAMFVLGASLHFLYINW
TGQQMIDHSKELYLNVYSNEWYNLTKEAKTLLKIVMLRCLKPSKFTAGGLYTLNLESFGT
IMESALTYVAIMSSFR
>NvitOR153
MNKKEVDKAFNDSWLQINKELNVFNGLWPNRPNGDKIFRRFIVLTVLITVTLPHVLGIVM
QCGSNMALCGENVCGFCYCSGVIAKFIVPIVSKKKFVTLYEKIALNWKDITDPYEQSILEE
FSKLGRLKTWFYFVYCIVAGFAFCQMTALPALMDIIQPLNESRPKILVTKAEYPFNPFDYYY
ELYFIYCTAAVVSVSVLVSTDSTYSVIIHQSLGIFSIVNHRLQKAAQHKNPDESYRVMVSAI
KLHKSALQFLELIESTYQSVFLMFIFVTVAFLSFGSLIIVEHSEEIIDLIRMTMMELGAMIHIF
FISWPGQLVIDHSENLFLSTYTTKWYNMSMKGKILLQFMMMRCFKPSFLTAGGFYIMNFE
NYGSIVKTTLSYVTVALSFH
>NvitOR154
MDIYDSRYYKTNIFYLKLLGLWPFDDFLNKRVRRILIIIAVVSLIIPQVIRLFEEWGRDIDIVIE

VIGSLIYFSGCQIKYLSFLRVEAKMKYLYNKIAEHWKSLSSKDEIKTLEEYGEIGRGLTLGY
IIPINIILVIYISLPLLPLLLDVIDPQNETRPKQFPYFAEYFIDDQKYYFELTIHGWIVCILSVQI
YGTFDTTYTQCVQHACGLFGIVEQRLRKATKLASSNAFSTQEEKDEKVYDKVIDAILLHK
EAIQFVNLIEDCYSFSYFFVVTLNTAVVSLAAVDTMLNLENGNTKQMVRIGALYIGFSFHL
LYNMSPGQRVIDSSTNIQNAAFHCDWFNASSKTKTLIRIIMLRSLTPCQFTAGKLIVLHLESF
AFVFKNSISYVTVVGSMR
>NvitOR156
MSESKTLKIFESDYYRTYKNSVKLIGLWPHENIHKKRITRFFITALLTTFMILQGIRLYEELG
NDIDIVLELIGSIAYFSGCICKYLTTIKAQAALQFLYEQIQGHWDTITNKRERQILEQSASES
QFLSKFYMGASYVALVVYTASPVLVPIILDIALPLNESRRKTFPYFIEYFIDTEFYYYQLMV
HGTICFTISVLVYISIDTMYAACCQHLCGLFDIVEHRLKEAVKTNSNRINLEPDRTDILMHK
LLNEAITLHQDSIEFAVLIENTYALCYLLVLGLNLAVIVLAAVDIVINLDDTNQIIRLSILYIAF
SFHLFFNSVPGQKIHDKSVNVMNSAYFSEWYNLPLNARKLIQLIIHRSLNPCQFTAGGLFV
LNIENFGSIMKSSMSYITVLASIR
>NvitOR157
MDIFNSRFYRTNCFFLKLLGLWPLGDVSNNRIKRVTVVSLVVSLIIPMVIKLVQEWGNDIDI
VIEVIGSLIYLSGSQLKYISCASVQSQIKFLYTEIERHWNTLTNEEEKKILKQYARDGYNLSF
GYLMLLNVILVGYLLVPFTPMLLDLIDPLNETRPKAFPYFAEYFIDNQKYYFELTVHGWIIC
ILSVQIYGTFDATYTQLVQHSCALFAIVEYRLGQATKMVASDEDSSHKDTDKVAYNMMVG
AINYHKQAIQFVGLIEKCYSLLSFLIIILNTAVVSLAAVVTMLHIEKGNQKQAIRIGMLYVAF
SFHLLYNSYPGQKVIDSSTRIQEAAFHCEWFNTSSKTKQLIKIIMLRSMVPCTLTAKTLVVL
DLESFAFVFKKSISYITVIGSMR
>NvitOR158
MSESKRLEIFESDYYRMYTNALRLIGLWPFECTYRQRIIRFFIIILLITFTILQGIRLYEEFGQN
LDIVLELIGSITFFIGCILKYIVTIQTQSMFQFLYEQIQSHWEIVTNRERRILEQSANDSQFFTK
LYMGAAYGALIVYVSTPIIVPNVLDVVIPLNESRAKTFPYFIEYFIDTEVYYYQLMAHGTLC
FTISALVYVSIDTMYATCSQHLCSLFDVVEYRLEKASKTDSKMNVNLDLNGNDKNIYKLL
NEAIVLHQDSLEFALFIENTYASCFLPVLGLYLTSIVIVAVDIVINLGDMNQIIRLSILYFAFSF
HVFFNCVPGQKIHDKSVSIMNSAYFSEWYNLPLEAKKLIQIIIHRSSIPCKFTAGGIVVLNVE

NFIVIMKSTLSYITLLSSIR
>NvitOR159
MSVRKDMHVFESQYYRIYKNSVKIIGLWPYENIQIKRVIRISIILLLISLVILQAIRLYEELGR
DLDIVLELIASLSYFAGCLSKYITTIRAQSAFRFLYDLIAGHWQIITDIKEREILEESTRQSQT
LCLSYMVAAYSALVVYSTMPAIAPAVLDIVIPLNESRKKTFPYYAEYFIDDEAYYYQLMGH
GTIVFTVSVMVYVSIDTMYACCAQHLCGLFSIVEYRLQEALRTDDKLHLEPPERDKLTHK
KLHEAIILHKDSIEFAFLIENTYALCFLLVMGLNLTVIVFTAVVIIINLGDMKQMIRLTLLFGA
FSFHLFFNCVPGQKVHDKSISIMNSAYFSEWYNVSLKSRKLIKFVMHRSLNPCQFTAGGLF
VMNMENFGSIMKSSMSYVTVIASIR
>NvitOR160
MSVYSSDYWKMPVLAQKFMGVWPFNNRQYDKCMRVFVYVALYSLIVPIGIRLVEELGVN
TAIAIENLVGQMYLNAAVIKFSMTILFKEKHKQIYELIARDWKMTSDKEELEIMEKHAAIG
RTISLAYGICCCSTAGAFLMIPTLLPLLDYVAPLDNSSRPVVLPYYAEYYIDQRKYYLPLML
KALVAGMISMTVFITYDMAFAMCVQHVCSLFDIINLRLQRASQLGSQLGSQLGRSARTSAS
YDSGVFRLIQKAIELHQIVIENVSSLENAYNLNWFFILLLNTTAVGGALLVVLLKLGHPEDL
VRYGMFFAAIFIHKYFIFLPGQKIINYSLEVFEYSYSCEWYNLSAECKVLIKIMMLRSIRPLN
LTGGKMFLLCMETYSAMLKAGMSYFTVFASTQSF
>NvitOR161
MELFESNYWKLTVFLQKLIGLYYFQSLWKNIVAWIYVYTFTLSFIIAIGVRLYQEIGIDINIVT
ENLVAEMYLIIVFAKLTTSVVYMKDLKRLYKSIANDWRVMSDEKELKVMHEYTDIGRKSV
QLYSGYMIIGIAIFLSLPISAPLWDYIVPLENATRPNALPYYAEYGVDQEKYYFPLMGQAVF
GGIGTGMLLVTFDLGFILTVQHVVALFALVCYRLDQAANLSLSVERGKIDFIKADRSAYEY
TVKAINLHQTVLGYVDLVENCYNAGWLVVLFMNMLLCGGGLAVLLMKTDRPEELLRYF
TVLFAGFIHFYYIFLPGQKIINSSLEVFDKCYASRWYNLSEKSKSLIKIMMIRSLRRCELTGG
KMFILCMDTYCNMMKTGLSVFTVLR
>NvitOR162
MFDSKINTQDDFNLDIFETTDYKLYKDGMKLIGLWPFESSTKKTLKRAFVLISMISVLISIQI
RFVEELNQNIDIVLQSAGSEILSIGCIAKFVTTLRAEDSFRVLFIQIAKQWASITDETECKILA
DNVKLCHPLCTFYRVMAVFALSSYACLPSFGPVIMNILLPLNETRQKRIPAPAEYFVDEEKY

FYILFSHGMILYMLVCVLYVTIDSMYSCIVHHTVGLVGIVTYRLQNIIDLDITSSPKNHTNN
LEIRRRLRRAITLHKESIEFAENIEATYSLCFIIVMFVNLFSMVFTAACGIRTLHYDKVESFR
WLMLYGSIIFHLFFNSNPGQNLFDKTSEIINTLYFTAWYDSGISTSNKRIIQIMMIRCLRPCQL
TAGGLLVLNMFNFGAIVKTSFSYITMLLSVG
>NvitOR163
MFDSKINTQDDFNLDIFETTDYKLYKDGMKLIGLWPFESSTKKNIKRAFVLISMISVLIFIQI
RFVEELNQNIDIALQSAGTEILSIGCIAKFVTTLRAEDSFRVLFIQIAKQWASITDETECKILA
DNVKLCHPLCTFYRVMAAFVLSSYACLPSFGPVIMNILLPLNETRQKRIPAPAEYFVDEEK
YFYILFSHGMILYMLVSLLYVTIDSMYSCIVHHTVGLVGIVTYRLQNIIDLNITSSPKNHTNN
LEIRRRLRRAITLHKESIEFAENIEATYSLCFIIVIFVNLFSMVFTAACGIRTLHYNKVESFRW
LMLYGSIIFHLFFNSNPGQNLFDKTSEIINTLYFTAWYDSGISTSNKRIIQITMIRCLRPCQLTA
GGLLVLNMFNFGVIVKTSFSYITMLLSVG
>NvitOR166
MLSFKIQAKVNNGDVLGVGYWKLNKLMLKSVGLWPYQKSSTKMCIRTFIFIAIYSMMIPQ
IIRTFEEWGKNSEIVIENITGFLYFQVVITKYVTSCIAESNLQYLYVRITEDWNHFRDEGEQK
VLSHFASHGRFLTIGYSVYLYTAGIAFTTLPCLIPAVLDLIIPLNDSRQKVLCFYGEYFIDQRV
YYYELLLHTFVCVMCTIMLFTTIDAAYACCIEHVIGLFNIVDYRLNQAFNLVKDKYDTKSE
VMRSEIHKCVLRSIEVHNHSIEIVELIQTTYTTCFFFTTGISLICLSLGTVDMMLSVNNYINF
ARVFFAWCGIVIYFFYISMPGQRIIDASSDIFNSVYFSGWYDFPLKTQRLLKFMMMRCSVP
CQFTAGPLLVLNLENCGVILKTAMSYCTFVFAIS
>NvitOR167
MEEHEILNNEYFKVNRFLLKLTGLWPYQKRHVKLIIRILYICAIHSMMIPQVIRTVEEWGKD
FEIVLENIVGFIYLQCVLAKYIITFTAEPQLVFLYKKMAFDWTRYIEAEEQLSLQRAASNGQ
LMTIIYSVYVNFAGVGFATLPGTLPTILNIIAPLNESRPTKVLCFYAEYFIDQEEYYYQLLFQ
TFIGVMSTVFINATVDTLYVICAHHSDGLFNIVSYRFQKAFNKSQERYQVKSRNLVAAKNL
DEEIHEYVLTAINIHNESIEFINLIQSTYTLYFFIQMSLTIISLSLATVVAMMNLHDIINLIRIFFI
WCGIILNLAYISIAGQQIIDTSLQIFDSAYFCGWYNHPLKTQRLLKFIMLRCSRQCQITAGPM
LVINLESCSNILKSSLSYCTFMIAVS
>NvitOR168

MEHDVKKYFKYKRGIVFMLSASGVWPNYTSHPAAVRLFLNICSALASGCMFYCIVNFCL
NYATNINAFTSCLGLMIGFFSTFIKVIILPMQKEDLQSLNEGVSASYERNLRIVKFRHHLLA
HFPMFSRFFYLYSYSVGMSVLLLTIMPLLALRQGKYVRMYPQLVPFSYEPGGSLHWSIYAF
EVFCGFYLWSVTSGVDSVFGLYALHMVGELRLLNVRFQMLKSSNNYAKDLKSCVDSHIM
LMESRHKLQRIFGFLAIWLAITCAIALCALVFQALQAKHATIIRIIYLCGHCFLKLLQAYFYA
WYGNIIAIESDACQSAIYESQWPGSGDKRFMNDVLVVLSQTPMIFKAKQWMPLRLDMFS
KVVHTSVSYFFLLRTLDES
>NvitOR170
MELKVERKAKSGLREEDIFNNKYFILNKKLLALVGLWPYQDARLKRVVRILLVLCIYSMM
IPQMMKGIEECREKNPNPEIILENISGFFYFQGVTAKFLTAILTEDKLKYVYEEVMKDWKRF
TDKNEIAILCKFAHVGRVLTVVWSIYAAMSCLLFVTLPAVIPMILNIILTRNETFKKSLCIYCE
YYIDQDKYFFYIFLHHIIAGIATIFLTIGIDTSYVNCVQHVLALFNVSSYRLKVAFDTIHHSK
KNDYNLKTLENNVHSYVVSSIRLHQRSIKFVDTIQSAYNIVFFIVCALLLFGISIITVDLVWN
VHNPINLIRIACLWMGTIMYMFYSNWPGQKLIDSSNELFDAIYTCGWFEFPMKTKILIRFM
LLRSIDPCRLTAGPLLQMNFESCSLILRSAMSYFTVLVTTG
>NvitOR173
MDIFDGRYYKTSKWFLEFLGLWPFQSNRRRYVTCFIFVFMTATVVFPQVLLLIELKTSNFNI
LIENSLSIIFGFACLLKYGVTFASRSRLQTLLTQIASDWQRLTDKAEIDILSQYGEEGRYLVL
FYTVYVFLAWVTCNFVPFIPPLLDILLPLENGTHDLVYPFYADYVFFKQTDYHYESCLHVF
FVYFGTTSLFAGMDTIYVATVKHSCGLFAITWLETMARTGKSNRSNYSIKPNSVVHHDMV
EAIVMHNETIRFVELLEDSFSLCFLMVQCMIVAGLATLCFYMMRIYDKTFNMCQFSTFTV
GLVIHLLYLHWVGQKIIDSSDKVFYSTYYSDWYLISRNERQLTKIILARSLYPCQLTAGKISV
LSMETFGALMKTSMSYCTVLLSVS
>NvitOR175
MDIFDGAYYKSCKWFLSTLGLWPYQTDNRKRISAVIFFVVNISLAIPTLWLKSFTVTFENTV
SVMFAIGCCAKYVVTYTSSQRVRNKADELVRLFKQIASDWQRITDTTELSILTKYSEKGKF
LITFYQVYVWFGWTVYTLMPFIPYFLDKVSPLNESRPLLMPFYADYIVFDQADYHYTSCF
HIAFVYISSALLFCGVDATFVMSVQHTCGLFAIICHRLEGEKIKKESEYAQNVMKTLSEEEL
REVVIFHNNCITCSGLLEDSFNLSFLILNSMSVLGLALSGVYFIYIYEDYYKFVRIMAFFVG

LIIHLLYLNWVGQKIIDSSEDVFLAAYCSKWYVISTSARAFIKIIMVRALEPCRLTAGGLSTL
CMESFGILIKTSVSYFTVFLSVA
>NvitOR177
MDLFDGQYFKLNKIFLTICGLWPYQSKLRRRITFAMLASSTLLFIFTLVAGILSQSKFDFVNT
EETFIFIFYCSAGLLKCTILYNQQNKIKKLYERIATDWKQLTDTSERDILRSFLLEGRKLNFII
MISCSSAFIIYSCVDLLPRILKEKSEYHRPHSFPYYFRPMVINEKLYDLQVAVHITVIVFYAGF
AYMSAIATYISSVKHVCALYEIARYRLQNAVSYDKSNNSLLELVEDTSIVPKLVKVIDMHA
QALRGIQIIEKVFSADFFVLEASSLTALATDVYELKYCRANVRTFIRAKLLTPIVIIYLFFVNC
SGEQVIQACNDMRITAYYIDWYRTSSRARVFVLMIMRRTLNPKYLTAGTIVMIISIENFAAII
TTAWSIGTILLTT
>NvitOR179
MDSFDSQYFEINKRVLTICGLWPYQSKLGKRITFAMLASNTFLFNFTLIAGILTHSPGEFVNT
AETLVGLFFCSTGLLKCAILYNQQNKIKKLYERIATDLKKLTDNSERGILRSFLLEGRRQNF
LTMVYGVSAFIVCSCVEFLPRIFNEESEYHRPHSFPYYYRSMVIHEKFYDLQVAVHGTVIM
LYSGLTYMSAIATYISSVKHVCALYEIARYRLQNAIIYDKHNYPLQELTDDTSTIPKLIKVIN
MHKRALRVTKKIEKVFSADFFVMEASCLIALASGIFELNYFRGNVRAIIRPLLVMPIVTIYL
FFVNQSGQQVIQACNDIHTTAYNNDWYKASARVRIFVFMIMQRTLKPENLTAGSILILSIEN
FATILRAAWSFGTIMLTTLKHSPSRNEDA
>NvitOR180
MDLFYNQYFNINKHVSMICGLWPFQSQFGRRISYMIFAMSTFSMIFSLTAGIISQLNPDLLNI
LETCVALFFCVCGFLVCTILYNQKNQIKRLYERIAADWENLTDDLERDVLRTFLLEGRKLIF
ITLVYSFPAFSLFACITFLPRMFSEESTKLCLHSFPYYLESMVIDKNLCNLQVSLHYSVALGY
VGLSFLSVGATYICSVKHVCALYEIARLRLENATVRYGNYDPLGELTDETSIIHNLIEAIDM
HKNALRGIQIIEQVFSTGFFIIQIFGLSLLAILICELKYHEGEITEMIRFMLVLSVFVIYLFFMN
WSGEQVIQSCDDIQKTAYDIDWHRISSRTRIFVLMIMQRTLKPVHLTAGNMMILSIQNFGTI
LKSAWSFGTILLTTQKSV
>NvitOR181
MDLFDSQYFKINKLALTVYGLWPYQSEIGRIINHVIFVVTSFSMIFAMAAGIQSQVNAELK
NILETVVALVFCGAGLVKCTILYNQRNQIKKLYERIAADWEKLTDTSERDILRAFLLEGRHS

IVITIVYAVPAFCLFICVEFLPRIFSKESAKHRLHSFPYYYKSMVISENTYDLQVCVHLMVVI
IYVGFSYLCASATYISSVKHVCALYAIACQRLRNAIVYRKNSTPLKELIEDTSVIPNLIKVIE
MHKEALRGIQIIEQVFSAGFFVFEISALTTIAILIFDLNYHQGNPFQMMRVLLILSVFVLYLFF
MNWCGEQTIQSCNNVNAQAYNIEWYGISLKARVFVLMILRRTLKPIHLTAGTIMILSMENF
GTILKTAWSFGMILLTTQTSARNKDPNFFGY
>NvitOR182
MDLFDSQYFKINKRVLMICGLWPYQSILGRRIAFAMLANSIFLFVFTLIAGVISQSQLDIINT
EDTIIITFFCLLGLLKCTMFYNQQNKIKNLYECIATDWNKLTDSSEHNILRSFLLDGRKINFI
TMVFCSSAFMIYSCIDFLLRIFNKESEYQRQHSFPYYFKPMVIYEKLYDWQVALHVTVIVIY
SGLAYLSAIVTYISSVKHVCALYEIARHRLQNAIIACDKINHPLQKCIEDISLIPKLIKVIEMH
EQAVRGIRIIKKVFGADFFVLTVFCISALTIGTFELNFCRADIHSFIRVLLLMPIIMIYLFYVNY
SGEQVIQACDDMYTTAYNIDWYKTSSKTRIFVLMIMRRTLKSEYLTAGTMIMILSIKNFATII
KTAWSFGTLLLTTQKHRKNEDANFVAENTLI
>NvitOR183
MTLINSHYFKLNKLLLTFCGLWPYQTKLKRRINYTTFAIITLSMIFSLAGGIQSELNTGFMNI
SESIIALLFFSTGFLKCTIFYNQRNQLKILYEQTAHDLKKMTHHLERDILQAFLLEARNFNV
VSLVYSIPIYIVFAIATYLPQVFGFTNESAKYELHFFLYYKPMIIHESVQDLQVLIHATISTIYV
GSAYLCVSATYISSVKHVCALFEIARYRLKNVIVDHSNNNRRGLMKNASVISNLIKVIDIHE
KALRGVQRIDNVFNASLFILEVTALSAVTILIFHLNYHQGNFRQMTRYSTILSAFVSYLFFC
NWFGEQVIQSCNDIRETAYNVNWYNMSLRARMFVLMIMQRTLKPVHLTAGTVVILSMEN
FSAFLKTAWSFGTLLLTTQKPSPKENSIFFEY
>NvitOR187
MKLNGYDKFYYTLHKYVLTCIGLWPYQSRMSKRFFFITYGISSCSLIIALIAGLSEKWSTDP
VIILENMLGIIFLTSSTAESSILYMHESKIIEFYDKIKTDWKKLTNKKEIEILQMHTRKGQFVS
TAYILYGIPAFAIFGFVTFLPPILDPPSRVEYSHIFPYYFYCMIINEDFRYYQIVLHCMVSFSYA
SVSYLAVNCTFAKCVNHVCGIYAIICYRLQNAIEPTVVRGPFNKLKNSKLIRFNLIDVIAKH
REVIHGVDMIEQIFSTGFLVIEIAGFSGIALVIADILYNQKNAYQLFRIMVVTAIFLVYIFYIN
WMGEQIIQVSDDVRLTAYFIDWFTLSIEAQEIIHMIVWRSCKTNKLTAGSFVALSLENFLSM
LKTSWSVATVLLSAHRSQKNAHFTGYGITNSFTNSSST

>NvitOR188

MNIFDFPQRHLLTCLGLWPYQSKFTQRIFFTCAILSFFSLFVAMAAGLGEEWSTELVIIYETI
VALFVIFGGLAKCIVLFCRKHQMKSLYDQIRKDWQELTNEKEAAILQSFMLKGKAQIILYV
VCAIPGYFIFVALTYVPIISSEDASKDYSHTFPYYTDLLILSKRFRVYQVFIHAGLGIFCGGIT
YVAFMAMYITCVRHVCALYAIVRYRLENMVKSQDKLMDKLNDDEEVIPGLLEIICTHKRA
IKRVRLINRIFSRTFFMVEICLLICLALLIFDVKYNQHNVRLVIRMLMIALMFIVHVFCMNY
CGEQVIQFSTDVQYAAYFMEWYMISSRAQKILIMILCRSSNPDYLTAGNMALSLKNFASIV
RTSWSMATVLLTTQKVNRPSYSIS

>NvitOR189

MEEPDLDIPYMKLQKLLMNCCGLWPYNSRLINRLIYSFFLLILISTIVPLGLGLIEEANNDIV
TYFESLVSVVTMFGGVAQITMLRTIRNHLVKHLYKKITADWQTLKDAKEIKILSAFSFKGR
SLTFLYMLITMSSYVIYLMLIYIPLANDKATSWDYSKIFPYYSKHWIISERVRHLQVTLHGC
FGIFYGGVAYVVGMALYICCCKHVCGMYAIVGYRLKRLIISCEVTSSGKLRDDVLVYNLYA
IMDQHKEAIKGVHLLARLFSHSFFLIQLCLLVCLSFLIFGVQYNIYSHKGMVRMFPASIIFVA
HVFFMNYGGEQIIYYSSKIHTTTHFMQWYLLSVKSRRILLMLIRRSCKPEQLNAGTMTLSL
VNFTSIIKASWSMGTVLVSAHRKH

>NvitOR190

MPMEEPDSSIPYVKIQKFLMNCCGIWPYNSRLVNCLIYSFFVVFSFTTMTPLSLGLNEEAN
NDIVTYFETLVAVVAIFGGFAQITMLGIRNHLLKCLYRKISTDWRTLKDARETEILSAFSIEG
RSLTFLFMLITISSYVFYLLLTYIPLINDEVASSDYSEIFPYYSNDWIISDRMRHLQVILHGCF
GIFYGGAAYIIVMALYICSFKHICGMYAIVGYRLKRLVTSCKLTSSGELRDDDAVSKLYAIID
QHEEAIKGVRLLVRLFSRFFFIIELFLLICLAFLIFVLQYDIRSSKVIVRIFLASIILVTHVFFMN
YGGEQIIHYSSKIHTTTHFMQWYLLSVKCRRILLMVIQRSCQSEQLSAGIMTLSLENFTSIIR
ASWSMGTVLMSAHRKE

>NvitOR191

MDIFQSSYYIRCNRYLSFCGHWPYQSLRNRIRNFVLLMFLMSTILIPQIIKFWQLRHNIHVF
VAALPSMLYYCAFLFKNSFSMLQSKEIKKVLEKIKSDFQRYKDEDLKILHKYSGQANKINT
FYTVYMFMAVGGYSMLPLTLHVMDIALPKNESRLPTKPRLINYNIEAFDENIFFIIIHGVIV
DTAVIVFIIGFETLCFSFSYHVCALFVIVTNKIRDSIDERITSKHSEVDQDIFYRNFVKIVIMH

KDALDFVDTVETALSVLNLFAIGFAMMPLTITGFEFILSKGNVGEMARWSLFAFGEIVHLF
YYNWPGQKIRDHSLCVYQSCYAIEWYKEEIPDKCKKLLNLMMLRGQKPCSLTAGKVYIL
GLENFAAVMKVSMSYFTVLSSVM
>NvitOR192
MDILKSSYYIRCNKYLSFYGHWPYQNVIVKIRNQIVIMLLIMSIFLPQFMKMIEIRHYFHYF
ILSLPSLLYYTQFIAKNVFAFVGRKQIKNVLDKIQQDFQVYKGEDLAVLHEYSKKAQKFNK
FYTVYMFMVVGAYSMLPFTLYMLDTFVPLNYSRLPYKPRLVKYCITTFDDNILFIIIHGGIA
DMMAIVFVIGFDTLFLSFAYHICALFVIVTHKIRDAVNDEIDSQNSTINECSNLREDISYRNF
VKTITLHKYVLTFIDTIETAFSPLNVISIALAMVPLTITGFEVVMNKGNPGEMLRYAMYAIA
EMIHLFYYNWPGQKIRDHSMLIYEACYATNWYRKDFSVRSKKIMNLMMIRSQKPSYLTA
GKIYVLGLENFAAVMRVSMSYFTVLSSVT
>NvitOR193
MEDNVLDGPYYVYCKNYLSSFGTWPLQSYKKKVLLRTLMYLGCSSALIPHVTKAYELRN
HLEYFFLCIPSIIFYVQVLTKISCMILNEDKCKELIKQIKSDFQSYTGDNLRILNEYAEQARK
VNHVYIYYFMGTVVVYNTSAFVPLLLDLLVPLNETRPRPVLRLMKYNIQRIENNFFVTTL
HGFVLNILGMMLIMGFDTLLLNCSQHACALFQIVITELKDTIDKHKIEATSDTAKDTNSRD
VFYQEVVKVIIKHKHAIEFVDLVESIYAMANLLVIGITLGSITLAEFETVQHKDNHEIAFRYA
IFTSGELLHILFHNYPGQRIKDHSLMVYQSCYNCEWYREGITDECKKLLSFMMLRSQKPS
CLTGGGLYVLGLENYATILKASLSYFTFLSSV
>NvitOR194
MDIINGPDFVYSRICLRPFGLWPFQDPKSKLISRVITLMAVSTVLIPHIMKTYEFRNDFHILL
MCIPSLLYYAHYITKFLYIAFREEKVFRNVLERIKDDFVTFRGESLNHLTNYSEEARKFNTF
YMMYLCSTVVIYNVTAFIPHMLDFVFPLENATRPRHAARLVKYNIHQIDNNFYFVLIHGMI
FDVVAIAIIIGFDALFINCAQHACALFKIVVVELRKSTKLDEKMSNSASDLVTLQCRQDIFY
AKLVRTIIAHKHAIEFTDNLESTYALVNFLMIGIAVATITLTEFETIVHVNEVDIMCRFAFFSG
GELISMLYQNWPGQRIKDHSLRVHASCFECEWYREDVSYKSKRLLMFMMLKSEVPSALT
AGKLFILDLQNYVKIFKASLSYFAFLSSVAKVSSN
>NvitOR195
MKMKDNVLDGPYYVYSKNNLSPFGMWPLQSYKKKVLLRTLIYLGCNSVFIPHVCKAYE

VRNNFQYFFICIPSVIFYIHVMLKMACMILNEDKCKELFKQIKNDFEIYTGESLRILNKYAE
QARKVNRVYVYYFMGTIVAYNTLAFMPLFLDFLVPLNETRPRIITKHMKYNIKRIENNYFV
TTLHGYVFNILGMMVVVGFDSLLLSYTQHACALFQVVRNELKDTIDKHEIEVTSHAAKD
ANSRDVFYQEVVKVIIKHKHAIEFADLVESTYAVTNLLVIGITLGFITLVEFETVQHKDNRA
LGIRFAIITIVELLHILFHNYPGQRIKDHSLRVHQSCYDCEWYREGITDECKKLLSFMMLRS
QKPSCLTGGGLYVLGMENYATILKASLSYFTFLSSV
>NvitOR196
MAYAEEPRENILESSTFLYSKSSLRVFGLWPYQEPKQRLICRTSTAVLIGSLLIPTICIVLEQW
RNFDYVVLGLPSFLYYVEFVTKYMYLAANQKKLEQIFGHIKNDFDTRKDRKLEILKDYAT
ETRLFNHIYAAYLIIVVVMFNLSFYQPHFLDLIMPLNESRPRPIPRLARYYVTSLDESFNFVV
LHGLVIDWYSMIFFLGHDTLLVNCAQHACALFKIVINDIQDCLVVQKNDKADDEDQFYRR
ISNTIDLHKWALEYTAMVDKMYMYVNLCIIGVSLLAITLSQYQTAIHLDNTDLVIRYSFFSI
GELVHILYFNWPGQRIRDHSLSIYQACYNCEWYRDDISYRCKKLLKLMMARSQLPSNLSA
GKLYVLGYENFAQVLKASLSFFTVLLSVN
>NvitOR198
MDKERNMDRMNFYYIYSRISMLSLGLWPYQSWSSMMTLRSLWIIQHISIMLPELIKIYENR
GHFNLLIESLPPFTYNIVMAIKFTNGVLNQRKLKSILEKIKYDWNKFTDKKEIEMLCYYSH
RGKSLNTVYIGLVAVVLLSYMLLPMLPAVLDLINPLNESRPKSPLYMVEFYIDQDKYFYSV
LTHAYITSLAGVLPLFATDLLFSNCAHHACGIIKILGRRIENILSEEPALKRSYKVDDEKKAI
ACVIEHQNIIKYCESINSLYTTSFFLILSISIGLMSVTGFVTLIKMNEEFKDCIRFAMFTFAQIF
HQFCYYFLGQSVLNHEEKLKDYVSNFNWYKASPKTKFIIKFMIMRTLKPTKIRAMIFPLTL
ENFTSLMKTTMSYFTVIKSTR
>NvitOR201
MDKERNMKFYFVYTRLSMLCLGIWPYQSWSSMLTLRSLWVIQHISILLPEGIKIYKNRKNL
NSIIDGLPPFIYNVVIAIKFINGIINQHKIKSILEKIKNDWNQLSEKKEIEMLRDYSDTGKAFN
TVYLSLVTVILLSYMLIPMLPAALDLVNPLNESRPTSPLYLVELYIDQDKYFYSVLTHAYITS
LAGILPLFAIDSLFSSCAHHACGMIEILGGRLENIINEEASIKEIDNNEEEKNAIACVIEHRGV
IKYCESINSLYSTSFFFVLSFAIVMMSVTGFVAVIKMGEEFKDSIRFAMFTFAQIFHMFCYYF
LGEIVLHHEEKLKDYASNLNWYKASPKTKYIIKFMIMRALKPTTMRAIIFPLTLENFTTLM

KTTMSYFTVIKSTR
>NvitOR202
MEQHYSIRTYFKLNRVFMVSSGVWPYQPLHVARIIRLLWITQHISIMTPEIIKLIEVRGMAD
LLLECIPSVFYNIVIAVLYGTTIHHQRKIKELIEKIQKNWITISKKSEVEILTRYSNMGIRIGWL
YIGALYFTLFIFCLFPLSPIVMDYVNPLNVSRQRLPLYRVQFFVDDKKYYWTILMHAYTTT
MIGIIPLLTVDLFLANCTQHICGMMLILGKRLEKTMETTKLVVNKLDDNIYKDIRKCTILHT
EILDFIEDINYIFSTAFGILLAVLTFLTSFTGIVVLIKWGDWNEVIRFGMFTMAELFHAFCYS
YHSQDVIDHNNQIHKSIMNSGWYKSSMRTRVLVQMMFLRSNKPCLINCIIFPLSMENFTTI
LKTMFSYFTVVKSCRF
>NvitOR203
MDGKQCLQTYFIVNKVFLFSCGTWPYQHTIFAKTFRYFWITQQIVIMAAKSIKLYEIKNDT
DLVIEAVASFFYNISITIKFVNQVINEHKVKIILEKIQDDWKSLEDDSEIKILSYYARLGKLFN
FMYIGAVYSALISYMVLPLTPIILDFIVPLNESRPKQPLIMAEFFIDQDKYFYPLMIHAYLSVL
YGIIPLLGTDTLYMNCVHHSCGMLKILGNRIRNILNSSSRELSNKIKYEKMVKCIIQHQNIIE
FCNNINETYSTSFLIVLCFSITLMSFSGVATVIKLGDNFNDVIRFGFFSVAQIFHLLCYNYMG
QNVLNYGEELRAQIYNTNWYEASLKTQRLVKFMMAKNMHPIILRANIVPLCLPNFTRVIK
TSMSYFTVLQSTR
>NvitOR204
MEKENDCIRAYYKLNKFSMTLLGHWPYQSENSVKIVTFLWIFQHLSILLPELIKFVEIRNN
VDYVILAFSPIIYNIVVGIKFVNGSLNRHKIKITLDTIQSDWKSLRTEEEARILANYSSFGKL
CTVGWAWICTTTIICYLLFPFTPFVLDLIRPLNETRPRQLIYMVEFFIDEDKYFYEIQIHSYAT
TLIGFIPLISIDTFYAASVQHACGMFAILGHRLRRINGAMSKSKKRSDEDAYREIVSCAIQHD
KILQYCDNLNDTYTDSFFYIMGCNMISLSFCGVLLILMWGRIYDMLRNGIFTFAQIFHLFY
YSFQGQVLSDRSLMISDCVYDSEWYTASLRTRKIMTMVSMRSLKPFLLTAKVYVMSLPNF
TLVIKTSMSYFTVLKSSR
>NvitOR207
MIFSFKDKFFPYYAWQTVTVLEKAAGWWPFQNRTKNMTLRLMHFGNLLLIIIMCSVRLFQ
EYKQKKLYIVVENTVILVMIICTKMKVIMFFINEKQRKIFYENVLIHWKDTNDEEEMMILK
QYAKLGLKTIINYAISVNILFQGVPVLSDVINYLNDPNITYLKKELPIYIEVYIDQEKYFYQL

YVVLFFMTCAALLLAFSHELTFFQSVQHINAMFKIIEIRIIRLSKIVKRTECGLDTFKEADRK
IFVCISRAVDLHNAVLNNIKFINSSFGATYLVVLLFNCLIFGASLFLIFNNSDQKIHLIRYGLV
FLGLSVHFFIIFWPGQKIMDGSESLFNVCCSCDWYKLSKRSKNLLRIMMLKSVMQCQITAS
GMFVLSFETYVKLFKTGLSFVAVFS
>NvitOR216
MTISETDLAVFDGPHYSLNKKLLIMFGLWPTLSRTRKVICLIFFTMIDLSLYASFADGINYYR
KQKKWMYVIEDTISIIYLSVTWIKYVTSYIFESRIKLIYEQIAADWKSLIDEEEIQILNNYSAF
ARLLTVLYVFYAIIATTLFYVLPFLTIVIDRIKPLENGTRFRAQPYHQHYFDLIDNEKYYYH
MYIGHGYVVSIIVTVAVMAIDTMYAANIQHACGLFAIVRHRLSKIGILNGEREYEFRVVDD
KKVYESIRAVCEMHKNSIKIVELIWDSFSISFLIFMGCSLVGMGMLMFNYIFNMIHPIEKVV
GTGLLLGIAILLFYMNWIAQQLTNSSDEIFIAVYSNRWYNLSIKGQKLIYSLLQSNANSVTL
RAGGIAEMNLQQFAAILKTAMSYATVMMSMNG
>NvitOR217
MGIPDPENPLAIFYTDYYKYNRKLLEICGLWPELSRPRKIIMMILFALLMTSLIIPMGAGAIH
YFHKGRIMYVVEDLIGLLYLTVASSKYFTYSVFEGRILRLYHQVGEDWRTTTDEEERKILQ
EYSEFARLLSIIYFVYAAIGAFYFNMSPYLPLGLDRWMPLESNESRVRIRTYHPYYFDLIDA
EKYYYECYFFHGNSVVYVSTMVGLSVDSMTAFNVQHICALFHIVGHRLRKIGSTLEINAK
GEKIARVDDITVVRQIKHVCGMHRTSIDSVELLQSSFGMNWLVFLIGTVTGIALLMFDLIFS
MKHPLEKMTGLVIFIGIQILIFYINWIAQKLTDSTEQIFLAACETCWYNLSVKGQKLVYFM
MQKNIIPLTLTAGGIAELNFQQFASVSKTSMSYAMVILQMNDE
>NvitOR218
MPVETKDQRKSYDFLSISSSHLTFMRLSSFLPLKGKSFFHPLSLLLQLWDHFIVIGYNVMW
QGYGIRMIQRGDVEVDFICEDIITVGFTIRYLLLCAKREKLCCLVESCEKLWDLLKDGEVIF
VRQFARKGYYFRNFILINAMLMAALYSVTAPFVRLPPIEANGTERKILPFRFFMDIQKEPAY
SIVFAFQSILLQFIDLMIVSTETVSLYLIMMACGYLRSVRNRLLSFKGNDDNTSEKGEAALK
FVIDCAHFHQQIMIFCEDIERMTRTLFFFACFCPIYNVSITGIVLFNNDEDKYKFLPLLCYNF
FQFFLCQWAPEHLAVESEDIALAAYSASLRPQAPSHREKINRILYFMMMRAQKPVQLTAGG
FVDLSIETFGAMTKSAFSFFMVLRKFRS
>NvitOR219

MSTKAEDSHTFQSISASHLNYLRLCSLLPWQGKGFSHPFSLLFQLWNHLAIFGFNAMWHG
YGIRMLQRGDVEIDLICEEITVLDITARYFLLLIKREKLGRHIETCRKLWSYLKAGEDMFVS
QFERKGYYLRNFVMINSVMVTTAFIITATFVRLPPLEANGTERRMLPARFFMDVQEDPAYS
IVFASQSILLLSVDVMIGSTQTVSLYPIMMACGYLRSVRNRLLSLEGSDNGTDAKGEATFK
FVVDCAHFHQQIIIFCEDIERMTRMLFFFACFCPIYNVSIAGIVILNSNEDKIKFVLLLVYNFF
LFFLCQWAPEHLTVESRAIAEAAYFASLQPLASSYREKINWILYFMVVRAQKPIQLTAGGFA
PLSIQTFGAMTKSAFSFFMVLRNFRT
>NvitOR221
MRGAMSAEPKDLRESFTFLSISSSHLIFMRMFSYLPLKGKSFSHPLSRLLQLWNHFAVFGF
NAMWQGYGIRMIQRGDVEVDFLCEDIITIGFTIRYIVMRIKREQLCRLVESCEKLWDLLED
GEAVFVRKFERKGYYFRTFILCNALLMAGSYSIAAPFVRLPPLEANGTERKILPFRFFMDV
QEEPAYSIVFVLQSIALQFLDFMMVMTETISLYLIMMACGYLRSVRNRLLNLKGSDSDPSE
KGEAALKAVVGCAHFHQQIMIYCEDLSKMTETLFLISCFCPIYNVSVTCLVILNTEEDNLK
FVPLMLYNFFQFFLCQWAPEHLTVESDNIAEAAYFASLQPQAPSHREKINRILYYMMMRA
QKPVQLTAGGFAPLSIKTFGAMTKNAFSFFMVLRNFKN
>NvitOR222
MTTKTKDGYTFLSISSSHLIFLRLSSFLPLKDKSFSHPLSILLQLWDHFVVMVYNSMWTGY
GIRMILRGEMEIDFICENVVVMGFTVWYIVIQMKRQQFCSLVKFCEKLWSYLEVGEEVVV
RQFERKGHYFRNFMLFNLLLMCTLFITTAHFIKLPPLEANGTERKILPFRFFMDVQEEPAYS
AMYTLQFFVCYFVVFMIASAETVSLYLIMMACGYLRSVRNRLLSLEGNDDDTGEKGEAA
FKLVVGCAYFHQQILIFCKDIERMTRTLFLFACFCPIYNASITGIVLLNNDEDKFKFILNLFY
NFFQFFLCQWAPEYLSEESEVIAEAAYFASLQPLASSHRQKINRILFFMMMRAQKPVQLTA
GGFVKLSIETFGAMSKNAFSFFMVLQNFRS
>NvitOR224
MPGAMSAETKNTFLSISSSHLIFLRLASFLPLRSKSFSHPLSLLLQLWDHWSVLAGNMMW
SGYGIRMTLRGEMEIDFICEDIIMVGFTMRYILLATKRKKLCHLVESCEKLWDYLEIGEDAL
VRQFERRGYYYRNFMMLNLLLMCTLYIVTAHFATLPPLEANGTERRMLPFKFFMDVQEEP
AYSIAFVSQSVVTYFICFMFVSTETVPLYLILMACGYLRSVRDRLLSIEGSDDDTSERGEVA
FKFVAGCAHFHQQIMIFCEDIKHTMRTIFLFACFCPIYNLSITGIKLLENDEDKFKFIVILVYN

FFQFFLCQWAPEYLIEESEDIAAAAYSASLQPQALSHREKINGILYFMMMRAQKPMQLTAG
GFVRLSVETFGAMTKNAFSFFMVLRNFSS
>NvitOR225
MSTEMEDPHESYTFLSISSSHLIFMQLSSFLPLKNKRFTHPLSLLLQLWGHFVVFASNVFWT
GYGIHMVMHGEVEVDFICEEIVVLDFTARYILLIVNREQLCCLVKSCGRLCSYLEAGEDIF
VRQFERKVYYFRNFVIINSLLVSTVFDVTAYFTRLPALEANGTERRMLPARFFMDVQEQPA
YVVTFVMQVILDYYLDFLIASTGAAPFYLIMMACGYLRSLRNRLLNFKGGDYDTSEQGE
AALDTVIGCAHFHQQMMIFCKNIERMTQTLFLFACFCPIYNVSITGIAILNSDEDILKFTPLL
VYNFIQFFICQWASEYLAEESEAIAEAAYFASLQPQVPSHRERINRILYFMMMRAQKPVQLT
AGGLVNLSIQTFGAMTKSAFSFFMVLRD
>NvitOR226
MSAKKKVQKEGDTFLSLSWSHILFLRVASFLPLKGKSFSHPLSLLLQLWDHINVIAFTSLW
QGYGYRMIKRGEMEIDFICENIITIGFTLRYIILCLNRELLCHLVESCEKLWDLLEDGETVFV
RQFERKGYNFRNFFFGNLMFMATLYTITAAFVKLPPMEPNGTETRMLPFRFFMDVQENPG
YAAAFVFQDVVVFYTDVIFASAETVPLYLVLMACGYLRAVRNRLLKIEGNDNDSSEKGEA
ALKVVVGCAHFHQQIMIYCEEIGQMTKTLFLVSCFAPIYNVSIAGIKLLENDEDKFKFIVIL
VYNYFQFFICQWAPEYLTEESESIAVAAYSASLRPQAPSHRQKINGILYFMIMRAQKPVQLT
AGGFVNLSIQTFGAMTKSAFSFFTVLRNFSG
>NvitOR227
MPTGMDSLTTTFQSISSSHLMFLQLSLFLPLESKSFSQRLISNLLQLWNHLMVIVYNVSYAG
YGIGMALRRDIEIDYICEQIVVETFSARYILLCFKRAQLRRLIESCKRLWGYLEVGEDIVVR
QFERKGHFFRHFLILSSLMAVTSYVVTAHFLRLPPLEANGTERKMLPFRFFMDVQEGPAFN
AMYALQIINSYYLVFMFASVETVSLYLIMMACGYLRSLQDRLLSLITEMNEDDLSKNGEAT
FNVVMGCAHFHQKIMIFCKDVDQMTRTLFLFACFCPIYNMSITGIKLLESDEDKFKYASLL
FVNLFQFFSCQWAPEFLIIESEAIATAAYFASLQPFAPSHREKINRILYFMMMRAQKPIQLTA
GGFIKLSIETFGAMVKSAFSFFAVLRSFRT
>NvitOR229
MLIEKKIESFTFLSISSSHLTFLRMAAFLPLDNRSFHHPYSRLLQLYGHICIFIYNTMWTGYG
YRMISRREFEIDYICEQMVVEGVCLRYIVLCAKREQLCALVESCKRLWSYLRSGEDVIVRQ

FERKAYFFRNFMLINSILVVMLFIGTACFVRLPPLEVIGTERKVLPFRFYVDVQEDPMFSAV
YALQAVVCTTISFVIASIETVSLYLIMMACGYLRSLRNRLLSLAENEDDAILAGETSFRLVV
GCAHFHQQIMIFCEEVDRMTRTLFLFACFCTIYNMSITGIKLLENDENKFKFGAILSLNLFQ
FFTCQWAPEFLIIESEAIGKAAYFASLQPMASSHRERINRILYFIMMRAQKPIQLTAGGFIKLS
IQTFGAMVKSAFSFFAVLRSFSST

>NvitOR230
MEREPIKYEDISRLYYRLFRTMGILPSSSSRRTTLLRVYFHVTIVLYYSMSMFDGLRMLGHN
DIEIEYVFEEVVIHGICARFLILSCRREELAELLLSCEKLWRMLKPGEDRVVKSYEKIARYL
AHYITWTTLVAIFFYIVAARIVKLPPAEVNGTERRMLPFRFYVDVQRQPWYDIVTVLEIVV
VLNIAMIVSTIETTGPFLITMACGYLRSIRNRLLAIADEAEGRGEISRLSTIRVVSCVKFHQKI
MRFCQDIEKLTSSVLLVQVVCTAYNISLVGFRILKNDPNAVKFVPLLLLNLLQLFTAQWIPE
HLLSESKAIANAAYSASLLHPEYEPRANRALLFVMLRANRPVQITAGGYMKLSLETFKRM
LTSALSFFTVLRSINDGAGDEGE

>NvitOR232
MRLHEINSFERVPASGTIRKFVEFRETDGSLRIFSPPHRGFTFGEPRLQLSLLQDASHLLRVY
WPTQQCSGVVEVDSIFVMVMAVSTIMRYYILVYHRFDFRDTMDACRVIWNDCTPNEHQI
VRWFERKTWMLFKLLAGSGMFINVFCSIGSIVVRLPPDEPNGTERRLLPYRWFIEDREYH
WLGYELIFGLQVLITHHLTVIAATVDTAGPLLMMISCGFFKALQERFFAAAARNEMILCKD
KLEFKQTIVSCSKFHQSVLVLCKKIEVMTRMIFMVQLICLGYNISLIGLKLTGTDPERFQYIP
NLVLCLCQLFITQWASDYLLEQSEEVATAAYFATLMSLDARIGGLLLTMVIRAQKPVQMTA
GGVIKLSIERFGSLITNAISFFMVLRNFTTQV

>NvitOR233
MRVHEINSFERALASDEIRKVAEFQESDGGLRIFPSTHRGVTFGKPRLQLSQLPDASHLLHV
HRTPHQRHGAVELDSVFVTVMATSTIVRYYILVYHRFEFRDAIDACRDIWEDCTPSEHQIV
RWFERKSWILFKLLAGSGLLINIFCSIGSIVVRLPPDEPNGTERRMLPYKWFIEDREYYWM
GYELIFGLQVLILHHLTVLTATVDTAGPLLMLLSCGFLKALQERFFAAAVSNEKFFIEDKLS
YQPLLTSCSKFHQNVLNLCRKIEVIMRMIFMVQLMCLGYNISLIGLKLAGNDPERFQYIPN
LVLCLCQLFITQWAADYLLEQSEGVATAAYFTTLMSLDPRIGGLLLTVITRAQKPVQITAGG
VINLSVERFGNLITNAISFFMVLRSFTA

>NvitOR236
MEETSAFYRRIRRIQTRVLRLAGLVPFENRTLIFAGTILMSIYVNFAFTAVSSVYIWAFFEDC
LNKRFNPDITSELFSFVGFHFRFMYIFSRRRKLGEMLGYAESLWERVRSEEKVHVRLFVRK
VSKLSVCYSGIILTTITLYVLSSQLPQLTAAATNETVHRVLPYPFYVDVQSSPRYEILLGAQI
VCLLTVTQTSVCVDTAIAFLIMIACGHFRLIQVRLGVIARHIEENEDKRKSQRSVGKNGEVI
EAEAEMDEEDFERTDDRVRERVKELVMHHQEILSFCDDIKNLSSEIFMIELISTTYNLSLIGI
LLAGNMPLAEKFKFAPVLFILTTQLFVCQYPPDLLIQESEAVANAAYFVPPFRRDRRRIDRIL
LSLLTRSQTPYQLRAGGQIPLSIESFGNMIRGAVSFFTVLRSFN
>NvitOR241
MELELLRYEAYTHNVIWFLKSAGLWPEAHPVPRKILSMVTLCSTFVVMVTVSNFSFQNVG
NVMVLTRGMSLAVSFSSAFSKVALFLLHHDDLVYLNKHLTGGFMRDMKEPENRPDLLNN
VKTFNRFMFTHAISVAIAMSMYSIGPLLALRKHGKYIRAFPAIYPFAYESGGLVHWILYALE
VSGAASLWTVTVGVDCVFGLYALQVCGELRILAKKFRELRATENYREKLHDCIQRHHVLI
NAKNKLDNIFGLISIWLAISGALVLCSLIFQVTELLKTNNSYLRAAHLCAYLLPKFLQIFTYA
WYGNLIAEESGACLDAMYGSHWTDSCDKNFKSDILIVLAQEPLALVAMGCMVIQLDMFT
KIVKTSVSYFFLLRTLNEENE
>NvitOR242
MIMEKEVEKYKKYKSNLKFMIVSNGVWPDYEKHPYCVRKFLNFCSISSISMTNYCMMLF
VIATTTDVRSFTSFFGLLLGGFGNLFKVCALTMNQKELHALNEGISASFERNLRVPENRPH
LLANFPMFSKFFNFLSYSTLGTIGFLTVIPLLHLRHGTYSRMWPILLPFSYEPGGTIHWIIFVF
ELVVSFFAWITTCGVDCLFGLYSLHIVGEMRLLSSRFQKLEWSENYRKDIRSCVKSHLLLL
KTLSQMQEAFGDLAVWFAFNSAASLCTLVFQFSQLTVMNPARVLYLLCHTCIKLVQAYSY
SWYGNIITVESEVCLNAAYNSHWPNHGDKHFMRDVLIILLQRPMVFKAKSFIALRLDLFA
RIANTTLSYFFLLQTLDEKV
>NvitOR243
MELELLRYRAYTHNVIWFLKSAGLWPEGHPVSRKIRSMVTLFSTFVVMVTVSNFSFQNVS
NVMVLTRGMSLAVSFSSAFSKVALFLLNYEDLVYLNEHLTGIFERDMKKPEYRPDLLKNV
KTFHRFMYTHVASLTFTLIMYVIGPLLALRKHGKYVRVFPAIYPFAYEPGGLVHWILYILEV
LGATCLWSVTSGVDCVFGVYALQVCGELRILAKKFKELGAIENYREKLNDCIRRHHVLIK

AKNKLDNIFGLISIWLAISGALVLCSLIFQITELIKAKSSYLRVVHLSVYLLPKFLQIFSYAWY
GNLIAEESTGCLEAMYDSHWTDSLDKNFKSDILIVLVQEPLTLIAMGCMVIQLDMFTKIVK
TSVSYFFLLRTLNEK
>NvitOR245
MDQKRFKYKAYERNVIWLLKSAGLWPEAHPVPRKILSLVTLFTSFVVMVTATNYSFQNV
GNVRMLTKGMSLAVSFSSVFSKIAFFILHQEDLLYLNKHLTGGFMRDMKRPENGPALLSN
VKTFNRFLYMHAVSVAIAMIMYSITPLLVLRKHGKYIRTFPSIYPFAYELGGLVHWIIYAVEV
SAAATLVTVSAGVDNLFGFYALQMCGELRMLAHRFRDLRAGNNYKDNLKDCIERHQVLI
NAKNKLEDIFGLITIWLAISGSLVLCSLIFQVSELIKNHVSYLRIAHVCAYLLPKFLQIFLYA
WCGNLIAEESKICLYAMYDSHWPDSHNTNSKRDILIVMSQEPLSVVAMGCMVIQLDMFAK
IVKTSVSYFFLLRTLSAENE
>NvitOR246
MEHDVKKYFKYKRGIVFMLSASGVWPNYTSHPAAVRLFLNICSALASGCMFYCIVNFCL
NYATNINAFTSCLGLMIGFFSTFIKVIILPMQKEDLQSLNEGVSASYERNLRIVKFRHHLLA
HFPMFSRFFYLYSYSVGMSVLLLTIMPLLALRQGKYVRMYPQLVPFSYEPGGSLHWSIYAF
EVFCGFYLWSVTSGVDSVFGLYALHMVGELRLLNVRFQMLKSSNNYAKDLKSCVDSHIM
LMESRHKLQRIFGFLAIWLAITCAIALCALVFQALQAKHATIIRIIYLCGHCFLKLLQAYFYA
WYGNIIAIESDACQSAIYESQWPGSGDKRFMNDVLVVLSQTPMIFKAKQWMPLRLDMFS
KVVHTSVSYFFLLRTLDES
>NvitOR247
MELELAKYKSYARHVITRLIFAGIWPESNKTIKTILYFISFTSTLTVSVTSINFGIQNANNVILL
TKGIGLASAFSSVFSKALLLPLHQEDIIFLKNRLTTKFMSDMETIEYRADLLSSVHVFSAFF
NMHEAMVAFAMFMYCFVPLYVLFKHGTYLRTYPCLYPFSYTPGGLVHWLIYALEVAGAIS
VWTITVGADCGFLMYALELCGEFKILARKFTELKAGDGYKRNLKECIERHHLIIEAKNRLE
DSYGLIVIWLALSGAFLLCSLIFQITELYDNHGSYVRIAHLCSHLVAKNLQIFMYAWYGNLI
ADESKAFLNAMYDSHWPEACDKNFKNDILIVLTQEPLVVVAKGCMYVQLDMFTKIVKTS
MSYFFLIQTLAN
>NvitOR248
MMDNEVASYVKYSSYLKRLTAFIGLWPDYQKQMPAISLLLSIQAAFSSFTTFCFIAYSCYLD

SADIGAFTSYIGGLVGYLTTVMKIFVLGIQQKNLKKLNNGISASFEANLKVPENRQYLLAH
LPMSLRFFYTYAITTGSSLALLVLIPLLLLRHGVYVRMLPLTLPFSYKPGGMVHWMFYLYE
ILCGWNLWTVAVGTDNLFSLYCLHIVGELKLLSSRFRNLKSSKNYRKDMKDCIQSHMLLM
KTFLKLQKVFGFVVMWFAITCALCLCSLVFQAVEMDKVSVMRVFYLFNHSFVKLLQAYL
YTWCGNIITVESEICLNAAYEAHWSDSGDKRFMKDILTVVLQRPMVFKANKFMELRMEL
FLKIVNTSVSYFFLLRTLDDDS
>NvitOR249
MESKVARYAKYKRDIKCLIVASGIWPHYEKHPHVLRKLLSFCSAFCSGSTFYCIVAFCFKYA
TNINIFTSCLGLMIGFFTTFIKIVILSMRQEDLQSLNEGVSKSFENNLKLPENQPHLLYHFPSF
SRFFYLYAYVVGISFVFLASTPLSIMLRYGKYVRMYPQLMPFAYEPGGSVHWAVFGFEMFT
GFYLWSVTIGVDSIFGLYALHMVGQLRLLGSRFQNLKSSSNYDKELGECVRSHIQLMKSR
HKLQRVFGFLAIWLAVTCAIALCSQVFQALHMRNTTPVRALYLFGHWFIKIVQAYSYSWY
GNIIAVESDLCLNSMYYSHWPGSGDKRFMADVLIILSQKPLVFKAKQLMELRLDMFLKIV
HTSLSYFFLLRTLDENPKAGT
>NvitOR250
MELELAKYKSYARHVIFRLIFAGLWPESNPKIKRMLSFVTFTSTLTVMVTAINFGIHNASNV
ILLTKGIGLASGFSSVFSKALMLPLHQEDIVFLKKRLTSKFMSDMETIEYRADLLSSVHVFS
AFVNMHEAMMAFAMSMYCFVPLYVLFKHGTYMRTYPCLYPFSYTPGGLAHWLIYALEV
AGAISVWTITIGADCGFVMYALELCGEFKILARKFTELKAGDDYKKKLKECIERHHLIIEA
KNRLEDAYGIIAIWLALSGAFLLCSLIFQITELYDNNGSYVRIAHLCSHLLAKYLQIFMYAW
YGNLIADESKAFLDAMYGSHWTEACDKRFKNDILIVLTQEPLIVVAKGCMYVQLDMFTKI
VKTAMSYFFLIQTLAS
>NvitOR251
MERDIQTYKVCSENVTLCLIFSGVWSATHPVLKKIAFFVTFFSTFSIMAHTLNFSLHNAQN
VRILVRGLAAASSFLSISSKAFLFLQHQNDLNYLKDYLTEKFMSDMKNPENLPDLLSNMR
MFAVFVTMYKTTIAFIMSMYCIVPLFSFLKYGKYLRVYPCLYPFSFVPGGVVHWLLYGWE
STGALSAWAISVGTDCAFGMYAIQICGEQRVLARKLKDLRVGSNYTRELRDCMERHHLIIT
AKNTFESLYGLISIWLAISGAIVLCSLIFQVTEYLENRGGYVRAIIFFAHFSGKMMQVFMYA
WYGNLINEESLAFPRAIYSSHWTDCCDTRFKNDILIVLAQRPLIVTALGCMNVQLDMFAKI

VKTSISYFFLLQTLKAKTEEK
>NvitOR252
MEEDIQTYKVCLQNVVICLIFSGVWPATRPLLKRIAFFVTFFSTFSIMAHTLNFSLHNAQNV
RILVRGLAAASSFLSISSKVFLFFQHQDDLVYLNDYLSKKFMSDMQNPENLPDLLSNVRTF
AVFVRMYKTTAAFIASMYSVVPIIAFLKYGKYLRVYPCLYPFSYAPGGVVHWLLYGWESA
GALSAWAITVGTDCIFGMYAIQICGEQRILARKLKDLRVGSNYKKQLRDCMERHHFIITVK
NKFEDLYGLISIWLAISGAIVLCSLIFQVTEYLENDGGHVRAIIFFAHFSSKMMQVFMYAW
YGNLINEESLAFPRAIYSSHWTDCCDTRFKNDILIVLAQRPLIVTALGCMNVQLDMFAKIV
QSSISYFFLLQTLKAKGEEK
>NvitOR253
MELEMAKYNSYSNTIIWSLICSGLWPKGHYVLKKILSCISFLSITTIMTTAINFSFQNARNV
QLMTKGMGTAVSFSSVFSKIVMVLYHQNDFIYLKKHLTTRFKRDLEQTENRQDLLFNVHI
FTKFVNTHEASMAFAMFMYCIGPILALYRHGKYVRTFPCLYPFHYEPGDVVHWVIYGLEV
TGATVIWFITIGVDCGFCMYALELCGEFKVLGRKFRELRVADDYKEKLRDCIERHHLIINA
KNRLEDAFGIMAIWLALSGAFLLCSLIFQITEILENHGSYLKIAHLCSHLLAKYLQIFMYAW
YGNLIADESQSFLYSMYSSHWIDACDKRFKSDILIVLVQEPLMLVAKGCMNIQLDMFLKIV
KTAMSYFFLLQTISSEE
>NvitOR255
MEVELKKYKRYYRDIKLLLVVSGIWPNFYPILDRVVSIVAAISTLLLTMALLNFCAHHVANI
MILTKSMGIAISFFSSFLKICIFLSHHDDLVYLNDYLTSSHTSDLSNPDDRSHLLEKFSSFSKF
FYTLTIAVALTFVLNTIAPFFALKRGKYLHIYPVIFPFDYEPGGSVYWSLISLELTAGFFVWS
VTSGVDSVFGLYALQMCGELRVLAKRFEELRATGDYRMRMRECMDRHHLLMRSRDILEK
VFGFLAIWLAVTSALVQCSLVFQAKVEFKTLSPFKIGFFFFYILMKLVQAFTYAWYGNLIAE
ESALCLNAMYNAHWPGSGDVRFMNDVLIVLSQKPLIFKAKSCMSLHMDVFTKIMNTAVS
YFFLLQTLDEGSVRHL
>NvitOR256
METELRKYERYSRDLKCLLVLSGIWPDFHPIIQPLLGCFAAFVCFVTVIAFLNFSIHHITNVV
VLTKSFGLVISFFSSFLKICVFLWHHDDLVYLKAALTDRFNTDNLNKSFRRFTLAKVNVFA
NLFYILTIAVGLTTGMAVVLLIISLRHGKYVMLYPSIFPFSYEPGSRVYWILLMVELFANLFV

WAVTSGVDSVFGWYTLQICGEFRVLAHKFQNLKSSENYRDDLKECVERHYVLMKTRDV
LQDVFGFLTILLALTSAIVQCMLVFQAIQVFKNLSLGMMVFLIAYITLKVVQAFIYAWYGQ
LIAEESEVCLGAIYNARWAGSGDTRFMSDVVIVLSQKPLIFRANGCMSLKMDIFIKILNTSV
SYFFLLQTLDEGSEHHQH
>NvitOR257
METELRKYERYSRDLKWLLVLSGVWPDFHPVIQPMLGCFAVLVCSLTAIAVLNFSIHHITNF
VVMTKSCSIAIGLCLSTLKLCACLWHHDDLVYLNTSLAASFNADNQNKSFRRFTLAKVNV
FANLFYILTIAVGLVIVMGLVFMILSLLHGKYVLVWPSIFPFSYEPGGWVYWILLTVQLLAN
FFAWTVPSGVDSVFGWYTLQICGEFRVLAHKFQNLKISENYQDDLKECLERHYALMKSGE
VLQDVFGFLAILVGLSSAIIQCMLIFQAIQVFQQLSFGMMILIFAFITLKHVQVFIYAWYGQL
IADESEDCIEAMYNAQWAGSGNIRFMRDVLVVLSQKPMIFRAKGCMLLKMDMFIKVLNT
SVSYFFLLQTLDEGLQN
>NvitOR258
METELRKYARYSRALKCLLVLSGVWPDFHPVIQPMLGCFAVFVCSLTAMATLNFSIHHITN
FVVMTKSLTIAIGLCLSTIKIVVCLRHHDGLVYLNSSLTASFDADNQNKSFRRFTLAKVNIF
ANLFYTLTIAVGLATGMGVVFLILALLHGKYVMVWPSIFPFSYEPGGRVYWILLLVELSAN
IFAWAVPSGVDSVFGWYTMQICGEFRVLAHKFQNLKTSENYQDDLKECLERHYALMKSG
EVLQDVFGFLAIMVAVTSAVIQCMLIFQAIQVFQQLSFGMMILIFAFITLKHVQAFIYAWYG
QLIADESEDCIEAMYCAQWAGSGDIRFMSDVLMVLSQKPMVFRAKGCMSLKMDMFIKV
LNTSVSYYFLLQTLDEGLQS
>NvitOR260
MELEVRKYENYSRDIKRLLIVSGIWPNFYPVLQRFVAVLAICCTAMTFMGAFNFCLEHVSN
VVVLTRGMGLLFTLLSTGMKICVFLHHQKDLIHLNQHLSARFLDDLQNKAYQSHVLARLP
AFSELFYSLTYTIGSTAFLTTILIPLLALRHGKYIQVCPSIFPFEYAPGGLVYWLLQLTEALAA
FFVWAVTSGVDSAFGLYTLQMCGELRILGSKFESLRVSDKYREELRECIERHHLLMKARDS
MEKTFGLLAIWLAVSSAVIQCTLVFQAMEVAKSMNPLRIGFFFLYIVLKLLQAFMYAWYG
NLIAEESAMCLNAIYNARWAGCGNSRFMTDVLIILSQKPLVFTAKGCVSLKMEIFSKIVNTS
VSYFFLLRTLDEGSQN
>NvitOR261

MEAKLAKYARYRNVVRRLLLLSGIWPHLEDTCRLYRVLTFSATFVIAALGAKVFAYCIDNI
AHVSLFAKGMSNAFSFYTSVLCYLVYRKDLVMLNDCLGRRFEDELKREDRRPLLLQSISV
YTRFMCIVAGLTATALVFYTLVPLVFIFKYKKLTQIYQGRYPFAVEPGGRVYWCVCFVESIS
VVFVWNVVCSVDNAFGLHSFRMCGLLRSLADRFAKLQPDDPGYIVELRDCVRTHQLVLR
AKEALQRVYGLVVLWTYVTSAIIMCSILYQADQAKKHMTVTRVIFFTSYITLKLLQSFTYA
YYGSLVSQESEKCQNAIYTSNWPGSGDLRLMKDVLIIQSQRPIVLRANGFFIVSMEMFEKI
VNTTISYFFLLQAVEEK
>NvitOR262
MMMELELLRYKAYTQNVIWFLKLAGLWPESHPVPKKILSTITLSSILVIVLTVSNFSFHNLG
NIMVFTSGMCMAASSTSAFSKVALFLLHREDVVYLNKHLSGGFMRDMDEPDNRPDLLSN
VKTFERFMVTHVISVAIAMFTYSVRPLLVLRKHGKYIRSFPAVYPFAYEPGGLVHWILYAVE
VSGTASLWTVTIGVDCVFGVYALQVCGELRILSRKFRELRADDNYKEKLKDCIRRHHVLI
NAKNKLENIYGLISILLITSTTLVLCSLVFQVSELMKTNSYLRTAHLCVYLIPKFLQIFTYAW
YGNLIAEESGACLDAMYGSHWTDSCDKNFKNDILIVLAQEPLALVAMGCMVLQLDLFAK
TVKTAVSYFFLLRTMNEGSE
>NvitOR263
MMELELLRYKAYTQNVIWFLKLVGLWPESDPLPKKILSTITLSSILVVVVTVSNFSFHNLSN
IVVFTSGMCMAASSTSAFSKIAMFLLHREDVVYLNKYLSGGFMRDMREPNNRPDLLNNV
KTFDRLMVTHVICVAIALFTYSIRPLLVLRKHGKYIRSFPAIYPFAYEPGGLVHWILYAVEVS
GTASLWTVTIGVDCIFGVYALQVCGELRVLSRKFRELRASDNYKEKLRDCIQRHHVLINA
KNKLDNIYGLISILLITSATLVLCSLVFQVSELIKTNSYLRVAHLCVYLIPKFLQIFTYAWYGN
LIAEESGACLDAMYGSHWTDACDKNFKNDILIVLAQEPFALVAMGCMVLQLDLFAKTVK
TAVSYFFLLQTLNEKNE
>NvitOR264
MKTKDESLQPNIFLQHYLNINSKMLRYMGLVVRTKGNKTDSKSKILERLPTYATNLISIIDA
FFQMRWIMDLWQRDNDLVMQITTSGISNIVCICKGFRLAYCREDIQTLFEKLATIWDQTCV
PEDIRDTIVKKAQSTLVFCRCYIVMMLGLGICFALPPMKNFLIQYFARKEMNHTYDYSERV
FLVRYPFEINSSSIYFSVLFEEQWVLFCSALYWVCCDTLFAQLTTHTSLHFEILQYDIEAVVN
RENDEDRLKQSMIDFVKRHRELLRICHMIEKLFSPVIFTTMLLTSINICVNVFELREMISEAK

LGDALLHGFHLVNIFFQLLVYCIFAERLTQQAGTIANATYNCKWTEKNNKLRIYLQILIMKS
QKPFHCTAYGFFPIDHKTITIIVNRALSFYMMLETTN
>NvitOR265
MKTEDKSAPLTPDFEDYTKINSLFLRCMGMGIGTDGNKKDRRSQIIERVPTALINVLCLLD
SVFQVQWVSELWKTDKKLVLQILTNALSNIVCLCKGFQLAYSREDLQTLFEDLAMIWRKR
IPHHEIRDEILRGAQKTLVFCRCYISMILVLGLCFGLPPLKYFILQFTDRNANRTYDYTERIF
LVRYPFDVNNLTAYNFIFMEELWVLYSAAIHWMCCDTLFVQLTSHTSLQLKLLHYDIEASG
NTEDERQFKENVMDIIKRHQELLRICDLIEDVFSPVLFVIMLLTAMTMCVNLFELREMLLE
AQYVGAILHSFHLINVIFQLLIYCVYAETLTEQAGSIAEAIYNCKWTENSHEVRTNLRMCI
MKSQKPFYCTAYGFFPIDHRRITYIFKTAMSYYMMLHQTTS
>NvitOR266
MKTEDSPSVTPAFEDYTKLNSLLFRCMGMGIGTDGNKRDKRSQIIERVPTVLINIICLLDFV
YQMQWINDIWKTDKKFVLQILTNALSNFVCLCKGFRLVYNREDLQTLFEDMAVIWRRRM
PRHEIRNEISREAQKTLVFCRFYVIMILFLSLSFCLPPLKYFILQFTDRNANRTYDYTERIFFV
RYPFEVNNLKVYNFLFIQELWVLYAAALHWMCCDTLFVQLTSHTSLQFKILHYDTETSDN
TKDERQSRKNIVDIIRRHQELLRICDAIEDVFSPIIFIIMLLSAITMCVNLFELQEMFLEAQYA
GIALQSFHFMSVFFQLLVYCDYAETLTEQAGSIAEAVYNSKWTENGHVLRMNLQMCIMKS
QKPFYCTAYGFFPIDHQRITTILKTAMSYYMMLYQTTS
>NvitOR267
MSQPTEDDLEYYFAFNLKLLALVGFKCSMDKKEKGLGFVNKLPSYIMCIQGTILSLFEVYL
LRDIYKDEDKTIVMQVLSQGVENTLNVCKGFFLAYSIERMENVLQEIKFLWNTYRPSPDN
RKIILAEAQQTYSYCKIYFCVLASCCTSYFLCYLPALFKLAQQYRDREANNYTYDFSQRLL
LLKYPFDIPSIPIYFLVELQEGFYLFYAAALFFVSGDTLFAQTVTHICLQFKILKFDIDAMFNP
ENTGEKDHLNLVTFIKRHRDLLRVCALIEEVFSPIILSMMLLSSIALCVDLVGIRGTMEKNN
YEETAVVITLMMLTLLQILFYCTFAEKITEETRSLADTMYGCNWTMKNNKLGLYIHLMILR
AQKPFQCTAYGFFPIGHSQLTTIINTAFSYYMMLQTTS
>NvitOR268
MLPPTEDDFEYYFAFNLKLLSLVGFKCSLEKNEQSLSFINKLPSYMMCMHGIILSMCEVYF
IRDIYSNENKTLVMQILSQGINNTLCISKGFFLAYSIERMQNVLQEIQFLWKTYRPSQDNRK

IILADAHRTFLFCKIYFCVLASCCSSYFLCYLPALFNLAQQYRNRDANNHTYDFSQRLVLL
KYPFEIPNIPTYFLIELEEGFYLFYSAALFFVSGDTLFAQTVTHICLQFKILKYDIDETFNSES
TGERDHSILVNFVKRHRDLLRICALIEEAFSPIILSMMLLSSLSLCVGLVGVRGTMAKHSYE
ETAVVVTLMMLTFLQILFYCTFAEKITEETRSLADAMYSCDWTVKNYKLGLYIQLIILRAQ
KPFQCTACGFFPIGHSQLMTIINTAFSYYMMLQTTS
>NvitOR269
MKTSVNQPTEDDLEYYIGFNLKLLSSIGLKCSLEKNLKSLGLINKLPTFIMCIHGLIFFIFEN
YFIRDIWSSDKTLAMQILSQEVSNIQCISKGFFLAFAIERMQNVFQEMQYLWKTYRPSQDN
RKKILLGAHQTFSFCKIYFFVLLSCSISYFLCLIPSLFNLAQQYRNREANNYTYDFSQRLGL
VKYPFEIPNIPTYLLIVFQEAFYLFYTAALFWVSGDTLFAQSVTHICLQFKILKYDIDATFNR
EDMRDHLSLVTIVKRHRDLLRICKLIEEVFSPIILSIMLLSSLNLCVNVVGIRGTIAKENYQE
TAINVTIFMLTFLQILFYCTFAEKISEETRSLADTIYNCDWTVKNYKLRFYIQLIIMRCQKPF
YCTAYGFFPIGHLQLTTVLNTAFSYYMMLQTMN
>NvitOR271
MKMTAINPEDYFGLNIKLMSLCGLRCSMTKTIGSFINKVPTFLANLVGIIYLVFQATFVMEA
VRLRDVALTSQILSQLVSNIQCITKGFLFAVSIEKMQSILYEIRSLWERYQPDIEIQESILDDAD
RTLNFCKYYVIANFSCVLAYALPLVLNLFMQYQARESTNHTYDLSQMILLVKYPFEVTKV
SRFIILVLLEEYLLVVSVIIWVSSDTLFAQTTTHICLQFKVLKQDIEKTFNYGGPNSKEILLKL
VHRHRELLRMCMLLEDVFSPIIFFTVFLSSVNMCVNVIGTRETISDKTYLNTGIYATILTMTI
FQILFFCIFAEKISEETTSLADMVYNLNWTAKDNQLGFYIYFIIVRAQRPFYCTAYRFFPIGH
QRLTSIIRASFSYYMMLQTTDNK
>NvitOR272
MKMTSINPEDYFGLNIKLMSLCGLRCSMTKTIGTFINKVPTFLANLVGIIYLVFEATFVIEAV
RLRDVALISQILSQLVSNIQCITKGFLFAVSIEKMQSILHEIRFLWQRYQPDEEIQESILDNAD
RTLNFCKYYVTANFSCVLAYALPLVLNLFMQYQARESTNHTYDLSQMILLVKYPFEVTKV
SRFIILVLLEEYLLVMNVIFWVSSDTLFAQTTTHICLQFKVLKQDIEKTFNYGGPNSKEILLK
LVHRHRELLRICMLLEDVFSPIIFFTVFLSSVNMCVNVIGTRETISNQTYFNTGIYATILTMTI
FQIFFFCIFAEKISEETTSLADMVYNLNWTAKDNQLGFYIYFIIVRAQRPFYCTAYGFFPIGH
QRLTSIIRASFSYYMMLQTTDKK

>NvitOR273
MQTNTEEKAITAVDAEYYFDLNIKLMSLIGLKCSMTETVTKFIYKIPTFLTNVLGIIYLIFQIS
YVREAVRSHDTSLAAQILSQTVCNIQCNSKGFLFVISIAKVQAILHEIRILWETYPPDDEIQK
SILLVADKTVTFCKYYVTANLSCVLAYALQMGLNFFMQYQAREATNHTYDFSHIILLVKYP
FVVTEIPTFITLFLSEEFLLIMGATLWAIIDTLFAQVTTHICLQFKILKRDIQEKFNTEGSNDK
EILLKLLRRHRNLLRICMMIEDIFSPIIFFTVILSSVNMCVNVIGARETIASKAYFETCIYASIF
LMTIFQIFFFCIFAEKLSDETTSIADTVYDLNWTTKDYKLRLYLRFIIVRAQKPFYCTAYGFF
PIGHQRLTAIIRASYSYYMMLQTTDGK
>NvitOR275
MQSKERDKPKVLDIEYYFDLNIRVMSLIGLRCDGPKITGFVHRIPTYTSNTIAILILIFEICLM
SDPVCSSNMELTIQTASQTVSNIQCVSKGFLFVNAIEKLQVVYNELQVLSQKYPLEDEIQVL
VFDIAEKTMNFCKYYAIAICSCILFYYTPIVVNVIVYILQDPSTNHTFDFTQTLFYLKYPFTI
KTFPIYSTIVSIEAVNLIAQGIFWFLGDTLFAQVTTHICIQFKILKHDIQKTFNDEGSKSKEILI
GLIKRHRQLISMCMLTEDIFSPVIFSVMILSSTNLCVNIIGASTAINDGDYMNAGVYATILLIT
VFQIFFYCIFAEKTTEETRSLADTVYHLNWAMKDHHIRLHILLIIMRAQKPFYCTAYGFFPI
GHQKLTSILSTAYSYYMMLRTTANV
>NvitOR276
MDYKMQTKEETIEVNAQYYFSLNLNLMSMIGLKCNMTENVGRFYHRIPTFITNVCALMY
QSMTVYYLVEAISAKNTSLSIQIISQLVSNIQCFTKGFFLAFGINKIQFILQEKQILWKKYPPN
NNNHHTILGIAQQTLTFCKFYVVAIFSCVMSYDVPLAINIFMQYLKRESTNYTYDLSRRVIL
VKYPFEVTEISTYVILCLQEALFVFIQCIFWVNSDTLFAQVTTHIGLQFKILKCDIEAAFNRD
DAKNKEILIELVNRHRELLRICMLIEDVFSPIIFCTVFLSSINICVNVIGVRETISEKAYLDTGI
YFTMLLITLFQIFFFCIFAEKLTEETRSLADAVYNLNWTIKDYKLRVYINLIIMRAQKPFYCT
AYGFFPIGHQKLTGIISTSYSYYMMLQTTDK
>NvitOR277
MKTLAKVQSTKDDIEYYFGFNLKLLSQIGLKFSMNEKTDKFTFLQKLPSYIFLVEGMILFIL
EVYLIRDTIQSDTLLSIQIMSQIISNLQSVSKGFLVLNKAKSIKNVLETLGIIWRKYPLNNSD
RALLNAPSKIISLSKIYWGIAVALLVIYDLPPFVIFFMQYQNRDAMNHTYDLSQTILLLKYPF
NITRKSTFFFLISQEAFVLYASGVYWIGSDALFAQFTTHICLQFKILKCNTKEVFNRGSKEA

HSSLIDLIKRHRELLKICEMTEEIYSPIIFSTMLFSAINMCVNVVGVRETITRGFYQETGVYL
FLFLVTFAQILLFCIFAERITEETKSLADLAYNLEWTKEDHKLRVYILFIILRAQKPFSCTAYG
FFPIGHKKLSSIINASFSYYMMLQTMS
>NvitOR278
MKSQENIFTKDDIEYYFDFIFKSLNTLDLKFSISKKTDEFKFRHKLPTIIGCLIGLIIFFLEIYFI
RDALHNHTILPIQIFSQVISNFQSISKVILIVYKVNKIQQILEKIGVLWKTYTPDEGNRAVLY
NTLQRTLSICKIYYAVLIATVLIYYVQPIVNFVGQYGARNSINHTYDYSQTLVIIKLPFKVTQ
KRYFFVISQEAYLLYMSGVYWGCSDTFFACFTTQICYHFKILKYHTKAFFDEKNNNSRLNL
VTLIKRHQELLRLCVLIEDVFSPIIFSTILFSAMNLCVNVIGVQETILNGSYRQAGIYLFLFIIT
FSQILFYCAFAEATMEEAWSLADLAYNLEWTSKDYKLRYYIHVIILRAQKPFHFTAYGFFPI
GIQKLTSIINASFSYYMMLQTVS
>NvitOR279
MKSRENIFTKDDIEYYLNFILKSLRTVGLKLSLSKKIDEFKFRHKLPTIIGCSIGIIIFFLQIYFI
GDALHNHTILPIQIISQVISNLQAVSKGFLVVYKINKIQRILEQIGVLWKMYTPDESNRATLY
NILQRTRSICKTYYAVLIATVSIYYLQPIANFMGQYGARNGINHTYDYTKTLLIIKVPFQVTL
KRYFFIISQEAVLLYMSALYWACSDAFFACFTTQICYHFKILKYHTKVCFDVKNENSRLNL
VTLIKRHQKLLRLCELTEDVFSPIIFSTMLSSAMNLCVNVIGVKETISNGSYRQTGMHLFLF
IITFSQILFYCAFAEAMTEEACTLADLAYNLEWTSKDYKLRYYIQVIILRAHKPIYCTAYGFF
PIGIQKLTSIINASFSYYMMLKTVS
>NvitOR280
MKTQENDLSIEDDIECNYSGYIFKAFHFMGLKLSLKKKTDGFKFVHKLPTTIGILQSIVVFF
LQMNFIRDVVQCDSNPPIQIISQVISNIQAGLKQTLLVFKKIEDIQRMLETLGEFWKKYSPD
KNYRVVLFRELGKTSSLCKYYFGTLVGIMIAYDVQPLVYFLTYYFEQNATNHTYDLSRRIL
LVKYPFEITRKSTYCFLLSQEAYLLYITAIYWANGDTLFAQFTTHICLQLKILKYETGKFFNQ
SNQEGRSDLLILIRRHQELLSMCDMIEDIFSPIIFSTMLLSAINMCVNVIGVTETIAAGSYEE
TGIYTFIFIATFLQIIFYCVFAETLTEETRSLSDFVYNLEWTSKDYRLRFLIQVIILRAQTPVYC
TAYGFFPIGHQKLTSIINASFSYYMMLQTVK
>NvitOR281
MKSQEDTKDDIEYYLGFILKSLHTAGLKLSISRKTDEFKFYHKLPTIIGCSIGIIIFFLQIYFIR

DALHNHTILPIQIISQVITNLQSISKGFLVVLKINKIQRILEQIGVLWKSYTPDESNRATLYSIL
QRTLSICKTYCAVLFVTLLIYYLQPIANFLVQYRERNGLNHTYDYTKTLLIIKVPFQVTLKR
YFFIISHEAVLLYTSALYWGVSDTFFACFTTQICYHFKILKYYTKVFFDVKNNNSRLNLVTL
IKRHQDLLRLCELTEDVFSPIIFSTMLSSAMNLCVNVIGVRETISNGSYRQTGMHLFLFVITF
IQILFYCTFAEAMTEEACTLADLAYNLEWTSKDYKLRCYIQVIILRAQKPIYCTAYGFFPIGI
QKLTSVINASFSYYMMLQTVS
>NvitOR283
MQIKVVENLTLTKHDIKYFFKENLKLLSKIGFKCSLTKKSEKFKFHHKIPTYIANFCGLIVFA
LQIYFVIDKIQTNTVLAMQSLSYAVINVQSILKGFMTANSIENIQQIFENLGIFWQKYMSRK
PGRELILDRAYKTISLCKFFFVMAIVCYFLFVMQFLIKFSIQYLNREATNHTYDFSNTVDLI
KYPFEIPNLPVYFLLISVEINYLFVCIVFWCNTDSLFVTLTSHVYVQFKALKLDTTLAFNNS
MLKERSILIDMVNRHRELLRMCYLIEDTYSPIIFSTTLLSALNMCVTVYAVREYIDKGYYLE
MGIPLFLFIGASLQILFYCIFAESLTDETRSVADSVYNLKWTTKDNKIKFYIQMIIMRCQKPF
YCTAYGFFPIGHQQLTSIISAAFSYYMMLQTMSN
>NvitOR285
MKIVGEKSSPNKDIEKYLGLNLKMLSCIGLDVSLENDDVIQERRILEKMPIFMTNGLGIFA
AILQISLITDSMTHNRMFLATQVSSHLFSNMLCISKGYQLATAIAKLGEILREIALIWKQNPL
NDEFHRNILSDAAKTLLFCKVFVVVTLCAVFGFGLPPLQNLFFQYLHARNSANHTYDYSQ
RVFIIEYPFPIQDVLTYSSVLLEEEYLLLASGLYWVCCDTLFAQLTTHISLQLEILQYDIETLI
NRESAEDRLNENFIIIVKRHRKLLSICELIESVFSPVILTTVVLSGMNICMNVFELSKTISEGN
YAEAALHAFLFMNTFLQIVFYCTFAEKLTEQTSFVANSIYNCKWTEKNCKFRVYLQMLIIR
SQNPFYFTAYGFFPIGHKRLTTVINTAFSYYMMLQTTS
>NvitOR286
MTLLHETVNHPPQNNDIREYLGLNLKMLSFIGLEFNLNNDRPIKKSKFMQILPIFMTNVVS
LTIAALEITFIAFVLRNHEEHLAVQICSELFSNILCIGKSLRMATAVASIQTALDEVSILWAKH
RPNQHCKMEIMKKARNTLNFSRWYLGFIITGIAGFALPPIHNFVYHYFIRDANNYTLAFSK
RIFLLRYPFEIKNVPLFFFVLTEEGYILLISAMHWVTCDTLFAQITTHTSIQLKILHYDIGALI
NHETVEHRLKAKILIIIRRHQCLLRVCRLIEDIFSPVILTTVVLLSALNICVNIFETKAMNAEGN
YARAALHANLVLVLFLQILFYCSFAEТLTNQTSAIAESVYNCKWTEKNHKLGFYLRMIMM

KSQSPFYCTAYGFFPIGHARMASIISTSFSYLMMLQSMS
>NvitOR288
MKKTILQEYDKENQKAFDEAKTLITWNKYLMSALGLWPSHRYDFIFVSLFCYYIFHFLLD
YAAFYFALRSFNLIKIIGATMENVTMAQIFLRLYTMRRYNRQYGEILEEFTRDFSVKNYKSE
EERNTFLSYNSRSKFFIKIVVIFLGVTAILYFTKPLIRQLSLSKNVNTTKAFTYDLPYRIHLLY
KITDIQTYIATYISRIPILYIIGFTQTAMDCLTLTVIAHLCGQLGVLSIRISNLDVVNKSNELNE
IIQRHQKLIKIGLRLRRMYRLCLLGHFLGATIAICILVYQVLISIAAGQKTNLVTFFVFGFLNI
FRLYTHCWVGEYLIHESINVSHAYYRCKWYKLPLKDQKSFIICIKRSQQPLSLMAGNFSHY
SLVMFTNVMKSAMAYLSFLRNFI
>NvitOR289
MMQNHQLQGQAELDDSSQVFRYNYILLTTLGLWPASLSDVRFFLNFGYFCYEMLLEYLD
LFLFIDNFENVLMNLTENMAFSQIFIRMLMLRIYNSELGEIIGDAKKDFDAKNYTEEERKTF
VAYHVKSRTFMKLLITNTALTASSYYVKPLLGQMGELMEYANSNGENSTFIFMLPYRFYTF
YELNDAQTYFWTYGSQLPFVFISGFGQSAADCLMVTLVYHVSGQMAVLALRIASIDTHPS
KCTQEVQKIVKAHIRLLRMGKVIQRTFSATLLGHLVGATSLVCILGYQILTSLANGERAILIS
FFAFIFLVLLVLYAHCTVGESLITESERVSQAYYDCEWYNMSKENARIIILCMARSQKPLQLT
AGKFSMFCLQTLTDSIKASMGYLSVLRTVM
>NvitOR291
MNISGSEQTILAKYKNDLQKASKILTWNRRLLSLLGLWPESPMDLLFCASAVYYIFYLGLIF
VSFVLYLKKKILNVSIFIALLSYGHISARLLLLRRHNRTFGVLFAEMKQDYELRNYKSDQE
LRVFLKYNILAKSMIKFLLFCSTFFAIVFYVKPLLMTYNIHRAIRKSHRNATAPFVLAQNSF
YQFYKITTVKKYAINYVSMLPFSVLTGFINCATDCLVLTIGCHLSGRLAALSHRIRNVEFCN
GSQEFKAVIRLHQQVLRIGDMVENSLNTLMTCHILTAGVIMCFILYKTLIYLRPGKRIHLIHI
VILLSLNIVRLYFHCCVGEFLMQESRVVHEAFFECKWYTMLLQDRKLIVLNLLRSQRPIRF
AARGLGTFSIELFSEVLKSSLGYLSVLRNVI
>NvitOR292
MNLYESDVNQKSLTKCKDDLKNASKILTWNKRLLLLLGLWPESPMDFLFCASAVYYIFYL
GLDFVSFVLFLRKKILNVSIFIQLLAYGHISARLLLLRRHNKTFGILFTEIKQDYELRNFESD
QELRMFLKYNRPAKTMIKLLFICSTFFGVVFYVRPFLTTYFVHRAIRKAHRNVTAPFFWNT

YFYKFHKITTINVYAMHYVSEFPFSILTGIISCATDCLVLTLGCHLSGRLAALSHRIRNVNFR
NGSQEFKAVIRLHQQVLRIAEMIEDSLSSLMLCHILVASILMCIVLYKTLICLRPGKRIHLINT
VILLFLNIVRLYSHCCVGEFLIQESRAVQAAFYECKWYTMPLQDRKLIILNLLRSQRPIRFT
AGSLGTFSIQLFSEVLKSSLGYLSVLRNIV
>NvitOR293
MEFVRKAYGDKQRKRQASSKCISRADRVFKRCVFFHKFVGIWLEKDRSQRLLDRLKGYV
SAAFTLGICIFQIVMLSVESSSVVTVLQNNLLILIRKTKEIAAPLHASKIKERKIVDRWLNNQ
DKILKILLTSYTFTFSSYSLFPLLKENGLPFTGRLPAICYVNPWYPTIFAAQLVFIIFRFFCVLS
NDILCITFLCQLCSELELVKHLIVELGNGKDRNVKQIIIRHAMVLDYGEIICETYSATLIMQH
LNCSIFLCLSGLVTMKTSDMFALLKIGSLSLIGIITMLIICFVGEMVMSSSLEIASTIESSVYK
DYRNDVANLKLLNFMLMRAQKPLCMMVCTQGKLSLRFFSENINKVASFFIYLKTLVE
>NvitOR294
MNIKSAESSLQSTTFEYEAIFHKVVGVWPGDDYFLARYSRIRGYVLAAFAVVVCVFQFTA
LLEANSDDVPENDFINLMRMTKEINEFSTLTDEEETIHIDWQSVQDKLMKIISRYYFLTVIG
LYFVAPMFRNALPLRGIVPEVLRVTPWFQMIYVLQCLLLLSNVITSISSDAFSVTFMCQLCK
QLELVQCSIKHLGSHTKVNLAETINRHAVALDYGQRVCNTLKTMFLLQHIFISMFLCFAGVI
VLNTQNSLILMKMIVISVIFVSTLLIICFVGETITSSSLKIASATESSNYEIFLGDVSTLRTVSFI
LCRAQKPLRMAVSLSGSMNLSFFTETMNKLVSAFMILRTMME
>NvitOR295
MGLIEVLESRKIFLWICGLWPKEYQHKPKLSQMKLYFIWFNMLMMCLLVFAGLVAIATPD
NIPQASIRLPRKRMMKVIMETLKLEETSKFENDNDLEIIRSWRRTDGVLKYHMRIYGFISVA
YCFLPIISQVNTYPAQTIIQASLFVSPWYEFFYGFHCAQLFLYLFIIIATDGLSMILIFKLCEEL
QRFECLLFERHRADDATLSEKYKSRGEFLRCIIRKHCTILDYGESICNLLTGALFTQYFLLS
GTLCFSVFTILSSNSSAMANQMSIMAGTCIVQLFMISLAGELVSTRSLALADALLQSDFCCS
IFGELKSSELRQTVLMQLRMQKPLKLSIGTLGVINIEFFSRIMKGVYSFTMLLRTSYV
>NvitOR296
MYVKLLSTREPHGGDASDGQLINTELRPKHTNLLYRTQAKIKMNVGEEYDKLALPMTLS
SRVVGSWPSRAELEGQGGRSVLVHRLHRYLAIVSIYLMSMGVAAEVIVFFGEDMNETIEC
ALISSAFFMALTRIITFASHQPEMLYVVETMREDWIRSTDEERAILRDKCLFAFKLAKFFAIS

VTITCSAFILMPMLELKFVENAKRMLPYRGYFFFNHTVPGVYEYVYLVNSMLGVLGCSTI
ACATSFSLITSIHGAAKFAIVQKDFERIDQVTWNNSEIVGRCVRRHQECIRFAETVENIINVL
ALAQFVISTGLICFAGFQMTTMLTDRARFTKYASFLNAAVTELFIFSYGGQSLKSESEEVAE
GVYSSNWIGSALSSNLRLIVLRSRKPCTITAGKFYDMSFESFLKVLSSSFSYFTVLLAMEEE
>NvitOR298
MLGKSSLNSKIPIRERDFNYSMKLSRITLSIIGLWPFRENIRCSNFKFVVILVSILMTLLSSLTF
VYQTDDDDKMFHSLINSLYMLMTLVKLLMMRCKNDKLEVILSEMRIDWRKYERFSDGN
KRLVDLYTGKARTSSFVCIFFMEFSITTYFISRVAYALQQPAKIREWDLPYTAVYPFEVTSSL
FVPMYLWQVFSAMCLGSVTISIDCLLVTTACHATGQLAALCENIKSYGHEQRHRDETLSSE
IECSCIRCIIERHVDIVRYCRLVEDAYNLILLTEFIGTTFQFCLQMYIIVEHSHDKNIVGLLSFC
IYLLVFNFRLFMYCNVFDAMVEMGEKVGASAYDISWYDFHPEAVRQLMFCILRANKPLN
VTAGKFFSLNRNSYKNVIMTSSSYASVLLSIK
>NvitOR299
MKTESSNARLKGKNVTFCTEVFECDEILQLDNELGLIQWSLKLMGIWPFWTRFSNVKFFL
CGAILAFNVVGCFSGILNVNSDIEQFIECLLYFNVNLATLLKFLIVKYKRRSIEFILRCILDDC
SRYSHLSVSCRSRVAGNIKKRKLMTLTLALFILAPVAGKRSFLSMLWSTAYIFSAFAITKYIE
YRDDLAMIRELPIFSALPTFVRHSQIFYLALLSGLFGILMSTLVIVTIDSVFAILMIHATNQFI
VLSEELKAYREDHLDACYKISKNMRNCKCMRCIIDGHVNILRYTLNFYWYSKFILYKIDSV
FSIVHIAVILLSMNLHLFMYCVTSKSMTDASEQIGIRAFKMKWYSFQKTTVRSIVLMTLRS
QIPCYVTVAKFINLSLETYTSVLKTSISYASVIIIAREHLVNER
>NvitOR300
MGKGKVRSFSDYFWLSQGMLKFCGVLPMPERGLFVNYFLIMLSISSLVFLFFPGFYIIAFH
GSEINAAAKVDIIAGEALEIWVTTIKALVLLPCRQTMLSVSRRATRLLVDIEDEKEQQLAEP
YARRGYYLLYGFGGTVFFALLSIVIKPFGQQVQYGANGTILASKDLPYSIGIVHENQQLFN
AWWIGQCFAGIIAIIAIIGIDTTLAIFVLHACGHFRILRSRFQAVAENSSSRRMSVSGRDDRR
RLIDLIDKHQEIIQFVSTIIIIIRYVITLRKCFLFSIKLLRRNRIRLQSGGTDSNDTQHLSDLRIRL
QFAGGKSLYIYIYTSSQVGFEAYNLRWYDWIEDDKSLVTFLITRSQKPMLITAGRFTSISLET
FSAVLSSAFSFFSILRKTL
>NvitOR301

MFVRIIILNEINFLGNMDSEIYDSEYYHEVKLLLTYFGLWPNLSRFRKVVSFIAMVAMPISL
VIPMSFGLKRAIRLKEPIQIIEDTIGILYFLAITTKYICTFIFEGRMIVVYEQIASDWKKIKDKN
ELEYLHGRAKEGKIITILYLGYGAVGCTIFASTPYLPLFLDLVIPLNVSRDKIYPYYADYDIV
DSEKYFYTLYTLHGIFIIILVTMSAISIDCLFIMMVKHSVGLFQIVCYRLKKIGEEHNEKPHE
CKRLMDDKIIHTRMKEIFDSHKSSIECVDAIQASFDVSFLFIMTMSGVGVSLILFDLLLNLD
DLTQILRINSMMFGVYIAVFVICYAAQMTLNSSEIVFNDTYCGYWYNISPNARKYTQMVM
VRSMKPCIITAGGLINMNLQSFFAILKTSVSYATVMLSMQEESNMQN
>MmedOR54
MAILERNIFILTLVGVWKPDHWKGFKAALYYFYICLVTIVNHSFLLSGVLDFELRNIDVVVIIDNLSLLSCLFTVRYKIVTILYYRKLIEEFVNRFERDPFRAKDDEEEKIYI
KFDKFTRTISMLYPGLFTVAVSWYSVGHIFRMSPPNVMPYQGWFPYNYTIYKYYWPTAIYQLYAICSAACVNLAYDSIFCCILYYICAQTHILKYRFSVLVENLQKINEGN
DGSVNVREIERKMIGDWVDYHNDILDLVKFVKSLFSTAIFVQYAASSLLICSIAYTLSHTETRSMNFAGDFFYLTAMTIQIFFQCIAADQVTVEFADITNALYNTNWYNL
SNNVRKAMTIILAEPLKPTLINSGYFVILSLESFTKVIKLSYTIYNVLQ
>MmedOR55
MEILPECFFIFTCIGLWKPAGWTGYKSIAYNVYTVLMISIPSLFVISGFLDLVLLTTDVSDISDNVFLVLTIMAGCGKMFNIILNRNMIFYIIDCLQNKPLRPQDDEEINIKN
RYHQISRFITFSYSTMTGVGVTLFVLGKIIENGPQRILPYRGWLPYNYSQPVVYWLSAGQQAASMIIAGGVNAAFDTFFPGMMFLVCAQINIFKHRFKIMLNTLEVSDH
NNNNIADNDCRKINNIFGESVKHHNYIFQLFDNINTVFSTVIFVQYSVGSVIFCTSIYHMSDIKITTVEFVSNIFYIGSMLSQIFLLCVSANQVTLEFEDLNTALYDSKWFA
VNNSARKCLIIMMINSQKQITFTTGYIVTLSLDSFTSLVKLSYTIYNVLQQS
>MmedOR57
MQVLELNFSLLSILGVWKPRHWHGMKSIFYHIYRSIVVVINHFLLLSGILDLEFKNVDLDAFVDNLSLIFAMIVVRQKVICMIENRTSISYVLDSLGKGPFKLRDHQEEVI
FKRFTDFIRNIIIYYPLVIMSTLMTYSAGHMSVMDSPNVLPYNGWFPYNYSRTNKLYWVTAVYQLYSVFSMSTIYLTLDLLLPCIMCYMCGHIHILRYRFRVMTEKLKIM
VENEKTQDEIINAERKMMAEWVNYHIDILSLVKFINEIFSSVIFVQYTVSSLLLCTIAYLLSHTKPTTMSFAGNFAFITAMFFQILLPCYCADKLTIEFSDISTAIYDSHWYY
LSNNIRRSVVIILRQSYRPVAMTSSFFIILSLESFTKVIKVAYTIYNVLQ
>MmedOR59_partial
MKLTASPEAALQFTKMNVYLVSSWPLPKSVSKSTKIWFNVRWWTAFVSLLLLFLPLTNGVYVFRNDLLIMVKAACQSSASGQALLKLIICRFQSNRLQLLHDEMEIFIK
NADSLEKKYLLKYIKKSGFFHVFGSVSVWAVCLSYIIEPILMSHRYPTDTAYPFVVKSQVLRTILYLQQIMALFFVAAALTIDFQVATLLWFTCVRFEVLGHYFREVSNETE
LVTCIKKHQKILWYANEVKYAVRYITLTTIATTIGVICGCFTLISVSN
>MmedOR60_partial
SHLLFTRFLRYPENCPLLRNYPIYIIAISGNYNDLFIEFVPTKIQSSRQCARRSGVCKRLTGDFRFVLSTMEVLKFNFCLLSIMGVWKPRGWRGIKAIFYYIYRSFVVIIILNS
LVSGILDLRLKNVQLDTFVDNLSLTLAWVVARQKVVCVIENRCGITRIIDSLEKTPFKLRDYREELIFQRFKKLAR

>MmedOR61
MKPLLPESFFILKCIGLWQPVHWKGFKTWIYKLFTVFSIVILFTDTGSQIVDCFVTCKTVSDYADHSFILLTMIGICIKMASLVKNRHRIFELMERLKTGAFQTNNLEEENI
LVKFDNIMKWRTVLFCIFVQMGTVNLIISSVIYLAPQHVTITKLYLPWDTKTVAGYWTAWVLQAISRLFGGPVNVACDSLVSGVMYRASAQFKILAARLRGFLLEPAN
FENSKYDYHRVEKHEAKKMAELVKEHLEIIQIITKLNDIFGFVIFMQYCVSSTVLCVTVFVFAQVRQFDIHCAMMITYTGCLYFQIYLLCSAGTEMTSESLNFVSYIYTFD
WNELKLSTKKSLLIMMLRCHRPLKFRSSQMIELSIESFSQIVKFSYSAYSVLAQSQP
>MmedOR62
MKILSENLALLFYLGLWKPSDWSRWSFKSILYTLYTILIIIISIVFFTTELLDLILVTSSINEFTNNIFMLSAILAACLKISMVLRHRNVFNIILDNLKNYLFENDTPYNEEIIIW
TRYSRTIRLITMFFMSTTFFGVTMMTYATTSINIPRRELLYRAWVPYNYSRPSAYWISTLGQLVAMYILAGVNVAFVLLFAGIMSSICAQISIFRLRFETAFSDVNKPGSGIK
RKSTEEIISKFVRMYLSIRRLFDIVHKLFSWTIMFQYSVGSIIFCASAYNMSQMKVLSFDFLSCVLYVQNIMIELFIMCVACDEITIEFGKLHNIFYDSPWYFFDNYNKKSIA
LMINNSMNPIYFTCGYVVHLSIDSFTHVLKLSYSIYNVLQSTF
>MmedOR63_partial
KIFLLLIALKIIAIVVHIDDPDIIFEALSSVAIDLMFVAMYINIIYRTKSIRKLYDRMRSDWRLLLNDEEKSSLKYHSNLGQLFSSAFAGFAYTSMAIFVAEPIFPRIINSFIETN
ETVPLKLALPLEYIVIDKEKHYWIMLIISSLLVLNISAVVISCDITYITFVQHVCGLFAVVGCRLENTPTDENNSESHTGSGSSSNSKDVSYKHLVSCIRSHRRALKFAELLE
DAYCITFGFIVGINLPLISITGFQI
>MmedOR64_partial
MEKTPIDSHCKVLDIGLHLAKIWGLIGLDNEPPGRYIAIVVFQIFNWVMTTILIILNTSDFIVNFDDLGEVSYNLGYIFPMTTGTIKAYTLLKHQKSIRKLLDDIHKPITKL
KYSSDLGALTTVRIGLTYQNMDFVIFTIACTTVVLTLVLPLPIILKTRQLPIRVVVPFDKDPDPIFELVYLFELYGLFTECLWTIIFDPITMGFIRWIDVQLIILRANYRHCHD
LDTPRATFSMEEQDYRTIKNYKYFNVTPEQRKIRAFVPFSPEEAYVENDSFVKRFKLCVKHHRRLINLIDRYNSTYSVVLFAQISSDCLLTCIGLFQMALGLKRERNVFR
DVIMLGSGLIHLAYWCILGNQLIIEVFQRYTSDKSSLN
>MmedOR65
MTKSRISDYTSLYQLTKKLGTVIGVWPYENSNIFYRIVPYIPMTLHLIISLAIFGYVGEHFPNIALVSRGLSISTSFVTTILKIVCLKINHEDLTELHQHLHPYFKKLLKDSKL
SELILKDVEMFKLVSWSLTICVATCLGSYILTPLSFIISCYFHHIEITNYPLIYPCSYPWKITSNGIVYQFHFVFETCCGLAMFFVSTSVDILFPFYIFQMIAQFREISYNITHIDD
DDDENSIIDKCVTQYERLMRCRDILEKIWGPIILWSVTTNAIVLCTVLFQISKMRSITIVKGSLLVAYVGLKLGQTFIYAWSGSCLIDESEVYRDAVYAANWYGNKRLMTS
IVMMLTQRPLTLTACNFSIVSVKIFITFLNTAVSYFFLLQTLDDKE
>MmedOR66
MDNRCLKKVSQPSSSNSSKCDAFKYTYWYLNLIAIWPLISQSLAIRRVISIIHVFFVTIMLSWQIIFRCYHVYFYAIDLDEQVTLIGPMIFILVSLLKYLAIIAHRKTLMKSIE
HIKTDWSMTKCREEKNIMIKNITVSKKITIVFSVLMYTSGVFYNFMMPHVIPLIFRSNDRNYTKRLIIFPGYNSAFDVDSSPLYEITYLFHTIAIFFCFTVLVSTCNLIVVLVT
HTNCRIQIVIVQLKSLIEDFFDGGKFSYPKMSNMIRHHIRVLEFSDKILRKLVSEICLVEIGATSILLCVDEFCCLRLLSNQDFANIVPYIMLFLALSLNILILSYFSELLNSQFI
EISNQCYMTDWYKIPLKARKYFILIMNMSQRPQKISAGGIIDLSYMTYVQIIKTGFAYLQILKASNMQSNE

>MmedOR67
MKNSIQTHRLFLNKSISILRHCGIFDFNDSTSVYRKIFIYFIILFSQSMTLIYGLTIVADAVANCTDLMIVASDGCIIAGWILVYFKIQKSYSVRHKILKLIDDIRNPIDVLLKS
HDLGVLILVKEYTIFETMDCYLVISCTVFLGTVLIFLSLLTGDLPCRAIFPFDTTISPFFEIAYFIQSYATAFNLVSLMTLEFISLEFLRWTTVQFKILSSNYQNCSSVPLKPLLSF
GFTQNTIDDITVFNVLNIEDEQKKINSFVAFEEREIDIINDCFKWRFKNCIKHHWRLITIINNLNNTFSSCLMAQLGGSLFIMCLNGYLAVTFSDNKQILVRAVIYLLGGF
LHLLYWCALGNELKFQADFLTTSQWMSGWETKYDSSIKNLVTTSMIKTMQPLEMRAGGLFVLTMETFISILKSSYSVFVLLTSVSN
>MmedOR68
MKTLSYSFMVLEFFGGWKPLEWNRNIKGKLYDMYSLTVALTMGTFSISCMLDLFYTTNFEDIVHDMAVSFTFIVNFFKLVFLKWRYNELRNVIKLLDLNICKARSANE
AYIQFKFDKKVESLVKKYGGLVSGAVVAITCASILDNTSTKTLPFNGWFPYRHNDSTGFWVAYFHQSIAHFYVAMIGFSFDTVVYGVLLQNCSQLQILKNRLENFVEIIN
EEKLNNKTNGSVFTIRDSERNLIKQCIHHHWIILQFSEQSNDLFAQIIFIQYSFSSLVLCLSVQSSTKLVFMSPEFISMILYLGCMLSEIFLFCWYSNEVTQESSGIPLAIYNM
DWQVLTTRSQKDLLIMKTRAILPISFTSGHLIELSLDSFTKLIKFSYSAYNLLHQK
>MmedOR69_partial
DQKDLNEGVKIFNWGKWVSKGLGMWPLAPNNYIFIITFFYFTIVMTLEWLDFYHSLNDLDKVISNLSDSLAFTHTYVRGLILRFHVNEMRMLIKNSMRDFDVSTFKN
SWEVEIFMDCINKGKRVTKYIILFIAITEIIWFLQPLAIPGHFVNDNGTIRYILPYQFHIVYEIQTFKSYVLTYILYIPHMFISGFSHSSTECFLITLVYYLSGRLVILADRINALS
KKPNLRKSDLNDIIAEYIRLLK
>MmedOR70
MDEMEILNSVPFESFFRTEIRILRYMGLSYFNRVFSNKEETEKWWEKITPLFGILIMSLLISMEIMKIIRVISISITLAAGIFTAMLSGMLCTFKAVRCWTHRKELFDFIRQLK
VFWDTASSNKFITKNQLNSALYARSLRNYVIVLVFALAFSYAFPVYVGICKHFLYHRDEYFFTFSRIMYPVKYPFTINSFLRYFMCLFFEQVSEILAIVYWLCGDILFIQLTT
HVSIQCEILVNRLHDLNKDNSSDDEGSRRQLVDIVLQHNQLFSHCQWLQRFFSPIAFFVTLINGLNLCFSLYRVDQQVSQGNWSHLIENTIHLINVFGQTILYCMHADL
LTEKLGEVNNAIYYSNWVQSNKKLKMMVLMIMRRAQKEYRFTVYGIITLNLRQFTKIVNTAMSYFTLLRSFG
>MmedOR71_partial
IQIICSWPLELRNIYWKIRSTLFFAYLITSDIFLAQELLDHCGTSYDITSLFGIIFAAVSGHIKILLLHIYNDNIKFIVTNFINDWSIIEDEKSRKIMRQYSSLNRILFFCILTSMFC
YTLKLTIEGLPQKIIIDNITVIERLLPLSAKCWDFSDTPIVIYAFQFTCRIFEFIIYNITSCGIDLYFFILTMHICGQVEITNSNLMNFKTITNGVFDKKKFYEIIIRQKYLFNLIN
LLRESFNYTILTVLLISGIHLNIIIIMIFIALKNNDINSVIRDAAGTIFYFFSQIFIYCYAGEKLSSVIQNSCFSVYSCYWYNFSIKTRKDIKYIMLRNSQEFHLTAGKFYHMN
>MmedOR72
MKKSPIVMRRRYLNKAIFILRFFGILTYDSSASMHLKFLNNFMRLFSMTIVIIFVSTMIADLIVNYDDLMIFADDTCFIVGWFLAFSKEQITYSKRHQIYQLINDIHNPVDV
LSQSSDLEVLVNVKECMLFEAIDCYILMSFVWILIAIINGLSAIVGQLPSRAIFPFDTTTSPYYQIAFCIQAYNVVYHLVNLICVEFISLQLIRYTTLQLRVLSFNYKNCKGDS
KKISSFNSIRKTIDAITIYNLFDIEDEQREITNFAAFENKEINNIDNSFNWRFKTCINHHQRLIRIVKDLNETFRNSLMVQLGGSLLIICLNGYLAVMFPHDKKALIRAVM
YLLSGFTQLLYWCAFGNQLKFQADHLTTGQWMCGWEKNYDRGIKNLVTTAMIKTMQPLEIRAGGLFVLTMETFISILKSSYSVFILLTTVTD
>MmedOR73

MIVDNRLGYVRFQTLIRRLLFIIGFWNTKNSSVLFRSLLLVHLSFFVLPLIGVVNFFKTHISNIFLATKGLSVLVGYSTVIMKMICFMINRKDLNELHAILEPYIDGLVEKSG
VSNDLLKGVSIFRGLCAGLTGCVTTSSTLYAIIPIMTIISQYRHHVRPLKFLHLYPVIYPWERSPTGFIFYCQILNEYFTTFSIITVTASVDSLFVYYVFQMVGMIRDISYNISTF
TDENCEETIRRCVRQYEVLLKCRSKVDKVFGPIILWSMGTNAIVLCGLIFQLSHAKAIPFLTMVLCAAYVSFKVTQIFMFAWAASKFTTESHKLIDTIYAADWIGNKRIST
SIIIMLTQRPLVVTACSYSTISIEMFSAVMNTTISYFLLLKNFDTDV
>MmedOR74_partial
RLKNVQLDTFVDNISLTFAWVIARQKVFCVIENRYGITRIIDSLEKNPFKLRDYREELIFQRFRKFARTIFTYYPILFISSLLTDSFGHMSVMNPPYALPYPGWVPYNYSRTT
QIYWGTVIYQVYAILTSGTINLILDLLLPCMMCYMCGHIHILRHRFQVITEKLQIMSESNKPQKEIITTERRMIAEWVENHRDILNLVKF
>MmedOR75
MPVSTNKIKKASLKNNDNKKDNEMYIKHTRWLFYILGLWPFMSNHLTTSKIFYALIIQIICYSVLMFSIVPSFYHMFWYDKSIKIKIALFGPTGVSVACAFKYLAVIYHLK
NIKNCLNQMKKDWEVSKSEEDWKIMTNYAKKGSNVTYFCMAVMYGGGVSHQSAAPFLPSSPTSLLRNSSDRPLLYPTSLFDPYFNTQNSPIYEIFYLSHIIMGVVVCT
MMIGTCNLGAILVTHICGQIEIIIIKLQNLAENRSHNENFVKNMSSIIQRHNKIIKLSVYTEQILREMCLIEVVAATLFICMDEYYCLMAWKNDNRIGLTIYSILLVAFIFNI
YIFCHIGELLKQQFGKIGDSIYMINWYNFSPKNASNLIMMIAISQNPQKITAGGLIELSFKGFSNVIKTSVAYFNILRMVEL
>MmedOR76_partial
MTKSRISEYRAFHDFTKRLLTIGGLWPYDNTNIFYRLLPYVQIFLNLGMALVVYGFVQKHFSNVAVVTRGFGIMTSFVTAILKVMCLVINHNDLTKLHKNLDPYFEELL
KNPKLLEHILKKVNIFRFLSWALGCCVFAVIAFYIITPLSFIISCYFHHIEIKKYPLIYPGSYPWKIPSSGFVYKAHFIFETLGSFALFFVTTSVDSLFTLYVFQIIGQLREISYCIT
HINHENDDGDFVIYKCIAQYEQLIKCREILEKIYGPVILWIMGTNAVILCALLFQVSQMKSITIIQGLLFTTYSILKMVQTFMYAWS
>MmedOR77
MDIFEEPYYRIIKNFSRVLGQWPYQSPRTKFTITSFVWIAIIVQLVPQIIAIVVHFDDRELLFEAFSAMAIDFACIVKYLNAIYRMDLMKELWDCIRRDRKLLLNDAEKRT
LQYQTNLGYFFSTGYAGFAYMTVVIFVTEPILPIFINMLKDTNETIPLRLALPLEYIIIDIEKHYWLILCISNLLVVNITTVIIQCDTTFITFVQHVCGLFAVVGYRLENTPIDQ
NDSEGHKGDYSLSNSKDVPYKHLVSCIRSHRRALEFAELIEEAYYVSFGLSIGANLPALSVNGYQIITQSHTVQQLLKYASFTIIELLHLFFLCFMSQQLTDMSLEMHESIA
DVSWYDISVKSQKLLILMTLRSQVPCKLTAAKIMDLSIENFATMLKTCASYLTMLLSMQ
>MmedOR78_partial
EYFVDQEKYYVLLLIHAYMTVPVSLGVLLYFDILLGTHVFHACAMFEILRTYLQSIHVSVCNTEISDKAKSDKIIHCVHMHRNALEYADELDASYNVAWFVLLTICLVG
LTFTGTSTVLKLNETSEAIKFIAFSTGHVFHLFFTSFQGQLLIEHSEAIFYSTYLSEWYNMPLLLKKRLIPIMMRSMTPCRLSAGKVYILSLDTFSSVIQKAMSFFTVLTSMR
>MmedOR79
MDLFDDLNWRHTKLLMSSFGAWPFQSRAKRLILATTGHFMLESMLIPEIIKLVTIRNNLKMVADCIPLLILHIMVLIKILSCHQNTQRLKNLLIRVQRDWQLNLDESEIK
ILRNDGHNHKIFMDFYYISMYSAAVIYMLVPIIPKILDYIVPLNESRPSLPFYQAEYFVDPNKYSTIIYVCSCLVTPITPTVFVAFDSIYSCLIQHSCSMFTIIGRRLQNLTNDL
NISRNEKKNDDDKFKLLVNCIRIHKDVLEFVALVEMNFTNYLFVLLGIIVIGISFTGFQTVIMANKLVDKIRIGWFGMCQVVHLYVLSYFGQKLIDHSEYIHKSACLTQW
YSFPHKTKPLIVLIMLRCKILSKITAGKLYVMSVENFTSVMKKSISYFTVLTSVQ

>MmedOR80_partial
KCVIITLKKKVFTFFTDAKYKRINFRFLFCCKAVCIQVKRKEVNELHETLDSYYDDLIRKPKLSNIVSEGITTFRRLPILITGFVTLTCTSYAVVPILSIISQWRHDIHPIKYNF
IFPTAYPWYPLPHTFIYNFHFVNEYLTTFSIIFITGSFDSLFIYHIFQIIGILREIAHDISLIDQDTNSEVIVRQCVIKYEILLECRHKIEKIYGPIILWTMKTNAIILCAVIFQLSHA
KTIPFMVAIIYAGHAGLKLTQVYLYAWSGTRLTIESDKLRDAIYNANWIGNKQLINSLVIMLTQKPLIMRAYHFTTVSIDMFSAVINTTISYYLLLKTFEPNV
>MmedOR81
MVTEKQIEYVKFQSIIKRLLFIVGLWTKEDSSLLFRSLFHIYLSFFIIPIIGVVNFLITNITNMNLATKSLSTLLGFSTVIIKGICFIINRKEVDALHTILDPYFDELLKTPEVSTLV
LNKVSTFRRLPTFITTFITIVCISYAIVPIISIINQCRHRIWPISYNLVYLTIYPWEVSPNSLMYNFHFLDEYLLTISIVLITSSVDSLYTYYIFQMIGMLREISYRISIFDEKNSESIIR
QCVDKYDILTQCCSKIEKVYGPIILWTMNVNAIVLCAVIFQLSHAKSIPFISMVLCAAHACLKLTQVFIFAWAGSLLTTESEKFRDTIYASKWLGNNKLKSSIIIMLAQKP
LILTACNLLYVTIDMFVKVINTTISYYLLLKTFEQGA
>MmedOR82
MSQDELSSYIEYENFIKIILSILGLKTSATDSNSIINRSLLHLQMSMSLLPMIGIFNFLKTYITNVFFVTRGLSILVSFLTIIIKGFCIILNRQDVNELNVILSSQYHKLISNPKMK
AAILKQVTTFRRLSYTMTIFVAISCLSYVVIPLISMINQSINGIKPIKHILPFPAIYSWNIPPDSYRFKLHFLNESLTVLSVICITVGVDNLYTHYVFQMIGFLRVIAYRMINFN
EKNKESSEIVVRECMSQYETLINCRNKLQKIYGPIILWTMGTNAIILCAVIFQLSQMNSISVSRIILFTTYAGAKFSQVFIYAWASSLLTAESDKCRAAIYAANWVGNKRF
MQSIIIMLSQKPLILTACNFTVVSMDVFMSVLNTTLSYFLLLNTFAEKT
>MmedOR83
MQVILQRYHFILFSYSKILLIFLKKIMADKKISDYLIYRTVIKKLVISVGLWPLDKPSIFYNLIPYIQIIINVFMCFGMIGYVRANFTNVALVTRGLSIMTSFLSTIVKVGSFVM
NRKDAIDLHNTLDLYFNKMLNSKQLPWVVLNNITTIRPLTWTFLVLVSISCGVRIIDPFSSVISQISQKISPIKYPLLYPSVFPWSLVPGDTLYNCEFIIELLATMTLWLITMS
VDCLFTFYVFQMIGQLREIADCFDNLDGDDDYDCQNIVRKCVNQYQTIIKCRDLLQKIYGPIILWLMVTNAVILCTVAFSATKMDSIPIGKGLILLTYILLKLVQTFMYA
WSGSLLMAESEECREAIYAAKWFGNKRLMTAIIIILSQKPLNLTACNFSVVSVDIFQAVVNTTVSYFFLLQTIEPDS
>MmedOR84_partial
QCIEFHYECGVNKFILGLIGVWPESNQKFIKRHRTLVDAIVLFVTLWIPRAAAFILLWGEIDALAQAGATNVPIALSVVKLLILYRRKNDFSRILDEMKADWKKPMTKE
EYEVVMKMATLGRRISILSTFLTLNAIWLGAIVQIFYNVETTIVENPDPRLTLNLFWVVYLPYDTSKTTNYVLTWLVHFYTSIMSAVVYGSFDGFVVVLVIHLCGQLDLL
KIWTRKLADQPDLENFKRKVKFIVNRHEHLNEIADTLESSCNFVFLPQLMSCALTFCFQGFAIATKMFTGQFNPLQIAFALFYTGVMVTHLFFCCLAGELLLSKSQDL
GYATYESKWYHLPPKAARSLIMIFRKSTKPFILTAGKFMPLTYNLFLTVLKTSMSYLSVMFAMSSD
>MmedOR85_partial
YLKISDVQVMKWITEIADFENRQFLTVFVMVILIASARIFSADIKNREFPVRIFVPFNASESPYYYLMYLAISYGILIVDYSLFGVDIMVIVIMRYLTVQLEILKTNCRYCRS
DSNKRAINLSSDKNSTIFESSELTNDLNDGDDNKDNEIKEFVMFEMQEKNLYDKDTFYWRFKHCVTHHQKIINMLNVANNCFSFCAVVQIITSTILVCLNGFQIILVSN
SLCQETV
>MmedOR86_partial

MNILQTRHFREFKIALLFLGQWPSQSPLQRYCARFVVLVAIFSIITPKMIKLIESIKDVDQVIECLPMVILHLVSLTKYFNWIFNEKKVNHLFVLIDQDGKNLTSDRDVKI
MKKWLEYIRKISLSYTTAMFSILVLYLSSPAFPKLMDMISPLNQPRGRIYLYQTEYFVDQDEYYVHILIHAYMTVPFSLAVIVYFDNMLATNVAHACAIFEILSTYLENIKY
DISKISVTNAEEHSRKLRNSIIRCVDMHKNALR
>MmedOR87_partial
CNKIIKTLKNNSQRICPCNNNYRYSGVWPLLPTVNIFWKILHLIFRIFNYSALIIQIAGMSTIAINNITDLTVAGEIGGFIIGLIMCLLKFLKFTTSYDEIMNHVDVVFNPINI
LQQSSDQGIVMCIKNCAFREEAQVTLFFLPSSFLPLLMLFFGHKQKVGLPIKTQYPINTAMSPNRELAVFFQSYLVAYCITILLASDMLVIGLIRWSTMQFAALSSNYQNC
NSKLVKRATLVSPKETFDILNKCDAMKITDEDMEIHTFLLFEENEIEKNIDDSFSLRFITCIKNHQRLMKVIHDLNATFSMFLLLQFATSLTVICLNGFQMILNLDDVKNF
MQFAIFIAAMLEELFIYCWYGNEFTWMANSLSYNQWLSGWEYVNDKNNNSDHNTNNNCDNNKLSNLITISMIQTMRPLEFKAVGIFILSMPTFLSVVKSSYSALALL
ITIMDKT
>MmedOR88_partial
NTIMYAGVVYGSMVPFMLGPLVPIFFKLMPEGLLPINSSMALAKPVMFHVEYLYDLDKYYFPIVIHSYFGTMTYITTTVAIDSMFMVYVQHACAIFAIVGNRLEHLVDH
DNIDINIYPKISNDESYSRMVKCIIEHSKALEYAQLIESANSFSFIFQLGFNMVTISFTGYQAMTKLDRPDEAFRYATFTMAVTTVLFYLSWPSQRLADESTRIIESTTRSAW
YLTSMRSRKLLQLFIQKSSVPCQLTAGSFYVLNMQNFSAVVRTSMSYFTVLTSMQ
>MmedOR89
MDRISIKTFKHKKTQVIDGYYVVHYTQLLMKILAVPQSEFELFSVKKIIFSIHIMAVISLSVWSIVFRSLYAYFNVKDFDEQIIVLTPVIFIFVILIKYFIILYRHKEINQSIEYIKN
DWKKITSQEGKSFMIENARISNQLSFLIIVLMYTSGMFYNVVMPLILPIVFKTNTHSNTSERLPIFPGYDVVFNAKISPVYEITYVFFICAVIVVFSTITLMYILTIVLVSHVR
GQVQIIINLLKLLISNITDEKTLLIKMSDLIRHHMKALAFSNHIRKTLYELCFVDLWASAIFFCLDEYCFLKMLDQNDFINMIPYAMLFISLNFNILIMCYSSELLDSQFVEI
GKETYETEWYRFSVKIRTYLVLIISISQRSQKITAGGIVELSYATYLSIIKTGFAYMQILRAADM
>MmedOR90
MTVELRSLNVLTYAGVWKPVKWKGLKGRVYDSYTIIIIISHLTFLISGLMDMGFDDFEFAAMVDHLSLVVGYVQNFPKITSIVGYRRDIINIIDKLNSYPLQLRNNEEKLI
HAKYDRLDRIITFWFPTLGMTSIAWYTMRHFVAIDTAYGLPYRGYFPYNYSSNPIVYWCTAVEQMYSVICLAVVNAGFNVFLPTMMFQVCAKLNVLQHRFKLLIEKLE
TISTHEKNYNQLRLSKIEQQLIRDWVENHIAILNLFKFSNLLFSKAVFIHYVTNTVVLCTVAYMLSQTPPSDLNFITNVFFFMVMCSQQFLQCITAQQVTIEFENLNNEIF
NTNWYTVRTTTIKSMLIIMFKTSRPVIFVTGHFVNLSLDSFKSIMKLSYTIFNVLE
>MmedOR91_partial
MEISNNNELPPCEQFFNPELSLFKLIGFGILERAFIKNNDKIYVKIFEILLILSGFMIMIILVFSEFRTLNKYFFSDLTKTGEVIAMIFGSTIIITKLFRFWISRTDLILILKEFDNL
WEINVRKRLDLKDKVNKIINASKPIRYCYFIAGGSLITSYGVRPYFLMLRYFLKQSENKTMDLTETVYPIIYPIPSGTWPGWLSCVTYEQGIIFFGIIYWIACDTLFILLTSHI
CVHFMVKIKKINFVLI
>MmedOR92_partial
NYYCNKFKELTTTMPLAAVGMTELFKFTGFLIRRNEIIQLNAYTENKFWKIKYNERDIKVLNDCNRLIMSFYPIFIGIVYTIPTQYTLLPIREYILSNGTVRILPYRLYFGVD

WMENPLYTVAYIEEILAGYGVSLCAMATTSFLFTINIYAAGQFRILQNQFTEFCSDITIFNNNFRLSEIHMKLKQLIGKHQLLLSYIYRIENLYNYFLLLQSFGIVLAMCFS
GFGVLMVTGLLKKK
>MmedOR93_partial
GKIIGVLYKHRMITHVIQLLDEKPFLLLDPFENDIKKKYDDMFSFVIRLYCMFFTAAVSGVLITQTLMAEFPTVLPFKGWFPYNYSQPNIFIVTAALQLFTFFHGAYVHVA
FDTLFLGMMLYVSVQVNVLQYRFKYIVKTIVKFNVINQNDDEGINPNKKLFAEWVEHHNHVLSLSNYVHGIYSGPVFLQYCLSSTQICVTVYGMTSVAIFSVEFISRITF
LCGTTLQIFMLCMAAHQVTLEFADLSNSTYNTDWYDLDIKTRKSIIIIVSQALKPVIFTSGYFVTLSLELFKNVIKLSYSIYNFLQ
>MmedOR94
MGTFTSESSGDFEWAIGMTKLGLKVCGVWPDLQRAKWLRVLISLRVTIATLIVVIFTLIPGLIALTRVWGNMTLIIDNLIITLPFFTAVFKLNVLWHKERDLQKLFDKINE
DWSIPRSTMEHNLMIKNATVARNVTLFAYILVTWVILIHHLPLWIGGIVPRTPTNITDGSRALVMQTIYFYEVTESPIFEITGMCQFMSSLVAGSAYTTIDCVFGFLILHVS
GQLQVLELRVNNLCSNYNKKTNYVDKRSFQRDLNTTATVHQRLIDFVEKMEDIFSMMLLEQFTAFAIIFATEGFNIISIPYQCHATNKMRAHVLLLASIGVLATSLMCA
NANWGNHNWSLSNDDIGRIHRTWSLVHRHPVNFGSLFYSRLFQLHPEVRSMFGNFRDVPQDQLLRNQDFLNLGSRVTNVLDDIIWSIHDVSANRVNLNQKWPCP
YAKQGGWKQNDMALTQNVFLDVLSHHVPNWGQADSVAWNRAWNAIWHNLSARV
>MmedOR95_partial
PSLKSRNNSNNNNSLDNIYSTASKDLKWAVGLNRAVLKILGVWYYNNQSQWQTLTFNFQTFIFVIGIFTFVTFPQTLALIKIWGDLTLIVDNLIVNLPITTCELKIFILWW
HKKVLVALFDEIEKDWHGIKNEYERNIMIKYATISRIMTICGLCGAFFSLILYQGPLTFGIVLRTITNLTDDPDNLFSLQAVYLYDTSTTYSYYLTRLSQIVGCGLSAVAYTS
ADVLFGMIVLHVCGQLEILASRIQIIADKPQYFSRLLREHVKNHVRLIRFVQDIEKIFSLMLLALFGSVAVTFCIQGFQFINVFTDTTVDLPITQMVFYMQFLSYCLFLTFV
YSWVGETLLTQSSAIYTAACDCNWISCDHNEVKDIILMIFRSQYPLQITAGNLIPLSMNTFVQLLKTSGGYISFLLAVKE
>MmedOR96_partial
FGILRGLGLWHMENNSGWRIFFQDIHCYVVIIIVYAEVLFESIALFGTFNDLEQFVASSIIYLTMIGVCGKMANIVIKRKEILNLINILKADPCWYRNAEEKNIQDKFDKTI
NYRTLAFLVVTESVIVSTVSISIVRDTPNRSMFYNSWLPFDTSTFFGYWVAYIHQVIAHFCGALVNVAYDTLIPGLMLKICSQLSILEYRLKLLCDNNYFSDLSGDVKQKE
TDAISECVKHHLQIFQLAERANKVFNIVMFLQFSISTFVICVTIYKLSQVEITDPEFTSMIIYGLCMLAQIFGTCLASTECNLKSFEIATAVYQTTWYDLNIDTQKSLFIIMT
RSSRPLRFNAGNFIDVSLNSYNQLIKLSYSAYNVLK
>MmedOR97_partial
MFNRDEKCDYLTTTSCEELWNLDITILNTVGVSLRDAFGGEYRKSNLLETTLYIVGLIVHSLFIYFALHTLYVVKNIDLDFITDFIPVVFGMSMTIMKGSTLWFHRRELFEI
LKEFHTRWIYTKTKVHLQNKIHMLINTSKRVRYCYMTSIIVIGLSFGLRPYLLLFTYYFKTLVLQSNSTIDFSVVIYPLAYPFTYQTMNRYVLLLLYEQCVNYFAICYVTCD
TIFIQLMTHTSINFLVLADDFRNMDQHIDINNDDSDNDNDGVQHMIELVNKHRSMLSICEKIESSYSRVVFFTMVLNGLDLCL
>MmedOR98
MNTNVEKEFTEAKLLFDKVGWIIQFLSAWPLNPTYTKLILFLLYLIYENLYLLIAFNDFFSIFGDLQLMTANLIGSLVQTIMMIRLIFVKFSKKVRNIIIEIQDDVCEKNYKN
PDDKKIYVQYSSLAIKFYRVTMGFGTGAGLSFFILPLQNCILSWFLHKPVFLELPYRVKLFNSNETSFKESILLYIFEAPLPVTALCFFASVNMQFIIITNICARIAILTSRIKK

VRGEVESVSAIKHIVLKHLELIRLTKKVDEIWTPVFCVEIIVLIPLLALVMFSAILALEANETIAFMTLFTYVGAVLSCLFANCLMGELLLTEREQLLEAFYLCNWYKMSIE
CKRSWLICMVNCANIPMHMTAGKIYIYSFNGFTGILKSSMGYVSLLRTLTM
>MmedOR99
MKQLSKDQKDLNEGLKIFNWIKYVSRTLGLWPLAQNNFVFFTTFSYFTVVMILEWMDVYYSLSDFDRVLDNLLENLSYSHIYVRGIILRLKIKKIRRVIEDTIDNFNVDK
FKNSSEIKIFLSYINEGKFFVKSFISFMAMTVIIWYISPLTAPALIADDNETIIYILPYRFHVVFEINDYKSYMLTYISYAPYTFIHGCSHASVECILITLIYYLRGRLVILAGRIN
ALTDKPKVEKYEINEIIMEHIELLRFGETVISTYSTSLMFYMMSATMALCVICYKILINFMIGPNKDLVQYLIYILATYLIIGVLSTVSEGLISECNKVSEAFWNCEWYNMP
PGSINDIMFCISRSQKPLALKIGKFSTFGNNTMTIVKIYF
>MmedOR100_partial
MSLVTWNSGTSYELTIYKIIMWPLGIWPLNRGELFSDIRLFLAAITQASTCICLHIEMWLNCQGLEDILDIFVLSVFSLLACMKGIIVRYHQEKLHCNVTSAVKDWFTLSL
KTNLRNRKIMMEHARIGRIVCISLMAPASGGTLSWIVFALPLPMFIPENSTSVVRNYPLQTACTFQSVTLSGYYHIIFIIQIYQLIATCLGNCGNDVFFFGLGMHICGQLEI
LKNEFRELKTTNNKSEDRKTFQNVVRRHSHLMSLIYKLESSFNLVILAQLIMSGILICIMGE
>MmedOR101
MFSVKITSYFIFREIIKGPLLALGLWPNENPSLFYRLLSYVQLPLNFGMFLAIFNFVRLHVNNITLLTKSFGVMTTYLSTTLKITCLLINRKEALELHRALDPHFSKLAQDV
QMKKIIFKKFKTLKLITWLFFSCVFITLSAMIITPLIDIAMQHHKYGKSNKYPLIYPSKYPWQTSLNGWPYKVTYLFESLATMSLFCITSSVDSLFLFYIFQIIGQLREMSYSI
TNTNEFNNDKEKDVVLRKTIYQYQTLIKCRKIIEKIYGPIILWIMCTNAVIMCVFIFQFMQMKNLPIIRLMIIFTYVLSKVLQTFIYAWSGTCLTVESEDYREAVYNMNWY
GNKNVMTSVVIMLAQKPMVVTACDFSIVTVNIFVMVLKTTVSYFFLLQTLEEK
>MmedOR102
MIVEKRLEYMKFQTLIRRLLLVIGFWSSKNSNIFFQSLLFLHLSFFIIPIIGVFNFFKTHISNIFLATKGLSVLVGYSTVIMKILCFIINRKDINDLHTILDPYIDELIEKSGLTNFI
LNGITSFRRLCKIATGFVTISCTSYAIAPIISIISQWRHHIRPIKYNLIYPTAYPWEHPPSGLLYNCHFLNEYLTTFSIISVTASIDSLFVYYVFQIIGMIRDISHHMTCFNEENGE
ATIRHCVLQYEVLIECRRKIEKVYGPIILWSMGTNAIILCAVIFQLSHAKAIPFTVMVLCAAYAGLKLTQIFIFAWAGSQLTTESENLIDTIYAANWLGNKRTMTSIIIMLSQ
KPLVLTACSLSTVSIDMFVSVINTTISYYLLLKTFDPDS
>MmedOR103_partial
KLQSVSKLFNCYRKIFFIKGMSVVLNRDDVKDLHEMLDSHYNKLIRNPKMTNVILKHITTFRWLSYVAIFFVVVACMSYIILPIVFVISQLIHGAESIKYLLPFPSIYNWNI
PPNGFRYRLHFLTESMTILSIICITIGVDNLYSLHVFQMIGFLREISYRLTHADENSDKEGSSDLIVRECVYRYETLIKCCKKLQRIYGPIILWTMGTNAVILCAVTFQLSQM
SSISVGRIILFTAYAGVKLIQVFIYAWAGSRLTAESEDCRAAIYAANWVGKKRYMRSIIIMLSQKPLILTACNYSIVSVEMFGSVLNTTASYFLLLNTFEQES
>MmedOR104
MNNNDNNIKMKTPNHRSSDIAYALGLYEILGRTLGIWPLDCHNISSIIRITFVTVTQLSMSISLIKQLLVEGNCGEITDVVDVLSLIACGVVTVVKVIIPRIYYNKMYVIVS
TAINDWKTVNNEKARRTMLRYAYIGRVVFIVQIIGAYAAGFQLIVSRLPFIMNWNNDRNYSTPMYTVPIGPSCWISSEISSFHYTAYYTFQCIQLFVVCTAYIGADTYFFGI
AMHVCGQFELLSNSLTNIYSDGQISNQKQNFSKFVERHKHLLCLANNFEETYNLIILSQVGIDALLICISGIVLLMTLHTEDLFIIIGLIIRIYLVYVQLFLYSYVGEQLRTQ

ANKMQLTIYNCPWYKMSPRITKDMVFTIMRTNYSFNLTAGKMYCMNMENFKNIIKTMGSFFSVLRLMFIEKQ

>MmedOR105_partial

MKSTSVETHRVCLNKCLRYFRFSGIWISDSSTPLRKLISLFCRLLSFFVLIAYILTSMADLVVNCNDLVVIVDDSCFIAGAGSAFFKICTVIFKYQKFEKLISDIHDPVDVLR
QSNDKGVMKIIKRCAFFETLDYYLWSNATFVLGFATIFLVTSQKGELPSRAIFPFDITRPSMYAVALSIQIYTVIYGLISLMAIEITMWGLLRWTTVQIQVLSYNYKNCDRN
LSRRARVLSQSQFDGVEKSNFSEIDDEEMEIKNFLLFENDKNNRQVDMNCFQWRLRYCIKHHQRIVEIVNRLNDTLSTCLMVQLAVSTMIFCLNGFLAVTFPHDKKR
LMRSIFFLLVGFIQIFYWCRFGNDLKFQADYLTTSQWMSGWESNFNSNSKNYLTTAMIRTMKPVEIRAG

>MmedOR106

MTVLENSFFLFTCVGFWRPIKWNGLKANLYNFYTAFVVITNCSFFLSGITDIDYAHFDFFGSMDLITLMLQFVENTPKVLCVMNDRSIILEVFRHFQSDPLDPKNEHEK
LIQRKFDNFNRRVNLWFPVLGFTSIIWYTVNHILLMESPTVLPYDGKIPYNYSSNKKIYLLTAVNQIYSVFSLASINAAFNTVFPTMMFQICAKLSILEYRFKLMLKKFEC
DKNNNREKFGEKNFYEIRSNLIGNWVENHIKLLNLFNSVNSLFAKAVFIHYIVNSFLLCTIAYIFSHTPFGGMTSVSYLFYFVVKCSQQFLQCASAHRVTVEFENLRDTI
FDTNWYTTKRAVQKSMIIIMSKTMIPVVFVSGYFVDLSLDSFKSIMKLSYTIYNVLE

>MmedOR107

MRNEMGILGTVPDSTEQSQSDIKYSTEMNRWFLQPIGIWPASSNIFEKIISKILVIVCYFLICFLLIPCGLHTFLNEKDPRLKMKMIGPLSFCLMAISKYCFLVMRKKQIREC
LKHIYVDWRRVRLPADRTIMLINAKIGRFIASLSAVFMYSGGFFYHTIMPLSAGNFITPDNITIRPLTYPVYAPLFAALTSPSYEIVFTIQWFSGFVLYSITIGACSLAAIFVL
HTCGQLKIVMSRLENLIEDNNKKFTDSLDNRIAEIVQIHLRALNFIVRTEKILNEVCLIEFVGCTMNICFLGYYFMTEFERAETIATVTYCVLLVSFTFNIFILCYIGEMLTQ
QGIKVGLTAYTINWYELSGKKAKDLILLLAMSNYPNSITAGKMAELSYNSFCGVLKSAAAYLNLLRTAIL

>MmedOR108_partial

LKSLDYVYYMICIKYCLLQFLVVIIFIHELIVKGNCSVITDIVDALSLIVTSTLSIVKIILPLVYRNRMYLIVNSAIEDWASIRNKQSRVIMLKFAFVGRIVCIVQMVGAYMTIV
PLIFGNPPSFYQFPSNKLEDNSTLLRNIPIGPNCWVSTSMSMFMYIAYYGLITVHLFILCTSYIGGDVYIFGIAMHVCGQLQLLYNEMENLSGKTNYFSLRKQIRQLSQRH
SHLLQLSNEFERTFNFIILLQVAANTFLISISGILLLLSLKTGDSKITFSTLIRIYLLYFQLYIYSYIGEALSTQTKKLQTAIYNSTWYEMSPLLAKDLMFIIMRTSYPFHLTAGKI
YDMNTSTFKDLVKMMFSYFSVLRLVFLE

>MmedOR109_partial

MLLPYSFGVLWCLGLWHTEIISKWRLCLQMIYSYFMIIFIYSDALFESVALVNNYNDFHQFIDGSIVLLTMIGVCGKIANAVIKRKEILQLINILKTDPCLCQNDEEKIIQD
KFDKTINYRTLAFLAVTESVIIISVGVSVLQDTPNRSLFFNTWLPFDTSTIIGYWVAYI

>MmedOR110_partial

MESSESVNKRIKWQIFFMSILGLWPLQKDNFYRKFRVGYSLSQLIIVTVSMIYQFIINCSDIDDTFDSYLVLIFSILIFIKMYYTSTRREYLKQLLISSYQDWESMKDTEDIKL
MVNNSELYNNISLLIFSFGFVSVILYDIQIVIFTDSSAKPILNDYSNKTIYKHQHKFLLPGSCIYDDVSKNLYY

>MmedOR111

MKSVENINERFKWQRFFISMFGLWPLQRDNFYRKFRIGYYLCQLIILTVSVVYKLTINCGDVDDTLLSYLLLIGSIMIFIKIFYASTHRDYLKRLLISSYQDWESIKNADYIE

LMVNNSAIYNSVSLIICSSGLISIILYDIKIIFFTDSSAVPILDHYSNKTVYKDQLQFLLPSSCIYNNVSKNLYYVILVNQMVQSYIIIAGGSCSSAMFILVTGHICGQFDILIKR
MDKFCDNNYNFEVKNHELTTIIQRHQHLIFLSDSLEIVYNKVILTQMAGCLTSLSISGIVLLLSINEKDIITTMRSACIMNLIILESYIFAYMADTLSGKGDIMLRTIYSYWY
KIPIINKNIAVITMRLCVAPYLTVGKFFNLTNESFMDIIKTAFSYLSVLRLFLIE
>MmedOR112_partial
FLKPVLSTILLAVIKSIIWIIFRRQDTTILINFLFNDYWDIVVTYCRANEFKYIDKNAKMAKRITVSYIVLIVNALFIFYLLPLRHHIIKAAGILKIGAQEPTLNVSLNFPFIAA
YPQFCYKSPFYEVVYFSQMLATSMCGLIILAMDTLIATAIFHSCGHFNVLCAKLQRINFQNSYHLTDELITIIKHHQLAIRFSDHLEYVFNPLMLFQVIASSIIICLVGFQV
NTTLKHDNKDKLVEYICYLMMALFQLLLFCWPGDKLINDSLRINEDVYLTNWYSHRFSHTIKTELILMILRSQRPSYLSAGKFHLMSIENFSTILSTSVSYFMLLQNLDV
>MmedOR113_partial
ELINIKNNLSSSNSRIIGILDYTPSTLFLRIKSNVIKVFNWAVALCVTTSLAVDVFKNYSDLEALTNDVGYLFPMLGILLKTIAVNLGQKNILGLIEAVHAPILKLRYSSEIGV
LTKIRITIFYQSLDFIIFAVVLISTGITIIVMAVLSDTKLPLRGVFPFNETVLPAYAGVFYIQSWMVAMCCLWILLIETSAIELIRWKNVQLVILQRNYENCCNWMEPRANFE
MSDDTYERIKNFSYFKLKDEDLKINLFVPFDENEVNVKNDSFILRYKTCLKHHWRIINHVDEYNDFFSVLQFFTVFITCLFVCLCLFQIAVNKQSKAAILNTGILMCSEM
CHLGFWCIFGNLLMNEAETIRQSQYNSGWEKELDSEVRHLVINSLIESKEPLKITAGKFFVLSLATYLAGCIFMLQIFVIQKSYSYFAILNTVHSDD
>MmedOR114
MKWVLMETNSPIITNSNLNKNYAVKYTYWFLNLLGIWPLIFQSLVIRKIIALIHVLIFISICIWEFIFRFIHMYYYVDDFDEQITLIAPILLIFVILLKYMAVIYRRNSIMELIN
HINADWNMIKCQEEKEIMTKNLNKCNNITFVFTVSMYISGAFYNCIMPHVIPFLIGSSNNQNTSERMIIFPGYNVVFNVEFFPLYQITYIFHILTAFLCYTVLIATCNLTV
VLVTHVSSQIQIIISQLNSLLNDFSREKKFSYSMLSLTIRRHVRVLKFSNNILKKTLFEICLIEIGASSALLCIDEFCFLRMLDKKDFANMVPYSMIFLSLSLNILILCYFSELLD
SQFMEIGIQSYAIDWYEIPLQARRYLHLMINMSQRPQKISAGGIIDLSFLTYVQIIKTGFAYLQVLRASNMQKK
>MmedOR115
MTITPIETHRRFLDKSIKILRYCGVMTFDSTASMYQKFYNNTLRIFNYVIVVFYFITLITDVIINYKDLMTIADDGCFIAGWIVTYFKIHKFYTQRHKICKLIDDVHNPVDV
LRQSCDLGVLTTMKTYMFFDALDFFLFSNCAVTLGIALVILVPREKGKLPVHAVFPFDIKKSPNYELALFIQLYTLIFGLISIAMEEFISLGFLRWTTLQLKVLSANYKNCN
SHLIECADFNLTQDTYDCIKKFKISNILDDQIEISKFVEFDRREDKKKIEKVVDCFKWRFKTCVKHHQRITHIIDDLNDVFDSCLIVQFAVSLFLICLNGFLIVMCADDRK
KLISALTYLSVGFLQLLYWCGFGNELSFQANSLTASQMMSGWENWFEIGLKNLVTTAMIKTMQPLEMRAGGIFVLSLDTFINVSEKILTLK
>MmedOR116_partial
CNNHWASTGVPTLLLSTYRIIFTILSCIFVSKEMLEHCGSMKDRILLSAHLLASVTSVGKIIILNLNRKNLSTIITNAVDDWEKTNDVNFKKIMNRYALINRTLLYMMLSP
ALLYVIKVTSDRIPYTLIIDNVTVLIRTTPLSSECSNYADAHIIIYIVRFSFRVAECFIYNLTNNGLDSFFFLLAMHLCSQLEILNIYFRQLIIDEAGVFMKDKFCKLIARQQHL
YYLLNILDDSFNLMILIVLISDFFYLSIIAFTSLVYIKEQKIMEAVVITAAISLNVSF
>MmedOR117_partial
MSQEKISDYLIYREIVKKLLIVVGLWPYDNPSIFYNFLPYIQIFVNLFLCFGMLGFVQKNFTNIGLVTKGMSIMTSLMSTIIKVVCFIKNRSDAMELHKNLDPHFNEILQD
LKLSKFVLKRFSVVRHLSCSFTIIVTISCALRLIIPISIMIKQIKHNIHPVRYPLLFPSVFPWKVTPESYIYEFEFAIESFAVVTLCFITLSVDCLFTFYVYQMIGQLREISYCFKNL

TEKSDSQSILRKCISQYQVLLKSRDILQRVYGPIVLWIMVTNAVIMCTIAFQVTQMDSIPLGRGLLIFTWISLKILQ
>MmedOR118
MTILQNKNIFKKDTEYLIGIAESIKHTRWLFTLLGLWTLMSDHKTTAEKHLNKITRIICLGLMIFVIVPAIYHLVYYEKDYKVKIALFGPIGVCIACALKYIAVIYRRKEIRK
CINELEKDWTKINNDTERDLMFKNIQKGNKVTVYFAMFMFGGGISHQAAAPFLPGTPLSLVRNSTDKPLVFPTSMFNNIFDAQIIYIYVLVYISHILMGAVVCIMTVGT
CNLGAIFVTHLCGQIQIMIYRIKSLSNTNSDINMDINNSQKQIIRIIVLHNRVITLSQNIQSILNEVCFIEILASTIIICVDEYYCMMVWRYNEMFGFAIYMTLLIAFISSVLIF
CYIGELLKEQGEKIGQSVYMVNWYLLPSKTARDFILIIAMTQHPGKITAGGLIELSCNGFTSVCISFFF
>MmedOR119
MEKTPIETHRQTLDLSALALRLCGCWDYQSTQFSLRVVNFLSRMFNWIIVMTIVITMSADAIINFRDLEAVTNDAGFIFPILTILTKAIRLHSRRKQINQLINAVHNPIEK
LRYSSDVGVLTVIRTAISYQNFDFCFFSIMLGSVFCIIFAITSDSEQLALRGYYFCNETVSPGYEIAFFLQLYTIFMCCLWVVIIDTTMLGLIRWINLQLYIIRYNYKNCKHDK
IDRADFSMTREAYEVIKNYNYFKVTDDQTKIRLFIPFDMSEVNIKIDSFTIRFKLCIKHYQKLTKNINDFNEIFSSILFIQIFLVIFFTCLCLFQAVLTMNQKAKFIKYIFLLGA
ELVHLFYYCFFGNELINQGEAIVDSIWNSGWVDHMSPEIKDLMINALLQTTKPTVINAGYFFTLSVKTFLSIIQKSYSYFAILITMMNGSN
>MmedOR120_partial
VRTSSQIESSPLQFLILFFSYRNILVFQPMIFSSALLFYSCSRKNIIDPPYILPYRGWIPYSYTSPVVVYWGTTIFQFYAIYTATAINIAVDPLLSAIICQMCAQIHILRHRFGEMV
NRLKVIDNYEPDTVFAERKLIAQWVEYHIHVLSSVKYMNKIFSSVIFVQYTVSSLVLCTLSFLLSHTETMTTNFVGYLGFLTAMYVQIFLPCYCTHMLTIECLNLSSGIFET
DWFKLSTNIRKSIIIIVSKCYKPVLITSSFFIVLSLESFTKIIKLSYTIYNVLE
>MmedOR121_partial
ILPQSFFILTCIGLWRPVDWQGWKCILYDAYSYFVVVTNFAFSLSQFLDIILVRTSINELTNNLTMLLVMVTACGKILGILCNRNEIIQIIKSLEEKPFKPRDQYEINIRNKYV
YVSSVLTRSYCVYLTIGISGILLTRIPEAKLPDVLPFSSWLPYNYSNPKIYLLTAAQQIINSLISTYAQVGFDTLFPGMMMYVSAQTNILQYRFKKVIKALEKINLKNSADQ
VKNHKDAEKNIITEWVECHIAVLRLCLRNIFEACFFAVLLKLNNALRNSILFF
>MmedOR122_partial
MTERKMEKGKFINSITFEEFLNGEVLILRCMGLSYFNRVFSNSEEIEKWWEKIIPFCGILTMCLLISLEIRLVLRVIVVDITLATEILTAMLSGMLCTFKAIRCWTHRKELFD
FLRQLKTLWDTADSNKYITEDVLNIVLYAKSFRNYLTAVVISLAVSWGFPAYVVLGNHLIFHRDEYFFNLSMIMYPVAYPFTINSYSIYFSCLLFEQIAELLAIVFWLCGDA
LFIQLTTHVGAQCTILVNRLRGINKDGNGSDNQENHRQLADIISIHHKLYLYVTLLLATKIFQPHRTFCHPN
>MmedOR123_partial
NVKLLSGFTYACQWSEISLRILGIWPNSKPSFWNKIKAYLYVSFVLAAYYLPQSATIVIIFGDIDSMVNLLSYNVSGLVAIAKYIVIYKHRKVLMKIFEVMAKDWGAHNS
DYDLTIMMNNAKIGRLLAFISILYAPLIVILHILVTTYLKPDRLLLYGSIPTIANNLMWPSYFPFNSSRKYIFELTWLCQITATMGASLIYGTFDTFIAVIILHLCSQLAIVRNR
LRNLNEDMNQGLLFHDSVKKKLRAIIKRHEQLISMAESIEKTFNVILLPQLICYPLIFCFQGYAMLTVSVDF
>MmedOR124
MATDVEKQFKEAKLLFDKIGWMIQILNAWPIDRTTRGLILFIFYLIYENIYLSMAYNDFFSIFGDLQLMTANLLGTLIQTVMMTRLTFVRFSKSLEKIIIEVTDNFCDKNY

QDPNDKRIYVQYSSLAVKFYKITMIFAAGSAFGFGLLPLQKCIFSWLLHKPVVLELPFKIKVFNHKNLSADQTVLLYMYELPLPINACVYNASISLQFMIIMNICARTAIL
RNLMKDVNVNRETTSQKLLFKQIALKHLVLLKLTKAFEQTWTPIFCFETLILVPLMSLVMFSVLAIDGNETVAILTLIAYVGSMMSCLFANCWMGQLLLNERDQLLEA
FYGCNWYAMSNECKRFWLICMINCGNIPMQITAGKIYIFSFNGFTGILKSSLGYVSLLRTLTL
>MmedOR125
MDLQVENELKKAKSLLNKVSWVMGFLGAWPVQKSLFQRIKFTIFVIYKSLFLLMAFNGLVANFDNVKLMTVFVQQIAGLSMTFNRILFINLSTNVKEIIIATQNEIRNI
DQKNLSEKMIYIKIHQTAKIYFTLTISFVFFTAVYWYILPLQRCFVAWLSHKPLVLVVPYKVKVFYFNVSSFERSFLLYISQIPTSLMQPTYVASINMQLVVVTNLCAQIAIL
SSKVRSLDLEKNSSSSNLILRDIVLRHVELYKLARKIENTWTPIYCLELAVLTPLISIVIYNAMILVGSGQKVTAIPYCTYMIVTLGSLFGHCLMGESLKNQCDLLSEAYYH
CNWTDMSMINKKKLIICMTYTNITLHITAGKFFVYSFNAYTGMLKSIMAYVSLLRTVAM
>MmedOR126
MALSEINAEDKLLLDKIIWAIELLGCWPLNRSIKQTILFLVSITYYLIHLSMSYTDLINVFGNLELMTINLMETAVQMIAIIRLVYLKLSPRVKKTIISYKENLNIERTNDSTE
VQIYDYYRSVAKLYFSIIMPFSAMTSFAWYLMPLQNCLIAWLKNEPVILVPPYKVVYVFFNLTSMEQIIVAYIYQAPMSFLPLGFIGIICLKIVIIANVCSQLAVLSYRIKNLN
FEDNNKKFSVFGYIVRKHWGLVRFIMEIDDTWTLIFCFELMLSTILCALVTYNAVMAIGNKDKIEIISLFTYVISSLLILFANCLMGEMLKFESENLQEALYSCDWFEMSI
NHKRSLLICMTRSQIPLQLTAGKCYVYSFNAYVQIVKSAMGCVSLLRTLTM
>MmedOR127
MDFEKFVDNIFNAITHIKFWEKNVSLTFSKNIINIFLNLVLLTLSSLLVISETIDIYNSYDLTSFAGHLNPVLFHSIGLFKWIFIIMKMNDIEDILLKMKRCHSICLEYHENEK
ERYQYDLQIINFQQKMIKFTQIWFFICLSGVIQWCMNPVVYDYYNIYILEIVNGNYTRNLPYPGVYPWIIDSNYKYIMTFAFQLTSAITTSIEMATSDILNVIFLSNISFNIQ
LLNETLIKEKDVLLSLRFSNSDLAIKKFKKKLRNCLIHHQEILEFVDKLKTVSSYPIFLLCLDSTVALCLISLEVSTIKINSSIECIMKLMSMAEYWSGVTVELFLFSFIATKIE
ELGLKTADAIYSCNWEQSMVNHKGKFSGEHRQITVEINQMINFSLMRAQKPILFNGGLFYILSLQTFKAVSLKLIVALL
>MmedOR128_partial
MKLSTHTKGYKVTPEGAVNFIKVTVYLTCISFPLTERTKVRIKYEILLWLSIVLSIFLFAPLLVSIIKYSDNTFIVLKSFLLMSAITNFVIKVIIVRIYHKELQQLGSALDQYLEK
ASESERVILQKYVDHTWKFHGFITCSYYVTATTLMMGPLILPQKFPTDAVYPFPVDNPIISLIVYLHQCAVGYQCSAGMALDCQTALFLWYLSARFEILILEAKNVGSVD
ELRNYIRKHQKILL
>MmedOR129
MKHSVNRQGCKVTPEAAIEFTKTSVLLTYIWSPPSAKKVRFMLFRIFMYTSVFFSVIFILSLTMSIIKYFDNLLIVMKSMAVLCGVTNFVAKVIIVRIYCKEFEQIELTLKDF
VKNANESERVILQKYVNKCWKFQFLVTCGSYVTTTLIMLGPLVLPQKFPTDAVYPFPVENKIVSCIVYLHQSIIGYHCSAAIILDCQMALFLWYLCARFESLGLEIKNVT
NYQELCYIIRKHQSLLVYAEEVTRPIHAVVFSTVTITKFIMICGAFFLLSDEPIAGKIQFGIMVMSTTLNIYTSIWPADCLLDISSKLITNEIYDMCWTWTQQMRKLCLLLIR
RTQQPVVIKIPGLLETLSNQYYSSFLSAAVSGFTALRVIVNS
>MmedOR130_partial
FITSVLRIVRKINVTKNILCISVDILIFRYRLKNAANNKYVWNKRKKIIREDRMHQLMINCIKHHNNILEYCQILKSTYNSCLLCIVMINMLALSITGFQTVMKMNETSE

MIRFGTFSIGQVVHLFFLSWPGQRLLDHSLSIHQLVYSGEWYNISTKTKKLSIFVMMRSRKPCILSAGKMYTMSCESFSKVIKASMSYFTLLTSLK
>MmedOR131
MNLDEKKIEEAGLLLGLDQMAWIINLVGAWPIQRTLKRNIIFSMYLVYHNLYLVTSYSYLVIVFGNLELMTGNLMESAIQSMTLIRLLTINLSPQLKKNIFVVRQNLRKK
HYEDSTEKNLYDKYYLIAKKYCTFTMIFTVVTSVSWYFMPIQTCVISWFLNKPVILVAPYKVHLFFNVSSIKRTVILYIFQSPLQYPPICCAATINVNVIIITNICGQMAVFS
HRIKNLKTNDSSNKVFNNIVNKHLELVRLTKNFEESWTIIFGFELVVTTILCALVLYNALMAFSADDKIGLISLVAYVYASLLFLFANCLMGELLKFESQNLMESLYICDW
YNMSINNRRALLICMTRAQLPLEITAGKFYVYSYNAYIKIIKSAMAYVSMLRTLTM
>MmedOR132
MGMLNIYWKFFMAVGLWRPIKWHGLKAHLYDCYTMFVMLMNYLFFMTGVMDINFRNLDFFGSIDIITLMLQSIENFAKIFCLVKKRNDLLNLNIYLHSEPFNLRND
DEILIQKKYNDINRIVNFVCLTLGLLSTLWYTGFNAFQMIMPTVLPYRSKMPFNYSEPKIFIITAASQCYTVVTIGIINTTFGILFPSMMLQICAKICILQYRFRMIITKLENN
DYKGNDNSSVEKTNENSPKYLIAKWVESHIALLHLFKCANSLFAETVFIHYVINSFVMCTLTLLLSRSSFDISLIVNAFYFALKCVQQFGQCASAHQVTLEFEDLRHVIF
STNWYAIDVEVQKLLTIIMSKTIKDVIFVSGYFVDLSLDSFKSIMKLSYTIFNVIDQ
>MmedOR133
MTMDIFDEPYYRVVKNSSHLIGQWPYQSPQKKVMITVTIWTAFFMQFIPQVIAIVMHLDDPDILFEAFSSMAIDFGFIFKYFNAIHKTNLMKKLYNRIVSDWKLLLNDE
EKTTLQHHTNLGRIFSSGYAGFAYTTTAIFLSEPILPRIINYFSKSNESVPLKFALPLEYIIFEKENHYWLMLAITNMFAINMIIVTISCDIMFITFVQHVCGLFAVVGFRIENS
PTGKITDSNHRAVSLRKNSQDFSYKHLVSCIRSHRRALEFVKLLEETFTGTFGVVVALNLPMISITGLQLITQSNTVEQTLKYLMFALAQVLHLFFDCFLSQNLTNMSSRI
PQCIANMKWYNISKNSQKLTLLMTMRSQTPCKLTAGKIMELSIENFGMMMKTAGSYFTVFLSMR
>MmedOR134_partial
MLLPYSFGILQCLGLWQTTERVSRWKIYLYIIHKNLMIMFIYSCVLFELIALIASFDDLDEFINNLIVLLTMVGVCGKIANVISKRKEILRLINILETYPCLCQNPEEKCIQDE
FDKTIKLRTIAYFGLTESAVAMVVSVSILCDTPNRSLPFKTWLPFDSKTFFGYWTAYNHQILAHVFGALVNVAYDTLIPGLMLKICAQLSILEYRLKSIPKKIYSSDLFIDVK
RREINEISKCVIHHLKIFQLSI
>MmedOR135_partial
MENISTDTYRRLLDIYITALRFTGIWPLLPSAKTGWKIFNFTYRFFNLALFIFYLIALGTDALDNVTDLTIFGCDGCFFFGTIMIMFKAYKFNISYDKILMLIDHVYDPIRLL
SRTSDSGIIMSIKNCIFQERLEISFFCVSCSMFALTVIFLVPREKGALPIRCIYPFDTIKSPNHEIVVLHQSYAMVYSLVVLIAMDAMTVGFIRWSTIQIQVLTSNYKNCNVHS
IRRATLISPLLTDKSVKIENNIEIFDEDTEICEFLPFYHNEIENIVEDSFFSRFRTCIKNHQRIIEMINELNQIFSSSMLVQFATSTAIICLNGFQLIVNSNDTSMFMKFFSYWIS
CFAQLLFWCWYGNQFTYLYTLL
>MmedOR136
MENIPIDIHRKFLNINITILRYSGVWPLLPTAKIGWKVFNFIYRIFNLTVFIFYLITLGADAVTNYKDLTIFGSDGCYFFGTCMCVFKACKFWASYHKIIKLIVDVYDPIDVL
VRSADPGILMNIKKSYYQESIAFWGFSTLCSFFHFSVIFLIPREKGILPIRAIYPFDTKISPNYELAIIYQAYCLAYALCVTIALDITTIGFIRWSTLQIAALTSNYKNSNPNVTK
RASLVTSSSDARKIIEKLNKIKITDDDVEIETFLPLDYHETKYFINDLFLSRFTTCIKNHQRLIKIIRDLNAVLSPLMLVQFATSTCIICLNGYQMILSTNNSADFTKFTAFLAI

CFTELFFWCWYGNEFSYIADTLTHNQWISGWEDAYDNNYYYFNSNNHKISNYVTISMIPTMHRLQFKSVGIFSLSMPTFLSVVKSSYSLLIILNTFTSDK
>MmedOR137_partial
TLRTYRLCGIWSLDSSSPIFLKLLHNISNIFGIITLIIFVGTLTIDLILNSNDLLIATDDGCYLAGISVIVFKVYEFHRQHKRIKNLTDATYQPIYVFWKSTDTGVKTVLKTNK
FYEDLGFKFFVSLGGFLVIALIFFVPTEEGALPIRGAYPFNTTISPMHEVAFCLQIYAVTYGLMAILMMDTVGLGIMRWLNVQCIILASNYRNCRANQNNSFYLESRDLS
KIASIEDDNNNVTDIYDEPDSNITTFCPFDEQDPAGMSDCFIGRFKRCIKNHQRLLNTIDELNACFSSCMLMQLFASFSMICLTGFQAVLGATTKTSLIKFVLYLGAAFS
QLLYWCWFGNELLYEVFILPHD
>MmedOR138_partial
FSDPGVRKIIKRTAFFENLMDIFFIILGCFLAVALTFFVPKVNGALPIRAVFPFDTTQTPLHELAFIIQAFSIFYGLLTIVFMDELIVTLIMWINCQLVILNSNYKNCSIDTEGQ
ASIEKKSCLSGEINELSNEKFLIRTFVLFDDETDGRIDDNFLVKFKYCIKHHQRLIVIVDHLNDIFSSSMLLQLFASFSMICLTGFQAVLVRFLRSKVTEDNIFFYIGISPQFSA
SSL
>MmedOR139_partial
MIFFLKYFLHRGHYKSVLCSSTIIVQLTTCTSSIITTQNMFKTKVYIYLIYRKMMKRLLLPFGLWPKENPTVFYRLLSYLQLPLNLGMSLAISNFVRLHANSIKFLTKSFGV
MTTYLSSTLKVNKFNKQKKNISYKLFFQITCFLINHKEALYLHKTLDLHFDKLTQDVKMKKIIFKKFTLLILVTWIFFFTVLITLSVMVMTPLIDIVTQHNENHENMKYS
LVYPSEFPWQISSNGWSYKITFIFESLATISLFCITMRVDSLFLFYIFQIIAQLREMSYRITNTNDNKEINFVLRKVILQYQTLTKCREIIEKIYGPIILCIMCTNAVIMCVFIYQL
MEKILFS
>MmedOR140_partial
MSKNIISDYRAFHKLTKRLTTVVGLWPYENPCIFYRLLPYLQISFNLILVLAILAFVCKHFSNIFLVTSCLSIMTSFMTVIIKLLFLVVNRKDLTELHQNLDPYFNGLLNNP
KLSKLIYKKMNTFKCLSWASFTCVFFSSALCIITPIVFVCCYTNEINIKKYRPTCSSDLRKLTSNGLFYQIYLAFETLASFESFFVTTTVDSLFMLYVFQIIAKLREISHCISHIE
DKNDENSVICKCVSQYVKLIRCRDSLEKIYGPIILWIMGTNTIVFCSLLFQISQV
>MmedOR141_partial
MRNNEKDNLDNIKMFNEKYALQINRWTLKILGIWQFIIESSYFSKIVAVCLMIVCINLLTFVIIPGALFAFVLIKDPALRLRITGALSYIVMGIIKYYYLTTQRYKISDCINH
LIYDWKKINEFDDKNIMIDYAKFGRQSSILSAIFMYSSSIIYVIILPFESTGINNKGNVTTLSFVYPCHFIIFNQYESPAYEIVYIMHCSCAFVLASITNATCNLATVFIMHAC
GQLEIMTLWLNDLVTPENTDKKTYSKYSAIIEQHTKTLRLVEKIKELFQHICFVEVIGCTLNICLLGYYILLVSALNI
>MmedOR142_partial
DLFLGFVYVSTVMYVTEPALPMIINFIFKTNLSAPHKFSVPMEWVVIDKEKYYWILLNNSGVCISVILSVLVSCDVIFITVVFHACGLFAVGGYRIKNLTNAKYCKNNLA
ELKLSTNIDDVHYVHIVSCIRIHRRALEYFNLIESTFAGCFGVAIGFNLPIMSITGVQIITQTNTMQELIKLALFTIGQIMHLFFECFLSQQLTDMSSQIQQDIVNGEWYNIS
KKSQKLLILMTLRSQVPCILTAGKIMGLSLESFGMMMKTSGSYFTVLLSMQ
>MmedOR143_partial
RKWKIQVPFVYERFEYAIKWSKISLTLLGIWPNINYSISQKINVVFDIIFFITTTYLPNLGAIILMWGDIDSMVHLLSYNMATAVSIIKYIVFYKKRNVLASLIEFMKEDWID

AESSGNVNKMMSNAKIGRTLAFISIAYTPITIFLYIIVEIHLKPATMFLYGQHPKILTNIVWPSYLPFNLSIYTLELMRVCQVTGLLGASLIYGTFDTFLAVLILNLCSQLSIV
RNQLRHVCSNSRDTELTKYKFDKELRTIIQRHEHLMK
>MmedOR144_partial
SGAGITMAISILSEINLNCGEVNDVIQMYSLAACCAIVIIKITSLRYNNKKILAIITSSIYDWETVTHPKALEIMKRNAKLARSVCLFQMISAYLTTVMIIIGPLPYIALQINA
TLNSSDMIFRPLPLRTVCFYGEMSTKMYSAVYILQAIQTLSTCTANIGCDCYFFGIALHVAGQFEWLGVEFETLNTKASENECRKSLATLVSKHNHLMQLSNYLEDSFH
SCIFLILLVNTVQICLNGMQMIISVRTGDAATAINAVIIIYVMNLQIFLYSYAGDRLTSGIANLHVAIYGSTWYDLPQKTIKDLSFIMLRVNKAFNITAGKIYPMNIDSFKSI
FKAMLSYFSVMQAMFEE
>MmedOR145_partial
IMISGIFIIQELLDHCGTSYEIASLFGMIFGTISAITKISLLNIYHKNISFIVKNVINDWRIINDNNCKTIMRRYSSLNRVLFYGILTPLLLYTLKFTIDRIPHTIFTNDNTTIYVR
TTPISSECWNLADIPTVFYIIRFACRTFEFVIYNIVSCGIDLYFLVLAMHICGQMEISNMNIQNFLVANDDRFEREKFYKLIDRQKYMLGLMDELRESFNYITLAVLLISGIH
LNIMIIMIFVALKDNNMNAVFEDAAGTILYFSAQIFIYCYAGDELSSKVENSRLAVYSCSWYNFSINTRKDIIYIMLRVNKEFHLTAGKFYYMNLLNFTNIVKTMVSFFSV
MRLVIFE
>MmedOR146_partial
MTDKERALVEKEFDNVSSLLMWSKRFLTIGGIWPLDATYKRATVWTVYLTFHLIMEYAELLAVYGDLEMMVLSVLESVMQSMVYAKLFVFRYSIMLRHLIHAIIEDFE
NKYYDNWEEKKIYLEYNVLAKLFYKISMPYIIVAASLYYLRPILTYYVIMPLVTDTSNFTFLVLPFQLKTFIPIETTQSYFIMYGYLSPMVYLLVCHNAWICLLITMVLHICG
QLAVLNHRIKKIPVDIDEDKVQIIFRRLVRRHMRSVWMAKTLDDTFHFVLLIDLVGTTMLLGLMSYIVIIVIK
>MmedOR147
MNHTNINMQNSERKCSLAKKEFYLYEFLTSNIGLWALQKDNMNRTFRIFYATVQMLVAILATVAGFLIGCGNTEEYLDCYLMVVFGVLVLSKIYYAYTYRHHLRYILNS
AFRDWKYIRKKDEVKIMMNSSRFCNNMAAMFYISGMISLISYVLKTMIFDDPRKIIVIDSLNITYKYGHSFILPGGCVYDGVNQYLYYFILINQCVQMTIMCFTNLGND
TMYAAITGHICVQFDILIKRMKKFGQFKNDITVNSHELGNIVKRHQHLISLTENLEKVYNRIIFVQMLITVSVLSVGGVSLILSINSNNMMNIIKSAGVMNFMLFESYLY
TYPADNLANRAELMLRAIYSSCWYKMPQSITKNLILVMMRINLPPFLTCGKFFFMTRRSYMDVIKTAARIYQYYE
>MmedOR148
MYENNDFLEFENFVDSLHNFFTHIQFWKNNATSVGQKIFDIFLNVSMLTLSILLIISETIDIKNSYDLPSFAAHINPICFHLNGLLKWCFGLMKLNDIETLLVKMKRCHR
LCLNYIENEKEINRYNMKMLKYKQNMMKFSYIWLFFTIYGVTQWCLNPILYDLYNDIYSEKMLNSSFDRHLPYPGLFPWNVDTLSKYIMTFSLQYISAIAAAIEIAGYDI
LNIILLVNIYVNLQHLNKTLFEQAENLSKFSNKVDTIEVRNKLKMYMIHHREILEFVEKLKKVSSYPMFILCLDSTIALCLVSVEVSTMRIDVIIIFLSYIDNVTLCMENIFM
CYLKILFMLKFKNFDADNHQL
>MmedOR149
MEANISVRNLSLPSNNETSVKDYEWAVNYHRKVFKLCGLWSYSKPLEWYIKLLTDVHSLFIVACIITSLTIPEVMALTKVWADLTLIVDNFLSSIPLCSAQIKLLVLWTKR
KAIGKIFDAVKSDWLEPKNEFERQIMIKYARIANIMMIIGITNIAYNLIIFHGCVIFGFSFRTTTNLTDIEGYLIATQTVFPFDVTIGYRYWIIRIVHAVECYLAGTVYTGIDV

FFGMSVLHNCGQLEILGEKIKCMVNSKEPLIFKKLLKTIVLRHYRIISLIEEIKDIFSSVLLLLVMCFGITFSVLGFLIASSLGSSGTQVPFIQMNFYLGYIFFFVGLMFVYSW
VGENLVTHSEGIYLAAYNCDWTALDSKQTAQLLILLVRSQKPLEVTIGKFAPVSLNTFAQLLKTSMGYISVLLAKQN
>MmedOR150_partial
AACIVINRKDVNELRTIFDSYFERLIRKPELLNIVLNGVSSFRLLTIIGTVLTTIICTCYALFPVISVVNQLKQYSEPIKYNHVFPIIYPWNDSFNNVVYYLHILNENLTSFSLI
VITSGVDSLFIYYIFHLIGMVREISYLISSLNNQDNIEATIKQCIFKYELLLKCRDKIEIIFGPIILWNMKTNSLTLCASILQLSNAKSTPLVLIILCIALTSLKIFQAFILGWTGSR
LTIESEKLRDVIYAADWLGNKQVMNSIIIMLSQKPLVIKACQFATVSIEMFSAIINTTISYYLLLKNFEIDS
>MmedOR151_partial
KIFELIITSVSQAFVYSACIMYILEPMIPWIISIIYKSNNPVPRKFSLPLEYLIFDQDKYYWLLLIISNVFLLLIMSVVIACDVLLVTCVHHVTGLFAVVGFRIENPPPRVEIRQS
DSQKNNFSNLRDAQYEHYVSCVKAHSRALKYAELLETNFSISLGAVVALNLPLMSVTGVQLITQSNKIEQTIKNVMYIVAQSVHLYFDCFLSEKLIDMSMNIQRCIAKA
QWYDNSTKSQKLLMLMTIRSQKPCKLTAGKIIELTIENFGMVLSNCFFNTFIKISSFNIYAARRN
>MmedOR152
MKELLNEGVKVFDWARWISVGIGVWPLTLNNYIFNIAFFYSTVVLIFEFIDLFVHIYDFAAVVDNLSESLAITIDYSYTFSLRFYHKKLAQVFKEALMDYNSASAFKNLG
EVEVFMMYTNNAKVFTKYIIISIAMSEFLWYIQPLTTPTKSNNSADALAIDNEMATFSLPYRIYAFYEINSLKNYVITYVSLSLFAVINALGIMSLNIFLIILVFHVSGRLAVL
AIRIDDLQKNDSQPRDEFVDIITEHIKVLNRLGNDIADVFSTPLLIYFLLTNLLLCILGYEILLNFMSGLNSDIMQFIVLLSTVYLMLFIICMNSENLASESDNVCQAFYNC
NWYNLPKNTVKDIIFCLVRSQKPLFLKAGKFATFSYITLTDVRFYF
>MmedOR153_partial
IFNIRFCGCWNYDSSKFSLKLVNFLSRTVNWACCLCILIPLAADIIKNIKSLEIITNDMGYLAPLYSTFLKSIKVQTLQKEIGQLINSIHKPIDKLRYSSDVGVLTKIRTAICY
QNFDYSLYASVLSFVFCAVIIISASVSDTGLPMRGYFPFNETISPAFQIVFLLQFWSVLINCAWALLIDLLLIGLIRWINVQLYVLQNNYENCRPDISDRDNFSISHDNYALI
KNYNFFKVPEEQFKIRSFVAFQMDEINVKNDSFALRFKTCIKHHQRIIDSVNDYNDLFNSMLFVQIFNNQSVACLFLFQGVLVRILLPHCSA
>MmedOR154_partial
FLQINIKIQKISSFFTYKIFKVICFMIRREDLTNLHKTLDPYFNEMLKDSKLSKFILRKVNTFRYISLVYAVLLSLCSVFYIVAPLIAIYYCHHHKINLTKYPLIYPTTYPWKIIS
TGLVYQIHYIFEILASLTLFFVTTSVDSLFTFYIFQMIGQLREISYCITNINDEDDGKSAVCKCVTQYEKILKCREILEKIYGPIILWIMTTNAIVLCTLLFQISQMKSITVIQGIL
FTTYFVLKMIQTFMYAWSGSC
>MmedOR155
MNLSVEEKFEEVKLLIDNIAGFINILGGWPIEPTLKRQILFYIYLVYHIVYLSMGYNDLFEIIGNLDLITGNLTTTAMQTIVLVRMLYVQFSKNIFQIIMSVKVGILEKNDYS
QSEKEIFLSYYPLARKYHKVTIVFAFVAGFSWCALPLQNYFISLLLNRPAILIAPYRVKIFFLNATSFKSTVVIYILETPLPVPPICYVAIVNLQVVLVIYICAHMAILSQRISN
FRLENKSSYRKFCHLINAHLEMIKLAQNIENTWTPIYLFEMLVLTPIVALVMYYGLLAFDAGEKVAFISLCTYVCAAMSCLFANCLMGEMLKTECERLFDAYYHCNWY
DMPISDRGVVLTCMTYTKNPLTITAGKVYTFSFNAFAGIVKSSLAYVSILRTLLV
>MmedOR156_partial

RYTSLIITLTFAAVPLIFHRNIFDMMRGFFFVAAGLIDLYVITLPADDLHDLSTKISSGIYESNWMGSSLSVQKSLIIMMCRAQKPLVINVEGILPALTCQFYAASVSFIVSFF
MTLRALVVDK
>MmedOR157_partial
GFVYMTTVMFITEPLLPMIINLILKTNISAPHEFPVPMEWIVIDKEKYYWLLFSNSSVCIMVILTVLISYDVIFITFMHHAYGLFAITGYQSFPNLLELNPDLSWFKLNVFSV
DIESKICLVMQYVKTILHPEQMYHQIQMMSIINIWFRASEFTNGLWNMLI
>MmedOR158_partial
LMCYQRFAELIETTFSTSFGVVVALNLPIMSITGLQLITQSNTVEKILKYLMFALAQMLHLFFDCFLSQNLTDMSSHIPQCIANMKWYNNSKESQKLILLMIMRSQVPCK
LTAGKIMELSIENFGVMVKTSGSYFTMLLSMQ
>MmedOR159_partial
IVFFNYQFFFSLVDKICIGTRCICKYRLLHSIGLEFIGSYCNWYDVIKLHQPDEAIKFGAFTMGAFFHLFYSSFQGQILITQSEKVFYSIYESEWYNLPRYHQNLLNIILLKSI
KPFTVSAGKLYILSMDTFSIVLKNAMSFFTVLSSMR
>MmedOR160_partial
IIAIVTHFDDREVLLEALAPFIIDIVCVAKYINSIYNAELMVTLFERIKKDWRLLPRDREKKILEYHVDLGRLMSVGYAGFAFITTVIFVTEPILPRIINKFTKSNESVPLKFA
LPLEYIIFEKENHYWLMLIITNIFAINMIVVIISCDIMFIT
>MmedOR161_partial
NETIIYILPYRVPSIFEINDFKSYVIMYSFYGPFIFIVGSSHATLECFLITLVYCLRGRFVILADRINALNDKSEVGNNEVKDIIVEHSKLLR
>MmedOR162_partial
QHTKVANLNTTYHNSLVEFVKSLFSTAIFVQYASSSLLICSITYTLSHTETRSINFVGNALYVIAMTIQIFFQCIAANQVTVEVKYFYLNTNNYTFINYKTKKI
>MmedOR163_partial
SIKITRFCMSGIGMWHVEKPRDKIISNIVLCYTIATTIIALIVEGFDIYHCFGDLHAISYTAPCTITVVIELFKLTKFVINRSEVMAFNDYTFRKFWSIPYIDSERKILDDCNK
KSIKIIIAYITIVQFLVWQ
>MmedOR164_partial
TPMKFSRRRLEKLTADQNIINIKRNSINEENGIKSLVICIKMHKEILRFAQLLEENYSNYFFLLLGTIVLGLTVTGFQFVVLATEFGEKIRCLWYGTGQIIHLFFLSYLGQKLI
IHSEFINESM
>MmedOR165_partial
SAHTKNISGIIEHALYVFWSGFSLIIYCWLGEVLGKKSDDIARAVCEIDWSIKKKKKSLSLMLIIIRCRKPFAITAGKIFTMNLIFYKECILASVSYLSVLIAIGLGKLL
>MmedOR166_partial
MKHSGNSREFKVTPEKAVQFTKITVFLTCAWPPSNESRLFKLFIYSSVFLSFALFLPLVVSIIEYHDNFFIVMKSVIFICGITNYVTKVITVRIYRKEFQVCI
>MmedOR167_partial

DDDDDDYESNKIKNFVMFEMQENSVFDEDNFDWRLKHCVIHHQKVIDMLNVINDCFSFCVVVQILTSTILICLNGFQIILGNDSFHLLMRRFLAITVVLIQLLFWCWY
GNKMSSAAESLTINLWMCGWENEYKYGIRNTVSIPMILSLQSFELRALGLVPLSLQTFVSAIKTSYSVLILLLTVAKDQ
>MmedOR168_partial
MQVLKFNFFLLSLMGVWKPRGWSGIRAFLYNGYQVFVIIVNLIFILSNLMDFKLENFNLEAFADSLSLVFALSIVQKKINCVIENRTSITHFIDLLYKNPFKFQDHQEQLI
FSQFDKFARSVFTSYVVAHTGFLSIYSLGRMTLMDPPYTLPYNGWFPYNYTYTPKSYWMTAAFQFYAVFSLGVIDLLLDLLLPCIMCYMCGHIH
>MmedOR53
MVVLKDIFTILFYVGLWKPATWHGRKSILYTLYTSCIVIMASTFLITEVMDLIFVTSNIVEFTNNVFMMS
AVISSFIKTIIIIRHRKIIADIIDVLKIYLSKISGNEEIIIIDRYTRLIKFMNRSFLCTALFGVSLMVYV
ASSQNISQHILFYRAWLPYNYSQPMAYWMTTTGQVLTIYVLTIIYTVFILLFSGIMFNICAHINIFKYHL
QITFSDEYYHSRNDKRRCISKKENDKKIIHDCVETYLSIRRLFNTVKNLFSSIMLYQYSVGSIIFCTSAY
NMLQVEIFSAHFFSITLYMLNMMTELFIICITCNQITLEFQGVPNALYHSLWYVTDNNNRKSIVIMMSYT
LKPVYFTCGYVIDLSLDSFTNVLKLSYSIYNVLQSTF

>MmedOR52
MEYNQDFKYAVAWNRTSLRFVGLWPEPNDGFFTKLKGWLGAWSIFMTIYLPQSTLAYVNWGDMNAVIESL
SINGPILIAIIKIIIFRHYRDVLKLAIVTMTKDWNELRSKEEYKVMLKTAKISRIISVTSTIITNTLFIA
FVFFKIWIGMQLMKRTDLDPRLSVGLLYPGYLPFDSRIMTYFIPTWIAQCFATCFSMTAYAAFDTFVSCM
VLHICGQLAVIGVSLKNLINDDVKVDSKVFWIKFSEIIKHHEEINKLGLMIENSFNSILLPQMFVCTVTF
CLQGFAMITSFIDPSAGEISIFEMLFSIVYVFYTVMHLFVYCYVGDYLSFESTLIGQSYYKSKWYELPVI
KSRSLMFIGHRARRPLLLTAGKFCAFSRNLFLAVLKTSFGYLSMLLAVKQEKISDT

>MmedOR51
MKRKEKKNSQTKKKINLTVFDDIEYTQQLLSILAIWPLVTKSSTIKKILSWIHLLVIIGSLIWNILFRCI
FIYIYVKKFDDQIMLIGPTFFRVIILLKYLAIIYHRKTIKKIFNHIQTDRSGVECQEQQNIMMRNVQINR
HVTLVFAIFMYSSGTFYNFVMPHILPLLTHRGSRNTSQRLTIFPGYNVLSDVQKSPIYEITFFFHIFSVF
AGFSVLVLACNIAVVLVTHACGQIQIIIGQLNSLIDDFTNNEKTLYIKFSSIISRHIRVIQFSNNIIKDA
LYETCLVEIGASSVLICIVEFLLLKMIENQNYANMIPYAMLLASLTLSILIICYFSELLESQFIEAGIQA
YSINWYQLPPKARKYLILIIIISQRSYKITAGGIIDLSYIAFVQILKTGFAYLQLLRATK

>MmedOR50
MKFFNHPDWRIGKLMLCSFGGWPYQSQHSRRILNFISIFTVQSIFIPEIFRLTRIWNSMEMIVECVPMIS
LHIVANIKMFNCIINLNKVKVLLEDIERYYQSDLCKSELRILYKDRHSHKKVISVYVFYIYSIAVVFASI
PVLSKVLDLIAPLNESRPKVYLYPAEYFVDQDKYSTYIYIHGYMALPITMTLCTAYDFLYSACGHHVCSM
FKIAGSRLKNFIDNKIAWSKNESLDRNYRQDEVYKSLVECVKMHKFILNYVDCYQETFSDSLFLVIGVNM
LALCITSLQSLITMNQFHDAVSYIVFAFGQLTHLFLLNYQGQNIINHSENFYNDAYQTNWHEFSRKSRKL
YILIIMRSSEFSVIRAGKIYVMSSDSFSNVLKTAMSYLMVLNSLR

>MmedOR49
MEVHSRDQRDLDKGVRVFTWARLMSKCIGVWPLEPNYHLFNICFFYFTYIMITEYINLYYCLPNFKKVLG
NLVENLAFTHIYVRTLMLRIHIDKLRDIISESLKDYHASAYKNSDEVNEFLIYVRKGKFFVKAAGIFVIS
TATSWYIRPITSPSLPNNETAAFTYILPYKFHIFYKISNYRTYVLTYLSHAPFAIISGVGAVTSAWLLIM
LSFHVTGRLAILAKRINSLKDKNGGYRSHLDEIISEHSRLLEMGEGIKSSYAIALLIYFVNGTILLCIIG
YQILVTITLGVKHNLMPYFVFILTVYLVISIFCILSENLLAQSNKVSEAFWSCEWYKMPQDCVKDITFCI
LRSQKTLGLTAGAFLTFSNSTLTDVTKTAMGYLSILRNFLIVE

>MmedOR48
MEVHSRDQRDLDKGARLFTWARFISKGIGVWPLEPNYYLFNICIFYFTYIMITEYIDLYYCLPNLKKVIN
NLTESLAFTQMYVRAVMIRVHIKKLHHLMSEALKDYHVSAYKNSDEVYEFMSYVKRGRFFVKSVTIFILS
TTTSWFLRPITSSTPSTSMSAPDNETAAKFTYILPYKFHVFYEINNYRTYVLTYISHGPFPYVSVLGAIT
SAVFLIILSFHVSGRLAILARRINALNCKNEGFRTDLTDIIAEHTRLLEMGEEIKISYAVALLVYLVVGT
TQLCIIGYQILVLITMGKQHSLMPFFVFILTTYLLISIYCILSEHLLAESKKVSEAFYSCEWYDMPQDCI
KDISFCILRSQKTLGLTAGAFLTFSNSTLTDVTKTSMGYLSILRNFLLNEQ

>MmedOR47
MSEKATNSIITSKIVNSKYALDANRWILKILGVWHFAIDSSYFHKVIALCHIIICTFLLSFVVIPGILFI
FVIVKDVTTRLRISGVFSFCVMGVIKYYYLIKSNKQIGNCVKQFNSDWAQINDIKDKTIMVKYARFGRNS
SIICAAFMYGSCMFYACILPHVSGVFKNKNDPTERTFAYPCHFIVFNQYESPAYEIVFSIHCCCAFVLAS
ISNAACNLITVLITHACGQLEILMVWLNDLDSCQQNEVYAEKYSKIIKQHVKTIRFIVKIENLFQQICFV

EVVGCTLIICLVGYYVLLDWNQKDTGGMTTYVMLLISFVYNIFLYCYVCELLTAKCKLISESTYLTRWYQ
IPENFARGLVLTIAISQNSNPIKAGKLIPLSINTFGTVMRTSVVYLNFLRKLME

>MmedOR46
MKVNPSDQNSTVIQNQSSEPENIVNNEQDFEWAIHYHRKVLKFCGIWIYSNSNRWYLKLITDLHSLFIIG
GVLICLTTPEAMALVKIWGNLTLIVDNLLSSAALISTQIKLFVLWTRRKAIARIVEAVKSDWLEPKTEAE
RKIMRRYARIARIMMVCGLSNIAYNLITFHGSVLFGFVYRTVNNITDIEGYLIPTQSVFPFDITIGYRCW
IIRIVQALQCFGAGITYTAIDVFCGMSVLHNCGQLEILADKIKDLVNPDEPRVFQELLKTIVLRHYRIIG
LIEEIRNIFATVLLLLVLCFGILFSVIGFLIASSFESDGTKVPVSQMNFYIGYILFFVGLLFVYSWVGEN
LLSHSEEIHVAVYSCNWTDLEPHQIAQLIIILVRAQRPLEITIGKFAPATLNTFAQILKTSAGYISVLLA
RNG

>MmedOR45
MDVFDSMHWRTTKKLLSAIGLWPFQPVIQRIVVGAIVYFIIQSIFICVFLKLIVAWGNLAETLYSVPILV
YFSMIQVKITNCHLNLHKAKFLLLRVKRDWESKLEDSEFEILRNDGRIHKIIMDVYFSGLICVATVYIVL
PLMSPLLDIIIPLNETRERILPYPAEYFIDIQKNFFMLYPHGAIVTPIALTVLVGFDSLYAGFVQHACSM
FTIIGRRLENLTVDRNNIDEEKNSLNERHGLQSFITCVKMHKDILQFVKLVEKYYSNYFFVLLGVIVVGL
SAAGFQFVVLLSGVGEKVRCLWYAMGQVTHLFFLSYVGQKLIDHSQVINASLSAAKWYDYPQKMKPLIIL
MLMRGKRVSTVSAGKIYVMSIENFSSVIKASMSYFAVLTSMES

>MmedOR44
MENDQKELDEGVKAFYWAERMSRCIGLWPVTPNYYLFNICLLYFSVLMVLELIDLYNSVYDIDKLIDNFT
ENLASTHMYARILMLRVHNYRIGEMITQAMKDYRISAFKNSYEIKVFMEFVNKGKFLIKGLFIFIMSTEI
SWFLKPLTTPSSSDNSIVNANKTFPQFILPYNVYIFYEVNSIKRYVLTYLSFMPMVYVSGIGHSAVDCIL
VLLVFYISGKLSVLTMRIDALKNNQYDCRKELKEIIAEHSRLLKMGDEVKDVYSTGLLVYLVNGNLLICI
IGYQILINYMTGPNSDLLQYFVYIGATYFMIANFCIISEHLTAESNKVCEAYWNCEWYNMPQDCVKDIIY
CIVRSQRPLALQAGKFSTFSIVTLTDVTKTALSYLSVLRNFLIAE

>MmedOR43

MSKSRVSDYKAYRGLTKTLLTVGGLWPYSNSNIFYRTLPYIQIILNLGMALAILGFVRDHFSNIALVTRG
MGVMTSFLTTILKVTCLVINKKDLMELHGNLDPYLNDLLKNSPSTEVILKDINSFKFLSWGLTLFAFIIL
HCHQHEIELINYPLIYPSVYPWKIISNGWIYKMNYVFETLASLILFFVTASVDSLFTLYVFQMVGLLREI
SYSIRRLDEKNVNRDSVICKCITQYEKLMRCREILEKIYGPVILWIMTTNAVVLCTLLFQISQMKSISVT
RGLLFTTYITLKMIQTFMYAWSGSCLTDESENYKDAVYAAHWYGNKRFMTSVIIMLAQRPLTLTACNFST
VSLKIFVMVLNTTVSYFFLLQTLDYQE

>MmedOR42
MAILERNIFILTLVGVWKPNHWKGFKAALYYFYICLVTIVNHSFLLSGVLDFELRNIDVVVIIDNLSLLI
CVFTIRYKIITLLYYRKFIEEFVNCFERDPFRAKDNEEEQIYVQFDKRTKTLSILYAGLFTVAVSWYSVG
HVLRMSPPNVMPYQGWFPYNYTVYKYYWPTVIYQLYAVCSGAWVNLAYDSLFCSILYYVCAQTHILKHRF
SVLAENLQKINEENDGNDNKEIERKMIGDWVDYHNNILDLVKFVKSFFSTAIFVQYAASSLLICSIAYTL
SHTETRSLNFAGNFFYLIAMTIQIFFQCIAADQVTVEFADITNALYSTNWYNLSNNAQKSLAILAEPLKP
TLINSGYFVILSLDSFTKVIKLSYTIYNVLE

>MmedOR41
MKQTATKEGLGRKGFSVTPETAISFTRITVYLTCIWSPSTSSTKLTFILFEIFLWFSIFLSLGLLFPLIV
SIIKFIDDTFVVMKSFILISGIVNFVIKVIVCRIYRQELQELGASLNDFVRNSNENEKFYLQKYVDKCWK
FHGYMTCSYYLTTSAVLMGPLVLPEKFPSDAVYPFPVDHPVVATIVYLHQCIVGYQCAAGMALDCQAALF
LWYLSARFEILISETVNIASHKEIQDFIKTHQDILKFGKQVIRPIRLIVLTTVTMTKVGMIFGAIVLISD
EPITVKVQFAILVVSATVNIYVCTWAADNLLTVSSSTISNEIFHVSAIHPPAMKKLWLTVIHRALKPITI
EVPGFLETLSNEFYSNFLSTAFSYFAAMHAVVNS

>MmedOR40
MSMQYSQDFKYALAWNRTSLKIVGLWPEDDDGIITKSLGWFCACLIIIIIYLPQSASVYVYWGNMDAVIE
CLSVNGPVFITIVKIIIFRYYRRVLKRVIDTMAEDWSSSRSNEEYAVMLKTAKISRAISVTSTIITNSLF
VAYIFFKIWEGLEMSKRTDLDPRLSVGLLHPAYFPYDTKKIKFFVPTWIAQLVATLFSMTAYAVFDTFVS
CMVLHICGQLAVVGISLQNLINEDTNSDPNYFWSKFSKTVERHEKLNELANVIEDSFSSILLPQMIICTV
TFCFQGFAMITSFIDPLAGKVSILEMLFSIVYVFYTVLHLFVYCYVGDYLSFESSIIGQSYYKSEWYKLS

QDKSRSLMFIGHRARRPLKITAGKFCAFSRNLFIRILKTSFGYLSMLLAVKRDKSA

>MmedOR39
MTAGMIAARIDIDVFLEAIPTVLADVICGIKMFNFSVNAAKMKKLLLTMEEDWKIYASGPENKILNEYAH
FGRKVTIYYTGALYGTLAPLVLVPITPLILDVIAPMNESYPKHLMFQQIEFLVDADKYFFPLFIHNYMGT
VAFLTIIIAIDTMLMVYIQHGCAKFAILGLCLERIATNADQNIDRHTSEFDDIDYREIVKCIVIHNRAIE
FANLIEEANHLSFLIVIGINIIMMTTSALVAVFKLAMNETEIAGRFAFFTLGEICHIFYSSWQGELILKH
SESIFYFVYQANWNNTSTRSQKLMVPLLLRSAIPCRITAGKMFEMSLKSFSMIVKTSFSYLTVFASMRV

>MmedOR38
MKTLPYSFMILEYFGGWKPLKWGTSIKGKLYNMYTLTVAVTLVTFCLSCIIDLFYTANIEDIVHNMSMSF
TLIVVCSKLTLVTVKRNEIIRIIKLLDINICRVRCAKEANIQLKYDEKAKNIVKKYGGLVCGAVFAVTGA
SILENIPTKTLPFNGWFPYRHDNSTGFWVAYFHQNIAHFYVAMIGFSFDTVVYGVLLQNCSQLQILKNRL
ENFVEIINEEKLKDKINGLSRTIRECEHKFIKQCTHHHWIILQFSQESNDLFAPIIFLQYLSSSLILCLC
VQLLTKLAFMSPEFIFIVVYLGCMLTQIFLFCWYGNEVIMESSEISSAIYKMNWQVLTNKTKKDLLFMKT
QSILPIKFTSGHLIELSLDSFTKLIKFSYSAYNLLHQK

>MmedOR37
MEVLTFNFFLLSIMGVWKPRGWRGIKAILYNINRSIVVIVNHIFLLSGILDLEFKNVDLDAFVDNLALIL
AMVVVRQKIVCVIQNRTGVKHILDSLAKGPFKLRTHQEKLIFSRFDDFARNIFTYYPLVFMSSLLTYSSG
HMAVMDPPYVLPYKGWFPYNYTRTTKIYWTTAIYQLYAVFTTATINLILDLLLPCIMCYMCGHIHILKHR
FKEMIEKLLVMSENNVPQEKIISTERKLLGEWIEYHIDILRLVKFTNELFSSVVFVQYTVSALLLCTIAY
LMSHTDTMTMSFAGNLAFFTAMFIQILLPCYCADKLSYEFLDISTGIYDTNWHYLSNNIRKSVVIILRKS
YRPVIITSSFFIVLSLESFTKVVKLAYTIYNVLE

>MmedOR36
MEILTTNFFVLSLVGLWKPRGWSGIKAILYHFYSSIVIFANVSFLISGIMDLEFTNIDIAAFIDNVSLLL
SLVTIRQKTACAIGNRGDIKEIIDSLGRSPFKPQDEEEENIVKRFDDLTGYILKYYPVLFTVAITWYSIG
HMFIMDPPYVLPYRGWFPYNYTTTGVYWLTAVYQLYAICSAASINLAFDPLLPCIICHMCAQVHILKYRF

GVMLKKLEVISDNEPRYVIASAERKLMGEWVDYHISILNLIKYVNSTYSKVIFVQYTASSLILCTVAYVL
SHMDALSMNFAGNFFYFIAMNFQIFFQCFCANQVTLEFLDITTALYDENWFNLSNNVRRSMTIILCEPFR
PVLFTSSFFIVLSLESFTKVIKCAYTVYNVLQ

>MmedOR35
MAILKESFCVLTGIGLWRPVEWQGIKGAFYNCYTLLVLLNSITFIISESMELIFFNDGIFDFFNNSSMLI
TVIGMCGKLITVVTNRETIIKMIERFHRSPFSPRDYEEEIIHKNFNQKIRFNTLAYIVFFEASVTVYTVG
KIFEDRPPGVLPCRAWLPYDYSNNIIIYWMTASQQLLTVVMTANVDIAYDTLFPGMMMQVCIQINVLKHR
FRLTLDALENISDDKMDPVMVKTVEKKFFSEMVDTISHVFGPMIFIQYSFSSVVLCSSVYALSQMVPFSP
EFCACSVYILCMFFQILYICLSGNRVTLEFAKLGTAMYDTYWFALSNNAQKHIIMMMMSSVKPIIFASGH
VVTLSLESFKRLLKLSYTIYNVFQQSS

>MmedOR34
MEDLTQEQKDLNEGAKMFDWGKWISKGIGVWPLAPNDYLFTTTFLYFTAVMTLEWVDLYTCLGDFEKVVD
NLTENLAFVHIYVRTLMLRVHIDKLRDVMTESLKDYRTSAFKNSTEIKLFMTHINKGKVFAKVVITFIAM
TEVTWYLQPLTTPSPPVDNRDNETISILLPYHFYVFYEINDFKTYVLTYLSHGPHVVISGFGHATSDCFL
IILVFHLSGRLAVLAERINALKNKPEMNGIQIKSIIAEHIRLLKMGENIRSAFATALLAYLFNGTILLCM
IGYQILVNFMTGPNSDLMQYFIFILATYFIITVFCIVSERLIFESTKVCEAYWNCGWYNMPREHINDIMY
CIVRSQKPLALQAGKFAYFGNSTLTDVTRTAMGYLSVLRNFLIVN

>MmedOR33
MQVLTFNFFLLSIMGVWKPRGWHGIKAALYYIYQTITIILNHLCLLSLLLDLQFKNIELGDLIDNLALVF
TIIIIRQKIVCIIGNRPGITHILDSLKNSPFKLEDSKEEFIFSRFEKLARNIITYYPLIYLSTLSVHSTG
FISVMDPPYTLPYKGWFPYNYTRTTKTYWVTAVYQIYIVLTMGSINAISDILLPCIICYMCGHIHILRYR
FQVMAEKLRIMSKNNEPKDKIISTERKLMGDWVKYHIDILNLVKFTNEIFSSVIFIQYTVSSLLLCTIAY
LLSHMEPTTMRFAGNSAFLTAMFFEILLPCYCADKLTFEFLDISTGIYDTNWYHLSNNIRKSIVIILRKS
YRPVTMTSGFFIVLSLESFTKVIKLAYTIYNVLE

>MmedOR32

METTATKNDNTNQNKNELQLTKTNKYENVDYKSDADYAVVVARTLLTPLGIYPLHGSDTSLSKFLIAIQI
IIVFGLMLFLLVPHFIWTFFDAEDLKKLMKIIAAQIFNSLALIKFWTMIIHKKELRNCLIQLENNWKNVL
CEEDRVIMIKNAKIGRFFTIAYLSLSYGGALPYHILLPLTAERIVKEDNSTQIPLPYPTDYVFFVPEDSP
GYEMLFVTHIIISTMILSTNCGIYSLIATYIMHACCLFEVVCRHLDEFSKNNTNNFKTELTWIVENHNRA
IQFAETLESSLNIVFLCEMVGCTVIICFLEYGVIVDWEDGKLLGLVTYVILMTSIFVNCFIISFAGERLK
EQSIKIGESAYFAEWYLLPKGLVYDFMLIMIRSSKPASLSTGKVSDLSLAGFAGLVKTSAAYLNFIRAVV

>MmedOR31
MLFKATPEFAIAFTKLTSILGSSWPHYKNATKCQLIVFNIKWWFFWFMSITAFLPMCYAAYNNTKNILSF
TKSLCDAANCSQAFIKMLLCKIHYRKLQFLFYEMEKYVEQARANERELFISYIKRCGRLHVSIMISAVMA
AVIIIIAPIGMPQPFPNVAEYPFPVDGHPTFEIIYLQQSIATIHCMSIPVFDCQIALLLWYAGARLELLG
DEFRKVTDNQQFVACVKKHQYLLWFIQEIIMSSRHILATTVVMCTIAVITSGVHIVGKEPLADKVTSVIL
STGLSAVLYLCAWPAEHLAQMCENVGAALYCSTWIKNSKESNKNIFIVIQRSQKPETIQVPGILPILSLT
YYATFLSKTFSYFTTLRVVLDKMED

>MmedOR30
MLFKATPEFALKFTKLIALLGTSWPNYEGTPKWKLVVFQIRWWSTFFLAITACLTMCYAACNQYQNILNL
TKSLFDISNTSQTFVKMFFCKVHYKRMQYLLCDMEKYVTKAKPHERDLFIKYIKRCGKLHLTVMGSGLLI
IHIIILAPIALPQPFPNIAEYPFPIDGHPTYELLYLHQSCATIHCLSIPAFDCQIAMLLWYAGARLELLS
EECKTITDNKQFVECIKQHQYLLWYIQEITTSSRHILATTGCTCILTAISSGVHIVSNEPVAFKVPFMIS
WVIVSSTLYITSWPAENVLQMCEQVGMALYESPWVQNSKELNSSILFVVQRSQKPSTIEVPGILPVLSLR
YFAMFLSRTFSYFTTLRVLLDKINLDMEIPAED

>MmedOR29
MYHKLLNKLVRILRYNGIWPVESTVRSYKLLNLIFRLFNLSIIVIMMLLTIADAIANFNDISLITDNLCF
FVGCSEALTKGIKYCIEYKNIVKLMNDIYGPIDIINKKNNTEVMKGINEIARFENRQFKIIFGIVSLLIV
ARVLGADFKNKGFPIRALFPFDATATPYYHLIYLLISYGVLLVDYTLLGVDLMVVVIMRYLTIQVDILRA
NCRHCDIESTRRNIVINGYDDKNNENTDIRNFVGFEIEHEDSDGKDSFDDRLKRCIIHHQKVIYMLNGLN
DCFSFCVVVQILGTTVLLCLNGFQIIMGRDIHLFMRRVLASTAALLQLLLWCWYGNKLSAAADSLTINLW

MCGWEDNYKHGLRNFISIPMTLSLQTLELRAIGVVPLSLQTFVSAIKTSYSVLVLLLTVAKDE

>MmedOR28
MTRSLKLQDQETFDQVAKVLKWNKWLLSTLGLWPQSPNTFIFTVNFSYFVYHMAMEYLDLFLFIDNLEHV
IENLTENMAFTQILVRIAMLKKYNRQLGEVVNEAFKDYDARIYRTDEERQVFIDYMKKAKLFIKLLCAFV
TMTATSYYAKPITSPPPPPEGELDVEMENATMSFILPYRFHLFYQVNDSRTWALTYLSHFPFVFVSGFGQ
TAADCLMVTLVFHVSGKLASLAIRISEINTEPGVCKQELRSIIIEHDRLLKMGQSIEEAFSETLLAHLIG
ATSLVCILGYQLLVNYARGQGADLVTFFVFIFLVFLVLYAHCVVGESLITESFKVCEAYYDCLWYKMPKE
SSKTIVLCMARSQKPLGLTAGKFGAFCLSTLTDVVKTAMAYLSVLRTFLVIE

>MmedOR27
MKFFEQSYFTLPRNFARSIGRWPYQSSLQSFLIGIVIISAFILQVGPKILADIVHSDDQELILETLAPTI
TNVMAFAKYINTFVNARMLKILFERIKDDWESVTDKKEKIILESYAGFGKLMATGYAGFVYAATVQFITE
PVLPIILNNILRTNLSAPHKFADPMEWIIIDKEKYYWILLSNSSVCIMVILTVLISYDVIFITFVYHACG
LFAITGHRIENLPHDENFKIINRNTNSLKNSRDVHYKHLVSCIRIHRMALKYVDLIESTFAGCFGVVVGL
NLPLMSITGVQGMKFFRLLHNRMTLQQKIKYVMFTGAQMLHLFFECFLSQQLTDMSLRVQQHIANGNWYD
ISTKSQKLLILMTMRSQVPCILTAGKIMELSVESFGMMMKTSGSYFTVLLSMQ

>MmedOR26
MDIFDAPYYRIMKNSAKLIGQWPYQSREKRIIIIIIVWAFFFMQFVPQIIAIVVHIDDPDILFEACSNMA
VDFVTAIKYINTICKTNLIKKLHDRVIIDWSLMLNDEEKSTLEKHTNLGYLFSSGWAGFAYMSATIFVLE
PVFPRILNVFISVNATDPFKLALPLEYIIIDREKHYWIMLFVSTVFVYNIIIVLVSCDIMYITFVQHVCG
LFAVVGCRLVNTPINENYSERHKAGDYLSNSKDIPYKHLVSCIRSHRRALEFAELLEDAYCISFGLTVGL
NLPVISVTGFQIITQFNTIQQLLKYASFTITEILHLFFECFMSQRLTDMSLEMQKSIAEVQWFDNSIKSR
KLLIIMTMRCQVPCKLTAAKIMDLTIENFGMMVKTSGSYFTMLLSMQ

>MmedOR25
MIFKATPEFAIAFTKFSTLVGTCWPNYKNAPKWKFVLFQIRWWLTFCLSVSAFLPMCYAAYNHWRNILSF
TKSLFDAANTSQTFIKMILSKIHYKRLQYLLYEMENYVTNAREDERELFIVYIKRCGKLHLFVMIFGFMA

ILIIIVAPIGLPQPFPNIADYPFPVDESPAFELVYAHQSAATLHCLSIPVFDMQIALLLWYSGARLELLA
REFKTVTDNKHFVECVKKHQYLLWYIQEIIISSRYILATTSVTCVIAVITSGVHIAGNEPVGFKIPFAGA
SSIIAIILYISAWPSEHLIHMCEGVGTALYESEWVQNSKALNNSMLIVMHRAQKPSTIEVIGVMPILSLP
YYATFLSKTFSYFTTLRVLLSKVEMD

>MmedOR24
MELDNIKLRHLEPYYNIKVSLTLTKYIGTWPPVLEPYRSIYLLYTCVSFIFILGIYLTVQTVNLFVIWGN
IELMIATGFLLMTNSIHAYKVFVILGNQKRIQVLLDKLSTTNYYHNDDKYERVFTYYAWQGLYHHIAYQS
FGTVAVLCWGLTPLADAVAGNTRRLPMEAWYPYNTKKNPAFEITSGHQAVAVLIACVHNIGMDTLVTGLI
NAACCQLEIIKQNLKNVDLDFEYQIDKCDYEDFMNKQINKIIKHSNEIYK

>MmedOR23
MFKINPEFAIAYTKLTVTLVCSWPPGRNSSRLDFLLFRIKWWISWLMGIFLVIPLIYAAYIDRRNVLEFT
KSLCLAVSCGQCAVKMFFCKLQHHRIKFLLDEMEEYVKVAEPFEREIFLGYIKNCGLVHVTLNVCSLVAS
VGVILGPLVLPQSLPTEAKYPFSVENHPNYEIIYIHQAFAGILCSSIGSIDCQIAMLLWFSIARLELLSL
EMKNITNVYQFHNCVRKHQFLLWFVDEVIKAGRNLVATTVIMTTFAVILGGVHIVGNEPMLVKLQFVIIV
GGFSMLLYVTAWPSEILTRMCQNIGWTIYNSEWIRNSKELNKGIEFVIQRSNKPAVIYISGIFPAISLNY
YATFLSKTFSYFTTFRIILAKLE

>MmedOR22
MDNSLDSFLRINRLFLSSLGQWPQQEQISKIFTLINAVFFLITQAYFQTGGMIAAKCDQPIFMESIAPVL
ISFMCLVKFVNFNYNADKMRKLLEIIQADWNSINDLEELKILNSWAKDSRKNTIMYAGALYGTMAPFMLG
PLVPIFCKLMPAGVLPANSSIVLEKPVLFHVEYFYDLEKYYYPLLIHSYFGTMAYMTVAVAIDSMFMVYV
QHACAIFAVIGNRLEHLADDSSINFYYNPHILNDEPYKRMIECIVQHSKALQYAQMIQSANSLSFFFNWD
SICSLLLSVDFNWPSQRLADESARISETTTRCAWYLTSMRSRKLLQLFIMRSSVPCQLTAGSFYTLNMQN
FSAVVRTSMSYFTVLTSVQ

>MmedOR21
MDYLSSPYCRLNKILLSCLGEWPYQTSTQRRFIRSTIYFFSASIIIPKIIKLIKVWGNLDMIIECIPMLL

LDAVNFVKVVNGFINFRKMRELFDRIQDDWGLNYSKREFEIMQNYAEDGKKLSQFYASYMYATMLIYFCM
PIIPKVLDIVLPLNTTRPELYLFEAEYFVDQHKFYYPILIHAYITCAVAVSMLVAFDTEYAIQALHGSGI
FSALRYKLENLVIKDDEADYKNDEKIKQSTYNMVVQCAVLHKRALDYADLLESSRVTCFFFVLLVNIAAI
SITGVQTVMKLDQPTEAIRFGVYTLAQITHIFYNSYPAQMLFDNSWKTSDAIFAGNWYRAGSKSKNLLHM
MIMRSRIPCKLTAGKIYLMSLENFTGVVKTSMSYFTVLLSFR

>MmedOR20
MENTSIESRQSVLSMAIAGLRLCGIWSLDSSSPIFLKLLHNISNIFGIITLIIFVGTLTIDLLLNSNDLL
IATDDGCYLAGISVIVFKVYQFHRHHKRIKNLTDATYQPIYVFWKSTDIGVKTVLRTNKFYEDLGFTFFV
SLGGFLVIALIFFVPTEEGALPIRGAYPFNTTISPMHEVAFCLQIYAVTYGLMVIVLMDGMGLGIMRWLN
VQCIILASNYRNCRTNQNNSFYLESRDDLSKIASIEDDNNNVTDIYDEPDSNITTFCPFDEQDHAGMSDC
FIGRFKKCIKNHQRLLNTIDELNACFSSCMLMQLFASFSMICLTGFQAVLGATTKTSLIKFVLYLGAAFS
QLLYWCWFGNELLYEVFILPHD

>MmedOR19
MDVFEEPFYKMIKNFSHLIGQWPYQSSRKKFTIVTLIWIAFFMQFIPQIIAIVIHFDDRDVLFEAFSSMV
IDFAFIIKYLNAIYRAGLMKELWESIRRDWTLLLNDVEKRTLQHHANLGNFFSMGYAGLAYMSTTIFVTE
PIFPRIVNIFVETNETIPLKLALPLEYIIIDIDKHYWLILTITNIFVFNIIIVIISCDIVLITFVQHVCG
LFAVVGCRLESTPFDENYLEGQKGEDFLSNSNDIPYKHLVSCIKGHKRALEYAERLERAYTLSNGIVSGL
NAPVMSITGFLMITESSTIEQLLKYATFAISQMSHLFFLCFMSQRLADMSLRIQENIGNATWYNNSLKSQ
KLLVLMLMRSQVPCKLTAAKLMDLFIENFAVVVKTFASYITMLLSM

>MmedOR18
MYSILPGSFVVLQAIGLWKPPEYNNSPILNYYYRLRTFITFFLIYSFTITGITGLILTTKDIADVTSDCF
ILLSIFAICGKIANIIWSRNEIIWIIDTLNSEPCKPLNNDEIIIQQKVDRLIWHSTLFYGILTEITVFMV
TFGTLLLQLPIGTLPYNTYLPWDYSHGYLYWVAYGYQIISVCLSANSDIGFDTLVPGLMLQITAKLEILK
YRFINLVDTLKLTQWNGVNDKSYHNFRIENKLIADYVKCHLIILKLADTINKTFDKVILLQFFISSIVLC
ISVYNLAFLDVFTTEFTSIILYLCCMLMEIFILCAAGNQVTIVSSTLSDAIYHTDWINLDTSAVKSLMII
MNRGLKPIIFSSGHIIKISYDSFKTPIKLSYSSYNVLQRT

>MmedOR17
MENIPIDIHRKFLNINITILRYSGVWPLLPTAKIGWKVFNFIYRIFNLTVFIFYLITLGADAVTNYKDLT
IFGSDGCYFFGTCMCVFKACKFWASYHKIIKLIVDVYDPIDVLVRSADPGILMNIKKSYYQESIAFWGFS
TLCSFFHFSVIFLIPREKGILPIRAIYPFDTKISPNYELAIIYQAYCLAYALCVTIALDITTIGFIRWST
LQIAALTSNYKNSNPNVTKRASLVTSSSDARKIIEKLNKIKITDDDVEIETFLPLDYHETKYFINDLFLS
RFTTCIKNHQRLIKIIRDLNAVLSPLMLVQFATSTCIICLNGYQMILAETMTYNQWMSGWECAYGKVKSNELRNLVTIAMMPAIKPFAFNAVGLFALSMPTLLAVVKSS
YFMLILLTTVTED
>MmedOR16
MKLPAHTKGYKVTPEGAVNFIRVTVYLTCISFPLTEKTKVRINYEIILWLSIFLSISLFAPLLASIIKYS
DDTFIVMKSFILMSAISNYVIKVIIVRIYHKELQQLGSALDEFIKKANESEKVILQRYVDNTWKFHGFMTCSYYLTATAVLLGPLILPQKFPTDAVYPFPVDNQIISYIVYL
HQCIVGYQCSAGMALDCQAALFLWYLSARFEILISEAKNVETFDELRNYIKKHQIILLYAKELIRPTRLIAFVTVVMTKIGMIFGGIVLISDEPVVIK
IQFAILVISTTINIYVCAWAADNLITVSSTAMSNAIFEISWMHAPKLRNFLQTVIHRTQKPVVIKIPGLL
ETLSNEYYAQFLSAAFSCFAAARVVVSS
>MmedOR15
MEAFRLHLFFLSILGVWKPQGWHGIKAFLYSIYGSTVVIFNHIFILSGILNLTKFKHVSLDVFIDNFSQMLALIVVRQRIICVIENRNSISQIIESTDKYPFKLRDRQEKLIFS
KFSKLAKNILIYYPIVHMCIILVHTV
GHISVMDPPYALPFQGWFPYNYTRKTKIYWATATYQLYAIFSEGSIDLILDLLLPCILCYMCGHIHILRH
RFGVMTEKLQIMSENNEPREKIDSAERKMTAEWVEYHIDILRLVELVKKIFERMIFVQYTVSSLLLCTLAYLLSHTKCTTMTFAANFSFFMAMFIQILLPCYCADKLTFEF
LDISTGIYNSNWYQLSNNIRRSVVVILRNTYQPVTITSGFFIILSLESFTKIIKLAYTIYNLLE

>MmedOR14
MEKKTINDHTSIKLMRLCMNGIGMWSIEKRRDEIISNIVICYTIATLTVGLIVETTDIYYCLGDLREMSY
VAPCLLNVIVELFLMGTFVINRSEVIAFSDYTTREFWSIPYIESERKLLDDCNRKSVKIIIAFIVVIQLV
VWQYITIPIIESYGKNASERTLPYNLWFTFIPFKETPYYEICFFLQSAATLTTGVCATAFATFLFTINLY
ATGQFKILQQRLESSCQVYNIEKIKSVEQINLIAEESYANLRKCVELHNVLLKYITRLENLYCQIMLVET
LACVFLICTTGFQIVLGVDSSILRTSRSALYFCCLVTQLLLYSWSCHEIIIESLEVAEAAYRAYWYSLSW
SKYGKSFRQALLIIITRSRRPCVLTVGKFVPMSLETFTAVFNSALSYFTILRQMTEEMENS

>MmedOR13

MEEERSRDERDLESSVRAFYWGKWMSKWIGVWPLAPNFYLFNVTFAYFTAVMLLEYVDLFFCLPNFEKVLDNLTENLSFTIIYVRTLMLRVHNYKLGTAIRECLKDSS

VSAFRNSKEIDIFVQYTKEGKFFAKFVIAFAAMTETSWYLRPITSHTAIRVIADNETLNNVTLKFSLPFHFYVFYEINSIKTYALTYLSHGPFVPINGFGTASANCFLIALSF

HISGRLAVLAERIKTLKDNPDSYKRELKLIIDEHIRLLRMGEDVKISYGVNLLVYLLNGTILLCIIGYQILLTLTVGPRTNIMPFIVYIMTMYMVISIFCILSENLIAESNKVC

EAFWACGWYDMPPDCISDVYYCIARSQKPLALTAGKFLTFGYGTITDVTRTAVGYLSVLRNFLLLEE

>MmedOR12

MIKRPIETHRKYLDKCIHCIRFCGIWKFDSSASTYHKLMNLFGKIFNPTLLIFHFLTLFADIVANYNDLI

IIADDGCFLAGSFTVCFKAYEFHLLNNIYMKIINDVHDSVDVLQKSCDLGVLTIIKQYIFFETLDFFLLI

YVVTVLGVGLIVLLPLTRGGLPVRAIFPFDVTKPLMHKIAFFIQAYNISFGLVTIVALEYLSWGLMRWTIVQLKVLSSNYRNCNSDKVPIASFNVTKNTYNKIKNFNILK

VDDEDIEIHNFIVFEEKELNSINDCFNWRFRTCIRHHQRLIKIIYDLNDIFTVSLLIQLGVSTFLMCLNGYLAFMFPHDNQRLIRSVLYLVAGFVQLLYWCGFGNELKFQ

ANDLTTSQWMSGWEDKFDGGIKNLVTTSMIRTMQPLEVRAGGLFILSMETFLSILKTSYSVFVLLTTVSDEE

>MmedOR11

MQNEKEELPSCEKLFSLELAALKFVGLSSLRNGFRKENMNIPVKFYEIFLFICAASILSGFFISSALTVH

MLLQSDFILACEIATFIFAGAVTVSKVLRIWSYRIELIEILRELNELWEKIVKHRLNLKENILNMLNESR

PIRYGYCFIASSLNLSYALRPYFHMLVYFVKQSENKTIDLTVTTYPLLYPIERGTWLGYLLCVTYEQSIL

YFGGIYWIMCDTLFILLTSHICVHFMIISNDFNNLHINYDKNNNESFQLIEDLSRRHQKMFVLCQRIETLFSPIILLTVVFNGIDLCLCIFALDKDLSDGNWAKVASSVTH

ALTLFFQIVIYCEFSHVATEETSKVGEAIYNSSWIYFDKKMKKMLLIIMMRASKEYKFSVFGILILDREQLTQIVKTTMSYFTMLRSFS

>MmedOR9

MYSSNSTQVIADFFKQPCYRVNVLTLRICGLWPYQSKFEGKLLRFLWAFFVSSQMIPQVCVCITDFGMDTLTSTIPPFTVAIIAGAKMIISTLKVNQIIELLVTIQKDWSKL

KSETECDIMKKHLDQGKKLTLFISAWYYNSMVVFLLLPMRPKVMTWLGLSKGPADFDFPYPVNYGVDHDKYFYAIETHISFCSTLVITTIIAADTLFIVFVQHICGVFKII

SYRLENLVSSSSLDIDLHPNKINDRAFWVISDCIKKHNKAIQFAELLENSYCWIFFISVGFETLLMTFSGVQLVSQMGNIDGLFRYGPFAFGQLVHVFIENAISQQLINYS

GEINDAISMIKWYTLSMRSRKLLTFMIMRSQVICSVSAGKMFIMSMETFGMILKTTMSYFTILSSMQDD

>MmedOR10

MSTENTQLDADIEYNLKLTKWILKPLGVWSIVTKDYRFISKLSSVFIIILNSFIMFITFMPCCVHMIYRE

TDPAVKIILMGPFGFCLTNCCKYYFIIFRSNIITHCLENLKSDWARPKTKQDRLEMIKNVNIGHNITKLCAIFMFSGGVSYHTIMPIWSGSTVNEANETIRPLVYPGNEIFV

NCQETPIYEFIFILHLTCGMVMQTITTAACHLAAVFASHVCGQVDILKSQLQNLVDKESLKIDGTIENRIASIIQSHVRILDFSTNIEKMLREICLVEVGASTLIICLLEYYC

MTEWSNSETINILTYLMLLVALSFNIFIFCYIGELLKKQCNSIGESTYMINWYKI

PRNKGKQLMLIIASSNNQRKLTAGGMMELSLRSFGNIIKTSVAYLNMLRTVTE

>MmedOR8

MDVLQENFSVLFYLGVWKPLDCTGIKSFLYNLYTLFITSISYTFLLSQILDLIISTKTVSDFTNNIFIVS
AILTGCLKIFRFIRSRNTFINIINNFKRGLFKPANNDEIIIWNKYARITRLVTIGATTSLIIGLIVMSYA
LCSFNIPQRQLLYRAWLPYNYSSLPIIYWLSSMEQLATVHILAGINFSFDLIFFGTMLNICAQINILKLR
YKVALSHIYSINDSINNNDLGELRDVSKLIREYTDSHDSIIKLFNSAHHLFSTIVTIQYCTSSVAICTSA
FNVTKMKFFSFQFFSTALYINNVMIELFILCVSCNEVTLEFADLGNTFYDCQWYAINNANKKSVAIMMTNTIKPIYFTCGYVIHLSLDSFTSVLKLSYSIYNVLQSAD
>MmedOR7
MDIYDKPYYKITKISASLIGRWPYQSSRQSLVIVTVIWSAFILQAIPQIIAIVTHFDDREVLLEALAPFI
IDIMFVAKYMNSIYNAELMVTLFERMKKDWKLLSSAKEKRILEYHANIGRLISTGYAGFAYTTTAIFLSEPILPRIINYFSKSNESVPLKFALPLEYIIFEKENHYWLMLAIT
NMFAINMIIVTISCDIMFITFVQHVCG
LFAVVGFRIENSPTGKITDSNHRAVSLRKNSQDFSYKHLVSCIRSHRRALEFVKLLEETFTGTFGVVVALNLPMISITGLQLITQSNTVEQTLKYLMFALAQVLHLFFDCF
LSQNLTNMSSRIPQCIANMKWYNISKNSQKLTLLMTMRSQTPCKLTAGKIMELSIENFGMMMKTSGSYFTMLLSMQ
>MmedOR6
MTLIESCWMVFTWLGLFRPIKWKGLKARIYDLYTAIVLFFNYSFFICGVMDIDFTHLNFFADIDLITLMLQYIENTPKILCMILNRNALIEIDFKLQHDHFKIKDEDEKKI
QNKFDKFSRYVLLAYSALQATSLVYYTTGRILAMESPVILPYRSRIPFNYSSSGKIYMLTALDQLYSVSSLICINGAFNLVFTSTMYQICTKIRILKHR
FKVIIQQLEHDGELGNNDNKNLRDVMRKNDATTDKFESQLIANWVESHIALINLYDYAKSVFAKAVFIHYVINSIVMCTLAYILSHCEIDNIFFGNVCYFSVKCTQQFL
QCSSAHQITLEFEDLRDMIFSTNWFATKITIQKSIIIIMFKSIVPIEFVSGYFVTLSLDSFKRILKLSYTIYNVLEG
>MmedOR5
MDFWGNSDWRFLRFQLCTLGVWPFQKSNFKRVVGFFVILSVQSITLPEVIKFTYIWHDMEEFADCFPLIGIHFVCTIKWMCCVVNMDKIIALLNMIKSDNLSKELTEEE
HQILRDTGKINRLFVLVYSIWIYVIAILFLVFLPLIPVTLDIILPLNESRPKIELYHTDYLFGPVKYSWVISLHQCIISPFPTIIIIATGSLYCNCCQHAC
GMFEVIGYRLKNLDITIEAAMNKKNLGYYADTEIFYKSLVTCEQMHQRMLGYVEKFQDIYSLTLCFSMTTSIITLCITGLEAIIKKDQFFEVIRYVTCGLAEIADVFILCW
YGQKLIDSSDYLYLCACQGNWYKYSAKSQ
KMFISMIVRFSTPCSITLGKLYRVSLECFSTIMKTTMSYFTVINSLREEE
>MmedOR4
MDFFDSHYYKGMKILLCLIGRWPYQTLKERVLITIILSILWSYTMFHRMLVYSNYDNYSSKHEVILETIS
PLIVDTITFAKYITTICKMDTIINLLESIKQDWKIYTNKEEKKILEYYANLGKILSLGYVGAVYMVTVLF
MTEPIVEQTFFKLFQNETIPKRFSIPIYWKTPDIEKYYYYLISFQTLNTNFIISITCASDAMFINLLQHV
TGLFSVTGYLLENVPIEENSEENGQKKIKDVAYEHYVRCMRSHKRALEFAENLESIYVWCFGIVITLNMPVMSVTAMQLTTGTSNVIQSMKYGTFAGVQLLHLFFYCF
MSQKLLTSSSVIPECAMNGKWYLCSVKAQRLVTLVIMRSQISCQLTAGKILVLSMETFTSIVKTSGSYFTMLVQMRNV
>MmedOR3

MDIYERPYYKISKNFASFIGQWPYQSRLHSFMCGSVLWTLFIIQVIPQIIAAVVNSDDQELLLESVSPFI
TDGIYIAKYVNTIRKAKMIRRLFEKVREDWKVPKNNDEKLVLESYLKMGRFLSIGYAAFVNMGVIIYIMDPVLSAIVNIISKSNDSMPLKFSVPMRFIMFDEEKYYWLLL
ILSNTCVIFIINVIICCDVIFITVVQHVCGIFAVVGFRLEHSPSDTVSPDLIEGTRFSMNSQDISYKHFVSCIRDHRRALEFSELIESTFAISFGISVGL
NLPLMSITGVQLLTQSESMRATLKYIMFTGGQILHLFFDCYMSQKLTDMSSRIQHSVARANWYENSVKSRKLLILMTLRSQVPCKLTAGKIMELSIENFGMMMKTAGS
YFTVFLSMR
>MmedOR1
MVTVYEYEEMMKPIKMVNRLISIWPLEENDNSILSRLRIFHRISMFILILIQTVAVTADIVHHWGSMKEVTECALIATAFYLCVLRLTVYTIHDKDLQTSVQIMKNDWIK
FSGEDELTLKEKCLPIIKLAKFFIMTVFSTIGLFMVAPILEVKILGMEEKKLPFRGYFFENQTITPAYGGLYLLGVTAGGFGGSMIAGATTLNLILVMHGAAKFMVVRKNI
ESLKSNSENSITFIDCVRGHQDAILFAERVENTINVLVLGQFVISTGLVCFAGFQITEMAEDRGQLMKYTSFLNSAIFELFLFSYSGNELLTESDAISQSCYASNWVGTSFA
KSMQIVMTRSLSPCKITAVKFYDMSLANFSSIFSFSFSYLTVLRTMEAE

>MmedORCO
MMKTKHQGLVADLMPNIRLMQISGHFMFNYYGEGKKLMHKIYCSVHLFLILLQFGFVAINLVKEKEDVDDLTANTITILFFLHTLIKIVYFAARSKLFYRTLAIWNNPN
SHPLFAESNARYHSIALTKVRRLLFCVGAATVATTISWTTLTFFEDPHVERLNKETNETYIEEIPRLLVRSWYPFDARHGVAHIGMLIYQIYWLFICTVDANSIDVLFCSW
LLFACEQLQHLKAIMKPLMELSATLDTVVPNSGELFKAGSADHLRDNDGVPAEPAMNGDN
MLDMDLRGIYSNRQDFTATFRPTAGTQYNGGVGPNQLTKKQEMLVRSAIKYWVERHKHIVRLVTAIGDAYGVALLFHMLITTITLTLLAYQATKVNGVNVYAASTIG
YLLYSLGQVFLFCIFGNRLIEESSSVMEAAYSC
HWYDGSEEAKTFVQIVCQQCQKAMSISGAKFFTVSLDLFASVLGAVVTYFMVLVQLK
Supplementary Data S5. The amino acid sequences of 211 ionotropic receptors (IRs), from 15 Hymenoptera species.
>BdioIR8a
MKFYLWLMLLLTFGANGIVSQAPVTLLFVIEQPDAELMSNLNDVVAEAESQFGANLVKLDVKLVQVDREFVDENYERVCAELYKGITMILDMTWTGWDKLRDLARDFNII
YKRGDTTISPYVQAVDETMMYKNSTDAALIFENEKELNQTLYYLIGNSIIRLVVIDSLTAQTVERINNMRPLPSYYVIYARTQQMEELFRTALEGGLVRRDKVWYLVFTDNN
YADFSYFKQPNQLKVSVSVFTMKHEVCCHLLYQQSPCVCPPDFRIFQHYFRRLVQLVVETLSELQAINQLQEPQTGQCQNKNATESTASQSNGNNSTLSDFDRRLLNKIANN
DTFEYVPKRTLLTYKAAADLKLLHAHGELETIGTWSRETSIQPLPNKTIEAARRYFRVGTTEAIPWTYKKIDPSTKQVERDSEGEIVYEGYCIDLIKKLSELMDFEYDLVIPKE
GSFGQKVNGQWDGLVGDLAKGQTDIAVSALTMTSEREEVIDFVAPYFEQSGILIVMRKPVRKASLFKFMTVLRLEVWLSIVGALTLTGIMIWVLDKYSPYSARNNKHMYPY
PCREFTLKESFWFALTSFTPQGGGEAPKALSSRTLVAAYWLFVVLMLATFTANLAAFLTVERMQSPVQSLEQLARQSRINYTVLENSTIHQYFKNMKQAEEKLYQVWKEITL
NSSSDQVEYRVWDYPIKEQYGHILQAISQVGPVATVEEGFEKVEASENAEFAFIHDSSEIKYQVTLNCNLTEVGEVFAEQPYAIAVQQGSHLQEEISRKILDLQKDRHFEQLSS
KYWNQSRKGSCANADDNEGITLESLGGVFIATLFGLALAMLTLAGEVIYYRRRNARQAQDQADSKRNSGGAGKPNLINVNDNDQMIIQKLTAKLQLKPAPPVAFDASHKS
SPNINTTTKPRVSHISVYPRPFPFKE

>BdioIR21a.1
MRTCGWLLAPLALALCASARQAFLREERARPSSDSLARLLRHLAREHLHDCVLSLVYDGSYERGRPLDFREYFGALPLVFTQESVEASAGAERRQSKCANYVLFVADMGV
ARGVLARKIGVASKIVVVSAATPWRVKDFLKSDVAGRYANLLVVCRSTSRKSGHGVYLLYTNELYADGSGASQPLLLASWLNGSLSRPSVNLFYDKLQKGFMGHRLLVST
VENPPFTIRRSKSSQDEAQWDGLEVRVLQLAGSHLNFSVEFTEPRSQLPASLNAAKADVAQGLSSMAIGGIYQTIDLTEAFDATAPHYEDCAAFISLASTALPKYRAVLGPFQP
AVWIMLIVAYFALIVPLSFNSKYSVLSLLRHPSGLNNMFWFVFSTYTNSFVVKSPLLGYGIAQNSITLLMGIYWIFTIIVTACYTGSIVAFITLPVFPAAVESAEDLAYYRYRLGT
LDHNGWEERFNASEMEEPALRRLFRRLEHVPSLLEGVQNASRAYFWPYAFLASRTALDYLVQTDFAPSHSTKRALMHVSAECFVRYQVVQLFPRESLYTDKMSSFVVRAH
EHGLLERIVGEVDWEVQRVASMSGKKITKGLSQAVVLEDRVLSVEDTQGMFLILGSGVLAALLALTCENLNSRLRRRRALAKRGPEHASAHWNHRAPTPQSWIADHQRDN
PHPELRLRNSRGSV
>BdioIR21a.2
MLLIIAKILNFRPDFYLPDNIQNERWGNLQSDTYTGLFGEAKDGNAEFYLGDIYYNLRHLQILDLSWPYNAECLTFLTLESLTENSWKLLILPFRLYGWIAVILTLLLAGAVFL
LFAYIYENHINLEDKTAPTTIFTRRFVNDKWFLFIEKKPIVEEWKGLYLFADVQNSFLYTYSMLLQVSLPMLPNAWSIRIFIGWWWIYTILITVTYKASMTASLANSVDR
>BdioIR25a
MCAILLWLLVVAGGGGGGYVAAQTRDVANRPVNVFVINDVGNDVANKSLTNSLRALKEQNADNLGKVYVAQVNVTDSKETLNVICETWKSAINAGSANLPDFVLDTTTY
GVGAETVNRFTALLGIPTLSAQFGQEGDLLGWREISEDQKQYLVQVMNPADLMPEVVREQCSHFNISNAAILFDESFVMDHKYKSLLLNVPTRHVIVPTKPAGTALREQISR
LRDLDIVNFFVLGSEATISAALQAASSLDFTDHRYGWFGVNLNEDFTAQCPDCPRAKLMIFKPAPSPSQQQLSELTSKGALPKPLIQSAFYYDLARIGVLGAKAAIEAGEWRT
TTRTYRDCDGVDENSTVPARNFDFRKRLQQVTSNAANGFVPTYASFAWGKNGESQAKFEVNGVAIEMKDSRVTKEDTVENWQAGIDVPLTIKNEELAKNQTAVTSYRVVT
VIKPPFVMYNNNTGKWSGYCIELLDKIRDHVPFEYTIEEVADKEYGNMDEQGNWNGMVKVLKDKKADIGLGALAVMAERENVIDYTVPYYDLVGISILRKKPRIQTSLFK
FLTVLETDVWLCILGAYFFTSLLMWVFDKFSPYSYQNNREKYKNDDEKREFTLKECLWFCMTSLTPQGGGEAPKNLSGRLVAATWWLFGFIIIASYTANLAAFLTVSRLEAP
IESLEDLSKQYKIQYAPVLNSSEYRYFERMAAIEKRFYEIWKDMSLNDSLTDVERSKLAVWDYPVSDKYTKIFQTMQDAGFPKSKEEAIERVRKSTPTEFAFIGDATDIKYLV
MTNCEFTIIGEEFSRKPYAIAVQQGSPLKDQFNNAILLMLNRRELEKLKDTWWNKNPERRSCTKEDDQSDGISIQNIGGVFIVIFVGIGLACVTLIFEYYYYRYRPQARMKHQ
REQQQQQMNQRNNNTPKNPVATIGAMKFNLRPAPTQNLEPINHRF
>BdioIR25a.3
MKNMTQLRVRTIIKPPFVMYDEATNRFEGILPELMHELGIRLSIDFNVSIQEDGRYGHLDENTGEWDGVIGELTSGRADVGLAAFSVMAERMAVVDFSEPVYKPTGISVLML
KTQPITSFFRFLTILEAEVWWCIVGAYLFTSFLVWIFDTWSPYSYQNNKEKYKDDSEKRIFTLKESLWFCLTSLTPQGGGEAPKNLSGRLVAATWWLFGFIIVASYTANLAAFF
TITKYEKTIESFDDLIGQYRYSYTVIRNSTTHTYFRRMNDLEYIFYEKWKDMTLNDSLTPYERAQLAVWEYPLSDKFIKIYSAIEHNNMVNNFSEALAKLNEKDSRFSLITEA
TDVQYMALTDCRFREIGPEFSKKPLAIALPKGSVWTGKFNEVMMELNKEGWMYWANRRWWQENPDRVTCNDYDVNTGITIENIGGVFVVIGIGIITATCTLIYEFFYFTFW
REKMERFYDRNLKKLKSIISLKIRVKSVSGKALNHP
>BdioIR75a.1
MLNIFRIIKILKVVLITFLFLPVDYCDIDVNNAFIAEYFLFRAPSTVLAFTCNQDDSNIQLFKKFSDVKLQSAIWKLENTTELFSFARMLYQKIGIFIDLRCQQPENVKATFNEAT

NYKMFDELYYWLILGSSLDQSLSLMNDDSFGLSTDLVIAVFENNHFYLYDVYNPCKKRGGTLKVSRVGSWDETNGLTMPNEMRKLRRWNLNGMKLKISGIVTNKPSNMS
VAEYLLDNNFRLSDRMTRIAYALWLHIAYKFNFTIEFIESNNTNEANENDPVVIALMKDEIDASGTAEIMTTERVDFLKAVYPTIPYRTSFIFRSTSSDRLSVRDIFLPLSSTVWY
LTLALSILSVNALALLSVFAKEDFFEYYLRSIIINVGAFCQQGTEFSSNTLSGRIAILHILLFYLLLYNYYLASAVSNRLSEPIIFINDSLHELRNKDFEYASEPMLDFDQHMKSPD
WETRSFYTSRWSKIPESQRFLPPEKGMSLVLKGGFAYHTRPEISYPYVERHFDHRKICEVQEVHLIRPMHLSIFVNINSSFIEMFKSG
>BdioIR75a.10
MERFSTRMIFFFTLLFSLLIYCYYSACVVSARLDEPIYKINDSLVEMGKIPLKMASERIAYLEYLFKAPVWEIQQFYANHWLKLPESERLMEPENAMALVQKGGFTYHV
HPDVAYTLIDKFYNNREICELMEVHLAKPQNTMFGVNNNCTYSELVRVGLTKISEVGLRRRQLLKWQYRKPRCRHDILSASSVDIYEFAPHLILLLIGLLLGVFIYFFETKGN
RKGNHFYTRPCFRNKRLILRKKLFVRRTIV
>BdioIR75a.2
MMKKNEWPSFLNSIHTRVGVVIDFQCPMFEDVQILLDKASDYGMFDERFNWLIIATDLRQILKIINDKPFGLSVDFAIAIKTRYEYTLYDVYSMCRNCKSSLNITLIGTWNRK
HGFHFIAELHKPDRWNFHGSKLKLSGLISQKPENTTTVEFLQDYNSKHLEPTAKLAYALWVPLSEMYNFTIIYQERAIWKKGDKNGPVMNALLAGEADATGTPLTMTLERL
RLAYPVYPALPFRSCFFFRSVSSSQINYKVILEPFTEKVWHYIIVFGFFSINVMVILSAFQDIKLGHKYLNAIILNIGAFCQQGVDFKNDHLSGRIALLFIFVFNLLLNNYYMASL
VSSRLRNYHSKLNDSLNQLTKVNLDFAAEKSLYFDFYMKRNDWETKSFFTNVWTKIPDNKKFLDPNSGMSLVAKGGFAYHTLPETAYPHIERFFDDRKICDMSEVHLVRPT
QLSFFISKNSSFGEMMKCGFAKLTEEGVRNREIRRWLARKPICRIDVLTTEKVTFDETAPLILFMFLSMTLSVTLLVVEKIYYDKILNRLISKILRYLRSI
>BdioIR75a.3
MKYGEILRPFASEIWNLMTFTLIICGVTLFVTLSFEGTDDSLMRCSNSLLITIGAICQQGTNMVLNQISSRITFLCIMVYSYLMYNYYSTCVVSARLSEPITTINDSLNELFKTG
LKVASEPLLYFDFFIKENKWETRMFYTKRWLTTPEQERFMHPDRGMQLVREGSHAYHTHPDVGYPYINRFYTNRQICELTEVNLARLTYATIAVTYNSTMVELMKIGFARLS
EYGIRRRQLNWLTAVKPHCQQDILVATSITMNEFAPHLVVLAIGAVIAVTTCAFEKYIYEHYHHPEKFSRLHDLLRRKFHDASICSAELKVTDIGDT
>BdioIR75a.4
MVLLLSLSMIVLLAIVRYDWNYPEIMNVYHTFLLIIGSLCQQGTDLRMKNLSTRIAYLSLLIFGLLIYNYYSAGVVSARLNEPIVRINDSLNELGKLPLKLASEFMVYFDFFIK
RTDWETRTFYTQRWELVPESDRFMLPEKAMQLVQTGQYAYHAHPEVAYPFVDRFYDNHEICELTEVHLARPTWSTFALTFNSTIVEVVRIG
>BdioIR75a.5
MIFSILCYTYYSASVVSTRLNEPIFKINDSLNELGKLELKMSSEPMIYFEFIFNRITEWDKRVFYEDRWMKIPESEQFTYPENAIPLVREGGYAYHTHPEVSYPIIEKTFSFREICE
LMEVHPLVPAYSTLSVNYNSTFIELSKIGLSRMTEVGIRNRQVQRWTSRKPLCRKDVINVSSTDIYEFAPHLILLVMGMIMAGTIFTFEFIFANYWSSRDRYL
>BdioIR75a.6
MGFFMPFTVTILLIAMHQDFHESNVVIFSNAFLVTIGSLCQQCTDLRIARISTRIAFLFVMIFSLLIFNYYSASVVSARLDEPIFKINDSLIQLGKLRMKMASEDMVYLEFFLKK
PDWETKMFYENYWKPTVPDQEWFVEPEVGMRLVKKGGFAYHTHAEIAYILIEKLFDHREVCELMEVHMGGPFYTSFGLHLNSTVVEIGRVGMIRIAETGLTIRQIHRWQFR
KPRCRKDILSAESITIYEFAPHLIFLGIGAILAVAIFALEIYIKKRVHLLVRSRAHAIMEIIASRVKDEIADSFHG
>BdioIR75a.7

MYNYYSAMVVSARLDEPIFKINDSLNEMMKLHLKWSSERMPYIQNFFKAQFWEIKIFQKEVWDKVKDEEKFMDPEAGMRLVQKGGFAYHIHPDVGYGIVSRLFDNREIC
DLTEVHFSRPKMTQFAVTNNSTFTELMRIGLTKISETGLEIQQLQRRQYKKPVCRKDILSATSVDIYEFAPHLILMMIGVLFAFIIYAVEIVNINRRTILKKIDKRIEIEITQSWEML
T
>BdioIR75a.8
MYFEFAVSLLFASTLFVIFRTCFIFRNPQPTSIKMNEIFRPFANSIWVLLCINIVMVIFTFNMALSYDCNESQIMRMSNSCIMVVGALCQQGSDIKMTRVSTRIIFIFVMTTSLLLY
NYYSACVVSARLDEPIYKINDSLVELGKMNLRMASEKIVYIEYFFRLPYWETRIFHRDYWSKIPEPRRYMDPEIGVPLVQEGGFAYHAHPEAVYPLIDKLYDNREICELMEVH
LFRPAYTMFAVTNNCTFTEVMRVGYDVYFALKYHQKYLLFNQDFFSGYRDSQKPDSAIENSKNGNIKNQDAVETF
>BdioIR75a.9
MVQVYFNLPCSCFFHMKEFQYIDSILFFHYPLFLDDISLIKKFSEKGLRATFRKFDNKSNINRLASNLYYRIGILLDTRCCSMKNVAVILDNATNCRMFDELYYWLILGSNMSN
ILNVINDAAFGISTDLVIAISTGNGSYALHDAFNVFKGRGGILNITYYGNWNTSKGLAIKAGQIKSIARGDLRGLPLKAMFFQSPYKPLNMSLPDYYRNTQNGTKDGRSKFG
FWILSHLAEMYNFTIEPVETRKWIANDTIGPLIRAMMTHEIDLTGTPSNINSKRAHLAKFLHQDWPFRTCFIFRNPQPQNIKIGEILRPFADSVWFLMCLTIGMGIIILSLALRHD
LHEVDFISIANSFTMTIGAVCQQATCVDMQLISTRIAFLFIMIFSLVIYDYYSALVVSARLDEPIYKINDSLVEMAKMRLPMASERMVYLENFWKQPYWETQIFYQNYWLQW
PEEKRFMEPELALELVRKGGFSYHVHPDVAYTIIEKTFDNREICELNEVHLGKLVNSMFGVNNNCTYAELVRVGLTKISEVGIRNRQILKWQYRKPRCRQDVLSASSVDMFE
FAPHLVLLAIGFILAFLVYYVLETIGSKWTIESRSDMCNKKIKLILRKKVHKRVHNLFF
>BdioIR93a
MSKYTMFLLLIFLQRFGRIDGHNDFPSLITANATMAVIVEKGFFDDKDDYRDALMDIADVVTGTIKNNMKQGGIDIFVFENTNVDLGRDYTVLLSVATCQTTWNLFKRAQK
EKLVHLAITGPDCPRLLEGDGISLPLIDPGGELAQIFLDLRMSEALAWAKVNFLHDDTFDSDTISRVMKALSVELPNKKLLLSTRAIFSVQYDKSDTVMKQRIHKMLGGFQV
DELGSCFMVFVTVDMVPAMMEVAKSLRMVHPASQWLYVISDGTSHKAANVTMFLDLLVEGENVAFVYNASSLGPECNMGLTCHVKEFARALAISLESSLSTELELYDSVT
EEEFEVVRLSKAERKDEIIKSMNRELTEQRASTTNTCGECTSWKIASAITWGASFVVNESKRHRKANKTDTAKRSIGELINSGTWSPTPGVRMNEELFPHIAHKFRGRSLPVT
TFHNPPWQVVSLTDTGQPEFGGLVFNILDYLSQKLNFTYTVHLPSSQDTLSKLRPDNTTAKAQKAIKFEVSEAAISVARKVPTEVIELVRDSRVFLAAAAMTVNENTKNVNL
TYMIAQQTYSLLSAKPKSLSRALLFMAPFTPETWACLTSSLFIVGPFLYLMIKMSPVPIAPDDKNGLKTTWQCTWYVYGALLQQGGMSLPKADSARLVVGTWWIVVMVVV
ATYSGNLIAFLTFPRMDEPIDTVDDLVARKSEFTWAYPNGSAVENYLVAAAQNTEKYKEILDAAQQEDPTDPKRALAKVKDGQRVLIDWRTSEAFLMRLDLMDTGVCNFH
VGTEDFLHENMGLLVAADSPYLELINSA
>BdioIR75b
MMMQANDLRVYDEMHSWLIFADGDLTKTVKKMDKLNGFGTSTDSVIAVRIVDGYELFDVYNPWKEGGAKINISRFGTWNRSSRLHITLTQGQFRRRCNLHGLVMRANFF
RVKFSDISNSYTRFPIQDVIFIAE
>BdioIR75c
MKIFAIICIIGLEVFADRSDKFVVDYFVYKKVTSVVTLNCNAYKDNLSLVKQLNYVGIKTSAAKVNEDLNIYSLAKTEYHKVGVFVNGQCHKNGNLSSALYEAGYDRLYG
ELHHWLLVISECDDWANLVHDNYFDLSTDLVVALAKNGYEMYDIYNPGKSRNFKLNVTFMGTWTAGDGLKISLTQSKYARRANLHGMVWRVAYLTSKYKPTSVLLENHL

RDFENTGTKDNLAKFGYSVVSHLAEMHNAT

>BdioIR75d

MYENLHYWFILAQNLTQVVQYIDDRAFGVSTDLVIAISLDFNNYELYDVFNFCKDRGGVLNITFLGYWKQDTSFDISLKESKFVRRSNLHGLVLRAAFFQTVYRPPGMPLEE
YYEDHAHAQRDGLSKFGFHLMTHLSELFNFNLKLHQAKQWEIGDRIGPIVRALNSSDADLTGSPLTMNIPRTHWVKFVHQDWPFRFRINMNKVI

>BdioIR75e

MQERCTNNPQDHVAKIHVSYLEMYFILILANIFLVHAHGAHDSDFIVDYFFNKKVINVIGFTCYDLLGDVSLIRSLNKKHITASTYKLNQSKIHLLTRATYWKIGILVDTRCYN
TADITLAFNEATEYRLFDELRSWLILGNNLKNVLNLVNDKAFGISTDFVIATLVHPSSCTFHDVYNIFKERGSALNVTFYGNWNSIVGLNVSLTQSKFRRRANFHRLVIKAMY
YKSLFKAADTPLEEYYEDFEHKTRDSQSKFSYHVLSHLSDIYNFSLHSRESPLWVKGDKVGPIERALTNRDIDITGTPISFTTRRLLVMKYVHQDWPFRY

>BdioIR75u

MRTLYRIWLLIALLHAGVKFCESANNADELTELLVQLKSTGISSFSTVTIFGCSDDNYKLKIAGELSKAFISNNMHDLDSDQFAWKELIASFPAYQRLLILDLDCPHSESFLLEA
KAEMMFVAPNKWLMTTHDCPNTDCVLSELVDLDKKLLPDSEVLIWLSQSQRLISVYKVSVESGRDWQIEERTQYLHNHTLLLEDNSAIISKRRSDLQRAHLKSSLVLTDYD
SLKHLDDYQNREIDTISKCNYPWTKFLTSMLNATISFKLTPTWGYPRANGSWDGMTGMMQRGEIEFGASHSFVTRERLKVVHYLAGLTTPSHSRFVFRRPALSSIANLFALP
FRGSVWLAVLALALLFGFLLYPAMMLEWRRHQYSMTEPRLGDETLVVIGALSQQGFWYEARAASTRVLVLAGLLGFLSLHAAYAANIVALLQSTSSSIKSLKDLLVSPLDFG
VHDTVFNRYYFKSLGNLEPKIRGAIAEKSEWLDLDEGVRRLRGGNFALHAVLGWAYKLVHESFEEDEKCDFEEIDFLNVFEPHMVVAKFSPYKELLRVKALRIRETGMRTR
ETSRLYSRRPRCDAAASSRRFISVGFEECKGAFYALGYGLLVAVGLFVAEMVYRYLPVWICSDRLKQ

>BdioIR1

MYTFNPFTIFAPEPWELVKIYQQENGHPIALFRLAQKRDVRMALKFDKTLTLGGYPIKLVAKHNPPFTTYDDKNKKFIGLGGILFQVMMEKLDVRAEWHAYSGGPR

>BdioIR2

MSYFFIPSMYFQVVFLFLLVKDVYAVIENNLIVDYFVQEQVSSVIGYSCNIFKDDLLLFKKFSSHGLTSALFKINDETNRYSLTLNQYSYNLSLIYRKIGIFFDTRCYSTENISAV
YSEVRYMIFNK

>DmelIR7a

MFHHLWLLMGLRSLAMGALHPPQPEAMTPLVAAALEILAEQVSPSQSTLAVMDLTQDAEHRDERQEQLMTIILRSVGSEMALRTFQKPPAEVPASFVVFLVNSAQAFNTLG
FHFTDIHSTREFNFLILLTHRMSSRAERLQVLRDISRTCVRFHTSNVILLTEKRDGVVLVYAYRLLNMDCDLSVNLELIDIYKNGLFRHGHEARSFNRVLSLSGCPLQVSWYPL
PPFVSFIGNSSDPEERAQIWRLTGIDGELIKLLASIFDFRILLEEPCNKCLSPDIKDDCSGCFDQVIISNSSILIGAMSGSHQHRSHFSFTSSYHQSSLVFIMHMSSQFGAVAQLAVP
FTVIVWLALVVSSLLLVLVLWMRNRLVCGRSDLASHALQVLTTLMGNPLEARSLPRSSRLRILYAGWLLLVLVLRVVYQGKLFDSFRLPYHKPLPTEISELIRSNYTLINQEYL
DYYPRELTVLTRNGSKDRFDYIQGLGKEGKFTTTSLIATMEYYNMMHWSTSRLTHIKEHIFLYQMVIYLRRHSLLKFAFDRKIKQLLSAGIIGYFVREFDACQYRKPFEEDYE
VTPIPLDSFCGLYYISLIWLSAAVVAFILELLSQRIVWLRRIFE

>DmelIR7b

MKYWLYILSCCSLVASTMESSSDWDLAEALAQVVANSEMGRFKTLYIYTHTNSQSTGGHLEELLDQVLMIVPNNLQARRLLLQQSMEYKPYVHAVLALVDGLPSLSAIYAR

IRATQDLSHTLIYMSMPTDAYGEEMQATLRFLWRLSVLNVGVVLRPPGDHILMVSYFPFSALHGCQVISANVVNRYQVGTKRWASQDYFPSKLGNFYGCLLTCATWEDMP
YLVWRPDGSGSFVGIEGALLQFMAENLNFTVGLYWMNKEEVLATFDESGRIFDEIFGHHADFSLGGFHFKPSAGSEIPYSQSTYYFMSHIMLVTNLQSAYSAYEKLSFPFTPL
LWRAIGLVLILACLLLMLLVRWRHHHELPRNPYYELLVLTMGGNLEDRWVPQRFPSRLVLLTWLFATLVLRSGYQSGMYQLLRQDTQRNPPQTISEVLAQHFTIQLAEVNE
ARILASLPELRPEQLVYLEGSELQSFPALAQQSGSSARVAILTPYEYFGYFRKVHPMSRRLHLVRERIYTQQLAFYVRRHSHLVGVLNKQIQHAHTHGFLEHWTRQYVSAVD
EKDESVARIASTSYSTLDGIDGDPSLSESEEDQQVAPVRQNVLSMRELAALFWLILWANLGAVVVFVLELLLPRIKLRKILRKMKSDIKKQISKLVRK
>DmelIR7d
MDIRCVVALLLGLCKVQAVVWPHQHLLEEQLASQISATLQKIFINGLAVYNFGVFISTSYEEMDRDRVILVHQVLNRNLYPPNFPVAVVLASKMNRKITAQVFTQLLFVQNA
EQAIAIAEGVNRNGLCVIVLLTSQPERPIMTKIFTYFMQERYNINVVILVPRLHGVQAFNVRPYTPTSCSSLEPVEIDIKDGDLWDVFPRRLKNLHGCPLSVIVWDIPPYMRIN
WKSSDPMDGLDGLDGLLLRIVARKMNFTLKLIPNEPNGLIGGSSFMNGTFTGAYKMLRERRANITIGCAACTPERSTFLEATSPYSQMSYIIVLQARGGYSIYEVMLFPFEKY
TWLLLSTILGLHWIVGSRWRMPSPILAGWMLWIFVIRASYEASVFNFIQNSPVKPSPRTLDQALSGGFRFITDHASYRMTLKIPSFQGKTLISAGQPVDVFDALLKAPWKTGA
FTSRAFLADHLVRHRKHRNQLVILAEKIVDNMLCMYFPHGSYFAWEINKLLFNMRSFGIFQHHSQILAWDNLPTTTDTDTPGKRIHSSTESVATGFAESMSFVVAALNCLMG
ALCISIVVFGLELLSRRRHWTGLEWLFERV
>DmelIR7e
MNISALLNSYYDLSGEQMNHINEFVARAVLHVVHHYILSVTPSLVLTLCCRSNHTCNFYNKMMSTLFREWGLAPLQIVNVLRGVPWHPVPGRRHFNVIFTDSFAAFEEIRM
EYYSREYNYNEHYFIFLQARDRLLQGEMRLIFDYCWRYRLIHCSIQVQKSNGDILFYSYYPFGEHGCSDMEPQLINRYNGSMLVEPDLFPRKLRNFFGCPLRCALWDVPPFL
TLDEDQEEVLRVNGGYEGRLLLALAEKMNFTIAVRKVHVNMRDEALEMLRRDEVDLTLGGIRQTVARGMVATSSHNYHQTREVFGVLASSYELSSFDILFYPYRLQIWMG
ILGVVALSALIQLIVGRMLRERMGSRFWLNLELVFVGMPLLECPRSHTARLYCVMLMMYTLIIRTIYQGLLYHLIRTHQLNRWPQTIESLVQKNFTVVLTPIVQEVLDEIPSVQ
HMRFRLLEANSELDPLYFLEANHQLRQHVTASALDIFIHFNRLSADKVHQRGEQGSGAHFEIVPEDIISMQLTMYLAKHSFLIDQLNEEIMWMRSVGLLSVWSRWELSESYL
RNEQSFQVLGTMELYAIFLMVLVGLIVGLLVFILELVSMRSIYLRKLFT
>DmelIR52a
MALGWSVIILGFIGQLSAQILNYTQSRDLELLEGSLFRVLSRLNLEEEYNTLLIYGKECVFHSLLRKLEI
SAVTVPSGSTDYDWSFSTAILILSCGYDAENEENSYTLMKLQRTRRLIYLEDNSEPESVCMRYSLKEQHN
IAMVKSDFDQSDTFYSCRLFQTPNYVEGHFFKDQPIYIENFQNMRGATIRTVADSLVPRTILYRDEKSGE
TKMMGYLGHMINTYAQKLNAKLHFIDTSKLGAKKPSVLDIMNWVNEDIVDIGTALASSLQFKNMDSVWYP
YLLTGYCLMVPVPAKMPYNLVYSMIVDPLVLSIIFVMLCLFSVLIIYTQHLSWKNLTLANILLNDKSLRG
LLGQSFPFPPNPSKHLKLIIFVLCFASVMITTMYEAYLQSYFTQPPSEPYIRSFRDIGNSSLKMAISRLE
VNVLTSLNNSHFREISEDHLLIFDDLSEYLVLRDSFNTSFIFPVSVDRWNGYEEQQKLFAEPAFYLATNL
CFNQFMLFSPPLRRYLPHRHLFEDHMMRQHEFGLVTFWKSQSFIEMVRLGLASMEDLSRKRNEEVSLLLD
DISWILKLYLGAMFISSFCFILEILRCGERCKRLWRCRW

>DmelIR7f
MNTTSDSNAGSSLSSGSGYSIYKSYLENSRIDMQGEDANLYVARALRLVIENVLAQLSTTLVVTISTRHLGTAHWFEYMMNILMDSWRMVAVQLLRIRPDLVVNPVPGRKR
VSLLMVDSYQGLLDTNITASNANFDDPDYYFIFLQARDHLIPKELQLILDHCLAHFWLHCNVMIQTAQVEVLVYTYYPYTADACQKAYPIPVNTFDGRKWKASQMFPDKL
SQMHGCPLTVLTWHQPPFVELVWDPKHNRSRGSGFEIQLVEHLARRMNFSLELVNIALLRPNAYRLAEGSSEGPIEKLLQRNVNISMGYFRKTARRNQLLTTPMSYYSANLV
AVLQLERYRIGSLALLVFPFELSVWMLLLLALLIHLGIHLPSARRGNEEDGGGGLQVVALLLGAALARLPRSWRHRFIAAHWLWASIPLRISYQSLLFHLIRLQLYNTPSFSLD
QLLAEGFQGICTANTQRLLLEMPQLARDPDSIQSVDTPFDWDVLNVLTRNRNRKIFAVANQDVTLSFLHSSAHPNAFHVVKQPVNVEYAGMYMPKHSFLYEKMDDDIRRL
DASGFIHAWRRASFASVHRKEQVHMTSRRYINHAKLSGIYMVMAGLYLLAGLLFAGEVLLRQRN
>DmelIR52b
MTWLVILLCFLGYMAAHIADISVQNQSLMDNELINLLLKLRNEEFYDTLLVYGKDCEFHSVIKNVDVAVVLVSDSMNFEWNFSSLTLILSCGPDIDNGGPNSTSIKLQRNRRL
VLLKEDFQPSNICNIYTQKEQYNIALVRENFTKSKSIYTCRYFQDPNVDEVNLSGTKPIFIEQFQNMKGKAIRIVPDLLPPRVMLYQDANDGELKMIGYVANLITNFAQKVNA
TLQLDFLKPSTSITEISRMAKDDELDMGITLEASLNTSNLETSSYPYLLTSYCLMVQVPAKFPYNLVYALIVDPLVLGIIFVLFLLLSVLLIYSQKMSWQDLSVANILLNDKSLR
GLLGQSFPFPLNASKKLRLIFTILCFASIMLTTMYEAYLQSFFTNPPSEPEICSFQDVGSYNRRIAMSALEVNGLIKTNNSHFREIRMDDLEIFDNMPECYELRDAFNLSYNYVV
TGDRWRSYAEQQTLFKEPVFYFARDLCFSRLIFLSVPLRRHLPYRHLFDEHMMQQHEFGFVNYWMSHSFFDMVRLGLTSLKDLSRPLAYTPSLLMDDISWIMKIYLAAIVL
CVFCFLLEIGVDKWKRWMKFRNLQILNTC
>DmelIR52c
MVWLIIILFCLGNSSSQILDVTNNSHLDFDYRLFGLLQRLQVEKSYDTLLVYGEDCAIPSLFERLQVPAVLVSSGSTNFDWNFSSLTLILSCNFQDEREENYRTLMKLQTSRRLI
LLKGHIKPESVCDFYSKKEQHNVAMVKENFYQLEVVYSCRLFQDQNYEKLNLFDGKSIYKDQFRNMHGAPIRTLSDKEPPRTIPYIDSKTGEEKFKGYVGMLISQFVKKVN
ATMQIREDLIKDDEEVSFVDITNFTSNDILDIGICEARTLEMSNYDAISYPYLMSSYCFMAPLPDSLPFSDVYMAIVAPSILIMFLIIFCICSVLIIYIQERSYRSLTIRSVLMNDICL
RGFLAQPFPFPRQYNRKLKLIFMLVCFSSLISTTMYTAYLQAFLWGPPIEPRLTSFDDVKKSRYTMAINIYEREFLEALNVSLEDVEIYDYGKFSKLRSTFNTNYLFPVTALQW
FTINEEQKLFKYKIFYYCDAFCLNQFDILSIPLRRHLPYRDIFEEHMLLQKEFGLTKYWIDQSYRDMIRANLTTFKDFSPLLENDYIEVHNLYWVFTMYFVGMGMGLCFFILEI
LRPLRYWRNCKIKCEYCYAFLKNFAK
>DmelIR52d
MVRIIIILLCLGYTKARILDATNTNHTDLEERLLSLLLRLQQEQFFNTLLIYGEDCAFSSLSRRLQVPTILVSSGSTSFEWNYSSLALILTCEFKAEREENYQTLKKLQMNRRLIL
LNGNIKPDSVCDFYSKKDQYNIAMVNNNFHQVGIIYACRLFQERNYEKVYLSEGNPIYVDQFRNMQGALLKSITFNLIPGSMAYRDPKTGQEKHIGYVANLLNNFVEKVNA
TLDMQVKLHKAGKKTSFYNITKWASEDLVDIGMSYAAYFEMTNFDTISYPYLMTSTCFMVPLPDMMPNSEIYMGIVDPPVLVVLIAIFCIFSVMLNYIKQRSWRSLSLVNVL
LNDICLRGFLAQPFPFPRQSNRKLKLISMLVCFFSVITTTMYTSYLQSFMWGPPIDPKMCSFADLENSRYKLAIRRYDIEMLRPFNVSMDHVVVFDESSQLEYLRDSFDDNY
MYPMSALSWSAFKEQQKLFAFPLFYYSEKLCLKPISFFSFPIRRHLPYRDLFEEHMLQQNEFGLSTYWIDRSFSDMVRLKLATMNDFSPPRLEDYIEVSDLSWVFGMYFTGL
GISCCCFGLELLGLPSWTRRLRLTNWLRVRN
>DmelIR54a

MWTVITGIVLWAPVLVAGSAVDFIFRAAAEHSLSVIMIRIDYCPYNWAKDIFENQTIPVVVLSDSETFINIRMFSRPLHVACLPGHELQKDLALLENFTSSLMDFPSQKKIVYIS
NNFSDPTRMDYIFETCYHRRIWNIVGLLASDEHRYFYRYHLYPSFRTEYRSLESSTIFDKDFPNMHGHPLTVMPDQWLPRSVLYVDRRTGKQILAGSVGRFFHVLSWKLNAT
LQLSKKVTTGRFLNATALKELSESFSVDVPASLTIMERVEQLASTSYPMEVTHVCLMVPVARRIPIKDIYFILSSASNMFLAIVIVSSYGLALNLLRNMTHRDVRLVDFVLNDK
ALRGILGQSFNLPLSRSFSTRLIFLMLGIVGLNVSSIFGAGLDTLMAHPPRQFQARSFAGLRRTKIPLVTTEEDFPTWMKLRVPMLVVNVSEYNHLRNGRNTSNAYFASRLYW
NLFSEQQKRFTRELFIYSTDDCLWSLALLSFQWPQNSLFTEPVSQLILEVNANGLYDFWVGMHYYDMTAAGLSGLEDPSLQLKEREHPTSLRIVDFQWMWQAYGTFMVIAI
LVFLLEVSWHRITSLFVSLVY
>DmelIR56a
MGSRFFIRNLILFGLLASSNMQIPFGELEKKFELDVDFLLGVTELVGHIQGLYSITVYADCIDIHPSIQQRIMDKFMVPVNTIGSNLSRPNYHKLDNSRIRIVLFTGLNDTILVNL
NKTDVPYSDNFYMLAYASAIKNKCIELDFIEEVFTLLWKMSIQNAILLIRGEFMMEMWSYLYMGKIHKIKLTKPNSYLESLRKYNYRFSLEVINDPPAIFWYNSSEQADVTG
GGNLSVSGPLGLIIINFLRHLNVTIDIVPIPGKQTSQYELFQQPDNLRAENGVNMVGSALLKYSPMVTQSRMCLLVSNRRMIPFSRFLDRLVSPGVHKLTFVSSIGIFVIKYFSH
RPRSFVDAIFCTIRFFFAIPLPSIILNRLPVVDRFIEVFIIIFVQILLSSNISITTSALTTGFWEPPIINVETMRASGLHILTEDPTILQAFKENILPSSLADLVILVDEDTYFHHVTTLNN
SYVYVVQAHNWQIFRLYQQQMTNEPFEIASEELCSKWRILGIPLNPKSPLRFMFKDYFYRILESGLREQWVHSGFKKFCEFNNLKKLPVDSVDSWQPLSIEFYSNVIRAYIIG
LVIATLAFVAELLHNGYRRKNVKKT
>DmelIR56b
MLLDTDLASGVIRSPYSFDIPHAFIFNETQFVVPKFCGPYMEIVKHFAEVYHYQLFLDSLESLPKKSVVEQDIISGKYNLSLHGVIIRPEETSDFFNATQHSYPLELMTNCVMV
PLAPELPKWMYMVWPLGKYIWTCLFLGTFYVALLLRYVHWREPGNATRSYTRNVLHAMALLMFSANMNMSVKLKHASIRVIIFYTLLYIFGFILTNYHLSHMTAFDMKPV
FLRPIDTWSDLIHSRLRIVIHDSLLEELRWLPVYQALLASPSRSYAYVVTQDAWLFFNRQQKVLIQPYFHLSKVCFGGLFNALPMASNASFADSLNKFILNVWQAGLWNYW
EELAFRYAEQAGYAKVFLDTYPVEPLNLEFFTTAWIVLSAGIPISSLAFCLELFIHRRKQRRPQYERFECYDY
>DmelIR56d
MDNRAAELILRERNIFPTNGSDNITLLNNMFVLEMFYRITQLYHFKNFIFYISERLDLNNKDSQEFFHNFWTYFPMAPNLIITREHHLGIPMMQFISTPSLVMVFTTGKDDPIM
ELASHNQQGIHWLKTIFVLFPSLQSRDFETNPESLAQFTAEIKDVYDWVWRKQFINTFLITIKDNVFILDPYPTPSIVNKTGVWQAEEFFHKYAKNMKGYLVRTPILYDMPRV
FKSDRPTNRYEKNFIHGTSGNLFLGFLEFVNATLMDTSANVTADYLNMTNLLDLVSQGVYETLIHSFTEITTKFVVSYSYPIGINDCCIMVPYRNQSPADQYMHEALQENVW
VLISLFTLYITVAIYLCSPLRPRDLSAAFLQSICTLTYSVPTFIIRTPTLRMRYLYILLAIWGIVTSNLYISRMTSYFTTAPPVRQINTVQDVVEANLRIKMLAIEYERMAKSPLQYP
ESYLNQVDLVDKHMLDLHRDPFNTSFGYTVSSDRWRFLNLQQLHLRKPIFRLTEICEGPFYHVFPLHKDSHMRSVMTEYIMIAQQAGLMNHWERETFWEAVHLHRIHVHL
FDDEPMALSLDFFSSLLRTWTLGLILAGLAFAAEMKWHEHVTFKRRPVIRITRKPRSFLRRFMKL
>DmelIR60a
MWCNNPGLIIIIFLGQILNLCQGIVNLSNETANTVIFMLPEKDLGPDVWKAGVGCLDSFAQIFFFRNPKERFTRAYNLMLVHAFHLSSPADQIQEGFSKLINEAVTNPGPPDRE
ELFQMRVASDYNITNGTEDKGELILADNYVIVVDSVDRLKELMKKKIVEMRSWNPGARFLVLFHNATCRNRPLGVASNIFKDLMEMFYVHRVALLYANSTMNYNLLVNDY
YSNVNCRILNVQSVGQCHDGKLYPNNAVVKASMQDYVSGFSPRNCTFFACSSISAPFVEADCILGLEMRILGFMKNRLKFDVNQTCSLESRGEMDGPANWTGLLGKVQNN

ECDFVFGGYYPDNEVADHFWGSDTYLQDAHTWYIKMADRRPAWQALVGIFEAYTWIGFILILIISWLFWFTLVMILPEPKYYQQLSLTAINALAVTISIAVQERPICETTRLFF
MALTLYGLNVVATYTSKMIATFQDPGYLHQLDELTEVVAAGIPFGGHEESRDWFENDDDMWIFNGYNISPEFIPQSKNLEAVKWGQRCILSNRMYTMQSPLADVIYAFPNN
VFSSPVQMIMKAGFPFLFEMNSIIRLMRDVGIFQKIDADFRYNNTYLNRINKMRPQFPETAIVLTTEHLKGPFFILVVGSCWAALTFIGELIIHRWRTQLVSTSEQQDRRSDKRR
RRRRRRKPEKDNRWQRQVQVAPVVRFTPVKRRKVFQGQTSQK
>DmelIR60b
MRRSLYLIIAIGLVDVHCVSLRYILNALENELQYRAILLVESASEIESCWEQKYIQGAVPILNFNANQSLYLKDALNTNILALVCLNENVESTMQALYENLEDMRDTPTILFVL
SDSKVQDVFLECLRRKMLNVLAFKGLDRGFVYSFRAFPTFRVIERNVMDILQYFEQQLEDLGGHTLTTLPDNIIPRTVVYKSPDGSRQLAGYLYPFLRNYVSTINATLKVCW
HLVPEDGMIQLGEVVRLSEIHDVDFPLGMHGIEHGSTSQNVPLEVSSWFLMLPMEPSLSRAQFFIMLGFEKVTPVLLLLTILLSTAHRIEMGLRPSWRCYVLGDRVLQGTLG
QAFFLPRRLSVKLMLVYSLILLNGFTFSNYSITSLETWLVHPPSGHPIHSWEQMRTLNLKVLIVPSELDSMTKALGKQFTESNSDLFELSKSGNFQDKRLAMDQSYAYPVTCT
LWPLLEHAQIRLPKPEFRRSREMVLIPLLIMAMPLPKNSMFHKSLNRYRALTHQSGLYEFWFKRSFNELVALRKIHYKVNGDHQIYRDFEWQDFSYVWLGFVGGTIASILVL
LAEIGYHRWQLNQN
>DmelIR60d
MRLAIYVAFLSSIGNRSGFLSSLLMSLGKELHYKTILLVGGSSTCWSLEPFETGVPILNLRGENNAYPQDTFNSQMLALACLQTESEDAVKLLYRSLKDMRDTPTLLFASSEE
HIHDTLFLGCFRENMLNVLALTASSKEFIYSYQAFPTFRVIKRKLVEIHRYFEPQLKDLGGHIVSALPGNIMPRTMCYRNAEGERQLAGYLNTFIRNYVESINGTLRISWGLVP
EDDMRHLTISRLSKIQHVDFPLGIIPLYNKTDKQHVYMEISSWFLMLPMETSVPRAHLFVKLGLERLLPIIVVVGAVLGNAHRIEVGLGPSWRCYYLADKVLRGALAQPIVLP
RRLSPKLMLIYSLLLLSGFFLSNYYMASLTTWLVHPPASDRILEWDQLRYLHLKVLTIPEEFKYMSLILGTDFMTAYGSIFQLTNSTDFQRRRISMDPSYAYPVTTSLWPFLELS
QVRLRRPLFRRSYDMVLQPFQVMSLPLPRNSIFHKSLLRYAALTRETGLYYYWFRRSYYELVALGKISYKEEEGNPYCDLKWNDFRIVWLAFLGGTIISCLALLLEVAHYRW
HLGNSSL
>DmelIR60e
MVIKMISFLLVSVLLCLVGASDSESMQVQVLQDLNLALQTELNVFIDFECCATSEILHKLDSPRILLSSNSREARDLRIRGNFTESTLIIVSVMDSDLNPLVASLLPRLLDELHEL
HIVFLSNEEPGFPKQDLYTYCFKEGFVNVILMSGKGLYSYLPYPSIQPISLSNVSEYFDRARIIRNFQGFPVRILRSTLAPRDFEYSNEQGGLVRAGYLFTAVKELTYRYNATIES
VPIPDLPEYDVYLAVAEMLHTKKIDIVCYFKDFSLEVAYTAPLSIIREYFMAPHARPISSYLYYSKPFGWTLWAVVISTVLYGTVMLHLAARGARVEIGKCLLYSLSHILYNCH
QKIRVAGWRDVAIHGILTIGGFILTNVYLATLSSILTSGLYDEEYNTLEDLARAPYPSLHDEYYRSQMKAKTFLPERLRRNSLSLNATLLKAYRDGLNQSYIYILYEDRLELILM
QQYLLKTPRFNMIRQAVGFTLESYCVSNSLPYLAMTSEFMRRLQEHGISIKMKADTFRELIHQGIYTLMRDDEPPAKAFDLDYYFFAFVLWTVGLISSLLVFFAELVSGHL
>DmelIR64a
MHWWLLVFLPLSCQGLPEHELLELELDYGLAEPQRTSLLQSSLILQFSQDYKHIPRITYFTCQKPHLQTPNQIPNAAEHRDAFAAKNFQLIKSLYESELFVRIVLLDVLAQSPT
SGRPNRPGNGPTGGFSQTPSQAQSNSEWLEGVLRMEALRQIAVVDLACGAVSRRFLELASAKMLYSEKFHWLLIEDFAWHGRTQTAEGSGKRDDGEMEEEEPPGQQIQAT
DDEDLPSIESFLGGMNLYMNTELTLAKRMSEAAHYTLFDVWNPGLNYGGHVNLTEIGSFTPTEGIQLHTWFRTTSTVRRRMDMQHARVRCMVVVTNKNMTGTLMYYLT
HTMSGHIDTMNRFNFNLLMAVRDMFNWTFVLSRTTSWGYVKNGRFDGMIGALIRNETDIGGAPIFYWLERHKWIDVAGRSWSSRPCFIFRHPRSTQKDRIVFLQPFTNDV

WILIVGCGVLTVFILWFLTTIEWKLVPHDGSALIKPKGGAPPRHHYQQQQQQEQVEAPVRPITAVSVVVSKEKVEEKQEEYEDSTPIDAGTLWQRCYQKLNKYIKDRKAKQ
KKAPERVGLFLESVLFFVGIICQQGLGFSTSFVSGRCIVITSLLFSFCIYQFYSASIVGTLLMEKPKTIKTLSDLVHSSLKVGMEDILYNRDYFLHTKDPVSMELYAKKITSVPTT
KENEADEDEPVDPNPVSTDPAKSYRDIVHSHETGAHAKDNAASNWLDPETGLLRHLGFAFHVDVAAAYKIIAETFSEQDICDLTEVSMFPPQKTVSIMQKNSPMRKVISYGL
RRVTETGILTYHFNVWHSRKPPCVKKIETSDLHVDMDTVSSALLILLFSYAITLMILGTEILYSKWHNRIQLKWVGAT
>DmelIR67a
MLPILVPVLLLFNETSWINPILTSIYKDRHHETVLLLQHSQHGNASGLERFPWPVFSFNEQMDFYVRGKYNSEMLVLIWQTGNSDWDLDLWQALDRSLLNMRKVRVLLLR
KWEKIPTADVAATAEHLLFLHVAVIGQGNRIYRLQPYAPQSWLQVDPIESPIFIKIRNYFGRYIVTLPDQFPPRSIVYRNPKTDEIQMTGYVYKFLLEFIRIYNFTFRWQRPIVQG
ERMNLILLRNMTLNGTINLAISLCGFETPSELGVFSDVYDMEEWYIMVPRAQEISIADVYVVMVSGNFLIVLIIFYFIFTILDTCFGPLLLKERVDWSNLMLNERMISGIMGQS
FNMSARNTISSKVTNATLFLLGLVLSTLYAAHLKTLLTKRPTSQQISNFKQLRDSPVTVFFEEAERFYLKHAWDRPIRYIKDQLNFRETIEYNALRMGLNRSNAFSALTSEWMI
VAKRQELFKQPIFTVQPELRVIQTSVLLSLVMQSNSIYEDHINDLIHRVQSAGIVEYWKHQTLREMITMGMISQKDPFPYVAFREFKVGDLFWIWLLWVSFLFMSFVIFLCELL
VDCFISKTLIRNKRPH
>DmelIR67b
MELLYLNTLQSLSLLEGNRLVQTVQELNNIYQTELNVFLEFGNGADILESAQGTFVPTLWIKNPQNQKVMKGNFTSCTLTILYLEDEHLDRGLYYLANWLWEYHHLEVLIFF
NGGSYDKLIQIFSRCFNEGFVNVLVMLPGSDELYTFMPYQDLKILNLKSIKEFYSLSRKKMDLNGYNITSGLVIAGAPRWFSFRDRQNRLILTGYMLRMIVDFTNHFNGSVRL
MNVLTVNDGLELLANRTIDFFPFLIRPLKSFSMSNILYLENCGLIVPTSRPLPNWVYLLRPYAFDTWIAWLIMLIYCSLALRILSKGQISISAAFLKVLRLVMYLSGSRDMGTRP
TTRRLFLFVILTTSGFILTNLYVAQLSSNSAAGLYEKQINTWEDLDKSDSIWPLIDVDIKTMEKLIPDRTKLLKKIVPTLEADVDTYRRNLNTSCIHSGFFDRIDFALYQQKFLRF
PIFRKFPHLLYQQPLQISAAFGRPYLQLFNWFVRKIFESGIYLKMKDDAYRHGIQSGLLNLAFRDRHLEVKSNDVEYYYLIAGLWFGGLTLATVCFLLELLIGYAKIKVTISCK
MNIM
>DmelIR67c
MFCWLIFLNIILLSDRSESWSAREVIHQFNHDQQLQLNIYLDCNDVELQIGQEVSNLFVNSTADKMKILGRFSSHSLIIACFKDSTRNRTLNGVKELLWGLQYLPILFVVDSN
MDFYFQQALRHGFIHVLALNFMNGSLYTYKPYPKVEVHQIKDMQKFYKLTKLRNLQGQAVRTTVETMTPRCFRYRNRHGQLVYAGYMYRMVKEFISTYNGTEEHVFGN
VDTVPYKEGLAALKNGEIDMMPRIIHALEWYYFYRSHILYNIKTYIMVPWAEPLPKSLYFIQPFRGTVWITIMVSFVYASIVIWWIRYRQQGNSSLTQSFMDVLQLLFQLPLS
KIWHFNMGTHQVVSFIVLFVFGFMLTNLYTAQLSSYLTTGLFKSQINTFDDLFREKRTLLVESFDAEVLHNMTKEKIIQKEFESIILITSIEEVFKHRKSLNTSYAYEAYEDRIAF
ELSQQRYLRVPIFKILKEVYDQRPVFVALRHGLPYVELFNNYLRRIFESGIWIKLQEDSFLEGIASGEISFRKSKSREIKIFDKDFYFFAYILLGMGWCVSTIALFLELWSFKYSV
TNVLHEG
>DmelIR68b
MKFLVGLLLQWYLPGIYALAEIACRIAVEQNVQVTYLYRCASCPASFDADYSALELDLYRCVGSRLPVITRNMEAHELEPFRRTDSLSIFQIPAAEKGDSLVRRILDMLNPHQ
RRKHMHKYLFVWPNAGRHQLLRLFRGSWAKKLLYGLAITGRENGTFDFDPFAWGGLQVIQRLDGEVPYARKVKDLRGYPLRFSMFTDPLMAMPRSPVETAGYQAVDGV
AARVVGEMLNASVTYVFPEDNESYGRCLPNGNYTGVVSDIVGGHTHFAPNSRFVLDCIWPAVEVLYPYTRRNLHLVVPASAIQPEYLIFVRVFRRTVWYLLLVTLLVVVLVF

WVMQRLQRRIPRRGVIQFQATWYEILEMFGKTHVGEPAGRLSSFSSMRTFLMGWILFSYVLSTIYFAKLESGFVRPSYEEQVDRVDDLVHLDVHIYAVTTMYDAVRSALTEH
QYGLLENRSRQLPLGIATSYYQPVVRRRDRRAAFIMRDFHARDFLAITYDSQAERPAYHIAREYLRSMICTYILPRGSPFLHRLESLYSGFLEHGFFEHWRQMDLITRVGASP
DAEEFLEDLGDQTDTDSGSNELAIRNKKVVLTLDILQGAFYLWSVGIGISCLGFAVEHAHWFWRRQTLRNAVEARTS
>DmelIR75a
MQLVQLANFVLDNLVQSRIGFIVLFHCWQSDESLKFAQQFMKPIHPILVYHQFVQMRGVLNWSHLELSYMGHTQPTLAIYVDIKCDQTQDLLEEASREQIYNQHYHWLLV
GNQSKLEFYDLFGLFNISIDADVSYVKEQIQDNNDSVAYAVHDVYNNGKIIGGQLNVTGSHEMSCDPFVCRRTRHLSSLQKRSKYGNREQLTDVVLRVATVVTQRPLTLSDD
ELIRFLSQENDTHIDSLARFGFHLTLILRDLLHCKMKFIFSDSWSKSDVVGGSVGAVVDQTADLTATPSLATEGRLKYLSAIIETGFFRSVCIFRTPHNAGLRGDVFLQPFSPLV
WYLFGGVLSLIGVLLWITFYMECKRMQKRWRLDYLPSLLSTFLISFGAACIQSSSLIPRSAGGRLIYFALFLISFIMYNYYTSVVVSSLLSSPVKSKIKTMRQLAESSLTVGLEP
LPFTKSYLNYSRLPEIHLFIKRKIESQTQNPELWLPAEQGVLRVRDNPGYVYVFETSSGYAYVERYFTAQEICDLNEVLFRPEQLFYTHLHRNSTYKELFRLRFLRILETGVYR
KQRSYWVHMKLHCVAQNFVITVGMEYVAPLLLMLICADILVVVILLVELAWKRFFTRHLTFHP
>DmelIR75b
MLQLHNLILHNLIHMAKLSHVLILHCSLSHLALLAQSKNIFTQFQPLHSDIQLNDDFLNHNILKLGVFLDINCDKSGTVLDMASAKRFFSHRYHWLIYDRSMNFSVLESHFK
EAQIFVDADVTYVTHDPFSKNFLLYDVYNKGRQLGGELNITADREIFCNKTNCRVERYLSELYTRSALQHRKSFTGLTMRATAVVTALPLNVSIKEIFDFMNSKYRIQLDTYA
RLGYQARQPLRDMLDCKFKYIFRDRWSDGNATGGMIGDLILDKADLAIAPFIYSFDRALFLQPITKFSVFREICMFRNPRSVSAGLSATEFLQPFSGGVWLTFALLLLLAGCL
LWVTFILERRKQWKPSLLTSCLLSFGAGCIQGAWLTPRSMGGRMAFFALMVTSYLMYNYYTSIVVSKLLGQPIKSNIRTLQQLADSNLDVGIEPTVYTRIYVETSEEPDVRD
LYRKKVLGSKRSPDKIWIPTEAGVLSVRDQEGFVYITGVATGYEFVRKHFLAHQICELNEIPLRDASHTHTVLAKRSPYAELIKLSELRMLETGVHFKHERSWMETKLHCYQ
HNHTVAVGLEYAAPLFIILLGAIILCMGILGLEVIWHRHCTLH
>DmelIR75c
MTSWPLYRLIVFNLLEINLSNLMVFHCWSIKEAFPLVEMLNQNGIFSQYIDVQNPDNLANVHKEYLDSDLVRLGVFLDLGCDKAELVTNQSSRARLYNQNLHWLLYDEAG
NFTKLTQLFEGANLSLNADVTYVSREDEERFILHDVYNKGSHLGGKLNITVDQTLQCNRSHCQVKEYLSELHLRPRLQHRMDLSSVTFRLAALVSVLPINSSEEELLEFLNS
DRDSHMDSISRIGNRLIMHTQEILGFKLHYIWCGTWSVQDAFGGAIGMLTNESAELCTTPFVPSWNRLHYLHPMTEQAQFRAVCMFRTPHNAGIKAAVFLEPFMPSVWFAF
AGLLIFAGVLLWMIFHLERHWMQRCLDFIPSLLSSCLISFGAACIQGSYLMPKSAGGRLAFIAVMLTSFLMYNYYTSIVVSTLLGSPVRSNIRTIQQLADSSLDVGFDTVPFTK
TYLVSSPRPDIRSLYKQKVESKRDPNSVWLSPEEGVIRVRDQPGFVYTSEASFMYHFVEKHYLPREISDLNEIILRPESAVYGMVHLNSTYRQLLTQLQVRMLETGITSKQSRF
FSKTKLHTFSNSFVIQVGMEYAAPLFISLLVAYFLALLILILEICWARYAKKKFSTIIPQNQ
>DmelIR75d
MKVQVAHWLPLIFFLLVSGTPRVAGSWRSEYSRQDPDPKTRWGNQLPDMLVAYYRHHGVHSLMLVVCHTDIADFRLWKLWQHFNLNNFYVQVSTESSLRDLQHVDALDE
HKDAPPPKSFHANNSTHWETSFLLPALPYKMGILLLEFSSECALNLLRWSAASEHNYFTTNRFWLLLTEDPGDIDLLEDPEIFIPPDSELRVLHYENVGNFSCSLIDLYKVAAW
KPLKRTLVGHNIRNSRHVIHALQHFGSAITYRQDLEGIVFNSAIVIAFPDLFTNIEDLSLRHIDTISKVNHRLMLELANRLNMSYNTYQTVNYGWRQPNGSFDGLMGRFQRY
ELDLAQLAIFMRLDRIALVDFVAETYRVRAGIMFRQPPLSAVANIFAMPFENDVWVSILMLLIITTVVLVLELFFSPHNHDMSYMDTLNFVWGAMCQQGFYVEVRNRSARII

VFTTFVAALFLFTSFSANIVALLQSPSDAIQSLSDLGQSPLEIGVQDTQYNKIYFTESTDPVTKNLYHKKIASKGENIYMRPLLGMEKMRTGLFAYQVELQAGYQIVSDTFSEP
EKCGLMELEPFQLPMLAIPTRKNFPYKELIRRQLRWQREVSLVNREERKWIPQKPKCEGGVGGFVSIGITECRYALGIFGCGAAVSFVLFLFEFIFRHFKQVYRIIKGYREVQR

>DmelIR76b

MATGIELLVAAALCVACPPLNDSPPTNLIQMGENGTLSPVTELPMDVDASEAGFDADAPVETLETINRKKPKLREMLDWIGGKHLRIATLEDFPLSYTEVLENGTRVGHGVS
FQIIDFLKKKFNFTYEVVVPQDNIIGSPSDFDRSLIEMVNSSTVDLAAAFIPSLSDQRSFVYYSTTTLDEGEWIMVMQRPRESASGSGLLAPFEFWVWILILVSLLAVGPIIYALII
LRNRLTGDGQQTPYSLGHCAWFVYGALMKQGSTLSPIADSTRLLFATWWIFITILTSFYTANLTAFLTLSKFTLPYNTVNDILTKNKHFVSMRGGGVEYAIRTTNESLSMLNR
MIQNNYAVFSDETNDTYNLQNYVEKNGYVFVRDRPAINIMLYRDYLYRKTVSFSDEKVHCPFAMAKEPFLKKKRTFAYPIGSNLSQLFDPELLHLVESGIVKHLSKRNLPSA
EICPQDLGGTERQLRNGDLMMTYYIMLAGFATALAVFSTELMFRYVNSRQEANKWARHGIGRTPNGQSVAPSRWLRGWRRLNSGHGQLLGASTHGQNVTPPPPYQSIFNG
GSHGDPLNRWRRPLANGNALGNGVLLGGDSEGGVRRLINGRDYMVFRNPNGQSQLVPVRSPSAALFQYSYTE

>DmelIR85a

MSIQWLKHILLLAILVNLAGTRENHIPLDLKKSSIVMVKMSQILCKARIKVLFVYFENQTSHEHTGQILKEVTKCDISNQNTPLEAVKDDGILMYMVMITTNISQPLELSLIRK
KSAAKHRSHVFLLVRDADTVSDAWMRASFRQFWKIWLLNIVILYWRDGRLNAYRYNPFMDNYLIPVDNKPNEVPTLEQLFPKTIPNMQRKPLRMCIYKDDVRAIFWRQGT
ILGTDGLLAAYVAERLNATMMITRPHSYNNHNLSSDICFLEVAKEYVDVAMNIRFLVPDTFRKQAESTVSHTRDDLCVIVPKAKTAPTFWNIFRSFGSLVWALILVSVLVANV
FCYILKSEVGRVPMQLFAGALTMPMTQIPPNHSIRLFLIFWLYFGLLICSAFKGNLTSMMVFQPYLPDINQLGALARSHYHIIIRPRHVKHIQHFLTLGHKHESRIREQMLEVSD
TQMYEMMRNNDIRFAYLEKYHIARFQVNSRVHMHLGRPLFHLMNSCLVPFHAVYIVPYGSPYLGFLDSLIRSSHEFGFERYWDRIMNSAFIKSGVKVVNRRRGSGNDEPVV
LKLQHFHAVFALWLVGIGMACIVLAWEHLTHNYNLAVTKRRD

>DmelIR87a

MSTPEQRFWLAALLFLLSQHSEVRGFGINLMKVQTEDKGQEACILALLRKYFDSGDGLSGSVLCINRNYQLPNIEEQLLRGVNNYENYPWSLLITNSREGPSPAKFLMNEKP
QCYFLIVDNLEDEDLDEVFEHWKGMVNWNPLAQFVVYLASLEETDEEMNDLMVELLLTFINKKIFNVNVIGQSEENQFYYGKTVFPYHPDNNCGNRVISVELLDACDYPS
EETDSEDENDEDEGDGAQEEDDGPQEEGDGEQEEEDGPQEQEDGDQAKGDEGQENDDGGLENKVENEFRIGASDDDELENDLSSNSSEPEAIIEEFFRAKFEDKFPRDLSG
CPLTASFRPWEPYIFRNSEEQPVDDYYYGLQGDEDDYNDTSPNYGESDDESYADPGEDGDGAIPDTETQSGGKLKLSGIEYEMVQTIAERLHVSIEMQGENSNLYHLFQQLI
DGEIEMIVGGIDEDPSISQFVSSSIPYHQDELTWCVARAKRRHGFFNFVATFNADAGFLIGIFVVTCSLVVWLAQRVSGFQLRNLNGYFPTCLRVLGILLNQAIPAQDFPITLRQ
LFALSFLMGFFFSNTYQSFLISTLTTPRSSYQIHTLQEIYSNKMTVMGTSEHVRHLNKDGEIFKYIREKFQMCYNLVDCLNDAAQNEHIAVAVSRQHSFYNPRIQRDRLYCFDR
RESLYVYLVTMLLPKKYHLLHQINPVIQHIIESGHMQKWARDLDMRRMIHEEITRVREDPFKALTFDQFRGAIAFSGGLLLVASCVFAFELCYVKYVYRTEKRERKTKKITKK
VHNIKIQHD

>DmelIR92a

MLLQPLVMHLSQLLRIIVGQYFAEFPSILIVYNNSASTTPLQLEYLSALELVLRELSKPIRLQWINVAFLKDLNDLEDQVMGALNSSVTEGFITILSQTHHFIHARYYATRNANV
RLKDKRYLFLCEDESPAELLCMDILQFYPHHLMVRPGTETAPTGPTGPHPDPRRGGGASVSTKNKDDGEGGAGNKTTSPYRDINFELWTQKFVGAVGNLDALLLDAFLPNE
TFANRVELYPNKLLNLQRRSLLVGSITYVPYTITNYVPAGQGDVDPIHPQWPNRSLTFDGAEANVMKTFCQVHNCHLRVEAYGADNWGGIYDNESSDGMLGDIYEQRVEM

AIGCIYNWYDGITETSHTIARSSVTILGPAPAPLPSWRTNIMPFNNRAWLVLISTLVICGTFLYFMKYVSYRLRYSGTQVKFHHSRKLEKSMLDIFALFIQQPSAPLSFDRFAPRF
FLATILCATITLENIYSGQLKSMLTFPFYSAPVDTIEKWAQSGWKWSAPSIIWVHTVQSSDLETEQILARNFEVHDYSYLSNVSFMPNYGFGIERLSSGSLSVGDYVSTEALEN
RIVLHDDLYFDYTRAVSIRGWILMPELNKHIRTCQETGLYFHWELEFIDKYMDKKKQEVLMDLANGHKVKGAPQALDVRNIAGALFVLAFGVAFAGCALVAELLIHRMDL
SK
>DmelIR93a
MNPGEMRPSACLLLLAGLQLSILVPTEANDFSSFLSANASLAVVVDHEYMTVHGENILAHFEKILSDVIRENLRNGGINVKYFSWNAVRLKKDFLAAITVTDCENTWNFYK
NTQETSILLIAITDSDCPRLPLNRALMVPIVENGDEFPQLILDAKVQQILNWKTAVVFVDQTILEENALLVKSIVHESITNHITPISLILYEINDSLRGQQKRVALRQALSQFAPKK
HEEMRQQFLVISAFHEDIIEIAETLNMFHVGNQWMIFVLDMVARDFDAGTVTINLDEGANIAFALNETDPNCQDSLNCTISEISLALVNAISKITVEEESIYGEISDEEWEAIRF
TKQEKQAEILEYMKEFLKTNAKCSSCARWRVETAITWGKSQENRKFRSTPQRDAKNRNFEFINIGYWTPVLGFVCQELAFPHIEHHFRNITMDILTVHNPPWQILTKNSNGV
IVEHKGIVMEIVKELSRALNFSYYLHEASAWKEEDSLSTSAGGNESDELVGSMTFRIPYRVVEMVQGNQFFIAAVAATVEDPDQKPFNYTQPISVQKYSFITRKPDEVSRIYLF
TAPFTVETWFCLMGIILLTAPTLYAINRLAPLKEMRIVGLSTVKSCFWYIFGALLQQGGMYLPTADSGRLVVGFWWIVVIVLVTTYCGNLVAFLTFPKFQPGVDYLNQLEDHK
DIVQYGLRNGTFFERYVQSTTREDFKHYLERAKIYGSAQEEDIEAVKRGERINIDWRINLQLIVQRHFEREKECHFALGRESFVDEQIAMIVPAQSAYLHLVNRHIKSMFRMG
FIERWHQMNLPSAGKCNGKSAQRQVTNHKVNMDDMQGCFLVLLLGFTLALLIVCGEFWYRRFRASRKRRQFTN
>TjapGluR3
MREKSRLFLACVLVLAFHPHLSPHHVGKDATTGVLALPPVIKIGAIFTHDQRNSSTELAFKYAVHKINKDRIVLPNTTLMYDIQYVPKDDSFHASKKACQQVKYGVQAIFGP
ADPVLGQHIHSICDALDIPHLEARLDLDQETREFSINLYPAQSLLNAAFRDVMSYLNWTRVAIVYEDDYGLIKLRDLVRQPKKSPEQEIYLRQADVDTYRAVLSEIKAKEIRN
LIVDTKPENMHHFLRMILEMQMNDYNYHYFFTTFDIETFDLEDFKYNFVNITAFRLVDADDVGVRGILRDMEKFQSAGNLLNRSHVIQAEPALMYDSVQAFAVGLRTLEHS
HSLRPMNISCELEHPWDGGLSLINYINSVEMKGLSGPIEFKEGRRIQFKLDLLKLKQHSLVKVGEWRPALGINVTDRTAFFEPGLTNVTLVVITILEQPYVMLRQRGNHSGVG
ISGNDQYEGFCIDLLKEIARMVGFTYKIELVPDGKYGVYNYETGEWNGIVRQLMDKKADLAVGSMTINYARESVIDFTKPFMNLGISILFKVPTRHQARLFSFMNPLAIEIWL
YVLAAYILVSVTMFVVARFSPYEWNNPHPCHSQGPVEIVENQFSLANSFWFTIGTLMQQGSDLNPKATSTRIVSGVWWFFTLIIIASYTANLAAFLTVERMITPIENAEDLAG
QTDISYGTLDSGSTMTFFRDSMIETYKKMWRFMENRKPSVFVPTYEEGIKRVLQGNYAFLMESTMLDYIVQRDCNLTQIGGLLDSKGYGIATPMGSPWRDKISLAILELQEK
GEIQILYDKWWKSPSDTCMRNDKGKESKANALGVDNIGGIFVVLLCGLTFATLIAIFEFCYNSKRNAPPESVSL
>TjapGluR2
MKHSIERFIIFMVITVCTALPNIIQIGGLFHDKNIEQENAFLNAVEQVNANENVLPKSKLKALVEKILPQDSFRASKRVCHLLDIGITAIFGPQNAYTASHVQSICDTMEIPHLET
RWDYKIKREGCLVNLHPHPVTLSKAYVDLVKALNWKSFTIIYENNEGLVRLQELLKAYGPSHTSINIKQLGEDSLNGHGYRPMLKQIRNSAESHIILDCSVDKIYTVLKQAQ
EIGMMTDYHSYFITSLDLHTIDLSEFKYGGTNITGFKMIKPESLMITSISKNKKMKDDHSLNMKTETALIYDAVHLFAMALHVLDTSQQIDVKPLSCDSTDTWDHGYSLINY
MKNVEMTGLTGTIKFDNQGFRSDFTLEIMELNTKNGLEEIGSWNSSFGINFTRSFREVYTQMIDSLQNKSFIVTTILSAPYCMWKESSKRLAGNAQFEGYSIDLIHEISKILGF
NYTIQLVPDGRYGSLNRETREWDGMIKELLDQKADLAIADLTITYDREQAVDFTMPFMNLGISILYRKPVKQPPNLFSFLSPLSLDVWIYMATAYLGVSVLLFILARFSPYEW
EHPHDYNHQSKMTENEYTLLNSLWFTIGSLMQQGSDIAPRALSTRMVAGMWWFFTLIMISSYTANLAAFLTVERMDSPIESAEDLAKQTKIKYGALKGGSTAAFFRDSNFS

TYQRMWHFMETSKAPNEVFTKSNVEGVTRVIKDKGNYAFLMESTSIEYVIERNCELTQIGGLLDSKGYGIAMPPNSPYRTAISGTILKLQEEGKLHILKTKWWKEKHGGGS
CRDESSKSSSTASELGLANVGGVFVVLMGGMGIACVIAVCEFLWKSRKIAVDEKRQQMCQKPMCIGFTDIQ
>TjapNmdar2A
MTNRSGAKSKSLASLLLVSLLLSQQHWAVSAAGPGLLDGSVQRTPSSIKIGKSSRSHAAGSGATGQAGGSSAPVRGNSSIKIGKGNSLLNTTRMTRPVLTTSMTSTNIPLAYV
TSSTTPADMGLSSSGAEYTSSNGTDIKKLKVGLAVPYKSFGSREYTRAVIRAVSAMQKTSRHKNLSLFQHYDIHVKVAMQELTPSPMNILNSLCKDILSNNVSAILYLLNYEQ
YGRSTASAQYFLQLAGYLGIPVIAWNADNSGLERRSSQSSLHLQLAPSIEHQAAAMMSILERYKWHQFSVVTSQIAGHADFVQAVRERISEMQERFKFTLLQAVVFTGKQD
LRVLKKSESRVMLLYSTKDEATAIFRDAQELNITGENYVWIVTQSVIENRQPGYTFPIGTIGVHFDTSSTSIVNNIATAIKAYAYGVEDFMNDPSNAQYSLNTQLSCDGAEGES
RWSIGDYFFKYLKNVSVDSDTPGKPPLEFTQDGAPKSAELKIMNLRPSVSMHLAWEEIGTWKSWERRGLDIKDIVWPGNQHSPPQGVPEKFFVTISFLEEPPYINLAPPDPVS
GKCLMERGVHCRVSRDPESVENVAGTTEPSNGPGSSTTTAQPQQNGSTYQCCSGFCIDLLQKFSEDLGFTYELVRVQDGKWGTIENGKWNGLIADLVNRKTEMVLTSLKIN
SEREAAVDFTVPFMETGTAIVVAKRTGIISPTAFLEPFDMPSWMLVGFVAIHSATLMIFLFEWLSPTSGFRGLDSRSHHHRHHHHHHHHHRHNHHHHHHHGHHGAGQTNLSS
QRHRFSLCRVYWLVWAVLFQAAVHVDSPRGFTSRFMTNIWAMFAVVFLAIYTANLAAFMITREEFWDFSGVDDHRLARPMSHKPMIKFGTVPWTHTDSTLSKYFREMYAY
MKTYNKNNVAEGVEAVVNGDLDAFIYDGTVLDYLVSQDEDCRLLTVGSWYSMTGYGLAFPRNSKFLKMFNQRLLEYRDNGDLERLRRYWMTGTCRPDKEVQKSSDPLA
LEQFLSAFLMLMVGILIAAILLFFEYIYFKYVRRHLAKDSRTAKCCALLSVREMFMNPEMEVPN
>TjapIR93a
MTEPLFPHVSQGFRGINLPVTSYHNPPFQIISQTSSGSLRYSGLIFDILNHLSLKLNFTYTMQLLPGFSPVVTAAAATAAVAKETTAASFMTDNVTSSKLDVPTSHAAQVPPQLT
ELVRKRKVFLGAMAITVSDNLAGVNFTATVSTQTYGLMQAKPEILSRALLFVAPYTNEAWACLISALILTGPFLYLMVKLSPLSAEDRGSLGLATTWQCSWYVYGALLQQG
GMNLPKADSARLVVGTWWLAVMVVVATYSGNLIAFLTFPRTDAPIDSVDDLLGRGNEFSWSFANGSVVENYLSLAAANGDVKYKELFDGATRQETSKAANILESVKRDRL
VYIDWKMSLEHLATSDLKTTGVCNLHVGTEDFLPENLAMMIASDSPYLSLINDAIKRMHESGLIKKWTEDRLPPKNKCHENMKAQEATNHKVNMGDMQGIFFVLAIGFTV
AVAFIGMELAWHRKKEAAERLLIKPFVS
>TjapGluR1
MTINYARESVIDFTKPFMNLGIGILFKVPSSQPTRLFSFMNPLAVEIWLYVLAAYMLVSFTLFVMARFSPYEWNNPHPCLGETEVVENQFTISNSFWFITGTFLRQGSGLNPKA
TSTRIVGGIWWFFTLIIISSYTANLAAFLTVERMITPIENAADLAEQTDIPYGTLEGGSTMTFFRDSKIAIYQKMWRFMESKQPSVFVSDYEKGVKRVLEGNYAFLMESTMLD
YAVQRDCNLTQIGALLDSKGYGIATPKGSPWRDKISLAILELQEKGVIQILYDKWWKNTGDVCNRDDKSKESKANALGIENIGGVFVVLLCGLALAILVAILEFCWNSKKNA
QSDRRAAGHAVPMTAMKKSLSYDQMTVDLSNSNEGIS
>TjapIR25a
MPSSSSSSSSSSSLIWTLVCVGLVHLIITPGIRAQRGGGGDDSANIPVRPINLLVINDFDNDLANKSVSNSLKALKDKSPDKLGQVYVALVNISDGEETINSVCSVWKSALRSNP
DEIPDFVLDVTTFGAGAETVNRFTALLGIPTVSAQFGQEGDLLGWREITEEQKQYLIQVMNPADLMPQVIRQQCLEFNISNAAILFDDNFVMDHKYKSLLLNVPTRHVIVPT
REAGEPLEQQISRLRDLDIVNFFVLGDEATIAAALAAASRLNFTGHKYGWFGISLVQEFSLRCQDCSSMSLMLFKPKQSQNQQQLNELMSKGLLQIPLIMSAFYYDLAKLSV
LAMKSALDAGEWRRPRYVDCDEFNENVTVAMRNVDLRRRLRQVSGGSGFSPTYAGFHWGNKNGENHAKFDVDVKLWIIKDSRVVNEDQVAYWEAGIDNKLKVEKPTM

AADHTAVTSYRVVTVIKPPFVMRDNVTGNWSGYCIDLLNQIRDLVQFEYEIREVEDKEYGNMDEAGNWNGMVRELKDKKADIALGALAVMAERENVVDYTVPYYDLVG
VSIMRRKPKAATSLFQFLTVLETDVWLCILGAYFFTSLLMWIFDKFSPYSYQNNREKYKDDEEKREFTLKECLWFCMTSLTPQGGGEAPKNLSGRLVAATWWLFGFIIIASYT
ANLAAFLTVSRLDAPVESLEDLSKQYKIQYAPIINSSEYRYFERMAAIENRFYEIWKDMSLNDSLSDVERAKLAVWDFPVSDKYTKMFQTMKDTGFPKNLDEALERVRAED
KTEFAFIGDATDIKYLVMTNCEFMQVGDEFSRKPYAIAVQQGSPLKDQFNNAILMMLNKRKLEALKDQWWNKNPLKQQCDKADDQSDGISIQNIGGVFIVIFVGIGLACITL
IFEYYYYRYRPQARQRHRDRSSKTKFAPGGISGKPMKFNLRPAPTQSFEQSADTDSGGHRSRY
>TjapIR21a
MMANYLNLTMEFSSPRSSGLSPLESTKQDLLLGLTSSAVGGIYQTADLYERFDTSMPLLDDCASFISLASTALPKYRAMLGPFQLTVWLMLCASYLALVVPLSFNSKYSKRQ
LLRQPGAVNAIFWYIFSTYTNSFSVENPLLDYGIAKNSTTLLLAIYWLFTIIVTACYTGSIVAFITLPVYPEAIETAAELQAYRYRIGTLDHDGWNEWFGPDNGQEPLLDRLFRK
MEYVPSLMEGVRNASRAYFWPYAFLGSRAALDYLVQTDFALARQSKRSLMHISQECFVRYNVVQLFPRNSLYTSYADGFVLRAQQSGLLDRMRNDVDWQVQRAAINDN
KQVSSTSSASSFSLFT
>CvesIR1
MFNNVFLYFLIVPSVIAFSKSIILGGLFPEEDLSKQAFELSINAVNTIRAETDESDDPFFKPEVITIGDDVFDISQGVCALAGQGVAAIFGPEDTISSIHVQSMCDTMDLPNLVAR
WDSNLLRMKAINFYPHSETLPLIYLYLVTEFKWEKFTILYDSTEGLIRLNRLLQRWDTKGYPVTLRFLGEGPDYRGILKQVRNSGEENIIIDCHYPALEEILKQSQQVGILSER
HKVIITSLDLQTLDLDPYSHSGVNITGVRLLDPEDPDSVSIFNDYLADMGLEDLSKMRTEWALIFDAVQLFSRAFEQLEEAVEVEVKELACDGVDNWEHGASLGNFIRATEM
KGLTGLIKFHTNGFRSDFKLNVVRLTTDGLRTIGNWNSTGKIEWIPEPPPALPNGLIDLANQTFTVLISLLDPFNMLTESAATKSGNDRYEGFAIDIIHELSEILHFNYTLVEQLD
KATGGYDEKSKKWTGMLGKIIDGEADLAITDLTITSEREKVVDFTMPFMHLGISILYKKPKKAPPSLFSFLSPFSGAVWIALFGALFFTSLIAFIIGRICSPEWNNPYPCVENPTE
LENQWTFSNCLWFTIGSIMQQGSEVAPIAISTRMLATAWWFFCMLMTASYTANLAAFLTVETIVRPIKSAEDLAALNGDIPYGAKKNGATYKFFQGSNYSTYATMYKYMSE
HPEVLTESNDAGRDRALKEDYAFLMESSSIEYISQRFCDLTQIGGELDNKGYGIAMRKNFPYRNLLNTAVLQLQESGILSELKKKWWNEKRGGGGCSDESQGSAAQELTLD
NVGGVFLVLVVGVSITLLISAADMVSDVWGYARKENLPFKEELKKELAFFIKCRGTVKPSRSARRSSSQPDSSRGDTPPYGFVPTVITSPHSEEP
>CvesIR25a.2
MDNFRWCTIFGVLIFWSWFVFTEQVVQQPTGLRPVNLLVINDQENDLANQSIDIALADIRAKDPTVLGNVIVVQLNESEPKEALDQVCGVWNSTVKSGTSGVPDLVLDTTR
SYIGTKTVNIFTAFLGIPKIAARYGQRGDVKHWEDLTANQTNYLVQIMPPVNLIPKAFRQLDCEKNVSNVAIIVDDAYATNRIYKGLLQNISSQHIVVQAETDNPDIEQQLDRI
RDLDVVNYFVLGNENTLTNYLDVADDKNLTGCRYGIGSNQAHLRSLTAQNLLPPVWLTVAFYYDVVRIGVGAMKSAIEKKSWPTSLEPRFITCEEYNGNNTPSHNFDLLGE
LRNATTNKLEPTFTRFHWGENNGEHHAEFNIRVNMVVINDGNSIFSDDLGLWNVDIRSPFTSKSHSALVHYAPVPTYQIVTIERPPFIEFNKETKKWEGICIDLLEEMQKYTKF
NYEIYTSPDGEYGSLDDDGKWNGMINELITHKADVALGALSVTAAREYVIDFTVPFYEPVGYSVITKRRLDSTSLFVFRKSMSWKVWASSFVAFVSTSVLIYIFDRWSPYSFR
NDPHNRSVTQHTRIFTLRNSFWYTLSCLSPSGGGPPPKNFSGQILACSWWGFGFITIAAYSANLAASTTVGRLQPTIKTWDQVREQFKIQYAPIKDSNAYKYFFGMKAIEESF
YDIWKGISLNDSLTDRERAQYAVWDYPVDDEFSRIVSHMNEYGYPKSKKDALNRMFGLPPYRNNSNFALIAESTELRYWALTNCSFFLFEGEFTKKPYAIAVQQWSPIKDLF
DHALIELEKNYTMERLKKKWWDNNPKRVFNCPERINRSEGLHIGNIGGIFIVLAVGNVLGFLLVILQYWWYCIRSRTRLRKLLENGFSLDKVKPKKFKVKPKLRKITREDKN
SPAGIVVEEL

>CvesIR75u
MKLFVYKIFFCLFIASNSHEIDQVAETFIVDMIKAIRAPLTVTGFFCFSSNLKLSKKLSQNNIINDIKNLISHEYDFEENQELKLSHQNLYILDLDCPDATELLLKAEDSEMFVAP
TRWLILQDIKNNNLSQIDLKKQFDDLGIFPDSELYLAQRLKNDSINILSIYRPSIYVNLIFEYRGNWDALNGVNFMDSIPASQRRRNLHLTPLKTCLVLVNPDTLNHLTDYKDK
RIDAVTKANYIWMMHLVNRINATVTYTWRNTWGYQDQNGTWSGMIGLLDRNEIDFGGTATFLIKQRIGVIEYLHLYTPVGSKFVFRKPPLSYVSNLFTLPFGRTVWYAIAV
MTCVICGFLYITLKWEWKKTLESEENTRHPDDLQKDPSLSDDLLILSSAIFQQGLSYEPRTISSRIITLMLLLAALSLYASYTANIVALLQSTSNSINTLKDLIQSGLKFGIYDIVY
NRYYFGALDDPIRREFRERFVTNKSSVWLTMEDGIERVRKGLFAFHVDTAAGYQLMQETYDEDEKCGLQEIDYMGVLDPMLVIKRRSPYREIFKVGSLWLREAGLQQRDT
PRLFTKKPVCVGQTSFISVGTTECYAAFYTIIYGVAIAFGVLFIEIIYHRCFESKTDDSSESENDVENVDQLQEESRQTSGESLEVIE
>CvesIR76b
MIGLELMLAGIYALTSNNTLNYTLDHRYRWEVSLDDVVSWNYNKTKAAELYDGGLPEELLVTTWDEGPYSSYNNVNGKIIGGGYAFQIFDIIAKKLNFKYRIIPPEDLTVGN
NETGMISMLNQSKVDMAVAFIPIIPEYMNFVEFSPILDSLEVSVLLERPLQSAIGSGLLAPFSKTVWICIFVSLALVGPIIYFVTSFRTWLWDRTNKDRYSFIDCVWFAYGAILKQ
GSAITPEADSNRLLFASWWIFITIITAFYTANLTAFLTLSEFTLEFTSVKDIYNLRKSWTAQRHYIIDITVNRNDPGELEPLKKSLNKTRGMFYNITKEDNKAKKVAGYVTNDRL
FIDEVQFVEDFIYRDYLNRTRLNMIEKEKCYYIKMPNSVYEQNRGFAYPLNSRLRKIIDHQLLHLVETGIIKHIESVNRPMVTYCPLDLGSTERRLENRDFKLTYEVIATGFLL
ALICFIVEVSQHYFKCRVCLCCADSCTCCKTRMKVQVSPAKRPQLPLPPLPITKGPIKTEFSAFPQPQLPIYDSIDNVISSKTRFINGRDYFVIKGITGEKRLVPVRTPSALLFQY
TN
>CvesIR21a
MRIFFLIIMLSAVVNSNDQLDNNRRKVFNKKLSDSSARTESLGRLLKHIFREYFNGCIVIMMYDDKIYTEYPGLLNMLNEELSSVSFIRRNFNNSQSESSDKLQFKCYNYLLF
LNNIYTLQSTLSRQNHKKVVVVTQSTPWTVKEFLKSFPSRSYNNLLIISHTMSERTEPGSYLLYTHKLYADGSGASYPTLLTSWIRDHSTQKAIDLFPEKLTGGFKGHRLLIAT
AHQSPFAIRTGIDWDGIDIRIVKLLSKTLNFTADFRDPVTINSPIYAATTDVITGKASVAAGGIYKTTNITSVLDTSASHMEDCAAFISSSSLALPKYRAVMGPFQPSVWVLICIV
YFIAIIPLTMNTDYSLSSLITRPGRLIHMFWFVFSTFTNSFAVKDPLTVNGFAKNSTALLIGFYWAFTIIITSCYTGSIMAFITVPMFPESIDTIDQLLDEGYDVATLDHDGWEKWF
NWSTIDDPVAVKLLKSLEYVPTIEEGVRNASSSFFWSYTFLGSKMALDYLVQSDYTPSWSTRRSTMHITKECFTNFGVTLVLPKDSIYTQTFDTVIMRARQSGLTQKIIRDVE
WDLQRTAEGTRLPISEDYKRRKVIIEERPLALDDTEGMFLVLGAGILIASLVLGIECCLKRLKKKHTVADAGTTFTSLATTPRMSFLTVPNQPWDIESSNCSPRPRRFIRGSI
>CvesIR68a
MWSLSENYLFPRRFKNVSRIEEMEHQSGIVDLEGRELQVAAFFNPPLCYLFTAINRTVNGINGQFFTAPENEIDGIETILFVIIAERLNFTWLIRKPNDHYRYGRPNGTNWNGG
MIGQLFRNEIDVAFSGIWLKHDHYRFVNLTKPWHQLFINYLVPRPKHRFSFWALTRPLTPGVWLAIFGMIFLHSLNVYTKAWIIPHAERRYRSYITTLIELIARLVGTWAPKRV
QGFKIQTMAWHMAGILIVTAYSCSLATDLTFPDFEKRIDRVDQFILENLTWGRESPVPKFDDYFNLNVSYFIYIA
>CvesIR64a.2
MNLRKGIFLLPLLIRLIKCDQFLENFTRDYFDSIHVYQLIVFGCWNLQEAYDFAKNVMRQEAKVSYNEIRDNINLESTLRLNAQTLGVILDFDCPSSDLILNQFSRQLAFNESY
RWVLLTESLEFPTPSLKDLPLSVASEMTVAIRQNNTYKLYDVYNPSYRHGGVINVTLMGYWTPEHKLVNNFKHQFKYHRRQNFNLLPLNVSVALVNEPKPDLETYLTTPIDI
HLDTMSRFTYQIVLQLQEYYNFSINIKRDKSWGYKVNGSFNGIIGDMEKGIVDISSTPFQLKRERLEVIDYTVHVMTIRTVFFFRHPKKDDMQNGFLKPFDSSLWYIVFGVA

GVYWFILFVTTKVESHVKNIKVPDNSLMSLPATETNLITMAALFQQGANDSPRLISGRIAFLSLFVWTLLLFQFYSATVVGFLLAPPAFWITTLKNLTDSSLSCGMEDMDYYR
DFYATTYLETAIEMHEKKIKPTKKLPRGSYYPAVEGLLKVRDEPFAFHIEDTTAYRIIEDLFTEDQICELQEIPIRAPREVYVGVPRHSPFKEIITYGCRKSVEHGLANRLRQHW
RYKKPPCPESHSSKPMPVMMKEFAPALMFLLFGFGLSVFILFAECIYHAKFDRDSNESDNQSITPVQSAGTAEQHIEKKFDHDETVSHFSA
>CvesIR8a
MTHTGWDNVRDIVINNSIIYLRSDGSIIPFTQATDDLLKKKNATDFALIFENPSDVDPALFYLLGASRLRLVIIDNFNNASMHKLRDMKPQPSHYMIYSSTKGMEKLFQTALA
GNLVKRSGVWNLVFTDMDYKNFRYIKGPEVLEHSVGIFSMNVSICCSLIEKLTCDCPPDFKIFDSYIQLLINQLVVTLVMINSEPGAKLKSIKGTCSPNHPEQEDEIIQKFKNAI
MTVTDKTIALEYMPKSSIVVYRAVIDIEILNNGTLTPLAEWTKDSLTPFPGQVIVASQPHFRIGTTASVPWVTIHTDHLGRPLEDANNRVIAEGYCIDFIEELSKKMKFSYDLIV
PKDGKFGEKSEDGSWNGLVGDLANGKTDIAIASLTMTSEREEVIDFVAPYFEQSGILIVMRKPVKETSLFKFMTVLKSEVWLSIVGALVATALMIWILDKYSPYSASNNKQLY
PYPCREFTLKESFWFALTSFTPQGGGEAPKALSSRVLVAAYWLFVVLMLATFTANLAAFLTVERMQVPVQSLEQLARQSKINYTVVNNSNAYQYFSNMKNAEDTLYRVWK
QITLNSTSDQSEFRVWDYPIKEQYGHIFQTIKTVGPVANASEGFEKVLESTKAEFAFIHDSAEIQYQVTKDCNLSEIGEVFAEQPYAVAVQQGSHLQEEISRAILDLQKDRFFER
LSAKYWNASLKDRCPNMNENEGITLESLGGVFIATLFGLALAMITLGIEVLYHRRKNNLDGDKAKEANGRHVKSPVSKLMKPKLHHFKPTPTVAFIGRNRGLKPRVSHISV
YPKTFPYKD
>CvesIR64a.1
MKLSLNEMKSRLSTYWLFIIFLTLSFSSSKGHEILAKFTRDYFRNLYINQIVVFGCWDKYDSLKFSKSIIMDPEIKLIYESIDDNIDLDKLLHVDYWKLGIVLDLDCQRSKIIFD
QFTEQHLRHNESYFWLMPTTGEKIPDYFYNLPLTIANEMTLAIRRDENLYNGNNTDNNNKIIYELYDVYNPSYRHGGKLNITYMGHWSLNDRNEGILKILLTQYKYKRRGD
LQGLVLNASIVIDFPAVPDYNTYIHNPINPHLDTMHRYNYALTLQMRDYYNFTMNLQRGKTWGYLVNGSFNGIIGDMLKGLVDFGATPFQYKPERLDAIEYTVQTWVARPC
FIFRHPSTNDLSNPFLKPFENQVWYYIALFGAVNWIFLYTSVKLEHWLVMKKPQMTLDTYPASEILMIVTSAMAQQGLSDGPRFYAGRIVFIFLFMWGFLLYQFYSASIVGSL
LAKKPRWINTLQDLADSNLEVGIEDIAYNYDFFATTTDPVALQLYRDKVAVNKKRKREPYYSIEDGLKLMQKGGFAFHVDVATAYKIIEETFNTGEICDLVEIQLFPPKHTATA
TARFSPFKKMVTYGMRQIVEHGMARRLRNVWMHRKPECPESHKSDPVPVMIPEFSPALFLLAFGFFVALCLVLGEKIIMRHQKIRDMNDDDNDDSVSDQKTGSTNSASSQ
KQEELNSDKIDI
>CvesIR2
MTLSGKMALLASLTFALVMYNAYAGFITSILSVQATGIKTLEDLLQNNFKVGYSDLDDEFMRNTNDTNLRKLYINAFNTRESRIDTNQGLQKAVKGSYGFFASATLARRTLR
TSLIQERCSLKEIEVGQTFTVVALPMEKFSPYEKIINLNILRMLERGVIDRIGDRMLPDMPKCRDPTTFHSARIADVYSAFIILAIGIVIAVCIGLVERIWSRRLKFKNKLRQIIDIY
LRKKRTSEVHTNKNNPKEFLNDYINNINCNLHFKTYKRRLRHSSSRVKKFNPKVNYDC
>CvesIR25a.1
MSNWYWWNFILTFIVADCSVVFAQQNQQPTNPRPLNLFVINDEENDIGNNSVKNALVTIKDKEPTVLGNVIVVQINGSDPKAALDKICAAWDPAVRDGGPGVPDLVLDVTR
GGYGAETVCSFTAAVGVPTFSAQFGQKNDLRPWLNLDFDQQRYLIQIMPPADLIPEAIRQLCSDMNITNAAIIFDTGFVMDHKYKSLLLNIPTRHVIVRAKTTDKEVNDQLQ
RLRDLDIVNFFLLGNEDTLIKYLDIAEMKNFTGRKFGWYALTMTEQPDLKCNNKNISVLLFEPKLTSSNQQHLSFLTNQGIIQKPYLASAFYYDLVRVGVNAMKSAIKENAW
PNVPSHITCDQYNRNNTPSRDGFNLLKWLMDTTKSGFEPTFAGFAWDKRNGEHHASFDMSINMKVIDNGNVISTTSMGSWSANVDTPLNITAYDAVGSHTAITSFRIVTVQI

APFIYINETTHEWQGYCIDLLKEIQGIMNFEYELYESPDGNFGAMNEKGEWNGMIKELIDKRADIALGTLSVMAERENVVDFTVPYYDLVGISILIRKKKEETSLFNFLTVLN
RTVWLCILGAYFFTSFLMWIFDRWSPYSYQNNREKYKDDEEKRVFHLKECLWFCMTSLTPQGGGEAPKNLSGRLVAATWWLFGFIIIASYTANLAAYLTVARLEQPIESLDD
LSKQYKVQYSPIKPSEAYTYFERMAAIENKFYEIWKDMSLNDSLSEIERAKLAVWDYPVSDKYTKMFQQMHEAGFPKTKEEAIDRVRKKLEAHNNTEYAFIGDATEIRYLE
MTTCDLISIGDEFSRKPYAIAVQQGSPLKDQLNNAILKLLNQRKLESFKSKWWSRNPNRRNCEKESSQSDGISIENIGGVFIVIFAGILLACCTLAFEYWYYRYRPRVNAKKQR
KANAQDGNKIPSQRVKPTRFNLKPARKAFEDNGTEFRARF
>CvesIR93a
MNISGLSIHLSQDTGINLKQDYTILLSVTTCKVAWELFKRARAEKLVHLAITDLDCPRLPINEAITVPLVDPGEELPQIFFDMRLEQSLEWNRVNFIHDSNQPITKVIQAFSTDF
PKKLGLASQSLSGFTRGRSEVATRRSIRDLLTKFSSRKALDQQFLVIVGHKFVSLIIEVARSLGLLHPRSQWLFVIPDMADHNKGNVSYLLDYLEEGENLAFLYNSTKKNGTH
CSARAFCHARELVGALTVALEKVLSQELKLYEGVTEEEYEASGINKLIRSRNIINFMRNELYNESRSNGQRSSCSSCLTWNLDSAITWGSRLMSKTKKPSFKLQKTGSWTSDP
GFEALGFIFPHTKFGFLGKELEIATYHNPPWQFEILETDNHFNVKSRWDGLMINILNELAKNLNFTIKYLVIKVPAEAMLAKSDSKNSAMSAADKVPAALTDLVKTGKVMM
AACSFATSMYVNEKNINFTRVITSQSYGILAPRPKAMTRTLLFTSPFSNEAWACLASSIVLVGPALYFVHALSPRKADEQTRNPESTEMLGLGSPSRCIWYIYGALLQQGGMH
LPSTDGARLIVGTWWLVVMIIVATYSGTLVAFLTFPRMDPAVNTVDDLLARREEFTWSIPAGSLLENFLEISDYQQLLPEFDRHASFHETASYNENVEKVKARGHVMIDWTT
ALRISQRNHQINFGACFFSIGTNVLELVEPIALAVPKGSPYLGIIDQQLQRMQESGLINKWLDNWLPNPGDECSDDKMENQGTSNHQVDFYDMQGIFFVLFIGYLTGSVALLS
EFYKQHRKSNKERKLIRPFLD
>BterIR25a
MRLVGFTIAVCGFLQFCGESAVYAQRNVVGNRGSTKTRAVNLYLINDEANKVAKSSIIAALETIKEKYPNYLGEVWSVQVNESDVNDTLDRICKPWDSAVKEGGTRVPDLVI
DTTTAGLGAKISNSFTAALGIPTLSAQYGQEGDLLYWRNLNTDQESYLIQVMPPTDLIPEAIRQLCIQLNITNAAILYDHNFVMDHKYKSLLLNVPTRHVINEASQQVMEMR
TQLPRLRDLDIVNYFILGDENTINIALEAAEALNFTGKKYGWFLLTPQLNVWPRCECRNMNILFMKPEFNKKSPIESSLSKPVISSAFYYDLIQLGVRAMKSALDDGEWPIEP
RHITCDKYDKTNTPERKVNFFNRLKETYKNMTPTYAGIKWGSKNGEHRANFEMSIHLVDIKDGIVSNTIDSGSWNASISAPLQITNNDVMNTTAVKSYRVVTIIHPPFVMYN
EENGTYYGFCIDLLDEIKDTVGFQYEIRETEDRRYGSLNPNGSWNGMMRELIDKRADIALGSVWVTAERERVVDFTVPYYDLVGLSIMMLKTKTTSSLFKFLTVLENEVWF
CILAAYLFTSVLLWIFDRWSPYSYQNNREKYKDDDEKREFNLRECFWFCMTSLTPQGGGEAPKNLSGRLVAATWWLFGFIIIASYTANLAAFLTVSRLEIPIETLEDLSKQYKI
QYAPVINSSAYIYFKRMAAIEWKFYDIWKEMSLNDSLSDVERANLAVWDYPVSDKYTKMLQAMEEAGFPASTEEALRRVRRLDSNNEFAYIEDSTTIKYLTMTNCDLIQVG
EDFSRKPYAIAVQQGSPLKDQFNNAILILLNKRKLEKLKDTWWKKNPDRKDCDAENSQSDGISIQNIGGVFVVIFLGIIFACFTLAFEYWYYRHRTKITKINLNSTTKGKVTQ
VKPLRFNLQPAPTHGFQNSQLRPRF
>BterIR93a
MISVLLLLWCVNYGDSYNNFPSLITTNATMAVIIDKSFFDNNGDHRNVMGVVHDLIINTVKKEMHIGGIVVRIFRDADVNLWQGYTILLSVASCCITWRLHEVARKEELIHL
AITDPDCPRIPETDGMSMPVVVPGEELSQIFLDLRMMNILPWNVINILHDDTFDRDTISRVMTAISDKLPNKQVNLISRSIFTLKHETTRSERKSSVKKTLNDFHVEQLGHCFL
VIATVDMIADVMGVARSLKMVHPGSQWLYVITDSASKNMTNMTAFVDLLAEGGNVAFMYNATNLSNYCEIKLICYVEELIQALAKALEYSLTSEIDLFKSMEEEKFEMIRL
TKRERRAELLKNIRIHLSQNAFASEGFCGRCLLWRFSSSITWGNFFSRGRNMAHLLDIGTWSPGFGVNLTDVIFPHIAHGFRGTNLPIATYHNPPWQIISVSKTGQKLYEGLVF

DAINYLGSKLNFSYTAITPEVTRNSNSWNTSRYAKLGEKIKEMTMSATRKVPKEVIDLVREREVLLGACAITVNENKKDAINFTVPIFVQTYSFLTSRPKQLSRALLFASPFTK
ETWACLAVSIIVMGPILYLVHKYSPYSIKTSGLKSSFQCVWYVYGALLQQGGMYLPHCDSARILIGVWWLIVMVVVATYSGSLVAFLTFPRMDASILTVDDLLARKDGITWS
FPNGSFLEMYMQETDEPKYHTLLSRAESHNDTEEEKLVERVKDGKHALIDWRSSLRFLMRKDLLLTGVCHFSLSMDEFLDEPIAMIIPHDSPYLPVINAELHRMLESGMMN
KWITERMPIKDKCWEVPGSNQAVNKRKVNVTDMQGIFFVLFMGIILAFFFLFCECYCHRRKISKERKLIHPFVS
>BterIR21a
MILVTLFLQIILVSGKSVFYKEHQCENSGNNLKSVVEEIVEEIINQRNCIVFVSDSVYRNLVDVKNIKGSSTVLKYEIALRDNEQFLQPRRRVQRILIDGKAVNCSAYIILIANGF
LAAEFLQYTERERLINTRGLFLLLYDSRLFRSHLHYLWNRIINVVFIRQYNAYKYRSGEKASKERIDLDTVYFPFRKRKSIVTKYIDTWYKGKLLYGTNHFTEKITNLQEKHL
QIAVFEHIPAVTTKSRTYYNKQPNNNTEGLGIEFELMQIISKAMNFKPKYYMPDNITLEKWGINEDNQTHVGLVGEAIQGKAAFYLGDLHYTLHHLNYFDLTIPYNTECLTFL
TPESLTKNSWKLLILPFKFYTWIALVLTLILGGVVFYFLSISYKKHISLYKNQMHFQNTSMKKEIKGLYLFTEIENSILYTYGMLFQISLPSLPSSWAVRVLIGWWWIYSILVAVA
YRASMTATLANPVARVTIDTLEQLAKSSIEVGGWNKENKNFFSMSSDLSSQEIGNKFKLIQEEDKAIEKVANGSFAYYENSYLLQHVRVKRQILEKEQKENITTVDISSKHNL
HIMEECVINMPIALGLEKNSPLKPRVDTLIRRIIEIGLVEKWLSDVMEWSKIMEIRQEAESEKALVDLHKLQGAFIAIIVGYILAFMVLIGEILYWKHIVLKDPKFDKYHLDIFY
SINNPKI
>DallIR8a
MLNLWVVTAIALSFFSYCAESQSQIKLLVVVENLDDGVLKLLNDVIPAAEKAHESEKVSVDVKSVQVDRHNVEGSFQQVCAVLFDGITLVLDITYTGWDRLQALAHNNSIL
YLRTGGSIIPYVQAIDDLLLKKNATDVALIFENTRELNESLYYLIGNSIIRLVVIDDLSEVTVARIRLMRPSPSYYAIYSSTAKMESLFKTAMSGGLVKRHGIWNLVFTDMKYRE
FPYIAGPDTLNTMVGILSMNPSVCCRLIREYPCTCPHNFEIFPKFFERLIFLLVSTITQIQKSGIDVEPIKGQCITTDESPTIDPEKNATLWTFYTTLTTKIESDNDVFEFSKDRYLI
RMRAEINLETLEGGNLEMLGNWTKKNGIVAAPGKDIQPAKRYFRVGTAEALPWTTKKKDPVTGEIMKDKDGKIIWEGYCIDFIQKLSEKMNFDYDLVIPEDNSFGHKLPSG
KWNGLVGDLSRGETDIAIGALTMTSEREEVIDFVAPYFEQSGILIVMRKPVRETSLFKFMTVLRVEVWLSIVGALTLTAIMIWILDKYSPYSARNNKRIYPYPCREFTLKESFW
FALTSFTPQGGGEAPKALSSRTLVAAYWLFVVLMLATFTANLAAFLTVERMQAQVQSLEQLARQSRINYTVVANSTTHQYFQNMKNAEDKLYNVWKEITLNSTSDQVEYR
VWDYPIKEQYGHILQSINTVGPVKDSKEGFRKVIESEKAEFAFIHDSSEIKYEVTRSCNLTEIGEVFSEQPYAVAVQQGSHLQEEISRKILDLQKDRYFETLSATYWNASLKGTC
SVADENEGITLESLGGVFIATLFGLALAMITLAGEVIYYRKRNAAEGIKSKEANGEHVRSGEDKLTKGRLGFKPAPTIAFIGKPHTGPRARISHISVYPKNFPFKE
>DallIR21a
MRVANYIFIILLIFTRLEIIGSHTAHMRRSMKVDLRTDSLDRLLSYILKEYFGGCVIIIIYDDKTIEQQPGLLQGLYTSFPFASFIQKSTNTSLGQVPIIFKDKCYNYMIFLDDVYYI
ENVIEEETVNKVLLITESTPWTVKEFLKSFISRSYTNLVIITHSMSRRTEEGSFLLYTHRLYTDGSGSSKPVLLTSWIKDHMTHKNIDLFPEKLSGGFRGHRLLISTAHKPPFAIRT
DRISLGQIGWDGIDIRMIRLLGKVLNFTADFRDPTASTSPTYAALMDVEKRETTLAIGGIYRTNNVTTRFDSSFSHMEDCAAFISEASLALPKYRAIMGPFQGAVWALVVIAY
VIAVIPLATNTNYSILSLVTHPSRFMHMFWYVFSTFTNSFVVKNPLLDTGIAKNSTSLLIGIYWVFTIIITSCYTGSIMAFITVPVFPEPIDTAEQLLKKNYDIGTLDHDGWEVWF
NWTKIDEPVAKKLLKNLQYVSTVKAGIGNITQAFFWSYAFIGSKILLEYIVQEQFTPSWATMRSPMHISKECLLNFGVTFVLPKNSIYTEEFNKVIIRARQSGLAQKIIRDVKW
DVQRTAEGLLLPVSEEYKRRKIPVQDRSLALDDTQGMFLILGAGTLLAFLTLSIECCVHLWKKRYSNDVGHTMDGSTVVSETITPKMNHFRGLDMWAPDGSTSKRRRFSISS
I

>DallIR25a
MTTLQNQCEKHQFRTQITTKHIDEAVWNIVINDEMNDVANRSVNNALKNIRDSHTDWLREVIIIQINGSDPHDTLDKICTAWDRAVRDGGHGVPDLVVDVTRSGFGAETVN
SFTAAMGVPTLSTQFGQEGDLRHWRDLKEDQKGYLIQVLPPADLIPEAIRQLAITMNISNAAIMFDENFVMDHKYKSLLLNVPTRHVIVRTKEVGAIDAQLSQLRDLDIVNF
FVLAKEEVLTAILDAAEAKNFTGRKYGWFALSLDEFIPKCECKNLSILFFQPQSTSFSQEQLGGLTSKGLLQPPLITAAFYYDVTRLAVQAMREATKNNLWPIDPQHITCDEFS
GNNTPKRNFNFLEKLRNVNREVQFEQTYAGFHWGSKNGEHRANFTMKMSLAVIDDGNAISTNVLGEWPAGIDSPLKMLNHTAVKSFRVVTVITPPFVMYDPETDTWSGYC
IDLLENIRNILKFEYEIREVADREFGWMKPDGTWNGMIRELKDKRADIALGALSVMAERENVVDFTVPYYDLVGISILMQKQKAETSLFKFLTVLENDVWLCILASYFFTSF
LMWLFDRWSPYSYQNNKEKYKDDEEKREFSLKECLWFCMTSLTPQGGGEAPKNLSGRLVAATWWLFGFIIIASYTANLAAFLTVSRLETPVESLDGLSKQYKIQYAPIRPSQ
AYTYFDRMAKIETRFY
>DallIR64a
MHPRWWILVVLLPQCSTGSDDVTGGLTRDYFGGLLIRQIVAFGCWDSEEGVKFSRLIMGDDHSLTYVSIQDDLDMERILKVNYYRLGIFLNLDCPGSEKIFDQFHRQQLRHN
ESYFWLMPTTRTGLPKYFEHLPLNIATEMTAALKKSDGEYTLYDVYNPSYRHGGELNVTRMGSWSVKNGLNIELTEYKYRRRGNLYGLGLNASIVVDHPAVPDYETYIHNP
INPHYDTMHRYNFALTRQLRDYYNFTMNLSRGTTWGYLINGSFNGIIGDMIKGIVDFGATPFQFKPERIDVIEYTVQGWLARPCLIFRHPKKNNLSNPFLRPFEMKVWYWIA
IFGIVLWSALYLTVKVETKFDPQKSVNTIDTHPASETVLITAAAICQQGLSDGPRCISGRITFLTLFIWGLMLYQFYSASIVGSLLSGSSNWITTLQDLVDSDLEVGIEDMAYNH
DFFATTTDPVAQELYNKKVAISKKRKTEPYFSAEEGIKLIQKGGFAFQVDVATAYKFIEETFNVDEICDLVEIQLFPPKHTATGTAKHSPFKKMITYGLRKVMERGTPRRLLNIW
MHRRPQCPESHKANPLPVVLTEFSPALFLLIIGIMFATLVMAVERTFLSFPSLNLLDPESR
>DallIR64a.2
MRHDAAVSFNSITLNMNLEIILKVNYYHLGVILNLDCPLSDSVLHEFSDQLVFNETYVWLLLTTAPSPPSNRLRHLPLSIDTETTVATRDGNKFTLYDIWNPSYRHNGLFHVV
YKGRWSPEEGLINELTQYKYTRRNFNLTPLNFSITLRHPPLPDLETYMTTPINPQFDSMHRYHYALALILRDIFNFTINLHRASSWGYMKPDKTFDGILGDIAKKVIDISISPFR
YRPERFDVAEFTVQTLLVRSFFIFRHPSSASLRNNFLKPFANELWWMILMVSIVYWISLLITIRIQKHYDESRSSLMVPASEATLTTVAALSQQGVSDDPQIISGRIVFLSLFIWG
LLLFQFYSASIVGSLLTTPPHTITTVKNLTDSDIDCGAEDVAWAYDMFKTTPIAEESELYEKKIKPFENTPKNKYFSIVKGMQKVQKGGFAFYTESAPAYKQIKDTYHEDEICE
LQEIQSHPAREVTMVTAKHSPFTKMVIYGLRKIVQHGLSAHVLETWYAPRPRCPETHNSKPTAVKFEQFVPAIFLLLMGMSVSIFVLGIEYLYFYQTEDASHFHQEYSARATQ
TNPTPEHCTEEY
>DallIR64a.3F
KSDRILEDFVRDYYEANNVHQIIVFACWNDFDAFQLTRNVMKFDTTVSFIPIPSAVDFEKILQVNYYHVGVMLDLDCAESGKVQEEFSKQLVFNETYLWLLFTEALTPPTIRL
RQLPLSVDTEMTVATCEGDKFDLYDVYNPSYRHNGAYNVTYKGQWSPETGLIDVLTQYKYKRRGNFQLLPLNFSIVLTNPPKPDFETYIRTPINRQLDTMSRYHYSLVLLLR
EMVLHXSATPFQYKNERFDVAEFTVETLLVKAMFLFRHPKDATLRNNFLKPFTNDIWWMILAVGTVYWVSLWITVKIQIHYNESYMNRKEARGAFEIPGSESGLIALAALS
QQGLSEGLQIISGRIVFLFLFLWALLLFQFYSANVVGSLLTSPPRTINTIKNLSDSQLDAGTEDILWIYDYFNNYFQIMKTPSHIELYQKKIKPSSKRPEGSFWTAVEGMQKVKK
GGFAFYIDTATGYNLAQDMLEENEICELHEMPMITWAKVTLLTAKRSPFKKMIIYGQIVQYGLMIKQFSIWYTPRPKCPESYSSKPIPVGLKEFVPAIFLLLIGVSFAVFVLIIEF
LHFWR

>DallIR64a.4
MKVVALFLLAITLIKFTKSDKILAEFIRDYYGSCDIHQIVIFACWNYAADISQSVMGLDTVVTYQSTMNDVDVTTILRIAHFQVGVVLDFDCPFSKNILDKFSNQLHFNDSYY
WLVLSRLTPISVNFLQHLRLTIEAELTFAVREGDTFKLHDVYNPSYRHGCDVVIVDKGKWSPGDGLSNKLTQYKYERRHLHGVTLNFTVTVANPVDVDIVTYLSSQKNRGL
DPMQKSHYNLMLFLQYLYKFSITVYLSPLWGILVNGSYNGIMGDMVSNEGVDMSISPFEFDWYRLHVVEYVVPTWFTDFTFSFLHPTKSTMRNNFLKPFTQDLWWAILLV
GAVYWVLLLLSLMLEQHHETGRQNINAGAVETGLTTVAALSQQGLSDSPHFPSGRITFLSLFLWALLLYQFYSASIVSSLITAPPRWIKSLKDLTESDLEVGAIDVSFFRDWFKI
TNNSDIRDLYNRKMNSSVSNWPNAFMSVPAGLKKVQEGGYAFLTETASTYRIMRETYSEDEICAVQEIRPQPRNKMSPILPKNSPFKKMITYGFTKIIQSGLLAHVQHYWRG
SVPECPESYSSMPTAMGMKEFSPALFLLCIGAGISIVTLLIEYFHFYLEDQRDRATRHLEELPQ
>DallIR64a.5
MKVFVAILIAFALIHACQSDNILAVFMRDYYKACDIHQIVIFACWENAAHLARNIMGLDTVVAYQSITNGVDLRNILLVNYYRVGVVLDFDCPFGESILDEFSTQLHFNESYH
WLVLSKFTTIPVNYLGRLSLTIASELTFATRADDVFKLYEIYNPSYRHGGAVRIITKGEWIPGTGLIRVQYSLSEYKYKRRADLQGLSLNFSLTLANRPLPDLLTYLSSPTNREL
DPMTRSQYPLALYLQDMYNFSMKLHQATTFGYLVNGSYNGIIGDIISGFIDMSITPFEFHVPRLKVIDYAVVTWYADTTFVFLHPKSSTLRNNFLKPFTNDLWWMILLVAAIY
WLLLLLSLLLEQHHQAGTRDASLSAIETGLTTLAALSQQGLGDSPNFYSGRITFLSLFFWALLLYQFYSASIVSSLMTPPPRWIKSIKDLSESDIEVGAREHPYFHNYFEKMTD
PDCIELYDRKMKSPTKNRNGFLPVDRGFKKVQEGGYAFITESAVTYQILHDTFSEDEICALQEVRVGRPRWLAPILPKNSPFKKMIIYGLRKMVQSGLLNRLQKIWRASRPQ
CPESYNTKPTPMSMKEFSPALILLCIGVIISVVTLMMEYLYFYLETRLNSIRMIVEGSTEQYDDQNAEVFT
>DallIR64a.6
MGFWAVLLSSVVLSRCVQSERVVEKFIRDYYDAENIHHINAFGCWDDMDASEFSRKLMSLDNAVLYTPISPHVNLHRILKVNYHYIGVVLDYDCPMSDFILDKFSKELVFN
ESYFWLLLTNSSSPPNDVLQKLPLTVESELSVATSSGNSFELWDAYNPSYSHGGVLNVTYKGRWNPEDGLKNELNQYKYERRSNFNLLPLNWSIVLRSHPASDLELYLTTPV
NRHLDTMSRYHYALVLHLRDFFNFTINLQIHESWGYLVNGTFGGLIGALMKGQADASVSSYQYKLERMDVVDYLVETLNVKLRFFFRHPRSNDLQNNFLKPFAIHLWWVII
AVGFFYWGIMVILKKFEIYYERIEENENTVSSTALTTIAAISQQGLSTPPTITSGRLVFFSLFLWTLLLFQFYSASIVGSLLAPPQRWITTLDNLTDSNLECVVEDMPYMVDYFAT
TANPHTKKLFERKIKATKKKPKGSYMPAIEGIQRVREGGFAFHINVAAGYKIIEDTFKENEICELQQIDMVGQCLTSMVTAKHSPFNEMFTWSVRKAVESGLTKRLDRVWNQ
QCPQCPKSYSSKPTPVSMQQFSPAIFLLLIGFGSSFLILLIEYLHYWKCSDYLSNVEDTNSVAGTDGTEEEQSYMVEGFHVADDGQAVIF
>DallIR68a
MTQKSKCIVFMIDPYYRKLIRYNWAQLRVLPYYSIYVKESEEFTPPRRRVEDILSESKNDGCDAYVLLITNGLQVSQLLEYAERNRIINTRGNFLMIHDTLLFDVGMKYIWNR
ITKVMFIHRFTVLTRRSNKTTMKEWFNLETVSYPVRANNFVKTRYVNTWHKGRMLNKDVDNPFTNKTLRLERRSLRVAIFEHIPAVTRYSRQLQKHYRDYSEKASGVEFEI
MRVLSDKMHFKPNFYTPVDIEIEKWGTKDDNGSYNGLLGEAERGNAEFFLGDLHYTLRHFELLELSYPYNTECLTFLTPESLTDNSWKLLISPFRLYAWIAVILILLLGACAFH
FFALFYQNQIMPYVRNTNAEGIQMRGLTLFTDMQNSMLYTYSMLLQVSLPRLPRPWALRVFIGWWLLYAILVTVAYRASMTATLANPVAKITIDTLQQLAKSRISVGGWSEE
QRDLFGASLDPDLIEIATRFELTLKEDDAVARVANGTFGYYDNIQTLQEARAKRQLLEEMRKKKSTREEKVIDDRNLHVMSECVIYMPISIGMDRNSPLKPQVDEIVRRVVE
AGFVEKWLSDVTEWSKITELKDESPAAKATVNLHKLHGALVALGIGYFLGFLALIIEKIQWKYFVMKDPAFDKYQMDVFYSMSRSNFINKGGIASCRKS
>DallIR75u

MMREIRAIAIAWILLNLLARGRSDRIDAIIGNFITDVSSSLLVSSSFTGFFCMESDDIVKFSRQISRNYLLHKIASFNDSDLIDISQIATHNHYFVVDLDCPDGADFLIKANTRRLF
IAPAKWLILRDLRNQDELRGLPYLQSMVEMNEDTLISLLSNFDIFPDSEVIVGQRLNETTVQLSSLYRPNSDHSLTIENLGSWDDEGGLCLCSHDQSSRRRVNLQGTVLKTSL
VMTDLNTINHLTDYQDKLIDAVTKASYMWIVYLSERMNATFNFTVERTWGYKNEDGNWNGMIGLLDRGEINIGGTATFMISQRIGVVDYVQLYTPTGSRFLFRRPPLSYVS
NIFTLPFARSVWIAIVAFLTISFGFLYITMKWEWEKMQAIPLESRLGGDLEGKPTVTDNLLVLLGAIFQQGFSCEPRTISTRIVVLMVLLIALSLYAAYTANIVALLQSTADSIKTL
DDLMNSPLKIGIFDIVYNRYYFGAFEDPVRKEFYERLVKDKPAVWMPLEEGIRKVREGLFAFHVDLGFGYQMMQETYAEDEKCGIEEIDYLKVYDPLLVIERQSPFREIIRVG
ALWIAETGLKPRVASKFFTQKPPCIGSTSFVSVGIIDCYVAMLAIVYGCAISVGILLLENLWRRCIGERRSHDNTPLKTSLPESSLTRDEKSATQSSTSLQIEELFG
>DallIR75u.2
MSLIDPFYRYQINENKMFSAPGKWLILQESRSSFPQADHSATPTNETQLRGVFENLNIFPDSEVTIAQRIEDTVVKLVSIYRPNTVANLIFEDRGVWSKGNHIQLHNNEETSRR
RTNIMQTQLRAAAVITNPNTMNHWEDFQERRVDGVSKVDYAFTKVLVARMNATVHFTFTPTWGYKSSNGSWDGMIGSLLRNEIDLGGTGIFITEPRLEVVSYINLYTPTRVR
FIFRRPPLSFVSNLFVLPFARNVWFAIVIFCCLGYMVLYFSLSQEWKMIEKIPYEERLWGDLEIKPAFGDNFLIVIGAITQQGSAYEPRTGPARAVVFMLLVTCLSLYAAYTANIV
ALLQSSSDSIKNIKDLMESPLQLALQDIVYNHYYMGKFDDPLRTEFYERRIKNLKNPYMSTEDGVEKVRTELFAFHTDLVMGYDVVKSTYEEDEKCGFEEIDYLYVSDPTFII
QRQSPYAEIFRVGGLWLGETGLAQRFIDKIYHKKPECNNQKKFISVGTVDCYAAYLVIVYGLVVTFTILLCEVLWFKNFDKRSSEIDDEPENHDVASITSAAASDQTFGEELNS
TILEEIM
>DallIR75u.3
MLIVSVIAVLIFSVGVSGDQEMDKILQSFIVDVITSLYASSSFTVFHCAKPDDITEFSRFMSRHYQLHEVATISEAYKFRFIESPLSHQNFYVVDLGCAGVHELLIQANNTGSFV
GPTKWLILQDLQTDSNATANENFHSGATDQAELRSVFEDFHVFPDSEVIVGQKISDNSIKILSVYRPSPVRSLIIEDRGTWNSIDGIQLRDHDVSSRRRTDLQQTPLKACSVVT
HPDTMNHLEDLKDKQVDVITKVGYAFSKLLAARINATVTFTFAASWGYKEKNGSWSGMIGEIDRNEVDFGATATFIIASRIDAVDFIQLYTPNRIRFVFRRPPLSHVSNLFTLP
FTESVWIGISVLSCIGFVVLYLSMAWEWRIVKDLSHDEKLTGDLEIKPAFGDNFLILIGAVTQQGSAYEPRSVPARIVIFMLLVFCLSLYAAYAANIVALLQSTSDSIKNVEDLM
NSPLKLGIQDIVYNRHYFGSFEDPLRKEFYERRIKKQKDIWLSLTEGIGRMRNELFAFHTELTSGYDVVQSTYEEDEKCGFEEIDYLYVSDPAFAIKRRSPYREIFRVGGIWLQ
ETGIKERYIRIMYNKKPPCNNQKKFVGVGTIECYAAYLTIGYGMLLTFGILLFEIIWSKR
>DallIR76b
TPPYSSTIRKNGSLRGEGYAFEVLDLIAKKLDISYEIVQPRTPGLGNESAGLISLLKSKEIDVAVAFIPMLWKFTEFTRYSPIMDEANIVGMMVRPAESASGSGLLAPFDTTVWI
CILISLLVIGPIIFLFTAFRSYLWNHTKVDKYDFTSCIWFTYGALLKQGSSITPVNNSTRFVFATWWIFITILTSFYTANLTAFLTLSRFTLPYKSVEDIMRKRVPWFFEKDRTIDNI
LDTLQILIEVGIIKYLEKRNLPKVEYCPLNLKSTERQLKNSDLTLTYKVIAAGFISACIMFIYEMIRRRQHVSCLCCGKSCSFCWPGIETPDDPILPPPVVLQSENNYVEKESTIR
HTINHNFNNDSNHNNSNRHPQVEQISLIDESIYGAAASARKTYINGRDYWVITAPKGDKRLIPIRTPSALLFQYTT
>DallIR93a
MALILIVVLIYSCRFVVGFNDFPSLMTANATMVIVIEKTFYERKILIKETSIFTREAYEKSVASFTSAATKIARERMNISGLSIHVAQDMGANLARDYTILLSVATCSSTWELFGR
AKKEKLVHLAITDLDCPRLPKDEGISIPLIEPGEELPQIFYDMRISRGLDWKRAIMLFDESFEQDSIGKVVQAFCNELPKSDLGLASSSLYFLKRGKSEISTKRIIKEILAALPPR
KPIDDHIIVVAAYNVISFILEAARSLRMLSTGSQWLFVVPDMAKYTSGNVTYLIELLGEGENIAFLYNDTNLNIRSDQSCRTGATCHVRELVGALGIALEKSLSMEIELYNRVT

EEEFEQAGTTKFDELYNDTKPGGRGGTCGRCIKWTVVSALTWGNRIGSEADIEPHTLLGTGVWTPDPGYESKDYLFPHVMHGFRGKTLPVVTYHNPPWQFQMTKTEEITE
STKAQWDGFVFDVFHELSKSLNFTYKIVAVETPPEINLVKSNPLKAAMSAAEKVPEKVTELVRSKSVFIAACAYTVGVYRKDTTIKFTLPMTIQTYGLLAPRPKPLSRVLLFA
SPYTNESWALLTTAIIIVGPILYLVHTFSPRTIDEAVKNPQEPVYIGLSSPSRCTWYIYGALLQQGGMNLPKTDGARLIVGTWWLVVMVVVATYSGSLVAFLTFPKMEAPIKNV
DDVIERRGEITWSLPQDSFIEDFLTVSNEEGLVDYKRLLRGNEPHAQTHDTVSYEDNIHDVKGGKHVVIDWKSSLMISGRNDYIETGRCAFSLGTDVLFLEEPISMVVPTDSP
YLGLINVQLQRMHESGLMDKWIANRFPTQDSCSDSLMGGFEAANHKVDLEDMQGIFFILILGYIMGTVILGYEFFRQHRQLAKERKVIQPFVQ
>DallIR101
MKFSIPLIFFITFILAFFKLNGAHYHGPLLKAVHSKYKTNGGIIVSGTGHMSFGRTTIWHEAVRMLSNDGIFTVIVNLRQFGDKLKSYSGGRMSLLIVIAIDTVEELHSFESMSK
DFHLSYAVWLILFSRDASQDVCDFCRNPHDSLSNLGFGSKVLVSCCDSDMIEEWWSIGENRIERQELGRLMDDNQGILWLSEELVNRRRYSLNGQELRIVTVQDSFSFQEKN
GTYYGFLGEILKELREAMNFTVSIIYEEGYGALNLKTGNYTGYIGRIHRREADLGVAQYFIRKELLHVLSYTSPVLSGYFEFHFRKPDAVNVPWNVYLKVFTGHVWMAILS
LILTSTLLLTLITYRRRSHFVPLLFENHLIVWDIYCQQALPAFPDKTPLRIVYISLALSALVTLSAYSASIISQLAVFSYSPFRTPEEFVEDGSYKIIRLRNTPSHSTVMDYELSDEK
LMKKFESLLQLRDLNPRNPQEAFEQICRERVAFLAFETAKTAVNNEIPCEITSFKFGPVYHVAMLMPLGSSYMDLINHHIQQFKDNGILRRLKRKYSTVPRGNKSALAPVEIH
EIAPILFMLAFAFLIAFIIFISEMNYDPFTKELHKPRRRKTRKRRAKAGHFHLRHRI
>DallIR102
MKFANSSIFSIVSIVIFFELSEAHYYGPVIKDVHDQFGRTEVIIVPQTNYFSFENIMIWHETTRILSNEGISTVILNTRHFEEKLKTYNKETTRSLIVIALDTIEELHAFESTTQDLH
MSYAIWLIFFTRDADRDVCEFCRNPQESLSNMKLGSRILISCCAFNTIEEWRYAGKNRTERQELGRLNRDDRGIVWFSNKQVADIHGGRYSLAGRGLRIVAVKHCPIFWEKD
GHYYGVLGEILRELSQAINFTVSKIIWEDDYGAWNPETSSWTGAIGRIHRQEADLGVSDFMFSTHRSTAVAFTSHFTSAGFYLYLNKRYMARLHWNAYFKPLSMDVWMVIF
GLILTTSILLNLINYTRRSHFFPLLFQHCLYAWGIYCYQALPKFPKGTSSRIVYASILLSSVVTLIAYAAAMTSRLTVVSYIPFKTLQEFVDDGSFHVIKLNVSQDFDHYKFFDQT
LTKKMMSLMMPRNLLPVTDEEAFEQVCSKRVGYYLNDMAKKAIEAQGIRIPCELSSIKYGKTQILAMIMPHGSRCLDLVNYYIMQFRSNGMLQRLEHKYYKELKRNDLKY
SSVSLRGVAPLLLILAIGFLIALIIFIIEQNANAFRKKLRSYQKRRTTFLKTRKASLFLANDCNFKKRLRNLKQFIGRMQLK
>DallIR103F
MYLHSVIMVKLNEAHFYGPAIKAVHDKFEANGVAITAGINHLFWHETSRFLSNEGISTLIVSFQQLRNIWKTQPTRTTRSLIVIAFETFEELHTFELIIKKFRMNYGVWLIFFMR
DADREVCHICCNPHGTLANLKFGTKILISCCDSNMIEEWWSIEENLPKGQEIRRLMDENFTISWFSDELINGDKYSLKGASLRITAVTQSVFFRKKDGQLYGFLAEFLNKLSQ
AMDFKVSEIIWEEDFGVCITGSSDCTGSIGRVQREEVDLGVAAFSATVERHNLVDFTFPIITGNHEIYFRTYNAINVRWNAYFKPFAADVWIFIICLILMTAMFFTLIRYKRESSF
FPLFVDRYLHIWGILCHQSLPAFPRETPLRIVYLTMALSALVFSSTYAASVTSNFTLSFYSPFNTVEEFVKDGTYELSFSDNKLKKKMLSLLRSEDSLPNSSQEAFEEVCKKRV
AFFTHEATKRALFNLIPCEISSIRINTMNPMSMIAPRGSKYTKIINHHIHQFKEVGLLRRLENKYIKMENDKTEHPPISLQEVQSILMILVTGCLLASIIFIIELKLYTYCKNL
>DallIR104PSE
MKFFLLLILLGSSSLVFVNLNEALHHRPLIKDFYDKYKSDXVWIILGSNNLFEKTTIWHGVIKMLSKEGISTRTADFNAFKFMLKTVNENNMRPLIVLVMNEIEELCSFESIIG
KFYTDYATWLILFTADSSQDVCGFCRKPYGSLANPIFGSKLFTLCCNSKVIMKWRYSETNRSRRLEIGRLMDGNQGIVWSSDELDYNSKYSMDGRTLRVFRVRVRTXMISTL
TVSSGSTFNTMDEFVKDGSHKLIVLDSTLVTDMYKVITDSSLYYGNPSRLLNEGHKGQNKCQVGQNEGHEISPLLNRIPCEIVSVQTGVIGTAGMIMPLGSKYRTALNHLQQ

LKXGLLRRLEHKYLRQFERGKSGHPPVTVERVVPILFILAVGFLIAVIIFTVERNVYLFATILRDRKRRRALSGKCKTFSFPT
>DallIR105
MKFAISTIFLSVSIVIFCELSEAYYYAPVIKDVHNQFGRTEVIIVVPQTNHFSFENIMIWHETTRTLSNEGISVVILNTRPDEKLKIYSEKTTRSLIVIALDTIEELHAFESITKDLH
MSYAIWLIFFTKGDDRDVCEFCRNPHKSLSDMKLGSRVLISCCTSNTIEEWRYDGKNHTERQELGRFKKDDRGIVWFSNEQVADIHGGIYSLAGRGLRIVVVKRARMFWEK
DGYYYGVLGEILRELSQAMNFTVSEIIWEDDYGVWNPETSSWTGAVGMIHRREADLGVSDFLFSIKRTTAIAFTTHFTSADLYLYLNKDYTARLHWNAYFKLLSMDVWMV
IFGLILITTILLTLINYTRRSHFFPLLFQHYLYAWSIYCYQALPKFPEGTPSRIVYGSILLSSMAIISAYGAVMTSRLTVVSYIPFKTLQEFVDDGSFHVIKLNVSQNFDQYEFFDQT
LAKKMMSLMMPTNTLPLNDQEVFEQVCSKRVGYYLNDMARKAIEAQGIRIPCELSSIKYGKTQILAMIMPQGSRYLDLVNYYILQFRTNGMMERLEHKYYKEFKRNDFKY
SSVSLPGVAPLLLILAIGFLIALIIFIIEQNANAFRKKLLSHQKRRTTFLKTRKASLFLANNCNFKKRTRNLKQFIGRMQLK
>DallIR106
MQFFIFLILLCTSSVIIVKLNEAHFYGPAIKAVHDKFEANGVAITAGINHLSFEQLTVWHETSRFLSNEGISTLIVSFQQLRDIWKTQPTYTTSSLIVIAFETFEELHTFELIIKTFH
MNYAVWLIFFMRDADREVCHFCRNPHGTIANLKFGTRILISCCDSNMIEEWWSIGENLPKRQEIGRLMDENFTISWFSDELMNGDKYSLKGASLRITAVTQSVFFRKKDGQI
YGFLAEYLKELSRAMDFKVSEIIWEEDFGVCITGSSDCTGSIGRVQREEVDLGVAAFSATVERHNLVDFTLPIITGNYEIYFSKYDVINVRWNAYFKPFAADVWIVIICSILMTA
IFFTLIRYKRESPFFPLFADHYLHMWGILCHQSVPAFPREAPLRIVYLTMALSALVISSTYAASLTSTLTLSLYSPFNTVEEFVEDGTYELIVLDSALINDMYKFSDKKLKKKML
SLLRSEDSLPKSPQEAFEEVCRTRVAFFTHEATKKALFDLIPCEISFIRINITNPMSMITPRGSKYTEIINHHICQFKEVGLLRRLENKYFIKMENDQTVHAPISLQAVKSIFMILVT
GCLLASIIFIIELKLYTYCKNLCDQRRRKTLLGKRGKFPFSMYNFVIRKFP
>DallIR107
MLLGTSSLIFVNLNGALQRGSLIKHVCDRYNSDKVLIILGSNNLLFEKSTIWYGVINTLSNDGISTSIVDFNALNLSLKTVNAKNMHVLIVLVLDTIEELKNFESIAEGLYTSYA
IWLILFTSDSSQDLCEFCRKPHGNLSNPKFGQKVLTLCCHSNVIMEWRYSETNRIRRLEVGRLMDGKPGIVWSSDELDHNRKYSMDGKTFRVIGVKTSIMLWEEDGVFSGIL
GELLTELSQAMNFTLSKIIWEHDYGIWNPKTSNWTGAIGRIHRREADIGVSDFFMTTQRYAAVSFTSPIFFTPLKLHFKKRHADNLTWNAYFKALTIDVWVVILGLILITPLLL
TLIRYRRRDNFFAFLFEHYSYVWGIYCQQGVPVCPQGISPRIIYLSILMSAMVTLGAYSGSMISTLTVSSDSTFNTMDEFVEGGSHKLIVLDRTLVTDLYKFTDERMRMKMMS
LLKPEHSLPQSIHEAFYQVCREKVAFFTVEATKTALLNRIPCEISSVQTGVIGTAGMIMPLGSKYRTALNHHLQQLKRIGLLQRLEHKYLRQFERGKSGHPPVTVERVVPILFV
LAIGFLIAVIIFTVERNVHLSATISRDRKRRRVLSGKCKTFSFPT
>DallIR108
MKFFILLILLGSSSLIFVNLKEALHHGPLIKDFYDKYKSDGVWIILGSNNLLFEKTTIWHGVIKMLSKEGISTRIADFDAFKFMLKTVNENNMRPLIVLVMNAIEELWTFESIV
KKFYADYTTWLILFTGDSSQDVCGFCRKPYGSLANPKFSSKVFTLCCDSEVIMEWRYSETNRSRRLEVGRLVDGNQGIVWSSDELDYNSKYSMDGRTLRVVGVRMSMLLR
EKNGKLSGILGELLIELSKAMNFTISKIMWEDEFGVWDAEKSNWTGAIARIHHREVDIGVSNFIMTLQRYDAVSFTTPILFGPLKFHFKKRDINYLTWNAYFKALAIDVWMA
TIGLILITPILLTLIRYRRRDHFFPLLLEHYSYIWGIYCQQSLPDCPKGTSLRIIYLSILISAMVTFGGYSGSMISSLTEYSGSPFNTMEDFIKDGSYKIIFLDPTLINDIYTFRDVSLR
KKMMSLLKPSHSLPKNIEEAFHQVCNERVAFFLADVMKKDVVNDIPCEIYSVGTGSIGTIAMIVPLGSRYLDPVNYHLQRFKRNGLLERLKHKYFKLSQRRRSNHPLVTVEA
IVPILLILAVGLLITIVIFIAERHAAHVLVTKLRDRRELKVSLRKRKSFSFPMYDYVP

>DallIR109

MKNSVMLLLCFTGIRPIISTHTCNDVNCYGSLIAQVYNEYNTAGILVASTTTHLLFQTLIYWHEISTTLSDQGIPTVMVNFAEFTERMEFYRRHPNRPFVVIILHRPGDLYFFSQ
ITKSLPMNYVLWLILFIGDADKDACNFCRDPHENLLNLKFNSEVLIMCCNSNIIEDWWSVTRNGTNKGQLGRWIEERNEIEWFAHKSIHRRRTSLEGRAFRISFVQDSSYIWI
KDGHLQGFLADVLRELAKSMNFTISTATVEDTYGILDPGTSIWRGVVGQLQRQATDIGVDGFSRTSARRSVIDFTVPIITVDSRLYIKKPDGTNVQWNAYFQAFTRTLWAVIIA
VILIMPVFLTLIKYNRRFNFFPLIVEHYLHVWGIYCQQGLPEFPDGMPLRILYVSIFISALVVSSAYSASLTSFLAVSQLPFNTMEQFIKDGTYGLTAVYGSEDYNTFKFSNDTVL
RQMMSFMKPKGSLPKSYFEGFSQACKERVAFYTHYEITKGREMYVMPCEMVSFKTGSNQLSGIILPLQSEYRTFINHHLQRFKTNGVIRRIAQKYNRNDEPPKTVHTPVHLR
GIVPILGILIFGFIIASIIFLVERSFYSFRNKLRRRQMRKI>DallIR110

MLSLTSLILWTTCMASPIVFSHEVTHYGSLIKNIYDKYGRTGVIIASATSHLSFEKTSTWHELTGMLSNEGISALIIDFRQFENRLKVYIEKTYPPLIVIDLDTVEALHSFEIITKN
ADMSYSVWLIFFSGDVDHDVCKYCREPHGNLFNLNFGSKALISCCASKMIEEWWSTQKNHTKRQNVARLTNENPGIVWFSQKLISDGRQSMDGQILRVTALADLQIKNKR
NKDLYGDAGKFLAALSTVMNFTVPNIIWEKTYGAWNRETSKWTGILGRIHRQEADLSINSIVMTSERSNIIRFTTPIMSGVYQLHFRKLDSARFTWDAYFKVFAADVWIVIIG
LILTAPIVLTLIEWNARKSHFLPLLAKHYSFVWGIYCQQGLSDFPDETPLRIIYISLMMSALVVSATYGASFMSILAVSSSFSPFSSMEEFAGDGRYKFIVPRNSSSYYEFKNSNL
TLMKKMMSLMKPVNSLPQTFVEGFQQVCKDRVALYTHEFRKRLLSNLIPCEITSINTGKMETVAMIVPRNSPYIEPINHFIQELTFEGIFRKLVKNSNPQHYEYGFQPAHLQGI
TPLIVVWISGVLIASFIFIFERTSYLSTQESHIRNRRGTKNNAKHPH

>DallIR111

MNFPTLVLLLSTLNAISTIVSDKFSDFNYYGDLIKNVHDKYKGTGVIIASEMDRQPFERITIRHETTRKLSNEGVSTLFLDFSQFENRLNVYMKETDPPLIVIVLDTIGALRSFE
TIAKGLDMGYFVWLIFFSRDAGQDVCDVCCHPRGNLFNLKFNSKVIVLCCDSKIIREWWCVRDNRTWSQEIGHTNNQEITWVSDELLRDRRKSMHGLVLRVAAVKGTALF
LERDGKYYGYIGEILAALSEAMNFTVSQIIWDNDYGYWNRKTLNWTGVIGRIHREEADIGVPDFLITDARYNAVNFAYPIMNGAYQVHVKKLEVAQVAWNAYFKVLTVDV
WMVIIGFILITPILVTLIEYRKRECPLFPLLLEHYMFVWSIFCQEGFTFQIEMSGETSSKIIYVSLSLSTLVIYTAYGALMTSILAVSSSFVPFATMEEFADDGTYKFIVLNNTLFYN
TFKFSNNTLMKKMMALMEPTHFLPQTHEEGFKQVCSGRVGFWASEKIKKIYDNLIPCEITSVGTEMKESSTLITPRHSEFMSLINYNIQQFKYNGMFQRLEKKYWKQSQQN
EKSLSPVHLQEISPFVFALFIGGLIAFVILVIERNSYLSVKKVHNRKSKQVRKNRTSKFPAYSRL

>DallIR112

MKCWIVLILCFTATQPFPLTNEKGKINYYGSLIDIFYHRYKPSGVIILSPEKDYSFESLTFWHAISNEMSKRGITTAIIDSQSFRDRFTFYTAQSVRPLVVILLGSMEDIYTFGKIT
MNLYMSDIAWLVLFSGDSDENACSFCHNPFGNLLNLKFNSEVAIACCDSTIIKEWWSIGNNHTRVGQLGRWIDQNRGIQWFSSEALLQRRRSLEGRAFRVCIVKGSNDAWE
KDGHFHGPLGKLLEALSEFLNFTISTVIIEDNQGYWDSDISRWTGVIGRLVRGEADIGLAPFMMTAERLKVIDFTVPIYDGFSQLYIKKPDTIVLHWNAYFQVPTISRYFSPTVI
CLLSQLIIYLQAFHVNIWVVIIGSILIMPLFLTLIIYRKPLFFVPLIFENYLSVWGIYCQQGLPVFPAETPSKILCISIFLSALIVSATYSASLTSFLAVSSSYLPFHTMEEFVEVGTYK
LTSIKDSSEYLMFKSSNDTVMQKMWSLMKPKESLPANEEQGFYQVCNEKVAFHLTTGYRKEYLNTATTCKITAIETTRIQGSPLVMPFRSEFTAFFNYYIQRYKHSGLVSRWI
EHYHVEQELPRAIYPSVRLEGVIPLLAALAGGFIIALIIFLTERILHRRRDKLRHKKIGKLQLITFQRKQFPRATFHKNLGFRY

>DallIR113

MKLFISLELYLTGIYVFFVAGNDTVVYYTSLFESVHDTYGTAGIIIASSTNYQSPARLTTWHETCTILSDKGIPTAFVSFANFKVRLKFYTRRTVRPLAVVLMRKIEDVHIFEGIA

KKLDMSYPVWLLIFTKDADESVCEFCRHPHRNLFNLRFNSEILISCCDSNVIREWWSKARNFTHATKIGEWIGEDRGIRWATNQSLYSRRHFFVQPTVRVSIVRGSSYIWEKN
GELDGYLGEILKELSLTMNFTISALIKEPYYGSYDPKTSKWTGVIGRIVQHEADMGASEFTLSHERINVVDFTIPIAIGDCRLYVKKLDGARLQWNAYFGAFKADVWALIIGSI
IVTPIVLTVIKYTKKRRHAFSMAIEHYLYVWGIYCQQGLSEFPDETALRILYVSIFISALVVSAAYSASLTSFLTVASIYLPFNSMEEFANDGRYKLIVLQDSADYDMFKMSNETI
MKKMMSLMKPSHSLPQTILGGLQQVCTRKVAFYTNEALKRTLNKNLPCDIVSIKTGKIETLGMIMSRHSEYMGIVNYHIQRFKDNGMLMRLQHKYIQQEDTSEGALLPVG
LGGIAPILFVLIIGFFVAFCIFLVEIIFPPISDKLFKRKKRRLNGQNFQY
>DallIR114
MKVCMTLMLFFGAASLMDSSNGLDTIDYGSLIKNIYDTYGTAGIIIASSVNYQSFSRLTVWQRVTRMLSEKKIATGLVNFEQFKDRLEFYTSRTVRPLVVILFGKMEAINRFS
RLAVAIDMSYPVWVFLFTAETNSDVCKFCHAPHQNLLNLRFNSEALILCCNSLIIDEWWSKTENHTNTRELGKWNDERNEIEWFTENALYSRRSSVEGRKFRVAIVKGSGYI
WQKNAEVFGFLGGLLKELSRSMNFTISSIITTIGYGSWNPETSKWSGVIGSLRRNEADMAISEFSMTHKRLDMVDFTIPIAVGYARIYIQKPNGAHVKWNAYFKAFATDVWV
VIIGLLVTMPLFLTLIKYKRRKFSLFPLVVEHYSYVWGIYCQQALAEFPAETPLRIVYLSIFISSLVVSAAYAASLISFLAVSSSYLPFNTVEQFAADGTYKLVVLKDSSDYDTLR
TSNETIANKLMSLMKPYYQLPQTLEEGFQQVCNGRVAFITNEAMKDAVLAPMPCEIASIKTGRIETLGMIMPLRSEYTALVNFHIQRFKDNGILERLKQEYFRQEDSPELSHP
PVDQWEIAPILCVLTGGFFIALVIFLMEHIFHYLRNKFRNYKIKKFSCRKDRHVHFAREAVIP
>DallIR115
MELPVAVILSFITAYWSTLTTGHVHITYYGSLIDEFYKIHDADGILIVSSTNYHSFGSLTFWHEMSRKMSNRGRAADMVNFQELTPTLQLYRRQNVRPLFVIFIKNMKEVHFFG
RITKTLNRSNSLWVILFSGDSSGDACEFCRHPEGNLFNLKFNSKVIVACCESTIIQEWWTSTGNRTHIGELGRWIDESRGIEWFSDKSLYERRTSLEGRPFHINIVQGSTDIWQK
NGDLHGYLGRVVKALSQFMNFTISSVTIERSYGRWDPDASEWTGVLGKLHRNEVDMGVSSFIMTNERREVVDFTIPTVYENSRLYIRMPGANTVQWNAYFEAFGMDVWL
VIIALIMTTPLLLTFIKYKGRSFIFPLVFEHYSSVWGIYCQQGLPEFPDETPLRIVFISLSLSALVITMAYSASLTSFLAVNVSHMPFTSIEEFVKLGTYKLIAIQDTADYTLFKDSD
EVLMKKMAALMEPPGFLPATHHEGFQQVCRKSVAFHTTHEIREGYTSPFIPCKLAWIKTRKIQASGIIMPLNSEYTAFVNYHLQRFKYSGLLDRWKREYHYRIKDVPEIYHPS
VQLKGITPILGVLTAGLLIALIILLIERIFHRHRDQLQRKKIRTSEVEMLQRMLSRPQSSPSWSRDSNDFRTIYRNRQKKLAQAWIINFE
>DallIR116
MNLFVFLLYISIVMSTVNFLKAKKRVTYYGSLIKDIYEEYRTGGIIIAMPDNTRTRFARLTRAYEISRSLSRDEIPSIAVKFETFKERLASYTDRIFRPLVVIGFYKMVEVYTFQQI
AKDLIMGYPVWLIIFAENADADVCEYCRNPHGNLFNLKFDSEVLVLCCDSGIINEWWSTTVNRTERKEIGRWIGEDPGRYWFNHGSIYGRRKSLEGRELRVTAAKGSAYIW
KENGKYTGFFGEILNELSTTMNFTISEVTPTDGFGSLNPETSEWSGVIGRIHRNEADIGVSPMAMTHSRLNAVDFTIPMFSGKSRLYVRKLDGARVQWQAYFKAFAVDVWM
VVIGLILIMPLFLTLIRYRRGNHLLSQVMEHYSNVWGIYCQQGLSRFPDEISLRIVYLSIFISAVVSASVYSASLVSFLTVFSSYSPFNTMEEFASDGTYGLVVLKNSAQYDMYK
NSKDAFRKRMMSLMAPEESLPSSLAEGFRRVCQERVAFETNEAIRKTMAHSIFCEITAIDTKTIETFGMITPRRSEYLEFINYRIRQFELNGVFRRLKNKYFTKPRENKIDYPKV
HVEGVKPILLTLASGFLITLIIFIIELLFSWIQNKLQRREKQRVIQQQTKYFRFSHRAKSEIYFIL
>DallIR117
MILFKTFISAALVVFIKANEEVYDHTPYLITDIYETRETCGIILAAENYHSFKTLIIWHGLSRTLSDEGIPTLMINFKQFEERFEFYTKRTVRPLVVIFLAAMDEVYSFSKIAKNL
DMSCAVWIFLFNGNISSDICEFCHSPRRNVFNLRYGTEVVVSCCNSTMIEEWWSVMEGHTASLELGRWTDGNHGIQWFYNDSVYNRRHSMEGQEFRIAAQSVYFWETNG

KYYGYLGEFLGELSQSLNFTPKVIWEESYGTWNPETSRWTGVVGTLERNEADLVLSELRMTNERLYVMDYTIPIGVGGTRLYSRKLDAARLHWNAYFQAFTVEVWMVIV
GLILTIPILLTLITYKKKDCYLLPLVFERYLCVWGIYCQQGLPEFPKEASLRIVYLSIFISALVSSGAYSAALISFLAVSSTYSPFNTIEEFVEDGSYQLIVLKDSPDYDMFKNSNQ
TLMKKMMSLMKPIDLLPQSFQEAFNQACTQQVVFYTHEAIRRAMANRLPCELTSVYTGKTANLALALPRNSQHRHLVNFQIRRFGDNGILQRLGNKYFSEYHRNEICYPSV
HFQGIIPLLTMLAAGFLIACIIFIIERICSPPKKELFDLNRRRRC>DallIR118
MINNNGKNNNSLSYKKGINFSISLILLETFFSAASVLLVKATEESDYHTPLIRDIYDQRGTCGIVLASAENYQSFGTLTIWHGIFKTLSDEGIPTLMISFEQFEDRFEFYAGLTVR
PLAVIIFVTIGDVQSFSEISRDLDMSYAVWLLLFMGDASPDVCEFCHSPSGNLLNLKYDSEVVVSCCKSNIIQEWWSTLTEHTNSLELGRWTAENRGIEWFFNDSLYSRRHSM
EGQEFRIAATSVYFWERNSKYYGFLGEILRELSDSLNFTIPQVNWGTSYGAWNPETSSWTGIIGKLENNEADLAVSEFRITQERLNVVDYTVPIGVGGTRLYLRKLDAARLQ
WNAYFKAFSMDVWMVIIGLILTIPIFLTLMRYKRKHYYLLPLALEHYLSIWGIYCQQGLSEFPKPTSLRIVYLSIFISALVSSGAYSASLISFLAVSSSYSPFSTIEEFVEDGSYQLI
VLKNSPDYYMYKTSNQTLMKKMMSLMKPTNLLPDSYQEGFEQVCTKRVVFYTHEAIRRAMVNLIPCEITAINTGKTETLGMALPRGSEWRGLINYQIRRFGDNGMLQRL
GYKYFTEYNRNELRYPSVHLQGIIPIVAMLGAGFLIASIIFIIETIFCSSKKKSLNPRRRKSF
>DallIR119
MRKLIILLCISAHTGQSYCQLIRPNVIYESVIKGVHDYYNNTCIILLHATEDPIESQEESENLQRLQAYLSKAYIRTAVMQISTFIDRVGGSYYHIKRPLFVLLNDDDDVRNQFAF
EIAPWIDMSYPNWLVFLRPETSIEGFFDKIYVQFDCTMMVSQPDGNLESPGEIITEVYQIDRGEKLRTGLFATWSRETGIKLPRWSLYQRRSDLQGHLFRVMSIEDPPQSMIRR
DDNGQVTGLGGFFGGLMDLLQESMNCTLVYLETNEWGYLRGNGTWTGAVGSLIDNTSDIVAAELIMTRDRVDAIKFTTPVYSTKIRTYIKRPSLSALKWGAYFIPFEPSVW
VAIVVMIVITTATISLVNSAISLFARQWKDNDDCPTNVPDIFFAVFGVFCSQGMSASILDPIRISHFVIHLTGVIILAAYSAALISALAVKTFVLPFTTMDGLLKDNTYRFGVVRD
SADYSFFQNTTDEILGVLFDKLLVKEKELPNNYLEGLSLVCNEDKYAFMTVDNAVTQLQSEVGCVLVPLDTISQTSIAFGLRPGSPYRGILDSHLLLLRDSGVMQRLLNSHW
AMTGDNVEGGWESVEIGDILPLAVMLLTGIFMGFMILSIEKLVKRNAKIIKKEKKIVKKFLKNAKFLSGQLNHVKKSNK
>DallIR120
MKLLPGFSVFSLLRVVGSIASGESILVWDSENADFIPIWHFSLFRDLIMKNRNNSLGVEKARLQGQTLRIGYHSEVNLMTFENNGTKISGLLGDLWTMLSDLLNFTIEAVEVP
EAKFGAQSSESHIGLMGLLRRNEVDIIPRVAFYRNTNEVMDYSTPLWTNSFRVFVRPQFDSDDSWIFKTFPWPCWISIITSIALFSFFGTLFDRLTAARFQRNSLRHLLLEHFFY
TFGTFCNQGDIPANMERSRLMAFSRRTCAWLIISIFSTSLVASMTHKEMHLPFTGIASLLAKSDFKLVVNNASLGFSKFHDLILPNFTSPKYSRRFEFTRIAEDMYRKGCGSSG
KKAIFESEDRYRAWATRTCTFIPTQETYFSTWITTGMTKGFEYKRPIDNGILKIIEVGLLTALKDRWLIPPLTWPPDKYVVVGMKKVYIIFVVLSIGVMVSLMIFTMEHIVVIHR
RYYLRRKWDKQLRKRARRLKVAWSRRNPFEEKGILEDRNAFLL
>DallIR121
MAIKTREFTIIFLFFVILNGNQGQMHPPPSPLTLQLMMEYAKFRFWEQIVLFDDLSNGNNEILYYARPLISCLSDQGMSISIQSTLTNKLPEALNIRRHRVGAIVLLDRLNHTSA
ENVLKTASSKRLFDYYISWLLITTDSNDASIDLILRNLTIGINSDVVVATSSASAYNVRKEIFNYKNRQFREYIKTYNFKWENSNEVIPENVTRNLEYSLMENRSISFYLVHTYK
IRINDNSSLVVDPLGYWNPGAPMLKLPINVALRNNFFRLPMIVGILNGTSDNQNGEITSYEEEPSEDQPMNDFIDFLAHSLNASLEIVPHEKLGTLTNKVWSNLLGDVYTGAV
DIGLGYITTNEDRRRDMSFTHPLIRYTRNIYIRPPESGTMRDIFLQPFNNHLLLCVALMQFFIIVTIGSINYAANNVLSKRKGRQTGIGEATLWCTSIMCMQGSPWNPSTLSGKT
ALLASLIFALVTYNAYAGFITSILSVQATGIKTLDDLLHNNFKLGYSDVDDEYMRNANDSALRQLYIKAFNGRESRLSTSKGLQRAVGGRYGFFASATLARRALRTSLIHERC

LLKEIEIEHTFTTVALPMAQYSPYEKIINLSILKMDERGVIDKIRQRMLPDMPRCQDATTFHSARIADVYSAFIILAIGIITSLFLGVIERLWNQRKMFLAKIIGRVTSRKSVEAPK
NHSVNHNFHGASVTWWRHSVGKNSRLHQGKRFHRAFKLGTFPFHH
>DallIR122
MCKYFIILLHVISSFTSGERGDIILDIDRGLWLNDRDFEMMIKYSYHFSTCCNIFVNGTTRDIGTLFNLFIKIYQYEYTTGSIEYGCRGFFLLGSTGETLALAVGRVPTAVSTTEIL
IVVDADLRDDSPLLNVSLFQHSNVNIIARSGNWTLSENFLQPRMFKKVHRSSQMRHKTGIVDLEGRRLQVTTSNIAPFSYLSTTVNRTVNGIQGQFFVANDERELDGVEVKL
FLIMAEKLNFTWMMRKPNGPYRHGRPNGTSWNGGMIGQLYRKEVDLAFGEVWLEYEKSQYVNLSVPWYEVWINFLVPRPKPTENVWALAKPFRLNVWVAVIAIVILESI
AVWGKARINSKLPPRFRSYVNTLIEVIGRLVGTWAPRKTQGIRIQLQFWHFAGLLVVTAYSSSLAARLTTPDYEPRIDTIAQFVKANLTWGRERTPPNYRHYFDLNDPYAKQL
SNGFLIETSQEDRQSKILEGNYAIIGKISHSIFFPEVNVCNSDLANYRVMRESTAKFFISFGAQSWLVPSIDTMMRRLTETGLVEYHLRDVIRRRVNGSLRDVFIEHDGENTNGP
RALKLKPLGAAFIILLAGYVVATIILYFELKNKNKIDH
>DallIR123
MWKCVVLLGLTGNFVGGDRNVMIDFQRASWLDDQDFKGMIDYSFGGSRCCNIFVEGVTDGVNALFHQFIKLYGHDYTVNKINRKCNAYFLLAEESTSLISAVEKVPTTIAL
TEILIIFNAEIEKNSLLFNASIYENANVNLVSRTGRWSLSEMFLLPRVFKKVSSNSDMKHNKGIVDLEGRELQVATFYVPPLSYLSTSENRTVNGIEGEFFSSNDTMEWDGVEV
KLFMIIAKQLNFTWMIRKPNGGYRYGRAMNSTWHGGMIGQLFRKEVDLAFGGIWFMYDPFRYVNLSVPWYQVSIHFLVPRPHPIINFWALTRPITLEVWIAVAVTIAMQSLN
VWFKAWINPKVPSRFKSFSNTLTELIGRLVGSWAPRKTVGLRVQLQLWHFAGLLIVTAYSSSLAARLTTPDYEQRIDTADQFLKANLTWGREGPIPKFDDYFEEQYREKMRE
RFQSENSPDERQSKIEQGNYAIVGKIIRSIFFPENDIHSSDLHNYRVMKEGFGKYYVCFATQPWLVLSIDR
>DallIr124
MKTPLLRGALKFLVIMMCTGPFEAFHIPEPAWSADLMSYIKENYEHYRQVMIITCKDSGVPFENYWIRKILHTAMATFPTIRINVDFSSNINEEWSFHRTDATATLFIFVDDSD
TWRHHQPKDVGKVVPQQIISIMKDLSLNKKIAKYLALFLSSEQTLNFDDLLRHAWEMQMIDLTIIEVIGCQSVKTTILNDCVDDSSLPVIHHFNPFLDSIIRKTYEPGMKLFPD
IMKNMHGYPLKIGIRHHPPFSSVSWDNNSNYESMSGLDIQLIHTMAESMNFTLQILPQLMTFEEMQDNSSNGLFNFLRSDKIDIFASPHPHYTEDMEEHSFRSEAFIRDQLCA
MVPLRKTVRILLPKTVTETLILTIGIVLIFWASIWLFRFSQSWSIFTIVRVLFGIPVFAHHARLKPAQRVIIQILMIISLLYSAKIYASLTNINIDVVGAVEIETLDDLDQSGLIPKIHP
HLMDKTFGHVNKNDQTLMNLKRKTVSMRSMMKCLSEAEKFKNTTCLMTTVEGYWFIRSTYRHREPALKMTQACFWSDSYAFLFREGSPYRNKIDNTFRLLAEGGIPIIWA
RNDTSGNFEEQRKDNNVFHELQVPPPGVLRDQLTVVLLFGLTIAIITFIGELLWYHRIKRWKSV
>DallIr125
MKTPWQCGSIEHVVIVMCIGKLGAFNPLEPVWLKDLLHYIEHNDDEYHQVIFIASEHTGTSFENYWIQRIQQTIMATYPTIRLNVERPSDADEWSYHGIDATSTLLISVDDAE
VPRLDISKQTISVMKKLSLNKKRAKYLIIFLSSERTQNFSILLRYGWKIQMMDLVILEIVSSRREMITISESSIDDHSSLVIHYFNPFLNLMVRKPYEPGIEWFPNLMSNMHGYP
LKIGIRHQPPFSEVTWDEKANCVSMSGWDITVIQEVAAKMNFTLQILPQLTNFSEIIEDNSSYGLFNLLSSGKVDILASVNPHYTEDMEENLYRSEMIFRDQLCAVIPVKNTTR
ILFPTEIIEAFISTIGILSIFWVSTLLFKFRRSWSVFDIFRMLFGIPKHINHSSLKLAQRLMIKIILIVSLFYCVRIYACLTEIIIDVDHEVEIENFDDLDRSGLIPVVSAYLLNRTFGNV
DENDRALINLKNKAVATTAIWDCPSHADRFKNVTCLMTRNAVQILMKSTYQPGERILKISKACFWSDSYSFLVRSGSPIRKRMDYIINLLSEVGLKIMWFRNDTRIKLWEIDR
EDELIWDEMKYHPTSPLREQLTIVALFGFTTATITFIGELLWYHWLKKWKKTIKLFR

>DallIr126
MELYHFIVVLIITRALTCSGEFNWIEGIEDYASKHENLHQVIFVSESEETLRIPGMSELFRRIAEHRPIIQISANNSDMFNKMRQDTASTLFIYTHTPFGRGILPSTKIIDAMMEAS
FGRTAVRYLIIHYSNSKNDYLSETLKHAWRRQILHCTIIELLYNHKKMMQEEIFQVLVHLYKPFLNQFITANFSSQTELFPNTPNDMNGYPFKIRITHDPPFSTIRRQLDGTTKL
KGANMRLIDTLAKAMNFTVKAEEARVSKNDRLDTFLQPLMNREMDIYAHLYAHPSEHAELRSLRTEPIDVEHLCAIVPYVAKKNSQLPSMGTICGYLLVLLIVVLFWILEHF
THLNSHYWSPWIVIKLLFGVVVVKRPNRCADRIMFGVLCLVSIVYLTKLYSSLTGGIVYTNEVNKWLTLEDLVNSDLIPFISPLYYNKTFSYATGVELKLKEKVKMISSNMMN
CLNHLAEHKNISCIMSKNEYKVFQQYETTRRTELLVPCLIVDSSAFSLSKNSPYHEHIDHLIRIFRDVGLKNKWYDIGFSRRQNITQEESYDARDVLLEKNDFFQQCIIILTIGY
TLATIALLGELIYYHKCEKKHSSVSAFWRKLKLILIVFCFYDCRNGNGDHRLICFLRGEPLHYEGCRIKFC
>DallIr127
MFNRKISLVIFVITILSDDLQGRPEGDWFESVKTRLSNPHQVYSAMIIRDNNTDDFDVLRDELFGKIIESMPSEIFSNLDGVHNSNNWYRNVSSRMASSLLFVYYHRYSGDNT
RVHEVINTMRGLSKLSAKCYFLIVLRTSSVSKEGVEDMLRHSWNNTMLNILICEIRRDENSSVTNWKVPNDESNRVIIHLYNPFLDKFYHKKFFPKLELFPDFASNMYGKKL
NVLIVQQVPLTFVKWDKNNEMTEMSGANIALMQTLAGAMNFTPVILPKWNGTGFSVKTRTFDAWKFFKTTNTDITANLEPHFTEHISEESLRFRPIMAQEVGVLMPVEYAI
DKKYQDNAVESSIITLIIMMIFWFVGILLRLDKKIWNFSIIICLLFSFSVPRQPSKAYERILFLSLSMLGFMYSNQIYASLTNVAVLSLEEREFVTFEEIDASGLIPIVPIAHFERVFR
NAVGAELNLKKKSQIIVNIRDCPKMAMVYRNVCCIMYNTEGEYYRRLSRQDNGQYRLKFAKPILRSDNGAFGVRDTSPYKTKINYLIQRCFEAGLISKWYLEAISPSRSGNI
LNDDKPSRGERSATFRRQLMVVIVFGHSLATLVFFGELLVHRLRISHSNIDKSRLNLRKLWKMKTK
>DallIr128
MFITKTFLVIFFITILSNEFQSRPEIDWFESAYPHLSDSDSPHTALIIHYKGAHDADAIRDELFEKIIQTMPSASIEMNDEGIALKEFDFGNVSKLSTSLLFIFHTEYSGDNVQISRV
MHSLNKLADFSVNCYLLIILRTSSNLNRRLEDILHKAWKKTIMNIAIVEIRGINCNRLTKLSTDNQQFHDCEGKKYSAFSRIYDDSPKRVIIHQFNPFINTFYHKKFSTRRKLFP
NFANNMHGHDLKVRIINQPPFAAVTWNENNEMKKMSGPNIVLMQTITAKLNATPVILPNPKSMEFWLSNFTVEDLINESLNSDLTAHLCVRFTGHILEESVRSRHIITHELGV
LMPKERGINKITLYYAIESTALILVIMLTIWFAAILLKFDKKFFELSRIFRLILSIGVDYQPTRSSQRVLFLVVIMIGFMYSNNIYASLTNLGVDPLVEKEYKTFEDIDNSGLIPIIRA
PVFRKTFNDAVGAKLNLKKKSIQVIDSSNCLRMAMVHRNVCCVLFRAEAELYERLSHRKDSQYQLKLSEPIFWASDAAFRFRQGLPGKKTINEVIDRCNEAGLINKWYWN
MEGSADPNNNTNDDLHAQDNAVQTQLLIQLIVVIAFGHLLATLVFIGELLAHRFKNSSKGKRKVRFCVY
>DallIr129
MFTIKYLVIFFIIILPDELQSRPQVDWFESINTLFTAHKIHTALFIHSNNTDDSDIKDGLFAKIMESIPSVSFEMDHEGIVPKDLDIGVLSKLSTSLLFIHYAKYSGNPVQIYRVMH
SINKLADFSVNCYLLIVLETSSNSSEEIEDILQHAWNKTILNMAIVEIRLAANFNTSTTVIAEISKFSDEANRGLHKNIASRVIIHQFNPFIKKFYHLEFSSGKELFPNYAKNMHG
HGLKVVIVDQPPFASVTWKEDGEVGKMSGPNILLMQTITEKLNATPVILPKPKNMRFWLSNFTVEDLINESLNWDLTAHLCVRFTEHILEESVRSQNIITHELGVLMPRGYAI
NNKIINNILESSALTLVMMLAIWLAAILLKFDRKFWKLSTIFRWIFSISVHHQPSRNFERIFFFVVVMLGFIYANNIYASLTNLGVDPLVENDYETLEAIDDTGLTPMIRNPLFRR
TFKNATGVELNLKRRSIRVMEITDCTQIAILHRNVCCVLFKSEAEYHKQWSRTKNGENQLKFAAPIFWSHHAAFLFRQSLPGRKNINKVIKRCSEAGLLSKWYTHMTRGFG
GIAEDVAHVTDDLHTLRGQLIVVAALGHSLAALVLIGELLTHRFRNSFKKKCRRTKKSH
>DallIr130F

MPIAKILLLIFCITILLDKTLSRPEVDWFESVSTRLLRSGDIHTAVIVHDLDTDDSDVLKKELFGKIMGNMPCVTVNVDDDILHLKKLDFGVVSKSSTSLLFIFYADYSGDNVQI
FKVMHAMNRVADFSVNYILIILRTSSNLDRGVENILQHAWRQSMLNMVIAENLNSRIDEDSSIQVIMHQFNPFIKKFYHDEFHPGIELYRNFAKNMHGHQIRVTFSDQPPFTY
WKAPDLKMGIRKRNNNAMTGPNVHVIENIAQALNFTIAYAIFIEDKYEWESRPNNYVNSINTVLYLLKKSATDIFANAMVLTTDETLDESLRSPLIMTNELTLLMPSDFKFNK
KLMNNAILSGVLTLAIIILIWFATIFLKFNEKMWKFSVIFGLIFSITITRQPLKTFERILFIVVVILAFMYSSDIYASFTTLGIDPLVENKYETFERIDDSDLTPIIQYLLFNKTFEGAA
GAKLNLKLIKXXKKTVPYFSTRFCPEMAMVHRNVCCIMTRIEAEYYKSQSHTNDGQYRLQVAKPSFWSDTGVFHFRWNLPGKTNMKKIIENFREAGILNKFYATPYMHPQ
NDQNDNAYEMDEQSQIHLLRQLVMLAVIGNSLAVIVCIGELLAHRFKNSVKGKVKLNLCGVFRRRMKQIQTYLLLTYRTLNVQVVLLLRAMRKYKIQLLDRRRWPKWLK
SFSRTADRQAQI
>DallIr131PSE
MSITNFLVVIFFITILTDEIQSRPEIDWFDSVATRLLHSRDIHTAVIIHNRNNSEVSDAVRDELFGKIMGTVPSVIAEVKDGLIELEEMDFGVVTNEFTSLLFIFYADYSGNNEEIY
DVMYAINDLTDFAVNYMLIILRTSSNLTRDIESILQYAWNESMSNILIVENRLVTNSKMFAKESADNSQFLGCGNLQCYGVNRKIVEDNSVQIIMHQFNFFTKIFYHEKFQPGM
ELFPNFAENMHGHKIRVKIWKREPHLIQAMARILNFTISTVVGVKNHLSFKARATVYKVS*LDIFTFGMLTGRASQLSATKCIVMKNLVLVMPNEYVFNTNLLSNTFASVIFIF
TVIIVIWFVTIVLKFQEKTWEVFKIFSLIFSIGISCKPSKSHERILFIFIFILGFIYSNDIYASFTNFGIDPLVEKQYKTFEQIDDAGLPIIVDHKLFIEEFYAGTVEAKLNLKKRIIFKDI
PCVKMAAMHGNVSCLILQNSAKYVEYKDRETSGQTRLKIAKPIFVSLPEVFLFRYGLPGKKKMNDIIERCRETGLLFRWYTSKYLKTSSQSDKTVIANQMEKETKTQFLRQ
LIVLSLLGQLLAIIVFLGELLAHRFRNSPKGEIKLDLRGIFKRKIIRREWYAINYCLNLTR
>DallIr132PSE
MSITNFLVVIFFITILTDEIQSRPEFDWFDSVATRLLHSSDIHTAVIIHNRNNPEVSDAVRDELFGKIMGTVPSVIAEVEDGFIELEEMNFGVVTKEFTSLLFIFYADYSGNNEEIY
DVMYAMNGLADFAMNYVLIILRTSSNLTRDIESILQYAWNESMSNILIVENRLVTNSKMFAKESADNSQFLGCGNLQCNRVNSKIVEDSSVQIIMHQFNFFTKIFYHEKFQPG
MELFPNFAENMHGHKIRVKILKREPHLIQAMARTLNFTISTGVGIQNNFSFAARGTDYNLS*LEIFTFALLAGGGAPQLSASTYIVTKNLVLVMPNEYEFNVNLLSNTFASGIFI
FTVIIVIWFVTIVLNFQEKTWEVFKIFSLIFSIGIPCKPSKSHERILFIVIFILGFMYSNDIYASFTNFGIDPLVEKQYKTFEQIDDAGLPIIVSEILFIKEFYAGAVEAKLNLKKRFIFK
DMRCVKTAAVYGNVSCLMLQDRAEVTEYDDRKKSGQHRLKIAKPIFVSLPEVFLFRPGLPGRKKMNDIIERCRETGLLFRWYTSQYLKTSSQSDKTVIANQMEKETKTQFL
RQLIVLSLLGQLLAIIVFLGELLAHRFRNSPKGKIKLDLRGIFKRKIIRREWYAINYCLNLTR
>DallIr133
MSITKTSLMIVLIAILADEIQSRPEVDWFDSVTTRLFRSGEVHTALIIHQRSTDYSDAVKNELFGKIMGTMPSVIVEVDGGVINLKEQDFGVVSVKSTSLLFIFYADYSGDNVQI
YETMHAMKNLTDFSVNYILIVLRTSSNLTRSVETILQYAWDLSMSKILIVEIRRLNSKKRNEITADNAKSLGYGKLKCYRVNGKVNEASSVKLIMHQFNFFSKQFFHEEFHDG
VKLFPNFAKNMHGYVMKSIPNHNSRLRYGQHKNEYQMFPLLFLRTELYKDMSRVLNFTTKELLLPSKWNRNSVDALIDFDNKVGKIDIMGPYSAASLPSTKMKIFFLEEME
DRDLVMVMPTTYTINQNLINNAVASTVISFVIFALIWLVAIILKFDNKTWSALAIFSTLLSITVPNQPLKTNQRILFFTVVMVAFMFSNDIYGFLTNLGIDLFVENEYETFEQIDN
LCSTPMVNKYNFYPAFKTADGAKFNLKNKSVNESIVSCLKQAEQYRHVCCLMDRGIFEVLTFWYGRTIARYQLKIAKPVFAVQTECMWFRHNIPGTNEMIEVINRLRQAGF
LRSRYSDPDSHSRLRALLQRTDAMDKRRTEESRFQLLRELIFLCALGYFFAVGVFIGELLVHRFGNSAKRKMKLDLPSVPDRKIERREW
>DallIr134F

MFVTEIFRVICFITILLDELQSCPEVAWFDSVSRQLTDTYHIHTTLVVDFSNTSSFDFMKDELFGKIITSLPSITFSMDDQGIEKNGLHFGDVSKVSNSLLFIIYAEYPGNNVQMY
KLMDSINESADFSVNCYSLIILRTSSNMHRDVENILQYGWSKSMLNIVIVEIRRETDSRSTTKVSADNPKFLRYQNFNRKEDHSKICGDNASQLIVHQFNPFINEFYHKKFSPR
LKLFXAKNWHGYPLNVAVINRPPFAFIKWDKNNQMKEMSGPNIVLMQTLAKNLNFTPVIQSRLNTVEFWESDFTSPDFIRLFEIHGVDLVANLAIHYTNDTMEESVRSRTIV
TQKLGVLMPKRYYISKNHLHNAFKTTALMIITIVLLIAMFILRPDKKIWGVSCIIGLLFPIGVYHRPSKALERVLFIFVIILGFMYMNDIYASLSKFGVDCLVEHEYSSFEDIDDS
NLTLRIQAPLFKTAFRNAVGAELNLKKKTTKFADNIDCPQIAMVHSNVCCVMYETEVHFYKQWSHAKYGHNGFTSAKEFFWSGALAFTFRQGLPGKEYINKFIGRXPHEA
GLLSKWYPDSRSSMQDEGVRNEKSQTHLIEHLIMITIFGHSFAVIIFMGELLTYCLAKKIQEKKRAQCMRCI
>DallIr135
MCIIKTLLVIFLITILVDEIQSRPEVDWFDSVATRLFRSGEVHTVLIIHQRIADYSDVVKNELFGKIMGTMPSVIVEVDGGVINLKEQDFGVVSKNTSSLLFIFYADYSGDNVQIY
EVMHAMKKLTDFSVNYILIVLRTSSNLTRPVENILQYAWDISMSNILIVEIRRLNSEKRTVASADNTKVLGCGKLKCYGANGKFNEANSVKLIMHQFNFFSKQFFHEEFHNG
VKLFPNFAKNMHGYVMKSLGNYIWPLRYGQHKDKKKSFFVRKKFELYEDITRVLNFTTTQQIFLRGEWKGWRGNYTYGALLSYYNKTEKVDIMGPLSSMLLSDLHIKPFI
LEAMKDQELIMVMPTTYAINQALINKALASTVISLIIFALIWLVAIILNFDGKIWSAFAIFSALSLMVVHHQPLRTNQRMLFLAIVVVSFMYSNDIYASLINLGIDPFVENEYETF
EQIDDSCSTPMMQWYDVNDAFVGADGAKLNLKKKHVKGTLVSCLEKAVEYRNVCCLTDARTMYASRFWLRRNNGLHRLKVAKPIFASRTECIWFREGIPGKREIIEVIKRL
QETGLSSGWYSPADNPLSLQTDNHNRIGEMDEESRTQLLRELILLGVLGYSLAITIFIGELLVHRFGSSAKGKMKLHLPGVPDRKIERREW
>DallIr136PF
MSITKTSLVIFLITILADEIQSRPEVDWFHSVTTRLLRSDEVHTALIIHQRSTDYSDAVKNELFGKIMETMPNVIVEIDGGVINLKEQNFGVVSKNTSSLLFIFYADYSGDNVQIY
ETMNAINKLTDFSVNYILIVLRTSDLTRSVENILQHAWDI*MSNTLIVEIRFSKVSTDNSHFLGCGKLKCYGANGRINEDSSVKLIMHQFNFFTKQCFHEEFHHGVKLFPNFAK
NMHGHVMKTFRGYNRPLRYGEYEDKKNSSLVRKAFALIDDLTRVLNFTTREQLILRGEWKGCRGNYTYEALTDYYRTINKVDIVGPVSTTSVSGSIIQPFLLRPMKERELIMI
IPITYKINQNLINKTVASTVILLILYXLIWLVAIISNFDEKICNTFAIFSALFSITVHHQPLITNQRLLFFAIVIVGFMYFSDIYASLTNLGIDPFVENKYETFEQIDDSCSTVITSWYD
VDPAFEGADGVKLNLKKKSVNGPLMPCLDMALEYRNVCCLTDRQRIDESRFLLRKNNNLHLLKIAKPIFGSRIESIWFWEGTPGKKETIEVIKRHREAGLSSGLYSTDDDPS
WLRTNKHNNADEMDEESRTQLRRELISLCALGYSLAIVVLIVEIFVYRYRNSAKGRMKLNVPIVFKKKIKRREWYAISRRFNSVELIFINHFYFPSFQLEKSKPSYYSHLRA
>DallIr137PSE
MTHIFAIINSFEWMNHWKQVHRSYLSAPTRIALLITGSCTDSHVKSIKSILHGFWQREMLNVIAITPTQDLPLIHTYNPFLVGTENPPGELMNLTSEFFPDKLKNLHKYPVRIA
FYENPPYVYPKTHPAPMDGGDYALMTFLSERLNFTMKSSMKQVTFADTFFQLSNRKRTAAIGDLLMRDTDILGNSFYLESINGQELDILYPRWRTALVVIVPRTRPIRGILNM
FSSVDLTVVILFFIVMILLVVYLRVEKDTHIFIHVWRLLIYQHFDFIGTRASERLFCISFVIWSFFLVEFYEGRLVNDLSSSFYKDIKTLRGLIDSKLTVLVPSWQQTVLSHSSIQE
RMELVGQLEVEGNFTKCAQRAVEAGDVGCTMDANAADYFKDHIAKNAYEETNDGTVKGQLRVMEESLMSFWRLHATRKRFFLKDKLNEVIGIIQEGGLLKKWESDEKD
RQFREHVEEQKESVGMDKMGAAFQLLMYGHCLATGQLLIELAWHAFKKNHLRGWDDSEEAFVG
>DallIr138
MEKRLRILTQSIINQELSYLMNTPFEKEQWLSFKKLFPLPFHNFQVDHAILNFITMGYTSLRPVFLVQEDGFVHPYIDSICPKLRHVVGIFESIDTVHPINWKMLYGSTCLSRTK
IVLWITRHADDDDIKSILKAFWSLDFLNVILVTMSDDSTNHGLAVHSYDPFWVNKGGVRGKVYTLAVADLLYPNKMKNLQGYPIAVSMYDTFLSHPVTNISQLRANSDVEL

LLSLSSWLNFTIDLRSRPISTAEYYRMKSPNGTVVGIIYDLITNYSEVLGNSHIWGKASDRVEFLLPHARFISEAVVPMPEVIPEIICIFKSFDVMTFILLILFSFISVIYMYSKGTN
APVLNTFRLIIHHQFYQVGTRVSEKVLAISWIVFAGLFLLIQQSHLIENMTNPLFEKRIDTLKQLADSDLTVMTDAEHFPLLNRSRDEVMQKIVSRITTKHSSRYCYERLIAGG
NVACFLNTVIISSVRSKPEITQSYLKIPKMHKMREIVGQYWRAIIVKKGFPYLSKFNAGIGRLMQTGFVTKWQARDRERLKPRVIHADDAVPLNLSHFRGPFIIFLFGIVVATC
AFIYEIVIYRVKY
>DallIr139
MLGFGDDSGKNASFNFKPKIIILEGDSPEQLESDLSSFKETQWWNYMAHYFILGINQNQCENIVAILTTAWKMNIIKSIYICLNTENMPELYTLNPITNYALTPWERIEIPLVKG
AIYRQLFDANVSCQNLHYDKTKVLNGFKITGVIAKFSNKKAKDMNVVNQMALNFKKILNVSIALKLKPPGDLVIQTIADGTADMVLNYGFMYWNPERQFLFPYLWTQVM
AVRKFNGYLSTLEKIDNLWSVPIRVLALLIFSIFFLVMVYENRDISSAMLELIRLLTYVSIKTTREKLSFRIFFSMMMMFMVISNGVFQGAISSFLTKPQHAKGVDTVDELRDL
NYTLYTIPGTVERAKEECPDNEVIEVPLEVCPELLLKSSRAVCVTFREFFYEFYGNSSLTLMKQPLKSTYFNHKCRDDWPLHQRVNDYFVQTFEAGLINYWLTEKIRLTMQK
HRANEMGLLSPNYKPVQLKALDFVFKLLAIGLCLATVVFLLEVLAKECKIDLWLLR
>DallIr140
MVIVGDFDGRILMSLIESIPIPIVTIDSHSEFIFGDASDGLYVFQPDIIIMAMEDSPKQLGNDLQLLQEMNQWNHMAAHFILEISARFCKDALESLLITWRMHIPQSSYVCLNAE
QAIVLYTLNPITNYAPKPWQAITSADRFNSSDRWTLYSQLLNPNDIACDSLTFDRMKHLNGYEIKALTAPKNNVSFIPGKNYKQAGYKMLEFFGKTVFDTIITRLNATLVLQID
DIHIIPQYLANQSMDIDIRFTLSFSKTNASYLYPYFQPEVIAITRIKDYLSTFEKIAQLWSRSVLVLSFLTILITFGVMAVYKRQGFSLALLEIIRLLSYASIQNNFQSTAMRIFFSKI
LIFIVITNGVFQGRLAAFLTKPEEGYSPENSEDLKNLNYTLYGIQQNVKYLRTMFPDNRVVLVTQKDCVEMALASLSAACVGTKAKYLNKYWMKPIHTTREPLFVDYWNH
QCRKDWPLKHRVEAVLTQIMESGLLARWAFHSISTVLDKKRAEDVERAATKYRPVELKALDFSFALLAFGLVLATIVLIIEFLMKRESNTKIREEIKFRNFFIARRTTI
>DallIr141
MECWLALIMAMMLPCVRTIVIPEEENLRIEDPSNRELINPETDVAKSELNELSKTVVCLMKFVELYMNQLPSRISVLLMETDDPDFSGVYLSKLQQVRSTYILNGYLDSDSD
QNTSLDALLILKSYENLENSTSRLIDLCGRDCRYAVVVTNLFPDEASFMEEAVNYVKLLWIKRVANVVILGPVGNTLLAAQSQGFLANKLTEPSDPIPIGKCHQGEWITTTEV
FPVLKMNNSHIHFAIIDHEPYMSVTWEGDHMKARGIELKIINILRDTLQFRSTGSLLEWDEGNTVEDEIIEEFKSDRKIDLVVGGLLRTMVKDVDFALPYDVTQVVWLVPSHS
NISLLGLISPFTLKIWILTIASIIFGGMIKSLLFDKMSFLEIMALVIGVAWHKQPKRLSYRIKFMSWVLFGYVLTQVYLASLAGQLLAHGDLQINTMQELVDSGLIFGGTANHKQ
FFMQTDDDNDGEISAVDTIYKQFIVFSHEDYMKKLTQLMQGENTSLALVAVLNISSASTSVHHEMYHIVKETLATTPLAFPAWRGLPYLTQIDSKLAALIQGGIISYIANNETT
VQHIHDAIEEKTDANLELVDIAPSFLLLVMGHGAGMLCLLGEFAVFRWQNRKPKKVVKGTKVRRLKGVAKRTVRFDEKNLRLTKPNNGVRLVLRPNVTIGYRDGRLPWKI
V>CcunIR25a
ILLWLLVVGEGGPVTAQYNDVINRPVNVFVINDAGNDVANKSVTNSLRTLKDKNPDKLGQVYVVQVNISDSKETLNAICDLWKSAITDNQDNIPDFVLDTTTYGIGAETVN
KFTAFLGIPTLSAQYGQEGDMLGWREISEDQKQYLVQVMNPADLMPEVVREQCSHFNISNAAILFDESFVMDHKYKSLLLNVPTRHVIVPTKPAGTALREQISRLRDLDIVN
FFVLGTEATISAALQAASNLGFTDHRYGWFGINLNEDFTIQCPDCKRARILVFKPEMSTSQQQLNELTSKGALPKPWIQSAFYYDLTKIGILAMKAAIDAGEWKLDRRRFML
CDDYNENVTTPLRNLDFRKRLKLVTGGGSVFMPTYAGFGWGNKNGESQAKFEANGVAIQIRDSRVTSEEIVESWQAGVDVPLTIKNPGIAANQTAVTSYRVVTVIKPPFIMH
DNKTGEWSGYCIDLLNKIREHVKFEYTIEEVEDKEYGNMDEQGNWNGMVKVLKDKKADIALGALAVMAERENV

>CcunIR75q2
MRLFPGIWLWLISMRTSGFIVDGCESDQAKELVELLIELKTRGLSTFSRVTMFGCSDDKSQVARRLSRALVANSMHELDSNQFSWNLQKSVASTPDHHLLIVLEVDCPSSERL
LLEAKNATMFVSPYKWFLISRGCSSVDCVLSKLSELRRNVLPDSELLIWLKQSQRLVSVYKVSAEQGRDWQIEERARSIACDNATFNLLLDDDSAMITSRRRSDLRRTHLKS
SLVITDYDSLQHLTDYRNREIDTSSKCNYPWAKFLASMLNATISFKLAPTWGYPRANGTWDGMTGMMQRGEIEFGASHSFVTGERLKVVHYLAGLTTPSHSRFVFRRPALS
SVANLFALPFRGSVWIGVLALALLFGFLLYPAMVLEWRRHRDGTIEPRVGDETIVVVGALAQQGFWYEARAASTRVLVLAGLLGFLSLHAAYAANIVALLQSTSSSIKSLKD
LLASPLDLGVHDTVFNRYYFKSLGSLEPKIRGAISEKSEWLGLEEGVRRLRRGHFALHAVLGWAYKVVHETFEEDEKCDFEEIDFLNVFEPHMVVAKFSPYKELLRVKALRI
RETGMRTREINRLYSTRPRCDAAASSRRFISVGYAECKGAFYALGYGLVVALLLFAAEIAHRRLRLGIRSNRLKR
>CcunIR93a
MMLSLLLPFLLRLGWTNGYNDFPSLMTANATMAVIIEKGFFKGQDEYRNALTDITDIVIGIIKKNMKQSGIDIFVFGDTNINLGRDYTILLSVATCQTTWNLFKRAQKEKLVH
LAITGPDCPRLPESDGISLPFIDPGEELAQIFLDLRMSSALAWAKVNFLHDDTFDRDTISRVVKALSVELPNKKLLLSTRAIFSVQYDKSDSVMKQRIHNMLADFHVDQLGSC
FMVIITIDMVSSMMEVAKSLKMVHPRSQWLYVISDSASRDTNVTMFLDLLTEGENVAFIYNTTSLDLECKLGLTCHIKEFVRALAKSLENSLKTELELYDSVTEEEFEVVRLS
KAERKDEIIKSINRELSELRAGTTNTCGECISWKIASAITWGASFSINNEAKQQEQKQKQEHHKNKTELIKKSVGELVDSGTWSPAPGIKMNEMLFPHIQHGFRGKSLPVSTF
HNPPWQILSFSNTDKPEFRGLVFDILNYLSLKLNFTYMVQLPSGYDMLSKFQPNNTTVKGKKINKFDVNEAAISVARKVPAEVIELVGDGRVFLAAVATTVSENTKNVNFTY
MIAQQAYALLSAKPKPLSRALLFMAPFTSETWACLSSALLLIGPFLYVMVKLSPKPIELNEVVGLSTTWQCSWYVYGALLQQGGMSLPKADSARLVVGTWWIVVMVVVAT
YSGNLIAFLTFPRMDDPIDTVDNLIARQNQFTWAYPNGSAFENYLIAAAQDTEKYKILLDGASQEDPSEPKRVLAKVKDSNHVLIDWRTSEAFLMRLDLMDTGVCNFHVGT
EDFLHENMRLIIADNSPYLELVNAAIIRMHESGLINKWSLDILPLKDKCFVTKGNQEVTNHKVDMGDMQGIFFVLAIGFT
>CcunIR8a
MSRARRINVQLVFLLSFGLALVASQAPVTLLLVIEQPDAEIMGNLNDVVSEAESQFGANLIKIDVKLVQVDREFVDENYDRVCAHLYNGITMILDMTWTGWDKLRDLARDF
NIIYKRADTTISSYVQAVDQTMMYKNSTDAALIFENEKELNQTLYYLIGNSIIRLVVIESLTAREVDRISNMRPLPSYYVIYARTKQMEELFKTALEGGLVKRDMVWYLAFTD
NNHADFSYFRDSNNPNVSVNVFTMKEEVCCHLMYTVAPCNCPADFVIFHHYFRRLVQLIVETMSECQAANRLQEPQSGQCQNKNATENSVNATLSEFDKRLLAKIEKNDT
FEYVTARTLVTYKAAADLKLLKKGQLETIGTWSRDTGILPLPNKTIQAARRYFRVGTVDAIPWTYKQLDPDTNEPIKRPDGTYVYEGYCIDLIDRLAEMMDFDFDLVIPQDG
EFGQKVNGFWNGLVGDLSKGQTDIAVAALTMTSEREEVIDFVAPYFEQSGILIVMRKPVRKASLFKFMTVLRLEVWLSIVGALTLTGVMIWVLDKYSPYSARNNKHMYPYP
CREFTLKESFWFALTSFTPQGGGEAPKALSSRTLVAAYWLFVVLMLATFTANLAAFLTVERMQSPVQSLEQLARQSRINYSVLENSTIHQYFKNMKMAEERLYQVWKEITL
NSTSDQVEFRVWDYPIKEQYGHILQAIAQVGPVKTIEEGFQKVEASENAEFAFIHDSSEIKYKVTQDCNLTEVGEVFAEQPYAIAVQQGSHLQEEISRKILDLQKDRYFEQLSS
KYWNQSLKGSCSNADDNEGITLESLGGVFIATLFGLALAMITLAGEVIYYRRRNTRQDDSLQQSKNGTNNSGSISGSIQDVKQTKELDIQKLAARLQLKPAPPVAFEQKPSSL
NTTKPRVSHISVYPRPFPFKD
>CcunIR75q.2
MHRYIRQQCVYVSNNINMKRCTNSLWKCLLFSLIRLEILRVGQVSCRHIDESISNLVVEVSTVSFFPSAAISSLRCRHSDDNVIFSRILSENRMLHNHLSFDDFQKDDFPLHKIV
IFLDFKCHGAREFLLKANSSEMFSAPYKWIIFQDLEHSSPDNCTDGCAFKDFYSYAMYPDSSVVILQKLSKERVQIVSIYRPSPVRDMIVENLGYWSSTNGTKWHNLNIASQ

RRKNLQKTPLKSSIVVTNPDTLNHLTDYHDKHVDTITKCNFVWLHQLIDAMNATVTYSIVNTWGYRDKNGSWTGMTGQLSRKEIDIGGTSMFIIGDRWNDVHFIPLSTPTR
QAFIFRQPPLSFVSNLFTLPFRPSVWIAIGILLMIIFAMLLLATKWEWRKVYADREFSENEPKPNLSDQLLLILGVCAQQGFGRSPYTVPSRIVLLMLLLAVLNLYASYSANIVA
LLQSTTTSITSLKDLLESPIKCGANDIVYNRHYFKLEKDPVKRAIIDKKIEPKGSKANWMTADEGISRVRQGFFAFLIETGPGYRILQETFEEDEKCGFREMYFIDHFDPMFAIV
KRSPYKELIRVNSLKIWESGLKSKEMSRLYTKRPPCNGRNKFVSVGLNECYFAFYIIGYGVLFAILAFLVEILSKKSGSLRKRQPVESTARTSFAQRNLQQNSARESPFSAS
>CcunIR76b
FPSHITVTTYSDMPYSRYRKLDNGTFVGEGFAFELLALLMKKFKFTYTIIPPAKDIIGDESSGMIQQLYN
>CcunIR75p
SENEPKPNLSDQLLLILGVCAQQGFGRSPYTVPSRIVLLMLLLAVLNLYASYSANIVALLQSTTTSITSLKDLLESPIKCGANDIVYNRHYFKV
>MpulIR8a
MLATFTANLAAFLTVERMQAQVQSLEQLARQSRINYTVVANSSTHQYFENMNKAEEKLYQVWKEITLNSTSDEVEYRVWDYPIKEQYGRILLIISTVGPVQNVSEGFSKVIE
SKNAEFAFIHDSSEIKYEITRNCNLTQIGEVFAEQPYAVAVQQGSHLQENISRTILELQKDRFFESLSGKYWNKSANGKCSVTDETEGISIESLGGVFIATLFGLALAMITLAAEV
VYYRRRNAAQDETKRQETNPDRVRSGKNTFERILSKSKLHQLKQSSNTAFTTRNRGLRPRVSHISVYPRVFPFKE>MpulIR75u
MTDPDTLNHLTDYKNKLVDAVTKANYIWLQHIAERMNVTVNFTWRNTWGYRDKNGTWSGMIGLLDRGEIDFGGTGTFLVGERIGVVDYVQLYTPVGSRFLFRRPPLSYT
SNLFTLPFDRIVWIAIGVLLILVYVLSYISMKWEWNKIEKKQIDSHLPGELESDPSLSDNFIVVLGAVSQQGFTYEPYTISSRIVVFVLLIAALSLYASYTANIVALLQSTSSSINTI
QDLAESGLKIGVHDIVYNRYYFGSFKDPLRKDFYERFVENKSDVWMPLNEGIQQLRMGLFAFHGDSSCGYEIIQQTFEEHEKCGIHEIDYLKVLDPMLVIKRNSPYREIFRVG
AQWVRETGLQKRDAPKLFTEKPICTSHASFVSVGTTECYAAFLTMGYGMTISFGLFLLEIIWNKSFGHDKRTDIETNDTPPEGNVASAELESID
>MpulIR64a
MEISSFIPLLSILFSSTFTRGNLLSNLAEDYFQNIYIEQVVVFGCWDEHERVEFVRSMMQSDFRMTYVNIQPGLSMEKILKVNYYKLGIILDLDCQLNHIIFDQFWEQRLRHNE
SYFWLMPMSQSETIPNYFEQLPLNIATEMTLAILNNNNASYTLYDIYNPSYRHGGKLNVTYMGRWGLKDGLKIELTQYKYKRRGNLHGLVLNASIVIDHPPVPDYITYINNPI
KPHLDTMHRYNYALTRQLRDYYNFTMNLSRGTTWGYLVNGTFNGILGDMVKGIIDFGATPFQYKPERLDVCEYTVQTWLARPCFIFRHPSKNNVSNPFLKPFEMNVWFWI
AIFGVLNWSLLYMTAKIERVLDWHPPVNTLDTHPASECALIASAAICQQGLSDGPRIYSGRIVFMSLFLWGLLLYQFYSASVVGSLLAKKPRWINTLQDLVDSNLEIGIEDIAY
NYDFFATTLDPVAQQLYREKVAVNKKRKKLPYFTVEEGILRMKKGGFAFHVDVASAYKIIEETFTSAEICDLVEIQLFPPKHTATATSRFSPFKKMVTYGMRQVVEHGMAKRL
RDIWMHRRPECPESHNEDPVPVVLEEFSPALFLLSCGIFCAFTAMLGEMYIIRRNNSKVNLANDQPENVNDGNPFGKIISSATSTRSKDPENHIP
>MpulIR76b
MKLLHEKEVDMAVAFIPILPELLPYCSYGPILGFTDISVILKRPRVSAVGSGLLAPFSTPVWICILISLIIYGPVIFYFTSYRSRLFGGIKQDTFTLYGSIWFSYGALLKQGTAYAPI
NNSTRLVFATWFLFITIITSFYTANLTAFLTLSEFTLRIKSVEDIVNTGTYWTAQKQHIVDYALKAGHAGTLEELKYSLKYDYGQFVDVPWETKAQEIEKIARTDQVFITEMNLI
DTIIHKDYMMRTHMNIIEKRRCTLVMVPQQLYRQNRAFAYQPKSPVQEKIDQQLLHLIETGIIMHMESIPLPIVRYCPLDLQSKERQLSNDDLSLTYKVIATGYAIAIFVYVIEM
IQRWTNYGFCSCFEEACPCCQNRTIIVPQERPRIPTAPAAPFTIAIPKDPVRKSSNNPDIARKEVFNGRDYWMITQQDGFKRLVPIRSPSAMLFQYTS
>MpulIR25a.1

MAPFMMKDPETGEWSGYCIDLINEIRAILNFEYEIHEAPDGQFGAMAPDGTWNGMIKELQSKRADIGLGSLSVMAERENVVDFTVPYYDLVGITILMQKPKIPTSLFKFLTV
LENEVWLCILGSYFFTSLLMWLFDRWSPYSYQNNKEKYIDSEEKRIFDLKECLWFCITSITPQGGGEAPKNLSGRLVAATWWLFGFIIIASYTANLAAFLTVSRLDTPVESLED
LSKQYKIQYAPNFPSEAYTYFERMAAIEERFYEIWKDMSLNDSLSDIERAKLAVWDYPVSDRYTKMFQAMKESHFPSSPEEALKRVRGELANYSASEFAFIGDATIIKYLEMT
NCDLMVVGDEFSRKPYAIAVQQGSPLKDQLNNALLQLLNKRKLEHLKNKWWNNNPLKRVCKKEDDQSDGISIQNIGGVFIVILVGIILACGTLAFEYYWYRYRPIAMAQQT
RQTEKFASREVKPIRFQLKPAKGAFNNAEFRSRF
>MpulIR1
MALLFAVIVILVTTAMSLPPVIRIGAIFTEDQKDSPSELAFKYAVYRINKDQSLLPNTTLVYDIQYVPKDDSFRTSKKACKQLSRSVQGLFGPSDPLLGAHIQSICEALDVPHLE
ARMDFESTFKEFSINLYPAQDHLNKAFKDLMSFLNWTRVGIIYEENYGLFKLQDLVKSSPSSRSEMYIRQASSGSYREVLREIRQKEIYKLIVDTDPANMQQFFRAILQLQMN
DHRYHYMFTTFDIETFDLEDFKYNNVNMTAFRLVDLDEPKVAEVLRQMERFIPIGPAILNRTGVIQAEPALVYDSVQVFAHGLASLDRSHVLRPVNLSCEKEEPWDDGLSLY
NYINTAELHGLTGHIEFNEGRRNNFKLDLLKLKKEALVKVGEWRPGHGINVTDVDAFYETTSTNITLVVMTREEQPYVMVKNDKNLTGNARFEGFCIDLLKWIAQQVGFQ
YAIRLVPDHMYGVYDPETKEWNGIVRELMEKRADLAVASMTINYARESVIDFTKPFMNLGIGILFKVPSSQPTRLFSFMNPLAVEIWLYVLAAYMLVSFTLFVMARFSPYEW
NNPHPCLAESDVVENQFSVSNSFWFITGTFLRQGSGLNPKATSTRIVGGIWWFFTLIIISSYTANLAAFLTVERMITPIENAADLAEQTEISYGTLEGGSTMTFFRDSKIGIYQK
MWRFMEAKRSAVFVSSYEDGIKRVLEGDYAFLMESTMLDYAVQRDCNLTQIGGLLDSKGYGIATPKGSPWRDKISLAILELQEKGVIQILYDKWWKNTEIGRA
>MpulIR2
MMKIVTISTGLILITLISIGYSLPRPINIGAIFHDGDEINHDAFMRALAILKAEEIAPSFELVPVVRWIHSSTDSFMTSLRVCELLDAGVAAIFGPTSPHTQDIVTSITSQFEIPHIHY
AFRRTDEKPLPHTTVNVYPDSEQISQAISDLLDAMNWIQYTIIYETDNGLSRLQKALMKHGPHNYPITIRQLKPTFDRHHQPDYRPLLKEIANSTVYNVIIDIEPENLLTILKQA
QEVKLLRDYYNYIITCLHIPSPVMLAAVNGSEANITAIQLVTDEAYDLISVESALIFDSVFLLNDALEALEARNGLNGDQLQIKPPQLSCTGDEKYEAGHNITSLMRQVASVG
RSTGLMKFNDNGGRIFHLTFQEFLSGKAFESGSWEEGELKVTRTQKDREASLIQNIESRRFKVTTKPGDPWAIEVTDGSTRGIQVGNKRYEGYAFDLIQVMSEVLKFKYDFE
VVDASYGNYNPETKQWDGLIGGLLRRESDMAVCDLTITKVRESIVDFTTPFMNLGISILFAKPENEVPELFTFLSPFSADVWIYMATAYLAVSVMLFVQARMAPGEWHNPHP
CASEPTELENNFNLKNSLWLTVGSLMQQGSDILPAAPSIRMVASMWWFFVLIMVSSYTANLAAFLTEKKIDR
>MpulIR3
MTSIKTETALMYDAVHLFAKALHVLDESQQLDIKPLSCESSDTWPHGYSLINYMKIVEMKGLTGLIKFDHQGFRSDFMLEIIELNSKEGLKKIGTWNSTEGVNFTRTFGDVY
TQIVESLQNKTFIVTTILSAPYCMLKESSDILRGNARYEGYSVDLIHEISRILGFNYTFNIVPDKKYGSYNKEKKEWDGMIKELLEQRADLAIADLTITYEREQAVDFTMPFMN
LGISILYRKPIKKPPNLFSFLSPLSLDVWIYMATAYLGVSVLLFILARFTPYEWYITRPCGKNCDHVDNRFKLINCLWFTFGSLMRQGSDILPKFSPYEWENPHPCNGQSDVCE
NEFTLLNSLWFTIGSLMQQGSDIAPKAVSTRMVAGMWWF
>MpulIR4
MEIAWYFIIVIVVVLSQHVNAQVKIKMVVIIENGETQHLGPFNDVLLDAERAAGGNEIINIEVKPVQLDRDNIVESFEAVCKELFDGITIILDMTYTGWDKAKNVASTYSIIYL
RSMSSIIPFVQCNDDLLQEKKATDVALIFESEKDLNQSLYYLIANSITRLVVIDELSEVTVTKIRAMRPTPSYYALFASTAKMEGYLNTALQRGLVARNDTWHLIFTDMNYKHF
RYINGDDDLHNPIVILSMNPNLCCKLLSQTSCNCPHDIQIFNKFIERLIYLLVATLRDIYKAGIDLEPKTGQCIVENELTSTGQSATIKMFNETLYSNIAMDELFDYNSDKSMISY

RAVIDIERLDKERRIQMANWTLRDGLTTPAGSQIRAARPYFRIGTAYAKPWSTIKLDPITGQPMTDKDGKEIWEGYCIDFIEKLSEKMNFDYDLVIPKDKSFGVKQPNGKWSG
VIGDLARGETDIVVGALTMTSEREEVIDFVPPYFEQSGILIVMRKPVREIALFKFMTVLRLEVWLSIIGALAVTAIMIWILDKYSPYSARNNKTIYPYTCREFTLKESFWFALTS
FTPQGGGEAP

>MpulIR5

MDHNIRNGTRNKKKMWIALNSFAIFACIVTAHPVKEWKYSGSLSYEPSVIYKNESLPRLSIGLMVPHTNFGAREYTKAINKAISNLHKASRGLRKYSFFENYQFTQHHIRNV
MMELAPTPTAILNSLCKEFLTVNVSAILYLMNYEQYGRSTASAQYFLQLAGYLGIPVIAWNADNSGLERRASQSSLQLQLAPSLEHQTAAMLSILERYKWHQFSVVTSQIAG
HDDFIQAVRERISDMQDTFKFTILNAVLVTKPVDLLELVKSESRVMLLYATREEAIQILTDARDLKITGENYVWVVTQSVIENLQTPNQFPIGMLGVHFDTSSTSLVNEIATAIK
VYAYAVEDFVNDPNNVNYSLNTALSCEGLGESRWDTGDRFFRYLKNVSVEGDQGKPHIEFTQDGVLKAAELKIMNLRPGVSKQLVWEEIGVWKSWQKEGLDIKDIVWPG
NSHTPPQGVPEKFYLKITFLEEPPYINLAPPDPVTGRCLVDRGVHCRVAKESEMTEMDIQSAQRNESFYQCCSGFCIDLLQKFSEEIGFTYELVRVEDGKWGTLENGKWNGLI
ADLVNRKTDMVLTSLMINSERESVVDFTVPYMETGIAIVVAKRTGIISPTAF

>MpulIR6

MKFHYVIMVLGGIVSVKSTTKIWKKNDNSIERTNSLSLLSHHVVDTYFHGCAVIAIHDDIVFKRHPGLLRNIFRNSPGVTFVQQHLNITRTEPPLRFRDKCYHFMIFLDNIYDL
SKIIREESVNKVLVISESTPWTVKEYLKSFPSRSYVNLLVITHSMSQRTGEGSYLLYTHELFTDGSGTSLPVLLTSWIGNRTTHENIDLFPEKLSGGFKGHRLVVATAHKPPFAIR
TDIVAKRSSEWDGIDIRIIHLLSKTLNFTADFRDPTAIDSPIYAVTTDVLNGQASAAVGGIYRTTNITSKLDTTFSHMEDCAAFISLSSLALPKYRAVMGPFKPAVWILICLAYFV
AIIPLSMNTNYTLWSLIIHPSRFLDMFWYVFSTFTNSFVVENPLLDTGLAKNSTSLLIGIYWVFTIIITSCYTGSIMAFITVPMFPQAIETAEQLLDEGYDIGTLDHDGWEIWFN
WTTIDDKVAVELLKSIEYVPTVKEGVKNASSAFFFSYAFLGSKIVLDYIVQDEFTPSWSNLRSTMHVSQECFINFGVTFVLQKNSIYTEPFNSVIHRIRESGLGDKIIRDVEWDI
QRTADGQRLPISDDYKRRKVVIEDRKLALDDTQGMFLVLGAGVLIAALALTIECCVKVYKEKFSGIITDAHTSVTSDATVPPMDYLKVYDPRNSEGSDTGMRRYNRFSI

>MpulIR7

MPFMTLGISILYKKPTKAPPSLFQFLAPMSLEVWLALMAAYVFTSLLFFVCGRICPAEWNNPYPCVEEPEVLENQFTLTNSLWFTIGSIMQQGSEIAPIGTSTRVMAGVWWFF
CLIMANAYTANLASSLTVENVHRPIKSAEDLANLNGEIKYGAKKDGATYLFFKGSNYSTYAKMYKYMEDNADDVFP

>MpulIR8

MTGYGLAFARNSKYVEMFNKRLLDYRENGDLERLRRYWMTGTCKPGKEVQKSSDPLALEQFLSVFYLLMVGILFAATFLLLEHLYFKYIRHHLPKSDKGGCCTLISLSMG
KSLTFRGAVFEAQDIIRQHRCKDPICDTHLWKAKHELDIAKMKIRQLEKDLEVHGIKPSQSRDKHEKLD

>MpulIR9

MLVGIVAIHAATFMIFLFEWLSPAGFNMKKNPSGDHRFSLSRSYWLVWAVLFQAAVHIDSPRAFTSRFMTNVWAMFAVVFLAIYTANLAAFMITREEFHEFTGLDDPRLAKP
WSHKPMFRFGTIPWS

>MpulIR10

MESPIESAEDLAKQTKIKYGALAGGSTAAFFRDSNFSTYQRMWSFMQSAKPSVFTKSNVEGVEWVIKGKGSYAFLMESTSIEYVIERNCELTRVGTELDSKGYGIAMPPNSP
YRTAISSAILKLQEEGKLHILKTRWWKEKRGGGKCREDTLKSGSTANELGLANVGGVFVVLMGGMGVACVIAVCEFVWKSRKVAVEERVSVVK

>MpulIR11
MGATINNVEDLAAQSKIKYGAIKGGSTASFFSASNVTMYKKMGQVMADARPEVFTKSNLEGVERVAKGKRTYAFFMESTSIEFHVKRNCDLMQVGSLLDNKGYGIAVPPN
SPYRTQLSGAILHLQEKGILRELKEKWWSMGSKNCSDVDPSTDSGALTVGHVGGVFLVLIFGLALSILIAIFEFVWNVRKVAVEEKITPSEAFIAELKFAVNVWVNEKPIKISH
SSNASKSSASDSGFTRAAS
>MpulIR12
MESTSIEYIAERECDLAQINGLLDSKGYGIAMRKNFPHRNKLNTAVLQMQESGQITDLKKKWWREKRGGGHCLTSGGGAAVEKLTLDNVGGVFVVLVAGVAVSILYTAWE
MLWGIGCTAYKERVPFKRELMDELKFIARCRGTVKPVKQRHDSSQSAHESTGGDDTPPYGGFVPTIITTTHEKDQP
>MpulIR13
MMYSITFKLAFFCSSAFFIHSTVSFNDFPSLMTANATMVVVIEKTFYERKVLAKETTVTTREAYEKSVANFVSAVTKKARERMNMSGLSIHAFQDTGVNLARDYTILLSVAS
CSTTWDLFRRARTEKLVHLAINDIDCPRLPIDEGVSIPLIEPGEELPQLFHDIRMTEKLKWNRAVIIHDDAFGSDAIGKVIQAFSHESSDSANNLGSTSLFSIARGSSEKITKQRI
KQLLSTFPPRQPIDDHFIVFVGHETIPHIIEAARALGMIHPASQWFFVVPDIAKFSHGNITFLIEYLSEGENIAFLYNETNQNLRQDSSCHDGAVCHAREMIGALGIALEKALLE
EIQLYERVTEEEYDATGIDKFARSRIIITHIRDELYNDSKSYEVRGSMCGRCIRWVVASAITWGNHIGPTDHNEPHELRQTGIWTPDPGFEFTDYIFPHVAHGFLGKTLPVATYH
NPPWQFLTTKPEASAELHDTMGNNWDGFVFDILEELMKKLNFTVKTIVVDS
>MpulIR14
MYVVHLWSVTKTDGARLIVGTWWLVVMVIVATYSGSLVAFLTFPKMEPAVETVDDLLERREEFTWTIPAGSFIEDFLLVSTTEGLIGYKQLLQENKGNAGKHDSVTYDENV
EEVKGGKHVVIDWATSLKISSRNEHLNTGECFFSLGNDVLLLGEPISMALPSDSPYLRIVNTQ
>AdorIR93a
MISVLLLVWCINYGSSYNDFPSLITSNATMAVVIDKGFFSNKDEYQNATKIIQDLITDTVKKEMNLGSISMRVFRDMNINFKDYTILLSVATCYLTWRLHEVAQKEELTHFAIT
DPDCPRIPDTDGITVPSIVPGEELSQIFLDLRMTDILSWNVINILHDDTFGDKATSSNDNVTILLSNANTCSRLVSDRDTISRVLKAISNKLPNKRMNLISRSIFSLRHGNTGSER
KSSVKKTLNDFHVEQLGHCFLVIATVDMVADVMSVANSLNMVHPGSQWLYVITNSVSGNLINTTFINLLTEGGNVAFMYNATNLDGFYKIKLKCYIKDLIEALAKALEYSLT
NEIELFKRMNEDEFEMIRLTKSKRRAELLKNVRIHLSRNTSASNNVCEQCLLWRFFSSITWGNFFSRNKNMAHLLDIGTWTPIIGVNLTDVIFPHIVHGFRGINLPIATYHNPP
WQIISMSKTGKKLYEGLIFDAINYLSMKLNFTYTVIMPETSQIPRSWNTSQFAKLGEKIKEMTMSTTKKVPLEIIDLVRQKKVLLAACALTVNECGNTTFNYTVPIFVQTYSFL
TAKPSQLSRVLLFASPFTKEMWACLAVSIIIMGPILYLIHKYSPYSTKASGLNSSWQCVWYVYGALLQQGGMYLPHNDSARILIGMWWLVVMVLVATYSGSLVAFLTFPRMD
TSILSVEDLIAHKDRISWGFPNGSFLEMYLQNAEEPKYHVLFSRAERHNDTEEERLVGRVKEGKHALIDWRSSLRFLMRKDFLLTGSCHFSLSMDEFLDEPIAMIIPYGSPYL
PVINAE
>AdorIR25a
MRFVELTIATCWFLGLCGKSVIYGQRTVASNRESAKTRPVNIFIINDETNKVANLSIITALETIKENYPNQLGNVWSVQVNESDINNTLDRVCNNWDSAVEKGGAKVPDLVID
TTTAGLGAKISNSFTAALGIPTLSAQYGQVGDLQYWRKLNTDQQNYLIQVMPPTDLIPEVIRQLSIQLNITNAAILYDYNFVMDHKYKSLLLNVPTRHVINETSQRIIEMKRQ
LLRLRDLDIVNYFILGNENTINIALEAADALNFTDKKYGWFLLTPDINIWPRCECRDISVLFMKPEFDGKNNSAVELSLPKPILLSAFYYDIIRLAVLAMKSALDDGEWPMEPR

HITCDEYNNTNTPERKLNFFGKLKEAYKNITPTYAGIKWGSRNGEHQAKFVMSVHLVGIKDGVVSNTIDSGSWNASISSPLQLTNNDVMNTTAVKSYRVVTVIHPPFVMYN
EEKNEYYGFCIDLLNEIKKTVGFQYEIRETDDKKYGSLNLDGSWNGMMRELIEKRADIALGSLWVTAERERVVDFTVPYYDLVGLSIMMLKTKTTTSLFKFLTVLENEVWF
CILAAYLFTSVLLWIFDRWSPYSYQNNREKYKNDDEKREFNLRECFWFCMTSLTPQGGGEAPKNLSGRLVAATWWLFGFIIIASYTANLAAFLTVSRLEIPIETLEDLSKQYKI
QYAPVINSSAYIYFKRMANIEWKFYEIWKEMSLNDSLSDVERANLAVWDYPVSDKYTKMLQAMEEAGFPASTEEALRRVRRLDSNNEFAYIEDSTTIKYLTMTNCDLIQVG
EDFSRKPYAIAVQRGSPLKDQFNNAILILLNKRKLEKLKDKWWKKNPSKKDCDAENSQSDGISIHNIGGVFVVIFLGIIFACFTLAFEYWYYRHRTKITKIDRNNTMKNKITQ
VKPLRFNLQPAPTHAFQNVQFRPRF
>AconIR1
MRGDLLRLVLLLVWGIISCHLLSVSCTAHPNNNNNKTERAAKSRLKSSNSPWRLVISLSHSQSPSRTDVQRAWEEARIESIQNELEVSYVEGRMAPMSDSLSYPLAFMNKFC
TDIENGKTILSIVIGGGSAARFLMTAAASLNLPSLWLPMTHRDFLRQGKRGRYESRLGSDSEEAGAAAAAVMHKANWHAFTLLIDTTLLPLHHLLSKDHQHHHSLLPRATIH
LPASEKSLKLRLRRISEEGGSGGVIVMACDLNNARKILNVAGKYHMLHGRFLWLWLDLKAELRPNEPSLLSSHAIYNFLTSSNRLENNLPMAENTMKHIANLVLDDEAHTN
SLSNLVNDIHRLQDYHWQDKAPIKKRDDKSFTFDDDDEARPSDKILNSKTFMPVGMLALRPSSMKISGGDAILSRMLRETSQALDNTFLEYKSAINRLREAQIKDYFIPTCFF
SSDKNHPMLEIKTNISRTLTMKLRESMRQISHDKAEFQLLNLQAVQFPGNKTQLRWTKVGLIRGGKDVRLDTIMWPGGGIVPAYLEQGGEKVGMPQYRIVTALAPPFIMVT
NLQEGSCLRGLPCRKGQTVKCCYGLTIDLLSLVARELSFRFDLYVAHDGLFGKRNGSNGSWNGVVGELLHGRAQLAFAPISVSARRAEVIDFTTPYYFSGVSFLAAPKENAE
ISLLAFLLPFSPELWIAIFTSLNITAMAVAVYEWFSPFGLNPWGRQRSKNFSIASALWVMWGLLCGHLVAFKAPKSWPNKVLINVWGGFSVIFVASYTANIAALIASLFFHSAV
NNYSDRSLLSQKVGAPRASAAEYYVQKADRHLGAHIARFSVSDVGEGVKRLLNGSLDILIADTPILDYYRATDDGCRLQKIGDTITEDTYAVALTKGHPLKESISKVIANYTS
TGMLDILQEKWYGGLPCILGRDGREANLGSSSEGQPRPLGVASVAGVFCLLGMGVVLGAIILAGEHLFYKYTLPRLRHRPKTSIWRSRNVMFFSQKLYRFINCVELVSPHHA
ARELVHTVKQGHITSLFQKSVKRVCILYHGELVVVLL>AconIR2
MEELSRWSNKYVVLDCPTDMAKEIVVSHVRDISLGRRTYHYLLSGLIMDDRWESEVIEFGAINITGFRIVDLNRRPVKDFLDSWRRLDPMTLPGAGRDSISAQAALMYDAV
AVLVEAFNKFVKKKIDRTNPKRPGTPSSSQPANVSDPLDCNTSKGWVTPWEHGDKISKFLRKTEIEGLTGQIRFNNDSRRHNYTLHVVEMTVNSAMVKVAEWTDEAGFQPI
AAKYIRLPTQEIERNKTYIVTTIVEEPYIMLKKPEPGKVLVGNDRFEGYCKDLADLIASDLGIKYEIKIVDDGKYGTENPDVPGGWDGMVGELIRKEADIAIASMTITSERER
VIDFSKPFMSLGISIMIKKPVKQKPGVFSFLNPLSKEIWVCVIFSYIGVSIVLFIVSRFSPYEWRVIKLNGGVDPAMTARGDPSLQHPHSQPGSLHAPNPCMANDFSILNSLWFS
LGAFMQQGCDISPRSMSGRIVGCVWWFFTLIIISSYTANLAAFLTVERMVTPINSAEDLASQTEVQYGTLHHGSTWDFFRKSQINLYSKMWEFMNSRKHVFVNTYDEGIRR
VRQSKGKYALLIESPKNDYVNEREPCDTMKVGRHLDVKGFGVATPIGSPLRSKINLSVLTLKEGGELEKLKNRWWYDRTECRHSDKQDSRNELSLSNVAGIFYILIGGLLLA
LAVALLEFCYKSHTEATRAKIPLSDAMKAKARLTIGGGRDFDNGRWYGLQS
>AconIR3
MIQCATFAVMLLTNYQVSSAYETPSPLAVQLIIEFSKWQSWKQVVIFDDLTEGGGHVMKYTRPLLRCFSEQGVGLSLQPTSAPRLPEALDICTHRLGAIVFLDRLNNTSADNV
LQLASSKRLFDYYISWLLITTRPDDVTIDTYLRNLTIGINSEVVVATASQFSSQQREQMRGFTNRTCRALRDYAHNHKYRNATVQSAVAMKNVTQNLAYSPTENSTACFRLV
HIYKIRISDNCSLVVDPLGSWNPGSMMLRHPVDVVLRDNFHRLPIVVGVLNGTSEDQDNDGVSLEDQTPDDRPFDDFINFVANGLNASLETVLHEKVGTITNKVWSNLLGD
VTNGNIDIGLGYITINDERLRDMCFSHPLIRYTRNIYIRPPESGTMRDIFLQPFNNRLLLGVAFVHVIIVVTIGTINYALRHVFNHSTARHYGYGEATLWCTSIMCMQGSPWNPC

TLSGKLALLASLIFALVTYNAYAGFITSILSVQVPGIKTLEDLLQNNFKVGYSEYDDEFMRNANDSNLRQLYIKAFNGRESRLDTTLGLQRAINGRYGFFVSATSARRILKTTL
IQQRCYLKEIEVKQTFTTVALPMEKDSPYKKIINLSILRMRERGVIDRIVKKMLPDMPNCNDPTTFHSARIADVYSAFIIIAAGIVMSVSITLLERIWSQRNNLKNRLTKGVSHH
IKKNSQFPTHLTLQASENLIYNHGNSLRRRTQRPNMSLLPFQN
>AconIR4
MGQSVNKNNITSIKTETALMYDAVHLFAKALHVLDQSQQIDIKPLSCESSDTWSHGYSLINYMKIVDMHG
LTGLIKFDHQGFRSDFVLDIIELNSKDGLKKIGTWNSTEGVNFTRSFGDVYIQIVENLQNKTFIVTTILS
APYCMLKDSTMELKGNAQYEGYNVDLIHEISRILGFNYTFTIVPDKKYGSYNKVTKEWDGMIKELLDQRA
DLAIADLTITYDREQAVDFTMPFMNLGISILYRKPLKKPPNLFSFLSPLSLDVWIYMATAYLGVSVLLFL
LARFTPYEWFKCSYGKNCNHEQNRFKLSNCFWFTIGSLFRQGCDILPKFSPYEWENPHSCNAQSDDYENE
FTLLNSLWFTIGSLMQQGSDIAPKAVSTRMVAGTWWFFTLIMISSYTANLAAFLTVERMESPIESAEDLA
KQTKIKYGALAGGSTAAFFRDSNFSTYQRMWSFMQSAKPSVFTKNNGEGVEWVIKGKGSYAFLMESTSIE
YVIERNCELTRVGTELDSKGYGIAMPPNSPYRTAISSAILKLQEEGKLHILKTRWWKEKRGGGKCTEDNL
KGSSAANELGLANVGGVFVVLMGGMGVACVIAVCEFVWKSRKVAVEERKHKTSEKSICEGFTDLH
>AconIR5
MEVTDDPNRGYLIDNVRYEGYAFDLIEEISKDLDFEYDFEIVPDKQYGVYDPVTKQWTGLFGRLMRREADLAVCDLTITKFRLSLVDFTTPFMNLGIKILFAKPQNEVPDLFS
FLSPFSTDVWIYVATATLALSLSLFILARIAPTEWHSAHPCASDPEELENNFNLKNSLWLTLGSLMQQGSDILPVAPSIRMVTGMWWFFTLIMGSSYTANLAAFLTNVKMDAS
ISNIDELAAQSKIKYGAIRGGSTAQFFEGSNDSKYQKMWQTMLEARPEVFTTTNKEGVERVAKGKRTYAFLMESTSLEYAVERNCNLMPVGSLLDNKGYGIAVPPNSPYRTL
LSGAILKLQEKGTLRELQTKWWLKGSKNCTQGEPEPSSGELTMAHVGGVFLVLVLGCTGSFVIALCEFIWNVRKVAVEEKITPGEAFIAELKFTLNLWAETKPIKISRSSNAS
GSSASGGNGFTRAASAARSIVGSFLRLDTLGK
>AconIR6
MQFASEIAPWIDMSYPNWLIFFRNETKIQNFFSDIYVPFDCTFMVSTSEFEDQETILEVYQIERDKELRQGLFGTWDIENGIKLSTSSLYQRRNDLFGQTLRVVSVHDPPSSIV
MTDDKEIVNVGGFFGGIMELLKESMNCTITYQVADEFGNLLDNGSWTGAINQLVDKTSDIAAAELMMTADRLESVKFTTPLYSTKTRTFIKKPSSSTLKWDAYISPFSFGIWS
TMGIMIVLTSISISVIKSLTLILCSKIEENKEPSKILDISFAVFGAFCSQGMEMSMTDSVRIIHLVIHVTAVIILAAYSAALISALAVKTFIMPFTTMKGLLDDATYRFGVIGASADY
SFFQNSSDKIISDLSDNFLAKEVDLPINYLDGLMRVCSEDKYAFMALDNAVAQLKSSVDCVVVPLDTISQTSIAMALRKDSPFRGLINSKLFTVFCYYATVVCCRSY
>AconIR7
MTINYARESVIDFTKPFMNLGIGILFKVPSSQPTRLFSFMNPLAVEIWLYVLAAYMLVSFTLFVMARFSPYEWNNPHPCLAESDVVENQFSVSNSFWFITGTFLRQGSGLNPK
ASSTRIVGGIWWFFTLIIISSYTANLAAFLTVERMITPIESAADLAEQTEITYGTLEGGSTMTFFRDSKIGIYQKMWRFMEAKRSSVMVSSYEEGIKRVLEGNYAFLMESTMLD
YNVQRDCNLTQIGGLLDSKGYGIATPKGSPWRDKISLAILELQEKGVIQILYDKWWKNTGDVCNRDEKSKENKANALGVENIGGVFVVLLCGLALAILVAILEFCWNSKKN
AQSDRVSFSHLFLAY

>AconIR8
MTNTKLEPPFGDIGELFNSTKYRIVVYNGSMAFDRFKRFASHFRPEHQKRVIFDGSKTRMYHYACQNKNKIAVIESDDRRRATGRKICILVKAGAPLFRTWITSGVIKGFQNK
RSLDLGMIRLHEAGLIDGLKDRWMAPD
PIAWDGSNVESIRLNHIYMLFVILLFGAILSVIICVIENIIFLHQQNSTRRKKKRKRRHVRKFNQILI
>AconIR9
MENLTCATVKGSAVDMYFRRQVELSNMYRTMEANNYDTVEEAIRDVKIGKLMAFIWDSSRLEFEAAKDCELVTAGELFGRSGYGIGLQKGSPWADAVTLAILDFHESGFM
EVLDKNWILQRNLQTCEQYEKTPNTLGLENMAGVFILVGIGIVGGVGLIIIEMAYKKHQIKKQTKLELARHAAE
>AconIR10
MDIASAQKNESLYQCCSGFCIDLLQKFSEEIGFTYELVRVEDGKWGTMEQGKWNGLIADLVNRKTDMVLTSLMINSDRESVVDFTVPYMETGIAIVVAKRTGIISPTAFLEPF
DAASWMLVGIVAIHAATFMIFLFEWLSPAGFDMKKTPS
>AconIR11
MNPLAIEIWLYVFAAYVLVSVTMFVVARFSPYEWNNPHPCHSGPTEIVENQFSLANSFWFTIGTLMQQGS
DLNPKAASTRIVGGIWWFFTLIIISSYTANLAAFLTVERM
>AconIR8a
MQASSWPLHTLIFVMILRAVQPQTTVKMMILIEMGENNILEIISDALAMAERSLSGDQSVKIDIVPVVVDRDSVDESYNHVCSELFSGVTLILDMTYTGWDRVYEIARNHSI
MHVRSAAKITPYIQASNDLLMRKSATDVALIFQSEKELNQSLYRLIGESTIRLVVIDELSDITVTRIRAMRPSPSYYMIYASTDSMEGLFNAALRGNLVSRNDTWYLVFTDMN
YRNFRYFQADVLDVNIGVLSMNSVVCCRLLRLDTSCTCPTDTQIYEKFIERMIFAILEALDRLQKSNTDLEPRKFSCSSPNENIPTPEENESSTSFYKILVEILTSNNALEFISESS
EGPYHMPMIIYEPIINIEILKNGNLEDLATWRRDGIQTAPGKEILPVKHFFRIGTATALPWSMIKTDSTGAPMRDEKGNLMWEGYCIDFIQKLAEKMKFDYEIVNPKDNLFGE
KLENGKWNGLIGDLTSGETDMAIGALTMTSEREEVIDFVAPYFEQSGILIVLRKPVRKSSLFKFMTVLRLEVWLSIVGALTLTAIMIWVLDKYSPYSARNNKKIYPYPCREFT
LKESFWFALTSFTPQGGGEAPKALSSRTLVAAYWLFVVLMLATFTANLAAFLTVERMQAQVQSLEQLARQSRINYTVVNMSSSHQYFVNMRSAEDKIYKMWKEITLNSSSD
QVEYRVWDYPIKEQYGHILQTMLTVGLVNDTQEGFRKVEENENSEFAFIHDSAEIKYEVTKNCNLTEIGEVFAEQPYAVAVQQGSHLQEEISRTILELQKDRYFEGLTAKWW
NSSMKCSTADDNEGITLESLGGVFIATLFGLALAMVTLAGEVIYYRRRNAIQSEKAKDTSQEKVSSGPKIMTRINSKSKLRQLKPSPPVSFIGRHRGLRPRISHISVYPKTFPFK
E
>AconIR75u
MKNFAKLIFICLVISSEAHKIDQTIGEFIIDVTSSMYMRSSFTAFFCIDTTDAIIFSRQISRRQVMHEIIHDLKNINLTNLLTKISSQSFFILDFDCPNVEEFLLQMDLNEMFIAPNK
WLILQNLENFPDKNLTLTINTEDFKRYFEALQVYPDSEVTVGQRIDENFINIISMYRPGPSGNIILEDRGFWSDLEGLQISDKNPTSRRRQDLQGTHLKSCLVMTDLDTLNHLS
DYKDKQIDAVTKANYIWVQHLVNRMNATIDFTWRNTWGYQDKNGTWGGMIGLLDRGEIDIGGTATFLISSRIGVVDYVQLYTPVGSRFVFRRPPLSYVTNLFTLPFEQSVW
IAIGILLIVVSILLSLTMRWEWARIMESPSKYHLGGELESKPTASDNFLILLGAVAQQGFEYLPRTISSRIIIIMLLIAAFSLYASYTANIVALLQSTSDSINTIEDLMNSGLKVATYDI
VYNRYYFGSFEDPVRKEFSQKLIANKSSAWLTLEEGVARLRQGLFAFHMDTSAGYDLVQKTFEEDEKCGLSEIDMLNVLNPMLVIKHQSPYSEIIRVGALWVQETGLTGHD

VPRLFSKMPKCSGQTSFVNVGMTECRAAMLTIVYGMIASVGLFFMEILWHKCSRNDEIDEEVMEASEESPDPEIQSLE
>AconIR64a
MEARAMISYHQISDKIDLKSILHVDYYHIGVLLDYDCEKSMSFLDQFSRLLVFNESYHWLMLTESSAPPVDVLRDLPLSVDTEMTVAMRNNDSFQLLDVYNPSHRHGGRV
NITYKGQWTPQGGLRNQLTQFKYRRRQNFNLLPLNFSIVLLYPPKPDFHTYLTEPINPHLDSMTRYHYALVMQLKDFFNFSMNLQQAKTWGYLVNGTFNGILGDMVKGIV
DVSIAPFQYKEERMDVCEFTVETWTVRPMFIFRHPRTSDVRNGFLKPFAKEIWWFVLLGGLIYWIFLYGTAKIGIYFSKSDAENTLMSIPASETGLITLAAVSQQGLSEGPTNIS
GRIVFLSLFLWALLLFQFYSASIVGSLLSPPPRWITTLKNLSDSSLDCAMEDVAYSYDYYATTTNPMSLEFYDSKIKPTKKNPKGSYLKAAEGLNRMRKGAFAFHLDTATGYR
IIQDTFSEDEICELQEVELLTPRVTTLVTAKHSPFKKMIIYGLRKIVEHGITNRLRKVWHHSRPKCPESHSSKPQPVPMGEFSPALFLLLMGLAFSVFVMLAEYLHYYYNRPVD
KEDSTPEISSRKSSENFFNDELPNYNSNSA
>AconIR75a
MLIYCLDDIHLIKSFSNIGIYANVFQVEYYKPLDQTWTATLRSMGIWLDIRCIPEENITSILTEAGTSKMFGKLYSWLLVGNDFNQTLSYLDEYEMGLLNDIVIAVEREIKFDM
YDVYNPAKNRGGELNITRFGTWDAKTGLNVELTQSLFARRYDLHGIEVLVSIVTVYCDGQDLHHCLTTYDVLGVDLLSKFGYAMLVTISEIYNFRITVTANREFWGKFPNGS
LFGMMPSVQRGEYDFAGSPVNSNWIRMGYTNLFPVWTSRIFIIFRDPSKSFKFTELFDPLAGESWMGVMSLLALIAFLLTWILWFEEGGFWYQQFGWSFFITIGALCQQGLL
AVSNRISGRIVLLFLMVFIFLIVNYYSGKIITARIHIKPPRLNDSLYELVKLTARNYRFCNYPYKPLGNYVQTTKDKDILFFFKYVWSKVPLPNQMPIVVGLERVRKGKFAFVIN
EGDSYPYINNNFEPEIICELNEIHILKPHPFLLFEKPHSPFREIALVGQLKMMSTGVKSRNVHRWTPTPYKCDPQIRSNEPLNIRDVISALLILGSGMTLSVFILILEILNAKLSEL
KNTRIIYLKNMKKKPRKS
>AconIR25a
MMYDKETGEWSGYCIDLMKEIQEILGFEYEIYVAPDNEFGNMAPDGTWNGMIKELMDKRADIALGTLSVMAERENVVDFTVPYYDLVGISILMLKAKQPTSLFKFLTVLE
DDVWLCILGSYFFTSLLMWIFDRWSPYSYQNNKEKYKDDEEKREFDLKECLWFCMTSLTPQGGGEAPKNLSGRLVAATWWLFGFIIIASYTANLAAFLTVSRLETPIESLDD
LSKQYKIQYAPVKPSEAYTYFERMANIETKFYEIWKDMSLNDSLSDIERAQLAVWDYPVSDRYTKMFEAMKESGFPTSRDEAVARVRKQLQNHSATDFAFIGDATAIRYLEM
TTCDLIMVGDEFSRKPYAIAVQQGSPLKDQFNNAILQLLNKRKLESLKNKWWNQNPKRRFCKKEDDQSDGISIANIGGVFIVIFVGIFLACGTLAFEYWWFRYRPQQEAKRL
AAQGNQNIKPMRFHLKPARPAFQPGSEFRSRF
>AconIR21a
MRTSMIASILCRLACLNIVQASQKAPAAAVHDAVARTDSLSTLLHYILANNCHGCAVIIIHDGSLYDQHSGLLSGLCKDSPAVSFLQQYVDVLQNKAPRITLSDKCFHYLIFVQ
NIYNLNKIIPKETVNKVIVVAESTSWTIQEYLKTISSRSYTNLLVITHSLSRRTEQGSYLLYTHELYTDGSGSSLPVLLTSWIKNHTSKQDINLFPEKLRGGFKGHRLLISTANQP
PFAIRKNIISQQRSVWDGIDIRMIRLLGATLNFTADFRDAIATNSSIYAVMEDVLNEQVSAAVGGIYRTDDVIIKFDNTVSHMEDCAAFISLSSLALPKYRAVMGPFQPGVWVL
VCLVYFIAIIPLCMNTNYTLWSLIIHPSRFLDMFWFVFSTFTNSFVVKNPLLDTGLAKNSTSLLIGIYWVFTIIITSCYTGSIMAFITVPAFPAAINSAEQLLEEGYDIGTLGEISSS
FCNLIFFFFTL
>AconIR93a
MGKSLPIATYHNPPWQYEMSQTQSDEKQAGSMTSKWDGLIFDVVEELANRLNFSFKPVVVEAPPEIIIAKADPLRASMSASEKVPDAVTELVRSGSVFLAACAYTISTHGKD

PSINLTQAISLQTYGLLAPRPKPLSRALLFASPFSNEAWACLASAIILVGPVLYAVHTLSSRTTDTELKDPGSVLLGLDSPSRCIWYIYGALLQQGGMNLPKNDGARLIVGTWW
LVVMVVVATYSGSLVAFLTFPRMEMAVKTVDDLLARKSEFTWSIPAGSFLEDYLLVSSAEGTIDYRNLLQEHEPHAEKHGTTTYLQNVQKVKRGKHALIDWVTSLMISTRN
EYVASGSCHFSLGTDVLILGEPISMIVPAGSPYLALINVQLQRMQEAGLLNKWTADRIPVKDQCSEGGIIARETDNHKVNLEDMQGIFFILLLGYFIGTLTLGWEFIRRRLKIA
KEAKLIRPFID
>AconIR64b
MNLMKGSTWGYLINGTFNGILGDMIKGIVDFGATPFQYKPERLDVCEYTVQTWLARPCFIFRHPKKDDLSNPFLKPFEQRVWYWIALFGFVNWALLYLTAKIEKIINWKPLI
NTLDSHPASETALITSAAICQQGLSDGPRLFSGRIVFLSLFLWGFLLYQFYSASVVGSLLAKKPRWINTLKDLADSNLEIGIEDIAYNYDFFATTTDPVAQRLYNEKVAVNKKR
KKLPYYSAEEGIARMKNGGFAFHVDVATAYKIIEETFTAAEICDLVEIQLFPPKHTATATARYSPFKKMVTYGMRQIVERGMARRLRHIWMHRRPQCPESHSEEPVPVILAEFS
PALFLLTCGCCFSTIVMITEMIVQRKHRLEPENHLTGVFTQKSPSALSESSRKFEDISSPIDPDEVKFSAN
>BimpIR25a
MRLVGFTIAMYGFLGFCSESAVYAQRNVVGNRGSTKTRAVNLYLINDEANKVANSSIIAALETINEKYPNYLGEVWSVQVNESDVNDTLDRICKPWDSAVKKGGTGVPDLV
IDTTTAGLGAKISNSFTAALGIPTLSAQYGQEGDLLYWRNLNTDQESYLIQVMPPTDLIPEVIRQLCIQLNITNAAILYDRNFVMDHKYKSLLLNVPTRHVINEASQQIMEMRT
QLPRLRDLDIVNYFILGDENTINIALEAAEALNFTGKKYGWFLLTPELNVWPRCECKNMNILFMKPEFNKKSPIESSLSNPVISSAFYYDLIQLGVRAMKSALDDGEWPVEP
KHITCDEYDKTNTPERKVNFFNRLKESYKNMTPTYAGIKWGSKNGEHRAKFEMSIHLVDIKDGIVSNTIDSGSWNASISAPLQITNNEVMNTTAVKSYRVVTIIHPPFVMYNE
ENGTYYGFCIDLLDEIKDTVGFQYEIRETEDRRYGSLNPNGSWNGMMKELIDKRADIALGSVWVTAERERVVDFTVPYYDLVGLSIMMLKTKTTSSLFKFLTVLENEVWFC
ILAAYLFTSVLLWIFDRWSPYSYQNNREKYKNDDEKREFNLRECFWFCMTSLTPQGGGEAPKNLSGRLVAATWWLFGFIIIASYTANLAAFLTVSRLEIPIETLEDLSKQYKIQ
YAPVINSSAYIYFKRMAAIEWKFYDIWKEMSLNDSLSDVERANLAVWDYPVSDKYTKMLQAMEEAGFPASTEEALRRVRRLDSNNEFAYIEDSTTIKYLTMTNCDLVQVGE
DFSRKPYAIAVQQGSPLKDQFNNAILILLNKRKLEKLKDTWWKKNPDRKDCDAENSQSDGISIQNIGGVFVVIFLGIIFACFTLAFEYWYYRHRTKITKINLNSTTKGKVTQV
KPLRFNLQPAPTHGFQNSQLRPRF
>BimpIR21a
MILVTLFLQIILVSGKSVFYKEHQCVNSGNNLKSVVEEIVEEIIDQRNCIVFVSDSVYRNLVDVKNIKGSSTVLKYEIALRDNEQFLQPRRQVQRILIDGKAVNCSAYIILIANGF
MAAEFLQYTERERLINTRGLFLLLYDSRLFRSHLHYLWNRIINVVFIRQYNAYKYRSGEKASKERIDLDTVYFPLRKRKSIVTKYIDTWYKGKLLYGTNHFTEKITNLQEKHL
QIAVFEHIPAVTTKSRAYYNKQPNNNTEGLGIEFELMQIISKAMNFKPKYYMPDNITLEKWGINEDNQTHVGLVGEAIQGKAAFYLGDLHYTLHHLNYFDLTTPYNTECLTF
LTPESLTKNSWKLLILPFKFYTWIALVLTLILGGVVFYFLSISYKKHISLYKNQMHFQNTSMKKEIKGLYLFTEIGNSILYTYGMLFQISLPSLPSSWAVRVLIGWWWIYSILVAV
AYRASMTATLANPVARVTIDTLGQLAKSSIEVGGWNKENKNFFSMSSDLSSQEIGNKFKLIQEEDKAIEKVANGSFAYYENSYLLRHVRVKRQILEKEQKENITTVDISSKHN
LHIMEECVINMPIALGLEKNSPLKPRVDTLIRRIIEIGLVEKWLSDVMEWSKIMEIRQETESEKALVDLHKLQGAFIAIIVGYVLAFMVLIGEILYWKHIVLKDPKFDKYHLDIF
YSINNPKI
>BimpIR93a
MISVLLLLWCVNYGDSYNNFPSLITTNATMAVIIDKSFFDNNGEHRNVMGVVHDLIINTVKKEMHIGGIVVRIFRDADVNLWQGYTILLSVASCCITWRLHEVARKEELIHLA

ITDPDCPRIPETDGMSMPVVVPGEELSQIFLDLRMMNILPWNVINILHDDTFGRDTISRVMTAISDKLPNKQVNLISRSIFTLKHETTRSERKSSVKKTLNDFHVEQLGHCFLVI
ATVDMIADVMGVARSLKMVHPGSQWLYVITDSATKNMTNMTAFVDLLAEGGNVAFMYNATNLSNYCEIKLICYVEKLIQALAKALEYSLTNEIDLFKSMEEEKFEMIRLTK
RERRAELLKNIRIHLSQNAFASEGFCGRCLLWRFSSSITWGNFFSRGRNMAHLLDIGTWSPGFGVNLTDVIFPHIAHGFRGTNLPIATYHNPPWQIISVSKTGQKLYEGLVFDA
INYLGSKLNFSYTAITPEDLYTHSVLCPSSSSSAKRFCSXKIKEMTMSATRKVPKEVIDLVRDREVLLGACAITVNENKKDAINFTVPIFVQTYSFLTSRPKQLSRALLFASPFT
KETWACLAVSIIVMGPILYLVHKYSPYSIKTSGLKSSFQCVWYVYGALLQQGGMYLPHCDSARILIGVWWLIVMVVVATYSGSLVAFLTFPRMDASILTVDDLLARKDGITW
SFPNGSFLEMYLQETDEPKYHTLLSRAESHNDTEEEKLVERVKDGKHALIDWRSSLRFLMRKDLLLTGVCHFSLSMDEFLDEPIAMIIPHDSPYLPVINAELHRMLESGMMN
KWITERMPIKDKCWEVPGSNQAVNKRKVNVTDMQGIFFVLFMGIILAFFFLFCECYCHRRKISKERKLIHPFVS
>IkuwIR8a
MDKKADLAVGSMTINYARESVIDFTKPFMNLGISILFKVPTSHPARLFSFMNPLAIEIWLYVLAAYVLVSVTMFVVARFSPYEWNNPHPCHSGPEIVENQFSLANSF
>IkuwIR75c
MTEYGEGWFAESGHAIFATLGVFCQQGLVFVPNRLAGRIACLFLLLMGVLLSNYYGASVVSARLNEPPDKMNDSLYSLAKSHMTLASEEFRNVDDIIQFRSTGSRS
>IkuwIR75b
MTEYDEGYFAETGHAIFATIGALCQQGLVFVPNRIAGRMAFLFLLILGVLLSNYYGASIVSSRLSEPPNKMNDSLYALSKSHMTLTSEPYRYIDLNLAFRAQSEWEALYFYEKI
WNKLPMTKYVSTEEGLSKVSKGGYAFHTSAEVAYPYIEANWDDKMICAMTEVHIITPRILSFWERVDSPFTELMRVGLVRTANAGLRTRTIKRWKPKTPYCSPDTLSLESISI
FDIAPALLVVIGGTATAVIIYFFEVLMFKMSLGIPLLFKLRNN
>IkuwIR21a
MYTYGMLLQVSLPRIPEAWAVRVFIGWWWLYSILVTVAYRASMTATLANPISRVTIDTIADLVKSRLPVGGWTDEQRDLFLSSSDVDIRKIGERFELTTNEEEAIARVANGSFC
YYENIYILRQARAIRRQLEFNQMRIAERHNEIFKVDRDLHVMQDCLIDMPIAIGMDKNSPLKPQVDRTIRRVVEAGFVAKWLSDVTEWTKIVELRFRMPPEQTLVNLHKLH
GALVALAIGYFCSFVALAAEKLHWKYIVERNPLYDKYQMDIFYAKAQMD
>IkuwIR75u
MSRYNVMHNLWKSIERLDPLTLTSRINNQNFFVLDLECPGALDILIKASENRMFVAPAQWLILQDLRTPSKKTGNSSRDLGSLTSVDDVSWLYATFGNLTMYPDSQIILGRRLS
EDFFSILSPYRPSPQRDLILEDRGNWTLKDDLVLVNRDPASRRRQDLRRTELTSCLVLTNQDTLNHLEDYRDKHSDAVTKANYPWVLHLVNRMNATVKFRSENTWGYRAK
NGSWSGMIGLLDRREIDLGGTATFLIPERLGVVEYIQLYTPTASRFLFRRPPLSYVSNLFTLPFGRNVWIAIGVFLVLVIGLLYLAMKWEYKMKSLEKGSPKVHWSANIDDEP
SLSNNLLVIMGAVSQQGSSYEPRAVPTRIIVLMLLLATLSLYASYTANIVALLQSTATSINTPMDLM
>IkuwIR75a
MKLNVVTVVKYYPAGTDLREYLEDYKTKSLDPISKYGWGIASLIADLYNYTMHIDPVEEWLTEVNGTEQGMMAKLDRAESDFGISPSSAILRYAAKPYAHMLTPGYPYRSF
FIFRSNEVETLKWMELMTPFAFGSWYAIVLTAVFSIMIITTIQVTEYQEGWFPEIGHAIFATLGVLCQQGLTFVPNRIAGRIAFLFLLFFGVLLSNYYGASVVSARLNEPQDKMN
DSLYSLAKSHMTVTSEPFRYIDFNLVYRATWEWEVRYFLDNVWLKQPETKWVKPEEGLKNVALGGYAYHTSPEVAYPYVEANWDDKAICDMTEVHVVDPRILVFWERVD
SPFTELTKIGLIQTAITGLRQRTIKRWKPKTPYCSPDARSLESITIFDIAPAVMIVIIGNVLAGTICFFECVMYRLHPNDTAGQTKKKKPRLGGKKALIRIKKNNEMNLQ

```
>IkuwIR64a
MKIYIIFLIFLVVNTPCRSDKIRASFTKDYYKDRNIQHIVTFDCWDPYDKLSFARDVMEMDTRMSFITIMDNLEMKKILAVNYYHIGVILDIDCPRSAIVFDQFSQHRLPFNES
YHWLVFTESMTIPTEMLRHIPFTVESEMTLASRRQDSYVLHDVYNPSYRHNGQLNITWKGTWTPRDRLDDELTQYKYVRRGNFHGLTLNFSIVLTNPALPDFNTYLTTPINR
HLDTMIRYHYALVLQLRDTYNFTINMQRAKTWGYLVNGTWNGMLADMMAGLVDISVPPFQYKEERMDVCEFTVETYTVRPCFIFRHPKKNDLRNPFLKPFTSDVWYVIA
VVGIIYWLLLFLTARVEKHYRTEVPINTLDTHPASETGLITVAAITQQGLSDGPRVISGRIVFISLFLWGLLLYQFYSASVVGSLLAEPPRFITTAKDLMDSNIDVYFEDIGYYYD
YFRTIKDPFVLAFYDKKVKPTKKRPKGSYTDAVHGLSLVKKGGYALHVDTATAYKIITDTFTDDEICDLQEIEILKPKKVTLVTSKKSPFKKMVIYGLRRIVEHGLTHGLRQV
WVA
>IkuwIR25a
MAQRLNYTGHKYGWYAFTQDDFDPQCECTNLSLLFFKPKISVTSQQRLGDLTNGGLLPKPVLTSAFYYDLARLGILAMRKAIQDSIWPGEPKHMMCDSYNGNNTPTRDFD
FLKLLKTASSDVSFQATFAGFAWGKKNGEHRASFDMSINMITISGGNPVSSQELGSWPAGIDTELEVSTNDSVIRNHTAVTSYRIVTVVKPPFIMYDAETDKYSGYCIDLIENI
REILHFDYDIVQAPDKKFGTMDQNGNWNGMIRELKEKRADIALGALSVMAERENVVDFTVPYYDLVGISILMQKPKTEPSLFKFLTVLENDVWLCILASYFFTSFLMWIFD
RWSPYSYQNNREKYKDDDEKREFSLKECLWFCMTSLTPQGGGEAPKNLSGRLVAATWWLFGFIIIASYTANLAAFLTVSRLETPVESLDDLSKQYKVQYAPMLNSEAYTYF
ERMSDIETRFYEIWKDMSLNDSLTDVERAKLAVWDYPVSDKYTKMFQAMKEATFPVDMAEALRRVRRQDPNITNDFAFVGDATDIKYLSMTNCDLIMVGDEFSRKPYAIA
VQQGSPLKDQFNNAILQLLNRRILEKLKNKWWTHNENKKTCKEEDNQSEGISIQNIGGVFIVIFVGIGLACITLAFEYWWYRYRPNHNANINHNNANPKAVPVRNAKPVRF
TLKPARTPAFQSSGQFRSRF
>IkuwIR8
MVWAGFAMIIVASYTANLAAFLVLERPKTKLTGINDARLRNTMENLTCATVKGSAVDMYFRRQVELSNMYRTMEANNYDTAEDAIQDVKIGKLMAFIWDSSRLEFEAAQD
CELVTAGELFGRSGYGIGLQKGSPWADAVTLAILDFHESGIMEGLDNHWILQHNLPQCEQFEKTPNTLGLKNM
>IkuwIR7
MRIAYFSIFVSALVVSAAYSASLISFLTLSSPSLPFSDLLGFAKDGSYKLIVYQQSSQYDFISSSNDSVFVRMMTLMKPLQDLPRNTNEGWTQVCEERVALYTSTVEKITMDGI
TCNVIGIDTGHVESLAMALPAGSQYTDVVNYHIERFKTNGIFKRLKALYYDTEVPAATTYDVVELSGVTP
>IkuwIR6
MFTLFSGEIWSLIGAIIIILSLASLAMERILVRHGGRQLRSRSYAEHFFYVSSTLFNQVNAVDSIASGSKILSLTISVFCWLILIGYSSHLIYSMTEETLDAPFDNLDTLFESTNFHI
AVLHGSVAHSCFTSAYTYEIFKRIWKKNRVKVVSNVEALYRQVCSNPHKYVLLDAVDTMSVRKNTECSLIPVGEAYFAVPVASGISRRLKSKRSLDIGIMKLHEYGIIDALKK
RYMSRPNRASNLRLTDDLAIDMDRVSMIFGILCFGICASIVVCLAENMHFTFKKRDSTIESLIKRLPIINRIIKTKIR
>IkuwIR5
MPNGSYDGLVGMLLRDEIDIIPRTGYYVSESKVMAYTTPVWDNRYRTYIRPQFKVDSTWMFNMFASEVWYSIFCLLILISALGYTAQRISIKVSNDQRSPHNLHDHVFWTFA
TLCCQGFIPDSLYQRVKILALTKKMMAWLVLIVLSSHLISYMTNRKALLPFEDLGTLFNSSSYNIVIFKGSMIYDKFQRNVMRTTTRAIIDERVRPVATPERLYEKGCWGKRN
YAIMESEDRQRAYGRFMCKLVPVGIPYFATWVAAGVAKTFKYKRTIDIGILKLNEFGLTDGMKDRYMESATVTYDSSPFTSVNFDQVLMLFMILCMGMIA
```

>IkuwIR4
MHDCASFISLASTALPKYRAVMGPFQLSVWLLLCSVYMIAMIPLSMNSHYSLKSLLTKPSSLNSMFWFVFSTFTNSFSVKNPLLNSGLGKNSTSILIGIYWVFTIIITSCYTGSI
MAFITVPVYPTAMETADQLLRNRYRIGTLDHDGWNTWFNSTTMDDPLAIKLLKNVEYVPSVKEGVKNASHAYFWPYAFLGSRILLEYIVQAEFAPNWSTKRSLMHISTECF
VEYGVTIVMPMESIYTEAFGKVVIRARQSGLTQKIVKDVEWNGQRSASGKLLSVASGSKIQRVSVEERQLTVDDTQGMFLILGAGTLIGFFVLSIECCVHSIKKRWHSKVAN
EENNNFADGSYERNLDFWLSDNNVDSVGRRYSTTSI
>IkuwIR3
MTSSACIFPIFPAFFFTLFTFMIVPFVNAQTPVKLLFVVESTDTSIKGLLNDFLPTTEKEFGGGNINLDAAIVEVDRDNIEGSHQKVCAALYDGVTLILDMTWTGWDKLRKLA
TDNSIIYKRTDSTIIPYVQAVDDLLAKKNATDVALIFENERDLNQSLYYLIGNSIIRLVVIDELTETTVAKVRAMRPSPSYYAIYANTSKMETLFKTALDGGLVKRNGIWNLVFT
DNNYKNFKYISGNGNLNVTLGVLSMRTDVCCRMIDEMPCNCPTDFQIFPHYFKRLLGLLVGTIAEVIKSGVSVETKSAQCDAKNSTSGLSNATIDAFNANLVTTITAQNDVF
ETTSDMIYYKAEIELETLQDGNLEKLGNWSRRTGIQVAEGKEILPAKRYFRVGTTEAIPWTSIKRDSEGRPMKDAEGNEIWEGYCIDFIQKLSEEMNFEYDLVIPTDRSFGNK
LPNGKWTGLVGDLARGETDIALAALTMTSEREEVIDFVAPYFAQSGILIGSINKLV
>IkuwIR2
MSYPTWLIFFRDETRIEDFFMEIYVPFDCTFMVAQGEGNDSNTEIITEVYQIDRDQELRHSTFGTWHPKHGITTPTVALYQRRNDLYGKILRVASIHDPPGSLIVRDENGQMIG
LGGFFGGIMQLLEESMNCTVIYHESFNWGQFLDNGTWTGAIGLLTRNDVDIVAAELMMTRDRLEAVDFTTPLYSTKCRTFIKRPSVSALKWDAYITPFHSGIWSSIGILIIVTS
ATISMVKTITPIISASMQDVEKSPSNLVDIILAVYGAFCCQGMEQSLLDPIRMVHQVIHLTGVVVLAAYSAALISSLATKTFVMPFKTMEGLLQDGTYRFGVIGESADYSFFQN
TSDEILQVLFAEVLTKEKDLPNNYLDGLNRVCEEDKYAFMTLDTVVSQLNPKVNCKLEPLDTIMQASVAMAVRPRSPFRGIINTNILLIRDSGILQRLLHSEWALDDDRVNG
WTTVEIGDILPLVLVILGGIFFSLITLGSERVIAKQKHRKSNKKTKEAIVQPMISFRKHKKASSDLFKH
>IkuwIR1
MSLTEMLIVIDNDVLESSMLFNVSKYQTAKAKIATRNGLWTLTDIFLEPRVFKKFSSHDEIIHKKGIANLGGRKLQAAAFYQPPFCYLKSTVNQTVGGIEGEFFIAKKGIKEFE
LDGVEFKLFMLIAERLNFTWMIRKPNHFFRYGRRIGSTWYGGMIGQIYRKEVDIAFTNIWLTQDYYLFSNLTYPWTQISINFLVPRPKPHRGFWALMKPLTPIVWATILVMVI
AQSICIYATAWLRKKVPKRFRNFVATFTELVGRLLGVSSPTRTQGLKLQLQLWHFIGILLVTAYSSNLEARLASEGYEKRIDTLQDFIAANLTWGRQAPAPVFSRYFDESDLYG
KQLVDHFEIESNDSERHKKILKGHYAIIGRIIGKMFFPENTIEEEDFKGYRVMKESTGKFYTTFAVQPWLLEPVNKITLRLREAGLAQYHLRDVIHRRTGRELRQVMIEQDIGS
GAPRVLKLTPLGAGFAILIFGLTFSTIVFYLELKHASRKTFIPKVLHRMKKKSQRLASLKDYTKVNIRR
>AmelIR21a
MLLVTLFLQFIVLASSKRVLYKLHQCENNEANLKSLAEEIVEEIIEQTNCIIFITDSTYQNLIDIKNIKGSSNVSKYEILLRDNEQFSRPRRRIQRILVDGRTVDCNAYIMLISNGY
LTAEFLQYTERERLINTRGLFLLLYDLRLFQLNLYYLWKKIINVVFIRQYNAYKHRSGEISFKERIDLNTVYFPPRKRRLTATKYIDTWYQGKLRYGTNHFTEKTNNLQKKHL
QIAVFEHIPAVTEKSKLYYNKQPNNIIQGLGIEFELIQIISKAMNFKPKYYIQQNIPLKQKDIEGSNQTDTGLISKVIEENAAFYLGDLHYTLQNLNYLDLTIPYNIECLTFLTPESL
TENSWKLLILPFKFYTWIALILTLILGSIVFYFLSLSYKKHISSYKSQNTSIKNETKGLYLFTEIGNSILYTYSMLFQVSLPHLPSPWAVRILIGWWWIYSILVAVAYRASMTATLA
NPVARVTIDTLAQLAKSSMEVGGLNEESKNFFLKSSDLSSQEIGNKFIIIKHEDEAIEKVANGSFCYYENSYFLQYARVKRQIFEKEKKRNETANNRSSKHNLHIMEECIINMPI

ALGMEKNSPLKPKVDILIRRMIEIGLVKKWLNDVMEWPKIMEIRQEAESEKALVNLHKLKGAFFAIIFGYLLAFMILIGEILYWKYIVLKDPKFDKYHLDIFYNSNNNSKI
>AmelIR25a
MRFVEFTIATCWLLGLCGRFVIIYGQRSVASNRESGKTRSVNIFIINDEANKVANQSITTALETIKENYPNHLGNVWSVQVNESDINNTLDRVCNNWDSAVEKGGAEVPDLVI
DTTTAGLAAKISNSFTAALGIPTLSAQYGQVGDLQYWRKLSLDQQDYLIQVMPPTDLIPEVIRQLSIQLNITNAAILYDYNFVMDHKYKSLLLNVPTRHVINETSQQIIEMKR
QLLRLRDLDIVNYFILGNENTISIALEAADALNFTDKKYGWFLLTPDINIWPRCECRDISVLFMKPEFDRRNNSDSVEFSLPKPILLSAFYYDMIRLAVLAMKSALDDGEWPM
EPRHITCDEYNNTNTPERKLNFFGKLKDAYKNMTPTYAGIKWGSRNGEHQAKFVMSVHLVTIKDGVVSNTVDSGSWNASISSPLQLTNNDVMNTTAVKSYRVVTVIHPPFV
MYNEEKNEYYGFCIDLLNEIKKTVGFQYEIRETDDKKYGSLNLDGSWDGMMRELIEKRADIALGSLWVTAERERVVDFTVPYYDLVGLSIMMLKTKTTTSLFKFLTVLENE
VWFCILAAYLFTSVLLWIFDRWSPYSYQNNREKYKNDDEKREFNLRECFWFCMTSLTPQGGGEAPKNLSGRLVAATWWLFGFIIIASYTANLAAFLTVSRLEIPIETLEDLSK
QYKIQYAPVINSSAYIYFKRMANIEWKFYEIWKEMSLNDSLSDVERANLAVWDYPVSDKYTKMLQAMEEAGFPASTEEALRRVRRLDSNNEFAYIEDSTTIKYLTMTNCDLI
QVGEDFSRKPYAIAVQRGSPLKDQFNNAILILLNKRKLEKLKDKWWKKNPNKKDCDAENSQSDGISIHNIGGVFVVIFLGIIFACFTLAFEYWYYRHRTKITKIDRNNAMKN
KITQVKPLRFNLQPAPTHAFQSVHFRPRF
>AmelIR93a
MHYSQVSSVIYCANNTRGYFSLSIEMISVLLLVWWINYGSSYNNFPSLITSNATMAVIIDKGFFSNKDEYQNATKVIQDLITDAVKKEMNLGSISIRVFRDMNVNFKDYTILLS
VATCYLTWRLHEVAQKEELTHFAITDPDCPRIPDTDGITVPSIVPGEELSQIFLDLRMTDILSWNVINILHDDTFGDKATSSNDNVTILLSNANTCSRLVSDRDTISRVLKAISNK
LPNKRMNLISRSIFSLRYGNTGSGRKSSVKKMLNDFHVEQLGHCFLVIATVDMVADVMSVANSLNMVHPGSQWLYVITNSVSGNLINTSFINLLAEGGNVAFMYNATNLDG
FYKIKLKCYIKDLIEALAKALEYSLKNEIELFKRMNEDEFEMIRLTKSKKRAELLKNVRIHLSRNTSASNSVCEQCLLWRFFSSITWGNFFSHDRNMAHLLDIGTWTPIIGVNL
TDVIFPHIVHGFRGINLPIATYHNPPWQIISMSKTGKKLYEGLIFDAINYLSMKLNFTYTVIMPETSQISRSWNTSQFAKLGEKIKEMTMSTTKKVPLEIIDLVRQKKVLLAACA
LTVNECGNTTFNYTVPIFVQTYSFLTAKPSQLSRVLLFASPFTKETWACLAVSIIIMGPILYLIHKYSPYSTKASGLNSSWQCVWYVYGALLQQGGMYLPQNDSARILIGMWW
LVVMVLVATYSGSLVAFLTFPRMDTSILSVEDLIAHKDSISWGFPNGSFLEMYLQNAEEPKYHVLFSRAERHNDTEEERLVERVKEGKHALIDWRSSLRFLMRKDFLLTGSCH
FSLSMDEFLDEPIAMIIPYGSPYLSVINAELHRMLESGLMNKWITEKMPMKDKCWEAPGSNQMVNKRKVNVTDMQGIFFVLFIGITLAFFFLFCEFYCHRRKIAKERKLIHP
FVS
>AmelIR75a
MYVQNIFYLQLIVASYASNIDIIRDYFIFKNVPRVAGFSCGNIENDYQILKLLNEVGIGVSITQFTSIINIPQFLHTTYWNLGIFVDLECLVSDENIVKLFYETSTYYMFDHLHQW
LILEKNMTHILQLLNDNMFSIITDVTIAISKDNDYILYDVYNHCKNYGGLLNITKLGTWTKNNGLQIILETNKFSRRWNYHRMKIKVAGLVVKRPKNQSLIDYLQEENLYEHT
DNWSKFGYAIMKHIKQLFNFTFELIELNHWEKNDSNGPLIAGLKNGIYDLGYFPSILTKERFNYADVILQVWPIRTCFMFLTVPSLKVDMDIIFRPFARNVWYMILILIVAIILG
LWIIFKLEENDSAYGSTILIIIAALCQQGLPFFNNQFSSRIAFLQTMIFGLLVYNYYSAAIVSSRLNAPLDKMNDSLYSLVNSRMKLAAYKDIYFNILLHSSVEEVQYFKKYWEK
IPEKKRYLSIQDGLKKMTTAKFAYHADPMNVYPFIERVFDKQMICQLTEVHLLRPSSLGLWSTRHSQFQEITKIGLIRISTSGIRKREVIRWTYRKPYCDKDKHYVSSITIHETIP
ILLVLCFGIILSIVICFIENIIFHTIRKKQRQIKESEF
>Mmed25a.1

MGTMGNWFWWNYIFLTNFLIVFAQQNQPPTGVRPVNLFIINDEENSLANNSVKNALMGIKDKDKTVLGNVIVVQINGSDPRGSLDKVCAAWDPAVRDGGPGVPDLVLDTT
RSGFGAEAINSFTASIGVPTFSSQFGQKNDLRPWNNLTPDQKNYLVQIMPPADLIPNAIRQLCSQMNITNAAIIFDKGFVMDHKYKSLLLNIPTRHVIVQSKIDTNEILNQLQRL
RDLDIVNFFVLGEEVTLKSYLDAAESKNFTGRKYGWYAFTMNENLDLKCECRNMSVVFFKPILTTSNQQHLSTLTTQGLLPAPWLASAFYYDFVRVGVEAIRSVIKNNKWP
NEPYHITCDEYNGTNTPIRNLDLLRHLQEATTNGFEPTFAGFYWGKSNGEHHAEFDMRIQMVVIENGNIVSADDLGTWKADIESPLNLTDTESKVAKHTAVTSFRIVTVRIAP
FIDINATGHWEGYCIDLINEVQKIMNFEYEIYESPDNKFGTMDESGNWGGMIKELIDKRADIALGTLSVMAERENVVDFTVPYYDLVGISILMKKKKEETSLFNFLTVLNQT
VWLCILGAYFFTSLLMWIFDRWSPYSYQNNKEKYKDDEEKRVFHLKECLWFCMTSLTPQGGGEAPKNLSGRLVAATWWLFGFIIIASYTANLAAYLTVARLEQPIESLDDLS
KQYKVQYSPINPSEAYTYFKRMADIEERFYEIWKDMSLNDSMTEIERAKLAVWDYPVSDKFTKMFQQMHEAGFPKTREEAIGRVRKEIPKYNNTDFAFIGDATEIRYLEMT
TCDLVMIGDEFSRKPYAMAVQQGSPLKDQLNNAILKLLNQRKLETFKSKWWSRNPYKVDCTKKDSQSDGISIQNIGGAFIVIFVGILLACCTLAFEYWYYRYRPRAQAKKL
KNLNSKNGGGNKISSQRIKPTRFNLKPARKAFEDSGTEFRARF

>MmedIR8a

MNDILPEAEKAAGGNDIVKVDVEKVELDRNNVSESLNQACAVLSRGIAIALDMTHTGWDKLREIIANNSIIYLRSDGSIIPFIQATDEFLKKKNATDFALIFENPSDVDPALYY
LLGASTLRLVIIDNLNNASISKIHDMKPQPSYYMIYSSTKGMEKLFQAAIDGNLVTRSGVWNLVFTDMDYKQFRYIRGPDVLDHNIGIFSMNSTVCCTPLEKIICECPPDFKIF
DQYLQVLMKQLVATFVKINSESGPKVRSIKGQCPLVEKPAEEDKIIQVFKDAIIEVTEADPLHTLEYMPKSSIVVHECLIDIEILENGTLNLLAEWTKDAITPVAGQVIAASQPH
FRIGTTASIPWVTLHTDPHTGEVLRDANNNLMAEGYCIDFIRELSKKMKFTYELVVPKDRKFGDKLPDGQWTGLVGDLATRRTDIAIASLTMTSEREEVIDFVAPYFEQSGILI
VMRKPVKETSLFKFMTVLKYQVWLSIVGAIVATAIMIWILDKYSPYSASNNKQLYPYPCREFTLKESFWFALTSFTPQGGGEAPKALSSRVLVAAYWLFVVLMLATFTANLA
AFLTVERMQVPVQSLEQLARQSKINYTVVNNSNAYQYFSNMKNAEDKLYKVWKEITLNSTSDQTEYRVWDYPIKEQYGHIFQTIKTVGTVANASEGFVKVLDSTKAEFAFI
HDSAEIQYQVTKDCNLTEIGEIFAEQPYAVAVQQGSHLQEEISRAILDLQKDRFFENLSAKYWNASLKDKCPTSNENEGITLESLGGVFIATLFGLALAMITLGIEVLYHRRKN
NLNDDKAQEANNKSVKKIPSKLIKPKLHQFKPAPTVAFIGRNRGLRSRVSHISVYPKTFPYKD

>MmedIR25a.2

MYNFHWRNILAFILTQCLIQTLQQNQPQNTVRSVNLFIINDEENIIANNSLNNAITNIKDKDPTVLGNVIVVQINGSDPKPALEKVCAVWDPIVRKGGPGVPDLVIDTTRSYFG
TKTVNIFTSSLGIPKISGRYGQQSDIKHWGNLTADQKNYLIQIMPPVNLIPKAFRQLECEINASNAAIIIDNNYVRDPAFKSLQQNISTRHVIVQAQINNDDIDKQLNRIHDLDIV
NYFVLGRENTLTNYLDVADDKNLTGRKYGIASNQQYLRKLTAQNLLPAPWLASTFYYDFVHISVEAMRSAIEGNLWPKEPRYFTCDEYNGTNTPMRNFDLLSQLRNATTN
GIKPTFTRFHWGRSNGEHHAEFNRRVSMVVIGDGNSIYSDDLGLWNSEIRSPLNLTENFDTGLAHYDSIPIYRIVTVRRPPFIDFNNETNDWEGICIDMLKEMQKFMRFEYKI
YESPDDKFGTMDESGNWDGMIKELMLDNADIALGTISVTAAREYVIDFTIPFYEPVGYSAITKRLLDRTRLFSFVGSLSVKVWSSAIVGFFTSSILIWVFDRWSPYSYRNDPN
GRFSENYVRKFRLIDSFWFVFSSLSPQGGGIPPKNLSGQMVAGVWWLFGFITLAAYSANLAAYLTVSRLQPILKSWDDLKEQFDIQYSPVNPSDAYTYFERLKDIEERFYFV
WKDISLNESLTDRERAALAIWEYPVDDEHTRLLGQMNEYGFPSSTEDGINRVLGLPPYPNDSSYALIAEATTVRYLAMTDCRFLLLEGEFSRKPYAIAVQQGSPLKELFDNAI
LKLLTEDKISELKKKWWDNNPKRQPWCPQRINKSDGMDIRQLGGIFIVLWVGIASAIVTLAVQYWWYRYRSRMRFKKDLENAGSLDNVEPKKFKVKPALRRISRVTNN

>MmedIR64a

MKSRTSVYLLFIIFLALSLSAIEGYDVLAKFTGDYFKKFYVTQIVVFGCWENHISVEFSKSIMLNTHSKLMYVNINDNLNLDKLLKVDYWSLGIVLDLDCQRSHIIFNQFSEQ

NLRHNESYFWLMPTSKEKIPDYFHKLPLNIANEMTLAVRKNNNLQNSGNETNNNNISYILYDVYNPSYRHGGKLNVTYMGHWSLNDRDEGTLTISLTQYKYKRRGNLYGL
VLNASIVVDHPPVPDYNTYIHNPINPHLDTMHRYNYALTLQLRDYYNFTMNLKRGSTWGYLINGTFNGIIGDMMKGLVDFGATPFQYKPERLDAIEYTVQAWMARPCFIFR
HPTTNELSNPFLKPFEMKVWYVIGIFAAVNWIFLYTSVKLEHKLVMKQPVCTLDTYPASEIIMITTSAICQQGLSDGPRFYAGRIVFIFLFIWGFLLYQFYSASIVGSLLAGKPR
WINTLQDLADSNLEVGIEDIAYNYDFFATTTDPVAQQLYREKVAVNKKRKREPYYTIEEGLQRMQKGGFAFHVDVATAYKIIEETFDVNEICDLVEIQLFPPKHTATATARFSPF
KKMVTYGLRQVVEHGMARRLRNVWMHRKPECPESHKDDPVPIMITEFSPALFLMSCGWFVSMCIVIGEKLAMKKQEIGEMKDGDADAKGYEDPEDFDRETASTNSQTSR
KSG
>MmedIR76b
MRGIELMLIGIYALTSNNTIDYTLPGRYDWEENLEEVIKKSFLTSVQKEQEYNEIPKEIKVTSWDESLYSSISEVNGEYKGGGYALKVFDIIAAKLDFKYKIVLPEKPILGDNVT
GVIGMLNQSKADVAVAFIPIIPEYLRFVQFSPILDYLEIAVLLERPLQSAIGSGLLAPFSRTVWICIFVSLALVGPIIYIVTSYRAYLWGRTKKDKYKFIDCVWFAYGAILKQGSAL
TPEADSNRLMFASWWIFITIITSFYTANLTAFLTLSEFTLAFTSVKDIVSQHKTWSAQQHYIVDITINRNDPEELTPLRTSYRQHRGIFYHISNKDNKTEKVASYLTNRRLFIDEIQ
FVENFIKNDYLNRTRQNMVEKKKCFYIKMPNPVFQQNRGFAYPSNSTISKIINRELLHLSESGIIQHIESVNRPMVTYCPLMLGSTERKLDNNDLELTYQVIATGFLFSLISFIV
EVSQHYFRCRVCLCCADSCTCCKTRMKEPVSPSKRPQLPLPPPPTITTVPIKTEFSSFTKPPVIPIYDTIDNFKNSKIHYVNGRDYFVVHETTGERRLVPVRSPSALLFQYNH
>MmedIR75u
MFSAPTKWLILQDIRYDNDSNSSKNYNDQENKLKMFSAPTKWLILQDIRYDNDSNSSKNNDDQENRLKDKLKDFEIFPDSELFISQRFDDDKIKILSIYRPSFYVDLIIEEQAT
WDINNGINFFDSIPTSRRRRNLQLTPLKTCLVLTNPDTLNHLTDYKDKRIDAVTKANYIWMMHLVNQMNATVTYTWRDTWGYPDKNGTWSGMIGLLDRGEIDFGGTATFLI
KQRIGVIEYLQLYTPVGSKFVFRKPPLSYVSNLFTLPFGHTVWYAIGVMSCIVLGFLYITMKWEWKEIEKSPGEQDELKSNPTISDNLLILLSAISQQGLSYEPRTISSRIVTFML
LIAALSLYASYTANIVALLQSTSSSINTLKELINSGLKLGIYDIVYNRYYFGVLDDPIRREFRERFVTNKTSIWITLEDGIEKVRQGLFAFHVDTAAGYQLMQETYEEEEKCGLQ
EIDYMGVLDPMLVIKKKSPYREIFRVGSLWLRETGLQQRDTPRLFTKKPVCVGHTSFISVGTTECYAAFYTITYGAAIAFGFFFLEIILYKCFGNKMTDDEESTECDADIEINTT
EISRQSSESLEATLE
Supplementary Data S6. The amino acid sequences of 120 gustatory receptors (GRs) from ten Hymenoptera species.
>BdioGR43a1
MSDAIDQLITLHGTLCDSVDCVNRAYGTAILAGTISCLIHLIITPYFLYNRIYSRNPNNWLLLFSQMFWLLFHVYGMLLFVQPCHRVVVKSKKTGALVNEALTTNLNLE
VEKQLRIFSIQLLQRPIEFSACGLFYLNRDLMTSIAGSVTTYLVILVQFQ
>BdioGR43a2
MEAWGDVKEKRIARSKSQSSTTEAKAHQQTKSDVFYALTPIYHISKICGLLPVRFAPNKAGRYSGQLDYPQVGYGILLVLALAGAQCWGLYRDLRNGWQNSTRLSSE
TAITVTCSDVFAVISVTLVTILGSSSRWRHVQQGLNKLI
>BdioGR43a3
MDWSTVRAKEFPRRKSEITNADEKSHRAEFDILRNIMPIYHISKICGLLPVKFVLDKTGKYSGLVDYPQVGYGILLVLALTGAQCYGLYRDLRDGWQNSTRLSSESSITV
TVGDVFAVMSVAVVAILGSPHRWYYLQRALRKLVEVDEKLGIPTSSNLRKLSISVISITLIWLVIISILDFISWGVSATTENSDID

>BdioGR43a4
MEAWNNLKTSNRSKVHCFAFIFKYLLEVFAEKSSTQKNQNIKPPESKSHHARSELLRALAPISYLSKICGLLPVKFTLDKDDKFIGQVDSKRVGYGILLALTLAGLQCYG
MHRDLRDGWQNSTRLFSKTLIMVTSCDVFFVVSVTSFSILSSTYRWHHMRKALDKIVEADEKLDVATSPHLRKLSIAMMICTLIYLIIISIVDFVTWGLIVASKGSPVTAD
KGPINYSPLYFMYIVIVCLQMQYAVVLFNLGERFAKLNKVIENLTRTNSIVEYFRSDMGLAAQERRRQISFGSSELTQARPGRANRSMGSTIASEGVIYGNAASETIDQLI
NVHDTLCDSITHVNSAYGVILMMCTLSCLLHLVITLYFLYIEVNAETQRWYIFTRQTFWTIFHLWKMMLFVQPPYSVASKARNTGSLVSQALANNWNPAVQKQLEKF
TLQILHRPVEFTACGMFYLDRGLITSIAGAVTTYLVILVQFQNDEDTPTKELLQNATKLLRNVSGIEGS
>BdioGR43a5
MEIWDFASLIFWLAVKITILYFMCIIPEEISEKENKAKIIFSKIINVLNIQEAQQYNELKVFLLELFQREPGVSVYGLFELNSKSFYHVRFTKYLKLLLRIINKTGNYNFKYTF
LRLLQQLRCM
>BdioGR28b1
MTMLKCNIFGFKSFLILLHYFFVLNGLSTFSITQRKNTATFKLKQRYLFKKSSFSIFYNILLICMSGYVCYVGVYAVYYKVYKTKLFLFQIVDLSHAVFGTVVTASVLLSFCL
QADSLVSLVAKTYNTYTIFVYLDVIHYKKSIVYYVTSVFIIHIISYTLFMISGFFLFRNSFIFLIGFIPNFMITCLNMQHMFIVRVLFSMYKNLNEVIKKNASLIISTNQYQKF
MNVLSVTHKTNVFNLSSFQDLHKNLCDISHDISEFYNFPLLGSVLYIFVSLVVYNYFLLNLILERKTPFGIVEYVHCIMWDLMFVSSAFLLTRNVTNAVNEVNEH
>BdioGR28b2
MEELAREMQFTYTIYVEPGKNNGNCESTKVHDDYICKCTGMMEKIRTYEVDMAITDLTQTESRARCVQFSTSFMNLGITILAKKPQKVPSWYSFLTPFDYKVWLYVG
LSMILIPMLMVALARLCPTEWTNPYPCIDEPEELH
>BdioGR28b3
MEIPHLETRWDFRLKREGCLVNLYPHPATLSKAYVDLVTSLGWKGFTIIYENNEGLVRLQELLKAHGPTEFPITIRQLGEESNIGHGYRPLLKQIKNSAESHIILDCSTDKI
YTVLKQAQEIGMMTDYHSYFITSLDLHTVNLNEFKHGGTNITAFRIVNPDRTQETVQDWIFGEQRYFRKLEIEHNEKNYTFIKTETALMYDAVYLFARALHVLDASQ
QIEIRTLSCESSDTWDHGYSLINYMKNVEMDGLTGAIKFDNQGFRSDFELDIIELNTKDGLKKIGNWNSTKGINFTRSYGEVYTQIVDSLHNKTFIVTTILSAPYCMWK
ESSKKLSGNSQFEGYSVDLIQEIARILKFNYTIRLVPDGRYGSYNKELKEWDGMIKELLDQKADLAIADLTITYDREQAVDFTMPFMNLGISILYRKPVKQPPNLFSFLSP
LSLDVWIYMATAYLGVSVLLFILARFSPYEWENSHMMNNQTSGIENEYTLLNSLWFTIGSLMQQGSDIAPRAISTRMVAGMWWFFTLIMISSYTANLAAFLTVERMDS
PIESAEDLAKQTKIKYGALKGGSTAAFFRDSNFSTYQRMWHFMETAKPPNDVFTKSNVEGVERVVKGKGSYAFLMESTSIEYVIERNCELTQIGGLLDSKGYGIAMPP
NSPYRTAISGAILKLQEEGKLHVLKTKWWKEKHGGGACRDETSKSSSTASELGLANVGGVFVVLMGGMGIACVIAVCEFLWKSRKIAVEERRQRSSEKPMCIGFTDLR
>BdioGR64f1
MHRVDELDDDCNLQQITVCDEVMTMDGNYANNRNDTKRHSPGFGSRMLAIFGSSTSYGKTTRIFKSPKLSKVNAVQPRAAAMQNRRFSPSDDDPECFHRAISRILL
LAQFFGLLPIKGIRAHSVRRFTFKRFSPRAIYSYCTLFAIGVMTSISVLHLFRTLDAKSFSTKGGIADATAGAIFYGNSLLGNIMFLRLCPRWISIQYDWRAMERLLDNNA
GIAKCKRPALRWRFNLIAGIILSLALIEHILSMINNTPMEVFGRNHTLEDFLVVYTNKSHRFIVKNVDYNFTLGLFIFLVSKISTFTWNFTDLFIMVVSTGLAERYKCLNQ
RVTKVLPAQLSATDWQEFRQSYAALSALVKKVDHEISGIILLSFGNNIYFICLQLLNGLTPEGSGNQFINSLYFFGSFIFLIGRTTAVTLLCARINDECKVILPILYNCPSMN

YNLEAQRLQQQIATDDVALTGHRFFSITRNFMLAVAGAIVTYEVVLLQFNVALQRDI
>BdioGR1
MRLWDQYAELSRISRAVSNFYSFSILLVIIVEITTQMSIMYTVFRPVILGGQFLNTSQLVNGFFFYLVSSIPIIMLTIAVTNMISEVRKFQHSNTND
>BdioGR2
MSCIATANALTWYYRITEGFQLKFKHIVGTIMAHCAGVCTINAFSILRKKQIFILNNINQIRINLNEDCNSRESCISRKVKLIVFLHMGIWLGYAISAMFFDFYTNLANLI
FNINFFILNAVVLQYCIVIIFIKYLFFKLNHNLLYLLRNIYTNDASPFVTGFDKDLVHLWNKYTKLRKISRTVAKFYSFNILFVIIETLFELISVTYTVTIPMIFGGQRLKIAQY
VNLIFFLLAIVTPLILLTTEIANTKNEVNFSL
>BdioGR3
MTCMILANGLVWYYRIRFTPYFSLKSHPGNAVSTTAAICYILSTFCIKREKQIFILNSMIQIKDNLDKKGNSTKQNTDVNLKIMLFVYWCVWFGLTISLFFFDVFKALTIF
VFNMNHFIISAVALQYFVVVTFIQDLFIKLNENLLLTLKHTCTHNTFVLIFKTNDDISLLWNQYGELCKVSRVISNFYSSSILLVIIEIFSNAISAAYFIIKSTFREDQPLNIIRY
SNDICFVLALIIPLILLTRSIDNAVDEVRKFCIIIYINILLIW
>BdioGR4
MTGAINTTGVVEQLEDKQDKYLKYLFYCFKVCGIAPYNLNIEKVQNVNITKWMFAYSQRSIIYNIIVMSALIVMSSTVLYFYATSKLYEKIMYFAVDALGLLNALFIVGM
SSMKCQTQINMWNKINGMRKSLYLEHRSYEKNNIVPRKIKIIFLVHLLPWLVWTLSKFSSNDNSIVIYFICDIGHTISTIMIMQYCFTVVLIKNLFIKLNENLIYDLKLTSHI
IQERSVTKTYNDLSRLWNQYVELSKLYNDVSNYYSSNISLITINAIIMVPAAAIYYVIAKPIICTKTDITFFDITLAIFFALTYVCPCVLLTTSVCTAVNEVGSIFCLKSDVEFIE
NLFTLEICIFLRVKKHVKLFGTI
>BdioGR5
MIIHYCVVVEFIKYLFIHLNENLNCSLRHARCLRGDLSAAETNNDLLRISRLWDHYFDLSKLSGKVSNFYSLNLSLITINAIMVPASAIYYVIAKPLVCKDTYTTAFDLAV
AISFAFSYVCAYMHLTVSVCTAINESKRTREIIWNHLRKSHAPVIVEKLTQFSNYLLQENVQFSVFYVFSLNGSLLVSVGIITIKIEYICDKFIFVVFSYFYRW
>BdioGR6
MMRRVEKIIQNLKVDDMFLKWLIYFFKICGIATVDFSTEGCSNRTDRKKWTFTYSHKNIAINSIIMAFMFACSGFTVYVYKISGFYTRLIHFIGDMFEFFAATMVIGIFIIRS
NKETVILNEISNIREKLKDESCRYDERDVFKKTKFILFMHISLWMALTVSILFLSPYQILLYLIINTGSFVSCTILVQYSIIIEFIKHLIVQLNENLSHTLKDANASYSWSSVVKI
NNEFTRLWKIHMDIRNISRDVSSFYSVCILLNISSLLLLSFATLYYDF
>BdioGR7
MYAVELFFLPALLTPLILVTTSASSTLNESKRTREIVNDQLRKTESQIIKDKLTSFSMYLLHDNIKFRVFDLFSLNNSFLLTITGTVATYLFIVL
>BdioGR8
MIEPILLQGKYSSASTYINQVFFLLVTLSPLVFLTASVNATVGESQRTSEIVNDQLRNPLSRVIKDKLHQFSVYLLYDNMQFRVYDLFKLNGSLLVSFTGSVATYLFIVLQF
QD
>BdioGR9

MVKNVVQKYFHNIFLLLVRETKSCRTMSLTKRLENLKNNDSVWFNFLVYYFKIFGLAPVSFRVQKEEHSRRRRWIFAHSRMSIIFSYFVMSLLITWNALMFFKYMKKV
YVQDTIINTSANIFEILCSLCIIMIFCLRKTSGIMVFNKFNGVTELIPFNNQVNSLPKIVIVVFFMNTLIFIGIASTLFFYDFISSMIYSGTLLCHMVLNAMMLQYFVVLKLIE
HLFNVINNNLLAIVSDSNALWEPVTVVEINTKILNLWKAHSKLCVLSRDMSKLYSLPALLVTATEFLSMTVFAYFLAKPFFIDGKYLSGNDYVNWILFTLINLLLFVHLT
LSVSAAINESKKTRVIVNDLLQKPQMQVVKNKVR
>BdioGR10
MVENKLYELWNSYVNLCVLSKDVSHFYSLPTLLNIMYKILSATVSVYFIAKPLVLETIHSPIFYYIHESFYVLACLIYTIFFTMCITATINESRRTRKIVYDFLRMPQTPIMKA
MLSNFSTYLDCDNVKFAVFNLFNLDGTLLISIAGSITTYLVIVLQFQS
>BdioGR11
MTRRWKTFKNSLSCQKSSSHNYLFYYFRACGIFPINVEVRTNVNVRRWAFTCSTSGIFYNLFLLGCISICNFLTFYNYAHHVYGNGRIIFKIADLFQIFSSIFMIIVFSIQQK
REVNLFNQICNIKEALNIGNWNYKPSTLRKKVSYIIFANACITLGLIFTTFIYDFQRKLLYNSLNLCIISRNAITLQYCVVLMGLHNLFHTLNKKLVLILESSALFYALPSSI
ARKRLHDLWESYTNLCELSKNVSHFYSIPMLLNIIYTITTATASVYYIAKPLMLGGSSKRTFHYIHEIFFGVTYLLPLILLTTCVISAVNEVNIIGFYVL
>BdioGR12
MIRILKNTGNMESISSRCLFYYFKLCGVAPVNLNIQTINQGKIDRWTFTYNWVGTLYNLFLTCLIIMCNYSSFLLYYTIDKYNFSFETTVYIIVNVIYVINTACIVLVFCVKR
SATSILFNRLNYMREIIRNSDGWMGNSKVLFKKILTLSVINASIWVVLITTIFLYDDIELLLSLIFCILVQNALTMQYCINLKLIEDLFSIINRN
>BdioGR13
MIKIAKNHRFMESLHLKCLFYYFKVCGLAPVNLNVNAIDNTKVVRWSFVRAWIGIVYNLLLICCIVVWNYFSFYRSYFKNNTIFTFDRTVYIVVDLYSIFNTLCIILIFSFR
QKQLLTLFNKLNDMRETIKFSSDSSYESKRLFHKIMTLLVINIPIWVILIVTIATFDYESALMFSGLGFCIFVRNAMTLQYCMNLKLIEHLLYVLNRNLSTLSVCFSSPHNIE
NIDTVDNKVSQLWEWYSLMCKLTRNISDFYSFPMLLNIQCEFLMTITVNYYILKPIVFGISSSMNYLDYMNSLCFVLTHMCPIILLTTSVSKVINEVRYLFVYSPVCMISIF
FVNLLCTLQSQRTREIVNDYVKEPKARIVKERVNLMI
>BdioGR14
MLDLSTMKNITRKLKFTETIHLKILYYYFRLCGVAPLNVDIQAINSAKANRWTFTRSWKDTLYSLFLLTLIIAWCSTNLNDNYYKQKHLGFDNSVYAVVDTCATINTLCI
VLIFCIQQNKVLAIFSRVNNITEIIKRNNNSTSEHDTLLYKIMILLIINVLSWVFLITTIAMFEYEDVISLAASTFCTFIRNALALQYCISIILISDLLKTLNENLSDLSKYFSLM
PEVTSINRIEINNKFSELWEWYSLINKLSEDIAYFYSLPMLLNIFCDLLMTITIIYYATKPLIFENSDLDVLDYVNSVAFSFIHICPTILLTTSATTAIEKGRRTHELVNNYLKEP
ETQIIKDKLLHFSIYLLHHEIKFIFVGWFIASFGRYLK
>BdioGR15
MLSKESVFAKYLYYYFKIFGIMPVSLNVAHKKNDDSLFKHSKVGIVYNMLLTFFLILKSYFGLKYVDLAFYMHIKSPFEKTFDDLLNILSLLTVVFIFVKFSFSQRKIAN
>BdioGR16
MSKQPEIIYQIEQFFWVSCHIYVLIILTTWASATVDESRKTRLIINGYLKKRTSKTIEKQLKRFSTSVSYDNVRFKVFELFDLDGSLLMSIVGSVTTYLVIIIQFEMSTH
>BdioGR17

MGKKISYFKNVIDIETLRGICIFYYFKFCGLATMRLKINKSQKIKFEDCSLHESIWGCCYNVIIAVCVVCSSYYGMTHRGKFFEKESHFEEVYYLIIDVIITASVVIILMYFCF
ERAKANDILHRILRVEKLSKIIKGNTDDGKLWRNMRIIITLHLLIWLGIYVSTAFLDRSLYLYE
>BdioGR18
MNESNSLKRLYKLYRGAVVFYYFKICGLATMSYSYENSKKGRCWVMRESIWGRFYNVAMIGVLITMSYFGMKDYTSYNKRYSNFKLIASEVKDLLIAINAIAVLLFFSIK
QKELNDIIREVYDIEQKLTLFMGNRNKNKNNLRRAIGIIFLIHTLIFIGRLSLFCEWYPSLLLHSCVLCAYLTILDSVALKYSILLLLIRRHFKLTNDELSTALTISNRPNSIQI
VTVNDVKHYRELSKLFDIHSRLCKISCKISSYYSMPALFYILYKFSYLTASFYNLLVHVVWNPSNRFDSFDFRVNKMLNSIFPIFYLVVLTKCVTDITIEVHIFKSYIIFILYFC
RKWRILIMSF
>BdioGR19
MQSFSISALKNRQKIKKRRFMMTQDREPIKTVKKEKIMLLNWLFYYFKVSGVAPINFEFKLMNCGKLKRWVFTTNRKNLYYNVLLIFGVGVANYYSLLLILDPSYRYD
KHYFYMQAISMLCTIFILVTICIQQNKLPAILNGIVETKEAILNSCLSSVPDKLFQEIMLIFSMSSILWLATNVTLCLYPYGLFSFQSLLLSTSDAIQSALALQYIVALKFFKYL
LKIINDRFISQSNNAMIADRMSSVSLAKTELEIHNFLRLRNSFSSMCKISQELSDYYSFLLLFFLVAKFVIFTIQLYFIVELILNQEVLIAIIAFFICTFYILDCAFFMVLLAASV
SQIANERGKTCEIINIYLEHSRSPKMKEKV
>BdioGR20
MMLLSTTKECIFVNFIYDFFKIAGVVPFKLHINVNENKKIQKLIFQHSRTGIIYNLLLVILALVTNFIGFRVSLERYDFGRRSKFERIIDTIHEIFATFPCCYILITICLQQNKVI
KLDEQVLDKTSSVSCGILKIISINFITWILVLVTSRSEKIFEIIIYYIGLYFCSSIINHIILVYSVLWIIVKQLFNIMNQNFIKLAEKLSLPRGIEIIE
>BdioGR21
MIPTAIMWCKFGFRITNMYICDAIITGTMLQYSLLLILVRLLFKSVNNKFGKIGSHQEVECILVKCMRTVDIVNLRNKYSSIRDVCEQIERFYSMPMLLASSYVFISLIVYS
YGTLAPFFSGDNQLETKNVLYYVSRIIHGLTALCILTSTVSAVVDESGRTGKVVNTMIWKSTDDQEITKLSQFTDFLLHQNLRFTAAGLFPIDGTLVMSIIASITTYLVILIN
MQE
>BdioGR22
MLTRRVSAVVHESKRTTNIVNDYLLMVASDDVKNKLGEFSTYLLHTGIKFSAFDLFPLDGSLMISVRMEYFFVKLIML
>BdioGR23
MTLFYVVTYVCEIIVNGAIFQYSITLNLIKRLFVTNNDHLGDIAFLRNLAKDRFLRLHKQHCVLIDICEDLNRFYSIPVLSSISHIFLTLVLSSYYICKPFLYHKINLNIALLL
HCLAAIIHD
>BdioGR24
MAKHSTPPCIKFIFHLFKIFGVAPISFDSNFRFSPSKIGIAYNVSLIILVLINTYFAVNASYFSNFAARVQLEKISDTLHTLILIFTALFILIIYSVRQKEIANIANELRRLIDVSST
LCCKKTYDEKS
>BdioGR25
MNFTIYLFYHFKIFGIATISYRISYKNKTVHRLYFNRSKTDILYNIFLAIVLLVMNYFSIKFFYEFDVKQGLENFDRLFIRLVDVLNLSSAVFISFIYCTRQERVVIIANKIDRIR

EILFANNNSSNSLKCINYITKVFVINNAMWCLLLFTTTLYGIQYFPYFFGQLSSKLTITALVVQYTAILKILEEFYEIINNDLSKVATKCAFTTLNDTQDKVSRILQLREVHS
QLQ
>BdioGR26
MKSINDLVVKMDNQYYDYYGKIRRSLKIIFCIKGLIWFPIIATIDMTQLDISLSYVYIQACNLTILCAMLQYSITLNVLRHILGAVNTNFLNITK
>BdioGR27
MIHFDFFIIIFRSENITAEAQKTGQMIREIDDPRKNIIDEIHQFSMQMMHHPLNFTASGLYVLNFNFVQAVRIIIGFIPNSTIYLRLFFSSLLDQSHFT
>BdioGR28
MNWIAFRDASEAITLTIIFDVPLLINPMVEVNFSIAVSTLGKRFERLNALVQSVTDTPPASISPRDAQKYENILNSDQKKVAVQPISHQQNRSNLELLLKVTRQLHLDLC
GISRKVNDTCSKQMSMQMAATFLLLTGFSYSLYLLYNEPNIPLQNKIQHYVSLAVWILTSIFRMIQVVRISVNVTTEAQKTSQIAHEIQVPRSKNKLIDEIHQLSLQIMQH
PLYFTASGLIVLDFGYVRGFVGSVTTYLMILIQNQPDMIKAANTLVEGATDNGTISTTTTKST
>BdioGR29
MFLIFILSSCVNYRFEELNRFLKNIIPDLTNFLEFSFQNPISDIRKLNSQIVNPTHGNYVRTIEGVRQIHLQLKKLCQHASRMYGLQILMSILVAFTVLIGDMYMLYVTLVE
VHVTRDVIFKSITAAVIWGSYYIVKMMKFSSVCSYCVYQSVKTGDIINKLYDHTDPETQNEIREFNVQLIQSPLKFTAYGFFDLNYTLIQGMISSITTYLMILIQLDKLPLVT
VLKRNSVKILNNRTVNSN
>BdioGR30
MAINCNYIAYIMINIILISWFCDSLSKESMVTGYLISELYDEPYINENTK
>BdioGR31
MEKSYSKEIRKVALPIFASNWIFGIGIIEYPLGRPRRIFSCIYISTLLGIYTFASIYSFPYLKEFGLRMAANSIITKSFFGSHVFLVLSSILLGWHRTEVGIVIEIIIDFMYVFLELTG
ICIM
>BdioGR32
MAKAGNASQKCILPVFALNWIFGMGIIEYPAGNQRKTISIIYTALCFIVYASLAAYAYPYIIQMTREFEANQNVIRAIFFFPILLTIFTIFSGWVRNQGMHSILHQTECVDA
LLEKMSAVPRCTKVFRINWYDHVKSFILVFSVMSANVLMAILNDKHVKFQQIIALFVINHPIFIMFIVDSTFINTVKCAHRKFVILNSLLNNMLTCTTDCPQHMKIIQR
HFRSDGNLNEKMDNITLDNPANIIQTAKMIHSTLARVCQTADETFGLHNLLSIISSIIAITASLYHIYVLILYTHIPKADLYRILINTAIWLVYCFIKTYIITSCCSAIAEESKTS
GDIISKIYDEDYINSEAQLQIKDFCYQLTESSVTFTARGFVTIDFTMIHWIIGSVTTYLMISSQFGNFMAPT
>BdioGR33
MSEVHHRRRQVRRSVFGGRGSESPLYELVSPVLYVVRVFGLAPYAYPRGNRPPRLQASRAYCLYSLLWAGIYSWIVVTTMMRFGGLDRDKPVLGVTENGKLILNYLTSL
AELALTVGHREQFVHVWNGIQDFDESFQLGYELPRPPMLRRTRTCVWLSLAVSALAWTAINQLGMQAFGEPYVQNVSYMLTYVGTYVATLKFVGLVALLGQRFAYL
NRLLAQRRLARDPQADALAKAEEKTEDRNVVQQAAGHEREAERAVLVLALPVPAQPLLPRGQQHVLLHHLDDRRPELPQGLQRRAVSVYLAAGLPAAAAPPPRL
LPLYQPRGQPHGKRAAGLAKTHRAPEQTTRFQVDSALFEQETQVHRGRLLRRQHARSHHDLWTSHHLPSHSTADTRQRLLARLSAYSYHPSHVLVNVVNKIVPK

>BdioGR34
MVSLKDIKPLLYAARFFGCGPHVVSERDVLLSPVGLVYSGLWALGFVCSCGYGLYLIARGRESEPKIVALAAGRTLLAYVCFFSDGIVSVRRNERLRAALLQLRSFDAAV
GYGIARPSTWRHSCASWMAVATVFLFWLGVGYITYKCDMTSPLFNAIIYVVANAAISMQLIKFAGLLILLHQRFRYLRILLQPEGGIALTTGMAR
>BdioGR35
MSQLNKKMYYSNKLFLVWHEKKNSFIRYHLLVRKVGEQAGISISLGNSDLKSFAGGIALATGMARVRKGISSAVQRQEMQLQDIRWLHCTLSNAADTVNSMYSMQL
LFWLATMWLNCLSRIYAISENLAGTIGILPKIRESLLVTAYIVNMLIITTACHFTAHEANTIGKAVFKPQSALARKRHSFEQTVEIGTYFNLRELHFSAAGGFIYVDLPLLL
SIAGTMTTYLVVLHNNT
>BdioGR36
MDALGLLFQYCSVVCSWLTVIFRHDKLKSILCEFDNAKELAKALSVTLYENEFKTLLYIILRLSTINILYLAIFLTDHYSLSLYKKFQEQASVWVWFNVPKIVLYNVGAFF
LELMLILKQNFRVINKVLIHSFSSKTTEFRSFSTTSNPIQKLQKIGQLYESSSDLLETVTHFFSLSILLSIIATFIHSFLDIYTVYQYVTNKRTWEVGDFGTYSLNIIWMTTKFLT
LYFICGVPDSTCTEANKIIFNFDLLINNYCHMKESRDAMQKLTLQFHQKRTRVSVYGLVSLDYHLFKNIMGTIIMFLVFMFQLDDLVD
>BdioGR37
MERKKMLLRWLPLLMLLNIVDGDDAGFGYGGGGSGTSPGGIQRTGSTIKIGKTDRSSGQSTGRGSSIIKIGEGNKFLNSTRVVRPVLTTSMTSTSIPLAYASVQPDASSEF
TNGTKMLKVGLAVPYKSFGYREYTKAVSRVVTALQKSTKRPNLGLFQHYDIFVKVAMQELTPSPMNILNSLCKEFLSLNVSAILYLMNYEQYGRSTASTQYFLQLAGY
LGIPVIAWNADNSGLERRASQSSLHLQLAPSIEHQAAAMLSILERYKWHQFSVVTSQIAGHDDFVQAVRERIGDMQERFKFTMLNAVTVTKSQDLKDLVNVESRVML
LYSTKEEANNIFRAAAEFKITGENYVWVVTQSVIQNIQPGHHFPVGMIGVHFDTSSTSIVNEIATAIKVYAYGVEDFVNDRRNYGYSLNTQLSCEDLSSESRWSTGEYFF
KYLKNVSVEAEHYGKPPVEFTQDGVLKSAELKIMNLRPGPSMQLVWEEIGTWKSWEKDGLDIKDIVWPGNTHTPPPGVPEKFHVKITFLEEPPYINLAPPDPVTGKC
LVERGVHCRVAPKDPDPEAEIQAQGGRNGTAFQCCSGFCIDLLQKFSEEMGFTYELVRVEDGKWGTLENGKWNGLMAELVNRKTDMVMTSLKINSEREAVVDFTV
PFMETGSAIVVAKRTGIISPTAFLASSPSSPPSTSSSPPSPLPKRSRPRTSPAAPQVAEAPPFFPLSRLLARLGGALPGGRARRLAARIYSKVHDERLGDVRRCVLGHLHGQ
FGGFYDHARGVFRLHGSGRS
>BdioGR38
MHSMRKPKWGKKTSSMTFDEYKQRQFQLQVMNIQAGRETVVGEWNGKELVIDRNEKEMNNTLTEAAQNKIFKVTTRVGAPYIMLAEGETKGRQIGDKKYHGYCI
DLISKIEEFLKIKCEFDIVADGEYGSLNPQTHQWSGLVKQLLELKADFAICDLTITSERQSAVDFTAPFMNLGISILFSKPSKEVPKLFAFMDPLSTEVWMYMATAYLIVSL
MLFFQARIAPGEWVNPHPCNPNPDELENNFTLMNSMWLTMGSLMQQGSDILPRTPSIRMVAGIWWFFVLIMVSSYTANLAAFLTAVKMEDSINDVEDLAKQTKISY
GAVKGGSTYSFFKNSNTSLYQRIFNSMTDTKPSVFTSDNDEGVDRVKKGKRKYAFFMESTTIEYQIERHCELQMVGTLLDNKGYGIAMPPNSPYRTMISTAILHLQEKG
DLQQLKQKWWKEMGGGKCNDDSDEPTNSNELGMPHVGGVFLVLMLGCVISTMIAVMEFLWNIRKVAIDEKITLLEAFIKEIKFVINIWAVTKPVKIEKSSKSASSQNR
SSSKSESVSISPAHSLHRTDMTYAVNTNQIH
>BdioGR39
MYDSVQVFAVGLRTLEQSHALRPMNISCELEHPWDGGLSLINYINTVDRKGISGPIGFKEGRRIQFKLDLLKLRQQSLVKVGEWRPGSGVNITDKSAFFEPGATNVTLV

VITILETPYVMLRSKGNFSGNDRYEGFCIDLLKEIAHMVGFAYRIELVPDGKYGVYDYETGEWNGIVRQLMDKKADLAVGSMTINYARESVIDFTKPFMNLGISILFKV
PTSHPARLFSFMNPLAIEIWLYVLAAYILVSVTMFVVARFSPYEWNNPHPCHSQNSDVVENQFSLANSFWFTIGTLMQQGSDLNPKATSTRIVSGVWWFFTLIIISSYTA
NLAAFLTVERMITPIENAEDLASQTDISYGTLESGSTMTFFRDSMIETYKKMWRFMENRKPSVFVPTYEEGIQRVLQGNYAFLMESTMLDYIVQRDCNLTQIGGLLDSK
GYGIATPMGSPWRDKISLAILELQEKGEIQILYDKWWKSPSDTCMKNDKDKGSKANALGVDNIGGIFVVLLCGLTFAVLIAIFEFCYNSKRSVPVERVSIYIDRLNGIDC
CRYRESMLFAWL
>BdioGR40
MVSTYTANLAAFLTIEKPVKLLNGIDDLFNQDKIKFGAKINGSTYSYFKSSAKYYPLYEKMSDPEWQKEYMVAENEDGVQMVKRNDTDYVFFMESSSIDYEVKRNCE
LEKVGDLIDHKIYGIAYAKSENEKKKLIFLYRIS
>CvesGR1
MLSPKGSYSNTRRSVDYKSACRKVWPFLVTEDNNMSNSRDDRALHHYTVNRSVPVKNFLA
PEVSGKRNTPDENNESFHKAIGSILAVAQFFGVLPLYGIRAPSSQKLSFKLNSLKTFYSI
SLMIALIFMAVISIIHMVRTLNSDAFQIHGGIAAATAGAVFYGNSLFGLGLFMWLSPRWI
SLQQQWKSMELQLDRCKQSRPRLRWKFRAITITIMTLALIEHVLSILVNLPDNREPESSN
NTWKDSLRIYSQKSHAFILSSLNYNVALGIFLFTISKIATFTWNFTDVFVMLVSTGLAER
YKSLNKYLLKTSSIKNSTIDWGEFRENYATLSSLVKRTDSNMSPVILLSFVNNLYFICLQ
LLNGLSTEDMSMLNTVYFFFSFAFLLSRTIAVTLFTARINDQSKVALPILYNSTAANYTV
EMQRLQFQLTTDEVALTGLKFFYITRNFMLAVAGAIVTYEVVLLQFNVAMKK
>CvesGR2
MNKVTKSDLYQGLFPIYHLSKVLGLLPVRFVRQRSDRYLGSIYIIDIVYGICLLVLFSVA
EAWGLWRDLRDGWENSTRLKHQTALNITIGDVTAVALLAAAGVLGAPFRWKHIREIMARL
IHADERLGYITPKKTQRFAIILSISTMVFLIILASLDIYSWDLQTKMKRKMPDKGPINYS
PLYFFYLQALFTEIQYTIATYNLYERFIRLNKNLEHLLKNSKTYLRKDIDLVSELSDKDK
FPMTILKSEASGGSNKHDRRLFRTPKISGWTAEERESRDVMDTVIQLITIHASLCDVNKL
INKAFGLPMLIVALTALFHLIITPYFLLMEASSDRETFFIIVQCLWCILHVYRIVVVVQP
CYATTNESQKTAVLASQLLTYSWQPEIRKQLEIFSLQILHRPLHFTACGLFSLDRALVTS
MASAVTTYLVILLQFQKADDTKDSTNVLRNATLLLKNVSSTGIKLIT
>CvesGR3
MIRPADKSGQLALVVAEIKQKGQPSEPAFDTWNLPKKKSEKMRNSDCLHVALRPVITLAQ
CFGLFPVNGIRAPDTSGLQFTWRSLKILYCVLALFMSMSMTICSFIRIISTKFHTTKMTT

LVFSVSSCLTNIIFLKLARKWPEFAASWEKVEQEIKVRYRQPPKYSLIKRFKIVTVIIIT
LAFCEHALSLASGYISARECADLYDNSDVTAVYFKTQFPQVFTKTSYALWKGIVVQFTQV
LTTFSWNFMDLFLILICTALTHHFHQLNERLYNVKNKTMPEWWWAEARSDYNDLANLTRQ
VDSYVADVVLLSFGTDLYFICIQLMFSFDRMTSLMRTIYLCYSFGFLLGRTTAVSLTAAS
VHDESLLPAPILYGVHASSYSTEVVRFLTQVTTDNIGLTGMKFFSITRSFVLTVAGTIVT
YELVLVQFNNVQQTSHLNMTNVCEVK
>CvesGR4
MYQNAYKKYNQNLIISKFYFFKILGLSPWSLNLSAVLPKNRHLKITLCKFSNIGSLYNIF
LTIFVTTSGFYAFHHRSLIQGEHDALLTVSTVRLMEYFAILKSSVVFLIYTLRQKKMISM
INGINNVDYRLEKYGWNISGDDVIINMIFVINLFLCVFIVALEWYILSFSEALMRCFPTT
LFCWFLVQYTIIVDVINKRFKFINLCIAKLGSLKENSELPQRVFITKLSFLHESVIFNVI
NLKRAYNNLCEICRVVADFYGLMFLGIILHNGAVTIFILYFTLLRFFKSKDIVFIKLISH
GVCVLWMMFQIVVFTTYVDKTINQSKKTANIINAVIRQNRMDDKVEKELLKFLRDLSFRK
IKFTAYEIISLDRSILATLAGTVATYLIILIQFRVSAPPESLTSTQYNPTTMASTHTKEY
IDENMEYAHKI
>CvesGR5
MIMPYGTVQEAAFERQQKLFYKRENSYKDSFNSLRNKLKFYSLLLVFKVLGLLPWKVNVN
KILAAQNQKNNHENLFEVSKFGYFYNILLIIIMTTYCATLITLGLDKDGYVDSDLVNKSE
VILMLYGTLVLVVIWLNHILQMKKIVNIVNGLFNINHNLKSCNKYTFKSDCRLYTIFIIN
FFLTSSSLVPQFLINDPYSAVMWIALPVISSWVLIQYSLVLEIILQLFKLINEIILQLGE
INIENYYRTSLRSKILLRESIIEDIIRVDCAIMELCDICDQIADFYAIPTLMIILYLIAM
TTFNAYYIFVMRILLHELESLKFRVIICVIICVMFLTAAYNLVVFTSNVTRITREFSKTS
RYISMLTKRCTMNSTTKEALLDCSRDISQRNIIFSAYGVITLDTSLLQMISGAMVTYLII
LVQFHK
>CvesGR6
MLFIRLMSIQMISRVIMWKPKNFLQALLPIYILNFLFCHGAVEFLNVDKSKTKYLFLYTV
ITLVIHLSSYMYVLFNYFDPHWASGYLISYFINTNITFFVMIINIVVGWAFKTKSAMINT
RLIEADVMLHTLGVPLNFKKEFFFSIKIAVFWIVYLLFINISMVSIYREYDLFLRTFVAI
VIQHAYYVNLIIDLSFYLLINHMKVRFKNINKILIDILQLQSNNSMHKEETDYKWAISRK

ISFEKDFTEIIRKLKDIHLELGILCRELMKVYGIPIVFTMIRAFTNTTSFLFLIFVILKD
QKMIMPDKILFLSNFTIWLIVFICKVISINYICAGTINEWQETGSTIHKLECNSKDSNFQ
GEIQKFSIQMLQNPLKFSPCGFFDLGYYFLRDFFGSVSTQIFLLIQTYPIHNSATKQ
>CvesGR7
MKDVSSNNLSNKMFNNNRKSFLGSYKIIQHISSKIIGLAPWTLKVSSTFSDREQIPNRNI
TYSSSHVGTVYNIILFLGIASFGIYRMIANALSQRFNDSLLVITVYQLLFVTLINASFIP
LIYVIRQKIMIKAINNLKTVDFLLNQCADYTLESDYTNDVIYAFNMLMNFGLITVDLFNS
EVVSVLFESIPSIISSGVIIQYAIQLNKMNRRFSSINSAFSKLEDHNLKSGLNYVPSVTQ
VVQSRALIIHAIEITKKAYIELCEMCEMITDFYGIPILIAIFSFSVRALFTVYISILSIV
QIHLSTMVQWYLLAIRIIFAIFLFTTLTCSVTTITKQNKRLALTINHQTDQPEIDEKVEK
SLLKFASDLRHLKIEVTACNIIPLDRTLLAIVTGTIATYLVIGVQFALSSRPTKNN
>CvesGR8
MELINLKLKNKFFCDQYRSGYNRYFIFVQHIFYKIIGLSPWSLDMSNVILKKPPNDNDQI
IVCKLSYLGSCYNLLLFLFISSTNFYILYNSTSPYFLYDSILTPTVRAKLKFIAVLCITF
IPLIYICRQKLMIGVINRLKNVDIKLRNCQSYKARRRNCYTHFIFIMNFLSFISHIVIVF
FYFSKSSVIVHLIILFPDVISSWMIIQFTILLQKIFNRFKSINFAISKLDIRNKFNTSDK
NVSLLTESAIFEIRTIKFAFIELCEICQDSVDFYGLPILISIIHYMVISIIRLYFVILTL
LQKKEPNNMIYVCAGIVNVSIIFLLLVLTSIVTKTTKESNKTGKVINLLMDQCSIDQKIE
KQLAKFSSDILHLKIEFTAYDVIPLDRRLLAMIVGTVATYLVIVIQFNFGTASN
>CvesGR9
MELLNLKEKFNLYQYNLVCRRYFIIIQYVFYKLIGLSPWVVDTSEILSKSLNHIGEINLC
EISYFGSYYNLCIFSFIGLSNIYFELNSSLPHAQSDSILTPAFRARLKFIAILCINFIPL
IYVCRQKILITVINRLKTTDQKLKKCANYLSNKNNKHIYFIFMVKFLCSATSTSILLHHS
NFSFVTLLLRIYPDLIGGWVMIQFSTLLKVINKRFKSINTTITKLDNIVNDRNQIKVLSE
KEVSFTHETEIIKHAYVELCEVCDNVVDFYGLPLLISIIHYLTINIIRLYFIILSVLSIA
SYDRVIYICNGINFSLASFIFLTLTSSVTATMKESKKTLKVISLLVNRCPVDSKIEKQLT
KFSSDISHFQVEFTACDVIPLDRRLLLMIVGTIASYLVIVVQFRINSPSN
>CvesGR10
MQKKLFCQRANAQKSYFKLSCERISIYVSLFVFKVLGLFPWKFNVDTILKDENQDNNKKN

LLEIWKYGYLYNILMIIVSPTFCAMTINLGLWQENYVESNLVDKIEVSLMSYGVFLLVVV
WVNYIWQQRNFVNNSNNLYSIYKNFEIYHRHIENYCYLFIIFVVHFFLCLFILFTGFFAN
DAFSGLLWFVPFFTSSWILMQYLIILDVIFQLFKRINHIILQLGNINLENYYRAALPSII
PLREFAIKDIVCVNHATMQLCDICDQVADFYAIPMLLIIIYFITTTTFNGYYGIVSLLFQ
KVSGLATTYWIIGAWFFLVAFDLVVFTLHIIKITREFSKIPHHICLLLHRCSMNLTTKEI
VLDFLRDISHRNVNFTAYGVITLNGFLLQTIFGTMVTYLIILVQFHN
>CvesGR11
MQQNMMHKRVNSHQYIFDSLNRCMTIQGLLFVFKVLGLSPWKVNKYKISDKNKDNQRNLV
ESSNYGCVYNILLIIILSAICATVIELGLWKEEYMESDLVNKIDLCLMIYGISLIVIVWL
NYVLLQKKIVNIVNKLYSIYDNLMICNRYNFKSDYRLYIFPLLNFFLCGSLVIIDMVKSG
IYITIMWSTPLIISSWVLMQYTLLLNIIFQLLKCMSMKILELGDIDMENYYRTVLRSKIS
LREFAIKDINFLKYAIMQLCEICDQIADFYAIPLLMIVVYSITRAIFDIYYLIVSLFLLD
ESDLIKIYFFVGILCLIISFNLIVFTSNITRITREFSKIPQYVSLLLNRCAMNSTTKEAL
LDFLRDTSNRDINFTTYGIITLNGTLLQTIFGTMVTYLIILVQFRN
>CvesGR12
MYQNLYSSIHYRNLVIAQHIFFKILGLSPWALNFPEKLPKNRDGKIKFCKFSYLGSIYNI
LLVTLAISFATFAFYQRYTVLPQTDALMTNSTLTTIQLLSLLQMSFIFTIYTVRQERMIS
ALNKIFNVDLILQKYTYSKNYNSWKSDNVIINLFFILSLLISIFFLSVGLYLPSFLITLL
RVTPWVLFFWVIAHYTILLDTVNKRLKLINSHIMDLGPAEVDLEKSRPLFFIKPSILYES
VLQDVIKIKDTYKELCEVCEDIACFYGELVLVVILCIGGGVTVVSYFIILSFFKQVEILP
VMFVNDTVFISWATLQFIVFTSYVSLVLKESERTPIIINSLMSQCVVHDKVEKELLNFLR
ELAFRKIKFTACEIIPIDRSFLATAAGTVATYLIILIQFRMSTPSE
>CvesGR13
MKLNHLNEFNEVPTSIHHYSAYKLRFICFSNIWFKILGLSPWTIDTRMLRKNVSAEDKNF
KSKLSFWGTFYNVLLVIFMLITDIWIIYSLSLLENSTLTLETVTVKLSFVSIICASIIPI
IYVFRQKLLVNMNNRFEGVNRILSDCSDYSADDSNKNYLIFTVTSLLTIIIIILRVLYYL
SSAKVFVISLPYFISSWLIVQFVMFLNMIKVRITSVNNTLSQLGTSDNKILLSRESVLSD
IFIIKRIYVEICEISDGIMSFFGLPILIVIIVFSIRSIFNLYYIILILIHIQEIDLRLYE
YGFFLLRNVFLFLMMTSSATEIIKQNRKTARIINLLIDRYLLDKKIKNKLIKFANELWHL

TLNFNACDIIPLDRTLLAALTGTVATYLVIAVQFRISAIPKPAS
>CvesGR14
MYQSVYGSVHQRNLIYTQYIFFKILGLAPWKINIVTKPSKNRIAKTTVCKFSYLGLLYNV
LLTTSVFLMTSYFCHQHNSVDAQVDSLVTPSTMMNLQYFAAIISSSVFLNYTCRQKDMIG
IINEIIKVDRKLQKYAFNSAKNGNIIINLIFLGHLIACSTLLCLEYSFLQSFWLSLLRCW
PAFLYCWVIVQYTIMLDMMEKRFKFINLNILKLGEIKVSSEESEPLFTTKLSFLRESVLY
DVINIKDNYNKLCDICDDFSDFYGLSFLKVIMYHGTSTILILYFMLVAYYESKEFHLVLF
IKNVGFVLWMAFQIIIFTTYVNKTINKSEETPNIINTLMSRCAMHEKVEKELVNFLRELS
FRKVKFTAWGIISIDRSLLATIAGTVATYLIILIQFRTSTPN
>CvesGR15
MIVRNTKIYKRSKIQNTSCYRYYKFLQHMPCKVLGLSPWKIDSNNFWSVSYIGSFYNIQL
SFGLIAFSIIEHLYSKARIYLALAALMVSVVHQSLLFTALSASFIPLFYTIYQRSLIRIN
NRLAKVDGILTKCADYKLKRDYTNEVIFIANLLVIVFLIVIFDACYYDVQHILYGNFPTI
IGGSVMIQFAMLLNRIEKRISSINSTVSKIGTAEDNIIGTQILSLSKKVIMRESLIYNID
NFKCAFNELHEICFDSAYFYGVPILIALFCGSVRVFFTIYLALLTVLKLSKQTVVWHVVG
ARLLWIVSTIIILTSSVTTIQKQHRKLANTITRIVDRKTLNDKVSKKLSDFSNDLRFLEI
ELSACDVIPIGRSLLGVITGTISMYIIIATQLALIQSS
>CvesGR16
MHIRSKIILKLLFYYFKILGFMPYVYCKSGILKRSKSATIYTLILTLGYTKNVMNVMLSR
SSFIRPRETILAVIVDFVSLMLDYIAISMIWIYSITRFNQVQGILKLFLNTLESTSVIMK
IKMTKMYDDILREMFVFVLSLNTLFVITVTTDLIVCYGEIKDFDFPVWTIFNYPRIITLN
YAIVHIYFVKILERQFCLLNKSIGYLPEVFADEDSSMEVVKKRLKPREIDEKLREFDKLD
LDLRNQIKMSNEIFALPLITVLLLQFTHSIFNSYLFMNSLLKKSFSWPILINVSWVLMRM
SLLLFFVDNCHTVCEEANKKVKILHESWAFKRLDGFKEMLKSISLHQLQEPVEIKFHDAL
KLNHELFYKMAGVTTTYLIIMIQLDQQVQHSHDHRI
>CvesGR17
MNYRKIKSVSYRIFVISQYLFFKLIGLSPWSIDASKIITSNGRIESHNFKYSFSYIGVCY
NIIFIVVASSLNIYYFYYITSNRIIDQIMDTFPTAFAFISSFCIVFIVLFITTRQKLFIN
FINNHLQNVDKTLNTCADYDLKYDSTNDITFISNFIFICVIVLSKHFFSKLKLIVFINLP

HFLMTWPLIHYAIFINMIKLRFKSMNSMLSKLGTTESKISRSRVLILDNLDSIKRAYAEI
CKGSDEIVTFYGVPTLIVLIIFSAKTVRSLYFVMIPLISSPKIGTVTYFTGLTLLYPIYV
FIFFTSSVTQVVKENKETVRIINSLRDRFDMDEEIFEKLLKFSSHLSYLTVEFTCCGIIP
LNRTLLVIVFSTIATYLVISVQFYISLSAN
>CvesGR18
MDWSSVLNITTDSPEASPTVNYFSESNTTSKIPNKTIGNLTLECKTELSDGALFEFITNG
ILLNLVGVFGLFGNIISMIILSRPQMKSSINYLLIGLARCDTILIITAVLIHGLPAIYSY
TGLLFDYKFKTYPHIVRYLYPLSCMAQMVTVYLTLTVTIERYVAVCHPLKARSFCTYGRA
RLAVLCIVIVSTLYNLPKFWEVNLEEEIHWRYNVTIYCVGPAILRSNDLYISIYVNWMYF
FFYYAFPFVLLVVFNVAIYRRVRKANRDLQQLSRHQRREINLATMLLCVVIVFIVCNVLP
LVSNVFEMFQVPPVWLVQAGNLLVTINSSINFIVYVIFGRNFKRLFIRLFCSSRFFRAGR
DSPEFQTNDESVVTNMTNIELKNSIRRNHLQRSNTTVVRANNLHTNGSSRRQSVKILTTR
ASNPGPCVYYPAPASSSPVANQHQGSRSNVQNGWSPKNNSEHALN
>CvesGR19
MKNTRNNSDFYRSYKFLQRISCKALGLSPWGVSSKQNNLYNVSYVGTYYNILLIFVLIVL
GVCEVFSGSLSDDYPGKLMPLIMVEVLFITILCASLIPLLYIIRQRKLINIYNRLKFVDK
LLKKCENHKLKYDYTNDVIFIVNLLTTVWLIIILQLCYAEAHRVFFENIPTVIGGGVMIQ
YAMLLNAVENRFRSINLIISKFSLIKSNHDYPQIFSVTQKVLKRESVLYNIDNIKAAYVE
LYEMCHDAADFYGVPILITILCFAERVIYCVYLCVLTILKVQGFHVPWLLMILRLIWIVF
IFLAFTSSVTTIKKQNCKLAKTITLLTDKNTMDEKIMKKLSFFSSDLFHLEINLTACDIL
PLDRTLLAITTGTIAMYLVIAVQFGSSSISH
>CvesGR20
METLLPLFILDCVFCHGVMPYKSVKKLKINHSLLYSVISLILYLILLIFILLKFPTQHES
KMLDGAIKIMRGIYTIIMITNIIISWIFRNRATKLVTHILKADETLKSLGITMNIQKQFC
FVLKVAVSWIVYNVLTCPVVILYDFINLDLITKTVVIVLVYHGFFVNLLIILKFFLLIKH
MRLRFENVNKVLENVLRSHTKNMTQERTNDGWTTSVRKNLGLDFIRNVRRIHLELGILCR
ETTTVYGTPLAFTTIGLFANIIGSLYCQSIRVLSPDLEIPYKIRSIVITLICMVPCSLMF
VSVNYICKDTINEWRKTGIILDKLELKYKETEIQTEIRNFSAQVQRNTLKFSPGGFFDLD
FYFVRDFAAAVATYLVIMLQTYPSKN

>CvesGR21
MISHRPRNLIKESWSSLCMTGIVGLRPIELCKTKLFGYIYVIGVLILYYTLIYFTRDEAD
KVYEYLDKTTIYVAKIRRIEYYVSAVVLSLMLITSAFKFNLGITGVYSYLNEIDKKLKLL
NQKLNYDKNMRDNILNITGIVIFTILIGLTDYSTIKNAASNITMFNLMWFGNQFPQIVNS
IATATFVTIIASCCTRQQIINDLIFKLRNIDKESYNTDVISQEMHNGLKLKLLRKIFEIL
SNTVIIINEAYGFMLILTFITHFIGISAHLYIVYMTFGEQNYTVDLIVEFLWACFYFGKL
IYIISACHSFEREGKRASKLLHECRIDSDHNLLKQETESFARQVRNVDIKITASNLFTVD
YPALWKIVLGQTAYVFFLSQFTSAK
>CvesGR22
MKLVCHRLSEIWQYYFFKLIGLSPCSIDSLKIICGKRTVQNKNYKCCFSYIGIFYNFILI
AVTAVYNTWYIFYFLPTRSDFAQLKKFIVPMAAFAFLGIILVELAYTIRGKLLINFIDRL
RIVDQHLDTCADYNVGCDNTNDWIFFVNLVFASIITIMFQFLEKDLHLKIFICIPVFLTT
WPLIHFTMFINMVKLRFEGINCALSKLGTSKSKLLRTRELVLNNIACIERAYVEICKACD
DIVSFYGFPLLIVILIVSVRFVRLIYTVILNFISSIYYPVGNFSIVLLSQATIISIVLTT
EVTNVIKQNKKTLQVINLLRDRFDMDEEIFKKLLKFSSYLPYLTVEFTCCGIIPLDRSFL
AIATSAIMTYLVVAVQFYIDTSTN
>CvesGR23
MMLIFIFRQRSLISIIHQIKLVTKKIGQCDNFKKELDCTIYLTLMTDCLIISSIMIVEIC
YNFSVTMIVQFILECFFEGGMIIQFVIFLNIITKHFEAINSSISDVDNVKINTAQVHPSY
VINMAVSVPEPDLQIIDSDSVNAYVDLYKICDSLKKFYGLPILIDILNLSVKTLCMLYFT
IWNSIRYMQEDAREIMQMVFMSLRNIFLLIVLTSNVTKTINQSHETTKIINLLMLRPSAN
QEIKKQLIQFSSIISHFKVKFTACDMVPLDQTLLVNICSTITAYLVIIILIRINLSPYIN
>CvesGR24
MRHNYLNSVYFKITIFIKHIYFKLMGLSPWSLNTTKDNSKKQNYVIICVFSYLGSIYNIF
LALGIASLTIIKLYKNFDPIYKDSLMSLSFLASYHLVSMIGISFVILYYTIQQKKLIIVF
SEISKLDKLSYKCGFSSIKNRKFIINLIFSIDVIVRVVLILIEWYRSSLWRSLSNCCTMV
FFGCIVTQYIIILEMIDERFKLINSNIIKLCNKEVESIIPEPSFIALPSQLRRSVLVDVI
SIRKAYNKLCNICNKTADFYGVPLLITLIYNSILLMNALYFGIISYFGMNDPLPIEERIR
GVIVMWFSTQFVILTSYASKIINQSEETPIIVNALMSRCTFDGKIEREVNLFLVNKIAIN

YPFY
>CvesGR25
MLSQVHSQTRRPLTQRILTQMRRLKKTQYSGSNSPLYASICPIVYAARMFGLAPYEFKDN
KLVPSDTYVSFTFFWFIVYTYFIWGYLEGFTSSKQNKKATLTYTESAKTVSNYIVAITDL
IVCMVTRKQIAWIWNKIQDYDQAMRDLGYARTEKKARMLAWFIIGFNIVLWALINNLGMT
AFNESFLYNASYLIVYVGAATAVTKFSGLVLILGQRFKQLNEIAKTNVYKTRWIHADPII
DDKLVDCFHSELTTIGKELNYLYRWSLILWLGNLSFHSVCCSYFVMDWVLQGNIYPDLIN
CLLAWFASTVTQLFLIDYSCHYTSSEVSNLDLEEKNYANCSTSHHFQVKNSQISKYL
>CvesGR26
MAIAHKIKHRNTMLSQVHRQTERPLTQRIFSHIRRTKEKESRGSNSPLYKSICPLIYAVR
IFGLAPYEFEDNKLVPSDSYLSFAFFWFCVYTYVIWRYLVGVLDPSTDMKTTLYHTEFTK
TVGNYFVTVMDFIVCISTRKEISWIWNKIEDFDQAMRDLGYARNEKKTRVVVWFIIGYNI
IIWGIINNLGMAAFHETFFYNVTYLIVYVGAAVAVTKFSGLILILGQRFKQLNEIAKSHA
YKSRWIHADPIIDDKLVDCLHSELTAVGIKLNNIYKFSLIIWLGNLSFHSISSSYFVINW
VMEGKFQMHFINCLLGWFVAIFSQLFFLNYSCHYTSSEVSNLSFIFKKKQQ
>CvesGR27
MIFNIILGWIHRNKNYIINLKTIKIEETISAIGESIDNQRAFRYSLAVSICWIITLVLLN
YNKVAWFNMNIPLIHKIVIIFVTQHPYYINLLVDLIFCLSINHIKIRFKSLNKVLKSFVS
EDNFKNRITSDNLFSTGSRKTSEDNWAIRKSENVDVIKIIQTIKYLHLELGILSGEFMKT
YGLQIILGIIASFGLITHVLLFVYFVVQTKSQSTEDKIIQTVGSIVTVAINSFKMIFVNK
ICSETILEYQKTGELIHKLEIQTEDIKLKKEIQRFSIQILQNPLIFSPCGLLNLSYPFIK
DFAGSVTAQLLILIQTSDENTVLYSSYNETAVNDA
>CvesGR28
MNVTTDFRVIQDHDHGYQQAYPRAVTLTAAVCAILFSIVGVLGNLVTVIALMKYTRLRRH
ATTAFVISLSISDLIFSTVNLPLTASRYLNEAWVLGETLCQIFPLFFYGNVAVSLLSMVA
ITVNRYVLISKSDIYARLYTNRGITIMLIAIWVVSFSLLLPPLTGIWGRLGLDSRTFSCT
ILQKNGKSPKKMLFVIGFVIPCVVIIVSYLCIYWKVRQSRKNLEAHMVNGGMRRNTGFQR
REDSRVTKLMLTIFLCFLLCFLPLMLVNVVDDKVKIPILHVVASILAWASSVINPFIYAG
TNKLYREAYRQVLCPITSSKSNVPVKPTGSHSSKISSHQIT

>CvesGR29
MSVTEILISITWFVETISVSWIQSFYVYKQKTMIKVINRLNYVDRTLNIGVDSYAKKISS
YFIFMLNFLFVSAKIISAIPIGAAPIIVIVYENFPTVMYSWIIVQYTILLGIVEEQFKAL
NSSIKTSNFNQISTSIARLSLIEESAFQEIDHLENLYIELHDICEEINNFYGLPILMSIV
CLNNDLINLISVFILEDSIPSETLEFVFYLAQAIFVLWVIPLTLLLTFVVSKTIDQSQET
GQLIILLSEQCSMNPQVNERLSKFSRDLLFFKVKFSAWDTLPLDRDLLGIVCGTVATFVV
IMVA
>CvesGR30
MVFIKVHLVIFYFLGLIPFSYKKNTNVVDKKKFGPDTITLVPTEIHTCYSLVLIIIIVIG
NSIQIPLFLKERYSGDGNELTETLGLAISVSGNVVMVIMLLLYIVQRQKIIKLGNQLLDL
DVAMKKLHNIYKLESEKMELILICYINTIPCVIITLLTLLIEASFIRATCMYTFHTTVFN
YFVIQYAIVLTLIEKKLKCCNDAFLRMKKSIELQFRVYNTQKNTLEKTSIVEIFALKRCH
TILCKMCSDIADFYSFGFLFIIPYSIVCVILNIYDLLTPFLKIYPTDDVVVRRIGNIAWI
TARLFPILTTAICATKVSKQVSCKIYFSSITLRMLGM
>CvesGR31
MNVSMNINIIVSWFYKTKILMINTRIIEADTTLDKLGVPVHSQKEYCFSKKILFLWIVHL
IFINSVRIIWCSYPLDLQSIILIVFVLQHAYHVNFLIDLIFFVTIKHMKVRFNNVNELLS
DTYKQQINFTSISLADRKWAITRQLKYNTSSADILQSIKRIHLELGLLCDELVKIFQVPI
VLTMITSFVVFVGLLMRLYLNIIDSKTSVNSKVAAMIVIITWFSIYAIKIIAMNYICEET
VKEWKKTGEIVHKIEIETKNPELRNELQKFSIQLIKNPLKFTPCGLFDLSYYFVRDFAGS
VTAYLVLLIQTYSYDS
>CvesGR32
MLRKNYRSNNLNESLTPVLILNLIFGHQIMPHTMDGYKFIQTFFYFILVIIIYGATMGYS
IFSIVTAEWFKYSESVYFAVLVTNLLIMPVNFILGWVHRNELLLINIKSLNVDKKLGKMG
VILDQKVCNFATKIVIYWLVCCVTINFAAMLWTMKALSWYKNFSMVFSLQHPVHVNFLVD
LLFCSLIRNMQVRFKKINEELIKLEYNSHVTNTRVDDNKPAYILQLSKEIHLELEQQCGK
ITRVFGIQLIMSMASLFILMTTMTYNLYVTVFGPNIQDWALRISVIAFWITIHCSKLLFI
NHICSKTVRE
>CvesGR33

MIVLIWILFVLFLDISHIVATEKSDDSSEKIVRSIILQHGHHVNALINTIFCLFVKHMKI
RFKSLNKVLLDIFQLQDNDSKHKKSTEYKWAVSRRINFEKDFIKILHDVKRIHLELGILC
REITKIYGIPIVLSMITAFISISSLILVTFIIIMIPSNPNFDKFVTASNLLLWVAVFSFR
IFIINYNCAECVLEWKKTGEIIHKLEFDSKDEKFQKEIEQFSIQILQNPLKFSPCGLFDL
GYYCIRDFAGAVTAQVILSIQTYRFTPK
>CvesGR34
MILILNQLKDVDERLQKCAFFKIDNSNTIHVIFITNLVISLIFIIIYSFYHKIYVVPVLL
FPILITNWIIFQYSMLLDTITKRFKSINLTLAKLGDIKNYENSTNTLSVTKISLLRGSLF
YDIVNLKLAYIKLCEISQSVSDFLGLTILHGIVYVGQASTIILYSAILVYLEIDHIDFLA
MIDLFSVVWMNIAIFVLTNYVTKTINESTETPIIVNYLIDRCTMDERVEKELDYFLYELS
SQKVEFTAFGIIPLDRSLLATMTGTCVTYLIICIQFRSK
>CvesGR35
MTLTNSFYGLNVACAKIIPSVINSGVLIQYAMLLNKINKRFEGINSIFLKLDYTKINSTQ
ISTITQIVPLRVLILKDINNVKCTYKKLIEACEDTSNFFGAPVLITLISMIGEGLVSTYL
GVLVFINIPSTFIVSWHLIAVRMSWTMFLFIVLTSWITTITEQNSKLAKILTLLTDQSTL
DNKISRNLNKFLNDLWNLKIKITAYDIIPLDRKLLAIVTNLLTTYLIIGVQFVFNVPNTR
SSS
>CvesGR36
MVLNKINKRFEGINLIFLKLDITKINSTQISTITQIVPLRVLVLKDIINIKYAYGKLIEA
CEDISNFYGAPVLISFISLVGKGLVSLYLGILLLMNMNKVTSPWYKYVIRISWIELLFII
LTSKISTITEQNEKLAKILISLTDRSILDDKISRNLDKFLNDLWNLKIKITACNIIPLDR
KLLAIVSNLITTYLVVGVQFLFNVSE
>CvesGR37
MFVYSTTILVLKLLDQNHVESAMVKIIDKILLMSGIFIVLFVWLIYVLQKKTIVTILNRL
YDVNYSLKKCRKYTLESDSFVYITGFCNFALCFSIAILEIIVDSKYTAIMWAIPFIVGTS
VFAQYTILLSIIVQFFKSINKTILKIGNLSSKNEIKVSFVTKILYEDSLIKDIYNINFDF
INLCEISNQIADFYAIPILVLTIYFVPTTTYNLYFMILSFIVSDDKSTIALYFDCGILIF
CIFLSYAALTTNVTRITREVKYLYNLFHHHFGK
>CvesGR38

MRLRFKNINKVLKTFLKSHSKNMTQEGTNDRWATSVQKNLGLDFIRNLRRIHLTLGILCR
KTTTVYGTQLVFTIIGFFVSIVSSLYCIYFRLMKMNFDTCEIMLFIGISMFWLVPFSLSF
LSVNNICKETVNEWRQTGVILDELELESKDTEIQIEIRNFSAQVQRNDLKFSPCGFFDLD
FYFVRDLTGAVATYLVIMLQTYPSKN
>CvesGR39
MYLNAYSSVHQRNLFFGQFIFFKVLGLSPWTLNFSAKASKARNTKFTVYKFSYFGTFYNI
SLATIAISFILYAYHQHNVNQIDSLVTRSITTNLEYLGLIVISLFLVIYTFRQKKIIKIF
NEMNSIDQALQRYAFYSSENGNVIISLIFTFNILICVALVTLGCNLPHPFFKTCLRCGAT
IFYCWVMMQYTMLLDMIQKRFKFINSNIIKLGKIEEDSKLSPAFFLQRLPAQRRSALHDV
INIRHTYHKLCEICEDVSEFY
>CvesGR40
MCKILGAPNQNNNQESLAEVSKYGFLYNILLIIIVPAYLATNQILALAKHGKNNSDLVKT
VDVFLQTYGILVIVGIWLNYVMQQKKIVNIVNGLWRINNNLMGCNRYSCKSECRLYIIPL
INFFLCCCQSGFHILVFGIHRAPLWITPFIISSWVLIECTLLLNIIFQLFESIDAKILDL
GDINDINEENFYQTINLKAFLRESDIKDINCINSSIMQLCEIFDQIGDFYAIPTLLIIIY
FITSTTFALYVTIVKFS
>CvesGR41
MIQYAMVVNKLNKRFDIINTIFLKLNSFKVNATPQVFSVTQVASSRRLVINDIDNLKYAF
LELYEMCQYTADFYGMSVLISILCYAERIIFCVYLSLLPALELGNFETIWQVTAPRLIWI
VFVFLMFTASITTIKKQTSHLAKNITTLRDRCMTDDKIVEKLSQFSIDLLHLKVEFTAYD
IMPLDRTLLAIVTGTITMYLIIAIQFADS
>CvesGR42
MPFVINSYIIMQFTMLLDAIYKRFEMINFAISNLDVNKKVSSVPDRLCATNSSPSHQLII
YEIGSIKCAYKELCEICLEINNFYGLIMVFIILITGTISVIILYFEFFALSGEIEFNIYN
VTDAIWLLWIAYDFVVLTVFVSSTEQESQKTANVINIRKDQFNIDEIVENKLFNFSLDLL
HRRVQFIACGIFPLNRKLLQTIVGTVVTYLIIMVQFHPDVVKQKP
>CvesGR43
MLIRHMCLRFRKINKALRKFIALQSNDLIFIENNESITENRIVLSQTSKRLNHSLQTLKN
IHLELTSLCEKITQIFGVQLIMTIVSSFVLITSLSYDLYLTARDPEVMNSDKIFESFILS

SWMIVNVINFQFINYIAARTVHEWSQTGRIIHNLKSKSQDTDLCLTIQKFSLQMLQNPLK
FSPCGFIDLGFPFVRDFFGTITTYLIILIQMAPNTLNK
>CvesGR44
MSIRMVRSLYIAIVKIIYSSAIDGRIAIVGLSLLQYVFNFVMFTTAVTKVIKQNKNTSRI
LNLLRDRFDMDEKIFKKVLKFSSHLPFLTVDFTCWGIFPLDRTFLTLVSTLLT
>CvesGR45
MVKTSKRIFVFDLKSCVFLSVSQCLWPRAPIYQVLHQFLSTH
>CvesGR46
MIRKFIHITGYLYQEVAILRNRATIVPAKIPYTLTFLSWLLPSIYTLISINYACEKTIQE
WKQTREILARLELESKDPKLQEEIRNFSSQTQRNFLKFSPCGLFDLGSYFIRDFAAAVIA
YLIISIQNF
>CvesGR47
MASLFCCRLLCPLHVYWHTWQSHHNHRTLPYKEDEKRHRCIHNESFNLGSDVLLLQFTIS
NIYILVWLVDAWTTVVSSISFIALWSRCCKSVYGIGNHDQSIRNDWTSSTVSKVITKYFF
LSLVLLLTSNIIHNS
>CvesGR48
MVHNGILVTLVSYFLLVFFIGMIKLDLISCITYGVIIVWVAFQTTVLTSFVTKTIFQIEK
TANVINKLIRQCAMDEKVEKELLNFRCELAFQKVKFTAWGIFSLDRSFLASVSLINL
>NvitGR64f
MRSYHIEPAKTTDQAVQLPVWTITKRSLANTSELRSSKIDSSPKRGRYESLKMDSLPADVSRPSASNSGA
NLVESTRSFHCALRPIIILAQCFAVFPVSGVRSPDATHLKFTWRSFKILYCCLSTLGSIVLMFFSVYRLA
TTTISSNKTIGKLAGNLVFSLTAGITTLLFLKLARQWPSFAVSWENMERELATRHNPRRSSGINLATKFKILSVVVMVFALVEHTLSILSGYVSAVECASLRGDKDIMATYFAL
QFPQMFTDSNYTLWKGLIVQFVNFLSTFSWNFMDLFLILVSVALTDQFRQLNQRLYSIRGKHCFNVKAMPEWWWAEARIDFNRLATMTRRVDSQISDIVLLSFSTNLYFICIQ
LLNSFKPMPNAIQTVYFCFSFGFLLSRTSAVSLYAATVHDESLLPAPILYSVC
SASYSTEVRRFLTQVTTDNISLTGMKFFSITRSLILTVSTFVAGTIVTYELVLVQFNAVQAEHQQSESNI
TKVCETLEITDVKIEDF
>NvitGR2
MYEFAEPYSDAYKLKNIIVCNEVMTAEAKPKGHRNASPRRIHFGIGRKMMSFFRSSKSGRGKPRSQHTPI
FSKIHPAKRPALHSRRQSSNEDEPECFHRAIGNILLMSQFFGILPIRYIRSSSVRNFSFYKFAPRVIYSY

FVLLAISVMTSISFLHLFRTLNANSFQTKGGIADATVGAMFYGNSLLGNLMFLRLCPKWISIQHDWRAMERLIDNNGKWKGPVLRWRFTLISSTILSLALLEHILSMVNNTPS
DVWFGKKNLEDFLIIYTNKSHRFIVRN
AGVDYNFTLGLFIFFISKVSTFTWNFTDLFIMLVSTGLAERYKRLNARILEATPAQLSVTDWHELRECYA
VLSALVKKVDNEISGIILLSFTNNIYFICLQLLNGLSPSTAEHPIINSIYFFGSFIFLIGRTTAVTLLTA
RINDQCKLILPILYNCPSVNYCREAQRLQQQIATDDVALTGHRFFSITRNFMLAVAGAIVTYEVVLLQFN
IALQRDEELNNMAAGSNG
>NvitGR18
MTLATTKISECYDYVFKLRILLEFNIKWLQFMQPPRSLKAMIIFQLDLSVPNVYDLSYQFMSHVKAPSVK
MFLPSNTADKLLFLIKLFIIIRIPTCGKNIHVIVHSAWQTFPAMRRSKCDDLFLKCLFYVLKFLGVAPMA
IDNSPAPEKDSPRYVRFVASKLGVFYNGIIACLTVYPSYMTVRYLTSSEYTKNIELEKVIDEAQSTFAMI
TSTFIIVNICVRQKRAAILANQLSSIHSVIMDLAIDVGDDVRRNTIISYIKKIVFVNLVTTIVWVASTPP
EEYQYLSYFIVMSFYNIIIHAMLLQYSLVLKLLQQLYRSVNADLSSLLKKSSSISDDNNCHLMLKRLKHL
RQIHATLCQISQDVSNFYSLPMLFCVTHVFLTQIIYCYYVVMTLIVWNINEKPILVILNCVTLVTLLAVS
MTILVRDAGVTAVESKSTGEIVSGSIDDCQDHEIERQLNVFSNYLLHKDVHFSVFNLFPLNESLLISIVG
SITTYLVILLEFEVDSTKK
>NvitGR51
MGDIFEDMEKPFLPMLISNWIFGIGIIEYPIRRQLKLLSIVYSTLILVVYAYLVYICHAHIYLTAVAQIK
PIIEMLHYYTNILITISIIIFGWFQTQGLQKCMLKAAQANLLMQQIGIFKNHSNILKNELRKFMAFFLFI
LSIIIINSTVTFYNFVPNYQQIIFIVILQNVPLLYGYIADSSFLNIIGYAYFKFDSLNKLLKSISITKAD
NPMHKIIAKQPFYEKVYPQVYSSIDYKDYTFLIKKIKLAHLRLVKLCREANNLYSFHILLSIAIAFVMII
NKIFNIYVVLNDDDIDEGSKFRTIVRSVNWLIYYIVRNLTSCCLCTTVLNTATKTGDLICELYDEPYITE
NTRAEIRYFNIELVQNKLEFSAYGVVNIDLTLLQVMASTIATYIIIIVQFQKLHFVPNALVGNQTNTYRI
>NvitGR49
MYSGKVSPPASTSKKAPISFRDTCLPILWLNRILSMVVIEVPEGRPWLTLSIIYATAKWVGYGYLLWYTV
KNDESRINSIPIMAAVFQVILYVNVVIAVLSTYLGLANYKKYELYFKKIELADETLEIFGIDPEYSSGFK
DYMKITAIWSLGGALVCATDFAIACYTFSSVPYAIIRILVFEIPMVLNPMVELNFSLMINAIGTRFERLN
ALIQSVAVTPLQSMNSRNFDKYQNILNRNQSKIAVKPNYYYYKNRNNIELLLRTARQLHLDLCATAREVN
DVCSRQMSMQMAAKFLLITGFAYCLYLIYNDPNIPLSGKLQHYVSLGAWIVINIARMIFVVRTSVNVTSE
AQKTSQIAHEIQVSKSQSNLIDEIHQLSLQIMQHPLFFTASGLLVLDFGYVRGFVGSVTTYLMILIQNQS

DMLKAATTLANPNNDTSATTPSP
>NvitGR43
MILRKSTEDIIFFTTCLFYYFKVLGIAPISLYIKSTKKSASQCVVFTRSNRALVYDVVLILNLVTANIYK
ILYLCLRVSSTKIITIEAVTNCLEDFVTCLSAVFILIIICFSREKLSAMVNAISGLTECLDGFGVENPKK
HKLQLEIGMIILVNITTWILVFVTTAVAEFSYLLYDTIMYSNVIVVNALLIQYGVVLKLLRHNFKMLNEN
LLVISQEVPIKIQSPVESNRRVERLSQLRKLHASMCKVSRDVSNYYSYPALACVVCVFYTLIYTCYYLTR
PIVLYDQNLRGDMFVMSLVYGLLLVFSVVILTKSVTATIDESDRTKEIINAGLLRFEDDEKMSKKLNQFS
SYLLHTDVKFKVSKLFSLDDSLLTSMASSIATYLVIVLQFLQK
>NvitGR42
MFLKCLFYAFKLFGIAPMAIKALTSKKNKACHFLFVSSRLGILHNCILLCISISTIYFIIDDTLSRSLFT
DKSNLELVLDTACGICVALTSVVILLKMSINREKAIIIINKLNIIYQRKVESDKKNPSILLVGSVKIIIF
SIFIPTIFAAIALGLEQSSILISCLPFTTYQMTIIQYTLILKLLHYLYQSTNTELQSVLTSKVPISIVQN
RLLGMDTQRVSTKIELLREIHVVLSHLSNEVSGFYALPMFFCISNKFLVLIQYCYYIATVLSHKQDTTGQ
YEIILHCMSFSTVEALSIVYLTRAAGLVVTESKRTGEIVSQLIVDCPNKLVLKQLNGFFSYLLQVQVDFS
VFNLYQINESLLTSITSYITTYMVILLQFKETSCRSGSTEHTENMSTPPSVD
>NvitGR41
MFLKCLFYAFQFLGTAPITIKALGTKKNKACHFLFVSSRLGILHNCILLCISLPTIYLMIEDILARSFLR
KKTNLELVIDGVCAIYVVVPAFAFLLKISISAEKAIMIINKLNVIYQKRKIEFKKESPPVLLLRPVKIIV
FTNVVPLIIGCFVSGQHHISNLLPLPSFLTYQMTFMQYTLILKLLHYLYQSTNTELQSVLRSKVPIFIVH
NRFLGIDSQRISNKIELLREIHVLLCHLSKEVSDFYALPMFFCISNKFLILVQYFYYAAAILSDKQETAT
QYEILILTIWFTIVEALSLVVLARVAGLVVKESRRTGEIVGGLIAECPNKLILKQLNGFFCHLLLVQVDF
NVYNLYQINEPLLTSFTSYITTYIVILLQFKGISCPSDSTDHTENVSTPPSVN
>NvitGR29
MLNYFSIDLLYAKCLYYYFKCVGLATMSVSFKSTVENKKVPYSLFSPSKIGFLPNLVIVLIVIGTHFFSL
KMAFEVDEIETSVKFDRTVESVRLTFGVGVSVFILVFFCAKQEAAIDIANNIKKASVLSANFSTKTVSQK
ELFSVYRATGWIFSAHMVIWFLIYCSTPWSFGLMIYYVSLNIYELVITSTLVQYSILLKIVRQIFRNVNA
NILDIFGDSCAIDFHTVGTIGNNRSEVRFRRKMRKFSQLKDLHISVCDVAASLGQFYSIPALFCIKYEFI
SFTFYFYFVTKLFTGMYHETITIHTIFFYVFGILHFIVPLIDLVGSTSAVVNEGKTSVELISKWIEVVKD
QEQSTVRMSHFPNYFAQKKLKFTAAGLFPLDGSLILSIAGSITTYLMILLQFEGIKPYSS

>NvitGR15

MVKGTKYLFDIFKVFGLATMSMTDCTKKNNFKNRKMFSYSYHGIIYNGVLICFLIIAGIYKMYYIRDKLIDQSRMSEVIDVFGNFIIYAVSVVLLSKYMISQTLAVRIGNNLYS
INLVLKRFNLKYKNQYMIMHYKLVLLFDITIWLGVIIIGSFSDCTFIAAILTYIPNFIINCLVIQYVVIIIFIYGEAKALNNQLRKYVDRAFSNTLLYQFRRPVLSVHYYLPENNEII
LLQKSCLSIYEVSNNVSKFYSLSILICIVKLFFSIILNTYFFLKPSIFGKSMITSTMNHVWSISWLTLDTFSLCILTQYITMTVNEIKKTGDIVHQILRHSTSLGVIKQLNNFSLHLL
HKNIQFTAMDMFSLDCTLLHSIVGSITTYLVILIQFQENSSEKHKP

>NvitGR11

MKTTIINCILIKCVFYFMKLIGVCPFVLDKKEILKSSTSGKMYNLLLIVSYIYSYVIVIKCRRNLHYSEETQLGIIIDMIGITLKYSAVIVCWYTLAVHQTQVKSIIQHLKVIANN
QTMLASKCRREKINNEFKTFRYGLIVINILGLTILTQNNFINNYYKNCTTDFTFTLFDIFQIVIYNVIFIFLRIVLYTQENYRIINKALNKCTNYNELDNVNVTYDTILSLKKLQSA
GLAHKNISDLLENIVDFFRLPVLLIITAVFVQILIDVHLILYFIKTENWNHIKYYSLIHLLITFAIRVSATYFICSISDSTGIEGNNTKNIINVILNKWRFTKSHKNLAKMFIFNLHEH
KIQISLYGLFNVDYSLLKNLCSSTIMYVIFMFQLDGIIK

>NvitGR10

MVNLSDIKPLFYAARFFGCAPHRVTDSDVLLTTSGLIYSGIWALGFVCCCCYGLRLICAGVYTGERNMLALTAVRTLLAYVCFLADDALTMRWNERLRSALLQLRNFDVAVS
YGRKRSVNWKLRCCCWMLVGTIIAYWIGVGYVTYKCEMTNPLFNAITYVIANAAISMQLIKFAGLLILLRQRFRYLRELLPLEAAHPNSARRAIQLQDIWWLHCSLANAAE
TINSCYSLQLLLWIFTMWLNALSRIYAMNETLVDSGQFLLMLRESLLVTACIGNLMLIALACHYTAREANSVGRAAFAPQTSFSRKRSLLEHSLEVGVYFSLRQLHFSAAGGF
IQVDLPLLLSIAGAMTTYLVVLHNNS

>NvitGR7

KNSSSGSSPSSSIQDIRPTFLIARVFGLAPYAITNSSINVSKRGILYSVPWLGFYLYALYNRLNLYTHSDLETKFRILSVTRTALAVIALLVDLVVCTFRDDRFQNALDCVRKYDL
AVKYDVETNARLMRIHSWTIYSFMITYYLAIGWFTYVDEPYEGVMAAVIYVYLYLPLSIAVMKFVALITSILLRFRHLHRMLLPGFLSIMMELDSEPKRLHLRDVCWLHSCL
CAAAANVNSLYSLQLMLWFANLTFNTISRINDFGQPQNSIDAFKLARDAGLVLIFVTLVFFIAGVCHVTSTQANKVGAVVFSPGSRYFRSRVVDHQDKEDKFYIGQYFALHP
LHFSAASGFFQINLSLLLKIAGAMTTYLVILKSPSNC

>NvitGR45

KNMLFGKQIKKFDYLKLLYYYFKVFGLATMTFVTDSTKTTPNRFGTFSRSKYTIVYNVVIILVFVMPCLYNMTIFCVGTNRVKFEDFADCIQINMALFVTIFILSKFCISSDSLI
SIANSISRITESLLTLSSISLQKRIKVSFEIKQAFIVNITMWIAFIVINLSEIEPWMKNAVNMYVSNFLVSVLILQYSVILKFLQYDFKILNENLIEFRNEDSMKIRSPTETKAKIDGL
LKLQKLHESLSDTSRRVSMFYSYLMLVSVLNIFIMLIFVCYYLAKPIILTHDSNFSSIMLLRCFWYGLLFVVLLVTLTKFVTATIEESRRTKEIISSCLMIPDADEKLLNKLNQFS
LYLLHRDVKFTVWGLFTLDESLLTSMAGSITTYMVIVLQFQQKD

>NvitGR58

MGKKPMTRPILPLLISNWVLGIGIIEYPIGTPRPTFSFIYSTTLLVIYCTTSIMIRHEIFRVSIILKNNTVPMTIVFYTNIFLTISIVTLGWYRSKGLRRYVAKAAVADDLMERIGIPN
NHGKMLRAVAGQVIKGFFLVTVLIAIIAVIVLVEDAPLQTKILISSVMSFPLFTMFVSDAMFTSCVRCACYRFTELNKVLKAVLTSTHAFPQHKRVCSSVFESGGQDSNFVINN

VSQRKNPAVIVKLAKEIHLQLISACQEINNTYGLHLLLSIIFAFAVITGNMYLCYMSSRNSNIPHYILVKTLVVSGIWIVHYGMKICYFSIVCGCCTENSIKTGDYINEFYDEPST
TNETKLKIRQFNMQLIQKPCKFTAWGFVDLNCHLIQVMIGTITTYLMILIQLGTTTYVADDSYSKYLKSFSSLTY
>NvitGR47
MFFGKPINKKIHSFKLLFHYFRALGLATMTCDLAPAKNATKYYWSFASSKSAIVYNVILVLIFVTSNGYSMTYFCSSNYQVDFDTIADCGQTTIYSFVALFILVRSCISRNGLIN
IANSISQITESLQSLSSIEIREKNKVGLDIKKLLFVNITIWTGLFVSTVLEVMPWTKYTITVHISNFIIIVLMIQYSVILKFLQYDFKVLNENLVEFRNEELTKVRLPTETKTKVDR
LLKLEKLHESLSDASRDVSMFYSYPMLASFLEVFIYLIFVCYYLTKPMILGNTYTTFMLIRSFWSGFTYAILLVTFTKSVTATIDESRRTKEIISSCLIIPVDEKVLSKLNQFSNYL
LHRDIKFTVFGLFSLDQSLLTSMAGSIATYMVIVLQFQQNNRLLFHVPYKSCYNKKYAK
>NvitGR53
MSDIINKLEKVVLSIAYYTNWFCGIGIIEYPIGKQHRVMSFLYTGVVLIVYSVLSVYVYSDFAIVSRDYEVNQTMMKGVYCATFILTFSTIVMGWYRNKEIRSILQRMNIAMRI
IDKLGASKNYTKAFTVQVGYAVGTLTFLIIIVIINALVVYKRDPKHDSHVLTVMAVNYPLIILQVVDTLFINIIQYARKNMRVINDVLREMLTSTQDFPQHTKIVRRYLRTLDSP
ELDTDIVDQKTDAEDKMYTINMSKKAHLTLVKICQETDDTFGLHILLSVIVAIITITVSIYHIYMLVDYLSISRAIYDNTLLPVCILLIYYYVKIHAISHFCSSTSEEVAAVITGDII
SELYDDSSIGIESQTEIRQFGDQIVQNSLTFKAHGFVTLDFTLIQNVVGFVTTYLMILIQFGSSTSIEISR
>NvitGR30
GNMLIKNLSLNETVLEKCILYFFKLSGIATLNFDFKLSTNRSKKFSSTFTRSKTGIAYNAALISLITIVTNYLIEFQINHNMYKNFYDKLDIGYAALISVTAVLILVKFSFQQEKTL
TIANELNEIRDSLSLNDCSVDGKGHALRRFIVLVFLAHFLFLTILFSTSYVLNNTTINIVRTTLNYIAIYLSNFIMHSMMLQYSIILKLIEHLSRGINDDLVEFSRPPQGLNSLTFTK
KTTSQRVGQLANLRKNFSSLCKVSQDVSEFYSWPMLLCLSCNFIAFVRAAFYIAMPIVHGTDAFTANIYVRCCCYISHNAFSLIILTKSVTASMTENRKTREIVNDCIENCDD
QEILKKLEKFSCYLMHKKITFSVFNLFSLDESLLMSVIGSITTYLVIILQFQNNNAE
>NvitGR28
PKKFYQNARISDLWFAKCVYYYFKTVGLATVSLRLKSVKKNKKNSSSLCTSSKLGILINVVLSLIVIAIFSYTTIVIAEGTFKNSLKFDRAIGVIRIILGSSAALIILITFSCKQGSI
TEIANNMQVLTIFSVLSANFKTKIGNTNESFSIFRETGGVFFVNIIAWLLLFVTVPTTNWKVFAVTPYVPEVIMTSMLVQYNMVLNLVKRLMEVVNANLLYTSQYDDKYEDN
QITMIKNDRNENSFKRKIIKFTQLRDSHYMLCDISEDLEKFYSRLVLLCITYIFGSLILCSYFNTKEVLKQGVEFLTLRATLFFGVTVIHYIMPLVNLTRSTSAVIAESKRTVKIVN
RWSGNFHNQPEIAMFNQFPNYLDQPNLEFTAGELLALDGSLLISIAASITTYLMILLQVQDTSPN
>NvitGR21
MICKPHYTSDFFFLKCLFYWFKIFGTSPMGIDFTSAVKSNDVPQDVHFVFSKLGILHNAILVCLAIIPSCVTIKEAYHTEYTDRLQLERVIDTVHAVATIMTFYFILINVCINQKR
AVAIANQLNSIYSQSTSLFSKSKIRPNRAFRSVKKIVSIHMVLMLLLCLFAFTFVHQNLIYHLFINFTNVTIYVMALQYSLVLKLLQHLYRSLNADLRSFLITRDRRDPSSISLPI
DELQCRLMQVYEIHASVSRVSQDVCDFYSLPLFFLFAIAFFTLVLFFYYFMLVLLLMNKMIDYGFVLPFFLTLLGLTILAISTLARIAGGTVKESNITREIVSESIMNQRNQPISG
QLRDFLHYLQQKNAKFTVFDLFPINESLLMSIASSISTYLVILLQFKESNSTQQTAPSSAT
>NvitGR14
MKKTLYVCVYYFVKILGLCPFTRKKGRFVKVSHVGMSYNVLITILYSRAFVKAIQNRHSIRLSQETPLAVIIDTFTHVLSYSTIVSSWLVCAFRQKIFIKVFQSFKNVENLEND

LLPSMHCSKNGLEENLKEFRARFIVVNLICIIFTGSTVVIISMCEDMKNQSWFWFIYNIPINVTFNVAFILTEFMRCLRKHYKIINREASKLARSRKRSFARMPMAFSKKLQTIG
RIHSDLTELGETVVKLFSLPVLMTVHGHSANIILAIHGLYRILKSDQSMGGPCVLYAPTVKFMIYSIIIFFICSIPVSTCNEADKTLRIIGQIPYEWHEEEMHNKMIKNLMFQLYQ
KRLQVSIFGFFNLDYSLFRNVWIVIIMYLIFVLQLDPSSFIFAILK

>NvitGR6

QKKSPGNEESPLYEVVCPAVYLARVFGLAPYELEDARPDNNRPKRLGASTVYCFFSIFWLIVYTYIVVISLIRFGGLDRDKPVLGVTEDGKLILNYLVSMVDTLLCIRCRERFV
HVWNSIQDFDESFQLGNVPAGRDSPRYSPILRRARFWVWTILTTNVVGWTMINQLGMHAFGEPYLQNIGYMLTYVGTCVAVLKFVGVVMLLGQRFAYLNEELARQRKKE
EGRRSRAADEIVKKIESSYNKLLSTSEELGNIYSFSLFLYLLNLFCHAVSNMYFFTIWTILDPGYLKNPKIVFCLFSWLLIYLVQMLLIHVACHFTSLEANRMASVLLDWRRQA
YRQSKYEFSSTLHYLNRRLNFTAAGCFNVNLPLLTSIFGHLTTYLVILLQIPDSSNS

>NvitGR4

MYKDLYNTLEPIMWVWRFVGAYPFVIRGPIGSQQYVLSTYSIMLSLIFLIVTLQYCYKSVDFINQSATEYSLFLITISVQQVSNVICFFDSVVLKLFFGKKITRSIENIAIQDKKL
SKIGCSLNYSQAVRKGRLYIFILFIFLCFHIHSELVYFNGGAPLLLRIFGNYLAMLQVANVILFAWLMFNVGLRFQVINCKIKTCIFDIEWADQNSSCLLVLRTTAKAHAQLCQA
AKIINGTFVLSIINCVLSAFIFTTTMLYYIFMELKNTLPFSHAVYYCSVILIHATTIVIIVQSCNWVHRNAHATVKVLHEFSKENNRFDDDKHLNQIIHNFSMQILHHNLTFSAW
GLFPIDSTLLQSLAEAVTTYLVILIQFDPLVT

>NvitGR46

MLFLKKSNNKNLDYLKLLFYFFKVFGLASMTIDATTAKNTRNHFWTFTRSKSTVIYNVIFILVFVISNIYSMTFFCRGTYVVNFETIGDCGQTTLSLFVALFILTKSCISRNTLIII
ANSISRITDSLLSLSSTSIQENSKISSEIKKMFIINISTWTVLFGTFAFDLKPLTKYGIVVFFSNCIINHLVIQYSVILKLIKHNYKILNENLIEFGDQESMAIRSPSEAKIKVDRLLKL
QKLHESLSDTSREVSNYYSYPMLVCVLHVFIILIFVCYYFFKPMILHSKNLSTFTFLRTIGYGFAYGLLLVTLTKCVAATIDEQNRRTKEIIGSCLMISADEKVLNKLNKFSTYLL
HRDIKFTVLGLFSLDESLLTTMVGSITTYMVIVLQFQQNLKR

>NvitGR23

STHCFLFNFDEMFLKSLFYFYKIFGVAPMTLDSTKNRPSDVSFAYSKFGILYNLFLVILTIFAFYQCAILVHFDVSVGMDFQRFVNTTHLIFFVFTTLIVLIIFCVRQERAVALANR
LSSIYYLNKNIKLSAILPSIKGIVSMTFITTIMWLVTTPYDDAHLLTYYVAISLYNFVINSVFLQYSVLLKLLYHLYRSLNTDLRSLLESLDIAIEMDRHSNNEIKLTSGRLKRLRE
IHMLLCHLSGDVADFYSLPIFFCITNAFFVLIIYSYYVFRGFAIAIMPVLVTVHCTTMMVVIVVSLTILLRTASVTAAESRVSGEIVSESMASCSNQFIGRQLEVLSIYFLHKNVK
FCVFNLYSLDESLLMSIVGVITTYLMILLQLDGSSNCK

>NvitGR52

MVDIFVDMEKPFLPMLVSNWIFGIGIAQYPIGVPHRVLSFTYSLLNITLYCVVAFFAYPYYIKFIDVTKSTLTTMIFSFSISILLTIIMITSGWFQAKGVRKCIIKAAIVNYLMQQI
MIPKESTVIFFKEFIKFLIPLTSIFLIITFNLFITFSESFAHIGQIGANFTMNYPIIVMFIIDSSFVNIIGKVKNYILILMYANFKFIKLNELLYSLLTSSADVPQHKRTFKEFIYKEKNSK
FQYLKSIRKDYSDVIKCAKQIHLRLVELCQHASNTYGLHFLISTAFAIGIMIYNTYNIYNILASIVITSAEYNIHIMKPILYNCNWLTYNILKIVRLSWFCDSICKESVRSGDIASE
LYDEPFISENTKSEIRDFENELISNKLTLTAYGFFHLDFTLVHAMICTVATYLMIIIQVKSTCR

>NvitGR50

RNNKINPRKGRPILNIADSFRPFVWINGLMGFGMIEMPYGRPWPKLSVFYGLLRAIAFSILSWYVFKNIQPNTRISALMFLIYKIIIAASVGAVVISSIMGLINHEKSKKLYKKIK
LVDETLKMFGVEPEYASDLRRNRNIIINYYRAIIVLFIIKLGSSYIFSREAFCTRNILNVLYFNIPSIINPLVDINYTSKIHVLEKRFERLNALIHNVTTSPSKMMHTHDFKKYEMIL
NNRGVISVIPKYCFKNRNNIEHLLKVTRQLHLDLCNTARNMNNLIYTQMSAQLSAIFVHLTAGTYCFYFIFNEKTIIPLQAKIHSYVLLIFSIICGIVRIILITYATAGKISKILHEIQ
IQNTEKKLTNEIHQFCMQLKQHPLSFTVCGFVELNFSYVTGFVGAVTTYLMILIQNQTDMIEAAKTMVDPIKSNSTSVTPA
>NvitGR48
RRTKPDADALPSVENVTRTLTPVLWLSRFSGLTVFEMPAGTPWPKFSAAYALFLCSAYGTMIWFGETFIVKETTSVPLVIFIYALVKYVNAFLAAVSFAVGLLHYKKTMKFTK
RLKHVDETLKVFGIEPEYAASRKENIRIVLIWIVATVFQIIGDAAICFVIYDPAYIAILKFFIFHIPFQSMSLLELTFAMKISTVRSRFEKLNALFQNVLENPVLPMHFKHVNKYHN
ILRRQHSEEGDRNRKNLELLLMTSRQLHLELCAITREINEVYGKQLAMSIAAKFIYVTGYGYVFYLYYNEPSISLSIKILNCTYIAYNLTFITVMMIYFIGQSVAAQKTTQIAHE
MPVTQSQTKIIDEIHQFSLQVTQHPLEITAASLFTLNFAFMRGFIGSMTTYLIILIQYQPNIAAAAKSMIDEIMANMQNVSAFYHFANFST
>NvitGR40
MFIKDFINKKDKVFVKFIFYYFKVLGTATISFNTESTKSRKRNEWKFTHSKSSIMYNIGLIIFVTTVSSFGFIYASFQHQTNFKKFERVTDRAEDVFNILSVTIIMMMFCFKYKN
MAGIANKMSMIYQSLISSCPQTLYKSFTDNILLHIILIISPYIIIWPFIIVFNFINFPEFEIYNFTIFMNDIVITALLMHYSTVLILLKYFFKMFNVRLSFMLEEQDYLCEIQYLNCNN
RSKGKIKELFHMRKLYASLYEVSQDVSSFYSGPMFLCIFKILVSATLSLYYVAKPIIIDSHTILNVEIIRNSMFGLIYATALLIFTTLVTQTARESVKTREITNRCIINFENKYIIKELS
QFSTFLLQADVTFTVYGFFSLNQSLLTSMTASMTTYLVIILQFQQNN
>NvitGR39
MDILKITLNFFKMCGLATMRFDAGTTQNTSVQSSWCTSSKKGQVYNLFLICLITASNGYVATIVYEYNISTQEFDKSFDVAQYIYTSATTVAILMLYCFCQGRAVSIVNNLKIM
HKLVTNVNSRLSKEEEPTMGGLKRIAIMTTVIWFVVVFTSCNLSFGVVMYYLTLYPCILIINCTFLQYTMILHLLKQLFAILNANFRYVSRQSIVPKIAVAATNSMLQISTKKTQ
PFSDLCELYTSLCDLSMDISKFYHLAMLFCVSHVFITLTTWLYYITKPLVTGAIELSIIDYTHSLLILMHNFFMLFILTKSVDAVVDEKTGKITNRWLANLQDQHLVNELNLFSN
YLLHKNVSFTVYGLFSLNETLLMSITGSITTYLVIILQFQSGVQK
>NvitGR38
MLSIKHMFYFFKLSGVATMKFNTNLIETGRVQGSWFSGSRKGIAHNIVLICLIFVFNCFGVRTVYYWSHIQFERVIDVVLYAYTTVIAILILIVYCFRQKQAIAIANKLQILREFT
MSINGQLNQGEQLVVSSLKRISVAHLVIWFALIMSTSTEMYMLVYSIATYPCILIINCTIVQYSVVLKYLRQLYVILNANFFNCSKQFSANTSSVRTNSLSDLRELYMSLCNLSA
DVSEFYHLLMLLCLSYLFVTLLLWCYYIVAPIFVKATVLPILYGYVRSLIIVIHHVLMLVILTQSVSALTRENKKTGEIINKGISNLGNQRVLNELNMFSNYLLHKDMKFTVYG
LFELNESILMTFAGSITTYLVIILQFHSRD
>NvitGR37
HQSFQYKLSEKNMITLKRIFYFFKICGLATMKFDSNMAVKERLQGSWFVNSCKGKVYNAILIILLSIATYYVTAFVYDLSISSGEVGKLFDIVSYVFTTITTIVILAVFCLCQKD
AVLIANNLRRTHMLIANINSQLSNEKINILRTAKIICIVNLVMLILVFITTLRQEFGIIMYYTTVYPCLFVINFTFLQYSLILQSLKQQFTILNRNFHYVLRQCIMQKNLGATGSSF
QTHKVRAQSLSKLCELYASLCDLSTDLSKFYYPTMLFCVLYTFMMSTTWIYYTVEPVIVGKTKLTTFKYIHSLIFLIHHISMLIILSKSVNAVVLENKRTGEITNRGLANMQNQ
QTINELNFFSNYLLHKNVSFTVYGLFSLDESLLMSITGSITTYIVILLQFQSSVQ

>NvitGR35
LFMILKNWKISDLLFLKCVFYLFKLFGLATTSIQTKPSTSRFHLPLFTRSKLGQIYNVILAIGISFVYACLIRWTIKHYEHHIISRSHQAIDYTHTTMAMITAIFVLLVFCIQQEKF
LNLGNRTVRLGELLVGFYEVQRPSKQKTLTKHVKEIYIMVGMTWLSIFVTTETGGYFKNVVYFSMIYLCNQIITLTLLQYSAILRCLQQILWFVNENFLQFSKEPCQMDKIKI
QFSKLRELYLSVCEIAEDIERFYAKPMLLCIVYVFVTLIFFASFITKPMVSAVVISDFQICHCSFRILHYVVALIILVKSVTAAVSESKRTGKIVNKWLGDCNTLQVDPKIYHFSN
YLLHHNLHFSVLELFSLDGSLLMSITASITTYLVIFLQLQE
>NvitGR34
LKMPFNNPLTSGAAVLKCVYYVCKACGLAPIAIRSNDGRKLRFPPFEHSKAGLLYNAVLILIILSMSAAIIQCTFVYRTKAEILKFDGVIDVTHNTMASVTAIFVLTVFCIQQRK
ILELANRMRVLGEMSQSLCDVEICGGRKRLLRDVMMICLTTCCTWLSIFFTTQVESYKGLLYFSYIYLCNLIITLTLMQYSIVLRLMQQILRVVNANFHHFSTEPSQRKLKVQI
IETSCQGIGRFTRLRELYLSLSEVAEGFEEFYSQPMLLCIAYIFLTLIFYAHLITKPMVVGTRSVTNPQLCHCVFRIMHYVISLITLAKSVSTVITESKKTSKIFNKWLGTLDSLQL
DPKLYLFSNYLLHHSLQFSVFQLFSLDGSLLMSITASITTYLVIFLQFQNHQTTDS
>NvitGR33
MFGKFDETILEKLLFYFFKMFGMATMKYEISTIKNEKIKKRRLFTYSKIDIVYNSFLILVTTILNIILVGLKIDDKTSLPNVRGVQKITDVVQFGFATLTCVFILVYFCARQKKAI
EMADQICQIYGSMIVNNCDTGEKRSILLITLFVFSINFIFWLVMIITSVNVTMATPNNLIFYYLAVYSCHVIMQTLLMQYSIILNMIGYFFLHINKSLVILLKKPNQLFLDAQCRN
IDKTRGERLLKMRKTYLILCKISEDISDFYSPLMFFCLSVTFVTLIRSGLYIAVSIADKESNLTIKGMIHCIGYVVHYYFLLVMLTKKASKIVTESKRTGEIVSDCVHYVDNQEII
LNQFSNYLLHKKIKFTVFNLFVLDESLLLLFAGSMATYLVIMMDF
>NvitGR32
MWFSNFHDTREAFFVMFVYLFFKAFGLATVKFNFESIKKTLIAGKEASEVLKPSRVGIAYNMLLIIILSILNYVAIRVSYERPDFTDRSELEMKIDTVKAVTACFSSFIILLIFSFQ
QEKYVLTVHEVLSIRQSLISINSAIYFENESIWKIITKLLIFMFVNWILLFITIEIQNDYQFLLYFVTTNLCDMIMTHTVLQFSIALKMIEQLFRVTNANFDHDSKASFRLNDEICL
NVALKKVQVILNKLSRLQDLHLSLCNVFEDLAGFYAQSMLLCVWYIFVSMILSAFYVTKPIITGNTGLSVVMYLRTVIHFLHHTSLLIMLTKCVTDLIAEREKTGKIISVWLA
KIDNQQFEKKLTKFSIYLMHQKVKFSVFGIFSLDNSILLSIIGTITTYLIILQQENLSSNSNNSNCH
>NvitGR26
MHIRNIHCKITIGKVLHYSLKIFGLAPFSLDVEFLSNNKNQVSSEALTCSQLGVIYNLILAVLIMVITYLTFKISNKTHIFGSGTDLDMAIEAIKTVWACISSVIILFLFGTQQKKL
VQCGNIMLMIRERLITINETLYLENKFLWKSITKISLVVIVMYILIIVTLGGYIDLARLIYVIGATLCDVIIIFTVIVYGIKLKMIKQLIKIINANINSISTEFNRASNNILQNNEMKN
AQIICDKLSRLQNLHLLLFNATEDLTNVYATSILLCTLYIFLSIILNLFYILKNVMTGVASLSAILIRHIFIQFIHCTCSLIILTNSVTDLVLESNRTGKIVSEWLTKLKNSRVENEVT
KFLLYLKEHELQFSVFGIFSLDTSMLLSITSSITTYLVILLQLQFQQN
>NvitGR25
VKMLHKNIVNKESIILKFIFYYFKLVGLSCVSFSSKSRLDICFLTSKLGALYNVILALLITCFNYYVVLIVVKVSFGSLHFDRAIDFGRVCLAVISSVFILITYCFKRKKATIILNRI
NTIAELSVNLRSNGKSGHDGLCKPAKRIFFVFLVTWLVLIAVTPKLKFYALLYFAAQYSCEMVIMCMLVQYSMLLSILKQLFETINARFTISSEANFQVRRFSQHRSNEFGLEV
KLNRFSYLRELHLSLCEVAEDLSQFYSQSLLFCIAYVFSSLVLYAYFFVKIVTQKGDGIINTATTRFIIVKLLHYIGPTVTVTWAACAVVNESNRTGKIVNKWMGDSRDQYVAI

KLNQFSNYLLHQKLSFRAAGLFSLDGTLMMSIAASITTYIMILLQFQDSTKR

>NvitGR22

MFIKCLFYAFKIFGLAPMVIDTTSTEKNNKEAHKIIFLSSKLGVLYNAALAILITLPTYLAITFAYSDYVGRLEFEKITDTIQSVFTIFTSVFILINVCVHQKRAVDLANRLQTVN
YLSMTKVSCSDKSVKLLSSIKRIVLANAVTTILYFAVTPSNETRVLIYFLVINVYNTIIQATLMQYSLILKLLHHIYRSLNSELSSLNKSLIFVGEFNQFLNNSVQTILRRLQTVW
VTHLLLSHVSREVSDFYSLPMLLCLSNAFLTLIMYSYHFVRALFIWKEHSGNMVVLILRTFMQVVTVAVSLTILTRAAGLTVSESKRTGEIVSESTVYGHNRQIRSRLKEFADY
LLHKELKFCVFNLFALNEELVMSIAGSISTYLVILVQFNETSSIEN

>NvitGR20

MIVSKCVFYFFKIFGLATMRLDENETVRDSWCSGSKKGQVYNAILTCSIIASNCYVARLVYKENLSHREFEKTFDVVQYVYTTVTVAVILTVFCFCQGRAVLIANNLRKTYVL
VENINSQMSKKEEDPVISGLKRIWITSTVIWISVVFTTSKLQFAVVMYYMTVYPCILIVNCAFLQYTIILHLLKQLFTILNANFLYVSTRQSVVTRKVEAAHSSFQAAEQSSQQ
FSDLRRLYMLLCDLSMDVSKFYHLIMLFCVTYVFSTLTMWLYYITAPLVTGTTPSKLQYIHSLMIVTYHIFMLIILTKSVDAVVQEKNKRTGEITNGWLANLQNQQLINELNL
FSNYLLHKNVSFTAYGLFSLDESLLMSITGSITTYLVILLQFQ

>NvitGR13

MQSRHTYTKLFVRCFLRIIGLFPLFLDINGNPIFSYLGLFFNLCLVFAYIFMSIIAFQKRMTLVLPKETVVAQIVDMIADGLENLNIISCLLVVAFRQKCLVKFYEKLKAIDLRLY
DINVKHCKTFDINMNIISLGRKLTIVGISFFIICTVDHLRLLFDNYLLSIRFWIAYQSTKIVIYNLIIVFCETMIFFRKSFAKLNLLFQQSSSSYDKIQDIGQLHKILSELVDDFVGFY
SFMVGSTIVHNFIHLSSNMYRIYLFLKFNGDWSLLDFLDFTSIMIWLNVKILILYFLCALPAAVSEEANKKSIFIHRMLKAVRENDLTSNNKRIARLLTILYQTNLEISVYGVFIL
DFKCFQSILTTSTMYIVFMIQLEHLK

>NvitGR12

MKKVNGIHIIKTLYFYEKLMGLCPFILSNKIVIKFSYIGAVYNLLITLIYTYYFILIIGLRFELHLTRESTLSIALDAFGLAFQYCSIVSAWLTLTFRQECLKKILVTFAKVNLLANN
LSMTLTRYCLRKLQYIAVRLMLINLMYIVIFLSEHYLLKTYKKFEEHASTWIWFNLPKLVIYNIFGIFIELMIILQQDYRALNKVISYSFSEKIDATSFCNFSESPGVISKKLCTIA
EFHENLSDILEYTTNLFSLPLLFALLASFLHLTLDSYIVYQHLLSKRMWEFNDFSSYVICLVWISTKILGFYFLCSVPDSTSAEANHTVIILIKIINNCYKVRSCRDMMKKLMLQ
FKQKKNYASLYGLFSLDYYLFKNIMSTSVMFLVFMFQLDDLVT

>NvitGR9

GKKWKIFSATDFLSLIKPSLLVCRFFGLISYKILNGKIEQSKNCGSYCAIVTFVYICASLLILYIINVSPYMNRASTWMLQGNCFYTLVNFMLVSNFVFKSSTIKILQNLADTTAK
LPSEKFVKISKWIHSKDLVLYLLLLLHVPKVFVGNIYAVLSKIIGTYAAMTIYLLDFQYNSYVFIIASCFEHINEELVQLNYNACKERGHLLRRVYHHQFNPLLFVKLRYLKQW
HYELNEIIRKINSNFSLQVVATVIMTFTELTFGLYFYILDRRHKVRSLDKEIWYFYYHTMVMYFSTKLLLLTLTCQYANNENYKTRTIVNEIIISTDNKLFKEEIYLFSLQLLHTD
NKFIAKGVQLDATLLTGMAKGIFTYLLILIQFLITN

>NvitGR8

CKNLTFVQITNIYQLMRPYFFLYKLYGLFPYKISKNQIHSSKIGLCHTFFVAMSCIVYFVIAMYQCFYSLDIVFDTTESLMQFTSYFMLGTFIAVYSCASNKYKFLLLKKLILLS
SMLSEKEFFEVAKVIYFKDIIGYIFLMGQIFNIASEDLTAQNISKMFALHITMIVFLMDMQYSNFVFLLKSCLKNVNNNLQLLTKSYEGCEIISCNKSMQLLQFNNLQIKLRKL

QHNHHHVSCVIKELNTVFTLQIIATVLMTFAEVTFGLYFFILHIQGKKGIDLDKQLWFNYFITSVTYYSLKMAVMVWICQETKNESLKTGIIVHDVILNNNNEQLKSELSLFSL
QLLQCNNEFTSKCIVMNANLISGVVSGIATYLLILIQFLNTKKSTSKNNEQ
>AmelGR64f
MHSEDQIQLMMLKTKDGLGEIPKGKGRGSNLKIWSSVMYHKDDNNIEDISANQENDLSTKRPRAERNYFR
NSEALENFHCAIGPVLKAAQIFGMFPVSGIGSSSLSKLQFKIFSLLTMYSGFIALMISFMTIVSMIHMLK
TFNASTFQIRGGLGAATVGAVFYGNSLVGSILFFSLSSRWVSLQYEWRAMERYIDSNSTEPTRLRWKFFI
ISTMVLVLSLIEHVLSIFNNIDGYEWNESNSTFHNFLEIYTLRSHSFIFDTLNYNFVYGLYVFVVSKLAT
FTWNFTDLFIMLVATGLAERYKSLNKKLAVTMTKCQAAFNWRELREDYAILSCIVKKVDDHISPIILLSF
ANNVYFICLQLLNGLSISDKNSVLSEAYFFGSFAFLICRTCAVTLLTARIHDQSKQALPYLYNCSTSSYS
VEVQRLQCQLATDDIALTGLRFFSITRNFMLAVAGAIITYEVVLLQFNGK
>AmelGR64f3
MSEPVAFSANSFNPKTDSLHASMRPIIMLAQFFSLFPVSGVNSPDSSYLRFTWRSPKFIYCTISFLSSSI
MTIFNVLRIVTTGISSIKMTTFVFNGTNLIASFLFLKLAMRWPCLMVTWEKLEKELSQRHRKISKISLSM
KFKIVTIVVMTFALVEHSLSIIHGYFKAKECIEFHREQSILGVYFQMQFPQIFSRTSYSLWKGILVDIIN
ILSTFSWNFVDLFLILISIALTDQFRQLNSRLYSIRGKYHFIVKAMPEWWWAEARSDYNHLATLTRQLDS
HISIMVLLSFATDLYFICIQLLFSFNPMRGIIEKIYFGFSFGFLLARTTVVSLCAATIHDESLLPAPILY
SVSSSSFSTEVMRFLSQVTTDNICLTGMKFFSVTRSLVLTVAGTIVTYELVLVQFNTTQQTDASNATIVC
ELVSLDSLSS
>AmelGR43a
MEVKRVEEKRKILFNNELCQAIFPIYYLGKFCGLVPVRFFVHTSEGCQARLNIIDLIYSLCVLVLLLSAE
IWGLWRDLKDGWEYSTRLKSRTAVIATCSDVLGVMSLTVVCIVGSPFRWKYLQLVINKLIEVDEKIGVSS
AKVARRFTIVLTICSLSYLWFNSIIDFYTWNRKTKVDNKAMTGKGPINYAPLYFMYTVIISTEIQYTVST
YNIGQRFIRLNTSLKDLFNANSNNNDNAIDYFRKCPETAHDMDDKKIWNLKPKRQIVLGSYRLSRKLDEN
KMYVNNISELIMVHSSLCDAVSLINSTFGVVILAVTVTCLLHLVITPYFLILQAGERHEWIFLIVQGGWC
IFHITRMLIIVQPSYSAIAEAKKTAVLVSQLLSCTFEANIRRELEIFSLQLLHRPLEFSACGLFSLDRNL
ITSIAGVVTTYLVILIQFQNADDTKDDFDIIRNATQILKNASPLQNFTGLKTIV
>DmelGR21a
MTFLDRTMSFWAVSRGLTPPSKVVPMLNPNQRQFLEDEVRYREKLKLMARGDAMEEVYVRKQETVDDPLE
LDKHDSFYQTTKSLLVLFQIMGVMPIHRNPPEKNLPRTGYSWGSKQVMWAIFIYSCQTTIVVLVLRERVK

KFVTSPDKRFDEAIYNVIFISLLFTNFLLPVASWRHGPQVAIFKNMWTNYQYKFFKTTGSPIVFPNLYPL
TWSLCVFSWLLSIAINLSQYFLQPDFRLWYTFAYYPIIAMLNCFCSLWYINCNAFGTASRALSDALQTTI
RGEKPAQKLTEYRHLWVDLSHMMQQLGRAYSNMYGMYCLVIFFTTIIATYGSISEIIDHGATYKEVGLFV
IVFYCMGLLYIICNEAHYASRKVGLDFQTKLLNINLTAVDAATQKEVEMLLVAINKNPPIMNLDGYANIN
RELITTNISFMATYLVVLLQFKITEQRRIGQQQA
>DmelGR63a
MANYYRRKKGDAVFLNAKPLNSANAQAYLYGVRKYSIGLAERLDADYEAPPLDRKKSSDSTASNNPEFKP
SVFYRNIDPINWFLRIIGVLPIVRHGPARAKFEMNSASFIYSVVFFVLLACYVGYVANNRIHIVRSLSGP
FEEAVIAYLFLVNILPIMIIPILWYEARKIAKLFNDWDDFEVLYYQISGHSLPLKLRQKAVYIAIVLPIL
SVLSVVITHVTMSDLNINQVVPYCILDNLTAMLGAWWFLICEAMSITAHLLAERFQKALKHIGPAAMVAD
YRVLWLRLSKLTRDTGNALCYTFVFMSLYLFFIITLSIYGLMSQLSEGFGIKDIGLTITALWNIGLLFYI
CDEAHYASVNVRTNFQKKLLMVELNWMNSDAQTEINMFLRATEMNPSTINCGGFFDVNRTLFKGLLTTMV
TYLVVLLQFQISIPTDKGDSEGANNITVVDFVMDSLDNDMSLMGASTLSTTTVGTTLPPPIMKLKGRKG
>DmelGR43a
MEISQPSIGIFYISKVLALAPYATVRNSKGRVEIGRSWLFTVYSATLTVVMVFLTYRGLLFDANSEIPVR
MKSATSKVVTALDVSVVVMAIVSGVYCGLFSLNDTLELNDRLNKIDNTLNAYNNFRRDRWRALGMAAVSL
LAISILVGLDVGTWMRIAQDMNIAQSDTELNVHWYIPFYSLYFILTGLQVNIANTAYGLGRRFGRLNRML
SSSFLAENNATSAIKPQKVSTVKNVSVNRPAMPSALHASLTKLNGETLPSEAAAKNKGLLLKSLADSHES
LGKCVHLLSNSFGIAVLFILVSCLLHLVATAYFLFLELLSKRDNGYLWVQMLWICFHFLRLLMVVEPCHL
AARESRKTIQIVCEIERKVHEPILAEAVKKFWQQLLVVDADFSACGLCRVNRTILTSFASAIATYLVILI
QFQRTNG
>DmelGR64f
MKILPKLERKLRRLKKRVTRTSLFRKLDLVHESARKKAFQESCETYKNQIENEYEIRNSLPKLSRSDKEA
FLSDGSFHQAVGRVLLVAEFFAMMPVKGVTGKHPSDLSFSWRNIRTCFSLLFIASSLANFGLSLFKVLNN
PISFNSIKPIIFRGSVLLVLIVALNLARQWPQLMMYWHTVEKDLPQYKTQLTKWKMGHTISMVMLLGMML
SFAEHILSMVSAINYASFCNRTADPIQNYFLRTNDEIFFVTSYSTTLALWGKFQNVFSTFIWNYMDLFVM
IVSIGLASKFRQLNDDLRNFKGMNMAPSYWSERRIQYRNICILCDKMDDAISLITMVSFSNNLYFICVQL
LRSLNTMPSVAHAVYFYFSLIFLIGRTLAVSLYSSSVHDESRLTLRYLRCVPKESWCPEVKRFTEEVISD
EVALTGMKFFHLTRKLVLSVAGTIVTYELVLIQFHEDNDLWDCDQSYYS

>CfloGR28a
MPKHVTSILNALRPLCLYNGILGLTIFEMPIGIPWPKLSILYAVVRSIIYVYFFWQFRRMSHINYFAIETMITTIRIIHL
SNIFIAVVSTILGFKNYKEVKTFAKKITLADETLEIFGIDPEYKATFDLCMQAIFIWCFGSCVILSSDLAIAYYYFDDFY
DMLVRVLLFQFPLILNLTNELNFYLIIYILGTRFERLNSVVQSATSSALQPDDLKNHNKYQNTMKKYINKVTVQPVHYNK
NRNNIEFVLRIVRQLHLDLCAMSREVNDTFSKQLSLQMAATFLLLTGFGYCTYVVYIDVNYSLAKKIHHFYTLGIWIAMS
IFRIMCVIRVTVNVSEESQKTSQLAHEIQVSRWQKKITDEIHQISLQIMQHPLYFTASGLIVLDFGFVRGFVGSVTTYLM
ILIQNEPDMVKAANALVEVSSDNETSSKS
>CfloGR64f
MSELRFRATLAESPQHGRYETFKMDSIPIDASRSSASNSGANLIESTRSFHCALKPIVTLAQCFAVFPVDGVQSSDASSL
KFTWKSFKVLYCCLSATGSIVLTMFSVYRLATTSITSSKTSNLVFFFTAGITVLLFLKLSRQWPSFAVSWENMERELAAR
HNSRRLNAISLILKFKILSAVVMILALVEHTLSILSGYVSAIECAHIRGHSDIVATYFSLQFPQIFTETNYANWKGVIVQ
YINILSTFSWSFMDLFLILISVALTDQFKQLNHRLYSIRGKHCFVVKAMPEWWWAEARVDFNRLAAMTRRVDDKISDIVM
LSFSTNLYFICIQLLNSFKPLPNAIQTVYFCFSFGFLLSRTVAVSLYAASVHDESLLPAPILYSVCSASYSAEVRRFLNQ
VTTDHVSLTGMKFFSITRSLILTVAGTIVTYELILVQFNAVQSDHQHSVSNITKICESPDSSITDVKIEERYFCFLIAEH
ILSILSGYARSVQCARNHGNIDVANVYFILKFPELFTKHNYALWKAFIIKVVNIISTFSWTFIDLFLILMSVSLTDQFKY
FNRRLYTTFDKIMPEDWWTEARKDFNYLATITRKVDSEISSIVLLSFSANLYFICIQLLNSFNPMPNVIHRIYFCFSFGF
LLLRTVAVSLYAANIHEESLLPASVLYNVETTSFSIEVERFLSQVTTDHISLTGMKFFSVTRSLILTVVGTIVTYELVLM
QFNSIQDDQQENSSNMTKVCNLK
>CfloGR43a
MEVWSDEPKKNKTTTTRPRNDRLEAESKVGPNSEKIKSDVFHTLAPIYHMSKIFGLLPVKFTKDSGGRYRGRLQGSQIIY
GIVVLLGYMGAQCYGLYRDLRNGWQNSTRLSSETAITVTCSDVLAVISVVTSAIIGSSFRWRHLQAALNMIVDVDEKLGI
MTSKNLRRGSIIVISCTFVYLVTISLLDYISWSYSFDKKNVQVYDDKGPINYSPIYFMYIVIMTFEMQYALVLFNVGERF
LKLNKTIENLTKTNLIIEYFRKDMGLTAPSDPRKNLFQAISFVSSDIGHSRLRRTNKVSDFIVSSEGNGGTSEAIDQLIT
LHGTLCDCIIRINKAYGGTILIGTISCLIHLIITPYFLYNEIYLDNSSNWYVLALQVFWIIFHVYRLLLFVQPCYMVSVK
AKITGALVSQALAQNWDPKAKKQLEIFSLQLLHRPVEFTACGLFFLDRGLVTSIVGAVTTYLVILVQFQNADDTKGTKHL
LQNATELLKNASSFKNISVFKNTS
>TchiGR43a
MGIRRHAIPRPTTSTYHVKSDNMDAWSISPKDLKPIGVAAADQQKQQQLQLQQQPLGPKKLTSDDLKKGPDTGSGGGGGS
KSDVFHTLAPVYHLSKICGLLPVKFKANKAGKYEGRLDVAEVAYGIVLVAALAGAQCYGLYRDLRNGWENSTRLSSETAI

TVTCSDVFAVISAAFVAILGSGYRWHHLQDALNKIVDVDDKLLDVPTSERLRKVSIIVIVSSLVYIVVISSLDFVSWRAS
SAGKNNAHFGDKGPINYAPLYFMYIVVTVFEVQYALVLFNVGERFLKLNKSVANLTRSNMALEQLFRRYTHQPHHQQQQEQSMAFFSNEIGHIGRFRRVNNKISDFGPGAG
TETASKTAELIGRLIGLHGILCDSVKNVNKAYGGAVVVGTISCLIHLII
TPYFLYNEIFSDSISSWYVLTMQVFWTFFHVCRLLFLVQPCHMVSVEARKTGALVSQALASNWQPEAKKQLEIFSLQLLQ
RPVEFTACGLFFLDRGLVTSIAGAVTTYLVILVQFQNADETKGTKNLLQNATELLKNASSFKNITFKVR
>TchiGR64f1
MQQQADLEEDPYELKRIIVLDNDKNTMANGDAADGTDHNHHNHHHHVTSNGLISKNDVANGRIFSKAVVA
AANNNNNNAKKRQQVGSDESTLFAKVHHHNRTEARGALDTRKLSTNSDDQECFHRAISPILLLAQFFGIL
PINFIRAESITKLSFHKLGPRVIYSYCVICCIVFMTSVSFLHLFVTLNASSFQTRGGIADATAGAVFYGN
SLLGNFMFLRLCPRWIAVQYDWRAMERLLDKVKTKRPRLRWRFATIAAAILGLALVEHLLSMVNNTPASV
VGGNHSFEEFLAVYTQKSHGFIVKHVDYNFTLGLFIFFISKISTFTWNFTDVFIMMVSTGIAERYKVLNA
RLVGLTTSQLTVSDWHDLRECYASLSVLVKKIDDEISGIILLSFGNNIYFICLQLLNGLSPSGDDTSLVN
SIYFFGSFIFLIGRTISVTLLTARINDQCKVILPVLYNCPSANFCMEAQRLQQQIASDDVALTGFRFFSI
TRNFMLAVAGAIVTYEVVLLQFNIALQREEDFLEEQLLYANLDDDDD
>TchiGR64f2
MRPHRIDPVANSLTTIMNNNNINNNNNNNNNSNNNNGSIYDNNAEDNPADGKDYFYQPAWAIVNRRNVFNQHQHRRAPGGSAEIEEAVEQSNRREMHKLDTMPIEASTQP
SALSNSGGGGIDDLVDGQRSLHCALRPIIILAQVFAVFPISGVNSSDASTLEFTWRSPKIVYCCLSSLGSLVLTLFSIYRLATTSISSSKTSNLVFFFTAGITTILFLKLARQWPAFA
TTWQSMERELAT
RYTATTTTAQEGENNQRDDNPSGLEALSLSAKFKVLSTVVMSLALVEHSLSLLSGYVSALECASLRGHANIAATYFSLQF
PQIFTESNFALWKGALVQSINVLSTFSWNFMDLFLILVSVALTDQFKQLNRRLHSIRGKHGVAVKTMPEWWWAEARIDFN
RLASMTRRVDSQISDIVLLSFSTNLYFICIQLLNSFKPMPNAIQTIYFCFSFGFLLLRTAAVSLYAAAIYDESRLPAPVL
YGVCSSNYSTEVRRFLNQVTTDSISLTGMKFFSITRSLILTVAGTIVTYELVLVQFNAVQADHLQSDSNITKVCETHELT
DVVIDEF
>TpreGR28b1
MKCTGELVAAKFAKMFTALKLTELTFIRLLLRIFNVFGIAPLTLVQRGPQKEMRFVSSRLAVLYTYAFIAAIGVSNVMTV
DAIYTHRYEKKMSLFHIIDFCEIFLGTFVTTMILCTNCLKNRTFSAIATKMYRINESIGQLSAFEDGSGNKARRGADHCF
SIVGLVGFHALLTLIVLSSSAFYQENFVLFVSGATRNFVITWLCLQYAVIVNILQRSFSRINDLLRNLAGGRDAARINNN
PVKTIEMKNRYVIKNGHVDETTVPQLIAKASELHYALVRTIGEVTDFYDYTILGAVIYIFVCLVVFSYFILDYIIEKRIF
STVEYVNLFLWIVSFMSAFIALTMYVTKTLNEIDKTGMLVQELIRAKVNNQISIELKNFSSYLLHVKVNFTACGFITLNG

SLVQSILGSITTYLVILMQFQLDTSSKMKCQHRNIESP
>TpreGR28b2
MITENSSSGVEKKARAKRSSTLLRYPSDLTCLKFLLLIFKSLGLATFSIVESNDKNQSRVDFVDSTSSLVYTLFLLGLIA
LTNCWGFLSVYNFRYKRTLKLFNAIDLLEVFAGIVNSLAIGLFYCWNQTRSAGLAARIYRLKLSGDSLRDRDRNSPLDAV
LLVGFELLLFLALLVTCVVFYDRMLYTISSTLRNLVVAWLFLQYALVVRSLLGVAARINSALRALPPRISSGTSCCSVLV
NAKCLQALLRLRRMHCSLVETYHEVAKFYSLVVLGSVMYAFLCFVVILYFLMNLLLERRLMLSAIEYVQLLLRFVGLMSS
LVALLIYVTKIGEEFDQTGILVHRLIQKTLNKDVLTELNDFSLYLLHARTNFTAYGFFTLDGSLIISIFGSIATYLVILV
QFQLEFRSDEAR
>TpreGR28b3
MSAFLLRLHYFFKIFGLSTMSVEKLSKFNRYRFATSRKDLAYNVALILGFLLIGSYYTRVTWLMDYEGRSLFEKTWEVFD
IVFAFSVSITLYVLYCARQRRIVRLADRFYALARSLAGNSDELSKAITRRTRRTFVTYLILYAIFTISAQPAAYINGYLD
HAVMAGLALNFILLTVLLQYCLVLICLVEMFAMANCHLEGIFRRSSHKSYIEPRFKSSSNFLNFELYKNQDSQFSYHRSQ
YFSLCDLSQDLSDFFGLPMLAVVSFIFFLIIFAIYYPARTVINQVVIMGYTTFFFYCQTWFGFLATGYLLVVLTNSTARI
GKEKERTAEIIIKWLAIVDNAATEKQVCVARDKSVMPCIVSNRVFTLQLDQFYKYLQSKNVQFSVFGLFKLDQSLLLSIV
SSTTTYLVIVLQFRETK
>TpreGR2a
MRSQERVRKESALYEVVCPAVYVSRAMGLAPYELTARGQLDGQARLAPSRLNCLYSLFWTSLYSYIVVTSLMRFAGSKRD
KPVLGVTETGKLLLNYAVSLVELSLCVLRRAEFAGVWNAIQAFDEAYDIGLDNNKGGGDRDQQQQQHHPLLRQAKHWLWLLLFGFVLAWVSVNQTGMYAFNESYVNNI
GYMLTYVGTCVAVLKFCGMAALLLQRFRHLNQLLQAKLDTNVNDSKVDYARETEDVDRIETSYNYLLAIGERLNEIYSLPLFLYLLNLFAHAVSNMYYFSIWTIVDRDYVS
QPRVLLCLVAWLVVYFGQMCL
IHIACHFVSAEANKMPIVLLDWRRRDITSNEYRSTLHYLNRKMRFNAAGCFYVNLQLLTSTFGHLTTYLVILLQIPSEGSE
>TpreGR64f
MRPHRIDPLANSLTTVMNNNNLNNNNINNNNNNNGSICDNNAEDSPADGKDYFYQPAWAIISRRNVFNQHQHQHHQRRAPGGSAEIQEVVEQQSNRREMHKLDTMPMEG
STQPSAPSNSGGGGLDDPVDDGQRSLHCALRPIIILAQVFAVFPISGVNSSDASTLEFTWRSPKIVYCCLSSLGSLVLTLFSIYRLATTSITSSKTSNLVFFFTAGITTILFLKLARQ
WPAFAATWQSMER
ELATRYTRTTTTTTTNTAEEGENRGDDNPSGLDALSLSAKFKVLSTVVMSLALVEHSLSLLSGYVSALECASLRGHANTA
ATYFSLQFPQIFTESNFALWKGALVQFINVLSTFSWNFMDLFLILLSVALTDQFKQLNRRLHSIRGKHGVAVKTMPEWWW
AEARIDFNRLASMTRRVDSQISDIVLLSFSTNLYFICIQLLNSFKPMPNAIQTIYFCFSFGFLLLRTAAVSLYAAAIYDE
SRLPAPVLYGVCSSNYSTEVRRFLHQVTTDSISLTGMKFFSITRSLILTVAGTIVTYELVLVQFNAVQADHLQSDSNITK

VCEVK
>TpreGR66a
MKREIKNIVEAYQPLLWLNRILGLAVFEVPIGRVWFFFSIFYALIRSLAYGFLLWYAFLLIPPMPLSYHSVMLMFRIIVY
INVVIASVTTILGIYNHKKTKKFFKNVSMIDATLECFEIEPVYYDDLKENLRLVLAWLTGVIIIFTCDLILIYAIFDHLG
HALAVVVAFEIPLQINSMIEVNFIVYIRTIGRRFEKLNELIKNITTHPSQNDLQHVKSLDLLSPKKTKKNKNKISVRSSS
YIKNKYDVEIILKMSRQLHLNLCTTSREVNQTLSKQISMQIASSFFILTGFGYCIYLVYHLPNVSLPRKIQLFCSLGAWI
VVIVWRMLQVVRTTVKVSSEAHKVSQIAHEIHVTKFQSKLVDDIHQLSLQVMQHPLFFSACGLIVLDFKYVRGFVGSVTT
YWMILIQNQPDMIRAANVLVSSIDDNSTLSTTAP
>TpreGR68a
MRLLPRTAVEKPLYLTYLVNWLCGIGVIEYPMGRPRPWLSFGYSGGCLVVYCTLAVLAAPELAHCFPAEMLLPTTILFYS
HIVLTVSTIVLGWLRSEGMRACILNLAATDSLMDRFEATSKNYSRVAPCRMLEIVARLAMVLFIMILSSYLFYEEDTPSR
TRVLVSVVLSYPVMLMFVADTTFINIVNCVTYRFRNLNDLLENMLSESNEFRRSDRSKRSFEEKYTKWNMNISESAKKDQ
WNNIKLAKKIHLALVNICQEADRSYGLQIILSSVVAFAIITGNMYVNYLVLVELHLPRQIMMKSIIGGAIWIAYYTLKIR
CFSCVCTRCVEQSVTIGDIVNKFYDDPVIQLETQAEIRDFNIQMIQQPLKFTACGFLTIDFTLVQGMTATITTYLMVMVQ
LRKSSTSYAMMNAINSTK
>TpreGR43a
MTARRFTAVRSNSDDCTHSAARDPTTNNEYSQIGRYGCLVHQSQRSEADRRWGSRSAKAAAATATAATPWTQEIDDGGDGGNKSDVFHTLAPVYHLSKICGLLPVKFKVN
KAGKYEGRLDVAEVVYGIVLVAALAGAQCYGLYRDLRNGWENSTRLSSETAITVTCSDVFAVISAAFVAILGSGYRWHHLQDALNKIVDVDDKLLDVPTSERLRRVSIIVIV
ASLVYIVVISSLDFVSWRASSAGKNNAHFGDKGPINYAPIYFMYIVVTVFEVQYALVLFNVGERFLKLNKSVANLTKSNMALEQLFRRFTHRHQQQEQSMAFFSNEIGHIGR
FRRANNNNKISDFGPGAGIGTEAPSKTAELIGRLIGLHGILCDSVKNINKAYGGAVVVGTISCLIHLIITPYFLYNEIFSDSISSWYVLTMQVFWTFFHVCRLLFLVQPCHMVSV
EARKTGTLVSQALASNWQPEAKKQLEIFSLQLLQRPVEFTACGLFFLDRGLVTSIAGAVTTYLVILVQFQNADETKGTKNMLQNATELLKNASSFKNITFKVR
>MmedGR64f
MLWLEITNDSSRNSAIEYSFTFRQNERPLTQRILSHIHRMKKKQFLGENSPLYTSVCPLV
YIIRGFGLVPYEFEDNQLVPCDSYMIISFFWLFMYTYIVSGFIIEFIESEKNRKKVLLYA
EQARTVFNFAVVISDLLLCMRTRKEITWIWNKIQDYDQAMRDLGYAKNEKSARMWVWFII
GGNTIIWAVVSSSGMNAFNEPWLHNVSFMIIYVGAAAAITKFSGLVMILGDRFKQLNEIA
RSSVQRSRWIHSYPIIDDKLIDCLHSELTVIGNNINKVYKFSLLLWCANLSFHSVCCGYF
VLNWLLDGNFRWKYIECLTAWFVASVYQLFLIHYSCHYTSSEANCMSYIMLGWKRWLYTH
DSKMEVETSIHLVNRQLHFSAAGCFYVNLPLLHSTAAILTTYMVILLQID

>MmedGR6

MIHPAGKSAQPSLIASEIKHKAVPCEPTFNTWNILKKDQVKPNRSGLMRNSDSLHVALRP

VITLAQCFALFPVNGINAPDASGLSFTWGSFKILYCALTLIMSAFMTVASIIRILSTKFH

TTKITTLVFSVTSCLTSLMFLKLARKWPKFAKSWEKIEGELTIRYNQPSKYSLVKRFKVV

TIIIVTLAFLEHALSLASGYISARECACLLGDNDVAAIYFKTQFPQVFNKTNYALWKGIV

VQCTNLLSTFSWNFMDLFLILLSTALTYHFNLLNKRLNNVKNKTMPEWWWAEARSDYNNL

ASLTRQVDSYVAHIVLLSFGTDLYFICIQLMYSFDRMTSVMRTIYLSYSFGFLLGRTTAV

SLTAASVHDESLLPAPVLYGVNGSSYSSEVIRFLTQVTTDNIGLTGMKFFSITRSFVLTV

AGTIVTYELVLIQFNNVQQVNHLNLTNVCEVK

>TjapGR46f

MRPHRIDPLANSLTTIMNNNNNNNSNNGSNYENNCDDKQADGKDYFYQPAWAVVSRRNVFNGLGGGQHHRRASGGSAEIQEATEQQSNRREMLKMDTMPLEASNSDGGI

GQLVDSQRSLHCALRPIIILAQVFAVFPISGVNSSDASTLEFTWRSPKIVYCCLSSLGSLVLTLFSIYRLATTSITSSKTSNLVFFFTAGITTILFLKLARQWPAFAATWQSMEREL

ATRYTTSSSSAATTMTTAEIRRNSESGGGGGGGLQALSLAARFKVLSTVVMTLALGSFVHLFICIYFFSHNEDNDVKMRTILHALSRALVVAVVGLRERPGVRLAARPRQRR

RDLLLPAVSSSGQRFSFFFYDLFFSPHLHHTL

>TjapGR66a

MKREIKNIVEAYQPLLWLNRILGLAVIEAPIGRVWFFFSIFYASIRSFGYGLLLWYAFQLIPPMPPSYHSVMLMFRIIVYINVIIASVTTILGIYNHKKTKKFFKNVSMIDATLECF

EIEPVYYEDLKENLRLVLAWLIGVIIIFTCDVILIYGIFDNLGHALALVIAFEIPLQINSMIEVNFIVYIRTIGRRFEKLNELIKNITTHPSQNDLQHVKSLDLLSPKKTKKNKNKIS

VRSSSYIKNKFDVEIILKMSRQLHLNLCTTSREVNQTLSKQISMQIASSFFILTGFGYCIYLVYHLPNVSLSRKIQLFCSLGAWIVVIVWRMLQVVRTTVKVSSEAHKVSQIAH

EIHVTKFQSKLVDDIHQLSLQVMQHPLCFSACGLIVLDFKYVRGFVGSVTTYWMILIQNQPDMIRAANVLASSIDINDTLPTTVP

>TjapGR35

MQIAYNLFVSLTLISLYCLLLLDTPKNFHDNHLVFNRIFDTIHTTLAVSAACFILLVFSCRESRINDLARKLGELIVLDKQFSSDAKILTSIPCSTLLANILTTRTSGTDSKLVIYFG

LVYACNFIVTCALLQYSLTLRSIRRILLTVNRDLESMLELSAWSAASIRRDLSAKISKSRQSCSDACELAKQVAELYSLPMLFCITYVFVTLIFFAHFIMRPFVIDEASLTDFKLF

HLSLRILHYIVVLILLTKSASLLIKTVLSDPAMSEPKRAFIRNHESICVFQKNITSAIVNKSLEHFEENINDHQIVLKVRALASVDFEKRKTHCIHSCIIFPINFSAKTSTF

>TjapGR28b3

MAFSYFECIVMYVLYCTRQREIVRLANKFYAIAQSVAGNSDKLLRAILRRAKIIFVTHLVLFSIFTLSVQPLVDPEGSLDHTVLTCLIIDFILMSVILQYCLLLICLIEMFATINRR

LEGLSRKSSHKSIDARFKLSSHFALETNESQNSQFSYHRSQYFSLCDLSQELSDFFGLPMLMIVSFTFFVIIFSIYYPARKLINKVEIAGYDTLLSYFRTWFWFIVIGYLFVVLTN

STVGIGKEKERTAEIIIKWSAIVDNTATEKQLDQFYKYLQSKKVQFGVFGLFKLDQSLLLSIVSSTTTYLAIILQFRETN

>TjapGR43a

MYIVVTVFEVQYALVLFNVGERFLKLNKSVANLTRCNLALEQLFRRYTHQQQQQEQSMAFFSNEIGHAGRFRRANKVSDFGPVGDSPSKTAELIGRLIGLHGILCDSVKNV

NKAYGGAVVVGTISCLIHLIITPYFLYNEIFSDSISSWYVLTMQVFWTFFHVCRLLFLVQPCYMVSVEARRTGALVSQALASNWNPEAKKQLEIFSLQLLQRPVEFTACGLFFL
DRGLVTSIAGAVTTYLVILVQFQNADETKGTKNLLQNATELLKNASTFKNITFKI
>TjapGR2a
MRSQVRVRKESALYEVVCPAIYVSRVMGLAPYKLTARGQADGRARFAPSGLNCLYSLFWASLYSYIVVMALMRFAGSKRDKPVLGVTESGKLLLNYFVSLVELSLCVLRRA
DFAVVWNAIQAFDEAYDIGLKNGEHKKQDHPLLRQAKLWLWVLLFGFVLAWVSVNQTGMYAFNESYVNNIGYMLTYVGTCVAVLKFCGMAALLLQRFRYLNQLLTDKL
AKDWTKPEDYARETEEVEVIQMSFGRASTKLT
>TjapGR68a
MLSESHEFSRSDRTQISFEEKYSKWNMNISESAKKDQSNNIKLAKKIHLGLINICQEADRSYGWQIILTSVAAFAIITGNIYVNYLVLVELHLPRTIMIKSIIGGIIWIAYYTSKIRC
FSSCCTYCVEQSVSIGDIVNKFYDDPVIQLETQAEIRDFNIQMIQQPLKFTACGFITIDFTLVQGMTATITTYLMVMVQLRKSSTAIAMMNGINSTK
>TjapGR28b2
MLLRYPSSLTRLKFLLLIFKSLGLATFSIVQSDNDDDDDDDVKRQSSVVFVGSSASLYYTLSLIGLIALTNCWGFLSVYNWRYKRTLKLFNVIDLLEVFAGIVNSLAIGSFYCW
NRQRSADLAARVYRLILGAARHLRRLLRPNALHGQLISTQSRGHLAIFAVWSGGQDIARRRSCHQRRASGAPSHINFVPEPIQQRPLERQVSSGAA

Supplementary Data S7. The amino acid sequences of 29 ionotropic receptors (IRs) from 12 Hymenoptera species.
>BdioSNMP1a
MAMNRMMKFAIAGPCLMMFGILFGFVAFPKLLKGGIHKMVNLKPGTDVRALWSKIPFVIDFKIFLFNVTNPDEIKNGAKPIVREVGPYYFEEWHEKNNLVDNEEEDSVTYS
PKNTFIFRPELSNGLTGEEELMLPHIFILAMVFATMREKPSAAPLINKAINSIFKNPENVFVKAKAMDLMFRGLPIDCSVTDTAGAAVCSLLKANADDLIVDDPDHFRFALLGA
KNGTTSKNRIKVLRGVKAQNDVGVVIELNNKTKMTTWNDTKCDAYQGTDGYVFHPYLYADEDIVSFAPDLCRSIAAYTESTFKKQGLMVNRYTAWLGDPVKHPEQKCYC
PTSGCLKAGMMDLHKCVGVPLVASHPHFFRADEEYLTTVDGLSPSAEKHMIFIDFEPFSGTPLEAQKRLQFNIQIHKVEKVKIMKNFPSAMLPLFWVEEGLVIPDSFVSQVK
MLHMVVTVMKCLTWIKIVAGLGMTGYAGFLYYKSTQSSQKLEITKPPKQENGVKPPISTLDANFLRAQVPPAID
>BdioSNMP1b
MGKMSLMKKLGIGGGIMFVLSILMWFGFPKLLVSMIKSQINLKPGTDVRGVWSKIPFPIDFRVYLYNVTNPDEIKAGAKPIVRQVGPYYFEEWHEKDNLIDRDEDDSVEYAI
KNTFYFNAEKSGEGLTGEEELMLPHVFILAMVMTVVRDKPTAVPVINKAVNSIFKSPDNVFMKMKAMDLMFRGLMVDCTVSDFAGGAVCGMLKENPDGLIVYDEDHFGF
ALLGAKNGTAAKQRLRVLRGRKNLMDIGKVIEYDGKKNMSKWDDEKCDAFNGTDSYVFHPFLYEDEDVVSFAPDLCRSLGAYFQKKTKLAGLNTNRYTAYLGDPSTDEN
LKCNCEAPDKCLKAGMMDLHKCLGVPLVASHPHFYMADESYLEMVDGLSPKQEDHEIFLDFEPFTGTPMYAKKRLQFNIPITKVDKFKIMKTFPTAMLPLFWVEEGVLLP
DELIAQVKMIHTLLTVVAVMKWLMLVGGLGMGGGAGFLFYKATQNGNKLEITKVPKQNGKPSSTEKKISPLNVNTLQAQVPPVLD
>BdioSNMP1c
MKNTYYFNAEKSNGLTGDEEIVIANLFALGLVNTLLREKPSAIPIFGKAIDSIFKKPDNLFIKTTPKKILFDGIPIDCTAKDFAGAAICGEVREKYEDFGMIKTAEDEYLFSLWAT
RNATESKKPTRVLRGLKNIMDVGRVVEYNYKNNLSIWDDEYCDRLNGSDGTIFHPNFDKHGKDPMIAFNDGLCRAITLTYEKPSKFKGLKTLQYTTDLGTDPDNPLHKCY

CQAPDNCLKKGVYDAYKCVGAPIVVSNPHFYLADKEYLEQVEGLKPDKALHAVAIDLDAFSGTPVQAHIRAQFNFFLFKVEKYKIMKNFPSALLPIFWFDEHTVLPKFLIKE
VKMGHRLLMMGNVFKYLVLLGGLGALGYGSFMQFKEMQGNEVKIKKTVKAKNGNETNGNDKKMPMNISSTISVPPIVE

>BdioSNMP1d
MMITPLWFTHLKEVALTRGTLKRQTWTKFPFACELRVYAFNVTNPDEILKGEKPILKEIGPFVYDVWHEKINLVDDEETDTVTYSVDNTYLFNTEKSKNLSDTMELTVASYL
AFAGVNSAMKDYAALISVASKAIDAVGRNPPSIFLKEPLRTFLFGDEYYDCRGLTDFAPKVMCNSARDHYTEYRMRKRAEDLYSYSVFSGVSATTAYSIKHYL

>BdioSNMP1e
MGKIDLFSTRIRKIGAAGLCLFIFGIILRTAILHSMLESQIKKRIVLKKNSAMREIWSRFPYEMHVYLFNVTNPDEIAKGQKPIVKEVGPFVYDVWQEKINQIDYEDNDSLSYL
SKDTYFFNTEKSNGLSDDTELTLIHLVCIATPNKLLKINPTVLSYGVKGIYNIMNNPESIFFKFKVRTFLFDGIRFSCEGKDDFFATAVCNDIKQFWFSYRMEKGDDYFALSIFG
TMNGTDFYNGKTRIQRGTKNMMEVGDIVEYNDMNNMSIWKDDICDALLGTDGTMFHPFLDANKNNVLHVTRPFYCRSFTYQYDSDVEFEGLKLVRYTASLGSDLGTNP
KDKCFCISPDRCLKDGVYELLKCFNLARIGSNPHFYLADPYYGTTVEGLKPMKEKHMSIIDIDPLTGLAVRSHFRIQFNVELTAIKKYKLMQNFPDAMLPMTWIDEVTIMPD
YFISKIKNIYKMADVVKTLSYLMMFSGLTMCGYSIFREYKLKKEKRAVVKTVALEMRNNETNGD

>BdioSNMP1f
MRKIWSKWSSPHEYHVYFFDIINPDEIVKGEKLIVKETGPYIYDVWLERIDMVDHEENDTLGYKLKKTFIFNATKSHPLTEEDEVTLVHLVILGTANSLLRSRPSLLPLASKSI
DSLFNKPKSLFTKVSVKTYLFDGFQIDCVGVEEFFAKAVCNDAKEFWFNYRFVQVREDYYTSSFLDTFNNTVAWVGESRINRGIKNITKIGEFVKYNNTNNFAKWDDEKCD
AFVGTDGTMFRPFLSRTDDLHVVNEYLGRSFTYEYDSDVTNSGIHCFRYTASLGTNPETNTNDKCYCLTPDDCLKNGVFETWKATNWPCIISNPHFYLADPFYNGRVTGLN
PNREKHISTIDVHPLTGTVVSAKFRAQWNGPLTKQPKYRLLKNVYETLLPMIWYEEKFTLPARDIRSLKKLSSKIAFEKLFTIMIIVSGLMLFVVGVAYEISLKYKSLKSKT

>BdioSNMP1g
MRINRGIKNIEEIGKVVRYNNTNNFGKWGNEKCDAFVGTDGTMFPPFLSRSDDLHVVHTLTSRSLTYKYESDISYGGFDCYRYVATLGADLETNPDDKCYCLSPDQCLKNG
VFEGWNAVHWPVVGSNPHFCLADPYYIERVSGLNPDPKKHLSTIDVHPLTGIVVGAKFRLQWSAPLTKQPKYRLFENVSEALLPIGWTEERYYLPPPEMENLKKLNFKIVVE
KFLAYHIIAIGFALLFMGILFEVLKRKKPK

>BdioSNMP1h
MMENPKLLRYRVCAAIGLCVSILGVISLEYVIPSMEKRRILKAIALKDGWPARKTWNELSFSNEFRAYLFNITNPDEISNGGKPIVREIGPYVYDVQLEKVEVADHEENDTMS
FKIKRTFYFNATKSYPLSDEDELILTHYLILGTATGVLKTRPTVLPLASKSIDRLFNNPKSMFFNTKVKTYMFDGYELNCVGVEDFFAKAMCSDIEEFWYDYLMVKVRESVY
TSSTMGIWNNTLAWVGETRINRGIRNITKIGEFVEYNHTKNFGKWGDEKCDKFVGTDGTMFPPFLSRKDDLHMVFTTLKRSLTFHYDSDVVHDGIKCYRYVATMGIDLET
NPDDKCYCVEPDRCLKNDLFDTWKAVRFAAVVSSPHFYLTDPYYDDIIVGFHPDKEKHISYIDVHPLTGSVVNSKIRTQWCVPVSKQPKYRVMKNMADTLLPLGWLEDSFT
MPESNIKRLKKLSSMIAMEKICVYVVTAIGLAFFSVGVTCEVLNRRKSKQPN>AccSNMP1
MKPKKLGIIGGSLLAFGILICAIAFPPFLKSQVKKQIALKDGSEMRELWSNFPVPLDFKIHLFNVTNPMEITAGEKPILEEVGPFFYDEYKQKVDLVDREEDDSLEYNLKATWF
FNPSRSEGLTGEEELVVPHVLILSMIKLTLEQQPAAMGILNKAVDNIFKKPSSVFVRAKAREILFDGLPVDCTGKDFATSAICSVLKEKDDALIADGPGRYLFSLFGPKNGTVL
PERIRVLRGIKNYKDVGKVTEVNGKTKLDIWGEGNCNEFNGTDSTIFAPLLTEQDEIVSFAPDICRSMGARFDSYTQVKGINTYHYKADLGDMSSNPEEKCFCPTPDSCLTK

NLIDLTKCVGAPLIASLPHLLGAEEKYLKMVDGLHPNEEEHGIAMDFEPMTATPLSAHKRLQFNLYLHKVEKFKLMKNFPECLFPIFWVEEGILLGDEFVKKLKTVFKTISIV
GFMKWFTIVSGTCVSGAAAALFFKNKDKNKLDITKVTPQKSGEKKWPNQMTISTIQSAAVPPNLDAD

>AdorSNMP1

MIMYIIILFPKILPLFLVVSTAITVEKHEKINNKLLKKHDKRGLTNLEYSLSEPGYHPPEPAIFEPSAPDLSGHLESAIAPVAPPPAVLHPAPAVPVPAVPVPQPVXHPVPVAVPQPV
PHPVPVAVPQPVPHPVPVAVPVTKHVPIPVPIDRAVPVPVDRPVPVPVAVPVPKPYPVHVEKIVHVDRPVPVPFEKPVHVPVDRPVAVPVPVPKPYPVPFEKIVHVDRPYPVHV
AVPYHVPKPYPVPVAIHAHKSHGWLFNVKVLGNHSNSRFFFSKIFEQMHFKKLIYDVTTRFFHRGVHVILHSSFVSLQSFVTTGRKLHETLRVPVGCKRVTMKPKKLGIIGG
SLLTFGILICAIAFPPFLKSQVKKQIALKNGSEMRELWSNFPIPLDFKIYLFNVTNPMEITAGEKPILEEVGPFFYDEYKQKVDLIDREGDDSLEYNLKATWFFNPSRSEGLTGE
EELVVPHVLILSMIKLTLEQQPAAIGILNKAVDNIFKKPSSVFIRAKAREILFDGLPVDCTGKDFATSAICSVLKEKDDALVADGRGRYLFSLFGPKNGTVLPERIRVLRGIKNY
KDVGKVTEVNGKTKLDIWGEGDCNEFNGTDSTIFAPLLTEQDEIVSFAPDICRSMGARFDSYTQVKGINTYHYKADLGDMSSHPEEKCFCPSPDSCLTKNLMDLTKCVGAP
LIASLPHLLGAEEKYLKMVDGLHPNEEEHGIAMDFEPMTATPLSAHKRLQFNLYLHKIEKFKLMKNFPECLFPVFWVEEGILLSDEFVKKLKTVFKTISIVGFMKWFTIVSGT
CVSGAAAALFFKNKDKNKLDITKVTPKKSEEKKWPNQMTISTIQSAAVPPNLDAD

>AdorSNMP2

MWSYQVCAIICLIFGIYAFITNMFSEGLFSIKNIILKNLPLVKDKDTYNAWISPVSLIFKCYFFNVTNPDEVMQGANPNLVEYGPFTYREVYEKQIVDVDEEFDEIIYDIKSTFTF
DKYASVNISKRDTVTILNPAYIGTISMLTTLPPNYMDKFGNNIPKLFPNRNSIFLKANPKEILFDGVKLTCNEKKFPELSTICKTLKALRSPVLKEGEKDGVYYLSIFQRVNGTI
RGRFSVNRGINNISELGNIASYNGKKVQTIWKTEKCNIVRGSDTITWPPLINPMPSVLTFIPDLCRSVEVDYDKKVSIYGLTGFRFAMKERTWFLNTSQCYCLEKNKVPNCLP
QGLIDVSNCLVMLRYENEEISLVYVLKFNMLQKVPIIISEPHFLHGDPQLLMYAHGLNPDKDLHETFVVIEPYTGTPLSGQKKIQLNLKLERQPVNLLSNISEGYFPLLWCSNV
RIFSKIIIFQH

>AfloSNMP1

MRFKKLVYDVTSRFFHRGVHVILHSSFVNLQSFVTTGRKLHETLRVPAGCKRVTMKPKKLGIIGGSLLAFGILICAIAFPPFLKSQVKKQIALKDGSEMRELWSNFPVPLDFKI
YLFNVTNPMEITAGEKPILEEVGPFFYDEYKQKVDLVDREEDDSLEYNLKATWYFNPSRSEGLTGEEELVVPHVLILSMAKLTLEQQPAAMGILNKAVDSIFKKPSSVFVRAK
AREILFDGLPVDCTGKDFATSAICSVLKEKDDALIADGPGRYLFSLFGPKNGTVLPERIRVLRGIKNYKDVGKVTEVNGKTKLDIWGGDCNEFNGTDSTIFAPLLTEQDDIVS
FAPDICRSMGARFDSYTQVKGINTYHYKADLGDMSSHPEEKCFCPTPESCLTKNLMDLTKCVGAPLIASLPHLLGAEEKYLKMVDGLHPNEEEHGIAMDFEPMTATPLSAH
KRLQFNLYLHKIEKLKLMKNFPECLFPVFWVEEGILLGDEFVKKLKTVFKTISIVGFMKWFTIISGTCISGAAAALFFKNKDKNKLDITKVTPQKGEEKKWPNQLTISTIQSA
AVPPNLDAN

>AfloSNMP2

MWSYHVYAIICLIFGIYVFTTNMFSEGLFNIKYSILKNLPLVKGKDMYNAWILPVSLIFKCYFFNVTNPDEVMQGDNPNLVEYGPFTYREIYEKQIVDVDEELDEIIYNVKSTF
TFDKYASVNISKHDTVTILNPAYIGTISMASIIGLTNLPSNYMEKFGNNIPKLFPNRSSIFLKANPKEILFDGVKLTCNERKFPELSTICKTLKATRSPVLKQGEKDGVYYLSVFQ
RINGTIRGRFSVNRGVNNISELGNISSYNGKRVQTIWRTEKCNTVRGSDTITWPPLVNPLPMVLTFIPDLCRTVEADYDKEVSIYGLIGSRFVMKERTWFMNRSQCYCLERNK
VPNCLPQGLIDVSDCLKVPIIMSEPHFLHGDPRLLMYARGLNPDEDLHETFIVIEPYTGTPLSGQKKIQLNLKLERQPVDLLSNISEGYFPLMWCANVRIFSKIIIFRH

>AgifSNMP1
MPLWRKLAIAGGSMLAFGILVGFIVFPPFLRKQVKKQIALKPGAEMRTLWSQTPFPLDFRIYLFNITNADEIKTGAKPIVQQVGPFFYDEWKEKVDLVDREEDDTVEYSNKA
TWIFNKKKSGPGLHEDMELVFPHIMILSMVYATVRERPGMVGLAAKAVDSIFHKPDSVFVKATVREILWTGLPVDCSVQDFAGKAVCGILREDDSSLLKDGETNYKFAIFGP
KNGTVVPDRLRVKRGVKSYLDVGIVTEYKGEPKLSNWLEDSDCNLFNGTDSTIFHPFLYQDEDVVSFAPDLCRSLGARFSHHSSVKGLKTNHYTAELGDMSKDPELKCFC
QTPETCLKKGLFDLFNCVKAPLVASLPHLYLVDESYLEQVDGLHPNKDEHGIFLEFEPMTGSPLSARKRLQFNMFIHPVEKFKLLKTAPTALLPLFWVEEGILLGDDIIGQLKT
LFKVMSIMTILKWTMTLLGIGLIGGAGGLYYKDSQASQKLEITKISPNSLTNGNKNWPLDVNGLQSNTVPPALDG
>AmelSNMP1
MRFKKLIHDITTRFFHRGVHVILHSSFVKLQSFVTTGRKLHETLRVPVGRKCVTMKPKKLGIIGGSLLAFGILICAIAFPPFLRSQVKKQIALKDGSEMRELWSNFPVPLDFKI
YLFNVTNPMEITAGEKPILEEVGPFFYDEYKQKVDLVDREEDDSLEYNLKATWFFNPSRSEGLTGEEELIVPHVLILSMIKLTLEQQPAAMGILNKAVDNIFKKPESVFVRAK
AREILFDGLPVDCTGKDFASSAICSVLKEKDDALIADGPGRYLFSLFGPKNGTVLPERIRVLRGIKNYKDVGKVTEVNGKTKLDIWGEGDCNEFNGTDSTIFAPLLTEQDDIV
SFAPDICRSMGARFDSYTKVKGINTYHYKADLGDMSSHPEEKCFCPSPDSCLTKNLMDLTKCVGAPLIASLPHLLGAEEKYLKMVDGLHPNEEEHGIAMDFEPMTATPLSA
HKRLQFNLYLHKVAKFKLMKNFPECLFPIFWVEEGILLGDEFVKKLKTVFKTISIVGFMKWFTIVSGTCVSGAAAALFFKNKDKNKLDITKVTPQKGEEKKWPNQMTISTIQ
SAAVPPNLDAD
>AmelSNMP2
MWSYQVCAIICVIFGIYACITNLFSDGLFSIKNAILKNLPLIKGKDMYDEWILPVNLIFKCYFFNVTNPDEVMEGNNPNLVEYGPFTYREVFEKQIVDVDEELDEIIYDVKSTF
TFDKYASLNISKRDTVTILNPAYIGTISMASIIGLTTLPPSYIEKFGNNIPKLFPNRSSIFLKANPKEILFDGVKLTCNERKFPELSTICKTLKALRSPVLKEGEKEGVYYLSIFQRV
NGTIRGRFSVNRGVNNISELGNIGSYNGRRVQTIWRTEKCNTVRGSDTITWAPLINPMPSVLSFIPDLCRSIEADYDKEVSIYGLIGSRFVMRERTWFLNQSQCYCLERNKVP
NCLPQGLIDVSDCLVMLRYVMLQKVPIIMSEPHFLHGDPQLLMYALGLNPSEDLHETFIVIEPYTGTPLSGQKKIQLNLKLERQPVDLLSNISEGYFPLLWCANVRIFSKIIKLQ
Y
>BimpSNMP1
MRLKPLAHDFAARVFRRGVKVTWYSSLLGLLSFLTTGRKLHETLRAPTGHKRVTMIKPKKLGIIGGSLFA
FGVVFCAIVFPPFLKWKIKNQVTLKPDTEMRELWSTFPVPLDFKIYLFNVTNPTEITAGEKPILEEVGPF
FYDEYKHKVNMVDREEDDSLEYNLKATWFYNPSLSSPLTGEEELVFPHVLILSMVKLTLEQQPAAIGILN
KAVDNIFKKPSSVFVRAKAREILFDGVPLDCTGKDFASSAICNVLKEREDALLPDGPGRYLFSLFGPKNG
TVLPDRIRVLRGVKYWKDVGKVTEVNGKPELDIWTEDHCNEFNGTDSTIFAPLLTEQDDIVSYSPDICRS
LSARFDHKTKVAGINTYHYKADLGDMSTNPEEKCFCPAPDNCLTKNLMDLTKCVGVPLIASLPHLLGSEE
KYLEMVDGLHPDEEKHGINMDFEPMTATPLIARKRLQFNMFLNKVEKFKLMKNFPECLFPFFWVEEGIEL
GGEFLAKLKMVFKMISIIGFMKWLTIISGACVSGAAAAIFFKNKDKGSLDVTKVTPQSQNGKNEDEKKWP

NQMTISTIQSAAVPPNLDAN
>BimpSNMP2
MSPNNRQTDNFKEKVRSNFYKMWCYYGCAIIWIIFGLYVSQTELFSNKLISKITEALSLREDSKIYKAWK
SPMQLTFTCHFFNVTNPDEVMSGSNPYFNEVGPFTYDEILEKQIIDVDDAMDEITYTTKSMYSFNKDLSV
KLSEHDKITILNPAYIGTMSMLSSLPAGYIEKYGNNIPKLFPNRSSIFLKANPNDLLFNGIKVSCDLRKF
PELDVICKTLGSNLPQVLRKTDKQDVYLLSIFQRINATFRGPFSVNRGVNDIMRLGDITSYMGKRKQKIW
NSEDCNIIRGTDSIIWAPLIKPLPFVSTFIPDLFNIPYQLSRCMCRTIEADYKDEISVRGLIGSRFVMKE
RTWFLNTTQCYCLLENKIPKCLPQGLIDVWECQKLPVILSEPHFLHGDPQLLKYAGGLNPDDRLHETYII
IEPYTGTPLSGQKRMQLNLYLGKQSVELLSNVSEGYFPLIWCGNRMW

>FariSNMP1
MQRWLKLGIAGCCLFIFGIVFGFVIFPLLVKSQVKAQVNLKPGTEMREMWEKTPFPIDFRIFLFNITNPD
EIIAGAKPIVNEVGPFFYDEYKEKVEVTDVEKDDTSEYSNKITWQFSPEKSGMGLSEDTVLTFPHVMILN
MILAVMREQPGTISFATKAVDSIFHKPNSVFIRTTVREILWTGLPLDCTVQDFQGKSACLLLAEKEAAFI
KDGPGKYRFALFGAKNATTVKDRIKVRRGLQNYLQVGEMVEFKGEKVQEIWGEEGSCNNLTGTDSSIFHP
LLYEDEDMISFSVDMCLTVPARYVKPSNVQGVPTNHYVADYGDMNKDEHLKCLCPAPDKCLKKGLIDLFP
CIGAPLVASLPHLYLVDDFYLTQVDGLHPEKEEHQIFINLEPMTGSPVEARKRLQFNMFIRPVEKFPLMK
TFPQALLPLFWVEEGLLLGDKYVNQLKTVYTLISIVGIIKWLLILGGLVLIGTAGYLRRRIKKGSKIDIT
TISPQFTATALNSIDKKQSDLNTSQSAPADS
>FariSNMP2
MSSIKLNICFGFFLGLGIVVAILALVSKFIVFPKIINNEIAEEIQLINGSEAYDRWVKIPFPIIYKAYFF
NVTNPEAVSQGEKPILQEVGPYIYQQHRAKLNITDRLETLGYNEHQLFEFDAKASYPLTDSDPIRIVNLP
LMGLIDTTERETPALVRFLDLIIPQLFDNPKNLFLTTTPKDFLFDGIFLNCQSNNVLLRIFCKSIEKLAP
RTMVRQEDGNIKFSFLSYKNNTDEGRFVVNSGRIEPERVGQLSSWNGYNLTNIWLTNSSCNRIFGTDATI
YPPFDSTDSILDTFSIDLCRTVSLEYSEPIVYSGIDGLRFVAKKSTFLSPGQEPLNQCYCLHKTKGILGQ
DGCLLDGALELFSCHGAPIVFTHPHFYLTHQTYQNGVIGLKPNSIKHQNFVDLQPNTGAPLRGSKRGQFN
IFLRSVDGVSLTSGLRTTLMPILWFDEGVVLSDKYVDILKVDFLDKMRFIEIGGWAIFACGTATCILSFV
VFISCRCAKKNS

>MdemSNMP1
MKEMWVKVPFPVDFRIYLFNITNANEIKTGAKPIVQQVGPFFYDEWKEKVDLVDREEDDTVEYKNKATWV
FNQAKSAPGLTEDVVLVFPHVMILSMILATVREKPAMVGLAAKAVDSIFHKPDSVFVTATAREILWTGLP
VDCSVKDFAGSAVCGILREDDSGFLKDGENYKFALFGAKNGTVIPDTIRVHRGKRNYLEVGIVTEFKGEP
KLNVWPEEGDCNTFNGTDSTIFHPFLYQDEDVVSFAPDLCRSLGARYQRPSKVKGIKTNRYTATLGDMST
DPALKCFCPTPDTCLGKGLYDIFPCVKAPLVGSLPHFYDTDPQYLTQVDGLHPNEEDHQIFIDFEPMLGA
PLSARKRLQFNIFIMPVEKFKLMKTFPNALLPLFWVEEGLLLDDEYLKPIKMVFTMLKIVGIMKWLMMTA
GVGLGGGAGFLFWKSTQSPQKLDITKVSPKTIQNAGGDEKKWPTTVSTIQGNNGPPSVEA
>MdemSNMP2
MNYCRTGFRNFLSYVPGLLLISLGIYLATEKPHTNYVIDQIRKEAELTEGKYGYRMWKDLDIYFKVYLLH
VTNPDNVMEGENPIFEERGPYVYNLNMKKQVTHVDEKLDELAFTIFRTYQFNKNASGSFSEDDQVVLLNS
AYLGTLNTIVSKFPAFIGRFGNSIQNLFPKTYDVFLRSKVKDILFDGLPLICDPVKYKDLALLCNFLKGQ
KPPIIKNTDKPGVYSYSLFDKNNYTDSDPFTVNRGVENKDALGNTTSFKKLRVTKYWTEKECNLVSGTDS
ITWAPMTEKLPFVSVYEPNVCRRMTPNFKREVIINGLMGYRYELDETTWLKKNMGCYCLPNAKKVPECLQ
TGLLDITKCQVTLLFIYITRDAPVIFSEPHFLHADPNLLEYARGLKPDPIEHTTFITIEPLSGAPLSGSK
KIQLNLNVKRIPGITLLANISEGLFPILWAEEVFYLFIFINKQLSQ

>MpulSNMP1
MKLFMKLGIAGGAMFAFGILIGFIIFPPFLKSQVKKQSSLKAGSELREMWTTLPFPLDFKVYLFNVTNPE
EIAAGAKPIVKEVGPFFYDEYKNKVNLVDDEEDDTVEFSFQKTWYFNQAKSGPGLTEYTEIIFPHMLILG
AVMTTLRTQPTMVGAVGKALDSVLHKPESVFMKTTPRELLWEGMLIDCTVKDGAGKALCKELRKDDSGLL
KEGENYRIALFGHQNGTASRDRIKVKRGLKQILDVGVVQTFNNKTKLDTWVEKGNCNTLNGTDSTIFHPF
LYDNEDIVSFSSDICRSVSARFQQKSSVGGIKTNRYTASLGDMSKDPEIKCLCPTEDTCLKKGLMDLFNC
VKAPIVASLPHLYQVEKEYLSQVDGLHPNEHEHELFIEFEPFTGSPLSARNRLQFNMFIQNVTKFKLMKT
FPSALMPLFWIEEGILLGDDILSQIKMVFTLMSVVSGMKWTMITLGLGLGGAAGGLFYKAQQNSQKLDIT
KIVTKSSQNGGEKQWPVNISTVQGATAQPHLDS

>MrotSNMP1
MKPKTLGIVGGSMLSFGILICSIIFPAFLKSQIKKQVALKPGSEMREMWTSFPLPLDFKIYMFNVTNPTE

ILGGAKPIVEEVGPFFYDEYKHKVNLEDREEDDSVSYNFKTTWYWNPSKSNGLTGEEELVVPHLFIISMI
KLTLMEQPSAIGIVNKAIDSIFKKPGTVFVRAKAKDILFNGLPIDCTVKDFAGAATCNVLSQKAEESGLI
SDGEGHYLFSIFGSKNGSVVPERLRVLRGMKNWKDVGRVIEYDDKPALTTWPEEHCNQYNGTDTTIFAPF
LEEGGDIVSFAPDLCRSLGAHYSHKSVVKGVHTYHYTADLGDMSKNEDEKCFCPTPDTCLTKNLMDLYKC
IGAPLIASLPHLFGSEKKYHEMVDGLHPNEEAHGIGMDFEPTTATPLLAAKRLQFNVFLHPMEKVKLMKN
FPECLFPLFWVEEGILLDDEYVKIVKKLFMIKGIVGFLKYLTIFVGTCASGAAGALYFKNKDKNKLDITK
VTPQSQKENDDEKKWPPQMNISTIQSATVPPNLDN
>MrotSNMP2
MSNLYLMIFQIFIGISLICLGSYFVSENVLETLVVKFVQKFSLPLIKGSPHYEIWSKPTSINFSCYLFNV
TNPDEVMRGENPHLVEYGPFTYTEVQEKFISYIDKEMDEIKYTTKSTYTFDRYQSLNFSKQDKIIILNPA
YIGTIETLAALPDDFMKKYGNSIPKLFPNRSSIFLKARPTDVLFDGVKITCNPKKFPELQLICETLKLKQ
PPVLREGEKENVYYLSFFQRLNNTSRGPFTVSRGVKDITKLGDITSYLGYRVQEVFATDLCNTVRGSDTI
TWAPLMKPLPKVSTFIQDICRTVEIDYENEVVLNGMIGSQFVMHERVWYLNESECYCPLVDKQPVCPRRG
LIDAYQCQKVPVFVSEPHFLHGDPELLNYARGLTPNEVLHKTYVVIEPYTGIPLAGEKKTQLNLKLARRP
VNLLANISEGYFPLLWFENGNKLTPFDQVLYPYQLLRYLHFMRYLQYIPLIIGIYLISISLLFYGDTSRR
VHPNATNVQSILISNSRLNRPPQRQVVW

>MmedSNMP1
MLLFKKLGIAGGSVFTLGIIIGYAFFPPFLKSQIKKGIQLVDGSDMKEMWVKVPFPLDFRIYLFNITNANEIKAGAKPIVQQVGPFFYEEWKEKVDLVDREEDDTVEYKNKAT
WVFNQAKSAPGLTEDVVLVFPHVMILSMILATVREKPAMVGLAAKAVDSIFHKPDSVFVTATAREILWTGLPVDCSVKDFAGSAVCGILREDDSGFLKDGENYKFALFGAK
NGTVIPDTIRVHRGKRNYLEVGIVTEFKGEPKLNVWPEEGDCNTFNGTDSTIFHPFLYEDEDVVSFAPDLCRSLSAIYQKPTKVKGIKTNRYIASLGDMSTDPTLKCLCPTPD
TCLGKGLYDIFPCVKAPLVCSLPHFYDTDPQYLTQVDGLHPNEEDHQIFIDFEPMLGAPLSARKRLQFNIFIMPVDKFKLMKTFPNALLPLFWVEEGLILDDEYLKPIKLVFT
MLKVVGIMKWLMMTAGVGLGGGAGFLFWKSTQSPQKLDITKVSPKTIQNAAGDEKKWPTSVSTIQGNNAPPSVEA
>MmedSNMP2
MTSCSTRFRNFMSYVPGLLLISLGIYLATEKPHTNYLINHIREAAELVEGKYGYEIWKDLDIYFKVRLYHITNPDSVMEGENPIIEERGPYVYNLNMKKRVTNVDEKLDEMA
FTIFRTYQFNKNASGSCSEDDQVVLLNSAYLGTLNIIASKFPAFIGRFGNGIQYLFPKSYDIFLRGKVKDILFDGLPLVCDPVKYKDLSLLCNFLKGKKPPIIKDTDKPGVYSYS
LFDKNNYTDSDPFTVNRGVENKDALGNTTSYKNLRVTKYWREKECNLVSGTDSITWAPMTEKLPFVSVYEPNICRRMTPNFKREIIVNGLLGYRYELDETTWRKENMGCY
CLPNAKKVPECLETGLLDITKCQDAPVIFSEPHFLHADPNLLEYARGLKPDPIEHTTFITIEPLSGAPLSGSKKIQLNLRLNRIPGITLLANVSEGLFPILWAEEVFYLFIYFFFFLS
INS

>NvitSNMP1
MALTKIQKIGVGGICMFIFSFLFSGVILPPIVKHEVKKKVALKQGWMMREVWGKFPFSFEFHFYMFNVTNHMDIKGGAKPIVAEVGPFVYEEWKEKVNQVDHDEDDTISY
NAKSTFIFNAEKSKGLTGEEEVIMPHFFILGTVNSVLRDKASAMPIVSKALDSIFRKPDSIFVKAKVREILFDGIVIDCNVKDFAGSAVCNEIAQNYEEFRLQSIGDNKYSLSLF
GLINGTENKARHRVKRGLKNIMEVGKVVEYDGKNNVSVWDNEICDAFNGSDGSVFHPYFDKKGKDDLVAFNADLCRSVICHYDSDTKFAGLKLLRYTTDLGTDVEKYPH
HKCYCVTPDRCPKKGAMDIFKCVNAPIMITNPHFYLADPWYVSAIEGVKPDREKHMIMIDIDPFTGSPIHVHTRAQFNMFLQPVEKFKLMKTFPNALLPLIWFDEILILPDFL
LKEIKGGHRQVAMAKVFKFLMMFGGLGMAGYAGFMHYKATQGENTTEVKKVPVKSSPNGVGSGEKKINISTIQPAPLPPNVD
>NvitSNMP2
MSLRICRSVCVAFGFILAGVGLLLGFYVFPLLVNQQVDDTMKLVNGTEAYERWETLPIPLQFKVYFFNVSNPDEVQNGAKPIVKEVGPYVYDEYRHKYDITEDEDGTYSYN
QTQLFSFNENASKPNKEDDNIIVAHLPLMAISLIAEKRLMSELLGTVVPHLFDNPKNVFLTTTVKKFLFDGVNINCSNGGGTVRLICNQIRRNAPAQLKVPDKGVDGPFVFSL
LSYKNNTHDGRYKVSSGVKDISTLGEIYAWKNSSTVDAWKPNGTCNNIYGTDTTIFPPHRTQLSRVNVFQSDICRTVNLHYNDETEYKNIKGLRFVVEKDMLMSGANYSAN
KCYCLKETKGINGEDGCLLDGALELYRCQNVPLVLTFPHFYLAHEKYRESVEGLNPDKSKHEIFVELEPKSGTVLRGSKRVQFNIFYRPIHGINLTNKLAHSLMPVFWIDEGV
ELDDPNINLLYDSLVYPMKILDGVYWTLIGLGLAIGLISMVWCMLFAHKPKHLF
>DmelEMP.B
MQLDDILHINNCKADCSSLSTTPNPKTDLVNMNGPKHKFCTKLSSTYLRKWWITIVVAAALIIGGIVVAC
EFTVLIDAVVDRMVALRPGAKTFGWWAKPPVEPRISLYIYNVTNADDFLSNGSKAIVDEVGPYVYSETWE
KVNIVENDNGTLSYNLRKIYSFREDLSVGPEDDVVIVPNIPMLSATSQSKHAARFLRLAMASIMDILKIK
PFVQVSVGQLLWGYEDPLLKLAKDVVPKEQKLPYEEFGLLYGKNGTSSDRVTVNTGVDDIRRYGIIDNFN
GRTHLPHWTTDACNTLAGTDGSIFPPHIDHDRILHVYDKDLCRLLPLVFEKEVMTSNEVPGYRFTPPEWV
FADVDSHPDNMCFCPAGKPSCSPNGLFNVSLCQYDSPIMLSFPHFYLADESLRTQVEGISPPMKEKHQFF
FDVQPKMGTTLRVRARIQINLAVSQVFDIKQVANFPDIIFPILWFEEGIDNLPDEVTDLMRFAEQVPPKI
RVALIVGLCALGVILLLLSTFCLIRNSHRQSTLHLEGSNYLATAQVDMNKKQNKDNQPARY
>DmelEMP.A
MNGPKHKFCTKLSSTYLRKWWITIVVAAALIIGGIVVACEFTVLIDAVVDRMVALRPGAKTFGWWAKPPV
EPRISLYIYNVTNADDFLSNGSKAIVDEVGPYVYSETWEKVNIVENDNGTLSYNLRKIYSFREDLSVGPE
DDVVIVPNIPMLSATSQSKHAARFLRLAMASIMDILKIKPFVQVSVGQLLWGYEDPLLKLAKDVVPKEQK
LPYEEFGLLYGKNGTSSDRVTVNTGVDDIRRYGIIDNFNGRTHLPHWTTDACNTLAGTDGSIFPPHIDHD
RILHVYDKDLCRLLPLVFEKEVMTSNEVPGYRFTPPEWVFADVDSHPDNMCFCPAGKPSCSPNGLFNVSL
CQYDSPIMLSFPHFYLADESLRTQVEGISPPMKEKHQFFFDVQPKMGTTLRVRARIQINLAVSQVFDIKQ
VANFPDIIFPILWFEEGIDNLPDEVTDLMRFAEQVPPKIRVALIVGLCALGVILLLLSTFCLIRNSHRQS

TLHLEGSNYLATAQVDMNKKQNKDNQPARY
>DmelCroquemort
MCCKCCGETQRKVWVFGLGSVFLLLGILIVVFWPGIADNLVEDGLTLKPGTDAYESWLEAPIPIYLSFYM
FNWTNPEDIRNPDIKPNFVEMGPYTFLEKHKKENYTFYDNATVAYYERRTWFFDPERSNGTLDDMVTAAH
AITATVADEMRDQRKIVKKIINFMLNHEGGKLYVTKPVGEWIFEGYQDNITDFLNLFNTTKIDIPYKRFG
WLADRNESLTYDGLFTIHTGTDDISNLGRLTHWNGKSETGFYEMPCGIVNGTTGDMFPPKMNVNDEITIF
ATDACRFMNLRPRGTYENHGLTATKWVGTEETLDSGENYPNQACFCDEARFDECPKTGVVECKACRDKAP
IYSSFPHFYLADQSYVDAVSGMKPEKEKHEFFLAVEPITGVPVQVHGRIQINMMIEPDDDFDIYRGVQKV
LMPMFWFDQYAELSSELASKAKLAINLSSYGIIFGYSMIAFASVFLITGITLTVTKKWVRRTPEDEDILT
N
>TcasCroquemort
MPCCTPTCRKWSVFGFGTFFLVLGVAIIVFWNSLVQSIKDQELNLGNDGTTEYKMWKETPIPMYIEFYLY
NWTNWKEVVDSKWSLKPSFEEHGPYTYNEKHIRKNVIFNDNHTVTYKTQRIWHFAPEKSKGSLDDVITTL
NPILVTVGSMVKYKHPIVKMGVNFFIKEKGVNLTVTKTAREFIFDGYDDPLLDLLKKLHMRHINIPFDKF
AWFISRNESIDYDGIYNMYDGTDDVRKLGRFAWWNYNNQTEYFPNYCGEVNGTSGELWYPVENDQYAEVF
SPDTCSTLTLVKQGTEELHGVVGHKFVGDEKLFDNGTRYPDMRCFSPGDVLPSGVRNVSHCKFGAPAFIS
YPHFYLADPYYREAITGMTPNKTEHELFISIEPETGIPLHARVAAQINLHLEKIDRITLLEHVGREYLIP
AMWFKQYAVLSEDLANQAKMLIILPAVGQYTGIGAVALGSLLLSIFGFLTWKSYRKGREEETLLNQEEF
>CquiCroquemort
MCCRCSNKAKRWWSLGLSALICLFAIALGIAWPLRVDSAIKKQFVLQPGTEIYDSWLAPPVDTHLELYLW
NWTNAEEDYTLAGYKPSLEQLGPYTFREIHERSNVSWHDEDFSVTYYQKRIWHYEPQLSRGDLENDIVVT
INPILLTIGFTLKDNPFLLGFIDLIINENKEEMDISPLYKVTAKDILFEGYDDKLLKNLLDVVASNPGLA
DQIDLPPFDRFGWFYGRNESELYDGNFTIGTGVDALDNLGMMRLWNGLDRTPYYRDECGRVVGSSGELWP
PYQEPERPNVTVFSSDICSAMTLEFDGAFSLHGVDGFKWKGNDKPFDNGHNYAETSCQCTAVEEECPVLA
PGTMDVSSCKLGAPATVSYPHYYLAHPSYREAVEGMTPSKADHEFMMALEPTTGIPLAVKAQLQVNLDVK
QYGLTIFQGIPNVMLPVLWFRQTAQLTEELASDLKLLLILPNIGVYVAIALGVVGVVGFAVSLYCSLKVW
KD
>DmelPeste
MTSRTRHCARLGIVLLGICCIASGIYLFRNWIDMFTRMRGQEMALSPNSRSFEGWKVSPLPLDFDIYLFN

WTNPDDFYVGSNKKPHFEQLGPYRFREKPDKVDIEWHNHNASVSFHKKSWFYFDAAGSNGSLWDKVTTVN
SVAHSAARRAAVDWFARTGVNIANKLYRQGVTITKTVDEMLFKGYEHPFISVGKLLRPQDVPYKRIGYHY
PRNGSSEFDGDINMFTGADDIAKMGQIHTWNNLTHTGAFEGTCGQVHGSMGEFFPPNLGTKDTVYMYMPK
MCRAIPLDYVETVTVHGVTAYKFSGTRHAVDNGTLYPDTRCYCVGGKCMPSGVINIGPCSFNASVYMSFP
HFYMADPSYLEAIEGLRPEREKHEFFMALEPNAGVPMDVGGGFQANYYMEPIPGITLYENVPTVMIPMMW
CEERVRVSEEIAADIALVPLIVLLGQIVTGILLAGGLICTCWYPTRQVTHFCHSDPKAKASVLRPLNAFG
VNSSAATAPVAQLFRNNISSSGNERVGVRLLDYNRDSGIRLESGTMESSHRERLISEDSPDVVVR
>AaquPeste
MLQTAPHGVQATAADTEKGATYHPLGPVIASNGHHTNDTAPSVTKVSKSAYSQARKPTTTMLVKEFCNRR
CILTSAGLLLIASGTFFILFFPVIFTDILHEELKLRPGSRGYDAWVSPPFPLAMDVYFFNWTNPEDLTNH
STKPILEELGPYRFIEHPTKVDIEWHDANATVSFRKKSIYYFDEEGSNGTLDDMISTINIVAVSAASKAK
YWGYLQQKGVSMGFTLYDQQINVVKTAGELLFDGYEDNMVLMGKHMFQDVEIPFDRVGWFYTRNNSADLI
GHYNMHTGADDITKLGSMGEWNYRPRTDFFEDGCGMLNGSAGELYPPGLSKERPVELFTPDMCRTLPLDF
EEEVTVHGVKAYKYSGGERAVDNGTLFPETACFSSGEIVPSGVLNISSCRFGTPVFVSFPHYYGADPYYL
DQVEGLSPTKEKHQFFMSMEPTTSVPLDVAARLQLNIMIEPYENVGIFSGVKRVFLPVLWFEQHVIMPPE
LSGEIAFALTIPSIVRMTGIVMCVCGIAMLFWIPLERMIFRGRRVVAIGNKPAPAGAFNGVHAFANGAEK
GLLHANEKNGKPLTTLLLEKTEKQPLPECIGDKRAEAECFPLIDGKGATIVKS
>DbusSanta-maria
MPAQKKSIWRTGCNRGYITCISGFLLTVLGIYCGMFWEDIFNWIKHKEMALAPETRAYYNWKTPPIDLHL
DIYLYNWTNPEEFYGNSSSKPVLEQLGPYRFVDRPDKVNISWHPDNNTVTYKRRNFYYFDAAGSAGSLDD
VIVTLNAVALSAAATAKQWNSMRRSMVDVGLKLYGQQMSVVKTVDEILFTGYSDAMIDMAIAMPSGMFGD
EINVPFDKFGWFYTRNGSADLTGVFNVFTGADEISKLGQMHSWNYKTHTGFFESDCGLVGGSAGEFQPPQ
AQPGGSVSLFTADLCRSLPLDYVETVQIEGLKGYKFAGGPRSVDNGTLFPENLCYCGGECSPSGIMNISA
CRFDSPVFMSYPHFYQADQFYVDQVEGLQPDQQAHEFYMIVAPDTGIPLEVAARFQVNMLVEPIDGISLY
TDIPRIFFPLIWFEQKVRITPELADQLKLLPMVLLGGHIFAGFCLAIGLILLCWLPMKLLLSLCRSREYD
VKMQHKTNGQYKSRDDSSSEKLPADSEKIAPDSSPLLDEAQRKLPVLINSPTKDSGISMQLKETP

>DmelSanta maria
MPTQNSAMWGQKSNRKLIIGIFGFCLGLFGILCGMFWVDLFDWIMHKEMALAPDTRVYENWKSPPIDLSL

DIYLYNWTNPEDFGNLSTKPILEQVGPYRFIERPDKVDIHWHPENASVTYRRRSLFYFDAAGSNGSLDDE
ITTLNAVALSAAATAKYWPPVKRSLVDVGLKMYGAEMSVQKSIDELLFTGYNDAMIDVAMAMPIFGDEVK
VPFDKFGWFYTRNGSADLTGVFNVFTGADQLAKLGQMHSWNYQENTGFFDSYCGMTNGSAGEFQPQHLKP
GDSVGLFTPDMCRTIPLDYVETVDIEGLEGYKFSGGPRSVDNGTQYPENLCFCGGQCVPSGVMNISSCRF
GSPVFMSYPHFFNADPYYPDQVEGLSPNQKDHEFYMVVQPSTGIPLEVAARFQVNMLVEPIQGISLYTGI
PRIFFPLVWFEQKVRITPDMADQLKVLPIVMLSGHIFAGICLIVGITLLCWTPVQILLASCRNRRYDLRT
KTKTNGQYKSRSQFSSAEELKSKASTLVCEKSVKGSPDSSPLLEKGRKPTIIKSQTGESVATASTAISDN
KQD
>DmelDebris buster
MGLEKHYLRYGRTARDHLLDWATCGRGRRQQQQQQQQTHAQPSQGSTATGRRSRPHVTHRGTPLSMLIS
NGVRISNNRLAVIIIGIITLILGIILSSMPWLDYFILKNLRLWNDTLSYHYWQRPGVIRLTKLYIYNVTN
PDGFLRGEKPHLQEVGPFVYREDMQKVNVKFHENNYTVSYQHKKILQFVPELSIDKDTPITTPNIPLLTL
TSLSPKLGYLLSKTISVVVTAAQFKPFINVTAEQLAFGYDDALVSLAHRFYPKHMRPMERMGLLLGRNGT
LTEVSSVKTGMDSMDQFGYIDQLNGLDHLPHWSEPPCTSIAGSEGSFFPPRELTKSEVVHIYDKDLCRII
PLKYVESLEKDGIAADLFRLPNNSYGDSAHNPENKCYDTSEYEPIQGLQNISPCQYGAPVYISNPHFFES
HPDLLNSVEGLKPEREKHETYFKIQPKLGVPLEGKVRIQLNLKVTRAKDVYPVRDFRDFVFPVMWLEEGI
SELTPAIKRWIYLGTVIAPSAVPIGSYLMILGGAFAIIFSFVRAYQNFMFAQDPTLEILEMGRRSLRRGS
SFIAHQQHRLLVHHRDSYSLLRHGPMATCLGEGNREEDAQPIIDVSLSGGISQES
